# Identification and localisation of the NB-LRR gene family within the potato genome

**DOI:** 10.1186/1471-2164-13-75

**Published:** 2012-02-15

**Authors:** Florian Jupe, Leighton Pritchard, Graham J Etherington, Katrin MacKenzie, Peter JA Cock, Frank Wright, Sanjeev Kumar Sharma, Dan Bolser, Glenn J Bryan, Jonathan DG Jones, Ingo Hein

**Affiliations:** 1 Cell and Molecular Sciences, The James Hutton Institute (JHI), Dundee, DD2 5DA, UK; 2 The Sainsbury Laboratory, Norwich Research Park, Norwich, NR4 7UH, UK; 3 Information and Computational Sciences, The James Hutton Institute, UK; 4 Biomathematics and Statistics Scotland (BioSS), The James Hutton Institute, UK; 5 University of Dundee, College of Life Sciences, Dundee, DD1 5EH, UK

## Abstract

**Background:**

The potato genome sequence derived from the *Solanum tuberosum *Group Phureja clone DM1-3 516 R44 provides unparalleled insight into the genome composition and organisation of this important crop. A key class of genes that comprises the vast majority of plant resistance (*R*) genes contains a nucleotide-binding and leucine-rich repeat domain, and is collectively known as NB-LRRs.

**Results:**

As part of an effort to accelerate the process of functional *R *gene isolation, we performed an amino acid motif based search of the annotated potato genome and identified 438 NB-LRR type genes among the ~39,000 potato gene models. Of the predicted genes, 77 contain an N-terminal toll/interleukin 1 receptor (TIR)-like domain, and 107 of the remaining 361 non-TIR genes contain an N-terminal coiled-coil (CC) domain. Physical map positions were established for 370 predicted NB-LRR genes across all 12 potato chromosomes. The majority of NB-LRRs are physically organised within 63 identified clusters, of which 50 are homogeneous in that they contain NB-LRRs derived from a recent common ancestor.

**Conclusions:**

By establishing the phylogenetic and positional relationship of potato NB-LRRs, our analysis offers significant insight into the evolution of potato *R *genes. Furthermore, the data provide a blueprint for future efforts to identify and more rapidly clone functional NB-LRR genes from *Solanum *species.

## Background

Plants have evolved a sophisticated, multi-layered defence network to detect and respond to pathogen challenges. Inducible responses are governed by plasma membrane pattern recognition receptors (PRRs) and also cytoplasmic immune receptors encoded by resistance (*R*) genes. PRRs recognise relatively conserved small molecules, proteins and protein fragments, produced externally to the cell by invading pathogens, and collectively referred to as pathogen associated molecular patterns (PAMPS). By contrast, R proteins directly or indirectly perceive proteins and small molecules termed effectors that are introduced into plant cells by the pathogen. Genes encoding effectors that are recognised by *R *gene products, leading to effective plant resistance, are genetically defined as avirulence (*avr*) genes. Two modes of resistance may be distinguished: PAMP triggered immunity (PTI) that is mediated by PRRs, and effector triggered immunity (ETI) that results from effector recognition by R proteins and often produces a hypersensitive response, a form of localised host programmed cell death [1]. *R *genes have been implicated in resistances against diverse and taxonomically unrelated pathogens including bacteria, viruses, nematodes, insects, filamentous fungi and oomycetes. In addition to being pivotal for host resistance, PRRs and *R *genes are thought to play a role in non-host resistance [2].

The majority of cloned and functional *R *genes described within the plant kingdom contain a nucleotide-binding site (NB) and leucine-rich repeat (LRR) domain, and are members of the STAND (Signal Transduction ATPase with Numerous Domains) protein family of NTPases, known as NB-LRRs [3,4]. The nucleotide binding site forms part of a larger complex known as NB-ARC, which reflects its presence in the human apoptotic protease-activating factor-1 (APAF-1), plant R proteins and *Caenorhabditis elegans *death-4 protein (CED-4) [5]. Further subdomains and multiple conserved motifs have been identified within the NB-ARC domain [3].

Based on the presence or absence of N-terminal domains, members of the NB-LRR family can be divided into two major groups. The first group contains an N-terminal domain with homology to the *Drosophila *toll and human interleukin-1 receptor (TIR) and is referred to as TIR-NB-LRRs or TNLs. The second, non-TIR-NB-LRR, group is collectively known as CNLs as some, but not all, members of this group contain a predicted coiled-coil (CC) structure in the N-terminus. This division of NB-LRR proteins is also reflected in phylogenetic analyses of the NB-ARC domains in which TNL and CNL proteins form distinct clades [6-8].

NB-LRR genes comprise one of the largest gene families in plants. Approximately 150 NB-LRR encoding genes have been identified in the genome of *Arabidopsis thaliana *Col-0 [9], 185 within *Arabidopsis lyrata *[10], 92 within *Brassica rapa *[11], 416 and 535 in the genomes of the woody species poplar and grapevine respectively [12], and 464 and 483 in two genomes of *Oryza sativa *[13]. In addition, partial NB-LRRs that lack some NB-LRR specific domains and contain, for example, only TIR, TIR-NB, CC, and CC-NB domains, have been described in plant genomes [8,10]. NB-LRR genes are ancient in their origin and have been identified in ancestors of early land plants. NB genes with sequence homology to TNLs have been described in bryophytes [14] and TNLs and CNLs have been found in gymnosperms and eudicots [15]. However, the composition of NB-LRR genes varies significantly between species [16]. The unequal representation of NB-LRR lineages within plant taxa has been typified by the low frequency of TNLs within the monocotyledonous species despite the manifestation of TNLs prior to the angiosperm-gymnosperm split [15,17].

Within genomes, NB-LRR genes are organized either as isolated genes, or as linked clusters of varying size that are thought to facilitate rapid *R *gene evolution [18]. NB-LRR gene clusters are termed homogeneous when they contain only sequences that share a recent common ancestor. In contrast, clusters that contain more distantly-related NB-LRRs are referred to as heterogeneous [19].

Potato is the most important non-cereal food crop, with worldwide production yielding approximately 330 million tonnes in 2009 (http://faostat.fao.org/site/339/default.aspx). Like all plants, potato faces a constant barrage of pest and microbial threats. More than 50 functional NB-LRR genes have been cloned from potato and related members of the Solanaceae [20] and 738 NB-LRR-like sequences have previously been identified in a BAC library prepared from a heterozygous diploid potato clone, RH [21]. The genome sequence of a doubled monoploid *Solanum tuberosum *group Phureja clone, DM1-3 516 R44 (hereafter referred to as DM), has recently been described [22]. Among the 39,031 annotated protein coding genes, 408 NB-LRR coding genes were predicted. In this study we used a process of iterated computational and manual annotation to further identify potential NB-LRR coding sequences, determine their locations on the 12 potato chromosomes and study the phylogenetic and positional relationships between the individual genes. Our results provide significant insight into the evolution of NB-LRRs and, importantly, a blueprint for future efforts to identify and more rapidly clone functional NB-LRR genes from *Solanum *species.

## Results

### Identification of NB-LRR genes within the DM genome protein models

MEME [23] was used in conjunction with a positive sequence set of 53 characterised NB-LRR protein sequences from diverse plant species and a negative sequence set containing diverse nucleotide binding protein and PRR sequences (see additional file 1, Table S1) to identify 20 sequence motifs putatively characteristic of NB-LRR proteins. Some of the disclosed motifs (Table 1) are associated with known domains from the TNL and CNL superfamilies, and 13 encompass previously described features of the NB-LRR family, such as the p-loop, RNBS-A non-TIR, RNBS-B, RNBS-C, RNBS-D, GLPL, LRR-motif 1 (LDL), MHDV, TIR-1, TIR-2, TIR-3 [6], EDVID [24], and Kin-2 [15] domains.

**Table 1:** NB-LRR-specific amino acid motifs identified with psp-gen MEME [56].

Motif^a^	Sequence^b^	Domain	Group	similar to	Reference
motif 1	PIWGMGGVGKTTLARAVYNDP	NB-ARC	CNL/TNL	P-loop	[6]
motif 2	LKPCFLYCAIFPEDYMIDKNKLIWLWMAE	NB-ARC	CNL	RNBS-D	[6]
motif 3	CGGLPLAIKVWGGMLAGKQKT	NB-ARC	CNL/TNL	GLPL	[6]
motif 4	YLVVLDDVWDTDQWD	NB-ARC	CNL/TNL	Kin-2	[6,15,16]
motif 5	NGSRIIITTRNKHVANYMCT	NB-ARC	CNL/TNL	RNBS-B	[6]
motif 6	HFDCRAWVCVSQQYDMKKVLRDIIQQVGG	NB-ARC	CNL	RNBS-A	[6]
motif 7	CRMHDMMHDMCWYKAREQNFV	linker	CNL/TNL	MHDV	[6]
motif 8	MEDVGEYYFNELINRSMFQPI	linker	CNL/TNL	-	
motif 9	LIHLRYLNLSGTNIKQLPASI	LRR1	CNL/TNL	Motif1 LDL	[6]
motif 10	LSHEESWQLFHQHAF	NB-ARC	CNL/TNL	RNBS-C	[6]
motif 11	MPNLETLDIHNCPNLEEIP	LRR	CNL/TNL	-	
motif 12	IMPVLRLSYHHLPYH	NB-ARC	CNL/TNL	-	
motif 13	QIVIPIFYDVDPSDVRHQTGSFGEAFWKHCSR	TIR	TNL	TIR-3	[6]
motif 14	AIKDIQEQLQKVADRRDRNKVFVPHPTRPIAIDPCLRALYAEATELVGIY	monocot	-	-	
motif 15	KNYATSRWCLNELVKIMECKE	TIR	TNL	TIR-2	[6]
motif 16	DAAYDAEDVIDSFKYHA	pre-NB	CNL	EDVID	[24]
motif 17	FAIPKLGDFLTQEYYLHKGIKKEIEWLKRELEFMQA	pre-NB	CNL	-	
motif 18	KYDVFLSFRGADTRRTFTSHLYEALKNRGINTF	TIR	TNL	TIR-1	[6]
motif 19	IKMVEITGYRGTRFPNWMGHPVYCNMVSISIRNCKNCSCLP	LRR	CNL/TNL	-	
motif 20	ETSSFELMDLLGERWVPPVHLREFKSFMPSQLSALRGWIQRDPSHLSNLS	monocot	-	-	

^a^Motifs are listed according to their ranking derived from the psp-gen MEME analysis. ^b^Consensus amino acid sequence derived from psp-gen MEME analysis. References for known motifs encompassed in the MEME motifs are shown.

The 20 potentially characteristic motifs were used as queries in a MAST [25] search against a combination of the annotated potato genome v3.4 DM protein models (DMP) and the training set sequences used to derive the motifs. In total, 765 DMPs were identified as possessing the motifs identified by MEME, with an E-value of less than 2 (see additional file 2, Figure S1). The positive and negative training set sequences could be distinguished with 100% specificity on the basis of reported E-values. In total 343 DMP sequences had reported E-values less than the highest seen for a member of the positive training set (E < 2.7e-45). A further 134 DMP sequences had E-values less than the smallest E-value observed for a member of the negative training set (E < 8.5e-24). Thus, a total of 477 candidate NB-LRR DMP sequences were identified on the basis of motif composition.

### Manual re-annotation of DM gene models containing NB-LRR-like sequences

Manual inspection of the remaining 288 DMPs whose E-values lay above the 8.5e-24 cut-off indicated that several sequences contained motif patterns potentially characteristic of NB-LRR proteins, but that were truncated or otherwise distorted. Of these, 87 sequences that contained at least two TIR/CC-specific motifs, or three NB-ARC specific motifs, were noted as potential errors in automated gene calling or annotation and carried forward into the candidate set pending a manual check, to give a total of 564 putative NB-LRR DMP sequences.

Several of the candidate DMP sequences derived from the same DM gene model (DMG) sequence as alternative transcripts. We found that 469 distinct DMG sequences coded for the 564 candidate NB-LRR sequences. The MAST search was repeated against conceptual translations of these 469 DMGs, and indicated that 277 DMG translations apparently lacked domains characteristically associated with TNL or CNL genes. To investigate if misannotation might be responsible for these absences, these DMG sequences were extended by 3 kb at both the 5' and 3' ends to generate a counterpart DMG+ sequence set. The MAST search was repeated against the conceptual translations of the DMG+ sequences. We found that all 277 DMG sequences that initially lacked typical NB-LRR domains contained additional MEME motifs in an order characteristic of the other candidate NB-LRR sequences.

Gene models corresponding to the DMG+ sequences were modified to incorporate the additional characteristic motifs identified above. Conceptual translations of these genes (referred to as DMP+ sequences), were compared to NB-LRR proteins in the nr database at NCBI using BLASTP [26] to identify potential introns and start and stop codons. In addition, six DMG+ models appeared to encode two complete NB-LRR-like sequences, so were split into a total of twelve distinct gene models. A further 15 NB-LRR-like sequences appeared to have been split across two adjacent DMGs in the initial annotation. Thus, the number of identified NB-LRR-like sequences after manual correction was 454. A further MAST search was carried out on these sequences, from which 438 DMG sequences were found to have an E-value less than that for any member of the negative sequence set (see additional file 3, Table S2). Re-annotated coding sequences and the conceptual translations are supplied in additional file 4.

In total, 154 of the predicted NB-LRR sequences are encoded by a single reading frame without introns. A further 110 predicted NB-LRRs contain a single intron and/or a frameshift, and 100 genes contain two introns and/or frameshifts. The remaining 74 genes have between three and eight introns and/or frameshifts. Without further detailed analysis (e.g. RNA sequencing), it is difficult to determine if the predicted introns and/or frameshifts are genuine or a result of sequencing/assembly errors. However, of the 154 candidate NB-LRR genes without an intron, 116 contain all domains associated with TNLs or CNLs and are thus referred to as 'full length'. A further 97 genes that contain one or two potential introns but no frameshift are also classified as 'full length' on the same grounds. Among the other DMG+ sequences, 155 contain all domains associated with TNLs or CNLs, and are labelled as 'potentially full length'. The remaining 70 genes are classified as 'partial', as they show truncations within the N-terminal domains and/or absence of LRR domains. The average length of the coding sequence for partial genes is 1 kb, for full length and potentially full length genes 3 kb, and for all identified NB-LRR genes combined 2.7 kb.

Based on the presence of the TIR domain derived motifs (13, 15 and/or 18), 77 genes were identified as TNLs. This data was verified using a Pfam [27] search over all sequences. All 55 full length and potentially full length TNLs share the TNL discriminating aspartic acid (D) in the final position of the Kin-2 domain [6,15,16]. The 316 (potentially) full length non-TIR sequences encode for a tryptophan (W) in this position, and contain the CNL specific motifs 16 and/or 17. This analysis was further corroborated by the presence of the CNL-type NB-ARC motifs 2 and 6, that encapsulate RNBS-D and RNBS-A, described by Meyers et al. (1999) [6]. A Paircoil2 analysis [28] was carried out on the positive training set (see additional file 1, Table S1) to establish the conditions for coiled-coil domain predictions in well annotated genes. The highest minimum p-score for a functional CC-NB-LRR gene was found for *Rpi-vnt1 *[29] with 0.047 starting at amino acid position 73. The latest start position of a CC domain was determined for *R2 *and *Rpi-blb3 *at amino acid position 98 (data not shown). To determine the presence of CC motifs within the 438 predicted NB-LRRs, a p-score cut-off of 0.047 was used for domains starting within the first 98 amino acids. Under these conditions, 107 NB-LRR genes were identified that contain a predicted CC domain. A total of 254 CNL genes do not contain a predicted CC domain. The TNL and CNL prediction counts are summarised in Table 2 and compared to the initial analysis from the PGSC [22]. Amongst the predicted TNLs and CNLs, homologues of the functionally characterised Solanaceae *R *genes *Gpa2, NRC1, R1, R2, Rpi-bt1, Rpi-blb2, Rpi-blb3, Rpi-vnt1*, and *Rx *were identified with more than 80% sequence identity using BLASTP. Further homologues of other functionally described Solanaceae *R *genes were identified, albeit with lower percentage sequence identity (see additional file 5, Table S3).

**Table 2:** Comparison between DM NB-LRR genes identified and re-annotated in this study with the data published by the Potato Genome Sequencing Consortium [22].

	NB-LRRs		PGSC	
	#	%	#	%
**TNL**	**77**	**17.6**	**49**	**12.0**
TIR-NB	22	5.0	14	3.4
TIR-NB-LRR	55	12.6	35	8.6

**CNL**	**361**	**82.4**	**359**	**88.0**
CC-NB	4	0.9	22	5.4
CC-NB-LRR	103	23.5	60	14.7
NB-LRR	213	48.6	172	42.2
NB-ARC	41	9.4	105	25.7

**total**	**438**		**408**	

Partial genes (TIR-NB, CC-NB, NB-ARC) and (potential) full length genes (TIR-NB-LRR, CC-NB-LRR, NB-LRR) are shown.

### Phylogenetic analysis

**Figure 1:**
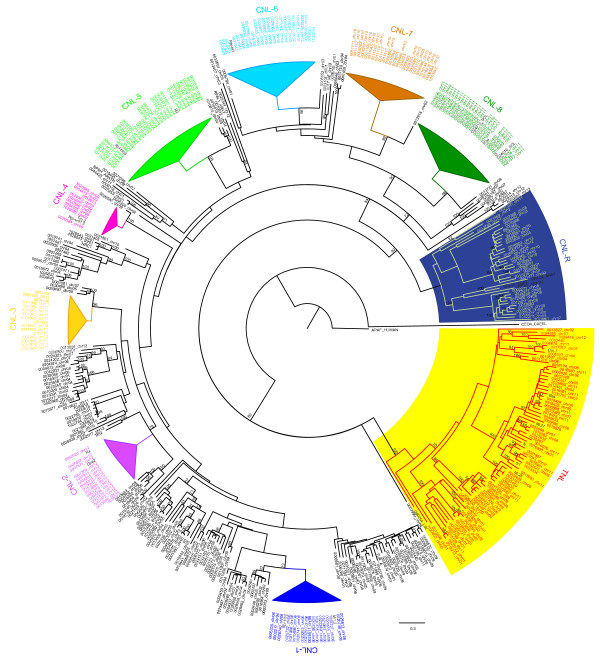
**Maximum Likelihood Phylogenetic analysis of the predicted DM NB-LRR genes**. The NB-ARC domains of TNL and CNL type genes were used, alongside selected NB-ARC domains from functional resistance genes, to study the phylogenetic relationships between them. Subgroups with highly similar gene products are marked: TNL genes have a yellow background, CNL-R type NB-LRR genes a blue background and CNL-1 to CNL-8 are shown in various colours. The gene product labels contain the 7 last informative digits from the DMG identifier, followed by their chromosomal position if known. Bootstraps over 70 (out of 100) are shown.

**Figure 2:**
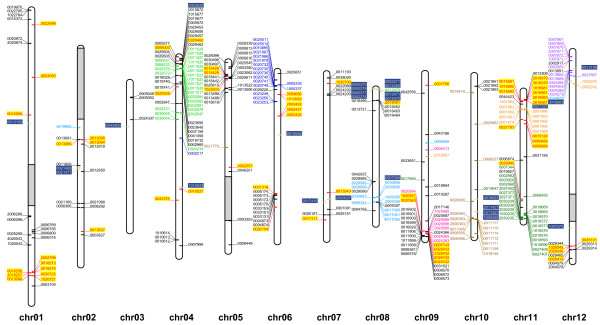
**Physical map of the 12 potato chromosomes with individual CNLs and TNLs**. The relative map position of 366 unique DMGs encoding for NB-LRR type genes is shown on the individual pseudomolecules depicting the chromosomes 1-12. Each gene has a unique label representing the 7 last informative digits from the DMG identifier. Genes encoded by the positive DNA strand are depicted on the left hand side of the chromosomes, whereas those encoded by the negative strand are shown on the right. Colours and background of the genes are identical to the phylogenetic subgroups (TNL, CNL-R, CNL-1 to CNL-8) shown in Figure 1. Grey bars on chromosomes 1, 2, 5 and 12 represent known gaps in the assembly.

**Figure 3:**
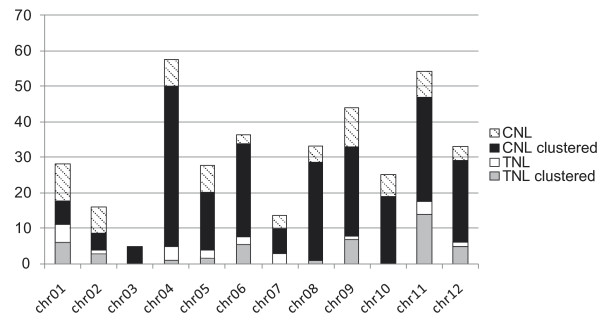
**CNL and TNL organisation within the potato genome**. The distribution of NB-LRR genes is shown for each chromosome. Bars are divided into CNL genes (white-textured for non-clustered genes and black for those found in clusters) and TNL genes (white for non-clustered genes and grey for clustered TNLs).

To study the evolutionary relationships among the predicted NB-LRR genes, a phylogenetic tree was estimated from the protein alignment of the conserved NB-ARC domains. Predicted NB-LRR genes containing ambiguous nucleotides in the NB-ARC domain were removed prior to the alignment. In addition to 413 predicted TNLs and CNLs, 33 functional NB-LRR genes from the positive training set were also included in the analysis. As expected (e.g. [6]), the phylogenetic analysis separates the TNL and CNL gene products into two distinct clades and confirms thus our TIR motif prediction above (see Figure 1 and more detailed additional file 6, Figure S2). The TNL clade contains 68 NB-LRR sequences of which 6 are partial, missing motifs 2 and 6, and can be divided into six small subgroups. Physical mapping of these (Figure 2, and more detailed additional files 7, 8, 9, 10, 11, 12, 13, 14, 15, 16, 17 and 18) indicates that members of five subgroups are distributed over several chromosomes (Figures 2 and 3). Only members of one subgroup reside predominantly (8 out of 9) in a NB-LRR gene cluster on chromosome 9 (Figure 2, and more detailed additional file 15).

Only a single DMG product, PGSC0003DMG400007999 (DMG identifiers hereafter are shortened to the last seven informative digits; DMG 0007999), could not reliably be placed in either of the CNL or TNL clades. The encoded gene product shows high sequence similarity (including the conserved TVS and PKAE amino acid motifs) to the atypical Arabidopsis/potato ADR1 CC-NB-LRR protein [30]. Bootstrap support is given that further divides the CNL clade into CC_RPW8_-type sequences (referred to as CNL-R) [31], and the canonical CNL proteins, that, with the exception of DMGs 0029313, 0029314 and 0029405, contain the EDVID motif (CC_EDVID_-type) which is typically associated with CNLs [24]. The CNL branch contains eight highly conserved subgroups (CNL-1 to CNL-8) amongst more diverse sequences and subgroups. CNL-1 contains 18 genes that map, with one exception, to chromosome 6. Members of this subgroup are homologous to the functional resistance to *Phytophthora infestans (Rpi) *genes *Rpi-blb2 *[32] and *Mi-1 *[33]. CNL-2 members show sequence similarity to the functionally validated genes *Gpa2 *and *Rx *[34]. Apart from one gene for which the physical position could not be retrieved from the assembly, the remaining 14 members reside on chromosome 12. The subgroup CNL-3 contains 16 members, of which four remain unmapped. There is a single gene from this subgroup located on each of chromosomes 9 and 12, and ten genes on chromosome 11. Members of the smallest subgroup CNL-4 are homologous to *Rpi-vnt1 *[35] and *Tm-2 *[36]. The eight mapped members reside on chromosome 9 and one gene remains unmapped. The largest subgroup, CNL-5, contains 30 genes of which six remain unmapped and 24 reside on chromosome 4. Functionally validated *R *genes with sequence similarity to this subgroup include *R2 *and *Rpi-blb3 *[37,38]. Half of the 24 members of CNL-6 map to chromosome 8, one each to chromosome 2, 9 and 12 respectively, and the remaining nine are unmapped. The *Rpi-blb1/RB *[39,40] and *Rpi-bt1 *[41] genes share sequence similarity with this group. Of the 24 sequences in CNL-7, 17 are localised on chromosome 10, one on chromosome 4 and six did not map to any of the chromosomes in this assembly. The CNL-8 subgroup contains 26 sequences. The physical mapping of these genes has placed 24 on chromosome 11 and the remaining two on chromosomes 9 and 10. The functionally validated potato and tomato *R *genes *R3a *[42], *R3b *[43] and *I2 *[44] share sequence similarity with members of this group.

### NB-LRR gene mapping and physical clustering

Physical map positions for predicted NB-LRR genes were established for 370 (84%) of the annotated NB-LRR genes, using anchored superscaffold positions in the pseudomolecules described in the publicly available potato genome annotation v3_2.1.10 (PGSC_DM_v3_2.1.10_pseudomolecule_annotation.gff.zip) and visualised using Biopython [45] (Figure 2, and more detailed additional files 7, 8, 9, 10, 11, 12, 13, 14, 15, 16, 17 and 18). CNLs are present on all 12 chromosomes whilst TNLs are absent from chromosomes 3 and 10 (Figures 2 and 3). The greatest number of NB-LRRs is found on chromosomes 4 and 11, harbouring 57 and 54 genes, respectively. Chromosome 3 contains the smallest number of NB-LRR genes (four) (Figure 3). From the map positions, NB-LRR gene clusters were determined by a combination of two previously described approaches [9,12]. To form a cluster, the distance between neighbouring NB-LRRs was required to be less than 200 kb, and for there to be fewer than eight non-NB-LRR genes between TNLs or CNLs. This approach identifies 63 clusters containing a total of 271 NB-LRRs (Figure 3). Thus 27% of the mapped NB-LRR genes appear not to be organised in physical clusters. Of the 63 clusters, 50 (79%) are homogeneous in that they contain only predicted NB-LRRs with a recent common ancestor, whereas the remaining clusters are heterogeneous, as they contain more distantly-related NB-LRRs.

**Figure 4:**
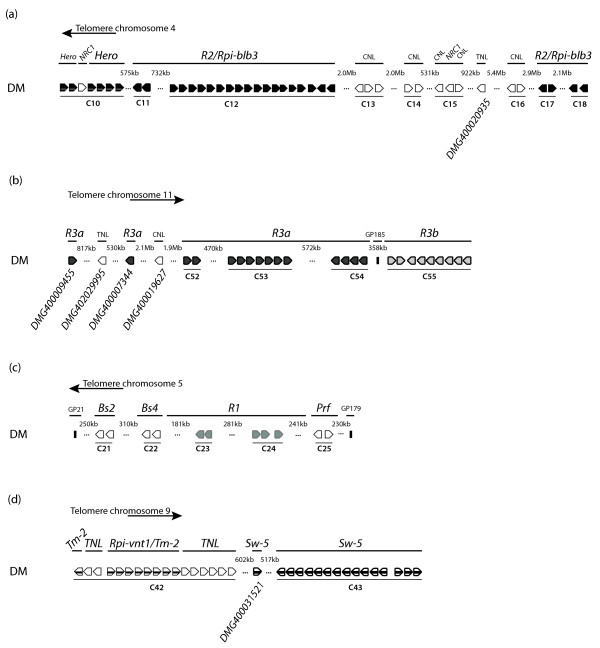
**Physical overview of selected resistance loci**. *R2 *(a), *R3 *(b), *R1 *(c) and *Rpi-vnt1*/*Tm-2*/*Sw-5 *(d). The directions towards the respective telomeres are shown. Boxed arrows symbolise NB-LRR genes and clusters are indicated by horizontal lines. Known genetic markers are shown. The distances between NB-LRR clusters are indicated above the gaps. Identifiers for single NB-LRRs are shown.

Chromosome 4 contains the greatest number of NB-LRR genes (57) and also the largest number of clusters (11). With the exception of cluster C10, which contains five homologues of the *R *gene *Hero *and one TNL, all remaining clusters on this chromosome are homogeneous clusters. The sizes of the clusters vary between two and 18 NB-LRR genes (see additional file 10). Eleven genes on chromosome 4 are not organised in clusters. The physically expanded and well described *R2 *and *Rpi-blb3 *locus [38] is located on this chromosome and its DM homologues are organised in the phylogenetic subgroup CNL-5 which spans four physical clusters (Figure 4a). Eighteen members form the homogeneous cluster C12, which is also the largest of all. The remaining members of CNL-5 are found in cluster C11, and two more are grouped (in C17 and C18) downstream of the bulk of the clusters.

The heterogeneous *R3 *locus that contains the *Rpi *genes *R3a *[42] and *R3b *[43] resides on the distal end of the long arm of chromosome 11. As mentioned, DM homologues of *R3a *and *R3b *form the phylogenetic subgroup CNL-8. Of the 26 members in this subgroup, 24 map to chromosome 11. *R3a *homologues are organised in three neighbouring homogeneous clusters: C52, C53 and C54 that contain two, seven and four members respectively. Two additional single *R3a *homologues are located upstream of C52. *R3b *homologues are organised in cluster C55 which harbours nine members (Figure 4b).

Previous studies have shown that the *R1 *resistance gene locus resides on chromosome 5 and is flanked by *Bs4*- and *Prf*-like *R *genes [46,47]. This structure has been maintained in DM. Four adjacent clusters (C22 - C25) contain two TNLs with homology to *BS4 *(C22), five *R1 *homologues in clusters 23 and 24, and two *Prf *homologues in cluster 25. Two *BS2 *homologues in cluster 21 (Figure 4c), lie approximately 310 kb upstream of C22.

The long arm of chromosome 9 features two large heterogeneous clusters. Cluster 42 harbours eight TNLs that are separated by eight homologues of *Rpi-vnt1 *[29] and *Tm-2 *[36]. The more distal cluster C43 contains 15 homologues of the Tospovirus resistance gene *Sw-5 *[48] (Figure 4d).

### Genomic organisation of NB-LRR genes

**Figure 5:**
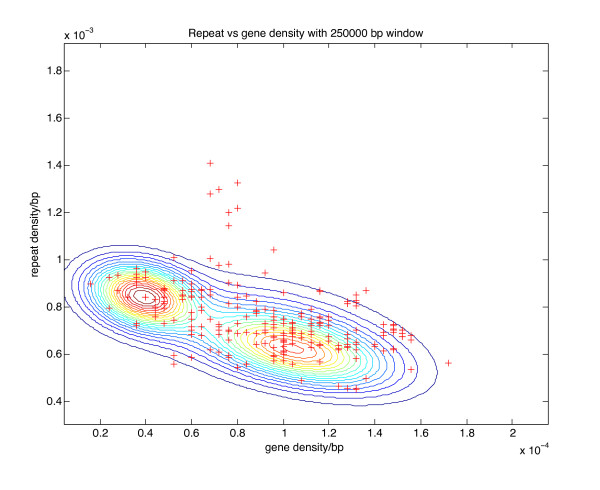
**Global gene density versus repeat density analysis**. The contours represent a genome-wide Gaussian mixture model (GMM) with two components fitted to the gene/repeat density data in a 250 kb analysis window. Overlaid on the calculations are the CNLs and TNL type genes (shown as red crosses).

Gene and repeat densities were calculated and visualised for mapped gene features of the DM genome using a window size of 250 kb centred on each gene in the corresponding superscaffolds. DMGs for which the 250 kb window would extend beyond a superscaffold were omitted from the analysis. Figure 5 indicates contours for a Gaussian mixture model (GMM) with two components that was fitted to the gene/repeat density data. The bulk gene/repeat density is modelled as two overlapping populations that are better distinguished in terms of gene density than repeat density. This is consistent with the potato genome analysis described by Xu et al. (2011) [22], indicating that there are relatively 'gene-rich' and 'gene-poor' regions within the DM genome. The GMM is overlaid in each case with a scatterplot showing data for predicted NB-LRR genes that were suitably placed for analysis within the superscaffolds. The majority of NB-LRRs lie within the contours of the GMM, consistent with the distribution of NB-LRRs being similar to that of all other genes in the potato genome. Only sixteen genes are visually distinguished as lying outside the contours of the GMM and mainly located in relatively repeat-rich regions. This number is within the statistical expectancy of sampling error. It is however interesting to note, that eight of these genes are members of phylogenetic subgroup CNL-1: DMG 0025512 from cluster 27 and DMGs 0031878, 0020732, 0020735, 0020736, 0020740, 0020741, and 0020749, which are adjacent to one another in cluster 28. Phylogenetically, members of the subgroup CNL-1 are most similar to the *P. infestans *resistance gene *Rpi-blb2 *and the nematode and aphid resistance gene *Mi-1 *(Figure 1, and additional file 5, Table S3). Four further CNLs that are located in more repeat-rich regions are DMGs 0029453, 0029505 and 0029506, and all of them grouped together in the heterogeneous cluster C10 on chromosome 4 whereas DMG 0016372 is a single NB-LRR gene on chromosome 1.

## Discussion

We used an iterative process of manual and computational analysis to identify 438 NB-LRR-encoding sequences within the recently published doubled monoploid potato genome [22]. This study has revealed a slightly higher number of CNLs and TNLs compared to the 408 NB-LRRs described by Xu et al. (2011) [22]. The difference, which is within the expected sampling error, includes 28 additional TNL genes and 2 additional CNLs. By extending the DM gene models by 3 kb at the 3' and 5' end respectively to produce the DMG+ sequences, more domains associated with NB-LRR type genes were identified and the gene annotations correspondingly extended. The number of annotated partial NB-ARC only genes fell in our predictions from 105 to 41 (Table 2). Whilst our analysis used NB-LRR discriminative MEME motifs derived from a training set harbouring functionally characterised NB-LRRs from the wider plant kingdom, the analysis described by Xu et al. (2011) [22] is based on NB-derived Pfam domain searches, followed by the construction of a potato-specific NB hidden Markov model. Both approaches yielded very similar numbers of NB-LRRs. Unfortunately, a direct comparison between the different resistance gene homologues (RGHs) was not possible as the identities of the CNL and TNL genes predicted by Xu et al. (2011) [22] were not made publicly available.

The MEME motif and phylogenetic analysis revealed a distinction between CNLs and TNLs in the N-terminal region, and in the NB-ARC domain of these sequences. Seven of the 20 identified MEME motifs (Table 1) distinguished between these NB-LRR subclasses, or between the canonical and RPW8-type CNLs [24]. Phylogenetic analysis, which was performed on the conserved NB-ARC domain, supported this distinction and was consistent with previous observations for other plant species [6,7,9-12,24,49].

The DM potato genome harbours 4.7 times more CNL than TNL genes. A similar distribution was found for the NB-LRR genes of grapevine (3.8×), but the ratio is smaller in poplar (1.7×) [12]. In comparison, the NB-LRRs of the Brassicaceae *A. thaliana, A. lyrata *and *B. rapa *contain CNLs and TNLs in a 1:2 ratio [9-11]. The genome of the monocot rice contains only CNLs; all other grasses analysed so far contain no or only very few TNLs [13,15]. Leister (2006) suggested that overrepresentation of TNL over CNL genes in the Brassicaceae Arabidopsis and rape seed could reflect the adaptation of the *R *gene set to the predominant pathogens. It can be speculated that the over-representation of CNLs in potato is a response to some of the most damaging pathogens such as *P. infestans*, which is typically controlled by CNLs. In line with this, it is interesting to note that 27% of the identified NB-LRR genes share high sequence similarity to functionally characterised *Rpi *genes (data not shown).

The proportion of all genes that are predicted to encode NB-LRRs is 1.16%, which is in line with estimates for other plant species that range between 0.6-1.8% [11]. The gene density around potato NB-LRR loci is approximately 100 genes per megabase. However, unlike RxLR effectors from *P. infestans *which often reside in gene sparse regions [50], a global analysis of the DM NB-LRR genes (Figure 5) shows that CNLs and TNLs reside in genomic regions that are not significantly different to the potato genome in general in terms of gene or repeat density.

The CNL branch forms two phylogenetic clades, containing the canonical CNLs and the CNL-R (CC_RPW8_-type), as previously described [24,49]. Within the canonical CNLs, eight major subgroups with high support and short branch length were identified, suggesting a recent common ancestor. Two-thirds (13 of the 21) of the functional CNL genes included in the tree are found in these subgroups. Only members of CNL-3 and CNL-7 (and some of the smaller subgroups) show no significant sequence similarity to a functionally characterised *R *gene thus far. Their role, which is hitherto unknown, could for example be to provide resistance to yet unknown pathogens and/or to mediate non-host resistance responses [2].

Several approaches for the identification of NB-LRR clusters have been described elsewhere, and we have utilised a combination of the analyses described by [12] and [9]. The identified members and the overall number of predicted clusters were very similar for both types of analyses, suggesting that the identification of clusters by these methods is relatively robust. However, cluster prediction based on the distances between NB-LRRs does not take into account the variability of gene density in the potato genome [22]. Similarly, the definition of a gene cluster solely based on the number of non-NB-LRR genes between CNLs and TNLs fails to take any physical distance into account. Predicted potato NB-LRR genes are unevenly distributed over the 12 chromosomes and cluster into groups of different sizes. This is in line with data for other plant species [9,11,12]. Various mechanisms including recombination, gene conversion, duplication and selection are thought to contribute to the genome-wide diversity and distribution of NB-LRR gene loci [19,51-53]. Equal intragenic crossing-over results in domain swaps between genes whereas unequal crossing-over influences the number of genes within a locus and potentially places genes into a new structural context. Tandem duplications, in which the copy is contiguous to the original gene, are typically associated with homogeneous clusters. Of the 63 clusters, 50 are homogeneous and thus likely a result of tandem duplications. Members of the subgroups CNL-1 to CNL-8 are often found on the same chromosome and, in some cases, within the same clusters, which is consistent with tandem duplication. In contrast, segmental and ectopic duplications, which involve the duplication of entire gene blocks or single/small groups of genes respectively, can position copies to unlinked sites including different chromosomes [51]. Both CNL and TNL distributions display evidence for events that placed homologous genes onto different chromosomes that could be a result of either segmental or ectopic duplication. These events appear to be more common for TNLs that are more widely dispersed throughout the genome and not found in clusters as frequently as CNLs.

The sequencing of DM provides a snapshot of the potato genome organisation, and specifically the distribution of and relationships among NB-LRR genes on individual chromosomes. Although specific to DM, this analysis provides important insight into the NB-LRR gene compositions of other members of the Solanaceae. Studies in Arabidopsis have shown, for example, that some *R *genes display high levels of polymorphism within and between populations [10]. A more detailed analysis of the potato *R1 *locus [46], for which three haplotypes from *S. demissum *have been described [47], confirmed evidence of copy number variations and is consistent with tandem duplications. As previously described, the *R1 *locus is flanked by *Bs4*-like and *Prf*-like genes but the number of *R1*-homologues varies between one and 17 in *S. demissum *and five in DM (Figure 4c; [47]). Another example is the *R3 *locus on chromosome 11 which was originally described in a diploid potato population, SHxRH [54]. Overall, *R3 *cluster organisation is syntenic between SH-haplotypes and the sequenced DM, in that the *R3a*-clusters (C52, C53, and C54 proximal) and the *R3b *cluster (C55, distal) flank the marker GP185 (Figure 4b). However, in DM, the physical distance between the clusters C54 and C55 amounts to more than 350 kb and is thus approximately 200 kb shorter than the same region in SH [43]. In DM, nine *R3b *homologues reside in cluster C55, and Li et al. (2011) [43] describe six and ten homologues for the two SH haplotypes. Unequal representation of lineages within the NB-LRR superfamily and copy number variation between haplotypes is consistent with a 'birth and death' model in which some NB-LRRs are lost and new lineages evolve whilst others are retained [55].

We have observed 438 NB-LRR genes in a doubled monoploid potato, which represents a single haplotype. Potato cultivars and breeding lines are generally heterozygous tetraploids, which exhibit tetrasomic inheritance during crossing. The high levels of structural diversity observed in homologous *R *gene clusters from different potato haplotypes (e.g. [21,46,47]), and the extremely high levels of sequence polymorphism observed in potato, imply that it is highly likely that any given tetraploid potato clone may contain as many as 1,600 distinct NB-LRRs in its genome. A key objective for future resistance breeding is to understand the allelic diversity of NB-LRR genes in potato. Such an objective will require application of high throughput sequencing technologies allied to advanced bioinformatic tools for assembling sequence data from very closely related genes.

## Conclusions

We have identified 438 NB-LRR type genes within the sequenced potato *S. tuberosum *Group Phureja (DM), of which several are homologous to functionally characterised *R *genes. Comprehensive analysis of the NB-LRRs, both in terms of the phylogenetic relationships of CNLs and TNLs and their positions on the respective chromosomes, provides an invaluable tool for the identification of novel and functional *R *genes from wild *Solanum *species in the future. New technologies, including exon capture followed by high throughput sequencing and allele mining rely on detailed information concerning *R *gene organisation and distribution. Furthermore, knowledge about the genomic organisation of these genes will facilitate comparative and evolutionary studies on a whole genome level or, alternatively, for selected clusters.

## Methods

### Identification of NB-LRR genes

'Positive' NB-LRR and 'negative' non-NB-LRR sequence training sets were used with the MEME Suite psp-gen script (version 4.4.0) [56] to encapsulate information about probable discriminative motifs in the positive set. Then, using the psp file as additional input, MEME was run on the positive training set to identify the 20 most significant motifs in the sequences (Table 1). A MAST search was then conducted on a combined dataset of all (~56 k) predicted protein models (PGSC0003DMP.pep.v3.4) and the training sets (see additional file 2, Figure S1). DMP sequences were considered to be candidate NB-LRRs if their reported MAST E-values were lower than the least E-value for any member of the negative training set. A manual inspection of DMPs with E-values above this threshold was conducted to identify potential false negative results. Sequences that contained at least two TIR/CC-derived motifs or three NB-ARC-specific motifs were selected for further analysis as described below.

DM gene models (DMG) corresponding to the identified NB-LRR like DMPs, were extracted from 'PGSC_DM_v3.4_gene.fasta'. DMG sequences were extended by 3 kb at the 5' and 3' ends using the DM superscaffold sequences in 'PGSC0003DM.superscaffold.fa' to generate the DMG+ set of potato genes, which were translated in all six reading frames. The MAST search with the potentially discriminatory MEME models was repeated to identify potentially missing domains, and the DMG+ sequences manually curated to produce the DMP+ set of protein sequences. DM homologues to members of the positive Solanaceous training set were identified by BLASTP [26] search.

### Mapping annotated DMGs and repeat densities to the pseudomolecules

All DM superscaffold locations were extracted from the spreadsheet PGSC_DM_v3_2.1.9_pseudomolecule_AGP.xlsx, downloaded from the PGSC data sharing site at http://potatogenomics.plantbiology.msu.edu/index.html (accessed on 25-09-2011). All DMGs were mapped from the input file PGSC_DM_v3.4_gene.gff, and all repeat positions were mapped from the file PGSC0003DMB.repeatmasker.gff (both provided by the PGSC), to the pseudomolecules.

Gene and repeat densities were calculated for each annotated gene, using a range of window sizes (50 kb, 100 kb, 175 kb, 250 kb, 350 kb, 500 kb) centred on that gene, and relative only to the superscaffold on which the gene were located. Only the parent superscaffold was used because the 50 kb spacer regions introduced into the pseudomolecules may not accurately represent the expected separation between superscaffolds. Gaussian mixture models were fitted to the observed frequencies of gene vs repeat density for all annotated genes, using 200 bins for each measure.

Genes are considered to form clusters on a pseudomolecule when the distance between two neighbouring NB-LRR is less than 200 kb [12], and no more than eight annotated non-NB-LRR sequences are present between two consecutive NB-LRR sequences [9].

### Multiple alignment and phylogenetic tree estimation

The NB-ARC protein domain region was chosen for phylogenetic analysis as the multiple alignment was tractable. NB-ARC sequences that were not full length were manually checked for sequencing and assembly errors. After this screening step, sequences of less than 50% of the full-length NB-ARC domain were excluded. The multiple alignment was built from 466 re-annotated DMG's, including 33 annotated *R *gene sequences (see additional file 1, Table S1) using the Pfam [57] NB-ARC domain (Pfam entry PF00931) seed alignment (12 sequences) and associated hidden Markov model using the hmmalign program from the HMMER 3.0 package [58]. Model selection, using the joint estimation of amino acid substitution model and phylogenetic tree topology, was carried out using the TOPALi package [59], resulting in the selection of a WAG+I+G model. This model was used to estimate a Maximum Likelihood phylogenetic tree using the PhyML package [60]. Bootstrap support was based on 100 bootstrap replicates.

## List of Abbreviations

TIR: toll/interleukin 1 receptor; CC: coiled-coil; NB-LRR: Nucleotide binding-site and leucine-rich repeat; DM: *Solanum tuberosum *Group Phureja clone DM1-3 516 R44; DMG: DM gene model; DMG+: re-annotated DM gene model; DMP: DM protein model; DMP+: re-annotated DM protein model; DMT: DM transcript model; PAMP: pathogen associated molecular pattern; PTI: PAMP triggered immunity; ETI: effector triggered immunity; STAND: Signal Transduction ATPase with Numerous Domains; NB-ARC: Nucleotide binding site and human apoptotic protease-activating factor-1 (APAF-1), plant R proteins and *Caenorhabditis elegans *death-4 protein (CED-4); TNL: TIR-NB-LRR; CNL: non-TIR NB-LRR; BAC: bacterial artificial chromosome; PGSC: Potato Genome Sequencing Consortium;

## Authors' contributions

FJ carried out the sequence analyses, designed and performed the manual re-annotation and analysed the physical and phylogenetic relationships. GE carried out the MEME and MAST analyses and sequence extractions. LP designed bioinformatic analyses, calculated repeat and gene densities, and created the gene map. KM and FW carried out the multiple alignments and phylogenetic tree estimations. PC visualised the gene map. SKS and DB aided the genome analysis. IH, JJ and GB designed the research. IH, FJ, LP and GE wrote the manuscript. All authors read and approved the final manuscript.

## Supplementary Material

Additional file 1 **Gene bank (NCBI) accession numbers for proteins used in the positive and negative training sets**. 'Positive' NB-LRR and 'negative' non-NB-LRR sequence training sets were used with the MEME Suite psp-gen script (version 4.4.0) [56] to identify discriminative motifs from the positive set. Click here for file

Additional file 2 **Graphical MAST search output**. Graphical overview of the MAST search output ranked according to the E-value scores obtained for MEME motifs. By including DMPs that yielded an E-value score of up to 2.0, 765 proteins were identified. Within the E-value range of the negative training set, 87 sequences encoded for very short DMPs and contained additional NB-LRR gene associated domains in the extended DMP+ sequence. Click here for file

Additional file 3 **List of identified DM NB-LRR genes**. Identified NB-LRR genes are listed, together with information on their PGSC identity, coding DNA strand, annotation, number of identified open reading frames (ORFs), the predicted pseudomolecule (LG), start of original DMG on LG, end of original DMG on LG, repeat density, gene density, and motif complement of the annotated sequence DMG+. Click here for file

Additional file 4 **FASTA sequences for the re-annotated DM NB-LRR coding sequences and the conceptual translations**. This file contains the re-annotated coding sequences for identified DM NB-LRR genes, as well as the derived amino acid translation. IDs correspond to the original DMG identifiers provided by the PGSC. Click here for file

Additional file 5 **Comparison of functionally characterised Solanaceae *R *genes to DM NB-LRR cds**. E-values, pairwise identity and coverage were established using BLASTP. The chromosome and cluster positions are shown alongside the phylogenetic group information. Click here for file

Additional file 6 **Detailed phylogenetic analysis of the DM NB-LRR NB-ARC domains**. The NB-ARC domains of TNL and CNL type gene products were used, alongside selected NB-ARC domains from functional resistance genes, to study the phylogenetic relationships between them. Click here for file

Additional file 7 **Detailed view of potato chromosome 1**. Genes encoded by the positive DNA strand are depicted on the left hand side of the chromosome, whereas those encoded by the negative strand are shown on the right. NB-LRR genes belonging to clusters are indicated by vertical bars. Heterogeneous clusters are indicated by an *. Click here for file

Additional file 8 **Detailed view of potato chromosome 2**. Genes encoded by the positive DNA strand are depicted on the left hand side of the chromosome, whereas those encoded by the negative strand are shown on the right. NB-LRR genes belonging to clusters are indicated by vertical bars. Heterogeneous clusters are indicated by an *. Click here for file

Additional file 9 **Detailed view of potato chromosome 3**. Genes encoded by the positive DNA strand are depicted on the left hand side of the chromosome, whereas those encoded by the negative strand are shown on the right. NB-LRR genes belonging to clusters are indicated by vertical bars. Heterogeneous clusters are indicated by an *. Click here for file

Additional file 10 **Detailed view of potato chromosome 4**. Genes encoded by the positive DNA strand are depicted on the left hand side of the chromosome, whereas those encoded by the negative strand are shown on the right. NB-LRR genes belonging to clusters are indicated by vertical bars. Heterogeneous clusters are indicated by an *. Click here for file

Additional file 11 **Detailed view of potato chromosome 5**. Genes encoded by the positive DNA strand are depicted on the left hand side of the chromosome, whereas those encoded by the negative strand are shown on the right. NB-LRR genes belonging to clusters are indicated by vertical bars. Heterogeneous clusters are indicated by an *. Click here for file

Additional file 12 **Detailed view of potato chromosome 6**. Genes encoded by the positive DNA strand are depicted on the left hand side of the chromosome, whereas those encoded by the negative strand are shown on the right. NB-LRR genes belonging to clusters are indicated by vertical bars. Heterogeneous clusters are indicated by an *. Click here for file

Additional file 13 **Detailed view of potato chromosome 7**. Genes encoded by the positive DNA strand are depicted on the left hand side of the chromosome, whereas those encoded by the negative strand are shown on the right. NB-LRR genes belonging to clusters are indicated by vertical bars. Heterogeneous clusters are indicated by an *. Click here for file

Additional file 14 **Detailed view of potato chromosome 8**. Genes encoded by the positive DNA strand are depicted on the left hand side of the chromosome, whereas those encoded by the negative strand are shown on the right. NB-LRR genes belonging to clusters are indicated by vertical bars. Heterogeneous clusters are indicated by an *. Click here for file

Additional file 15 **Detailed view of potato chromosome 9**. Genes encoded by the positive DNA strand are depicted on the left hand side of the chromosome, whereas those encoded by the negative strand are shown on the right. NB-LRR genes belonging to clusters are indicated by vertical bars. Heterogeneous clusters are indicated by an *. Click here for file

Additional file 16 **Detailed view of potato chromosome 10**. Genes encoded by the positive DNA strand are depicted on the left hand side of the chromosome, whereas those encoded by the negative strand are shown on the right. NB-LRR genes belonging to clusters are indicated by vertical bars. Heterogeneous clusters are indicated by an *. Click here for file

Additional file 17 **Detailed view of potato chromosome 11**. Genes encoded by the positive DNA strand are depicted on the left hand side of the chromosome, whereas those encoded by the negative strand are shown on the right. NB-LRR genes belonging to clusters are indicated by vertical bars. Heterogeneous clusters are indicated by an *. Click here for file

Additional file 18 **Detailed view of potato chromosome 12**. Genes encoded by the positive DNA strand are depicted on the left hand side of the chromosome, whereas those encoded by the negative strand are shown on the right. NB-LRR genes belonging to clusters are indicated by vertical bars. Heterogeneous clusters are indicated by an *. Click here for file
