# Supplementary figures and images for: Identification and localisation of the NB-LRR gene family within the potato genome
Source: BMC Genomics. 2012 Feb 15;13:75. doi: 10.1186/1471-2164-13-75 (PMC3297505; doi:10.1186/1471-2164-13-75)

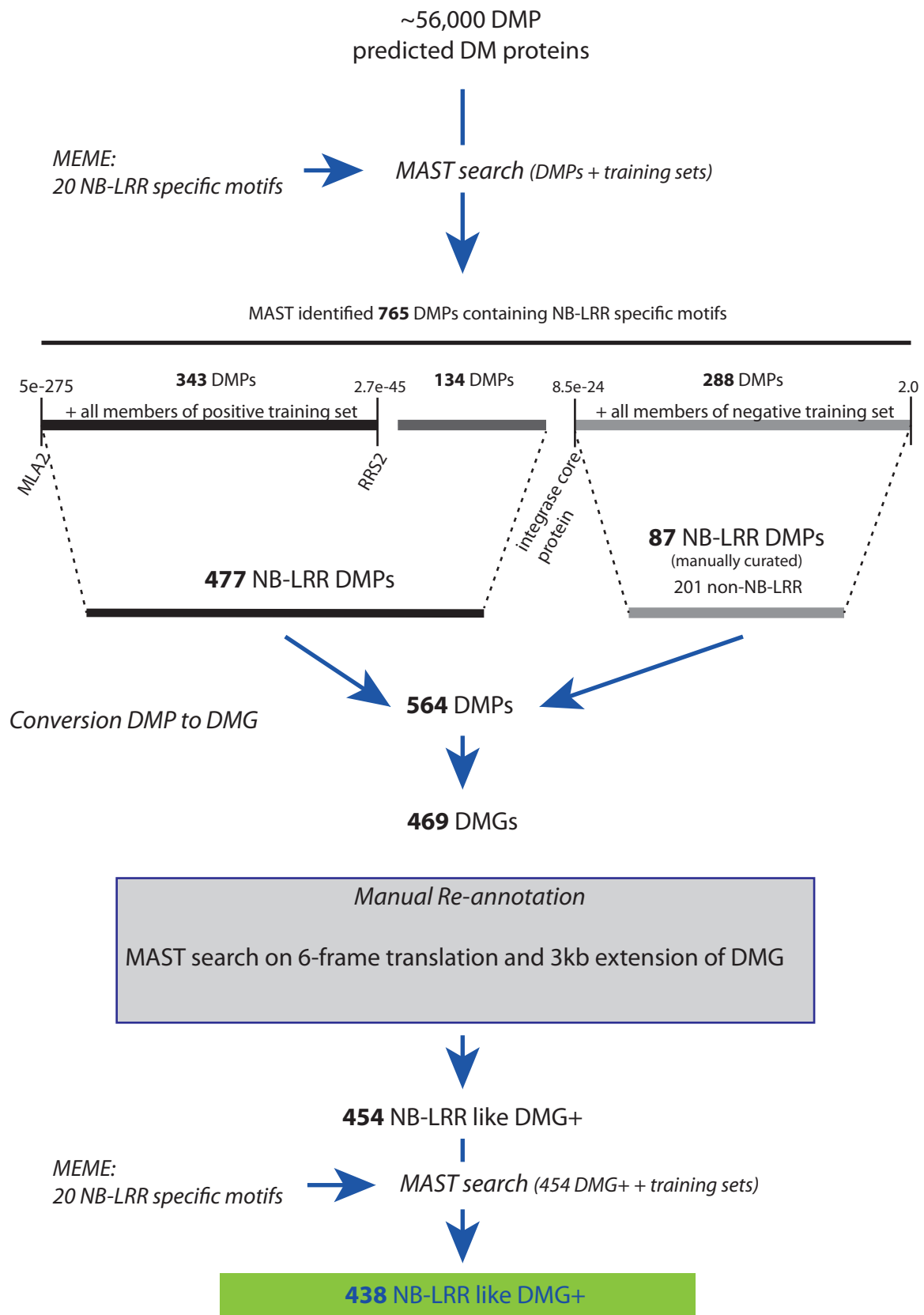

Supplement: Additional file 2 — Graphical MAST search output. Graphical overview of the MAST search output ranked according to the E-value scores obtained for MEME motifs. By including DMPs that yielded an E-value score of up to 2.0, 765 proteins were identified. Within the E-value range of the negative training set, 87 sequences encoded for very short DMPs and contained additional NB-LRR gene associated domains in the extended DMP+ sequence. [file 1471-2164-13-75-S2.PDF]

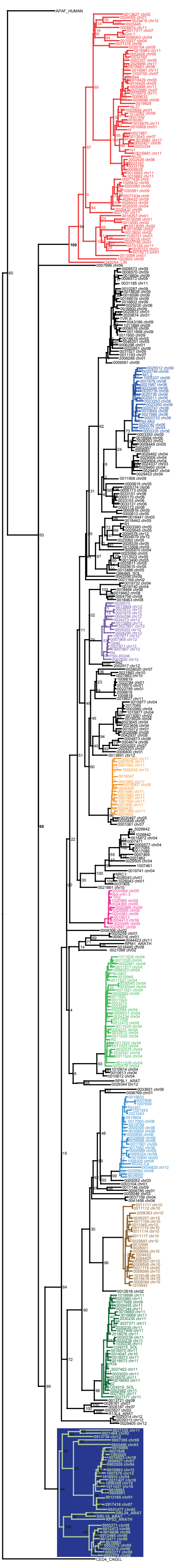

Supplement: Additional file 6 — Detailed phylogenetic analysis of the DM NB-LRR NB-ARC domains. The NB-ARC domains of TNL and CNL type gene products were used, alongside selected NB-ARC domains from functional resistance genes, to study the phylogenetic relationships between them. [file 1471-2164-13-75-S6.PDF]
