# Supplementary material for: Identification and localisation of the NB-LRR gene family within the potato genome
Source: BMC Genomics. 2012 Feb 15;13:75. doi: 10.1186/1471-2164-13-75 (PMC3297505; doi:10.1186/1471-2164-13-75)
Supplement: Additional file 4 — FASTA sequences for the re-annotated DM NB-LRR coding sequences and the conceptual translations. This file contains the re-annotated coding sequences for identified DM NB-LRR genes, as well as the derived amino acid translation. IDs correspond to the original DMG identifiers provided by the PGSC. [file 1471-2164-13-75-S4.DOC]

>PGSC0003DMG400019970

ATGGCGGCGTATGCTGCTGTGACTTCACTTCTGCAAACCCTGGATCATTTATCTCAAACTCATACATCACATTCTCTTCTTTACAAAAAAGAGCAGACTGAAGTCCTCAGTGAGAAGTACACCTTCCTTAAAACCTTTCTTGAGGATTTTACTAATATTTTCAATGAAGATATAAAGATGAAGCATCTGGAAAGGATGATCCAAGAAGCTGCTAATGGAGTGGAAGATACCATCGACTCACATGCATATGATAGCTCTGTCGTAGTACAAAGCAAGCGAGTACGAAGAAAGGCTGATATGATCTTTCATCAAAACTTGGAATATGCAATTGAGGAAATTGGTTTGATACAAAGAGAGGTAATGAAAAAGATAAAGGTCAGCAGTTTTAACAGAAAAATCTCACATTCAAGAGATACTTCNNNNNNNNNNNNNNNNNNNNNNNNNNNNNNNNNNNNNNNNNNNNNNNNNNNNNNNNNNNNNNNNNNNNNNNNNNNNNNNNNNNNNNNNNNNNNNNNNNNNNNNNNNNNNNNNNNNNNNNNNNNNNNNNNNNNNNNNNNNNNNNNNNNNNNNNNNNNNNNNNNNNNNNNNNNNNNNNNNNNNNNNNNNNNNNNNNNNNNNNNNNNNNNNNNNNNNNNNNNNNNNNNNNNNNNNNNNNNNNNNNNNNNNNNNNNNNNNNNNNNNNNNNNNNNNNNNNNNNNNNNNNNNNNNNNNNNNNNNNNNNNNNNNNNNNNNNNNNNNNNNNNNNNNNNNNNNNNNNNNNNNNNNNNNNNNNNNNNNNNNNNNNNNNNNNNNNNNNNNNNNNNNNNNNNNNNNNNNNNNNNNNNNNNNNNNNNNNNNNNNGTAGATGTTGCATCTTGTGCTTGCTCTGGCAATTCCCTTCACCAGATGCGTTTTCTGAGCATGGAGGAAAGCTGGACTCTTTTACGCGATAAGGTTTTTGGGAATGGTGGTTACCCTCCGGAATTGGAGAAAATTGGGAGGTACATTGGCCATCAGTGTCAAGGGTTGCCACTTGCAGTTGTTGCAATTGGTGGACTACTTTCCAAGATGAGCAAGGAAACAAGTTCTTGGGAGAATGTTGCAGAAAAGGTAGGATCACTTATGACCAGCGACACTATGGATTGCTTGAACATACTTTCGTTGAGCTACAACCACTTGCCTCAGTACTTGAAAACATGCTTCCTTTATATGGGAGTTTTTGCAGAAACTCGTGAGATTCCAGTCTGGAAGTTAATAAAATTATGGATTGCTGAGGGCTTCGTCAAGAGAGTTAATCATAAAAATCTAGAAGATGTGGCAGAGGAAAATTTGAGGGAGTTGGTTGATAGAAGTTTGGTTTTGGTGGGGAAGCATACTTCCCTAGGAAAAATCAAGACTTGTAAGATGCATGATCTCGTTCGAGATATGTGTTTGAGAGAAGTCCAATATGAGAATTTTATACACTTCAAAACGAGGTATGACTATGATGATCTTCTAGAAAACATTAGTTGTCTACGTCGTGTAGCTGATTTCCATAAGTATACACACAGTGGACTTTCTAAACTTATGCCACTCACACGTTCTGTTTTCTTCAAATATCCATACTTTTTTTGTTACCTCTCACAGTCGAGCTTTAGATTACTGAGGATATTAGATGTTTGCTTTACATGTTTTTTCTCCTGTAGTCAGTCCGTATCAGAACTTGTTCATTTGAGGTATATTGCTTGTCCGTCCTTCAATGGTCTTGTGCAATCACTATGCAAGCTACGGAATATACAGAACTTAGTTATTCATGATTCACATCCATTTGGTTCAAGTATGAGAACCCAGTCCCTACCATGGGAAGTTGTGAATATGCCTCAACTAAAACATATCCATACAAAAAAGTTGAGTCTTTTCATATCCCCACCTACATCAGTATTAATTTCTGAGAGGGAGAATCACTTACAAACGTTAACCGGATTGATGCCCTCATCTTGTAACGATGAGGTGTTTCTACGAATTCCAAATTTGAAGAAGTTGGGAATTTTAATTGTTGATGAATCAGACACTATTCAGAAGTGTTATTGTCTTGATAATCTTGTACATTTAACTCAGCTTGAGAAGCTCAAAGTTGAGGTCAGCGAGAGTTATCTGCTAATTTTTATGCGGATTAGCGAAGGTTGGGTGGACATTCCACATTGTGACAATTTCCCACCAAACCTTAAGAAGTTAACATTGTGCAGAACGCACCTACAATGGGAAGACATGAACATTCTCAGTAAGTTACCTAATCTTGAGGTGCTCAAACTTAAACATCATGCCTTTCACGGACTAATTTGGAAACTAAGCCATGAAGACGAAGATGGATTCTTGAAGCTAAAGTTTTACTCTCGCATGAGTCTGAAGCAATGGGAAGCAACAAGTTATCATTTTCCAAGCCTTGAGCACTTAGTCCTAACAAATTGCCGGTACTTGGAAAAAATTCCTTTCGACTTTGCGGAGATTCAGACACTACAGCTGATTGAACTACACGAATGTAAGCGTTCTGTTCTGTTTCAGCAGAGCAAATACAAGAGGAGCAACAAAGCTTGGGAAATGATGACCTCGTTATTCATGCGAACTCTATACGTGAGTAAACATTAA

>PGSC0003DMG400022785

ATGGCGGCGTATGCTGCTGTGAATTCACTTTTGCATACGCTGGATAATCTATCTCAAACTCATACATCACATTCTCTTCTTTACAAAAAAAAGCAAACTGAAGTCCTCAGTGACAAGTACACCTTCCTTAAAACCTTTCTTGAGGATTTTACTAATATTTTCCATGAAGATGTAAAGATGAAGCATCTCGAAAGGATAATCCAAGAAGCTGCTAATGGAGTGGAAGATACTATTGACTCACATGTATATGATAGCTCTGCCTTAGTACAAAGCAAGCGAGTACGAAGAAAGGCTTATACGATCTTTCATCAAAACTTGGAATATGCAATTGAGGAAATTGGTTTGATACAAAAAGAGGTTATGAAAAAGATAAAGGTCAACAAGTTCAACAGCAAAATCTCTCATTCAAGAGATACTTCATCTCAGATCCCTATGGATCAAAAAGATGTAATCATTGGACTGGATGAAGACTTGCTAAAGATAAAAGATCGACTCATCGTGCAATCATCAAGACTCGAAGTTGTCCCCATTATAGGGATGGGTGGTATTGGCAAGACCACACTGGCTAGAAGAGTTTATGATGATTCCTTGATTGCATACCATTTTTATGTNNNNNNNNNNNNNNNNNNNNNNNNNNNNNNNNNNNNNNNNNNNNNNNNNNNNNNNNNNNNNNNNNNNNNNNNNNNNNNNNNNNNNNNNNNNNNNNNNNNNNNNNNNNNNNNNNNNNNNNNNNNNNNNNNNNNNNNNNNNNNNNNNNNNNNNNNNNNNNNNNNNNNNNNNNNNAAAGAAACAGTACTACTGAGCAATTGGCTGAAAGAGTTTATCGAAATTTATCTTATTGTCCTAGATGATATGTGGTCTATCAAGGCTTGGCAGCATGTGAGGAGATCATTTCCAGATGATCGTAATGGAAGTCGAATCGTGTTGACCACAAGGCTTGTAGATGTTGCATCTTGTGTTTGCTCTGGCAATTCCCTTCACCAGATGCGTTTTCTGAGCATGGAGGAAAGCTGGGATCTTTTAAGCGGTAAGGTCTTTGGGAATGGTAGTTACCCTCCGGACAAAATCTCACATTCAAGAGATACTTCCTCACTGCTCCCTTTGTATCAAAAAGATATAATCGTTGGACTGGATGAAGACTTGCTGAAGATAAAAGAACGACTCACCGTGCAATCGTCAAGACTCGAAGTTGTCCCCATTATAGGGATGGGTGGTATTGGCAAGACCACACTGGCTAGAAGAGTTTATGATGATTCCTTGATTGCATACCATTTTTATGTCCGAGCATGGGCAAATGTATCTCAAGAATTTGACACTAGAGAAATTTTTCTTGGTATTCTTCGCTCCATTGGCATGGTCAACGATGAAGAAGAAAGAAACAGTACTACTGAGCAATTGGCTGAAAGAGNNNNNNNNNNNNNNGTCAACGATGAAGTAGAAAGAAACAGTACTAATGAGCAATTGGCTGAAAGAGTTTATCGAAGTTTAAAAGGGATGAAGTATCTTATTGTCCTAGATGATATGTGGTCCATCAAGGCTTGGCAGCATGTGAGGAGATCATTTCCAGATGATCGTAATGGAAGTCGAATCGTGTTGACCACAAGGCTTGTAGATGTTGCATCTTGTGTTTGCTCTGGCAATTCCCTTCACCAGATGCGTTTTCTGAGCATGGAGGAAAGCTGGGATCTTTTAAGCGGTAAGGTCTTTGGGAATGGTAGTTACCCTCCGGAATTGGAGAAAATTGGGAGGTACATTGGCCATCAGTGTCAAGGGTTGCCACTTGCAGTTGTTGCAATTGGTGGACTACTTTCCAAGATGAGCAAGGAAACAAGTTCTTGGGAGAATGTTGCAGAAAAGGTAGGATCACTTATGACAAGTGACACTATGGATTGCTTGAACATACTTTCTTTGAGCTACAACCACTTGCCTCAGTACTTGAAAACATGCTTCCTTTATATGGGAGTTTTTGCAGAAACTCGTGAGATCCCGGTCTGGAAGTTAATAAAGTTATGGATTGCTGAGGGCTTCGTCAAGAAAGTTAATCATAAAAATCTAGAAGATGTGGCAGAGGAAAATTTGAGGGAGTTGGTTGATAGAAGTTTGGTTTGGTGGGAAGCATACTTCACTAGGAAAAATCAAGACTTGTGTAAGATGCATGATCTCGTTCGAGATATGTGTTTGAGAGAAGCCCAATATGAGAATTTTATACACTTCGAAACAAGGTATAACCATGATGATCTTCTAGAAAACATTAATTGTCTACGCCGTGTAGTTGATTTCAATAAGTATAGATACAGTTTTGATATTCCTAAACTTATGCCACTCACACGTTCTCTTTTCTTGAAATATCCATACTCTGTTTGTTCCCTCTCACAGTCAAGCTTTAGATTACTTAGGATATTAGATGTTTGCTTTACATCTCGTTTGTACCGTAGTATGTCCATAACAGAACTTGTTCATTTGAGGTATCTTGCTTGTCCATCCTTCAATGGTCTTGTGCAATCACTATGCAAGCTACGGAATATACAGAACTTAGTTATTCAGGATTTACGTTCATCTGGTTTACGTAGGAGAACCCAGTTCCTACCATGGGAAGTTGTGAATATGCCTAAACTAAGACATATCCATACAAAAAAGTTTAGTCTTTTCATACCCCCTACATCACTAATTGCTGAGAGGGAGAATCATTTACAAACGTTAACCGGATTGATGCCCTCATCTTGTAATGAAGAGGTGTTTCTAAGAATTCCAAATTTGAAGAAGTTGGGAATTTTAATTGTTGATGATTCAGACACTATTCAGAAGTGTTATTGCCTTGATAATCTTGTACATTTAACTCAGCTTGAGAAGCTCAAAGTTGAGGTCAGAGAGAGTTGGCTAATTTTTAGGCCGACTATCAAAGGTTGGGTGGACATTCCACATTGTGACAATTTCCCACCAAACCTTAAGAAGTTAACATTGTGCACAACACACCTAGAATGGGAAGACATGAACATTCTTAGTAAGTTACCTAACCTTGAGGTGCTCAAACTTAAAGATCATGCCTTTCGCGGACATATTTGGAAACTAAGCGATGAAGACGCAGATGGATTCTTGAAGCTAAAGTTTTTACTTCTTGAGCACATGCATCTGGAGCAATGGGAAGCAACAAGTTATCATTTCCCAAGCCTTGAGCACTTAGTCCTAACACATTGCCATTCGTTGGAGCAAATTCCTTTCGACTTTGCGGAGATTCAGACACTACAGCTGATTGAACTACACAATTGTATGCGTTCTGTTCTTGTTTCAGCAGAGCAAATACAAGAGGAGCAACAAAGCTTTGGAAATGATGACCTCATTATTCGTGTGAACTCTATCTATACTAGAGTAAACATTAATAAAAAAGGTATACACTTTTTTTAA

>PGSC0003DMG401022784

ATGGCGGCGTATGCTGCTGTGACTTCACTTCTGCATACCCTGGATAATCTATCTCAAACTCATACATCACATTCTCTTCTTTACAAAAAAGAGCAAACTGAAGTCCTCAGTGACAAGTACACCTTCCTTAAAACCTTTCTTGAGGATTTTACTAATATTTTCAATGAAGATATAAAGATGAAGCATCTGGAAAGGATGATCCAAGAAGCTGCTAATGGAGTGGAAGATACCATCGACTCACATGTATATGATAGCTCTGTCGTAGTACAAAGCAAGCGAGTACGAAGAAAGGCTGATATGATCTTTCATCAAAACTTGGAATATGCAATAGAGGAAATTGGTTTGATACAAAGAGAGGTAATGAAAAAGATAAAGGTCAGCAGTTTTAACAGAAAAATCTCACATTCAAGAGATACTTCTTCCTCACAGTTCCCTTTGGATCAAAAAGATATAATCGTTGGACTAGATGAAGATTTACTAAAGATAAAAGATCGACTCATNNNNNNNNNNNNNNNNNNNNNNNNNNNNNNNNNNNNNNNNNNNNNNNNNNNNNNNNNNNNNNNNNNNNNNNNNNNNNNNNNNNNNNNNNNNNNNNNNNNNNNNNNNNNNNNNNAAAGTCGAATCGTGTTGACCACAAGGCTTGTAGATGTTGGGTCGTGTGCTTGCTCTGGCAATTCCCTTCACCAGATGCGTTTTCTGAGCATGGAGGAAAGCTGGACTCTTTTACGCGATAAGGTTTTTGGGAATGGTGGTTACCCTCCGGAATTGGAGAAAATTGGGAGGTACATTGGCCATCAGTGTCAAGGGTTGCCACTTGCAGTTGTTGCAATTGGTGGACTACTTTCCAAGATGAGCAAGGAAACAAGTTCTTGGGAGAATGTTGCAGAAAAGGTAGGATCACTTATGACAAGTGACACTATGGATTGCTTGAACATACTTTCTTTGAGCTACAACCACTTGCCTCAGTACTTGAAAACATGCTTCCTTTATATGGGAGTTTTTGCAGAAACTCGTGAGATCCCGGTCTGGAAGTTAATAAAGTTATGGATTGCTGAGGGCTTCGTCAAGAAAGTTAATCATAAAAATCTAGAAGATGTGGCAGAGGAAAATTTGAGGGAGTTGGTTGATAGAAGTTTGGTTTTGGTGGGGAAGCATACTTCCCTAGGAAAAATCAAGACTTGTAAGATGCATGATCTCGTTCGAGATATGTGTTTGAGAGAAGCCCAATATGAGAATTTTATACACTTCAAAACAAGGTATGACCATGATGATCTTCTAGAAAACATTAGTTGTCTACGTCGTGTAGCTGATTTCCATAAGTATACACACAGTGGACTTTCTAAACTTATGCCACTCACACGTTCTGTTTTCTTCAAATATCCATACTTTATTTGTTACCTCTCACAGTCGAGCTTTAGATTACTTAGGATATTAGATGTTTGCTTTACTACTTCTTTGGACAGTAGTCAGTCCATATCAGAACTTGTTCATTTGAGGTATCTTGCTTGTCCGTTTTTCAATGGTCTTGTACAATCACTATGCAAGCTACGGAATATACAGAACTTCACATCCATTTGGTTTAAGTATGAGAACCCAGTCCCTACCATGGGAAGTTGTGAATATGCCTCAACTAAAACATATCCATACAAAAAAGTTGAGTCTTTTCATATCCCCACCTACATCAGTATTAATTTCGAGAGGGAGAATCACTTACAAACGTTAACCGGATTGATGCCCTCATCTTGTAACGATGAGGTGTTTCTACGAATTCCAAATTTGAAGAAGTTGGGAATTTTAATTGTTGATGATTCAGACACTATTCAGAAGTGTTATTGTCTTGATAATCTTGTACATTTAACTCAGCTTGAGAAGCTCAAAGTTGAGGTCAGCGAGAGTTATCTGCTAATTTTTATGCGGATTAGCGAAGGTTGGGTGGACATTCCACATTGTGACAATTTCCCACCAAACCTTAAGAAGTTAACATTATGCAGAACACACCTACAATGGGAAGACATGAACATTCTCAGAAAATTACCTAATCTTGAGGTGCTCAAACTTAAACATCATGCCTTTGACGGACTAACTTGGATACTAAGCGATGAAGACGAAGATGGATTCTTGAAGCTAAAGTTTTTACTTCTTGAGCACATGCATCTGAAGCAATGGGAAGCAACAAGTTATCATTTTCCAAGCCTTGAGCATTTAGTCCTAACAGATTGCTATTCATTGGAGCAAATTCCTTTAGACTTTGCAGAGATTCAAATACTACAGCTGATTGAACTACACAAATGTATGCTTTCTGTTCTTATTTCAGCAGAGCAAATACAAGAGGAGCAACAAAGCTTGGGAAATGATGACCTTGTTATTCGTGTGAACTATATACGTGAGTAA

>PGSC0003DMG400016372

ATGGCAGCTTATATAGCGGTAATTTCTCTTCTACAAACAATTGAGCAATTTCAGCAGAGACATCCAAAACTCGTTCAAGGTCAAACTGCTAAAACGCTCGAATCCCTGCATGGTACTGCTCAATATTTTCAAAATGTTGTTGATGAAGCTAGCAAGAGTAGATTTGATCTCGAAAAAATTAAGTCTTTGGAGGAAATAATAAGAGATGCTGTTAGTTATGCAGAAAATGTTATTGAAATGAGCTGGACATGTGGAAATGCATCTGAAAGTTCACTAAACAAGAATTTTCTACCGGTTGTTGAAAGAATCGATACAATAAAGAAGGAAGTGATGGAGATTGTTTCTGATTTTAGCACTAGCACTCATGATATTGACGATGATCATATTCTTGAATCTGCCGGGGATTCATTGAGTGACACTTCTTCTAGAAGTAATCGAATGCTGCAACATTTGGAAGATGGTATCGTGCACGGACTTGATGATGACTTGGAGATAATAGTTGAAAGATTGAAAGGAGCAATGTCGGATCTAGATGTTGTCACAATATCAGGCATGGGTGGGATTGGCAAAACAACACTCGCTAGAAAAGCTCATGATCATCTAACAATCAGGTATCATTTTGACATTCGTGTTTGGATTACAGTATCTCAAAAATATGGAAGTAGAAATGTGTTGTTAGAAGCTTTACATTGCATTTCAAAACAAAAAAATATTGACATTGGAAAAGATTATGATGAGAAGGATGACAACGAGTTAGCTGACTTGGTGCAGAAAAAACTAAAGGGTCCAAGATACCTTCTTGTTGTTGATGATATTTGGAGTACGGATGTTTGGGATAACATAAGAGGAATATTTCCCGATTGCAACAATAGGAGTCGAATCTTATTGACTACTAGGGAAACAAATGTAGCAATGTATGCAAATCCTGTTAGCCCTCATAAGATGAACCTCTTGAATTTAGAAAACAGTTGGAAGTTAATTTGTGATAAGGTGTTTGGACCAAAACATGATTGTCCTCCAGAGTTGGTAGAAATCGGAAAGAAAATAGCAGAAAAATGTCAAGGACTACCCTTAACAATTTCAGTGATTGCGGGACATCTCTCAAAAGTGGCCAGGACATTAGGAAGTTGGATGGATGTTGCTCGAACTTTAGGTGAAATCATTGCTAGTCATCCAGATAAATGCTTAGGAGTGCTCGGTTTGAGTTACCACCATTTGCCTAATCACCTCAAACCCTGCTTTCTTTCTATTGGTGGTTTTCCGGAGGATTATCAGGTTGAGACTTGGAGATTGATTCGATTATGGATCGCTGAAGGTTTTATTAAGAGGCCGGGAAGTGAAAAAAGCTTAGAGGAAGCGGCAGAAGATTATTTGGAGGATCTTATCAGCAGGAACTTGATAATGGTTAGAAAAAAAAGATTCAACGGTGAGATAAAAGCATGTGGAATGCATGATCTGCTCCGTGAATTCTGTTTGACTGAAGTTGAAAGAACAAAGTTTATGCATGTTGAGACAAGTGACATAGTCCCTACTACTCTTTCAACACAAAAGCATAATCGCTTCAGTTTTCAGATTCACAGTAACTTAGATGATCATTGTTTCGAGATATTACCCTCCGTTGCTAGATCTATCTACTTATATCCAAAAAATCGATATTTTGCAAATCTTAAAGTTTTCCCCCATTTCCACCATCTCAGGGTATTGGCCGTTTTCCATGAATATGGAACTTCTTGTTCATTTCCACCTGAAATTACAAAGTTATGTCATTTGAGATATCTCCAATTTCGATCTTATGATCATTTTCTTCCTGCATCAATCTTAAAGCTTCGGAATTTGCAAACTCTAATTTTTGAGAGTCAACATGAATATACACCAGTTTTACCAGAGAATATATGGATGATGAGGAATTTAAGGCATTTACATGTGAGGAAAGCCTGCACTTTACTACTTCCTAGTAGGAATAGTATCTTAAATTTAGAGGAACTTTCTAACTTATGTTTCTCCTGTTGTACAAATGAAATCTTTTCTGGCATTCCCGATCTAAAGAGATTGATCGTCCATCATCGATATTCATCTGCTTTTTCAGTCAATGACATCACTGATATGTCCAGCTTGACAAAACTCGAAGCATTGAAGTGTGTTAGCGAGTACTTTTGGTTTTCAACGCCTCCGATCTCCATCAAGCAGTCTTTTTTCCCAAAATCACTTAAGAGGTTGACTTTAGACGGCGGGTTTTGTTTTCCTTGGGACGATATTTCAACTCTTCTTGTCATGTTGCCAAATCTTGAAGAGCTCAAACTTAAAGATCACGCAGCCAACGATCATGTATGGAGATTAAGTGCTGAAACCAAATTCGAAAGCCTTAAGTTTTTGTTATTTCATGGCCTTAATCTTAAGTATTGGGAAGCTAGCAGCGATAACTTCCCAAATCTAAGACGCCTTGTTCTGAAGAAATGCACCAAACTTAATGAAATTCCAATAGATTTTGGGGAAATTTGTACTCTGGAGTCGATTGAGTTATATCAATGTGGCGTTGGTGCTGAGGAGTCTGCAAGAAAGATTGAACAAGAACAAGAGGACATGGGAAACAATTTTCTTAAGATCTTCATCCATGTAAGTTTTAGATTTCTCCAAAATAAAAAAATTCATTAA

>PGSC0003DMG400022699

ATGGCATCTTCTTCTTCTTCTTCTTTTGTGAGTAATTCACAATACTCTCCTCGATGGAAGTATGATGTGTTTTTAAGTTTTAGAGGTAAAGATACTCGTAAAACCTTTACGAGTCACTTGTACCAAGGTTTGAAATACAAGGGAATATTGACCTTTCAAGATGATAAAAGGCTAGAGCACGGCGATTCTATCTCAGAAGAACTCTTGAAAGCTATTAAGGAGTCTCAAGTTGCCCTTGTAGTTTTCTCAAAGAATTATGCTACATCAAGGTGGTGCTTGAATGAACTAGTGCAAATCATGGAATGCTACAAAGATGAAAATGGAAAAACAGTCATACCGGTTTTCCATGATGTGGATCCATCACATGTTCGATACCAAAGTGAGAGTTATGCAGAAGCTTTTGCCAAACATGAATTGCAGTTTAAGGATGATGTTGAAGGAATGCAGAAGGTGAAAAGATGGAGAACTGCTCTATGTGAGGCAGCAGATCTGAAAGGACATGACATCCGCCAAGGGGTTGAATCAGAGAATATTCAACTAATTGTTAACCAAGTTTCGTCCAAATTATGCAAGACTTCAGTATCTTATTTGCGAGATGTTGTGGGAATAAACATTCATTTAGAGGAAGTGAAATCCCAACTAAAGCTTGAAATCAATGATGTTCGTATTGTGGGGATCTGGGGCATGGGGGGGATTGGTAAAACGACAATAGCAAGGGCAATTTTTGACACACTCTCGTATCAGTTTGAAGGTTCTTGTTTCATTGAGGATATTAAAGAAAACAAATGTGGAATGCATTCTTTGCAAAATATCCTTCTCTCGGAGCTGTTAAGGGAAAAAGATAATTACGTGAATAATAAGGAGGACGGAAAGCACATGATAGCTCGTAGACTTCCTTTTAAGAAGGTTTTAGTTGTGCTTGATGACATAGATCACAGAGACCATTTGGATTACCTAGCAGGGAATCCTAGTTGGTTTGGTGACGGAAGTCGAATTATTGCAACAACTAGAGATAAGCATTTGATTGGGAAGAATGATGTAGTATATGAAGTGTCTACACTAGTTGACTGTCACGCTATTAAATTGTTCAATCAATATGCTTTCAAAGAAGAAGTTCCCGATGAACGTTTTGAGAAGCTTTCGTTGGAGGTTGTACGTCATGCTAAAGGCCTTCCTTTAGCCCTGAAAGTGTGGGGTTCTTTCTTACATAAGAGGGACATAACTGAGTGGAGAAGTGCTATAGAGGAAATGAAAAATAACTCCAATTCGGAAATCGTTGAGAAGCTTAGAATTAGCTATGATAGACTGGAGACCATACAACAAGATATATTTCTAGATATAGCATGTTTCTTTCGAGGGACAGCAAAAGATCACATCATGCAAATTCTTGAGAGCTGTTATTCTGGAGCAAATATCGGGTTGCGTGTCCTAATTGACAAATCTCTCATGTTCATTTCTGAAGATAATACGATTCAAATGCATGACTTGATACAAGAGATGGGTAAATATGTGGTGAAAATGCAAAAATATTCGGGAGAAGCTAGCAGATTATGGGACAATGAAGATTTTGAAGAAGTGATGGTCAACGATACAGTAAGTAGACTAAACAAAGCAATACTATTATATTTCCAGTTTTCATTCAAAATTTGTTTATCTTATGTTATTAAGCCACAAATTAGTCTAGTGAAAGAATACTTTCTGTGTTCTAAACAGCATTTGCCGTCTCTAACGACGCTAGATCTCAGCTACTCTAGAAACCTGGTGCGAACACCAGATTTCACGGGGATGCCAAATTTGGAGTATTTGAATCTATCATATTGTAGTAATCTTGAAGAGGTTCACCATTCCCTCGGATGTTCAAGAAAACTCAATTTGTTATATTTGTGTGTTTGTACACTCCTAAAGAGGTTTCCATGTGTTAGCGGGGAATCTCTTGAATATCTGTATCTACATGATTGCTATAGCTTAGATACATTTCCAGAAATCCTCGGAGGAATGAAGCCGGGGTTGGAGATTAAGATGGAACGGTCTGGGATAAGGGAACTACCATCATCTATTCAGTACCTAACTCACATTACCAAGCTGAACTTAAAAGGCATGAAAAAACTTGTATCTCTTCCGAACAGTATTTGCATGGTGAAAAGCTTGGCGGAGATAGATGTGTCGTACTGCTCGAAACTTGAAAGCTTGCCAGAAGAGATAGGTGATTTATTAAACTTGGAGAAGCTTGATGCCACATACACTCTAATTTCACGACCTCCATCTTCTATCGTCCGTTTGAACAAGCTTAGGTTCTTGAGTTTTGCAAAACAATCTTCAGAAGTAGGCCTCGAAGATGGAGTGTTCTTTGTGTTCCCTCGAGTGAATGAAGGGTTACGCTCATTGGAAAATTTGGATCTTTGTTACTGCAATCTAATAGATGGAGGACTTCCGGAAGACATTGGATCCTTATCCTCTTTGAAAAAGTTGTATCTCATTGGAAATAATTTTGAGTATTTGCCTCGAAGCATAGCTCAACTTGGTGCTCTTCGACTCTTGTACCTATCAGATTGTCCTAACCTTAAAGAGTTCCCTCAGGTGAACAACGGATTACGCTCATTGGAAGATTTGGATCTCAGTCATTGCAATCTAATAGATGGAGGACTTCCGCAAGACATTGGATCCTTATCCTCTTTGAAAAATTTGCATCTTGATGGAAATAACTTTGAACATTTGCCTCGTAGCATAGCTCAACTTGGTGGTCTTCAATACTTGAACGTATCAAATTGCACGAGGCTTAAAGAGTTGCCAGACTTCATGTTGATGCCTGATTTGTATGTTTTGCATCTGATAGATTGTATGAGTCTTGAAGAGGTTCATCATTCCCTGGGATTTTTCGAAAAGCTCACTCACTTATTCTTGTATAACTGTAAACGCCTTAAGAGGTTTCCAGGTATGTGCATCGATTCCCTTCAATGTATCCGGACATGGGGTTGCTCTAGTTTAGAAAGTTTTCCTAAGATCATCGGAAGCATGAAAGTGGAATCAGAGATTCACATGTTAGACAGTGTAATGTGTGATCTAAATTTGAATAGTTCATTTCCACATTCCTTGTCTCAGAGGATCGTTTCGTTGCAGCATGACATCTCTGATTCAGATTCCTTATCACTAATTAGAGTGTTTACGATTGATCATCCCGAGAAGAAGATCCCAAGTTGGTTCCACCATCAAGGAATGGATACAAGTGTTGTATCTGTCAATTTGCCTGAAAATTGGTTTGTGCCGGATAACTTCTTGGGATTTGCTGTATGTTACTCTAGTGAACTAATTAACATCACAGCTCACTTGATTCCGTTACGTGATGATGGGCTGTCGTGGATGACCTTGAAACTAAACTTATCCCGCGGTTCAAAACGTGATGTAGCATACTCTGATTATTCGGAATGTGAGACAGAATCATCTGACCATTCAGAATGTGATACTGAACCTACTCTTCATTATTTCTTGGTACCTTTTTCGAGCTTATGGAATACATCTAAGGCAAATGGGAAAACACCAAATGACTATGGGCTCATTACGTTATCTTTTTCTGGAGAAATGAAGAAGTTTGGATGTCGTTTGTTGTATAAAGATGAGCCTGAGCTTGTTGAGACCTTGTTACAGATGAGGGAAAACAACGATGAACCAACAGAACGTTACATTGGGATCAGGAGCAGTAGATATAACAATAGTGAACATCATGACTCCGTCACCAATGAAGCCAGTTGCAATGATGTATCACAGATATAA

>PGSC0003DMG400020872

ATGGTTCAAAATGAAATTGAGGAAATGTTAGATCACCTGAGAAGGATCAAGAGTGGGGGTAATCTGAGTAGAGTCAAGATTGGTAAAATTGAGATACTTGAAATGGTGCTAAGAGTTTTTAGAACCTTTGTAAAGTATCATCATGTTCTTTTGCCGGATCCTTTAGACAAACTTATAAAGAACGCCAAATGGACCGTGAACATGCTTCAGTGGGCACTTAGTAGAATTCCAAAGGAACGTAAAACTAACCTTAATTTGGAAAGACTAGAATCACATTTGTTAGAATTTTTTGAAGGTAATACCAGTTTAAGGTACAATTATGAGTTGAATGATTTTGATCTGTTGAAATATATGGATTGCCTCGAAAAGAATCTAAATGATGTACTGATGATGTGCCTGGAAAAGGGTAGGTACTCGCCTCTTAAAATAAATTGCATTATAAAGCAACTGAAAATTGTTCAAAAGAAAATGAGATTTTTGAGATACTTGTATGCCACTGAGATAAATGGTTTCATCAACCATGAGAAGCTGGAATGTTTGGAGACTCGAATTGAGTTCATGGCCGACAATGTGGGACAATATTGTCTTGCTGTTTTAGTTTACGTTGCTGCGATGGGAGTTAAGGATAAAGATGATATCTTGAATAAACCTCCTTATCTATTATCGTTGATTGTGCTTGTGGAGATGGAAATGAAGAAGATTTTTCTTGGTGAACTAAAGGCTTCAAAGTTTACTCTATCAAAAACTTTCAAGAACAAGAAATTACCGAAAGGATTTTCACATCATCTCCACAGTCTGCTGATGTATCTCAGAAACAAAAAGCTCGAGAACTTTCCTAATAATATCTCTGCTCAAAATATTGATGTGGCAATAGAGTTCTTGTTGGTTTTCCTTGATGCTGATATGTCAAATCATGTTATAAACGGTAGCTGGTTAAATGAGGTTATGGAAAAGGTTGGAGCTATAGCGGGTGATGTTATATATGTAATTCAAAAACTTCTTCCTAGCTCTATAAACAAAGATGGCACTAGCAAAATAAGTCTTTGCTCGATACAAATATTGGAGAAAACGGAAGATCTGAAGGCACAAGTGGAGACTTACTACAAATCCTTAAAATTTACTCCATCTCAGTTCCCCACCTTTGGTGGATTGATCTTTCTGGATTCTCTTTTAAGGAAACTGAATGAGATGTCGAAATCTAAATTTGGTTTAGATTTCTTGATGAAACCTCTTTTAGGTAATTTAGAGAAAGAGCTATCAACTCTTACATCCATTTTAGAGAAGGAGTTGTCATACATTTTCAAAGATGAACATAGAATGTTTAAAGATCTTCAGAGACGTCCTATCAATTTGGCATATGAAGCTGAGGTTTCCATTGACTCTATTCTTGCTCAGTATGATGTCTTTTGGCATATTTTTTGCTCACTTCCTACAATCATAAAGAGATCAAGAAAATTAATGTGGAGGACTGAGATATGGTCGGTGGACATTGCCCTTAAGCCTTGCTATGCGGTAGTGCCATCTAAACATCTGCAAACTCGACATAGCAATCAAGTGAGTGATGAGGAGATAGTGGGTTTTGAGTTAGAAGTTGAAAGACTAATTCAGTATCTGACTCGAGGTACAAGTGAGGTTAGATGTCATACCTATTGTTGGGATGGGGGACAAGGCAAAACAACATGTGCTAGAAAGTTGTATAATAATGGCATCATCGTTTCTCGGTTTGATGTTCGAGCATGGTGCATCATTTCCCAAAAATATAACCGAAAAGAGCTATTACAAGATATTTTTAGTCAAGTTACAGGTTCCAAAGACAACGGAGATAGGGATGATGTTCTTGCCGACATGTTGAGGAAAAACTTAATGGGAAAGAGATATCTTATTGTCTTGGATGATATGTGGGATTGTATGGCATGGGATGGCTTAAGGCTTTCTTTTCCAAACGTTGGAAATAGAAGCAGAATAGTAGTAACAACTCGACTTGAGAAAGTGGGTAAGCATGTGATGCACCATATTGATCCTTACTCTCTTCCATTCCTCACAACAGAAGAGAGTTGCCAATTGTTGCAGAGAAAAGTATTTCAGCAGGAAGATTGCCCGCCTGGACTTCAAGATACGAGTCAAGCAGTTGCAGAAACATGCAAAGGACTGCCTCTAGTGGTTGTCTTGGTAGCTGGAATAATCAAAAAAAGGAAAATGGAAGAATCTTGGTGGAATGAGGTGAAATATGCTTTATTTGACTATCTTGATCGTGAGTCGGAAGAATATAGTCAAGCGACTATGCAGTTGAGTTTTGATAACTTACCCGATTGTTTAAGGCCTTGTCTTCTCTATATGGGGATGTTTTTGGAGAATGCAATAATTCCAGTTTCTAAATTGATAAGTTTATGGATAGCGGAAGACCTTGTGGAGAACATTAAATCTGCTGAAGATTACTTGATGGATCTCATTAGCAGTAATGTGATAATGGTTTCAAAGAGAGAATATAATGGTAAGGTCAAATATTGTCACGTTCATGATGTAGTGTTTCACTTTTGCTTGGTGAAGAGTAGAGAGGAAAAGTTTATGCTGACAGTGAAGGGCCAGTTTCAACCTTTGGATTGGAAGGGAAGTCGGGTTAGCTTCAGTTGTAGTGAAGAGCATTCCAAAACACAGAAGCATTTCCACCAACACTTGAGGTCACTGATAATGACCAATAGAGGAAAATCTAATGATGGAATTCCCTTTTGTCAGATTAGTCAATTGCGACTTCTTAAGGTCTTGGATTTGAGTTCTCATAGTGTGGATCGTTTGTCGTCAGCTACATTCAAACCACTATATCACCTGAAGTACCTCGCAGTTTTGGCAGATGAATTTTATTTTCATCCAAAATCACATCTCCTCTGTCTTGAAACTTTAATTGTGAAGAATGATTAGATACATATAGTACTGTTACCAACGTCTTTTTGGGAAATGGAAAAATTAAGGCATGTTGAGATTTATAATGCAAAGTTTGATGAGCAGGGGATGTTTGAAGGATCCTCTAAATTGGAAAATTTGAGGATATTAAGGAATGTTCGATTTCCAACTAAGGAAATTGATAGGGTGGATGTGTTATTAAAGAGGTGTCCTAATCTTCACCAACTTCACATTGGCTTTTGGGGCATTGTTGATTCTGGAGAGCCTTTTTGTCCCAAATTGGAGAATCTTATGCAGCTTCAAGTACTTCGCCTTTTCTTTCCGTGGACCATAATTGTATCTGGGTTACAGTTGCCTTCAAATATAAAGAAGTTGATACTATTAGGGACTGATATAGAAAGCGTGATTTCCTTCATTGTCGGACTACCAAGACTGGAGCAGGGGCGGATCTACATGGATCCAAGGGGGGTTCATCCAAACCCCTTTCGTATACTAATAGTGTGCTTACTTATCTTTTTGAACCCCCTCGATGGAAATCCTGCATCCGCCACTGGACTGGAGTATCTCAAATTAGAAGATGTGGATTTTACAGAAAATGAACTGCGTTCCTTAGAGTGGTTCCTTAGAGATATCACGTTCCATAAACTTAAGTTCTTGAAACTAGTGAACTTAAATATCTCAAGATGGGATACCTCGGAGGAATCCTTTCCCCTGCTTGAAACACTAGTTGTAAAACGGTGTGACAATCTTGAGGAGATTCCCCTTAGCTTTGCAGATATTCCAACATTGAAACAGATTAAGTTGATTTACTACAAGAATGAATCTCTGAAGGCTTCAGTTGTGAAAATTAAGGAAAATGTCAAAGAGAATGAAGGAAACGACCGTATAGACCTCATCACAATCAAAGTAAGTAGAGTAAAACTCCTACGTTCTGTTTTTGAGTATGTGCCTCTAATGTGCAAATAG

>PGSC0003DMG402020874

ATGGCTCAAAATGACATTGACAATGTGTTAGATCAACTAAGAAGGATCAAGAGTGGCGGTGATCTGCATGTTGACAAGATTGATGAAATTGAGAGACTTCAAATGCATATAGGAATTTTTAGAACCTTTAGCAAGTACAATCATGTTCTTTTGCCTAAATTTGTTGTCAAAATCACAAAGAAGGCCAAATTGATTGTGGAAATGATTCACTCAGTTTTTGGTGGAATTCCAGATAAATGCAATACTAACCTTAATCTGGAAAGACTGGAATCACAGTTGCTGGAATTCACTGAAGGTAATACCATTTTAAGGTACAATTATGAGTTGAATGATTCAGATCTACTAGAATATATGGATTGCATTGACAAGAACCTAAATGATGTTTGGATGTGCCTTGACAAGAACCTAAATGATGTAATACAGATGTGCCTGAAATTGGGTACGACTGATCATTCTTCAACAAATGAACAATTCCTTCAGTATAATCGATTTTTTGAGCAAGTGAAAATCATTCAAAAGAAGATGAGAATTTTGAGAGACTTATATGCCACAGAAATAAATGGTTATGTTGACTATGATAAGTTGGAAGGTTTGGAGACGCGATTTCAGTTCGTGGCTGACAATGTAGGACTGTTTTGTATTGCTCTTTCTTATAAATTAGATGAGGATGAAGATGATACAGATGATATCGAGAGTAAGTCTCCTTATCTTCTATCCCTGGTTGTGCTAGTGGAGCTGGAAATGAAGAAGATTTTTCTTGATGAACTGAAGGGTTCAAACTTTACTCAGTCAAGAACTTTCGTGGATGAGAAATTACCAATAAGATTTTCATATCATCTCTATAGTCTGCTGATGTATCTCAGAAACATAAAGCTGGAGAACTTTCCTAATAATGTCTCTGCTCAAAATATTGATGTGGCAATAGAGTTCTTGTTGGTTTTCCTTGATGTTGATGTGTCAAATCATGTTATTAATGGTAACTGGTTGAATGAGGTTATGGAACAGGTTGGATCTATAGTGGGTGATATTCTATACGCAATTCAAAACCTTCTTCCTAGCTCTATAGCAAACCTAAGTCTTGGCTCGATACATATATTGGAGAAAACTAAAGATCTAAAGGCACAAGTGGAGACGTACTACAAATCCTTAAAATTTACTGCATCGCAGTTCCCTACCTTTGGTGGATTCAACTTTTTGGATTCTCTTTTAAGGAAACTGAATGAGATGTCGAAATCTAAATCTGGTTTAGATTTCCTGATGAAGCCACTTTTAGATAATTTGGAGAAAGAGCTATCATCTCTTACATCCATTTTAGAAAAGGAGTTGTCATCCATTTTCAGAGATGTCGCAATGGTGCACCATGAACACAAAATTCTCAAAGATCTTCATTGGTGTACTATCAGTTTGGCATATGAAGTTGAAGTTTCCATTGACTCTATTCTTTCTCAATATGATATCTTTTGGCATATTTTTTGCTCACTTCCTACAATCATAAAAGAAATCAAGAAAATTAATGCGGAGGTAACTGAGATGTGGTCAGCGAACGTTGCTCTTAAGCCTAACGATGTGGTAGCGCCATCTAAACATCTGCCAACTCGACATAGCAATCCAGTGACTGATGAGGAGATAGTGGGTTTTGAGATAGCAACAGAAAAACTAATTCAGTAACTGACTCGAGGAACAAGTGAGCTAGATGTCATCCCTATTGTTGGGATGGGGGGACAAGGTAAAACAACTTGTGCTAGAAAGTTGTATAATAATGACATCATTGTTTCTCGGTTCGATGTTCGAGCATGGTGCATCGTTTCCGAAGAATATAATCGGAGAGAGATATTACAAGAGATTTTCAGTCAAGTTACGGGTTATAAGGACATGGGAGATGAGGATGACGTTCTTGCTGACATGCTGAAGAAAAAATTAATGGGAAAGAGATATCTCATTGTCTTGGATGATATGTGGGATTGTATGGCATGGGATATCGACTTGAGGCTTTCTTTTCCAGATGATAAAAAAGGAAGCAGAATATTAATAACAACCCGACTTGAGAAAGTGGATAAGCAAATCAAGTGCCATATTGATCCTTACTCTCTTCCATTCCTCACAACAGAAGAGAGTTGCCAATTGTTGCAGAGAAAAGTATTTCAGCAGGAAGGTTGCCCACCTGAACTACAAGATGTGAGTCTAGTAGTTGCAAAAAAATGCAAAGGATTGCCTCTTGTTGTTGTGTTGGTTGATGGAATAATCAAAAAAGGGAAAATGGAAGAATCTTGGTGGCATGAGGTGAAAGATGCTTTATTTGGCTATCTTGATCATGAGTCTGATGAATATAGTCGGGCGACTATGCAGTTGAGTTTTGATAACTTATCAGACTGTTTAAAGCCTTGTCTTCTTTATATGGGGATGTTTCCGGAGGATGCAACAATTCCAGTGTCTAAATTGATAAGTGTCTGGACTGAGGAAGGATTCGTGCAGAACATTGAATCTGCAGAAGATTACTTAATGGATCTCATTAGTAGTAATGTGGTAATGGTTTCAAGGAGAAGATATAATGGAAAAGTCAAATACTGTCAGGTTCATGATGTAGTGCTTCACTTCTGCTTGGAGAAGAGTAGAGAGGAAAAGTTTATGCTAGCAGTGAAGGGGCCATCTAACCAGTTTCAACCTTTGGATTCGGAAGGAAGTCAAGTGAGCTTCTCTTTCGGTGAATATATTTCCAAGTTTGCTTCTCTGGGCTTCAAAACACAGAAGCCTTTCCACCAAAACTTGAGGTCACTGATAACGACCAATCAAGGAAAATCTATTGAAGGGATTCCCTTCCATCAGGTTAGCGAATTGAGACTTCTTAAGGTCTTGGATTTGAGTTCTCATAATGTGGATCGTTTGTCGTCAGTTACTTTCAAACCACTAAATCACCTAAAGTACCTTGCAGTTTGGGTAAATAAATATGATTTTCATCCGGAATCACATCTGCCCCATATAGAAACTTTAATTGTGAAGAATAATTTTAATACAGTACTGTTACCAGCGTCTATTTGGGAAATGAAGAAATTAAGGCATGTTGAGATTGCTGAAGCTGTGTTTGGTAAGCAGGGGATTTTTGAAGAATCCTCTAAATTGGAAAATTTGAGGATATTAAGGAATGTTAGATTCCGAATTGATGAAGTTGATAGGGTGGATGTGTTATTAAGGAGGTATCCTAATCTTCAACAACTTCACATCGGCTTTTGGGGCAATGATGATTCTGCAGAGCCTTTTTGTCTCACATTGGAGAATCTTACCCAGCTTCAAATACTTCGCCTTTCCTTTCAGTGGTCCCACATTGTATCCGGGTTACAGTTCCCTTCAAATTTAAAGAAGTTGGTACTATTCAGGACCGACATAAAAAGTGTGATTTCCTTCATTGCAGGACTACCAAGCCTGGAGTATCTCGAATTAAAAGATCGGTATCTTATAGAAAATGTATCGCATTTTTCTCAATCAGAAGAGTGGTGCATTGGAGATATCATGTTCCATAAACTTAAGTTCTTGAAATTGGTGAACTTACGTATCTCACGGTGGGATGCCTCGGAGGAATCCTTTCCCCTTCTTGAAACACTTGTTATAAAAGTTTGTTACAATCTCGAGGAGATCCCCTTTAGCTTTGCAGATATTCCAACACTGAAACAGATTAAGTTGATTTGGTGCAAGAACGAATCTCTGAAGGCTTCAGCTGTGAGAATTAAGGAAGAAGTCGAAGAGAATGAAGGAAACGACCGTATAGATCTCATCACTATCTAA

>PGSC0003DMG400024055

ATGTCTTCTACTTGTACTCAAAATTGGAAGAATGATGTTTTCTTGAGTTTTAGAGGTGAAGATACGCGTAAAACTTTTGTTGGTCATCTCTACTATGCTCTAAAACAAAAAGGAATTCATACATTCAAAGATGATGAAAGATTGGAAAGAGGAAAATCAATTTCACCTGAACTTGTTAAAGCTATTGAAGAATCAAGATTTGCTATTGTTGTATTCTCTAAGAATTATGCATCATCCACTTGGTGTTTAGATGAACTTGGGAAGATCATGGAATGTAAAAAAGATTTTGGACAAACTGTGATACCTATATTCTATGATGTAGATCCATCACATGTAACTAAACAAAGTGAAAGTTTTGCTAAATCATTTGCTACACATGAGGAAAATTTGAAAGATGATGTTGAGAAGGTGTTATGCTGGAGGGATGCATTTCGTCAAGCAGGCAAAATAGCTGGAAAATCTCATTTCAAGTTGTTTTTGCAATTTCATGACAGGCATGAATCTCTTTGCATTCAGCATGTTGTTGAAGGCATACTGAGTAAATTATGTCAAGTTATTTCAACCATTGAAAATGATTTGGTGGGGATGGAATCTCGAGTGCAGGAAGTAAGTTCATTACTAAGGATGGAAACACGCAATGTTCGTTTTATTGGTATTTGGGGGATGGGCGGCATTGGTAAGACAACAATTGCAAGTGCTGTGTTTGGCAAATATTCGGGCCAATTTGAAGGTGTTTGTTTTCTTGATAATGTTGTGGAAATGCAAAGGACACGTGGACTGCAATATCTGCAAGGTGTTCTCCTCTCGAAAATCCTCAAAGTAAGTTTAACTATAGCAAGTGTGTATGAAGGCATGAGAATCATAAAGGAGAGGCTACGCTCAATGAAAGTTTTGATCATTCTTGATGATGTAAATCAAAAGGACCAATTAGAAATGTTAGTTGGAGGGCATGACTGGTTTGGTATCGGCAGTAGAATTTTGATTACAACAACAGATAAACACTTGTTACATAATCATGTGGTGGATGAAGTGTATTCAGTCAACTTGATGACTCGCGATGAAGCAATAGAGCTATTCAGCCTACACGCCTTTAAGCAAAGAATTCCAGATAAAGATTTTGAGGAGCTAATAAATCAAGTTGTACAGAATGCCGGTTTTCTCCCTTTAGCTCTGAAAATTCTAGGTTCGTCTCTCTACGGGCTAGACAAGATTGAGTGGAAATGCACAGTGGACAGACTCAAGGATATGCCAGATCATGTTCTTGCTAAGCTTAAGATAAGTTTTGATGGGCTGAGTCCTTCTGATCAGAGACTATTTCTTGATATCGGATGCTTTTATAGAGGAAAATTGAGGAGTTATGTAGAGGAAATTCTTGAGAGTTGTGATATTGGATCTACAATAAGAGTCTTGATTGAAAAGTCTCTCTTATTTGTCTCACCATATGACACAATCGAGATGCATGATTTGATACAAGAAATGGCCTGGCACATCGTGAGACTAGGTGACACAAGAAGGAGCAGAATATGGCTTCCCGAGGACATCGAGGATTTGTTTACTGGAAATTTGGTAAGATATGTCATTGTCTCATGCATAGGATTGGACAAACTTAAAACTTTGTACCTCAGCTACTGTGAGAACTTGGAAGAAGTTCCAAGCTTTGATATGATGCCAAATTTAGAGAGAGTAAAGCTAGAGGGATGTAACAGTTTGAGAGAAGTCAGCCCATCTTTTGGAGTTCTCATGAAACTCACTTCAATGGAGCTAATTGATTGTCCAAGCCTCGAGAAGCTTCCAAGTTGTATTGAGATGGGATCCCTTAAGAGTCTCAAACTTTCTTGTCTTCCAAAATTAAGGGAATTGCCAGAAACCAAGGGGATGCACCGTTTATTGAGGTTAGAGCTGACTAACTGTCAATACCTTGAGAAGCTTCCAAATTTTGATCAGATGGAATCCCTTGAGACTCTCAAACTTTCTTGTCTTCCGAAAGTAACTACATTTCCGCCACCTGACGGGATGCACTGTTTACAGGACATTGTTATAGAATATGTTCCGATAGTAGAGCTTCCAGCATCAATCGGGAATCTTCGTTCCCTCAAACAACTAAGATTAAGTCATTGTAAAGATCTGGTAAGCATTCCAAACAGCTTTTGTTGTCTGAAGAATCTAAGAGCTCTTCTGATCTACAGCTGCAAAAGACTAATCTATTTGCCAGAAAAGATTGGTGACTTGAAACTGTTGAAGAAGCTAGTAGTATCTGGTACTTCAATTTCCCGGATACCACCTTCAGTTACAGATCTTGGTGAACTAAACTTCTTATCGTTCTCTCGTTGGTCTGGATACAGACAAGGTGCAACTTTTCTGTTCCCCCCTGCATCAGGTGAATCATCGTCATTGAGGGTGTTAAAACTAAATAAAAACAGGTTATGTAGTGCTGAACATTTTCAGGATGTTGGAGGTTTATCTTCTTTGGCTCACTTGGATTTGACTAGAAATGATGAGAGCACCAATCAACTCTTTCATTACTTAGACATAACATTTTGTGAGAAGCTTGCGTTGCCCGAACTTCCGCCATGTATAAAGGAGTTATATGCATGTGATCCTTTAATCTTGAAAAGCATCCCTGATTTCCTTACCAAATATTCAGAGCTCTATTCAGTGTCATTCACGCGGCATATCGAGAACAAAGGTGAACTGACTGATATGTTGCTTTTTGTCCTCCGCGTGATTAGTGTGGCATCTCAGGTATCGCCTCTTTCTTGTGATTTGTCTGCTTATCCAATTATGTTTATGTTGGAGCTTATACCGGTTTCTTTCTTGTAGTGTGAGAAAAGGCCGCCATTTAGCCTTTTCTTCCCTGGAGATATAAAATGGAAATGGTTCACTTATTATCGGAAAGAACAAACGAAAAGATTCTCCATTCAACTTGATCCACGTTGGTACGAAAGTAAATTCAAAGGATTTGTTATATGCTTTCGTGTACCATCGGTTATTGGTCAGAACCTGAAACCTTCAGATGGTAAATCACAACGAGGAAGTAGTCGTAGGTTCGGATGCACTAAGGTTACTGCTAAGTTAGTGCAAAGACATAACAGGCAAGAACATGATGTACTCCAGAAAAAATGTCTGATCGTTGCTCGCCAAACATTTTGCTCTCACGGTAGTAAATACGCCATTTGTTTTAGCTACATACCTTTTGTAGCATGTGAGAAAGGGAAGAAGCTGCTAAATGAGTATTGCTTCTTAGAGGCATCGATAGAACCAGACATTGCAACAAAATGGGGACTTCTTCTGGTGTATGAGAATAAAATTCAACAGATAGATCAATCAGCCATCGCGGTCCAATGTGATGTTGATTCTCGGAATTCTGACCTGTTTAGAGAATGTAATGATGACCAAGGCCAGAGAATAGAGGATGCTTCTGTTAAGAGAAGACGTCTTGATCTTCGTCAAGTGATGAAGGATTCGTGCTCCACCAGTATAGTTCCTGATCAACAAATGGAAACACCAAGCTCTTCTGCAGCACAAAGTTTCCAGCATAGGGAAGAGTCATGCTCTTCAGGACAGCCCCAAACTAGACAGCTCCCTTTAACTGATCCGCGAGTTGATGAAGCAATAGATGAGGATACCTCATGCATTGTAATTGAACAGCCTGATGTTTTTGAAGTCTCTCTAGATGAGCACAAAGATAATTCAATGACAACTGGTTCTGGCTGCTCTGAAGTATCCCAACAATTGGAGACACAATGCACTTCTGGACAATCTCAAACTCTCCGGCTCTTCCCCGATCATTCATGA

>PGSC0003DMG400016808

ATGGCATCATCTTCTTCTTTTGCGAGTAATTCACAGTACTGTCCTCAACGGAAGTACGATGTCTTTCTAAGTTTTAGAGGAGAAGATACTCGAAAAAATTTTACGAGTCACTTGTACCAAGATTTGGAAAATAAGGGAATATTCACCTTTCTAGATGATAAAAGGCTAGAGGATGGAGATTCCATCTCAGAAGAACTTGTGAAAGCTATAGAAGAGTCTCAAGTTGCAGTAATCATTTTCTCAAAGAATTATGCTACGTCAAGGTGGTGCTTGAATGAACTAGTGAAGATAATGGAATGCAAGGAGAAAAAAATTGGACACACAGTCATACCGGTCTTCTGTTATGTGGATCCATCACATGTTCGATACCAAAGTGAGAGCTTTGCAGAAGCATTTGCCAAACACGAATCGAGGTACAAGGATGATGTTGAGGGGATGCAGAAGGTGCAAGGATGGAGGAATGCCCTAGCTGCTGCCGCAAATCTAAAGGGATATGATATCCGTGACTGGCAATATTCTTTTTTTATATATAGGATTGAATCAGACTTCATTCAGCATATTGTTAGCCAAGTTTCGTCCAAATTATGCAAGACTTCAGTATCTTATTTGCGAAATGTTGTGGGAATAGATACTCATTTAGAAAAAGTAAAATCCCTATTAGAAATGGAGATCAATGATGTTCGGATTGTTGGGATCTGGGGAATGGGTGGAGTTGGTAAAACGACAATAGCAAGGGCTATTTTTGATACAAACTCAAATCGATTTGATGGTGCTTGTTTCTTGCGGATATTAAGGAAAACAAGTGTGGAATGCATTCTTTGCAAAATATCCTTCTCTCAGAACTATAGGATACAAGGTAACCACGTGAATAATAAAGAGGACGGAAAGCACATGATAGCTCATAGACTTCGGTCTAAGAAGGTTTTAGTTGTGCTTGATGACATAGATCACAATGACCATTTGGATAACCTAGCTGGAGATCTTGATTGGTTTGGCAAAGGCAGTAGGATTATTGCAACAACTAGAGATAGACATTTGATGGGAAAGAATGATGTAGTATATAAAGTGACTGCACTACTTAAACATGATGCTATTAAATTGTTCCATCAATATGCTTTCAAAGAAGAAGTTCCAGGTGAATCTTTTGAGAAGCTCTCGTTGGAGGTCATTGATCATGCTAAAGGCCTTCCTTTAGCACTGAAAGTGTGGGGTTCTTTCTTACATAAGAGGGACATAATTGAGTGGAGAAGTGCTATAGAGCAAATGAAAATTAAATCCAAGTCAGAAATTGTTGAAAAACTTAAAATTAGCTACGATAGACTGGAGACCATACAACAAGACATATTTCTAGATATAGCATGCTTCTTACGAGGGGAGGGAAAAAAATACATCATGAAAATTCTTGAGAGTTGTTATTCTGGAGCAAATATTGAATTGAGTGTCCTAATTGACAAATCTCTTGTGTTCATCGACGACTATGATATGATTCAGATGCATGACTTAATACGAGATATGGGTAAATATATAGTGAATATGCAAAAGGATCCAGGTGAACGTAGCAGACTATGGGACATGAAGGATTTGGAAGAAGTGTTTGTCAACAATACAGTAAGTAGACTAAACAACACAATAATATTCAATTTCTCTTTTTATCAGGGGACAGTGGCAGTGGAAATAATCTGGAATATTTATATTCCAAAACTATGGTTAAGCAAAGAGGCAATGAAAAATATGAAAAGACTTAGGATATTACATATAACTGACTGTCACGGATGGTTCCAACGGAACCTCGATACAGATTATAGCTCCGGGAGTGATTCGGAAGACAATGTTTCCTATGATTCCAATTGCCATGATGGTTTCATTGAGTACCTGCCCAACAACTTGCGTTGGTTTACCTGGTATCGCTATCCTTGCAAGTCATTGCCAGAAAATTTTAAACCGCAAAGACTTGTTCATCTTCAACTCCAGGATAGTTTGTTGCATGAATTATGGACTAAAAGAAAGGTACAATTTTATTTGAAATACTTTTTATTTTTGCAGAATAACAAAACTCCCCTAAAATGTAGACATAAATTTGTGTTGTTTTCATTATGGTCTTTGTCATCATTTGTCCTCCCCACGAGTCTCGGGTTTGAGATCCACACCGCCACATGGCATTATATACTTTTGCCGTCTCTACGAAGGCTAGATCTCCGGTTCTCTAGAAGCTTGATGGCAACACCAGATTTCACCGGGATGCCAAATTTGGAGTATTTGGATTTGGGTGGATGCAAGAGTCTTAAAGAGGTTCACCATTCTCTGGGATGTTCCAGAAAACTCATCTCTTTAGAGTTGTATGGTTGTATAAACCTTGAGAGGTTTCCATGTGTAAACGTGGAATCTCTTGAAAAACTATATATAATGAGTTGCTGTAGTTTAGAGAAATTTCCAGAATTCCTTGGAAGACTGAAGTTGAAGTTAGACATTGGTGAGAGAGGAGGCTCCAAGATAAGTAGTGATTTGCCTACAGAGCTAGATTTGAGTAATTTCAAAAACCTTGTAGCTTTTCCAAGCAGCATTGGCATGTTGAAAAGCTTGGTGAAGTTATATCTATCGTATTGCTCTAAGTTTGAAAGCCTTTCAGAAGGGATAGGGGATTTAGAAAACTTGGAGGAGCTTCATGCCGACTATACTCTAATCTCACAACCTCCATCTTCCATTATCCGGCTGAACAAACTTAAATCATTGAGTTTTGCAAAAAAAAAAACAGGAGGAGGTCTCGTAGATGGAGTGTTCTTTGTTTTCCCTCACGTGAATGAAGGGTTACACTCATTGGAAAAGCTAGATCTCCGTTACTGCAATTTAACAGATGGAGGACTTCCGGAAGACATTGGATGTTTATCCTCATTGAAAGAGTTGCATCTCCAGGGAAATAATTTTGAGCATTTGCCTCTAAGCATAGCCCAACTTGGTTCTCTTCGATCCTTGGACTTATCAGAGTGCAGGAGGCTTAAAAAGTTTCTAGGTGTTAATGTGGCTGAAGGGTTACGCTCGTTGGAAAATCTGAATCTCAGTTCCTGCAATTTAAAAGAGGGAGTACTTCCCGAAGACATTGGATGCTTATCCTCTTTGAAAGAGTTGAATCTCCAGGGAAATAATTTTGAGTATTTGCCTCGAAGCATAGCCCAACTTGGTTCCCTTCGATCCTTGGATTTATCAGAGTGCAAGAGGCTTAAAGAGTTTCTAGGTGTTAATGTGGCTGAAGGGTTACGCTCGTTGGAAAATCTGAATCTCAGTTACTGCAATATAATAGATGGAGGACTTCCGGAAGACATTGGATGCTTATCCTCTTTGAAAGAGTTGAATCTCCAGGGAAATAATTTTGAGCATTTGCCTCGAAGCATAGCACAAGTTGGTGCTCTTCAAACCTTGCACTTATCAGATTGTCATATGGCTCTGAAAATTATTCCTAATTTACCACAGAGGGTGATATTCACGCCATTGCCATATGGCTCTGAAAAGTACAATGATTTGATTCCTATTACGTTTGCCCGTGCCATGTTTCAGAATATCTCTGATTCACATTCCTTGTCATTTAGAATGTCTACCATTAAGCATGCTGGGAATAAAATCCCTAGTTGGTTCCACCATCGGGGGACAGATAAAAGTGTATCAGTCTATTTGCCTGAAAATTGGTACACATGTGATAACTTCTTGGGATTTGTTGTATGTTATTATGACAGATTAGTTGAAACCACAGCTCACTTGATTCCCTTATGCAATCGGATGCCGTGGATGATCCAGAAGTTATCCGTAAGCAATCATTCAAAGTGCTATAAATCTTATCTTGATGAACGATCTACTATTCATTTATTCTTTGTACCTCTTGCTGGCGTATGGGATACATCTAAGGCAAATGGAAAAACACCAAATGACTATGGATTTATTGGTGTAACTTTTTCTGGACAAATTAAGAATTACGGATTTCATTTGTTGTATAAAGATGAACTAGAGCATGAGGCGTTGTTACAAATGAGGGGAGATAATGATGAACCAACAGAGCACTCCATTTGGACAAGGAGGAACAGATATGATGATAGTGAACACCATAATGAAGCCAGTTGCTCGTCTTCTAAGAAACAAAGGTCTGGTACTACTGTTGATGTGCCAGTTAATCCAGAAACTTTGCAGCTCTTTCCGACAAGTCCTGGATTTTCGCTTAAATAA

>PGSC0003DMG400013165

CAGAGTTTAGACTTGAGAGCAGAGAAAAGGTACTCAACTGGTAAGAAATTAAAAGAGATCATAGCTATGGATTTCCTATCTAAAGTTGGGTATCTCATTTACTACAAGAGAAACATCAAATCTCTGGATAAAGAATCTGATAAGCTGGAGAATATCAAAAGTGGGGTGCAGCAAAGAGCGGAGGCTGCACAGAGAAACTTACAAGTCATTGCTCCCAGTGTTGAGGCTTGGTTAACTAGTGTTGATACCACTATTGCAGATGTGGCTACTACATTGCGACGTAGAGCTGAGGTTGAAAGTGGTTGGTGTCCAAACTTGATGTCTTGTTACTTGCTGAGCAAGAAATCTAAGGAAATTGAACTGGATGTGATTGGTCTTCAAACTGAAGGCAACAATTATGTGGATTTTTCCTATCCTGCACCACCACCTGCTGTTGAAGATGAAATTGTACATGGTGAGGAGTTTGACTCCAGAAAACAGAAGGAGGAAGAGGTCATGGAAGCTTTGAGAGATGAGGGGGTCACTATTATTGGGATATGTGGTATGCCTGGTGTTGGTAAGACAACACTGGCTGAGAAAATCAGAAAAAGGTCAAAACAAGAAAGGTTGTTTGATGATATTGTCATGCTAACTGTCAGTCAACAACCAGACTTGAAAAATATTCAGGGTGAGATTGCGAGAAGCATCGGTCTGACGTTAGAAGGGGACGATTTGTGGCAGCGTGGAGATATGCTGTGTTCAAGGTTAATGGGACAGGAAAGTGTCCTTATAATCCTGGATGATATTTGGGAGGCTCTTCATGATCTAGAGAAACTTGGAATTCCCAGCGGTAGCAACCACAACCATCGATGCAAAGTGACATTCACAACGCGTCGCCGAGATGTTTGTCAAGCTATGGAAGCTCATAAGATCATGGAAATTGGGACCTTATCTAAAGAGGAAGCATGGATCCTTTTCAGGCAGAAATCTGGTAATTCAATTGATGATCCTTCTCTTCATGACATAGCAAAAGAGGTTGCCAAAGAATGCAAGGGGCTGCCGATTGCTATTGTTACAGTTGCAAGAGCACTAAAGTTTAACAGCAGGCCTTCATGGGAGGATGCCCTTATAGAATTACAAAGATCTGCACCAATTAATATCCCGGGAATGATTGAAAATGTGTATCAACCTCTGAAGGTATGCTATAATCACTTAGAAAGTGATGAAGCCAAGTACCTCTTTTTGCTTTGTTCCTTCTTCGAGGAAGATAGTGATATCGTGCCTGAAAAATTACTTAGAAAAGGAATGGGGCTTGGCATCTTTTCAGAAATTGAAAGTATTGAACATGCAAGAAATAGGATGTGTCTTCTGTTAGAAACTTTGAAAGATCGTTTCTTGTTATCTCAAGGTTCAAACAGAAATTATGTCAAAATGCCTGATGTGGTCCGTGACGTGGCTATATATATTGCTTCTGAGTGCAAGCATATTTTTATGGTAAGTCACTTCTGA

>PGSC0003DMG402006288

ATGGCACATAATGATATTGAGGATATGTTGGATCATCTAAAAAGGATCAAGAGTGGAGGTGATTCAAACAGTGTCAAGATTGATGAAATTGAGATACTTGAAGTGGAGCTAAGATTTTTGAGAACCTTTATCAAGTACAATCATGTTCTTTTGCCTGATTCCTTAGACATCATAAAAAATAAGTCCAAATTGATTGCGGAAATGATTCACTCTGTTTTTGGTGGAATTCCATATGGAAGCAAAACTAACTTTAATGTGGAAAGTCTAGTGTTGCAGTTGCTGGAATTCATTGAAGGTAATACTGGTTTTATAAGGTACAATAATGAATTGAATGATTCCTATCTTTCAGAATACATGGATTGCCTCGGCAATTTTCTTAATGATGTACTGAAGTACCTGGGATTGCATATGTCTTACCCTTATTTAGCAGATGAACATATCCTTAAGTTGAAGCGATCTTCAAAGCAAGTGCAAATCATTCAAAAGAAAATGAGAGGATTGCATATGTCTTACCCTTATTTAGTAGATGAACATATCCTTAAGTTGAAGCGATCTTCAAAGCAAGTGCAAATCATTCAAAAGAAAATGAGATTTTTGAGATACTTATATGTCACAGAGATAAAGAATTACGTTGACCATGAGAAGCTGGAAGGTCTGGAGACCCGAATTCAGTTCATAGCTGACAATGTGGGACAATTCTGTTATGATCTTTGGGTTTATAAGGATAAAAATGATACAGATTCAGATTCAAATGAAGTTGATATAGATGAGGATGAGGACAAAGATGATCTCTGGTATAAACCTCCTTATGTATTATCCCTGATTGTGCTAGTGGAGCTGCAAATAAAGAAGATTTTTCTCGGTGAACTAAAGACTTCAAAATTTACTCAAGCAAGAACTTTCAAGGACAAAACATTACCAAAAGGATTTTCACATCATCTCCACAGTATGCTGGTGAATCTCAGAAACAAAAAGCTTGAGAACTTCCCTAATACTGTCTCTGCTCAAAATATTGATGTGGCAATAGAGTTCTTGTCTTTTTTTCTTGTGGATGTGTCAAATAGTGTTTTTTATGGTAAAAAGTTGAATGAGGTCTTGGAAAAGGTTGGAGCTATAGCGGGTGATATTCTTTATGTAATTCAAAAGCTTCTTCCTAGCTCTATAAACAAAGATGACACTAGCAAAGTAGATGTCTGCACAATACATATATTGGAGAAAACTAAAGATCTAGTGGAGAGGTACTACAAATCCTTAAAATTCACTTCATCTCAGTTTCCCACGGTTTGTGGATTGAGCTTTCTGGATTCTCTTTTGCGGAAATTGAATAAGATATTGAAATCTGAATCAGGTTTAGATTTCATGATTAAAACTCGTAGTGGTATCATAGAGAAAGAGTTGTCGTTGCTTATATCCATTTTAGAGAAGGAGTTATCATCTCTATCATCTATAATCAGAGATGTCGCAAAGGTGCACCATGAACATGAAATTCTCAAAGATCTTCAGAGTCGTACTATCAATTTGGCATATGAAGCTGAAGTTGCCATTGACTCTGTTCTTGCTCAGTGTAATGCTTTTTGGCATATTTTTTGCTCTCTTCCTACAATTGTAAAAGAGATCAAACATATTAGAGCAGAGGTTTCCAAGATGTGGTCAGAGGACTTAGCTTATAAGTCATGCTATGTGGTAGATCCATCTAATCATCTGCCAACACAACGTAACAATCTTGTGAATGATGAGGAGATAATTGGCTTTGAAGATGAAGCAGAAAAAATAGTTCAATATCTGATTCGAGGTACAAATGAGTTAGATGTCATTCCGATTGTAGGTATGGGGGGACAAGGGAAAACAACCATAGCTAGAAAAGTGTACAAGAGTGACATGGTTGTTTTTCACTTTGATCGTCTAGCATGGTGTTGCATTTCCCAGACATATAATCAGATAGAGCTATTAAAACAGATCTACCATCAAGTTCCCAGTTACGAGGACAATGTCTGTAAGGACGATGAACTTGCTGACATGTTGAGGAAAAGATTAATGGGCAGGAGGTATCTTATTGTCTTGGATGATATGTGGAGTGTTAAGGCATGGGATGATTTAAGATTCTCTTTCCCAAATGATGAAAACAGAAGTAGAATAATTGTAACAACCCGACTTGAGAATGTGGCTAGGCAGATCAAGTACCATACTGAACCTCATTTCCTTCCATTCCTCACACCAGAAGAAAGCTGTGAATTATTATGGAAAAAAGTGTTCCAAAATGAAGGTCCCCGCCCTGAACTATACGATGTGAGTCTAGCAGTTGCAGAAAGGTGCAAGGGACTGCCTCTGGTGATTGTCTTGGTTGCTGGAATTATCAAAAGGAAGAAAACGGACGCCTCTTGGTGGCATGAGGTTAACAATTCTCTGCTTTCCTATCTTGGCGAGTCTGAAGAATATAGTTTTTCGACTATGCAGCTAAGTTATGATAACTTACCAGATTATTTAAGACCTTGTCTTCTTTACATGGGGATGTTCCAGGAGGATGCAAGAATTCCTGTGTCTAAATTGATACGTTTATGGATTGCTGAAGGCTTCGTGCAGAATATTGAATCTGGGAGATTGGAAGAGGCAGCAGAAGGTTACTTGATGGATCTCATTAGCAGTAATGTGGTAATGATTTCAAAGAGAAGATATAGCGGTAAAGTCATATACTGCCAGGTTCATGATATGGTGCTTCACTTTTGCTTGGAGAGGAGTAGAGAAGAAAAGTTTATGCTGGCGGTGAAGAGAAATCGTAGCCAATTTAAACCTTCTGATTGGAACGAAAATAGACTGAGCTTCAAATCCACTTGTGATATATCCGAGTATGACCAGCTGGGATACAAAACACAGAAGCCCTTCCATCAACATTTGAGGTCACTGATAATAATCAATGATTTACATTGGAATCCCTTCTCTCAGATTAGTAAATTGGGACTCCTTAAGGTCTTGGATTTGAGTTCCAATAGAGTGGATCATTTGTCGTTGGCTACATTGAATCCACTAATTCACCTTAAGTACCTCTCAGTTTCCATATTTGAATTCGATTTCCATCCAGAATCACATATGCGCCATCTAGAAACTATCATAGTGAATGGAAATGGTCGTCCTGTATTGTTGCCAGCAACATTTTGGAAAATGGAAAGGTTAAGGCATATTGAGTGTTATGCTTCTTTTGATTTGAAAAATAATAAGCAGTGGATCTTTAAAGAATCCTGTAAATTGGAAAATTTGAGGAAATTAAGTAAAGTTGAATTTCAAATTGATGATGCTGATTGCATGGATGTGTTGTTACAGAAATGTCCTAATCTTCAAGAACTTGGGATTAGTATTTTTAGCTATGAATGGGATTCTGCTGATATTTGTACTTCCGGTCCCAATTTGGAGATTCTTAGCCAGCTTCAATTACTTAACCTTTGCTTTCGTGGTGGCAACATTATTGTAACCGAATTACACCTGCCTTCAAATCTAAAGGAGTTGACACTTGAAGTGCCTCATCTATTAAACGTGGGATCCTTGATTGCGGGACTACCATGTCTGGAGTATCTCCAATTGATGGATTGGAGACGTGAGTCGGGAGAGTTGTGTTTCGGAGATATCACATTTAATAAGCTTAAGTTCTTGAAACTGGTGGAGTTAAATATCTCAAGGTGGGAAGCCTCAGAGGCATCTTTTCCCCAGCTTGAGACACTTGTTATAAAAAGGTGTTACCAGCTTGAGGAGATCCCCCTTAGCATTGCAGATATTCCAACACTGGAACAAATTAAGTTGATTGCATGCTATAATAAATCTCTGAAGGCTTCAGCTGTGAAAATTAAGGAAGAAGTTAGAGATATACAGGGAAGCGACCATCTTAATCTCATTATCGAAGTAAGTAGAAACAAATTCCTATGTGCTTTTTTTTGA

>PGSC0003DMG400006296

CTTCCTCACCGAGTGAGGCATTTGTCTTCAACAACTGCTCATTCTCAGCTCTCAACTTGTGCTAGAAAGTTGTACAACAGTGACATCATTGTTTCTCATTTTGATGTTCGGGCATGGTGCATCATCACCCAAACATATAACCGGAGGGAGTTATTACAAGAGATTTTTAGCCAAGTTACCGGTTCCAAGGACGAGGGAGATAAGGATGACATCCTTGCTGACATGTTGAGAAAAAGCCTAATGGGAAAGAGATATCTCATTGTATTAGATGATATGTGGGATGGTATGGCATGGGATGACTTAAGGCTTTCTTTTCCCGATGTTGGAAATAGAAGCAGAATAGTAGTAACAACTCGACTTGTGAAAGTTGGTGTGCATGTCATACACCATACTGATCTTTATTTCCTTCCATTCCTCACACCAGAAGAGGCTTGCAGATTGTTGTAGAAAAAAGTGTTTCAAAAGGAAGTTTGCCCATCTGAACTAGAAGATGTGAGTCTAGCAGTTGCAAAAAGATGCAAAGGACTGCCTCTAGTAGTTGTATTGGTAGCTGGAATAATCAAAAAAAGGAAAATGGAAGAATCTTGGTGGAATGAGATTGAAAATACTCTATTTTCCTATCTTAGTAAGTCTGAAGAATATAGTCTGGAGACTATGCTTTTAAGTTATGATAACTTACCCGATCATTTAAGACCGTGCCTTCTTTACATGGGGATGTTTCCGGAGGATGAAAGAATTCCAGTGTCTCAATTGATAAGTTTATGGATCGCAGAAGGTTTTGTGCAGAACATTCAATCTGGAATCTTGGAAGATGCAGCTGAGGGTTACTTGATGGATCTCATTAGCAGTAATGTGGTAATGGTTTCATGGAGAAGATATAACGGTAAAGTCAAATACTGTCAGGTTCATGACATAGTGCTTCACTTTTGCTTGGAGATGAGCAGTGAAGAAAAGTTTATGTTGGCATTGAAGGGAAATCATAGCCAGTTTCAACCTTGTGATTGGAAAGAAAGTCGAGTGAGCTTCAGTTTCAATAATGAGCTTTCCATGTTTGCATCTCTAGGCTCCAAAACACGAAAGCGTTTCCACGAGCACTTGAGGTCACTGATAACGACCAATCTAAGTGAATTTTATGATTGGAATCCCTTCCGTCAGGTTAGTGAAGTGAGACTTCTTAAGGTCTTGGATTTGAGTTCTCATACAGTGGATCATTTTGGGTTAGCTACATTGAAACCACTAATTCACCTGAAGTACCTCTCTGTTCGCACAATTACATTCAATTTTCATCGAGAACCGCACCTGCCCCATCTTGAAGCTTTAATTGTGAAGTGTTTCATCAAACCTACAGTGTTACCAAAGATTTTTTGGAAAATGGAAAAATTAAGGCATGTTGATATTAGTAAAGCTGTTATTGATTTGGAGGGGATCTTTGAAGAATCACCTAAGTTGGAAAACTTGAGGATATTAAGGAATGTTAGATTTCGAATTTGCAATGCTGATAGTGTGGATGTGTTATTATGGAGGTGTCCTAATCTTCAAGAACTCGAGATCTTTTTTAAGGGCGATAACAAAAGATATTTCAGTCCCACTTTGGAGAGTCTTACCCAGCTTCAAATACTTCGCCTTTATGTTAAGTGGCCCCGAATTGTATCCGAGTTACACTTGCCTTCAAATTTAAAGAAGTTGGTACTAACTGGAAATCCTACAGAAGGCATGATTTCCCTCATTGCGGGACTACCAAGTCTGGAGTATCTCAAATTAAGCAGGAAGTCTCAGATTTGGATGAAATTAGGAGAGTGGTGTCTTAGAGATATCACGTTCCATAAACTTAAATTCTTAAAACTGGTGTGGTTAAATATATCAAGGTTGGATGTCTCGGAGGAATCCTTTCCCCAGCTCGAAACACTTGTTATAAAAGGAAATGACGAGCTCAAGGAGGTCCCCCTTAGCTTTGCAGATGTTCCAACACTGAAACAGATTAAGTTGATTGACTGCAATGAATCACTGGAGGCTTCAGCTGTAAGAATTAAGGAAGAAGTCCAAGCAATTGAAGGATGTGACCGTCTTAAACTTATTATTATCAAAGTAAGTAGAAACAAACTCCTATGTGTTGTTTTTGAGTATCAACGTGCCTCTTATGTGCAAACAATATGTTCAATACAGGAAAGGTGGCAAAACATCCGGAAGTGA

>PGSC0003DMG400006769

ATGGCGGAAGCAGTACTTTCTGCTCTAATGGAAGTCCTCTTCCAAAAGACAGCTTCCCAAATTTTCCAAAAAAATGGACTACTAGGAAGTACAAAGAAAGAGATGTTGAATCTACAGAGCACATTTTCAACAATTCAAGCTGTTTTACAAGATGCTGAGGATAGGCAAATGAAAGAAAAAGCTTTGAAGAACTGGCTGGTGAAGCTCAAAGACATAGTCTATGAAGCAGATGATTTGTTGGATGAATACATGACTGAATTGCTTCGCCATAAGGTAATTCTTGATGACAGGGAAACTCGTTATTGTGTTTTCCATGCTGTTAATTCTCTTTATTTGAATGGTACATTAATTTTTTTGGGTTATAGTATGAAGTTGAAACTGAAACAAGTTGTGGAAAAGTTAGACCTTGTTGCGAATGAGAGAGCCAAATTTCATTTTAGGGATGCTGTTTATGAAAAAGGGTTTTCTTGTGAAAGACCACAGTCGGATTCTTATGTAATTGAATCGAAGATTTTAGGGAGAAATAAAGATAAAAAGAATATAATCAAGTTACTTATAGGATCAGATGAGTCAGTTGTGAGTATTATTGGAATTGGAGGAATTGGGAAGACAACAGTTGCGAAATTGGTTTACAATGATGCTGTTGTGGAGAATAGTTTTGATACAAGAATATGGGTTTGTGTCTCAGAAGGATTTAATGTGAAAAGACTTCTTAAAGCAATCATAGAATCTGGCACTGGTAGTAGTTGTAACCTTGTAGAGATGGATGTGATCCAGCGGCGTGTCCAGGAGTTGATATTGGGCAAGAAATTTTTGCTTGTCCTAGATGATGTATGGGATGATGATCATGAGAAATATGAGAGATTAAAGAACTTAGTTCACAATGGTTTGGATGGGAGTAAACTCCTAGTGACTACTCGCAATGAGAAGGTTGCATTACTGATGGGCACAACAAATCCATACCGTTTGGAAGGCCTTTCAGATGGTGATTGCTGGTCCTTGTTTCAGGAGCTTGCATATAAGAATAGGCAAAAAGAACTATTAGCTCTTGAGGAGGTTGGGAAAGAAATTGCAAAGAAATGTAGGGGAGTTCCTTTAGCGGCAAAGGCCCTTGGAAGTTTGATGTGCTTAAAGAATCAGAAATCTGAGTGGTCCTTCATCCGAGATTGTGCAATGTGGGATCTTATGGGACATGAAGATGGAGCTGGTATTCTTTCGGCCCTAAGATTAAGTTATGAATACTTGCCAACACATTTGAAACAATGTTTTGCATATTGTTCGATATTTCCCAAGGGATATCGGATAAATAAAAACACCTTGATACGCCTATGGATGGCGGAAGGATTTGTTCCGTCCTCTGAAAGCATACCACCGGAAGAAGTTGGGAATGGTTATTTCAATGAGTTGTTGTGGCGCTCCTTCTTCCAAAATGTGAGAAGAGATTTTGATGGGATTATAGTCGAGTGTGACATGCATGATCTTGTTCATGATCTTGCCAAATCTGTAGGCGGTGTTGATTGTTTAACAACAGAATTTGGCAAGGAGGTTATCATTCCTGTGGCGACACGCCACTTATCGATGTTTGGCAATGAGGTGGTACCTAAAAATCCCGGCATGTTGAAGAGTGCTCAAAATCTGCGATCATTTCTTCTATTGGATGGTCAGAGAAATATCACAAAACTTTCCAAGAGTTTCTTTTTGAGCTTTAGATCTATTCGGGCTTTAGATTGCAGCGGTACTCGCATAAAGAAGTTGTCTAATTCAATTGGTACTTTATTACATCTGCGGTACCTCAATCTTTCACATACTCTGCTGAGAACATTGCCTAAGTCTATCTGTTGCCTCCTTAATCTTGAAGCTTTAATACTTAAGCATTGCAACCATCTGATAGAACTTCCGGCAGAAATTAGAAAGTTGGTGAACCTTAGACATCTGGATATATACGGATGCACATCCTTAACCATGTTACCTGGTGGCATTGGACAGATGAGATCCCTTCAAACGTTGCCAGTTTACATTGTTAGCGATGCTGCTGCTAGTGACATCTCAGAATTGCAGAGACTTGATCTTCATGGTGAATTAATGATCAAGAACCTTGAGAATTTGTCCAATGAAATATGTGCCAAAAATGCAAACTTGAAAGGGAAGAGGCACATCCAATTTCTAAAATTAATATGGGCGCAAGTTGAAGAAATGGAGACAAGAGAAAATGTTGAGCGAGTTGTTGAAGGCCTTCAACCAAATTCTGATTTGAGGAAGTTACACTTAGAGGGTTACATTGGTGCAAATTTCCCCAGTTGGTTGATGACTACATACTTGGTAAACATTGTGGAACTCTCACTTCTCAAATGTCACAGGTGCGTGGAACTGCCTCAACTAGAGAAACTGCCTTTTCTTGAGGTTCTCACTGTTGATGGAATGGATTCTGCAATGTATTTCTGCGGTAGTTCTGGTGGAAAGGATTCGGCTACTCATTTTGCATCACTGAAGCAACTCACCCTCAGGAATATGCCCTGTCTACTGGGATGGTCAGTTAATGAAGATCATGGTATTCTTCCTCGTCTGAAGAAATTTACGTGTGAGGCATGTCCCTCTTTGAATAACTTGCCATATCTACCCTCTCTCAACTCATTAGAGTTGTCTGACTGTAGCAGTGAATTACTAGCTGAGACAGCAGCCAATGTTACCTCCCTTACTCATCTTATGATCAGTGGATTTTTGGAACTTATACACTTACCTGAAGGATTGCTGAAAAACAATATAAGTCTGCTGTCTGTAGAAATTAGAGATTGTCCAGAGATTCGAAGTCTTTCGAGTGAGCTGAAAGTTCTTCCTTGTATTGAGTCATTAAGCATCAGTAACTGTAAGAATCTCAGTTCTGTGTTCGATTCCTGTGGACTTGGGACTTTGAAGTCCTTGTCCATTCATGGTTGCCATAACATTAGCCTGGAAAAAGGATTACAAAATTTACAGTTTCTTCAATATGCATCACTCTCTGACTGTGGAAACTTGACAACCTTGCCGATGCCTATGCAACACCTTACGTCTCTCCAAACTCTGCATATATGGAGCTGTTCTGAGATGTATATGCTTCCAGAATGGCTAGGAGACCTCAGTTCCCTTAGAGAATTGGAGCTGTGGTACTGCAGAAAATTAAGTTCTCTACCAGAATCAGTGAAAAACCTAACCAAGCTTCAGTTTCTGTCTGTATGGGGCTGTCCAAATTTGGGATCAAGATGCAGAAATGATGTTGGAGAAGATTGGCACAAGATAAAACACGTTCCATTCATCAAGATTAATGGTCCATATATCCAAGCTATGACAGGTAATTAA

>PGSC0003DMG400006785

ATGACAGAAATCCTATACACTCTTGCAGCTGAAATCTTGATGAAGTTGGGTTCCCTAGCAGCCCATGAGCTTGGATCATTATGGGGCCTTAAAGATGATCTTTCCAAGCTCAGAGATACAGTTTCTGCCATGGAAGCAGTATTTCTTGACGTAGAGGAGAAGCAAGGGGATAGCCGTGAGGTCAAAAATTGGGTCCGGAAGCTCAGAGCTGTGTTTTTCGAGGCTGATGACTTGTTGGATGACTTCTACACAGAGATTACTCGGCGTAATTTGCTGATGCAGAAAAACCCATCAAAGAAAATTTCTATTTTCTTCTCGAAATCAAATCCTGTTCTGTATAGTCTTGAGATCCATCAGCGTCTCAAGGTTATTAGAAGGAAACTTGAAGCAATTGAGAAAGACAAGCATATGTTGCACCTTGTTGAAAAGCAAGTAGTTGATTTTGAGCTTCCAGATAGGGAGACTCATTCCTTTGTGAATGTTGACGAAGTAATCGGAAGGGAAGATGAGAAAAGGGTAACCGTCGATCGTCTACTAGATGTTCGAGAAAATGTTTGTGTGGTTCCTATAATTGGTATTGGGGGACTTGGCAAAACAACACTTGCACAACTTGTTTATAACGAGGACAGGGTTAAGAAACATTTTGATCTGAGAATTTGGGTGTTCGTTTCGCATATTTTTAATGTGAAACTCATTGTGGAAAAGATGATAGAATCTATCACTGGTATGAAACCTCAAAGCCTCCATTTTGACAGGTTGCAAGACCAACTTAGAAAGGAAATTGATGGTAAGAAGTACCTCCTTGTTTTGGATGATATGTGGAATGAAAATCGCGAAATTTGGTTGAAATTGCAGGATCTTTTGATTGGTGGTGCTAGAGGAAGTAAAGTATTGGTGACCACTCGATCAGGACTAGTTGCAGCAGCTATGGGTACTGCTCCACCATGTAATCTAAAAGGTCTGCCTGAAGACATGTCTTGGTCCCTGTTTAGTAAATTAGCATTTAAGCCCGGCGAAGAAATAAATTCATCTCTAGTGGCAATTGGAAAGGAAATACTGAGAAAATGTGCTGGTGTTCCTTTGGCAATAAGGATCTTAGGGAGCTTTTTGTATTATAAAGAGACTGAAGCTGAATGGTTGTATGTCAAAAATCATCAACTCACAGACATGGCGGAAAGTGCAGACATTGAAATTTTGCCAATTCTAAAGTTGAGTTATGATAATCTTCCTATACACTTGAAGCATTGTTTCAGTTATTGTTCAATATTCCAAAAGAATCAGACCATTAGCAAGAAAACCTTGATTCAGTTATGGATAGCCCAAGGTTTTATCAGATCAAATGATGAGGAAAATGAATGTCAAGAGGATGTTGGAGAACGTTACTTCATGGGTTTGCTGAGAAGGTCCTTTTTTCAGGATGTTAAAGAGCATAGGTTGGGCGATATCATCAGCTGCAAAATGCATGATCTGATTCATGACCTAGCAAAAATGGTTGCAGAGAATGAGACTTTGATGCTAACTTCTGCAGGAAATAAATCTAGTGTTAATATTTGCCATTTGTCAGTTGGTCCTGTTCACGATTCAGCATGGGAGCTCCCTCACTCCTTGCTCAAAGAAAAAAATTTACGCACGTTTTTAATGCCAATTGCATCAAGGGATTACCTGAAATCGGGAAGATTTATTGGGTCCGAGAAACAAAGTAAATCAGTTGTGGATGCAGTTATATCGAATTTCAGGAGCCTAAGAGTATTGGATCTACATGGCTTGGGAATCAATGAAGTGCCAGGTTCTGTGAGCATGTTGAAGCATCTAAGGTACATCGACCTATCTGAGAATAATTTTGTGACACTTCCAAAATCAATGAGCAAACTGCTGAATTTGCAAACTCTAAAGCTGTCTTATTGTTTTGACCTTTGTGAACTGCCAGAAAATATTCACAAAATGGTAAACATCCGACATCTAGAGCTTGATGGTTGTTTGAATTTGTCCAAAATGCCATGTGGGATTGGACAATTAACTGCTCTTAGGACTTTATCGCAGTTTGTGATAGGTCAAGAAACCTCTACGAGTTCTAAAGTCAATGCAGTGTTAACCGACTTGAATGGTCTTGTCAAGCTAAGAGGAAAATTAACACTTAGAAATTTGGGCTGCATAGAGTGTTTATGTCCTAAAATTAATGATGCTGTTTTGAAGAACAAGGAATACTTGCAATCACTGAGATTAGAATGGACATACGAAGCCGTCAATGATGAATATGATGAGCTGTTATTGGAAGGCCTTCAGCCACATGAAAATTTGAAGGTACTTTTCATAGAAAGATACGGAGGTCAAAGTTTCCCAAAATGGATGATGGTTGGCTTGCACTCATCACTACCAAAGTTAACAAAACTTACTCTGAAGAATCTGAAAGTATGCAAAAGTCTTCCACCATTTGGGTGTCTTCCCTCTCTCCAATCCCTTAAACTTGAAAATTTGACCTTGCTAGAGTACATTGAACATACTTCCTATGATGGATCTCAATTTGGAATGGAGCTACAAAAAGGATCTGCAATGTATTTTCCATCATTGAAAGAATTGAAACTCTGTAACTTGCCTTGCTTGAAAGGATGGTGGAAAAAAGAAGTAATGGCTGGTAGTGAATCATCGTCATTTCCTAACCGGCTCCTACTTTCATCATTTCCTTCACTCTCCAAGCTGACCATCCAAGATTGCCTTATGCTTGAGTTCATGCCAATTAATCCACGTCTCGAGGAATTGAACTTGATCAGAGTTGGCAATAAACTTTTGCAACAACTAATGATGCTTCTGGAAACAACATTGTCTGCAAATTGTTCATCTTTCCTTGCTGTCTCAAAATTGAAATCATTGTACATTCTTGATGTGAGAGAGTTATTCCTTTTACCAGAGGGATTGCAAAATTTGAGTCGGCTTGACCACTTGGAAATCAATGGTTGTCCTAACCTATTGAGTTTGCCAATAGAAGGAATGCGAGCGTTGAATATGCTCCGGTTTCTGCATGTTCATGACTGTGGGTTAACTTCTTTATTTCAAGCTATAAAGCATCTCTACGCTCTTGAAACACTAGTGATTAGCTCCTGCAAAGAGATGAATTTATCAACTGACAATGATCAGGAATATCTGCAATTTGAAGGCCTAAAAAGCCTCCATACAGTATACATCCAAGAGATACCGAAATTGGTTCATCTTCCAGTGTGGCTTCAGTATCTTCCTTCTTTGCGGGCGTTGCATATAGAGAAGTGTTATAGTTTACTGGACCTACCTGACTGGATCAGTGATCTAAAATCGCTTCATGCGCTTTTCATCTACAAGTGCCCAAAATTGACATCAATTCCTGCAGGAATGGTTCACCTTACTTCCCTTGAAGAACTCCGAATTCTCATGTGCCCTAATCTCTGGACATGCTGTCAAAACGAACAAGGAGAGGACTGGCCTAAAATAGCTCATATTCCAAAAGTTATTGTGAAGTGA

>PGSC0003DMG400006800

ATGGCTTATGCTTCTGTTGTTTCTCTTGCTCAAACTCTAGAGCAACTCAGTACCAGAATCCCAGGTTTGTTTTCTGACCCAAAAATTCAATCTTTATCTGCATCTCTTGATTATTTCAAAGCCTTTCTTGATGATAGTAGCAAAAGTAGTTATTATCACCAAGAAAATATCAAAGATTTGGTTGGAAAGTCTAGAGATGCTCTCAATGAAGCTGAGAATATTATTGAACTAAAGATTTGTGAGATTCATCAAAGAGGAAATGAAGATTTAGTTGGAAGTTTAGTACCAGTAGTACAGAAGATTGAGTTACTAAGGGAAGAGTTGGTGCTGAGTCTTGAGACTTGCACAAGCCATGACCATGACATTGAACCTACTGAAGATCATTTCGAGACGAGGCTTGGAAGTCCATCTAGACCTAGTTTTAATGCAAATTTGGAGAATAATGTTGTTGGTCTTGATGATGATTTGGAGAAGGTTATAGAAAGGCTACTTGGGTACTCATCCGAGAGAGAAGTCGTTGCAATTACAGGTATGGGTGGGATTGGCAAAACGACACTCGCTAAAAAAGCTTATGATTATCCTCGAGTTAGGTCTCGCTTTGATGTTCATGCATGGGTTACTGTATCTCGAGAGTATGGAATGAGGAGGTTGCTGTTAAGTCTAGTCCGTTGCATTCCAGGTATGACGACAGATAAGCTTGTAGAGAAAACTGAGGATCAGTTAGCGGAGTCATTATATAGAAAACTTAAGGATCGAAGATATCTTATTGTCATTGATGATATTTGGAGTACTAAAGTTTGGGACGATGTAACAAGATGTTTTCCAGATGATGACAATGGGAGTCGAATCATATTAACGAGTAGGCTCAAGGATGTGGCTGCTTATGCTAATCCTGATAGCCCTTTGCATGAGATGGGGGTCTTAAGTTTAGATGATAGTTGGAAATTACTCTCCATTAAGGTATTTGGGGTAAATGATCTTTGTCCTTCTGAGTTAGAGGATATCGGGAAGCAAATAGCGGAAAGATGTGGTGGACTTCCTTTGGCAATTCTGGTGGTTGCAGGGCATCTTTCTAAGATTTCTAGGAGAAGAGAAAGTTGGATCATTGTTGCCAAGACTGTTAATTCAGTTGTTGCGAACGATCCTGATAAATGCCTAGGAGTGCTCGGTATGAGTTACAATTACCTGTCGAACCACCTCAAGCCATGCTTCCTCTCTATGGGTGCTTTTCCGGAAGATTTTGAAATAAAAGCTCGGACATTGATCCAAGTTTGGGTAGCTGAAGGGTTTCTAAAAGCAGAAAGGGTCAAGAGCTTGGAAAAAGTTGCAGAAGAGTGTTTGGAGGATCTTATTAGTAGAAATCTAATAATGATTAGAAAGAGGAGGTTCAATGGTGAGATCAGAAGTTGTGGCATACATGATTTGTTGAGGGACTTGAGCTTAAGAGAAGCTCAGAAAGAGAAGTTCCTGCACGTGACATCAACTCGATATGTTTCAAACTTCCTAGCACAGAGGAATGAGGGTCGTGGCTTCAGTTTTCTTTCCAACATTTCTCTAAATGATTCTAGTGAGTTGTCATCTCATGTAACTCGATCGATGTTTTTCTGGGGTAAACTGTTAATCTCTGCTCCGCCTCATAGGCAAATTTCCTTATTCGCGAGCTTCAAACATATCAGAGTGTTGGCAATTTTTTCTCATATGTTTCCTGCATTTCCAGCTGAGATAACACAACTTACTCATCTAAGGTACCTTTGGATTCAATCAAATGGTGGTCTCCCCGCATCAGTGTCTCATCTTTATAATCTGCAAACCTTGGTATTTCAACAGCCGGAACTGTATTATATGCACAAAACTTTGGTTCTGCCTCGGGATATCTGGAACATGACACAGCTAAGGCGTCTGCGTCTACTGAGTGGCAATTATCTGTCTAAGCCTAAAAGAAGTACCACAACAGATGATGTTTTGGGGCTCAGCAATCTGGAGGAACTTTCTCACTTATGTTTTTCTAGCTGTACAGAGGAAGTCTTTTCATGCCTTCCCAATATACGAAAACTGAGTATCCTCGATGCTGCATCAGACGATGCATCTGAATACTTAAAAAATCTGGTACATTTAGAGAAACTTGAAACATTGAAATGCATCTGCTATGGCCAGAAGCGGCTCACCTTGTCAAACTGGTGCGCTTCCCTAACATCTGTTAAGAGGTTAGTATTATCCGGTTGTCTTCTGCTTTCTGAAGACATGGCAAGTCTAGCTGCATTGCCCAATCTTGAAGTGCTTAAACTTAGAGATAATGAGTTTGAAGGTTGTGCGTGGACACTAAGTGATGAAGACGAGTTTAGCCAGCTAAAGTTCCTACTGCTTTCTGAGCCACGTCTAGTGAACTGGGAAGCTGGTAGTGTTAACTTCCCAAACCTTCAAAAGTTAGTTTTGAGGAAATGCATCCGTCTTGAGGAAATACCTCTCGATATTGGAGAGATATGTACATTGGAGATGATTGAACTGATTTGCTGTAGCTCTTCTGCTCAGAATTCAGCAAATGAAATTCGCGAAGAACAAGAGAGCATGGGAAATTCTTGTCTTGACATACGCGTTTATGGTAAGTTTTCCCTTTAA

>PGSC0003DMG400025259

ATGGCAGACTGTGCTGTCGTTTTCTTACTTGACAAACTCACAAACTTATTAGCGGAAGAAGCAATATTGCTTCAGGGAGTCAAGCATGATATCCAGTATATCAAAGATGAATTAGAGAGAATGATTGCCTTTCTTGGTGTGGCTGATGCTTTCGAAGAGGGAGATGCTGAGGTCAAAGTCTGGGTTAGGCAAGTGAGAGATGTCGCCAATGACATTGAGGATGTTCTTGATGAGTCCATGCTCTTGTCGTATGATCATCACTACCGTGGATCTTGTTGTTTCATTGCTAAATTGGTTTTCTCAATCAGAAACATCAAATTCCGCCATAAACTTGTCATTGAAATCCAAGCCATCAAATCAAGAGTTGACAATATTGCAATGGGCCATCATAGGTACCGTTACAAATTTTATGTCCCGGAGCAAGGTTCATATTCTAACCATGCCTACGATACTGCAAATGATCGCAGAGGTGATGCATTATTGTTAGAAGAAGCTGAGCTTGTGGGCATTGAAAATCCCACACAGCAGCTAATCGGTTGGCTCGTGGAGGATGATCCTAGACTTAAAGTGGTTTCAGTGGTGGGGATGGGAGGTTCAGGAAAGACCACCCTGGTTAAGAAAGTGTACGAGGATGCAGCAGTCAAGAAAAACTTCAGTAGCCTTGCTTGGATAACAGTTTCCAAGTCCTTCAAGGTTGAGGAAGTTTTGAAAGATATGATTCAACAGCTCTATGATGAAGTGAAGCAGCCAGCACCTGAAGGTCTAAACACCATGAGTAGTAACAGGCTAAAAACAATAGCAAAAGTATTCCTCCAATCGAGAACGTATGTGCTAGTTTTTGATGATGTCTGGACCATTCAAGCTTGGGAAGCTATCAGACATGCATTGCCTGATGTAAATAATGGAAGTCGTGTTATCCTCACAACACGTCTTTTGGATGTGGCTTCCTTCTGTAGCATAGAGACCAATGGCTATGTTTATGAGGTGAAACCCTTGTCTACTGAAGAGTCATGGATTCTTTTCTGCCAAAAGGCATTTCATGGTTATTCATGTCCTTCACACTTGGAGAGCATTTCAAGGAACATCTTGAAAAAATGTGGTGGACTGCCGCTGGCTGTTGTGGCCGTTGGTGGGGTTTTGGCTACAAAGAACAGGAACAACATTAGGGAATGGGGAATGCTTAATCACAGCCTTGGTCCTGAGCTGGATAGCAATGACAAATTTGAGAGTATGAGAATAGTTTTGTTACTCAGTTTCAATGATCTCCCCTACTATCTTAAGCCATGTTTCCTGTATTTGAGTATCTATCCTGAGGATCATCTAATTGAGCGCAATACACTGATCTACCGGTGGATAACGGAAGGATTTGTAAAACAAAAAGTACGGAGGACAGTTGAAGACGTTGCAGACAGCTATCTCAATGAGCTCATCAACAGAAGCTTGATCCATCCAGTGCAGTACAATGATGACGGTAGCATGAAATTGGGTAGGATTCATGATTTATACCGCGAGCTTATTCTTTCAAAATCAAGAGATGATAACTTCACTGCAACAGTGGATGAGCATAATAAATTGTGGCCTGAAAAAACTCGATGGCTGTCAATGCATGGCATGTTGGGCAACCTACAAGTGAAAAGGTCAGTCACTAAACTTCGCTCTTTGCTTACATTTGGTGTAGCAGATCCACAGTCCTTGTCTTGCATAAGTCAAGTGCTTGGTAGCTCCCGGATGCTAAGAGTTTTAGATTTGAGAGGAGCTCCTCTAAATATGATTCCAGAGACAGTCTTCCAATTGTTTCACTTAAGGTATTTAAGCTTGAGGAATACCAATGTGAAAGTGCTTCCAAGATCCATTGGAAGACTCAAACAGCTAGAAATATTAGATCTGAAGCAGACATATGTCACTGAGTTGCCTGTGGAGATTTTAAAACTTGAAAATCTCCGTCACCTTTTAGTCTATAGCCACGTATCCTATTCGTATCTTCCTTACAATTGTTCACCAGGTTTTAAGGCTTTCCGAGGAATTGGAGCTTTGAGAGCCTTGCAGAAACTTGTGTATATTGAGGCAACCCCTGGCAGTGGCATACTTAAAGAAGTAGGAATGTTGGGTGAACTGAGACGACTTTGTATTCTGAAGTTGAGAAAAGAAGATGGTTGGACTGTGTGCTCATCAATTCAAAAGCTCCACAAGCTTGAGTCTTTAAATCTTAAATCAGTGGAAGAGCATGAGATCCTTGATCTTTCTTATCTGTCGTCTCCTCCGCCTCTTCTTCAGCGCCTGTATTTGACAGGACACATTGTTATGTTGCCTGCGTGGATTCAAGATCTTAATTCCTTGGTCAAAATTTACTTCAGGTGGACTCATTTAACAGAAGATCCACTGAAATATCTACAAGATTTGCCCAATCTTGTCCATCTTGAATTTCTTGTAGGATACACAGGAAAGGAACTCTACTTTGAGCAAGGGAAATTTCAAAGGCTTAAGCTTTTAAATTTGGACAAGCTGGAAGGACTAAGGCAGGTGACAATCGGCGAAGGTGCAGTACCTCATCTTGAGAAGCTGGTTATCCAGAGATGTGCTTTGCTTGAAACTGTGCCAACTGGAATCGAGTACCTCCTAAATCTGAAGGTTCTTGAATTTTTTGACATGCCTGATGAATTTATCATGACATTGCGTCCAGAAAAACTAGGTGCTGATGCTTGGAAAGTTTCACATATCCCTGAAGTATTCTATACATATTGGAGGGACGGTTGTTGGATGGTACATTCACTAAAGGAAAAGGAGAAAAATCAGATTTCTGATCAGTCTGGAGCTGTCACTAGGACCTATGGAAGGCGAAATTCCCTCTAG

>PGSC0003DMG400006316

ATGGCAGACTGTGCTGTCGTTTTTGTACTTGACAAACTCACAAACTTCATAGCTGAAGAAGCAATATTGCTTCAGGGAGTGAAGCATGATATCCAGTATATCAAAGATGAATTGGAGAGAATGATAACCTTTCTTGGTGTGGCTGATGCTTTCGAAGAGGGAGATGCTGAGGTCAAAGTCTGGGTTAGGCAAGTGAGAGATGTCGCCAATGACATTGAGGATGTTCTTGATGAGTCCATGCTCTTGTCATATGATCATCACTACCGTGGATCTTGTTGTTTAATTGCTAAATTGGTTTTCTCAATCAGAAACATCAAATTCTGCCATAAACTTGTCGCTGAAATCCAAGCCATCAAATCAAGAGTTGACAATATTGCAATGGGACATCATAGGTACCGTTACAAATATTATGTCCCGGAGCAAGGTTCAAATTCTAGCCATGCATACGATACTGCAAATGATCGCAGAGGTGATGCTTTATTGTTAGAAGAAGCTGAGCTTGTGGGCATTGAAAATCCCACACAGCAGCTAATGGGTTGGCTCGTGGAGGATGATCCTAGACTTAAAGTGATTTCGGTGGTGGGAATGGGAGGTTCAGGAAAGACCACCCTGGTTAAGAAAGTGTATGAGGATGCAGCCGTCAAGAAAAACTTCAATAGCCTTGCTTGGATAACAGTTTCCAAGTCCTTCAAGGTTGAAGAAGTTTTGAAAGACATGATTCAACAGCTCTACGATGAAGTGAAGCAGCCAGCACCTGAAGGTCTAAACACCATGAGCAGTAACAGGCTTAAAACAATAGCAAAAGTATTCCTGCAATCGAGAACGTATGTGCTAGTTTTTGATGATGTCTGGACCATTCAAGCTTGGGAAGCTATCAGATATGCATTGCCTGATGTAAATAATGGAAGTCGTGTTATCCTCACAACACGTCTTTTGGATGTGGCTTCCTTTTGTAGCATAGAGACCAATGGCTATGGAGGTGAAACCCTTTTGTCTACTGAAGAGTCGTGGATTCTTTTCTGCAAAAAGGCATTTCATGGTTATTCATGTCCTTCACACTTGGAGAGCATTTCAAGGAACATCTTGAAAAAATGTGGTGGACTGCCGCTGGCTGTTGTGGCCGTTGGTGGGGTTTTGGCTACAAAGAACAGGAACAACATTAGGGAATGGGGAATGCTTAATCACAGCCTTGGTCCTGAGCTGGATAGCAATGACAAATTTGAGAGTATGAGAATAGTTTTGTTACTCAGTTTCAATGATCTCCCCTACTATCTTAAGCCATGTTTCCTGTATTTGAGCATTTATCCCGAGGATCATCTAATTGAGCGCAATACACTGATTTACCGGTGGATAATGGAAGGATTTGTAAAACAAAAAGAGCGGGGGACAGTTGAACACGTTGCAGACGGCTATCTCAATGAGCTCATCAACAGAAGCTTGATCCATCCAGTGCAGTACAATGATGACGGTAGCATGAAATTGGGTAGGATTCATGATTTATACCGCGAGCTTATACTTTCAAAATCAAGAGATGGTAACTTTACTGCAACAGTTGATGAGCATAATAAATTGTGGCCTGAAAAAACTCGATGGCTGTCAATGCATGGCATGTTGGGCAACCTACAAGTGAAAAGGTCAGTCACTAAACTTCGCTCTTTGCTTACATTTGGTGTAGCAGATCCACAGTCCTTGTCTTGCATAAGTCAAGTGCTCAGTAGCTCCCGGATGCTAAGAGTTTTAGATTTGAGAGGAGCTCCTCTAAATATGATCCCAGAGACGATTTTCCAAATGTTTCACTTAAGGTATCTAAGCTTGAGGAATACCAATGTGAAAGTGCTTCCAAGATCCATTGGAAGACTCAAACAGCTAGAAATATTGGATCTGAAGCAGACATATGTCAGTGAGTTGCCTGTGGAGATTTTAAAACTTGAAAATCTCAGTCACCTTTTAGTCTATAGCCTCGTATCCTATTCGTATCTTCCTTACAATTCTTCACCAGGTTTTAAGGCTTTCCGAGGAATTGGAGCTTTGAGAGCCTTGCAGAAACTTGAGTGTATTGAGGCAACCCCTGGCAGTGGCATACTTAGAGAAGTAGGGTTGTTGAGTGAACTGAGACGACTTTGTATTCTGAAGTTGAGAAAAGAAGATGGGAGGACTGTGTGCTCATCAATTCAAAAGCTTCGCAAGCTTGAGTCTTTAAATCTTAAATCAGTGGAAGAGCATGAGATCCTTGATCTTTCTTACATGTCATCTCCTCCGCCTCTTCTTCAGCGCCTGTATTTGACAGGACACATTGTTAAGTTGCCTGCGTGGATTCAAGATCTTCATTCCTTGGTCAAAATTTACTTCAGGTGGACTCATTTAACAAAAGATCCACTGAAATATCTACAAGATTTGCCCAATCTTGTCCATCTTGAATTTCTTGTACGATACACAGGAAAGGAACTCTACTTTGAGCAAGGGAAATTTCAAAGGCTTAAGCTTTTAAATTTGGACAAGTTGGAAGGACTAAAGCAGGTGACAATCGGGGAAGGTGCAGTACCTCATCTTGAGAAGCTGGTTATCCAGAGATGTGCTTTGCTTGAAACTGTGCCAACTGGAATCGAGTGCCTCCTAAATCTGAAGGTTCTTGAATTCTTTGACATGCCTGATGAATTTATCATGACATTGCGTCCAGACAAACAAGGTGCTGATGCTTGGAAAGTTTCACATATCCGTGAAGTATTCTATACATATTGGAGGGACGGTTGTTGGATGGTACATTCACTAAAGGAAAAGGAGAAAAATCAGATTTCTGATCAGTCTGGAGCTGTCACTAGGACCTATGGAAGGCAAAATTCACTCTAGTCGCTT

>PGSC0003DMG401026043

ATGGTTGATGTGGTGGTTGATGTGGTGGTGGAAATTCTGTTAGAGAACTTGAAGAAATTGGTGATGGAGAATGTGGAGTTAATCGGAGGAATTAAAAATGAAATCGAGAATCTGTGTGATGATTTGAGTGAATTCAATGCCTTTCTCAAGCAAGCTGCAATGGTCCGCAGCGAAAACCCAGTTCTGAAAGAATTAGTGAGGAGTATCAGGAAAGTGGTGAATCGTACTGAAGATGCTATTGATAAGTTTGTAATAGAAGCTAAGGTTCATAAGGACAAAGTGTTTAAAGGGGTTTTCAACATACCTGTACATTATAAAAGAGTGAGGGACGTCGCTGTGGAGATTAAAGATATCAGAGATAAAATGAGAGAAATTCGGCAAAATAAGGCACATGGCCTTCAGGCACTTCAAGATCATGATGATTCAATCAGCAGAGGTGGAGAAGAGAGACAGCCTCCTGTGGTTGAGGAAGATGATGTGGTGGGCTTTGACGATGAGGCGCAGATGGTAATCGACCGTCTTCTTAAAGGATCAGGTGATTTAGAGGTTATTACAGTTGTTGGAATGCCTGGTCTTGGCAAAACTACACTAGCCACTAAGATCTTCAAGCATCCAAAGATTGAGTACGAGTTCTTTACTCGACTTTGGCTTTATGTTTCCCAATCATACAAGACAAGAGAATTATATCTTAACATCATCAGTAAGTTAACCGGAAACACCAAGCATTGCCGTCATATGTCTGAAAGGGATTTAGCTCTTAAAGTAAGAGAGATTTTGGAAGAAGGAGGAAAATACTTGATTGTCTTAGATGATGTCTGGTCGACAGATGCTTGGGATCGTATCAAGATTGCTTTCCCGAAAAATGACAAGGACAATAGAGTATTGTTGACTACTCGAGATCACAATGTCGCAAGATATTGCAATAGGAGTCCACATGATTTAAAATTTCTGACTGATGAAGAAAGTTGGATTTTACTGGAGAAAAGAGCTTTACACAAAGCTAAATGTCCCCCCGAATTGGAAACAAATGGAAAAAGCATAGCCAGGAAGTGTAAAGGACTACCCCTTGCTATTGTGGTGATTGCAGGAGCTCTAATTGGGAAAGGCAAGACAATACAGGAATGGGAGCAAGTGGATCAGAGTGTGGGCGAACATTTTATAAATAGAGATCAGCCAAATAAATTGGTGCGGATGAGTTATGATGTTTTGCCTTATGACTGGAAAACTTGCTTTATATATTTTGGTATATTCCCCAGAGGCTATTTAATCCCTGCCAGGAAATTGATCCGCTTATGGATCGCGGAAGGGTTCATCCAGTACAGAGGGAATTTATCCCTTGAGTGTAAAGCAGAGAATTACTTGAATGAACTCGTGAATAGAAATTTAGTGATGGTAATGCAAAGGACGCTTGATGGACAAATCAAAACTTGTCGTGTTCATGACATGTTGTATGAGTTTTGCTGGCAAGAGGCCACGACAGAGGAAAATCTTTTCCATGAAGTAAAATTCGATGGTGAGCAATCTGTTCATGAAGTATCCACTCACCGTCGCTTGTGCATTCATTCCTCTGTTGTGGAATTCATTTCTAAGAAGCCCTCTGGTGAGCATGTTAGGTCATTCCTATGTTTTTCTCCAGAAAAAATTGACACTCCTCCAACTGTCAGTGCAAACATATCAAAAGCCTTTCCATTGCTAAGAGTGTTTGATACTGAATCCATCAAAATCAATCGCTTTTGCAAGGAGTTCTTTCAATTGTATCATCTGAGGTATATTGCTTTCTCACTTGACTCGATTAAATTCCTTCCGAAACATGTTGGGGAACTTTGGAACGTACAAACCCTCATTGTCAACACACAACAGATCAACTTTGATATTCAAGCAGACATATTGAACATGCCCCGGCTAAGGCATCTGCACACCAACACGTCTGCTAAATTGCCTACGCCTGCTAACCCCAAAACAAGTAAGACTACCTTGGTAAATCAAAGCCTGCAAACCCTCTCCACAATTGCACCAGAAAGCTGCACTGAGTATGTTCTCTCGAGAGCTCCGAACTTGAAAAAACTGGGCATTCGTGGAAAAATAGCTAAGCTAATGGAACCAAGTCGGTCCATATTGTTCAACAATGTTAAGAGGCTGCAATTTCTTGAGAACTTGAAGCTGATAAATGTTGGTCAGACTGATCAGACACAATTACGCCTTCCTCCAGCATCTCTATTTCCAACTAAGTTGAGGAGGCTGACTTTATCAGACACCTGGTTGGAGTGGGATGATATGTCTGTATTGAAACAGTTGGAGTACCTTCAAGTCTTGAAGCTGAAAGACAATGCATTTAAAGGAGAGCACTGGGAACTAAATGATGGAGGTTTTCCTTTCCTAGAAGTGTTATGCATTGAAAGGGCAAACTTAGTTTCTTGGAATGCTTCCGGTGATCACTTCCCGAGACTTAAACATCTTCACATATCATGTGATAAACTTGAGAAGATCCCCATTGGCCTGGCCGATATACGCAGCCTCCAAGTGATGGATTTGCAAAATTCCACCAAATCAGCAGCAAAATCTGCCCGAGAGATACAAGCCAAAAAAAACAAGCTGCAAACTGCTAAATCCCAGAAGTTCGAGCTTTCTGTATTCCCTCCTGATTCTGATGTACAGACAGCTTCTTAG

>PGSC0003DMG404026043

ATGGTTGATGTGGGGGTGGAATTTCTGTTAGAGAACTTGAAGCAATTGGTGCTGGACAATGTGGAGTTAATCGGAGGAGTTAAAGATGAAATCGAGAATCTGCATGATGATTTGGAGAAATTCAAGGCCTTTCTTAAGGAAGCTGCAATGGTCCGCAGCGAAAACTTAGTTCTGAAAGAATTAGTGAAGAGTATCAAAAAAGTGGTGAATCATGCTGAAGATGCTATTGATAAATTTGTAATCGAAGCTAAGAATCATAAGGATAAAGGGTTTAAAGCGGTTTTTCATAAACCTGCACATTATAAAAGAGTGAGGGAGGCCGCTGTGGAGATTAAAGGTATAAGAGATAGAATGAGAGAAATTCAGCAAAATAACGCACATGACCTTCAGGCACTTCAAGTTCATGATGATTCACTCAACAGTGGGGAAGAGAGACAGGTATTGCTTCAAGTCCTTGATTTCGACAAAATTCGCTCAAAGCCTCCTGTGGTTGAGGAAGATGATGTGGTGGGCTTTGACGATGAGGCGCAGACGGTAATTGATCGTCTTCTTGAAGGATCAGGTGATTTAGAGGTCATTCCAGTTGTTGGAATGCCTGGTCTTGGCAAAACTACACTAGCCACTAAGATCTTCAAGCATCCGAAGATTGAGTATGAGTTCTTTACTCGACTTTGGCTTTATGTTTCCCAATCATACAAGACAAGAGAATTATATCTTAACATCATCAGTAAATTCACCGGAAACACCAAGAATTGCCGTGATATGTCTGAAACGGATTTAGCTCATAAGGTACGAGAGATTTTGGATGAAGGAGGAAAATACTTGATTGTCTTGGATGATGTCTGGTCGACAGATGCTTGGGATCGTATCAAGATTGCTTTCCCAAAAAATGACAAGGGCAATAGAGTATTGTTGACTACTCGAGATCACAAAGTCGCAAAACATTGCAATAGGAGTCCACATGATTTAAAATTTCTGACTGATGAAGAAAGTTGGATTTTACTGGAGAAAAGAGCTTTTCACAAAGCTAAATGTCCCCCCGAATTGGAAACAAACGGAAAAAGCATAGCCAGGAAGTGTAAAGGACTACCCCTAGCTATTGTGGTGATTGCAGGAGCTCTAATTGGGAAAGGCAAGACAATAAAGGAATGGGAGCAAGTGGATCAGAGTGTGGGCGAATATTTCATAAATAGAGATCAGCCAAATAATTGTAATAAACTGGTGCGGATGAGTTATGATGTTTTGCCTTATGACTGGAAAGCTTGCTTTTTATACTTTGGCACATTCCCCAGAGGCTATTTAATCCCTGCCAGGAAATTGATCCGCTTATGGATCGCGGAAGGGTTCATCCAGTACGAAGGGAACTTATCCCTTGAGTGTAAAGCAGAGGAATACTTGAATGAACTCGTAAACAGAAATTTAGTGATGGTAATGCAAAGGACGCTTGACCGACAGATCAAAACTTGTCGTGTTCATGACATGTTGTATGAGTTTTGCTGGCAAGAGGCCACGACAGAGGAAAATCTTTTCCATGAAGTAAAATTCGGTGGTGAGCAATCTGTTTGTGATGTATCCACCCACCGTCGCTTGTGCATTCATTCCTCTGTTGTGGAATTCATTTCTATGAAGCCCTCTGGTGAGCATGTTAGGTCATTCCTATGTTTTTCTCCAGAAAAAAATGACACTCCCCCAACTTTCAGTGCAAACATATCAAAAGCCTTTCCATTGCTAAGGGTGTTTGATACTGAATCCATCAAAATAAATCGCTTTTGCAAGGAGTTCTTTCAATTGTATCATCTGAGGTATATTGCTTTCTCATTTGACTTGATTAAAGTCCTTCCGAAAGATATTGGGAAACTTTGGAACGTACAAACCCTCATTGTCAACACACAACAGATCAACCTTGATATTCAAGCAGACATATTGAACATGCCCCGGCTGAGGCATCTGCACACCAACACGTCTGCTAAATTGCCTACACCTGCTAACCCCAGAACTAGTAAGACTACCTTGGTAAATCAAAGCCTGCAAACCCTCTCCACAATTGCACCAGAAAGCTGCACTGAATATGTTCTCTCGAGGGCTCCAAAGTTGAAAAAACTGGGCATTCGTGGAAAAATAGCTAAGCTACTGGAACCAAGTCTGCCCGTATTGTTCAACAATGTTAAGATGCTGCAATGTCTTGAGAACTTGAAGCTGATAAATGTTGGTCAGACTGATCAGACACAATTACGCCTTCCTCCAGCATCTCTATTTCCAACTAAGTTGAGGAGGCTGACTTTATCAGACACCTGGTTGGAGTGGGATGATATGTCTGTATTGAAACAGTTGGAGTACCTTCTAGTCTTGAAGCTGAAAGACAATGCATTTAAGGGAGAGCACTGGGAACTAAATGATGGAGGTTTTCCTTTCCTAGAAGTGTTATGCATTGAAAGGGCAAACTTAGTTTCTTGGAATGCTTCCGGTGATCACTTCCCGAGACTTAAACATCTTCACATATCATGTGATAAACTTGAGAAGATCCCCATTGGCCTGGCCGATATACGCAGCCTCCAAGTGATGGATTTGCAAAATTCCACCAAATCAGCAGCAAAATCTGCCCGAGAGATACAAGCCAAAAAAAACAAGCTGCAAACTGCTAAATCCCAGAAGTTCGAGCTTTCTGTATTCCCTCCTGATTCTGATGTACAGACAGCTTCTTAG

>PGSC0003DMG400002799

ATGGCTAGTGAATCCAAGTCTCAAGTGTTGTTGAGTTTCAAAGGCAATACCTTCGCAGATCATCTCTACGAAGCTCTAGCTGGAGCAGGTTTTGTAACATTAAGAGGTGGCGATGGAAACGAGGGAGGAGAAGAAATCAAATTGAAATTACGAAAGGGTGTTGAAGAATCAGGGATTTCAATTATAATCTTTTCAAATGATTATGTGTCTTCAAGTTTGTGTCTTGATGAGTTGGTAATGATCTTGAATTGTAGTAAAAGGAGATCAGTTCTGCCAATATTTTACCATGTGGATCCTTCTGATGTTAGGAAACAAAAGGGGAGAATTGGAGAAGCATTTGATAGGTATGAAGAAGCTAAGGTTAGAAAATGGAAGGAAGCACTCAAACAAGTTGCAGATTTGGGAGGAATGGTCTTACAGAACCAAGCTGATGGGCATGAGTCAAAATTCATACAGAAGATCCTTAAAGTGGTTGAGAATAAACTGAGCAGGCCAGTCCTGTATATTTGCCCTCATCTGATTGGAATAGAACGGCGTGTTGAAAAGATCAACTCATGGCTAGAGGATGGATCTACTGATGTTGACACTCTTGTTATCTGTGGCATTGGTGGAATAGGCAAGACAACAATGGCAAAGTATGTGTATAATTTGAACTATAGTAAGTTTGATGGTAGCAGCTTTTTGTCCAACATTAGAGAAAATTCAACACACCATAAAGGTTTTGTTACTCTTCAAAGGCAATTTCTTTCTGATATTTGCAAGAGAAAGAAGAAACCTATGTTTTCTGTGGACGAGGGAATGACTGAGATGAGAGATGCTGTAAGTTGTAAAAGAATCCTTCTTGTTCTTGATGATGTTGATAGTCGTGACCAATTGGATGCTCTACTGGAAATGAAGGACTTGTTATACCCTGGAAGTAAAGTCATAGTGACAACTAGGAACAAGAGATTGCTTAGGCCTTTTGATGTGCATAAGCTTTACGAGTTTGAAGCTTTGAATAGAGATGAATCAGTTGAGCTCTTAAGTTGGCATGCATTTGGTCAAGATTGTCCTATTAAAGGTTTTGAAGTGTGTTCAGAACAAGTAGCAATCCATTGTGGAGGACTTCCATTAGCACTTGAAGTTCTTGGTGCTACTTTGGCAGGAAGAAACATAGATATTTGGAGAAGTACAATACAGAAATTAGAAACAATTCCGAATCATCAAATTCTCAAGAAATTAGCAATAAGTTATGAATCTCTGGAGGATGATCATGATAAAAATTTGTTTCTCCACCTAGCTTGCTTTTTCATTGGGAAGGACAGAGATTTAGTAATAGCTATTCTCAATAGGTGCAACTTTTACACTGTAATTGGAATAGAGAATCTCGTTGACAGAAATTTTGTAAAAATTAGTGAGTCTAACAGGTTGATTATGCATCAAATGATTCGAGATATGGGAAGAGACATTGTTCGCCAAGAATCACCAATGGAGCCTGGGAAACGCTCTAGACTATGGCGCTCCAAGGATTCCTATAATGTCTTAATCCAGAACCTTGTAAGGAATTCTGTCTCATATGGCTTTTGTCTAACTTTGTGTTCATTTCTATCTTTTGTCATTCAGTTCCTTAAATATCTGAAGATTTTCGATCTCAGCCATTCCTACGAGCTTCTAAGAACACCTGATTTCTCTGGACTACCCAACCTTGAGAAATTGATCCTTCGATATTGTACAAGCTTAATTGAGCTTCATGAGACCATTGGATGTCTCGAATCACTTGTTCTGTTGAACCTCAAAAATTGCAAAAATCTGCAGAGACTTCCAGATAGCATTTGCATGCTAAAATGTCTGGTCACACTAAATATCTCTGGTTGCTCAAGTCTTGAATATGTACCGATGGATCTAGATAAAGTAGATTCACTGAGAGAGCTCTATGCTGATGAAATTGCAGTTCATCAAATGGTTTCTACTGCAGAAGAGGTCCAACCGTGGTATGGATTCCTGCGGTCCTGGATGTGCAAGGGGACAATATGTCCTAAAGTTTCACACATTAGTTTACCCAATTCTTTGGTTACTTTGAGTCTTGCTAAATGTAATCTATCCGATGATACTTTTCCAGTTGCTTTCAATAGCCTCTCGTTATTGCAAAACTTAGATTTGAGCCAAAATAAAATTTGCAGTCTACCAAAGGGCATAAGTTATCTTACTAGACTTCAGAAGCTAGAAGTGGAAGGCTGTGAAAAGCTCAAATCTCTCATAGGGCTTCCCAATATAGAACATCTCAATGTTACTAACTGCAGCTTGTTAGAGAAAATATCATATCAATCTAAATCATCTAGTCTGAAGAATTTGCTGGTCTCAAATTGTGTTGAATTAGTGGAAATAGATGGAAATTTCAAGTTAGAGCCCTTGAGAAATACTGAGGCAGAGATGCTTTCCAAGCTAGGCTTGTGGAACTTGGCCCCTATGAACAATGTCATGATCAATCTTACATCTAATATACTGAGCTACTACCGAATACACGGTAAAGGGTGGACTCCAACAAGGAAGACAAAGAAAGTTGTTCTTCAGGTATGTCCTGCTAACTATACCTGCAAATATCTACTTGCAATAGAATTGAAGACAGGAGCATATAAAGTTTTATGA

>PGSC0003DMG402018257

ATGGCAACCCCACAAACCCGTTATTCTTTCCACGTTTTCTTGAGTTTCAGAGGCGAAGACACCCGAAAGAACTTTACTGATCATCTCTACACAGCTCTCATCAACGCTGGAATTCGAACTTTTCGAGATGATGACGAAATTCGCAGGGGAGAAAACATAGAATCTGAACTGCAAAAGGGTATTCGAGAATCCAAGATTTCACTAGTTGTCTTCTCCGAAGATTACGGTTCTTCAAGATGGTGTCTTGATGAGCTTGTCAACATTCTTAATCGAAGAAAGAAAGAGGGTCATACTGTTTTACCTGTCTTTTACACTGTGAGCCCTGAGGATGTCCAGAACCAAACTGGAAGTTTTGCTAAAGCTTTTGTGAATCATGAAAAGAGAGGAAACGCAGAGAGTGGAGAGAAAAGAAAGGAATGGATGGAGAAGATGGAGAAATGGAGAGTAGCTCTGAAGGAAGTTGCTGAATTAGAAGGAATGTGCTTAGCCAAAGAAGTAGACGGGTCAGTTTCCCTGAATTTTCATCTTGCTTTGTCTCTAACATTTGCACTGCATCCCAGGCATGAGGCAAAATTCATCCAAAAGATCATCAAGGAGATACTCAAAAGATTGAATCGCACAGTACTTAGTGTACCTTCATACACAGTTGGATTAGAGTCTCGAGTGAAGGACATTAACTCATGGTTACAGGATGAATCCAGTGAAGTTGGCATTGGGATGATCTGTGGACTTGGTGGCGTTGGAAAGACTACTGTTGCTAAAGTAGCTTACAACTCTAATTATGACAGATTTGATGGGAGTTGCTTTCTTGCAAATGTCAGAGAAATCTCTGAGAAACATCCAAATGGTCAGGTTTATTTGCAAAAACAAATTTTTGAAAGTATTTTGAAAGGCAGAAAGGACAAAATATACAATGCTGATGAAGGAATTGTCAAAATGAAAGATGCTATTGGCAACAAAAAGGTTTTTATTGTATTTGATGATGTGGATCAGCTGGATGTCTTGGATTCACTTATTGGGACAAGGGATTGGTTTTATCCTGGGAGTAAAATTCTCATAACGACAAGGTGTGAGAAACTGTTGAAGTCCTATGAGAGGCACATGCTATTCAAGATAAAGGAACTTGGGGTTGATGAGTCTCTAAAGCTCTTCAGTTGGTATGCTTTTGCACAAGACTACCCATTAGAAGAGTTCAAGATGCTTTCAACAGAAGCAATACAGCATTGCGGTGGGCTTCCGTTAGCTCTTTATGATTTGGGTTCGTTTCTTTCAGGGAGAGATATGGAAATATGGAGGAGTAAATTGCAGAAATTGGAAGCAATCCCTCATAGCAGAGTTCAAAAGAATCTTGAGATAAGCTACAAGTCTCTAGATGATCATGACCAGAGGTTGTTCCTTCTTATTGCTTCATTATTTGTTGGGAAGGACAAAGATTATGTTATCAAATTTTTAGAGAACAGCGATTTGCACCCAACAGTTGGAATTCAGAACCTCATTGACAGATCTCTCATCAGCATTGATGACGAAAACAAAGTGATGATGCCTCCACTTATTCAGGACATGGGAAAAGAAATTATTCGTCGAGAATCACCAGATGATCCCGAGAGATTGTGTAAATTATTGAACCAATGTGGTCTATCAAATTGCTTGACAGAAAACAATGTAAGAATCTTGACTTTTCTATTCATGTTAAGCTTGTCTTGGTAG

>PGSC0003DMG400018256

ATGTCTCAGTTTGCTTTTCATGCATTTCTGAGTTTGGCAACAAAAACAGGCAAGTCTTTTGGAAATCATCTTCATTCAGCTTTATCAAATGCTGGTATTCGCGCATTCAGTGTTGATGAACTTGATATAGATGAAAAAGGGTGCAAAGAATTGCAGAAAACAATTCAAGAATCAAGAATTCTCATTGTTGTTTTGTCAAAAGACTATACCTCTTCAGAGAGGTGCCTTGATGAACTTGTGTTTATACTTGAGAGCAAGAAACTTTTTGGACGTTTCGTTTTACCTGTGTTTTATGATGTGGATCCATCAGAAGTCAGGAAACAAAAAGGAAGTTTTGAACAAGATTTTTTAATGTATGAACAGAGATATAGATCCGGGACCGAGGAAAGGAGACTGGAATGGTTGCAGAAGGTGAAGGAATGGAAGGCTTCTCTAACAGAAGTTGCTGATTTGGGAGGAATGGTCTTACAGAATCAATCTGATGGGCTGAAAATGGAACTCATTGTAATTTTCAGGTGTGAGTCAAGGTTTATTCAGGAGATTGTGAAGGTGGTTGCAGGAAGACTAAATCGTGCAGTTTTGAGTGTAGCTCTCCACCCCGTTGGAATAGATTCTCGAGTTAAAGACATAAATCTCTGGTTGCAGGATGGATCAACTAGTGTTGATATTATGGCTATATATGGTATGGGGGGGCTAGGTAAATCTACCCTTGCCAAGACTGCTTATAATCTTAACTTTGACAAATTTGATGGCAGCAGCTTTCTTGCTGATGTGAATAAAACTTCAGAAAGATATGATGGTCTGGTAAGTCTACAAAGACAACTTCTTTCAAATGTTCTAGGGAAGAAGGTTGAAAAGATATACAATGTTGATGAAGGAGTCATCAAGATTCAAGAGGCCATCCATTGCAGAAGAATTCTTCTTGTTCTCGATGATGTGGATGATCGAGATCAGCTAAATGCGGTTCTTGGGATGCGAGAATGGTTTTATCCAGGTAGTAAAATAATCATAACAACCAGAAATCAGCACCTGTTTGATGCTAGTGAGGTCTGCAGTTGTAAGATGTATAAGGTCATGCCATTGAATGCTCAAGAATCGATTCGACTCTTCAGCTGGCATGCTTTTGGAAAAGAAAAACCTTCCGAAGATCATGAGGACCTTTCAGAAAAGGTGATACTTCATTGTAAAGGAACTCCTTTGGCTCTCAAAGTTTTAGGTTCTTCTCTTTGTGACAGAAGTATAGAGGTGTGGGAGAGTGCATTAAGGAAGTTAAAGGCAATCCCTGACAATAAGATCTTGGAAAAACTTAGAATCAGCTACGATTTGCTGCCAGATGATGATGATGTACAGAAAATATTCCTTGATATTGTCTGTTTCTTTGTGGGAAAGGATAGAGACTATGCAGTTACTATACTCGATGGATGTGGTTTTTTCTCAGTAGTAGGAATTCAGATTCTTTCAGATAGATGTCTGATAGAAATGGACAAGGATAAGCTGAAAATGCATTCATTGATTCAAGATATGGGAAGAGAAATTATCCGCCTAGAATCCCCTTGGGAGCCTCAGAAAAGAAGCAGAGTTTGGCGATACAGAGATTCCTTCAATATATTGAGTACAAAAACTGTAAGAAATCTTACTCTTTGGTATTGTATATGCATTATGATTGACTGTAATATAAGACTTAACTTTACCTCTTACTTCATGTTGCAGATTCTTAGATCATTAAAGATCCTCAACCTTAGTCATTCCCATTTTCTAAAAAGAACTCCTGATTTTTCTGGACTTCCCCATCTGAAAACATTGATCCTGAAAGACTGCATTAAGTTGGTCAAGATTCATGAATCAATTGGATGCCTTGACGGACTTGTCTATCTGAACTTGAGAGACTGCAAGAATCTAAGGAAACTCCCTGGAAGCCTTTGTAAGCTCAAATCCTTAGAAAAACTTATTATCTCTGGCTGTTCTAGACTAGTTACGTCAGCAATAGAGCTAGGCAAGTTAGAATCATTGACAACCCTGCAGGCTGATGGAATGAATTTTGGACAGCTAGCCCCGGTGGGTGGTAACATGAATTCATGGAGTGCCCTCTGGAAAACTTGGTCATCAAAGTTGAGAAGATCCCCAGGCTCTAACCAGTTTTCGTTCTCTTCTTTATCAAGTTCGCTCGTCAGCTTAAGTCTTTCTAGTTGCAACTTAACAGACGATGCTCTATCCTTCGGCCTCAGCAACCTTCCCTCATTATGCTTCTTGAATCTAAGTGAAAACTTGATTTATAACCTGCCTCAGAGTATCAAAAATCTTGGTAAGCTCCAAGACCTTTGGTTAGATGGATGTCAAAGCCTCCTATCTCTGCCAGAGCTTCCGTCAAGCCTTGTTAAGCTAAAGGCAGTTAGATGCTCATCACTGGAAACAGTCACAAATCTACCAAACCTAATGACAACTCTCTTTTTAGATGTCATGGAAAGTGAGAGCTTAACTGAGATTTCAGGAATTTTCAAGCTAAAGCCCATTGATAATTTTGAAGTGGAAATATTGAATGCTCTACGCCTTCTTCTCAACCTAGATAACACATTCGATACAGTGGTGGAAATATTTAATAGGTTCACCAAAACAAAAAGGATGTATTCAGTACAGCAGGTAAGTTTTGTTTCTTACTGTAAACTTTCTCCTCAACCTGTTGAGCAAATCCAATGTTAAGTACCTTTTCTTTTTCCTTTTTGGCATCAGGGACTCTATGAATTTGGAATCTTCAGTACTTATTTTCCAGGAAATGAGGTTCCAAGCTGGTTCAGCAACAAAAGTGAACAGAGATTGTTGACATTGTATGTGGATTCACTTCCTAATATCAAGATAACAGGACTGCTTATTTGCATCGTCTATGCACGTTCTAGTCCTCGTATATTTAGCTATTTTAGTAAATTCGTGGGATCTCATACAATTGACATCAAAGTCCAAAACATAACACAGGGTCTTAAGTGGATTTATGCCCCATCATTCATTGGCATTCCAGGAGAGAACAACAAGTTGACATTTTTATGCCACTGGAAGTTTGGCAAGTATTTACAAACAGGTGATCAGATTAATGTTTCATTGCCTTGTTGGGGTAAAACTTTTAAGATGAAAGAGTTTGGAGCTACACTTGCTTATAACAATCCAGATTTAGACCAGTCCTCAGCATCTACTAGTGAAACAAGAGTGCTTGCAACGCGACATACTCCACTTAGTGATTATCAATCTGAAGAAAGTGTCATGGAAGGCTTCATGCCTTTATATCAGTTGGCGGTTCATCATTACTATCTTTCTCACCCTGAGTACTATGTCATGCGTGACAATGCAGATTTTGTTGTGAGGGCAATCTTGAACGAGAAATTGTTCGAGGATTATGATGTGCAGACTGCAGGTTCAGGTATGTGCTTCTTATATATCTTGCATATCAAAAACTTTATCATGACATATACTCATTTGTGTACTATTGGATGA

>PGSC0003DMG400018216

ATGGGTTCTGAAAGACCACAAACATCTTCCTCTTTTGTTTATCCATCCATTTATCACGTGTTCTTGAGTTTCAGGGGTGAAGACACCCGCAAGACTTTTACTGACACTCTTTATGCAGCTTTGGTAGGTGCAGGGTGGCGTACATTTAAGGATGATAATGAGACTGAGAGAGGGGAAAATATTAAGACAGAACTAGAGAATGCAATTATAAATTCGAGGAGTTCAATAATTATCATCTCGAAGAACTATGCTACTTCAACATGGTGCCTTGATGAACTTGTCAAAATCCTTGAACATAAGAGGACAAAAGGGCATGCAGTTCTTCCTGTCTTCTATCATGTGGATCCTTCTGAAGTTAGAGATCAGAAAAAGAGTTTTGCAGAAGCATTTGCGAGTTATGAGAGGCAAATTAAAGCTGAAAGTGATGAAGGAAAGAGGGAGTTGATAGATAAGGTCCGAAAATGGAGAGCAGCTCTTGGAGAGGTGGCAGATTCGGGTGGTGTTCTAGTGAACAATCAAGAGTATAAGTTGATTAGGAAGGAATCAGAGTTCATTGAAGAAATTCTTCAACTCATTGAGGATAAGCTGAACCGGACAATATCGAGTGTTGCACCTTACCTAGTTGGGATCAGTTCACAGGTCGAAAATATCATTTCATGGTTGCAAGACGGATCACATGATGATAATGTTATTGCAATTTGTGGGATGAGTGGAATTGGGAAGACAACTGTTGCCAAATATGTTTTCACTACAAACTGTAGAAGATTTGAAGGTAGTAGTTTTCTTGAAAATATCCAAGATATTTCTCAGCAACCTGATGGCTTAATTCGTTTGCAAAAGCAGCTCCTTTATGATTTAACTGGGAAAAAGAGTAAAATACAAGATACTGATGAAGGAATCATCAAGATAAGAGATGCTATATGTTCTAGGAGAGTTCTAGTCATATTGGATGACATTGATCAACAAGAACAAATTCACGCTATAATTGGAATGAAAAATTGGTTCTGTCCAGGAAGTAAAATAATCATAACAACTAAGAATTCATGTTTGCTGAAGGTTCAAGAAATTCAAAAAGTCCATAAAGTCAGGGAAATGGGTAATGATGAATCGCTGGAGCTTTTCAGTTGGCATTCCTTTGGGGAGGACCACCCAGCTGATGACTACATGGAACTATCAAAAAGGGTGGTAAAGCATTGTGGAGGGCTTCCTCTGGCACTTCAAGTATTGGGTTCTTCTCTTCGTGGGAAAAATATTGATGTATGGAAGAGTGCATTAGACAAGTTGGAAACAATTCCTGCAAGCCAGATCATTAAAAAACTAAAATTTGGGTATGACTCATTAAAAGATGATCATGATAAGAATTTGTTCCTTGACATTGCATGCTTCTTCGCAAGAAAGGATAAAGATTATGTTATTGCTGTGCTAGAGGAATCTTATATTTATACAAGAATTGGGATTCAGAATCTCATAGATAGATTCCTTTTGATGATTGAGGGTAACAAGTTGATAATGCATCAAATGCTTCGGGACATGGGCAGAGAAATAGTCCGCCAAGAATCACCTAAGAAGCCTGGGAGGCGTACCCGGTTGTGGCACTACAAGGATTCATTTAATGTATTGAGAGAAAACGTGGGCTCAGATACTATTGAAGGTCTTTTCTTTGACATGAATATGGTAAAGGAAGACCAATCCTTCATGGGAAGTTCTAGCAGTGGAAGAAAGTGGCTGTTTACAGAAGTTAAGAGTTATCGGTTCGGTTTCTCCAGGCATCCCAACAAATTTTCTTCAAAAACCTTAAATGAGCTTGAATTGGGAACGAATCTCTTTACAATTATGAATAAGCTAAGACTGCTTCAGATAAATTATACCCATCTAAATGGTGCTTATAAAGATTTTCCCAAGAACCTGAGGTGGCTATATTGGAGAGGTTTTCCTTTGAAGTGTGTGCCAAATGATTTTCCATTGGAGAGCTTATCTGTTCTTGACATGCGGAACAGCTGTTTGGAAAGACTGTGGGAAGGAAGAAGAGTATGGCAATTGTTTTTGTGCTATGTGCTTCCACTGGTAAAAATTCTCAACCTCAGCCACTCTCATTCTCTTTTCAGAACTCCTGATTTTTCAGGACTCCCAATGCTGGAGAAGCTTGCTCTCAAAGAGTGTGTCAATCTAATTGAGGTTCATGAATCAATTGGAACCTTAGATGCTCGGCTCATTTTCTTAAATATCAAAAACTGCAAAAGACTTCAGAAGCTACCCAGAGAGATTTGCAAGTTAAAAGTTCTTAAAACCTTCATAATATCTGGTTGCTCAAATCTTGTAGAGTTGCCAAGAGACTTGTGGAGGATGCAATCCTTGGAAGTATTCCTTGCCAATGAAATCCCAATGAGTCAATTACCCTCTAAGAGAAAACAGAACCCAATATGGCATGCACTTATCCGGTCTTGGGTTCCGAAGCCAAAAAAAGTTCTTGAGCTTTCATGGGTTTGTTTACCTAAGTCATTGGTTAAATTAAGTCTATCTGAGTGCAACCTATCTGAAGTTGCTTTTCCGAGGGACTTCAGTAATTTAATGTCGTTGCAGAACCTAGACCTCAGTAAAAATCCAATCAGTTGTCTTCCAGATTGCATCAGAACCCTTTCCAGGCTCAACAATCTTGAATTAGGTTCTTGTACAATGCTTAAATTTCTCATAGACCTACCTCGCATCCACAATTTGAGTGTGGGAGACTGCACTTCCCTAGAAAGAGTTACATATCTATCTGTAGGGTGCCGTGCAAAAGTTTACCATATAAATGGCTGTAAAGAACTCACTGATATGGAGGGAAGCTATAAGTTGGAATCCATGGGAGGAGTTGAGAAAACAATGAAGTCGTTGGAATTAAGCATGTGGGATTCTGTCGGAAGCTTTGAAGTGAAATTGTATAACAATTCAACTCATACAGAAAGTAGAGGACCAGTAAAGGTATTTTCTCCTCTTCCATTCCCCCTTCCCACCCCAAAGAAGGGGTTGACGGATCTACTGTCATTTGTGTGGTCAAACTTCTTCATGTAA

>PGSC0003DMG400018213

AACTCAAAGTTGATTGACTTTGGTGGTAAATTTTGTTCAATGACCACCACCAAACAATTAGCTTATGATGATCAATCATTCAATAATTCCAAGGGTTCATATGATGTGTTTTTGAGTTTCAAATGTGAAGACACAGGCAAAACTTTCACGGACCATCTCTCCACAGCTTTAAAACAAGCCGGATTTTGCACGTTCAAAGGTGGTGGTTGTGATGATATTGATGAATCAAGAAGAGGAGAAAACATTAATTCAGAGTCAATACAAGAATCAAAGATGTGTATAATTGTATTCTCACAAAACTATGCATCTTCTGGTTGGTGTTTGGATCAACTTGTTTCGATTCTTGAACGTAAAATGAAGTTTGCATGTATGATATTGCCCATATTTTACCATGTTGATCCTTCCAATCTAAGGAAGCTCAAGGGAAGTATTGGGGAAGCATTAGATAGACATGAAGAAAAATTCAAGTGTACAAGAAATGAAAAAGAGTATTGGGAGGACAAGTTGAAGAAATGGAAAAATGCACTTAGCCAAGTTGCTGATTTGGCTGGAATGGTCCTTCAAAATCAGTACTCTCTCTTCCATCTTTGCCAAATGCTAAAAAGAGGAAAACAGAAAAAATATTTATATGGATATTTCAGGCATGAATCCACTTTCATCAAGAAGATTATTAACGTTATCAGCACAAGATTAAGTCGTCCAGCCTTATATATTGCTTCTTGTTCAATCGGAATACATCGTAGAGCGAGGCCCATTAATTCTTGGGTTCAAGCTGATGTATCGAACAATTCGAATATTGAAATACTTCTAGTTTGTGGTATTGGAAAAACAACAATTGCCAAAATTGTTTACAATTTAAACTTTGGATATTTTGAAATTAGTTGTTTCTTAGCAAACATAAGAGAAACTTCAAAACTCCCTAATGGTCTCATCACTTTACAAAAGCAACTTCTTTCTATCCTTCTAAAGAATGAAAAGGTAAAAATATCAAGTGTTGATGAAGGAATCATCAAGATAAGAAATGCTTTATGTAATCGAAAAGTTCTTCTTGTTCTTGATGATGTTGATGAACCTGATTTGGTTGAAGCTATATTTGACATGAAAGATTGGTTTGGTTTTGGAAGTAAAATAATTGTGACAACTAGGCACAAAAGCTTACTAAGACTTCAATTAGGCCATGAGGTACATGAAGTTGGAATTTTGTACACAATTGAAGCAAATGAGCTCTTCAATTTTCATGCATTTGGTCATGAAAACATTAATCAAATAAGTGAAGATAAATGCTACAAAGAATACTCAAAGGAAGTGATTGAATGGTGTGGAGGACTTCCATTAGCTCTTCAAGTTATTGGTTCTTCCTTGGCTGGAAAATCTAAGGATGTGTGGAGAAGTGCAACAGAGAAGTTGAGAGATATACCTACAAATAAAATTGTTGACAAATTGAGATTAAGTTATGAGTTGTTGGAAGATGATCATGATCAGAATTTGTTCCTTCACCTTTCTTGTTTCTTTGTGTGGATGAAAAAAGATTTTGTAGTTAGGATATTAGATAAATGTGAGTTTTACACATTGGTGGGATTACAAAATCTCATTGATAGAAGTAGTTTAGTGACAATTGATGATTATGTAAATGATATAAGAATGCATCAATTAGTACGTGCTATGGGAAGAGACATCGTTCGTAGAGAAGCACCTACTGATCCTGGAAAGCATAGTAGACTTTGGCACCACGCAAATTCTTACAATGTCCTAAGAGGAAAAACAGTAAGTACTAAGCATGTGTTTATTGAATATTTAATTTTGTCACCTTATTCCCAAATTGGTGTTTCACTAGGTATGTGCTACCTCTCATGCATCAACAACACAAGTGTATCGGATGATTCTTCCCATCAAACAAAAAGAAATCACCTAGCACTTTGGTTGTATGTGGAAGTGGAACTTATATTTCTTTTTGAGCTCATCACAACACCAGATTTGTCGGGGCTTCCCAATCTTGAAATGTTGATACTTGAACATTGTACCAAGTTAATCAATGTTGACGACACCATTGGATGTTTGCAAACACTCATCCTTTTGAACCTTAAAGATAGTCAGAAACTAAAGAGCCTCCCAGATAGCATATGTAAGTTAAAATGTCTCGTGATACTTGACATCTCAGGCTGCCCAAATATTGAATACCTACCAACAAGAGCTGGAAAATTGACATCACTAAAGGAGCTTTATGCTGATGGGATCAATTTGGGGGCCCAAACATGGTATTCATCTTTATGGTCTTGGGCCTGGAAACAAAGGGTATCCCTTATAGTTCATGAAATCCATTTTCCAAGGTCCTTGCATGTGTTAGACATTGCAAATTGCAATTTATCTCTTGATGCTTTTAAGAATGTTGATTTAGGCATTGTGAGTTTGCTAACTTGTCTAAATTTGGAAGGAAATCCAATTAGCAATCTGCCAGATAGCATTAAGAATCTTACGAGGCTTAAATATCTTGGCATTTCATATTGTACAAAGATCAAACGTCTCGATTGATTACCATCAAATCTCAGTGAGTTAAATGCTAGTGGATGCATATCCTTGGAGAAAGTAGCTTCTAATTGTGCGAAAGGGTATCCAGTAGAAGGTTATATAAATTGCAACAAACTTATTGAGGTGGAAGGTGTGTTCAAGTTAGAGCCACTGGAAAATGCAGGTGCTCAACTGCTTGCCTTCATGGGAATTTCAAATTTACAACCTATGACTAGTATGATGGTTTCTCTTTTATTTGGTCGTGTGAGTAATATTGTTGGGAAAAAATATCCAAGCTTTGTACGCGATGACGTAGCAAGCTTATTTTCACTATCACCTAACAAGCTCCCCCCACAGGTCTGTCTCTTTTTACATGATTTATCACATGCACATTTTGTTGAATATAATACAAATCTAATTAATTTAGTGTACATGATGTCTCAGATATTGTATCACTGTGGTGTTTTCTCAACATTTTTGCCTGGCGAAAGCGTACCCAATTGGTTCCGCTACAAGTTCACAGATGCAGCAGATGTCTATTGCACCCTACCA

>PGSC0003DMG400013098

ATGAGAGTTGAAAAGGCATCAGTCTCCAGGCCTAGATGCTCTTATCATGTCTACTTGAGTTATAGAGGCAAAGAGATCAGCGTGAACTTTATCAATATCCTACACACTGCATTGACTGATGTTGGTATTCAAACATTCAAGAGACACAGTGAAGCTAGAAAAGGAAAGATTGTTGGATCAGAATTGCAAAAGGCAGTCAAAGAATCGAGGATTTCAATAATTGTATTCACGGAAGATTATGGATATTCTAGAAGGTGCCTAGATGAACTTGTGAACATCCTTGAAAGGAAGCAAAGTGCTGGTCATATGATTCTACCTGTGTTCTATCGTGTAGATCCATCCCATATTAGAAAGCAGAGAGAGTCGTATGCGAAAGCATTTCATAATTACGAAGAGCAGATCATGGCGAAGAAGGTTGAAAGAAGGAATGAGCACATAGCAAAGATAAAGATATGGAGATCATCTCTCACAGAGGTTGCAAATATGGCAGGGATAGTTCTAGAAGACGGGAATGTTTTTGTATATTTCTTATATTCTGTTGTTTTCCAACATTTGTCTCATCAGGGTTGTTTATGGACATTCAACTTCCATCTTGTTCAGTTGCGACATAATTTGCATTATAACCAGTGCTATCTATCTAAGTGGTCCTTTTCTTCTTGCAGGGATGAGCTGAAGTTTATTCAAGAAATTGTTGAGGACATTTGGGGTAAGCTGTCTCGTAAAGTTTTGAGTTTCGCCCCATATCCAGTTGGAATATACTCCCGTGTAAAAGAGATTAATTTCTGGTTACAAGATAGCTCGACTAATTCTCGCACATTGATGATTTATGGAGAACCTGGAATAGGGAAAACAACCATTGCCAAAGCTCTTTTTAACCTACATTGTGACAGATTTCAATGCAGCAGATTTCTTGGAGATATTAGAGAAATTGCAAAAGAAAGCTGTGGTCTGATTAACCTACAGAAAAATCTTCTTTCTGATCTTTTGAAAGAGGATAAGATTGATCTAAATGATATTGATAGAGGAGCTTCCACGATCAAAGAATGTTTAGTCCACAAAAAGTTTCTTCTTGTTCTTGATGATGTTGATGATTTGAACCAACTGAAAGGACTTCTTGATTCAAGAGAGTGGATTCCTCCAGGGAGTAAAGTTATTATAACAACTACAAACGAACACTTGCTAAACCCTCTTGATGCTTGTGTGATGTATGAAACCAAGAGGATGAACAATTATGAGGCGCTTAAGCTCTTCAGCTTGCATACTTTTGGTCAAGACCATCCCGTCAAGGAGTACATGAAACACTCCAAGCAGATAGTTAAACATTGTCGAGGGCTTCCTTTAGCTCTTCAGGTTCTAGCCTCTTCGCTACGTGGTGGAAGTATAGATATGTGGGAAGGTGCAATACAGAAATTAGAAAGATATTCTGAATGCCATAACCATAAAGTTCTTGAACTAAGCTATGAAGCTTTGCCAGATGATCATGATAAAAATGTATTTCTTGATATTGCTTGCTTTTTTGTCGGGAAGGACAAAGATTATACAATCAAAGTTCTGGATGAATGTGGTTTCCATGCTACTGCTGAAATACAGAATCTCATTGATAGATATCTCCTGACAGTGACTCCTGATAACAAGCTAATGATGCATCAGTTACTACAGGAAATGGGTAAAGAGGTCATCTGCCGAGAATCACCCATTGAACCTGGTAAACGCAGCAGAATCTGGCATCACAAGGATGCCCTTAGTATACTGCATGAAGAAACTGTAAGGCTCATTCCTCCTGTACCAATTTCGTTATTTTTCCTGGAGCTCATTTTCACTTCAACGTTACTTTCCATGTCAGGTTACAGAATCTATATTGAAGGGCTTGTTCTTAAAATGTGTGGATCAGATGAAAGCAAACCAGACAGACATGAAAGTATATCAAAAAGACCTTACTTCAATGATTCACAGAGCACTTCAACATTGACTCTCAAGAAAAATTCATCAAAGAGGCTTCGCGTAGGATGCCTCTCTTGGGGTCTGGGGAATTCTGTTTTGGCAAGATCACAGACTGTACAAAATGAAGCTGGAATGAGTGCTAAAGCATTTTCTAAAATGCAAGAATTGAGATATCTTGAGCTTGAGAATGTCCAGCTTTCTGGCACCTTTGAAGGATTTCCAAAGAAATTAAGATGGATGCGCTGGTATGGACGATTCCAATCAACATCCTTCCCAAATGGCTTTCCTCATGAAAATCTTGTAGTTCTTGAAATGAACAATAGCAACTTGCATCAAACCTGGGAGGGAGCAAAGGTTTGCTTACTTCCACTCGTTTCTGATTTTGTGGCTGATTGTGTTCTGGCAGACTTGATATTTTTCTTCACTGACTCTCTCTTTACCTATTTTTGCTCAGTCTCTACGATCATTGAAGATACTGATCTTGGTCACTCACACAGTCTCATGAAGACCCCTGATTTCTCTGGAATGCCGAATCTAGAAAGAGTGATTCTTGAAGATTGCATAAATTTGGTTAAGGTCCATGAGTCCATTGGAAGACTCCATAAACTTCTTGTCTTAAATCTGAAGGGATGCGAGAGCCTTAAGAAGCTTCCAAGGAAAATTTGGGAAATCAAATCCTTACAAGAACTAACACTCTGTGGGTGCTCAAAGCTGGAGCTTTCCAGGATTATGAGAAATGGTAAGTTTTTGCAAGCACTTACACGGGATGTGACTAACAGAGATCAATTTTCCTCTAAGGCTGAAAAGCCAATATGCAGCGACTCTCTGTCTGCTAAATCAGTCTATTCAATCTTCTGGTCTTGGATGTCACTGTGGCCAAAGTCAGCAGGTTCAATGTCAGATATCTTCCAGATCACCTTACAAAGTTTGGATATTTCACATAGCAATCTGACCGACACTCTTATTCCCTACGACCTTTCTGTCCTATCCTCCTTGAAATATCTGAGTCTAAGAGGAAATCCCATTTCCACCCTGCCGGAGAGCCTGAAGAGCCTAACTATGCTGCAGTCCCTTCAGTTAGCCGACTGCACAAGGCTCCAGTGGATCCCTGAGCTTCCGTTGAGCTTACAAATATTGAATGCAAGTAACTGCAGATCACTGAACAAAGTAACAAATTTACCCAATTTCATGAGGTCACTTGACTTGCACTTAGAGAATTGTGAAAAACTGGTTGAGGTTCAGGGAGTATTCAAGCTAGATCCTATTAGAGACATTGATGATATCCTCGATATGTCTTGCCTAGATAATTTGGAGGTCAGAGCAGTAGATGTGGAACTTTGCAACTATCTGTCCTCAACAAAAAGCAAAGGTCCCCTTCAGGGACTATACGAGTTTGGTATACACAACATTTACATTCCTGGAGTCAAGGTTCCTACCGAGTTCAGCAATATAAGCACAGGGAGCTCAATAGATTTCACTGTGCCTCCACTTCCTAATGCTAAGATCCAAGGATTAAAGATATGTGTTGCTTATGCAGAATGTCTTGAAGAATGTTTCAGTGAAAGACACTTCATAAAAGTGAGCAATAAGACCAAGGGAATCAAATGGATTTATGGTCCAACAATTTTTGGAATTGCAGGTCCTGGTAATCCAATGCTATGGTTTAGTCATTGGAAGTTTGGAAACCAGCTAGAAAGAGGAGATCAAGTTGTTGTTTCAGTAAGCATGGGTTGCTTAGTTAAGAAATTTGGTGTATATCTTGTATGCAGCGAACAAAGTGAGGAAGAGGAAGACACCCTATCTAATGCAGAAGGATGTCGCCGTCTTTCCTATCCACTACAGCATGCAATTGGTGGAGATTTGTCTCCTTATGAGCTAAGTTCAGGTGTCTATCACCTCTCCATTTATTCTTGA

>PGSC0003DMG400020722

ATGGCTAACATAAGTGAACTATGTTCAATGGCCACCAGACAATTAGCTTATGATCAATCATCCAATACTTACGGAAAGTCATATGATGTGTTTTTGAGTTTCAAAAGAGAAGACACTGGTAAAAATTTCACAAATCACCTCTCTACAGCTTTAAATCAAGCAGGATTTCGCACGTTCGAAGGTGGTGATAATGAATCAAGAAGAGAAGAAGACATTAATTCAGAGTTGTCTAAAGCAATACAAGAATCAAAGATGTGTATAATTGTATTCTCACAAAATTATGCATCTTCTAGTTGGTGCTTGGATCAACTTGTTTCCATTCTTGAAAAAAAAATGAAGTTTGCATGTATGATATTGCCTATTTTCTACCATGTTGATCCTTCCAAATCTAAGGAAGCACAAGGGAAGTTTTGGAGAAGCATTAAATTTTTCAGACATGAATCCACTTTCATCAAGAAGATCATTAACGTTATCAGCACAAGATTAAGTCGTCCAGCCTTATATATTGCTTCTTGTTCAATTGGAATACATCGTAGAGCCAGGCCCATTAATTCTTGGGTTCAAACTGATGTATCGAACAATTCCAATATTGAAGTACTTCTAGTTTGTGGTATTGGTGGAATTGGAAAAACAACACTTGCCAAATTTGTTTACAATTTAAACTTTGGATATTTTGAAATTAGTTGTTTCTTAGCAAACATAAGAGAAACTTCAAAACTCCCTAATGGTCTCATCACTTTACAAAAGCAACTTCTTTCTATCCTTCGAAAGAATGAAAAGGTAAAAATATCAAGTGTTGATGAAGGAATCATCAAGATAAGAAATGCTTTATGTTATCGAAAAGTTCTTCTTGTTCTTGATGATGTTGATGAGCCTGATTTGGTTGAAGCTATATTTGACATGAAAGATTGGTTTGGTTTTGGAAGTAAAATAATTGTAACAACTAGGCACAAAAGTTTATTAAGACCTCAATTAGTCCATGAGGTACATGAAGTTGGAATTTTATACACAATTGAAGCAAATGAGCTCTTCAATTTTCATGCATTTGGTCATGAAAACAATCAAATAAGCAAAGATTATTACAAAGAGTACTTAGAGGAAGTGATTGAATGGTGTAGAGGACTTCCATTAGCTCTTCAAGTTATCGGTTCTTCGTTGGCTGGAAAATCAAAAAATGTATGGAGAAGTGCTATAGAAAAGTTGAGAGAAATTCCCACAAATAAAATTGTTGACAAATTGAGATTGAGTTATGAGTTATTGGAAGATGATCATGATCAGAATTTGTTCCTTCATCTTTGTTGTTTCTTTGTGGGGATGAAAAAAGATTTTGTAGTTAGAATACTTGATAAATGTGATTTTTACACATTAGTGGGGATACAAAATCTGATTGATAGAAGTTTAGTGACAATTGATGAATATGTAAATGATATAAGAATGCATCAATTAGTACGTGATATGGGAAGAGATATTGTTCGTAGAGAAGCAACTGTGGATTCTGGAAAACGTAGTAGGCTTTGGCATCACACAGATTCTTACAATGTTCTAAGAGGAAAAACAGTAAGTAATTAACCATGTTCTAATTATTTGAAAAATTTTGTCAACTTTTACTAGGGAAATCAACTTTGTTATTTAGAGCTGATATATTTTCATTATAAAAGTAGCTCATATATACCATTACTGTTATACAAATTTCTCCAATACCTAGAGATACTAGATTTGAGCCACTCCTATGAGCTCATCACAACACCGGATTTCTCAGGCCTTCCCAATCTTGAAAAGTTGATACTCGAACATTGTACCAAGTTAATCAATGTTCATAACACCATCGGATGTCTACAAAAACTCATGATTTTGAGCCTTAAAGATTGCCAAAAGTTAAAGAGTCTTCCAGATAGCATTTGTGAGTTAAAATCTCTTGAGACACTTAACATCTCAGGCTGCTCAAACATTGTATATTTACCAACAGAGCTTGATAAATTAACATCACTAAAGGAACTTTATGCTGATGGGATCTCAATGATCAATTTGGAGGCCCAAACATGGTATTCATCTTTATGGGCTTGGGCCTGGAAAGGAAGTGGCCCATTATTATCTCCTAAAATTCATTTTCCTAAGTCCTTACATGTGTTAAACATTGCAAAATGCAATTTATCTCCAGATGCTTTTAACAAGGTTGATTTAGGCATTATGACCTTACTACACTGGCTAGATTTGGGAGGAAATCCAATTAACGATTTACCAGATAGTATCAAGAATCTTACGAGGCTAAAAACTCTTAACATCGCGTATTGTACAAAGATCAAGTACCTCGAAGGGGTACCATCAAATGTAACGGATGTAAATGCTGATGGATGTATATCATTGGAGAAAGTAGGTTCTTGTGCTAAAGGGCATCCAGTAGAAGGTTATATAAATTGTATAAATCTTGTTGAAGTTGAAGGTGTGTTCAAGTTAGAGCCATTGGAAAATGTTGATCCTCAAGTGCTTGCCAACAACATGGGAATTTCAAATTTGGAAACTATAATGAAAAGTACTATGGTTTCTCTTGTATTTGGTCGTGTGTATAATGCTGAAAGACTTAGAAATGAATATCCAGGTTTTGTACAAGATGACATAACAAGTTTATTTTCATTATCACCTAAGAAGTTACCTCCACAGGTTTGTCTCTCCTATCCTCTTTTTACATTCATTAAT

>PGSC0003DMG401020721

ATGGAGCAAGCTAAATTTTCTTACCATGTTTTCTTGAGTTTCAATGCCCATGATACTGGCAAAACTTTTACTGATCATCTCCATAGAAATTTGTTTCGAGCTGGGTTTCATGTGTTCAAGTGTGAAGATGATGATGAAGAAGAAGACAAAATAGATGAGTTGAAGTTAAAATTGAAAAAGGGAATCGAGCAATCGAAGATGTCAGTTATTGTTTTGTCCCAAAACTACGCATCATCGGAAAGGTGTCTTTACGAGCTTGTCGTGATTTTGGAGCAAAGGAGGAACTCTGGGCATATAGTCTTACCTGTCTTCTTTAACGTGGATCCTTCAGATGTAAGGAAGCAAAAGGGAAGTTTTGGGAAAGAAAATTTCCAGATTGTCAATGGTGAGAGTCACAAGTTAAGAGATTGGAGAAATGCTCTCAAAGAAGTGGCAGATTTGGGAGGAATGCCCTTGCAAAATCAAGCTGATGGGACTTGTAGACATGAGGCAAAGTTCATTGAGAATATTGTTGAAGTGGTTGCAAACAAGCTACGTCCCAGAGACTTACACAGTGCTCCTTACCTCATTGATATCAATTATCGGGCTGAAGATATTATCTCCTGGTTACAAGATAGATCAACTAATGTTGGGCTATATGTGATTTGTGGGATTGGTGGAATTGGGAAGACAACCATTGCCAAATTTGCCTATAACTCAAGTGCAAGATCATTCGAAGGAAGCAGTTTCCTTGCGAACTTCAATGAAATTGCGAAACAATGTAATGGCTTAGTTCGTTTGCAGAAGCAAGTTCTGTATGATATTGTTGGGAGAAAGGAGAGGATATCGAATGTTGATGAAGGAATTATGATGATTGAAGATGCCGTAGGCTATAAAAAAGTTCTTCTTGTTCTTGATGATGTTGATGATGCTGACCAGATAGATACAATTTTAGGAATGACAGATTGGCTTAATCCTGCTAGTAAAATCATTATAACAACGAGGCATGAGTCATTGCTAAGGCCTTCTGTACCTCACAAGGTGCTTAAACTAGAAGTTTTGAATAAAATGGATTCCCTAAAGCTTTTTAGCTGGCATGCCTTTGGGGAAGATCATCCTTGGGAAGGTTATCTTGAGCTCTCAGAAAGAGTGGTTCTTCAATGTGTTGGACTTCCTTTAGCTCTTCGTGTTTTAGGCTCAGCTCTATCGGGTAGAAGACCAGAAATATGGGGAAGTGCACTAGAAAAGCTGGAAACAATCCCTGATGGTCATGTCATTGAAAAACTTAAACTGAGTTTTGACACTCTTGAAGATGACCATGACAAAGATATATTCCTTCATATAGCTCATTTCTTTCTTGGGATGGACAGAGATGACTCTGTTAGAATACTGGATGGATGTGGTTTCCACACAATAATTGGGATGCAAAACCTCATTGATAGAAGTCTTTTGACAATCAGTGACTTAAATAAGTTGGAGATGCATCGGTTGCTTAGAGACCTGGGGAGAAATATTGTTCGTATGGAATCACTGGATCCTGGAAAACGTAGTAGACTTTGGAATAACAAGAATTCTTTCAGAGTATTGAATTATAAGACTGTAAGGATGCTTTTTTCCAATCAGGTTTTGAGATTTCTCAAGATTCTCAACCTTAGTCATTCTTGGGAACTTACCAGAACCCCTAGCTTCGCTGGAATGCCCCGGCTGGAGAAATTAATTCTTAAAGGCTGTGTTAAACTGGTTGATATTGATGAATCTATTGGTTATCTCCAAGAAATCGCCCTACTGAATCTAAAATACTGCAAGAGCATCAGAAAGCTGCCAAGAAATATTGGTAAACTTGAAAGTCTTAAAACACTTGATATATCCTTCTGCTCAAGTCTGGAGTCGCTGCCAATGGAGCTTAACATGATAGATTCTTTGAAAGTGCTAAGAGCTGATGGAATTGGTCTAAACCAAATAGTCTGTAACACCAATGAGCAGAAATCATTACAGGCACTATTTTCATCTTGGATATCAAAGCCAAGAATCTCTCCTGAAATATCATGGGCCTTTTTACCAAGCTCTTTGATGAGCTTGAGCCTTGTGGGTTGCAGGCTGTCTGATGGATCTTTTCCCAAGAAGTTTAGCAATCTTCCGCTGCTCGAAGAACTGGATCTAAGTGAAAACTCAATTAGTTGCCTCCCAGAGTGGATCAAGAGTCTCCCTCAGCTCCAGAGCCTCAGTGTCAAGTCTTGTAAGATGCTCAAATCACTTACTGAGATGCCAAATAGCATTTCAGAATTGTCAATACATAGTTGCTCATCGTTGGAGATAGTGACATACCAATCATTGCTATTGGAATATCCAAGCCTGGTTCACGATTACAATTGTGACAGTCTAGTCATGATGCAGGGAGATTTTAAGTTAGAAGCTCTAGAAAATGCTGACCCGCAGATGCTTAAACGTTTTGGTCTGAATCCTGAAACTGTGGAAAATACTATGGTGAAAATGGACCTCTTTTCAAGTTACAAGATAAAAATGCTTCCGCCACAGGTTTTTCTCTCACGCTGTCCCTATGACTACAAAATCTAG

>PGSC0003DMG400003104

ATGGCAGATTTGGTACTATATCCTGTTTTACAGGTGATCCTTGAAAAGTTAGCCACTCCATATGTGCAGAAGTTCCATGATCTCTACCATTTGAAGGAAAACATAGAAAAACTGCAGAATTCACTACCAACTGCTAGAGCTTTTCTTGATGATGCACAGAAGCGACAAGAAACGGATCAACATGTTGAAAACTGGTTGGTGAAGCTCAAGGACATAGCTTATCAGTTGGAGAACTTACTGGATGAATTTACTGCAGAAAGTGTAATGTGCGAGCATCGCAGTGGAAAAGGCAAACAGGTAAGCAGTCTATTTCTACCTTTTGAACCATCAAAGCATCTTTTTGACCTTGCAAAAATGCTACCAAAAAAACTTAAGGAACTAGATGAGATTGCAAAGCAAGGCTTTAGCTTAAATCTTAGAGCAACAACTACAGAAAGACGAGCTGACGATTATGACAGAACAAAAGTAACTGGATCGTTTGTGATAACATCAAAAATATGTGGAAGAGATGATGATAAAAAGAAGCTTTTAGAGCTTTTGTTGACTGCATGTGATGGCAAAACTGGTGGGGTTCTTTCTATCATCCCAATTGTAGGTATTGGAGGACTTGGCAAAACTACTCTAGCTCAACTAGTCTATAATGATGAAAAGGTCGTTCATTTCTTTGACATAAAAATATGGGTTTATGTGTCTAGGGATTTTGATGTAAGTAAACTCATGTTAAGCATCATACAGTCTGCAACAAAAAGGAAGTGTGAACTCTTGGAGATGGATCTACTTCAAGCTCATTTTCAAGATTCATTGGGTGGAAAGAGATTCTTGATTGTTTTGGATGATGTATGGAATGAAGACCAAGAAGAGTGGGATAAGCTGGGCGACTTATTGCAAAGTGGTGGAGCAGGAAGTAGAATCATTGTCACTACACGGAGTACCAAGGTTGCGTCAATAGTGGGAACTACTTCCCCTTATTGTCTCCAAGGCTTGACTGAAGATGATTGTTGGGTTTTGTTCAAGCAACGAGCTTTCAGCCAGGAAGAAGAAGCTGAGCATCCTAATCTATTGGAGATTGGAAAACAGATCATCAAGAAATGTGGAGGTGTGCCTTTGGCAGCAAAAACACTGGGAAGTTTGTTGCGCTTCAAACGAGAAAGAGAAGATTGGATGTTTGTGCAAGAGAGTGAACTTTGGAAACTTGAGAATTGTAACAGTGGCATACTGCCAGCTCTTCGGTTGAGCTACTTACAGTTGCCTCTACACCTCAAGCGATGCTTTGCATTCTGTTCATTGTATCCAAAAAATTATGAAATTCATAAGGAAAAGATGATCCACATATGGATTGCTGAAGGCTTGATAACATGTCATGAGAGGAACAGGCAGTTGGAAGACATAGGCAACAAGTATTTTAATGACTTGTTGTGTTTATCATTCTTCCAGGAAGTCAAGAAATTTGATGAGACGGATCTAGCAGTATACAAAATGCACGATCTCATCCATGATCTTGCTCGAAGTGTGGGGAGTCAGGACTTCGTGATATTGGGACATGACTTTGCTCAAGGTAACATGTCACGGGTTCATCACCTGTCAATCCTTTTCCATTCAGATCCCACTTCACTTCCAAAGGAATTGTATGGTGCCAAACATCTTCGAACACTCCAATTCCTGTTTTGCACCGGAGACATTCCTTCTTCTTTTCCTCTGAACTTTAAATACTTGCGAGTCCTTGATTTAAGTGGTTGTGTGAAGAAGGTGCACGAGTCAATTAGTGATTTGATATGTTTGAGATACCTTGATCTCTCCAGAACCTCTATTCAAACACTCCCTCATACTATTTGCAACCTTTTTAATCTGCAAACCCTGAACCTCTCATTTTGTGGTAACCTAATGGAGTTACCTTTTGGATTGGCTAATATAACTGGCTTAAGGCACCTTAACATAGTGGGATGTAATGGACTGACTCGTCTCCCTGCTGGTCTGGGAAATTTGGTTCAGCTTCAAACTTTGCCTTTATACATTGTGGGCAAAGGGATTGGAGAAAGCATCTCTGAAATCAGTTCTCCCCATATAAGAGGTGAATTGAGTATAAGGGGCCTGGAGAACATCAGAGACAAAGAAGAAGCTACACTGGCTAATCTGAGGGCGAAGAAATATGTTGAATTGTTAAGACTCCAATGGGGAAGCGAGAACATAGTGAGGATGTCAACAGGGTCTACTTCATACGAGGTTTGCAGAGAAGTTGATGGTACATCAAGATCACTATCAAGGGATAATGACAATGTTGTGGAGGGGATCATTGAGTGTCTTCAGCCACATGTAAATCTCAAAAAACTATATATCAAGGGATATCCTGGATTCAGATTTCCAGATTGGGACCTCCCTAATCTAGTTCTGATTGCTTTGATCAATTGCAGGGGATGTGATACTTTACCTACCTTTGGGAAGCTTCCGTTTCTCAAAACACTTTATCTACAAGGGATGGATGGAGTTACACATATTGGTGAAGAATTTTATGGAGGCGAACCCCTCAAATTCCCATCCCTGGAAGACCTAACAATCAAGGATCTTCCCTGCTTAAAAGAATGGTCTTGCATAGAGAACGGAGCAGCAGTACTTCCTCGCCTGCAAAAGTTAGTTGTAGATAAATGTCCTAATCTTATCTCTGCTCCAACATTTCAATCTCTTCTTCATTTGGAGCTCCATGATTGCCATCCAAAGATCTTGGAGTCTGTGGATAACATGTCTTCACTCTCCAATCTTGTGATTGATGCGCTTCAAGGTTTAGTGCACCTTTCAGGGAAATTGCTGGAGAATAACAAATCGCTGGAGACTGTGGAAATACTTTCATGCAAAAATTTCATTTCTCTTCCTCAAGAGATAGAACATCTCACTTATCTGAAGTCACTGACCATTAGCTACTGTGAAAAACTAACCCATTTGCCAACAGGAATCCGAAAGCTGCAAGCTTTGGAGTTTCTTGAGATCAACGGGTGCCACAGCTTAGAGTCATTGCCATCAGAGGAATTTGCAGGTTTCAATTCCCTAAAGAGCTTGTCAATTGAGAATTGCAGCAACCTAATTTATCTTTCAAGTGGGTTCCTCCATCTCACAGTCTTAGAACAGCTCTCCATCATGGGTTGCCCTCGGTTAACTTTATCTAGAGATAGTTTCCAGAATCTCTCATCTCTACGGAGCTTGAGTATTATCTCCTGCCCTGAATTATACCCATTGCCAGTTAGCCTTCAACATGTCACTACATTGCAAAGTCTGGTAATCCATAGCAGTCCTCATCTCACAGATCTTCCTGACTGGCTTGCTAAACTTTCCTCTCTTAGATCTCTTGCAATTTCAAATTGCGAACATTTGATATCTTTGCCAGAAGGAATGAAGTACCTCAATGCTCTTCAGCATTTGTCAATTCAAGACTGTCCTCATCTTGAAAGGCTTTGCAAGAAAAAAGGTATGGAATGGCGAAAAATAGCTCATATTCCACACATATATGTTGGTTCACTTAAATTTGGGAGATGA

>PGSC0003DMG400019900

ATGGTGGACGCTTTCCTTCAAGTTCTTCTAGAAAATCTGGCTTCTTTCATCGGAGATAAATTTGTATTGCTTTTCGGTTTTGAAAAGGAATTTGTAAAGCTGTCGAGTGTGTTTTCCACAATTCAAGTTGTGCTTCAAGATGCTCAGGATAAGCAATTGAAGGACAAGGCAATTGAGAATTGGTTGCAGAAACTCAATTCTGTAGCCTATGAAGTTGATGACATATTGGGCGAATGTAAAAATGAGGCAACAAAATTCAAGCAATCTCGATTTGGGTTTTATCATCCAGGGATTATAAATTTCCGTCATAAAATTGTCAAAAAGATGAAAGATATAATGGAGAAACTAGATGCAATTGCTGAGGAAAGAAGCAAGTTTCATCTCCTTGAAAATATTATAGAGAGACAAGCCACCTCCACGCGTGAAACAGGTGTCACTACTAATTGTTGTCCAAGAACTTTCGATTTTTCCTATAATTGGTATGGGGGACTAGGAAAGACAACACTAGCCCAAATGATCTTCAATGATGAGAGAGTGACTAAGCATTTCAATCCCAAAATATGGGTTTGTGTCTCAGATGATTTTGATGAGAAAAGGTTGATTAAGACAATTATAGGAAGTATTGAAAGAAGTTCTCCTGATGTCGAGGACTTGGCTACATTCCAGAAGAAGGTTCAGGAGTTATTGAATGGAAAAAGATACTTGCTTGTCTTAGATGATGTTTGGAATGATGATCTAGAAAAGTGGGCTAAGTTAAGAGCAGTCTTAAAGGTTGGATCAAGAGGTGCTTCCATTCTAGCTACTACTCGTCTTGAAAAGGTTGGATCAATTATGGGAACGTCGCAATCATATCATTTATCAAATTTGTCTCCACATGATGGTTTACTGTTGTTTATGCAACGTGCATTTGGGCAACAAAGAGAAGCAAATCCTAATCTAGTGGCCATTGGAAAGGAGATTGTGAAGAAATGTGGTGGTGTACCTTTAGTAGCTAAAACTCTTGGTGGTCTTTTACACTTCAAGAGAGAAGAAAGTGAATGGGAACATGTGAGAGACAATGAGATTTGGAGTCTGCCACAAGATGAAAGTTCTATTTTGCCTGCTTTGAGACTTAGTTGTCATCACCTTCCACTTGATTTGAGACAATGATTTGCATATTGTGTAGTATTCCCAAAGGACACCAAAATGATAAAGGAAAATCTCATCACTCTCTGGATGGCACATGGTTTTCTTTTATCAAAAGGAAACTTGGAGTTAGAGGATGTGGGTAATGAAGTATGGAGCGAATTATACTTGAGGTCTTTCTTCCAAGAGATTGAAGTAAAATCCGGTAAAACGTATTTCAAGATGCATGATCTCATTCATGATTTGGCTACATCTCTGTTTTCAGAAAGCGTATCAAGCAGCAATATCCGTGAAACAAATGTAAAAAGTTACACACATATGATGTCCATTAGTTTCGCTGAAGTGGTGTCTTCTTACTCTCCTTCGCTCTTGAAAAAGTTTGTCTCATTAAGGGTGCTTAATCTAAGTTACTCAAAACTTGAGCAATTACCCTCTTCCATTGGAAATCTATTACATTTAAGATACCTGGACCTGTCTCGCAATAACTTTCGTAGTCTTCCAGAGAGGTTACGCAAGCTTCAAAATCTGCAGACTCTTGATCTACATAATTGCTACTCACTTTATTGTTTGCCAAAACAAACAAGTAAACTTGGTAGTCTCCGAAATCTTGTACTTGATGGTTGTCCATTGACTTCTACTCCACCAAGGATAGGATTGTTGACATGCCTTAAGACTCTAGGTTGCTTTATTGTGGGAAGCAAGAAGGGTTATCAACCTGGTGAACTAAAAAATCTAAATCTCTATGGCCCAATTTCAATCACACATCTTGAGAGAGTGAAGAATGATAAGGATGCAAAAGAAGCCAATTTATCTACAAAAGCAAATCTGCATTCTTTAACCATGAGTTGGGACATCGACGGGCCACATAGTTATGAATCAGAAGAAGTTAAAGTGCTTGAAGCCCTCAAACCACACCCCAATCTGAAAAATTTAGAAATCATTGCCTTTGGAGGATTCCGTTTTCCAAGCTGGATAAATCGTTCAGTTTTGGAGAAGGTCTTCTCTATTAGAATTACAAGCTGCAAAAACTGCTTGTGTTTGCCACCCTTTGGGGAGCTGCCTTGTTTAGAAAGTCTAGAGTTACAATACGGGTATGCGGAGGTTGAGTATGTTGAGGAGGAGGATGCTCATTCTAGATTCCCCACAAGAAGAAGATTTCCATCCCTGAAAAGACTTTGTATTTGGTTCTTTCATAATTTGAAAGGATTGGTGAAAGAGGAAGGAGAAGAGAAATTCCCCATGCTTGAAGAGATGGAGATTCTATATTGCCCTATGTTTGTTTTTCCAACCCTTTCATCTTTCAAGAAATTAGAAGTTCATGGCAGCAGAAATGCAACAGGTTTGAGCACCATATCTAATCTTAGCAGTCTTACTTCCCTCCGCCTTGGTGCTAACAACCAAGTGACTTCACTCCCAGAAGAGATGTTCAAAAGCCTTACAAATCTCAAATACTTGACTATCTCTTACTACAAGAATCTCAAAGAGCTGCCTACCAGCCTGGCTAGTCTCAATGCTTTACAGAGTTTGACAATTGACTATTGTGAAGCCCTAGAGAGTCTTCCTGAGGAAGGGGTGAAAGGTTTAACTTCACTCACAGAGTTATTTGTTGAACAATGTAAGATGCTAAAATGTTTACCTGAGGGATTGCAGCACCTAACAACCCTCACAAATTTAGGAGTTACTGGTTGTCCAGAAGTGGGAACGCGCTGTGATACAAAAATAGGAGAAGACTGGCACAAAATTGCTCACATTCCAAATCTGGATATTCGTTAG

>PGSC0003DMG400013091

ATGGCAGCTTATAGTGCTGTAACTTGTCTTCTTCAAACTCTTGAGCAACGAAATCCACAACTCTTTGATGCTCTCACTGCTAAAGCGCTCGAATCTATTCATGCTACTGCTCAATATTTTTATAATTTTCTTGAAGGATCAGGCAATAAGAATGTTGAAAAAATCAAATCTTTGGAGGAAAAAATTAGAATTGCTGCTAATCATGCAAAATATGCTGTTGAGCTGAAGATTTCCGATATAATCGATGGCATATCCTACACAAATGCAAATGTACAACAGGAGGATTTGCCAGTAGTTGTTGATGAAATGTATACAATAAAGAAAGAAATGATGGAGATTATTGCTTCTGGTTTTAACACAACTACTCATGATGATGATGATGATCAAATTCTTGAATTATCGAGGGATTCCTTAATTGGCACTTCTTCTACATATTTGGAAGATGATATTGTGCACGGACTTGATGATGACTTGGAGATCATAATTAAAAGATTATTGACAGGACAATCGCGGGATCTTGAAATTGTCACAATAGCAGGCATGGGTGGCATTGGCAAGACCACACTCGCTAGAAAAGCGTATAATCATCTCGCAATCAGGTATCATCACTTTGACATTCTTGCTTGGGTTACAATATCTCAAGAATTTCGAGGTAGAAATGTGTTGTTAGAAGCTTTACGTTGCATTTCAAAGCAAACAGTTAGTTCCAATGCAAAAGATTATGATAACAAGGATGACTCTGACTTAGCAGGCCTAGTGAAGAAAGAGTTAAATGGTAGAAGATACTTTGTTGTTGTTGATGATATTTGGAGTAAGGACGTTTGGGATAGCATAAGAGGAATATTTCCTGATTGCAACAATAGGAGTCGAGTCTTATTGACTACTAGGGAAACTGAGGTAGCGATTTATGCAACTACTAGTAGCCCTCATAAGATGAACCTCTTGGATTTAGATAACAGTTGGAAGTTACTTCGTGATCAGGTTTTTGGACTAGAACACGATCATCCTCCTGAGTTGGGAGAAATTGGAAAGGAAATAGCAGAAAAATGCCAAGGACTACCCTTTACGATTTCAGTTATTTCTGGACATCTCTCTAAAGTGGACAGGACATTAGAAAGTTGGAAGGATGTTGCCCGAACCTTAAGCGAAGTCCTTGCTAGTCATCCAGATAAATGCTTAGGAGTGCTCGGTTTGAGTTACCACCACTTGCCTATTCACCTCAAACCTTGTTTTCTTTCTATGGCTTGTTTCCCAGAAGATTTTCAAGTTGATACTCGGAGATTGATCCAATTATGGATCGCAGAAGGTTTCATAAGGATGTCTGGTAGAAGTCGTAAAAGCTTGGAGGAAGTGGCGGAATATTATTTGGAGGATCTTATTAGCAGGAACTTGATAATGGCAAGAAAAAGGAGATTCAATGGTGAGATAAAAGAATGTGCAATGCATGATCTACTACGTGAATTCTGTTTGATAGAAGCTAAGATGACAAAGTTTATATATGTTGTGAGAACTGACCAAGAAGTTCTTACTCTTCCAACACAAAAGCATAATGGTGGTCGCTTCAGTTTTCAATCCCTATTTTGTTCAGCTGAAGATTTTATCTATCGATTACCATTTTTTACCAGATCTATCTACATCTTTTGTTCAACACGCTTTGTTCATTTCAGCCATTTCAATCTTCTTAGGGTATTGGTCTTCCCCCATTCTTGTTTTGCATTTCCATTTGACATTACTAATTTATTTCATTTGAGATATCTTGAAGTTGAAAGGTTTGATTGTCCTCCTGAATCAATCTCAAAGCTTCAGAATTTGCAAACTCTAATTCAGCATGAACAATGTGGGAGCTATATAACCGTACCAGGGACGATTTGGTTGATGAAGAACTTGAGGCATGTGTATCTGGGGTCACCGAGTAATTTACCCAGTCCTAGAATATCAGAACTTGAGATAGGAATATTGCCAAATCTAGAGGAACTTTCTGGTCTATGTTCCTTCAGTTGTACAAATGACGTTTTCAAAAGCATTCCCAATCTAAAGAGATTAATGGTTCGTGTATCTTGTTCGAAAGAAGATGTTATGACCAAACGCCTTATTGATATGTCCAGCTTGACAAAACTTGAAGCATTGAAGTGTTTTAGAAATGCTTGGGAAGCGATTTCCATCAGGAGGTTCAGTTTCCCAACATCACTTAAAATGTTGACTTTAACCAGTAACTATCTTTTTCCGTAGGAAGAAATATCAACTCTTGCATTGTTACCGAATCTTGAAAAGCTCAAACTTAAAGAATCGCCAGCTACAGGTGAAGTATGGAGACTGAGTGATGACGACAAGTTCCAAAGTCTCAAGCTGTTGATATTTAGCTATCTACAGTTTCAGCACTGGGAAGCTAGCAGTGATAGCTTCCCAAATCTAAAATGCCTTGTTCTGAAGAACTGTAGCTTCCTTCGAGAAATTCCAACAGATTTTGGGGAAATTGGTACTTTGGAGTCAATTGAGTTGCATTATTGCGGCACTGGTGCTGAGGATTGTGCCAGAAAGATTGAACAAGAACAAGAGGACATGGGAAATAATTCCCTTAAGGTCTACATCCATAACAGTCGTGGTAAGTTTTAA

>PGSC0003DMG400013095

ATGGAAGCTTTAAGAGTTGAGGAAGCCTCAAATTTCAGGTCCCAATGTATGTACGATGTGTTCAGGAGTTTTAGAGGCGAGGATACTCGTAAGACTTTCACTGATCAGCTTTACAAAGCTTTGGTGGATGAGGGTTATCGAACATTCAGAGATGGTAATGAAATTGAAAGAGGGGAAGATATTAAATCTGAATTGGATAAAGCAATTCATAGCTCAAAGAGTTCAATTATCGTCTTGTCAAAAAATTATGCAACGTCTAGTTGGTGCCTTAATGAGCTTGTCATGATTTTTGAAAACAAGAGGAAAAGAGGACATGCTATATTGCCAGTTTTCTACTATGTAGATCCGCTCGATGTTGGAAAGCAAATGGGGAGATTTGCTACTGCATTTTCTAGTTATGAACAACAAATAATGGAACAATCTGATTAA

>PGSC0003DMG400013094

ATGGAAGCTTTAAGAGTTGAGGAAGCCTCAAATTTCAGGTCCCAATGTATGTACGATGTGTTCAGGAGTTTTAGAGGCGAGGATACTCGTAAGACTTTCACTGATCAGCTTTACAAAGCTTTGGTGGATGAGGGTTATCGAACATTCAGAGATGGTAATGAAATTGAAAGAGGGGAAGATATTAAATCTGAATTGGATAAAGCAATTCATAGCTCAAAGAGTTCAATTATCGTCTTGTCAAAAAATTATGCAACGTCTAGTTGGTGCCTTAATGAGCTTGTCATGATTTTTGAAAACAAGAGGAAAAGAGGACATGCTATATTGCCAGTTTTCTACTATGTAGATCCGCTCGATGTTGGAAAGCAAATGGGGAGATTTGCTACTGCATTTTCTAGTTATGAACAACAAATAATGGAACAATCTGATTTACTCTGTTTGTCAACCGTAACACATATGACTAACCAACATTTCTGTTTTTGTTTGTTCCACTTTGATCTTTATTGTAGGACTGATGAGTTGAGATTTATAGAGAAACTTGTGAAAGTTACTGAAAATAAAGTAAACCGTTCAATCCTCAGTGTTGCTCCTTACCTAATTGGTATACATTTTCGGGTTAACGATATTTGCTTGTGGTTGCAACACAAAACAAATGATGTTGGTATTTGGGGCATTTGTGGTATGGGTGGTATAGGGAAGACAACAATCGCCAAATATGCATTTAATTCTAACTTTGAAATTTTGAAAGAAGCAGCTTCCTCTACAATGTTAGAGATTTTTCCGAGTCTACCGATGTACCTAATTTATCTTCAGAAACAATTTCTTTCTGATTTTCTTGATGGGAGGAAGATTGACATTCAGAGTGTTGTAGATGGAATCAATCAGATCAAAAGTGCTGTTTATGGTAAGAGGGTTCTCGTTGTGCTGGATGATATTGATGAAGTGGACCAATTAGCTGCTATCGTTGGAATGCGAGATTGCTTTTATTCAGGTAGTAAAATTATAGTGACAACTAGGCAGATAGAGTTACTGAGGGCTTGTGAAATTGAATTGATCGATGATGTTCAGAAACTGAACAAAGACGAATCCGTTGAACTATTTAGTTGGCATGCATTCGGACAAGGCCATCCAGTCGAGCATTACACCAAGTTCTTGAGAAGGATTATAGAATACTGTATTGGATGTCCTCTAGCTTTACAAGTTTTGGGTTCTTCTCTCTCTGGTAAAAGTCTAGATGTATGGGAAAGTACACTGAAGAAACTAGAGGTAATTCCTAATAGTCAAGTATCCAAGAAATTGCAGATTAGTTTCGCCTTTATACAAGATGATCATGACAAAAGCCTATTCCTTGACATTGCCTGCTTCTTTCTTGGAAAGAATGTAGACCACGTTGGCACTATACTTGATGCATGTGGATATTATTCAATGGTTGGGATTCAGAATCTCCAAGATATATTCTTGTTAAAAATTGATGAATATGGTAGACTGATGATGCATCCATTAGTTAGAGATATGGGACGAGAAATTATACGTCAAGAATCACCTAAGCATCCTGAAAGACGGAGTAGATTGTGGCATTACAAGGACTCATTTAAAGTTTTGAAAGAAAAAAATGTAAGATTTTATATATGTTGTTTCACAAATCAATTGATCAATTTATGTTTTCATCAGGGTTCAGATATAATTCAAGGCCTGAACCTTAGGTCTTGTGCAGAGAAAGATAAGCCACCAAGGAGCTTCCATCATCCAACAACACAGGAGTACTTGCAGGAATGTGCCAATATAATGAGGAAAATAAGACTGTCACGCCAAAATGATCCACCACGGCAACCACCACCTCATAAAGTTCTCCTTTATGGTTCTAATAATAAAAACTCTGAATCAAAGAAGGTGGACAACATCAATACTGATGCCTTTTCTGGTATGCGCAAATTAAAACTGCTGCAACTTGATGGTTGTGCTGGCACAAATTCCCATATAAATGTTTACCTGATAGCTTACCTTTGGAGAAAGTGGTGGCCCTGGAAATGCGCCATAGTAGATTGCATCATCTTTTCCAAGGAAACAAGGGTCAGATACAGATTTTTATTTGTTTTTCTTACAGGTTTTCAGTGCGTTGAAGATTCTGATCTCAGTCATTCAGAAGGTCTGTCCTGCACCCCTGACTTTTCAAAGCTCCCAAATCTTGAGAGGATAATTCTCAAGTACTGCACTAGGTTGATTGAGATTGATAAATCGATTGGGGAACTAAAGAGACTGCTGATTTTGAACCTAAGAAGCTGCCAAAGTCTAAGGAGGCTTCCCAGATGTATTAGTAATCTTCATTCCCTCGAGAAGATGATTCTGTATGGTTGCTCAAAACTTGTATGGTCTTCATTAGAGTTGAATAAGATGCAGTCATTGCTAGAACTTGATGCCGGTGGAACTGCTAACGATCAAGTTTCTACTTCTGTAAGTAAGAAGCATTTGCTGATGTGCTCTTCAGTCTCAAGCCCAAGAAAATCTCCTGGAATCCTGTTAACTACAGTATCTCATACATTAGTAACCTTAAGTCTTATTGGATGTGGTCTATCTAGTGATTTGATTCCAGAGGAGATTGGTGATTTCTCCATGCTGCGGAACTTGTTTTTAAGTGAGAATCCAATTCACAGTCTACCAGAAAGCATCAAGAGGCTTACCAATCTCAGGAAACTTGAGTTAGAAAATTGTGAACAACTTCAATATCTTCCAGAGATCCCAGCCAGTGTGAAAATCTTGAGTACTTGGCAATGCAGGTCACTCCAACGGATACACAATTTACCAAATTTATTGACAACATTGAATTTTCTTGGATTATCTTGCAACAAACTTATTGAGGTTCAGGAAATGTTCCAGCTAAAATGTATTTCTTCATTTGATGCTGAGTTAATCAGTGTTCTGAGATTACCCAACTTGAGTGACATGAAAGTGGATCTCTACAACAGTCTGATTCTTACAAGATGGAAAGGTCCTATACAGGTTTGCTCCATCTCCTACAAGTCCCATTTATATTTCAAAATTGGTTAA

>PGSC0003DMG400013090

TCAAAGAAGAAAATGTCTCATCAGTTCGTTCATCATGTATTTTTAAGTTTCAGAAGCAAGACCTTTGGAGATCATCTTCATACAGCTTTGTTAAATGCTGGTATCCCTTCATTCAGACCTGATGATAAAGAGCTTGATAAAAAGCTGCAGAATTCAATTCAAGAATCAAGAATTCTAATAGCTATCATTTCAAAAGACTATGCTTCTTCATACAGATGCCTTGATGAACTCACTCATATGATTCAAACCAAGAAAGCTTTTGGAAATTTTCTTCTCCCTGTATTTTATGATGTAGATCCATCAGATGTGAGGAAGCAGAAAGGGAGCTTTGAAGAACCTTTCTTTAACTTTAAAAAGAGATATAAAACAGAGAAGGTGGATCAATGGAGAGCTGCTTTAAGACAAGTTGCTGATTTGGGAGGAATGGTCTTACAGAATCAAGCTGATGGGTAATATTTTTCTGAATAAAATATCTGTTTATCTCATTCAATTCTTGTTACTAAATTGTAAAACTATTTCAGATCTGAGTCGAGGTTTATACAAGAAATTGTCAAGGTGGTTGTAGGAAAACTGAGACGTACTGTATTAAGTGTTGATCCCCATCCGATTGGAATAGATTCTCGAGTCAAAGAAATTGATTTGTGGTTACAAGAGGGATCCAATAATGTAGATATTCTTGCAATACATGGTATGGGAGGTATTGGCAAGACTACAATAGCCAAAATAGCTTATAACCTGAATTTTGATAGATTTGAAGGCAGTAGCTTCCTTGCAGATGTTAGAAAAGTTTTAGAAAAGTATGATGGTTTAGCTCGTTTACAAAGACAACTTCTTTCGAATATTCTTGGGAAGAATGTTGAAAAGATATATAATGTTAATGAAGGGTCTGTCAAGATTCAAGAAGCCATCAGCTGCAAAAGAGTTCTTCTTGTTCTTGATGACATAGACAACATAGATCAGCTAAATGCGGTACTCGGTATGAGGGATTGGTTTTATCCAGGTAGCAAAATTATAGTAACAACGAGAAATGGTCACTTATTAAGTTCTACTGAAGCATGTAGATGCAGGATGTATAAGCTAAAGACTTTGGATGCCAAAGAATCACTTCAACTGTTCAGTTGGCATGCATTTCGGGATGAAAGCCCCCCGTTAGAATATATGGATCTTACGATAGATGTTGTACATCATTGTAAAGGAATTCCGTTAGCTCTTAAAGTGTTGGGTTCGTCTCTTGGTGACTTAAGTATAGAGATTTGGGAAAGTGCATTAAGGAAATTGAAAGCTATTCCAGACAGTAAAATCCTTGAAAAACTAAGGATAAGTTATGAATGTCTACCAGATGATAATGTTCAAAACCTATTCCTTGATATTGTCTGTTTCTTTGCTGGAAAAGACAGAGATTATGCAGTAACAATACTTGATGGATGTGGTTTCTTTTCAGTCGTTGGCATTCAAATTCTTGTTGACAGATGCCTATTGGCAATTGAACATAACAAACTGATGGTGCATCAATTACTTCAAGACATGGGAAGAGAAATTATTCGGGAAGAATCCCCTTGGGAGCCTAGTAGTCAAAGCAGGATTTGGAAACACAAAGATGCCTTTAACATATTTCAGGGGAAGACAGTAAGAAATCTGAATCTTCGATTTTTAACGCGCAATCATCCATATCTATGTGCCTTAATATATGATTTTTCGTTATCATGTAGGGTACTGAAAGGATCCAAGGTCTGTACTTGTTATTGGCTATTGCAGATGCTCAAGTTATTAAAAATTCTCAATCTCAGTCATTCCCATTTCCTAAGAAGAACTCCTGATTTTTCCGGGCTTCCTAATCTGGAAAAACTCATTTTGAAGGATTGTGTAAGATTGTTCCATATTCATGAATCAATTGGAGATCTACAGGAACTTGTTCTCTTGAACCTGAGAGACTGCAAGAGTCTATCGAATCTTCCAAGAAGCTTTTGTAAGCTCAACTCCTTGGAAACACTCATCATTTCTGGCTGTTCTGGACTTGCTCTATCAACAATTGATCTAGGAAATCTGGAATCTTTGAAAACCCTTCATGCAGATGAAATTAATTACGATCACGAGAAATCATGGGTTGCACTCTGGCAATCTTGGTCATCCAAATTAAGAAAATCCCCTGATTATGACAACTTTTCACTATATTCTTTATCAAGTTCACTGGTTAGCTTAAGTCTGGCAAGATGCAAGCTAACAGATGATGCTCTATCACTTGGTGTTAACAACCTCTTCTCATTACGCCATTTAAATCTAAGTGGAAACCTGATTTCTAACCTACCACAGAGCATCACAAATCTAAGTATGCTTCAGGACCTTTGGCTAGATGCGTGCCCGAACCTCCAATCACTCCCCAATCTTCCTTCAAGGCTCATCAAGCTGAAGGCTACAGAATGCACATCACTAGAAAGAGTTACAAATATGCCAAGCCTATTGGAGACTCATACATTATTCTTGGATGTTAGGGGCAGTGAAAAACTAACTGAGATTCCTGGATTGTTCAAGCTGGAGCCAATAAGAAATTTTGAAGAGGAAATGGTGAACACTCTGAACCATCTAAACCTGGATGATATACAAAATGCAGAAGTGGAACTATTTAACAGGCTTACCAACACAAAAAAGAAATTGTCACGACCCGATTTCTCAAGTCATGATGGCACCTACTATAACCCACCAGTAGGTAAGCCAACCCGTAACCCGGAACAACAAGTAATGGGTCTGAGGGTAGAAACTAAACAAGAGTAG

>PGSC0003DMG400012918

ATGGCAGAAGTGCTGCTAAGTGCTTCAGTTGAGGTTTTACTTCAAAAGTTACTTTTGCTTGCAACAAATTGTAATAGTCATTTATGGGGTTCCAAGAAAGAATTGGAAAATCTGAGGAGATGTTTAGCTATGGCTAGAGCAGTTTTACATGATGCTGAGAGGCAACAGAGAAAGGACCAAGCAGTGAAGCTTTGGCTGAAGAAACTTGAAGATTTAGCTTATGATGCTGATAATTTGTTGGATGAGCTCAATCACACAACACTCAAGAAAAGTGAGTGGAAGGTATGTTTTGTTTTATCACTACCAAATCCACTTTCTTGTAAAATGAGGGCCAAAATCAGGGAGATCATTGTGAACCTGAAAATGATTAATGAGGAAGCCAATGACTTTGCTATCCCAAGGGGAGTTGAGGATGGAATTAATCATATCAACCATAAGGAGACTGACTGTTTCCATGGTGATTCCAATATTGTTGGAAGGGAGGATGATGTTTCAACAATGGTTGAATCCCTAATTTGTCAAACCAACCAAGTAGTTGCAGTTTTCCCTATAGTAGGCATGGGTGGGCTAGGTAAAACTACATTGGCTCGATTGATATACAACGATGAACAAATAGTTAGATATTTTGATGAGAGAATATGGGTCTGTGTATCTGAGAATTTTGATGTGAACAAAATTATTAGACTGGTTTTAGAATCATTAACACAGAGAAGTATTGATGTGCAAAGTAGGAATGCTTTACTTCAAATACTTCACAAGGAACTAGGTGGCAGAAAATACTTGCTCGTTCTCGATGATGTTTGGAATGAAAAGCTTGAGGAGTGGGATGATTTTAAAAGGTCCTTAGTTGGCATTAATGCAACAAAGGGAAATGCCATTATTGTGACTACTCGAAGTGAACGAGTGGCATCCATAGTAGCAACGCATCATCTTCATTTCTTGGAGAAATTATCAGAAGATGACTGTTGGTCTGTGTTCAAAGAAAGAGCATTTCCAGAAGGAGATGTGCCAATGGAATTGGTACCAATTGGTAAACAAATTGCGCACAAGTGTAGTGGTTTGCCTTTAGCAGCTAACTTGCTTGGAGGAATGTTGCGTCTCACGAAAGAAACCAGTGAGTGGTCATTAGTTTTGAGAAATGGCCTCTGGAACTTAAACGGGGATGAAAATGCAGTCTTGCAAGTGCTGAAGCTGAGTTTTGATCATTTGCCATCAACATCTGTTAAGAAGTGTTTCGCCTATTGTTCAATTTTCAGTCGGGATCATGACATTGAAAAGGATCAACTGGTACAATTATGGATGGCTGAAGGATTTCTCCAACTAAGCCAAGGAGATCATTTGAAAATGGAAAGCTTGGGAAATGAGTTCTTCAACATTTTGTTGCAAAATTCCCTATTACAGGATGTGAAAAGAGATGACTATGGTAACATTACCCATTGTAAGATGCACAGCCATGTTCATGCTCTTGCTCAATCAATTTCGAGATATGAAGGATTTAATATAGGGTGCAGTACTGAAGATGGCCATCCACATGTTCGATATCTTTCAATGAAGTCCTTAAGGGAATCAATGCCTTCAGTTGTCAAGGAAAGAGCAAGATCTTTACGCACCTTGTTTTTAGCAGATAATGTTTCTGGGAGCATGTTATCAAACTTCAAGTATTTGCGTGTATTAAGTTTCCATGGTGTTGACGTTGCTGAGGTACCCTCATCAATCAGCAAGCTAATACATTTAAGATATCTTGATCTATCAGGCACCAAAATTCGAGCTTTAGCAGATTCACTTTGCATGCTTTTTAATCTACAGACTTTAAGATTAAATGGATGTGACTTTCTTGAGAGTATTCCGAGTCAGTTGAGCAAGTTGAAAAACTTGAGACACCTACACTATTATTCATTCGATGCAACTTGTCTAATGCCATTCAAGATGGGACAATTAACTTGTCTGCAAACATTGCAATTCTTCAATGTTGGTTATGCTGATGGTCAGCAAATTGGTGAGATTGGATTTTTGAAGGAACTTGGAGGTGATCTTGAAATAAGAAATCTTGAGAAAGTAACTAACCAGCAAGAAGCTCGAAGTGCAGATCTGTGTAGAAAGGAAAATATTTATAAGTTAATATTTCAATGGAGCAGTGGGAGGCAAGACACAGTCAATGATGATTCTGTCTTGGGAGGCCTTGAACCTCATCCAAATTTGAAAAGCTTGACAGTCCAGAACTTCATGGGTGACAAGTTACCCACATGGATTATGACAATGATGGTCAGTACTATTGAGGGGCACTTACTGGGACTTGACAATCTGGTGGAGATCAAATTAAAAGGCTGTAGAAAGTGTGAAGAACTACCAATGCTTGGGCACCTACCGCATCTCAAATATCTAGACCTCACTGGACTGGATAATTTGAAAACCATAAATCGTTCCTTTTATGGTAGAGACTTTCTCAGATCAAGAACCTATCAAGGTGATAATACGAATATCGCATCATTCAGATCACTAAAGAGACTCGTTTTCTGCAACATGCCCAACTTAGTTGAGTGGACTGAACCAGAAGAGGTGACAACAGAGAAGGTGTTCCCTCACTTGGAGGAAATAGAAATACACAACTGTTCACAATTAACTACCACTCCATGTTCTTTCCCCAGCCTGGAGGAATTGAGGATTTCAAATGTTAGCAGTTACCAGCCGTTGGAAAATATATGCAGTAGTGATAACTCATCAGGTCTTACATTCCTACATATTGATGGGTTGCTGGAGCTTGCTTGCCTTCCGGACAATCTTCTTAACAATGTTAAGAATCTGGTATATCTAGCTATATATAAATGTCCTAATTTGGTGCATGTTGTTCCACGTGTGAGAGGTTTTGGTTCCTTTCTTCGTGTGTTGGATATCAAAGAGTGCACTAATCTTAGCACGTTACCTGATGATCTACAAACTCTTCAGTCTCTTGCAATGCTGTGGATATCTAGATGTCCAAAAATTACGTCAATCCCAAGCTTGGAAGGCCTGACGACTCTGGAAGAACTGAGAATCAGCTACTGTGACGAGTTGGCTTCTCTGCCAAATGAAATGCTGCTGTCCTGCATGTCTCTGAAGTCTTTGAGCATAGAAAACTGTGTTAATTTAACCTCCTTTCCTAATTTGAAACAGTTGCATTCCCTTTTATCCTTGAGAATTGTTGATTGCCCACAGCTGACTTGTCTCCCCAAGGGGCTTCATTCTCTTTCTTGCTTAAACTACTTAAGAATTGGTCCATTTTCAGAGGATCTCACTTCATTTCCGATTCTTGATTACGAAGACGCTCCTAACAGTGAGATACATGAGGAAAACTTGCAACTTTTCTCTCTTAGAAGTTTAACACTTTTTGGGCGACCTCATTGGGACTCTCTGCCTGCATGGCTTCAAAGTCTTTCTTCTTTAGCAGAACTACATCTTTATGATTTTGGATTTGAAGCTGTGCCTGAATGGATAAAAAACATGTCTTCCCTAGAACGATTGGGCCTATACTGGTGTGAAAAAGTGAGTTTTCTTCCCTCCATTGAAGCCACAAAATGCCTTGTCAAACTAAGAGAAGTGGAGATTTACAATTGCCCTTTATTGAGTGAAAGATGCTCTTCTTTGAGTGGCTCTAATTCTGAATGGTCTAAAATTTCTCATATAAACCAAATCAAAGTTGATGGTAAACAAATCACATCCGAGGCTACTTATGAAGTGTTACCATCTCCAACTCTCACAAAAAAAGAAAAAAAACGGAGGAAGAAATAA

>PGSC0003DMG400011809

ATGGCTTATGCAAGTTTGATTTCTCTTAAGACGACGATAAAATCGCTCTTGATGACATCCAATTTGCCAATGCAATCTCTAATATGTGATCACAAAGAAGGAACTTTGGGCTCTTCATTTCTCAACAACTTTGAGAAAAACAATGTTTCCGGGGAAATGACGGATTTGGAAGTACAGGTAAAAGAAGTTGCAAGTGCTGTTGAATACACAATTCAACTGAGAGTAACAGAAATTGAAATGGCAAATACTAAAAGTCAGAACAAAAGGACACGTCGAAACTTTCATCTCAGCCTGCAACAAGTAGCAGAGGACATTGATTGTGTCAGGAAAGAGTCAACAAAGATTCGAGATAAGAATCATCGATTCAAGATTTTTTCAAGTTCAGCAAATGATGTTCTGAAGGTTAAGAACAATATGGTTGGACGCGATGATCAAAGGGAACGGTTGTTAGAAGATCTGACTAGAGGATACTCTGGTGAACCCAAAGTCATCCCAATCATTGGGATGGGAGGCATTGGTAAAACAACTTTAGCAATAGAAGTTTACAATGATGCGCACGTTCGTTCTCATTTTGATGTTTGTGCCTGGGCTACTCTTTCTCAACAGCACAATGTAAAGGAAATCTTGTTGAGCCTTCTGCGCTCTACAAAGGGTGGCACATTTGACATGAACGATGAGGCAAAGTTAGCAGACATGCTGCATAAAAGTTTACATGGAAAGAGGTACTTAATTGTCATGGATGACGAGGGGTTCCTAAATTTTGAAAATGACTTGGAAGGAGAGGCTGAGAAATGTTTACAAGATCTTATCAACAGATGTCTAGTTCATGTCAGCAAGAAAAGTCAAGATGAAACAAAAATTATATCATGTAAGGTTCATGATCTAATATACGACCTGTGCTTGAGACAAGTTCAAAGAGGAAACCTTTTTATCATGAACGACATTCTGTTTGAAAAATCCGATGAAAAGAAAGCTCAAATCAATGATTTTGTATTCGATTGTGATCCAAATCATGTTCCTTCAGTATGCCAATCTCTCAGTAGGCATAATATGCAGCCGTTTAAGCACTGGACCAGTGACAAAATTGATAAATTTCCCTATGGTCTTTACAGGGCCCTTGTTACCCCTGGACATCATCAGTTGAGAGATGATGACAACAACAATCTTTTGAAACGAACTCGCTCTATTTTCTTTTCCGGTCATTATTTTTCAACTTTTATTCTCAAATCAGAGCTTATTCATTTCAAATTACTCAAAATCTTGGACTTGGCACACATGGCGATTTATAGTTTCCCTCTACAGATACTAAGCCTCATCTGGTTGAGGTACCTAGCATTACGCTACCCTGAGAATTTCGACATACCTCCAGAAATTTGCAGGTTATGGAATCTGCAGACATTCATTGTTAGAGGGTATACTCTACCGTATATAACTTTTCCGGAGCAAATTTGGGGACTAATGCAATTAAGGTATCTGAAACTGCAAAAAAATCATTTACCAAATCAACCAAGTGTATCTGTTGACAAAGAAAGACACATGAGTTTTTCAAATATACAATCTATTTCTTACCTATCTCCACTTTGTTGCACGAAGGAAGTTCTTTCGGGGATTCAGAATGTTAAAAAAATAGGATTCAATGGAGATCAAGATTACTATGAAAGTTTTCAGAAATCTGGACATTTCAACAATCTTGTCATTCTAGCTGCAGCAACTGAAACATTGAGTGTAAGAAATATGTATGATTGGAGTCTGGTGACCATTCCAACAACACTCAAGAAGTTGAAGTTGTACAGAACTTATCTAAGTTGGTCGTACTTGGACATCATCGCGGAGTTGCCTAACCTTGAGGTGTTGAAGCTGATGTTTAATGCTTGTCGTGGCGAAGAGTGGGATCCAAATGTTAGTGTATTTACTCAATTGAAGTAA

>PGSC0003DMG400021469

ATGGCTGACATGCTAGCAAATCTAGCTAAAGAAAGATTATGGGAACCTTATAGAGGACTCGAAAAGAAGATAGTGAATCTAAAGGGAAAGATGGAGTTGCTCGGAAGTCGAAAGGTTGATATAGTGGCTACAATAGAAGATGCTGAATTTCATTCATCCAAGAAAAGGAAAGCAGAAGTTCAAACATGGTTAAGCAATGTCAATAACAAACTAGAGGATTTCCAGAGCCTTGAGAAAGAAGTTCAAGAGTGTGGCCGATTTGATCGTATAAAATTGGCAAATTTTGCTGATACAATGATTGAGGAAGCCGAGGAACTCATAAAGCAAGGCAAGTTTCCTGAAGGGGTTTTACATAATGTATATGAAGAAAAAGGGGAGCCTTTGGTGACAACAAATTTAAAAGGCCAAGTATTCAGACAAAATTCGGAAAATATATCTGAAATACTAAGGAACGATGAGGTATCAATCATTGGCATTTATGGCATGGGTGGGGTGGGTAAAACCACTATGGCTATGAATATCCACAACGAGTTCTTACAAGAAAGTAGATTCTTGGGTCACATTTATTGGGTCACTGTATCTCAAGATTCGAGTATTCAAAAGTTACAAAATGGCATAGCCGAGAATGTTGGCTTAGATCTTTCTTGTGTTAATGATGAGATAAAAAGGGCCGCAAAGCTATTCCAAGCATTGAAGAGAATGAATACTTTTGTCCTAATACTCGATGATGTTTGGAATAACTTTGACGTGAAAAAAGTGGGGATTCCACTTGGAAATGATGGAGGCAAAATGATCATAACAAGCCGATCACTTGAAGTATGCCGTAGAGTTGGTTGCCAAAAGAATGTCAAAGTCAATCCTCTTTCTAAAGTAGAAGCTTGGGATTTATTTACTGAGAAACTTGGACATGGCAATAATAATGAAGTACAAGTAATCCCGATTGAAATAGAAAAAATAGCAATGAAAGTGGCAGAGAGATGTGCTGGGCTACCACTTGGAATCATAACAATGGCTGGTTGTATGAAGGGAGTAAATGACATTTTTGAATGGAAGGACGCATTACAAGAGTTGGAAGAATCTTCCATGATGCAAGATGATATGAAGAATGAGGTGTTCCCTATTCTCCATTGTAGTTATACTCGCTTGAGGGATCCCCGGTTACAAAAGTGCTTCTTGTATTGTTGCCTATATCCAGAGGACTTTGAAATTCCAAGAGTTGAGTTAGTCAACAAGTTCATCATGGAGGGATATATAAATGCAAGGAATAGTAGGCAAGCGCAAATAGACCAAGGCCATGCAATACTAAATAAGCTAGAAAATGTATGTTTGTTAGAAAGCACTGAGGATGTAGATGAAAATAAATGTGTTAAGATGCATGATTTGATACGAGAAATGGCTATAAAAATCACCGGCCATCCTCATCATGATCGCTTCATGGTAAAAGCTGGAATGCAATTGAGAAAGATACCAGAGCTTCGTGAATGGTCAGAAGATCTCGGCAAGGTATCCTTGATGCATAATTGTATAGATCAGATTTCTCCTTGTGAACTATACAAATGTTTGGAACTGACAACCCTATTGTTGCAAGAGAATCGCTTGTTGCATGCGATTCCATACTCATTTTTCATGTTCAAGCCTTGTCTACGTGTTTTGGATCTAAGCTATACCAATATTGAAAAATTGCCTGATTCTCTATCAACATTGGAAAATTTGAATGCCTTATTGCTTAAAGGTTGTGGAGAACTTAACTTTGTACCATCACTGAGCAAACTTAAGGTACTTAGCGAACTCGAGCTTACTGGAACAGGGATTAAGCAAGTTCCCGTAGGCATACCTAACTTGGTTAAGTTGAAATGTCTAACCATGAGCGGGTTGAAGAAGCTAAGAAGTAGTGAACCACCCATAGATATGTTTGGAAGCCTATCTCATCTCCAGCGTCTCATGACTCCGTTTTCTATACGAGCAATGGACCTAGAGAGGATGAAACAACTTGAAGAATTTGGGGGTAAGATGTTCAGTCTGTCTGATTTCAACAAATTCGTAGCAAATCGAGAATGTTATGGGCAGCCAATCTTCTTTCGGGTCACTCTAAATGGTTTGACCAGTGATTGTGTTGGAGATTTGTATGAATACCCTGTGATATTTTCCAGCAAAGAAGTCATTTTGAAGGATTACTGCCTGAAGGGTGGAAATGTAGTACAACCCTTGAGGGAACAGTCAGAAGCTGTAATCAATTTTCCAAGGGACATGCAAAGACTTGAAGTTTCATGGTGCGATTTCATAAGTTCGGATAATAGCTTCTTAAGTGCCCTGCCATCCTTGATCAATTTGACAGATGTGAAGATAGTCAAAATTGTTTCTAGTGACGGTATAGAGTGCATACTCCGATTACCCTCCAATTGCCAAGAATTGATTGTGCCAGAAGGACTTGGGTCGTTACTCAAAAGATTGGAGAATTTGGAACTGTACAATTTGAAGGATATTGTTAACCTTATTGACATCCAACCTAATACTGAAGCATCTTTTACGGCTCTTTCACATGGATCTTTTTCAAACTTGAAGAAGTTAAGGATTGAACATTGTTGCCAAATCAAGGTGATGTTTCCTCAATGGTTGTGGAAAAATCTCCACAACCTTGAACATGTTGTGGTGTCATATTGTGAGGGGATAGAAGAAATTATTAGCGAGAATGAGGAAGAGGAAGTGGATCAAGAGGCGAGCAGTCAATACCTGAGTAGTCCTTCATCATTGTTTGCCTCTACCGATGTCATCCTCCCTAAACTAAGAGTAGTTCATTTGACTGAGCTACCGGCACTCAAAAGCATATCTAAGGGAAGAATGACTTGTGTATCCCTTGAGGAAATAGGTCTATGTGGCTGTCCAAAGCTTGAAAGACTGCCATTTTTTCATCCTCTTCGTGATGGAGAACCACTGTTTATTCCACATGCTCTCAGGTCTATATATGTAAGTCGAGAACTTTGGGAATCACTAGAGTGGGACTATCCTCATGCTAAGAGTATGCTTCGACAGAATTTCATACATGATCGATTACCTCAAACTTGTGCCACCAGCTGA

>PGSC0003DMG400021477

ATGGCAGCTTTGAGAGATGAGGATGTCATTGTTATTGGGATTTGTGGTATGGGTGGTGTAGGTAAGACGACACTAGCTGAGAAAATAAGAGCAAGGGCGAAAAAAGAAAGGTTCTTTGATGAAGTTGTCATGGTAACTGGCAGTCAACAACCAGACTTGAAAACAATTCAAGCTGAGATAGCTGGAGGAATCGGTCTAACATTACAAGGCGACAATTTTTGGAATCGTGGAGATCAGTTGCGTTCAAGGTTAGTGGGTCAGGACAGCATCCTTGTAATCTTGGATGATGTCTGGGAGGCTCTTGATCTGAACAAAGGAAGCATGGTCCTTTTCAGACAGAAAGCCGGTAATTCGGTAGCTGATCTTTCTCTTCATCACACAACAAAAGATGTTGTGAAAGAATGCAAGGGGTTGCCGCTTGCAATTATTACAGTTGCAGGAGTGCTAAAGTGTAAAAGCAAGTCTTCATGGGAGGATGCCCTTAAACAATTACAAAAATCCACACCAAAAAATATCCCAGGCGTGATTAAAAATGTGTATCAATCTCTCAAGCTAAGCTATGATCATTTGGAAAGTGATGAAGTCAGGTACTTGTTTTTGCTTTGTTCCTTGTTCAAGGAAGATAGTAATATCTGGCATGAACAATTACTTAGATACGGAATGGGCTTGGCATCTTTTCAGAAATTGAAAATTAGAAGAAGCAAGAAAGAGGGTGTGTTATCTGTTAGAAACATTGATCGTTTCTTGCTATCCCAAGGTTCAGGCAAAAATTATGTCAAAATGCATGATGTGGTCCGAGACGTGGCTATATATATCACCTCTGAGGGAAGGCACGTTTTTATGGTAAGTCACAGTGTGAACTCAGAAGAGTTCCCAAGAAGAACTTCTTACGAGTCATACAGTCACATGTCAATTGTTGCACAAAAAATTGATGAGCTTCCTAAACCAATATTTTTACCAAGACTTGAGTTTCTAATGTTAAAACTATTTGAAGAGCCACTCAAATTACAAGATGATATTTTTGTTGGAATGAGTAAGCTTAATGTCTTAAGCCTAAGTGGATATGAGGACTCCATTTTGCCCTTCCCAAAATCTGTTCAGTTGTTGTCAAATTTGCGGACACTGTCTCTGATTAATTTAAGGTTGGATGACATATCAATTATTGGCAAACTTGTCACTTTAGAGATTCTACTTTTAGAGGTGCTTCCAGTGGAGATAGGAAAATTGACCAATTTAATTTTGTTAGAGTTTTGGAATGAGAGAGTACCACTTGAAAATATTTCACCTGGGGTCTTATCAAGACTAGTTCGATTAGAGGAACTAACATTAGTGGAATGTTCTGGAGATGTAATTCACAGTAACTTGGACCTTTCCTCCAAGTTGACACGGTATTATCTAAATATGGGACAACAAGTTCGTAGTTACCACGATAGTTCACTCATGGATAATTACCACAGGATTATGGTTCTGGATGTCATTGAGACCACCCCATTGGGTGATTGGATCTGCCGCATGTTGAAGAAGAGCGAACTTGTACATTCAAGAGGAAATGGCTCCAAGAATGTGCTGACCGAGTTGCTGGGAGATGGACTACAGAACGTGAAAGATCTCCTCCTAGCTGATTGTGATTCAATGACACATCTCTTGGATATCCACTGTCAGAATAATATTCCATTCCCCAAACTTGAAAGACTGGAGGTAATTAGATTTTGTAGTCTACACTATCTTTTTAGCCCGTCCTTGGTTGTGGGGAGATCGTCAAACTCGACAGTAGCTTGCAGTAATGATGAGGAAGAGGAAATCTCTCAGAGGACACATATTAGATCAGAAGGGAACATGGTCCAAGTAATGAAGTTCCCCAATTTATATTACTTGGACCTTCATTTTCTGGAATGCTTCACTCACTTTTGCAGTGACACTGTTGAGGGCATAGATTTTCCTCGGTTACAAATCTTGCGCTTCTGGGAGTTACCGGAGTTCCAAAATTTCTGGCCTACAGACAACAACTCTATCGCTGACTCAAATCCTCTTTTTGATGAAAAGGTGTGCTTCTTCTATTCTACATGTGACGTATCAGTTTCGTGTCCCAACCTGGAAGAGCTACAACTTAACGGGGCTAACAGCATAGCTGCTCTATGCTCTCCTCAACTTCCAACTGACTACTTCAGCAAACTTAAAATATTGCTACTATGGAACTGTGGCAAATTGAGAAACTTGATGTCTCCATCAGTGGCCAGAAGTGTTCTGAATCTCCAAATTCTAAGCGTAGAAGCCTGCCAATCAATGGAAGAAGTGATCAAAGAAGAGGAACTAGTAGAAGAAAGGACTATTAAGCCCTTATTTCCCCGGTTAGAAAAGTTGGTGCTCGAAGAGCTACCAAAGCTGGGGCATTTCTTTTTGACAAAGCATGCTCTTGAATTTCCATTTCTCGGAGAAGTGAGGATTAACAGCTGCCCTGAAATGAAGACGTTTTCCCTTGGATCTGTGAGTACACATAGTCTGGACAGGCTGATTGTGGACTATGCTGAGGTGAAAGATGATCTCAATAAAGCGATACAACAGCTCTTCAATTTTGAAGGTTTGTCTTGTACTCCGTCGTATAGCTAA

>PGSC0003DMG400012650

AGGACTGAGTTGAGATTTATAGAGAAACTTGTGAAAGTTACTGAAAATAAAGTAAACCATTCAATCCTGAGTGTTGCTCCTTACCTAATTGGTATGCATTTTCGGGTTAACAATATTTGCTTGTGGTTGCAACATAAAGCAGATGATGTTGGTATTTTGGGCATTTGTGGTATGGGTGGTATAGGGAAGACAACAATCGCCAAATATGCATTTAACTCTAACTTTGAAAGTTTTGAAAGAAGCAGCTTCCTCTACAATGTTAGAGATTTTTCCGAGTCTACGGATGGACTAATTTATCTGCAGAAACAATTTCTTTCTGATATTCTTGATGGGAGGAAGATTGACATTCAGAGTGTTGAAGATGGAATCAATCAGATCAAAAGTGCTGTTTATGGTAAGAGGGTTCTTATTGTGCTGGATGATGTTGATGAAGTGGACCAATTAGCCGCTATTACTGGAATGCGAGATTGCTTTTATTCAGGTAGTAAAATTATAGTGACAACTAGGCACATAGAGTTACTAAGGGCTTGTGAAATTGAATTGATTGATCATGTTCAGAAACTGAGCATAGGCGAATCTATTGAACTATTTAGTTGGCATGCATTCGGACAAGACCATCCAGTTGAGAATTACACCAAGTTCTTGAGAAGGATTATAGAATATTGTATTGGATGTCCTCTAGCTTTACAAGTTTTGGGTTCTTCTCTCTTTGGTAAAAGTTTAGCTGTATGGGAAAGTACACTGAAGAAACTAGAGGCAATTCCTAATAGTAAAGTATCAAAGAAACTGCAAATTAGTTTTGAGTTTATACAAGATGATCATGACAAAAGCCTATTCCTTGACATTGCCTGCTTCTTTCTTGGAAAGAATGTAGACCACGTTGTCACTATACTTGATGCATGTGGATATTATTCAATGGTTGGGATTCAGAATCTCCAAGATAGATTCTTGTTGATAATTGATGAATATGGAAAACTGAGGATGCATCCATTAGTTAGAGATATGGGACGAGAAATTATACGCCAAGAATCACCTAAGCATCCTGAAAGACGGAGTAGGTTGTGGCATTTCAAGGACTCATTTAATGTTCTGAAAGAAAAAATTGTAAGATTCATTATTTTTGCTGTTACATTTACTTTCGCAAATCTAGTTTTATGTTATATCTATTAGTAATCTTTTCATCAGGGTTCAGATATGATTCAAGGCCTGAACGCTAGGTCTTGTGCACACAAAGATAAGCCACCAAGATTCTTTTATCATCCAACTGCAGATGAGTACTTGCAGGAATGTGCCTACCTAATGGGGAGGAAAAAGCTGTCAAGCCAAAATAATCCATCAAGGCAACCACAACCTCGTAAAGTTCTTATTTATGGTTCCTCGATAGAATCACTTGGCAAATCAAAGAAGGTGGACAGCGTCAACACTGATGCATTTTTTGGTATGCACAAATTAAAACTGCTGCAACTTGACAATTTAGCAGTTAAAGGAAATTACAAGGAGTTCCCAAGGTCATTGAGGTGGTTGTGTTGGCACAAATTTCCATATAAATGTTTACCTGATGGCTTACCTTTGGAGAAACTGGTGGTTCTGGAAATGCGCTACAGTAGGTTGCATCATCTTTTTGAAAGAAACAAGGTAAATATGGCCCTCTCTCTTAAGCTTGTCAGTCAGATTATGAATTTTTTTGTTTTTGTTTTACAGGTTCTCAGTGCATTAAAGATTCTGAATTTGAGTCATTCAGAAGGTCTGTCCTACACCCCTGACTTCTCAAAGCTCCCAAATCTTGAGAGGATAATTCTCAAGTACTGCACTAGGTTGACTCAGATTGATAAATCGATTGGGGGACTAAAGAGACTCCTCATTTTGAACCTGAGAGGCTGCCAAAGTCTACGGAAACTTCCCAGATGTATTATTGATGTCCATTCCCTCGAGAAGCTTATTCTGTATGGTTGCTCAAAATTTGTATGGTCTTCATTAGAGCTGGGTAAGATGCATTCATTGCTAGAGCTTGATGCCGGTGGAACTGCTATCCATCAAGTTTCCACTTCTGTGTGTAAGAAGCATTTACTGAGTTTAGCTCTGTGCACTTCAGTCTCAAGCCCGAGAAAATCTCCTGGAATTGTTAACCTGTTAACTACAGTATCTCAGACATTAGTAACCTTAAGTCTTATTGGATGTGGTTTATCATGTGATTTGATTCCAGTGGAGATTGGTGATTTCTCCACACTGCAGAACTTGTTTTTAAGTAAGAATCCAATTCACAGCTTACCAGATAGCATCAAGAGGCTTACCAATCTCCAGGTACTTGAGTTAGATAAATGCGAAGAACTTAAGTATCTTCCAAAGATCCCAGCCAGTGTCACAACCTTGAGCATCCACGGATGCAGGTCACTCGAGCGGTTATCAAATTTACCAAACATATTGACAACATTGGATTTTCTTGCATTAAGATGCAACAAACTAATTGAGGTTCAGGAAATGTTCCAGCTAAAATGCATTTCTTTATTTGATGCTGATTTAATCAGCATTCTGGGATTACCCAACTTGAGTGAAACTAAGGTGGATCTGTACAACAACCTGACTCTTACAAGATGGAAATGTCCTATACAGGTTTGCTCCCTAATATAA

>PGSC0003DMG400021098

ATGGCTGAAGCTGCAATCATATTCTTTCTGAGAAGGCTAGGCTATCAGTTAATACAAGAAGGAAATGTTCTATCAGGTGTGCAAGATGAAATTGAGTGGATGAAGAAAGAGTTTGAAGCCATGGTAGCTTTTCTAAAAGATGCTGACAAGAGGCAACAGAGAGATGAAACTGTGGCTGGTTGGGTAAAAGAGGTAAGAATCTTGGCTTTTAATGCTGAAGATGTTATTGATGAATTTCTTATTCAAATGGCTGCTACTCATTGGAATAGCTTATACTTCTTTAAGTACTTGAAAATAAGGTATCAAATTGGCTCTCATATTAGAAAACTCAAGAAACAAGTCATTGAAGTCAAAGAAAGAAAAGATAGATATGTTATCAATGGATTAATGATGTGTGAAGATGCATTAGCAGCTAGTAGCTATAGAGGCACTGGTGGGATGTCCTCTAGAGGGCCTGGTGCAGCATCCCCTTTTGTTCGAGAAGACGATATTGTGGGAATTGAACATGATGTTGAACAGCTAATGAAACTCGTATTAGAAGGGAATGTGAAGAATTTCCTCGCGGTTTCAGTGTTTGGTATGGGGGGTTTGGGCAAAACTACTCTTGTGAAAGAAGTTTTCAAGAAGTCAAAAGCGTTGTTTGATTGCCATTCTTGGGTTTTCGTGTCACAATCTTGTAATTTAAAGGATGTCTTGAAACACATCCTCTTTGGATTCATAGCTAGTAGAGGAGAGCCAGCTCTGGATGTAATGGGTGCCATGGATGAGGGATGGTTACTGGAGAGGATCAATGACTACTTGCAAGACAAGAAGTACTTGCTGGTTCTTGATGATATATGGGATGATAACCTATGGGAAGAGCTTAAACATGCATTTCCACGAAGAAAAGGGCGAATCATAATTACAACAAGACTTCGCGGTATAGCATCTCCTCTTGAGGATAATTTCCACATCTATGATCTCCAACCCCTCCCTTATGAATTATCTTGGAGAATCTTTTGCAAGAAGGCGTTTCGATCATCTCAGGGGACTTGTCCTGATGACTTGAAGGAATTCGCGGAGGCCATAGTGAGAAAATGTGGAGGTTTGCCTCTTGCAATTGTGGCCATCGGAGGCTTATTGTCGTGTAAGGGAAGAAATACTCGTGTGTGGCAATCTGTTCTTGATACCTTAGATTGGGAATTCAACCATAACCGAGACATTGAGCGTCTAAACAAGGCCTTGTTGTTCAGCTATATTCACCTTCCATTTTACCTCAAGTATTGTTTCCTTTATCTCGGTTTGTTCCCTGAGGACTATGAGATTGGAAGGAAAAGGCTGATCCGAATGTGGGTGGCCGAGGGGTTTGTTGAGGGAACTGCTCAGAGAACAGAGGAAGAGGTCGCGAACCATTACTTTGTGCAGCTTACTGACAGGAGTATGATTCAAGCAGTCACTATACATGCTCGAGATGTGGTGAAAGCGTGCAAGCTGCATGATCTAGTGCGTGATGTGGCCAATCAAATGCTACAGGAAGAGAAATTTGGATCCATCATAGAAGAAGTCGACAAGACCATTCAAGAGAGACAAAGGAGACTTGCAATCTATGAAGATGCAGATAGTATCCCCTCAGACATCAGTAAGTTGAATCTGAGGTCCCTTCTCTTTTTTAGAATAAATGAATTGTCATTTTCTGCTCTACAAAAACTGCTAAGGCAGCTCAGATTGGTAAGAGTTGTAGATTTACAATATGCTCCTCTAGAAAAACTACCAAATGAGATTGGTAATTTGATTCATCTCCGGTACTTAGATTTAAGAGGGACTTTGATAAACGAACTTCCAAAATCAGTGAAGAATTTGAGGAATCTACAGACATTGGATGTGAGAAACACGGAAGTTAAACATTTGCCTGCTGGAATCAATGAGCTGCAACACTTAAGACATCTTCTCCTGTCAAGTTTCCGTGACAGAGAAAATGGATTTGTGAAGATGGCTAGTGGGGGAAAAGACTTTGTGAAGCTACAAACACTATCTGGAATTGAATCAGATGAAGACTTAGTTAAACAACTTAGAAGTTTAACAAGCTTGAGGAAAGTCTACATAGGGAAAATGACACAAGCCAACAGCGGAGATTTTTGCCAATCATTAGAAAGGATGAGCAAGTTGAGGTCCTTAACAGTGTTAAGTGAGAGTCCATTTGAGCAAAACATACAAATGGAATCGCTCACAAAGTCCACTAAGCACTTAGAGAAACTCAAACTCCAAGTCCATATGAAAAAACTGCCAGGATGGTTCGATTCTCTTAGTTGCCTTCACTCATTGTACCTTTTCAAGAACTTCTTAACAGAAGATCCATTTCCTATACTTGGAAAGCTTCCAAGCCTAGCTATTCTGACATTAGCATCTTCAGCTTACGTAAACAGCATCGTTAACATCCCTCCGGGAGGATTTCCTAAGCTCAAACTGTTGAGGATTCTTGGCATGGAAAATTGGACTACTTGGATGCCTATTGAGAAGGGTTCTATGCCAGAAATTCAGTTTCTTCTGATAGCTAATTGTCCAAGGCTAACTAACTTACCAGATGGCTTCAATCACCTTACTAGCCTTGATGATCTCACCCTTATGGGCATGTCCCTCTTTTTCGCTCATAAATTGCAGAGTAGAGATAAGTGGAAAGTCACTCATGTCAAGGAAGTTTCAATCATTTCAGAAGTCAATGGCCAAATTGTCAAGAAAAAACTCAACACTCCTCTTGTTAACTAG

>PGSC0003DMG400021169

ATGTTACCTATCACAATTGAGTTGTCATCAAAGTCAATGGTGATCTTTGAGGAAGAGGAACAACTGCTGATGGAGAATATTCTTGGTGGAACAGATCAACTAGGCGTTATCGCGATTGTTGGTATGCCAGGACATGGTAAGACAACTCTTGCCAAAAAGTTATATCACCACGAAAACATAGTCAATCGATTTGATGTTCGTTCATGGTGTACTTGTGTCTCTAAAGTATATGACAGGAGAAAAATATTGCTTGAACTATTAGGCTATGAGTTTGAACGCCGCGTTGAAATTGAGATGAGTGAAGATGAATTAATCCAACAAGTACAAAGGTGTTTTGAGAAAAGGAGATATTTCATAGTTATAGATGATGTATGGAGCACTGCTATATATGATGAGTTAGTAAGTTTTTTCCCAAATAACAACAGCAGGAGTAGAATTATCATAACTACTAGGCTTGATGAACTGGTTAACTCATCTGTAAATTACTCGTTTTGCTATACTCATCGTCTTTCTTTTATCGTGGAAGATAAAAGTTGGTTGCTACTAAGGAACATAGTTTTTAAGGAAGAAAATAGCTGTTCTCAAGAACTTGAGGAAATAGGGAAGCAAATAGCGAAAAGTTGTGCAGGGCTGCCTCTAGCAATTGTATTAATAGGGGGTCTTCTTGCAAGATTGGATAAGAAAAAAGGCTATTGGACTAAAGTTGCTCAATGGTTAAGTGCAAGTGCAAAAGTTGCTGGTGAGGCAGAGTGGTACATGGACATAATAGAGTTGAGTTACAAGCATTTGCCTAGTTATTTGAAGCTGTGTTTTCGTTACTTTGGTATGTTTCTTGAGGATGAGGAAGTTTCAGTTAAGAAGTTGATGAGGATATGGGCTGCTGAAGGTTATGTACAGAATAATGAGGTGAGGAGTGCAGAGATTGTCCCGATGGACTATTTGATTGATCTAATAACAAGCAATCTTGTCGTGGTTGCGGAGAGATTTCCACTTGGTGATATGAAATCAGTTTGTCTTCATGATTTGGTGCAAGATTTTTGCTTGAATAAAGCTAAAGAAGAGAACTTTTCGCTGAAAGTTGATAGGTGCTCAAATTTTGTGAGGAATTGTGACAGAGTTCCATCACAAATAGTGTTTTTTCTTGTAGTGATGTATATATTATGTCTAGTATCTCATTATTCTTCGTTTTCTTTCAGATCTCATGCTTTTGATCCTGCTACTTCTAATTCAGCTAGTGATGTTCAACGGTTCCTGGTTTGCTCGAAAATGCAGCACTTCATCAAGTGGCTTCATCCGACACAACGTGTCCATACTTTGAGGTTACATGCCCACAACATTAATGAATTTTACAATTTAACTGACGGTGAAACGAACAAATTCATTCTACCATGTGGAGCATTCACTTCAGACTTGTTCAAATTATCACTTACAGTACTGGACTTAAGGAACACTGTGATCGATGCTTCAGCTCTGGAGGACATAACATCCTTGATTCACTTGAGGTACTTGTCAATATTCGGAAATTTTAGTGAGATTCCACCAGCCATATCGAACCTTACAAATCTGGAAACGTTTGTTGCAAGACCAAAAAGTGGTACCTTAACTCTTCCAGGGTCTATCTGGGACATGATAAACTTAAAGCACGTGGATACAAGCGAAGCAATGATTTTCAACAGTACTGTTGAGGTTGAAAAAGAGGGTGTTTTCGAGTTAGAGAAATTAGAAAGCTTTTCGAAAGTAGTCATAGTGAATGGAGATGATATATGTGAAATGTGCAACAAAGCACCAAACTTAGTCCAACTTGAACTTATTGTGGAGCAATGGGACAATTTGTTTAGCTCTCTTAACTGTTTGATATATCTCGAAACACTAAGTATCTATCACCGTGGCGATTTTCCAATGAGTGGAACTAACATGATGTTCCCTCAATGCCTAAAAGAGTTAACTCTGTCTAGTTGTGGTTTCTCATGGGATGAAATTTCAACAATTGGCACATTACACAACTTGGAAGTGCTTAATATACTTCTTTCTGCCTTCACTGGGAAAAAATGGACAGTTGGTGTTGGTGGTTTCCTTAACCTTAGGCTATTGAAGTTAGAACATAGTTCTATCAAACATTGGAACATGTCTGTTGATTCATTCCCCTGTCTCGAGCAACTGGTGTTGCGTTGGTGTGGTGAACTTGAGGAGATCCCCGCGAGTTTTGGAAGCATGCCTTCGTTGCAGAAGATTGAGGCGCGGGCATGTTGTCCCTCAGCCAGGAAATCTGCTGTGAAAATCAGGAACATGCAGAGGGATGACATGAAAAATTTAGACTTCAAAGTCATCATATATTTAGATTAG

>PGSC0003DMG400008306

ATGGCTGAAGCAGCAGTTTCCTATGTTGCAGAGCGGCTTATTGATCTTCTCCAGAAAAAAATTGTTTTCCTGAAAAACATCCAACAAGGAGTTGAAGCAATGCAAGATGAGCTTGTCAGTATGAAGTGTTTCCTGAAAGATGCAGATATGAAGCAAGAGGAAGATGAGGCCGCAACAATCCGCAATTGGGTATCTGAAATCAGAGCAGTTGCCTACCATGCTGAGGATGTGATTGAAATATTCATCCACCAAGTTGAGTCCCAAACACGACAGTGTTTCTTCATCAAGTGTGTCTTCTATCCTAAGAAACTATACTGTCTTTACAAAGTAGGGAAGGAGATTGAATCGATTCAGACCAGAATTCTTGAAATATCCAACCGTCGTGAAAGATACGACATCAGGCATATTCGAGATGGGGAAGAGTCAAGCACAACACATGAGAAGCTGTGTGAACTAAGACGCTCCTCGCCCTTGGTTGCCAACAAGGATTCTGTTGGTCTAGAGAAGCATGTGAGCTCTGTCGTGTCAATCCTTCTGATGGAGGACAAACGGCTTCGTGTTGCTTCAATTGTTGGTATGGGGGGCGTCGGCAAGACGACTCTTGCCAAGGAAGTTTATAACCGAACTCAAATCAGAGACAAGTTTGACACTCGGGCATGGCTTTATGTATCCCAAGATCATAAGCCCATGAAGATCATCAAGGAACTCATTTTACAACTGGCAAATCCAGAAGAAGATAAAGTGAAGATAGTGGACACGATGGACAAATTGTCAAAGGCCGGTTTGGATGAAATGCTCCAGAGGCGTTTGAAAGATACATGCTATCTCATTGTTCTTGATGACATCTGGACCACAGAAGCATGGGATCTCATTGTTATGTCATTTCCTGACAATGGTAAGTCAAGTAGATTGCTACTTACCAGCCGCAGTAAGGAGGTTGCCTTGCATGCAGATGCTCATACTACACTCTATGAACATAAAGTATTGAGTAAAGAGGAGAGCTGGGAACTCTTTCTCAAGAAAGTATTTGCTGTGGATAGAGAGTGTCCACATGACTTGGTGGACGTGGGAAAAGAGATTCTGGAAAAATGTGATGGGTTGCCACTGGCAATCACCGTCATAGGAGGTCTCTTGGCGGGGAAGAAGAAGCAAAGAAGTGAGTGGCAGAGAGTACAGCGAGGCCTCAGTTCATACCTTGTCAAACCTCAAACTTATGGCGTATCAACAATTTTGGCATTGAGTTATCAGGACTTACCTCCCCATTTAAAATCTTGCTTTCTCTACTTAGGCCTTCTCCAAAAAGGCAGAGACATCCCTGTGAAGCAACTCATGCATATATGGATTGCCCATGGTCTTATTCATCAAAAGGGAGAACAAACATTAGAGGACATTGTGGAGGATTATCTTGATGAGCTCATTGGCAGAAATATGCTTCAAGTAATCCTAGTTACAGCAGATGACAGAGTTAAGAGTTGCCGGCTCCATGATCTCCTACGAGACTTTTGTATGAGGAAGGCCAAGGAAGAAATGTTTCTGGAGGTAGATAATCCATCTATATCCCTCTCTGGATCTCGGCACCGTGTCCTTTACTCTCCAGTAAAGAGGTACAAATATTTGGGAAATTCAAAATCATATATAAGATCTCTTCTATCCTTTGATTCATCTGCTAAACTAGTTAACCTTGATTGTATTTGTACAAGTAGTTTCAAACTTCTCAGAGTGCTATACATAGACTCTCCTGGTTTAAAGGTGATCTCAGATAGTATTGGGAAACTATGTTCCTTGAAGTACTTAGGCATAGGATGGAAGACATGTATTAAGAAGTTTCCCCATTCGATAAGTCGTTTGCATAACTTGGAAACAATAGATATGCCCATGTCTAGGTTTTATTCTGTGAAAGTTCCTAATGTCTTGTGGAAGTTAGAGAATTTACGGCATATACTTGGTTACATAAATTCCCCCCGCCTATTGAAGATTGACCTACCTAAAAATCTCCAAACTCTAGGCAGCATACCTGTTCATCACTGGATGCACCATGAAAACCTGACAAGAATGACTCATCTCCAAAAAGTTGGTTTACTCATTGGAAGTGCTGATAACCTAGACATGAATCGATTATGTGATTCACTTGCAGAGCTTGAGAGCCTTCAATCCTTGTGTTTGGAAGTAGAGGGTAGCATGAAAATACCTTTGGTTGCTGGTTTATCTAAGTTGAGTCATGTTGTCAAGCTTAAGTTGAAGGGACGCTTAGCATTAATACCAATTAATTCTTGTGTATTTCCATCTAAGCTTTGTCAGCTGACTTTGGTGAACTCGAACCTCCATCCATATTCAATCCAATTATTGGAGAAGTTAACCAACTTGTCAGTTCTAAAACTAGTTAAAGCCTTTTATCATTATGATTGGTATCAAGAGTATAGGATAAGAATATCGGAGAATGGGTTTCGTGGACTCAAGTTTCTCAGGATGGATCAACTAATGTATATGAAAGCAATGAATCTAGGGAAAGGTGCAATGGCTGGACTCAAATGCCTGCAAATCTTCAAATGCTACTCGTTGACGAGGCTCCCAGGAGAGTTGATATCATTGTCTAACCTTGAGAAGCTGGAGATTCGAGGAATGCCTAAAGCATTTATTGCTAGACTTCAAGTTTCAGATTTGCACAAACTCCAACACATTCCCAACGTTGTGGTTGGACATCCAAGTACAGATTATGAAGAATGGATCCAACAACTGAAGCGTAGAGATTATATGGGTACAAGTACAACAACTATGGAAAGAAGGGCCAAGAAAATGGCACAGGAAATTTTTAAAGAACATTTCCCTGAGGAGTCATTTTGGTTGATTCCTTAG

>PGSC0003DMG400008292

ATGTCGGATGGTCCTAATGCTGAAATGTCTTGCATATCATATGAGGTTCATGATCTGGTTCACTCACTTTTTCATCAGAGTAGTGGAGATGACATGATGGTTAAATTAACGGATCATATCGTTCCTCGCCTTCTTGAAAATATCACAAGTTCTAAAATCTCACATCATCATCATTCTGCCGCCATGACTGAGAATCAGTTGGTTGAACTTTTGGACGTGCTCCTCCTGAATCTCCATTATCTACCAAAGGTTCGTGCTGAGTTGATTTCCCCATCAATGACTCAATGTGAGCTTCTTCAAAATGTATTTGGCAATTTAAGAGATTTCCATAGGTTGAAAGTAAATGGTTGTGTTGAGTATGAGACAATCGAATATGTCTTACCGCAGTTTCAACTTATGGCGGAGAGAGTAGGACACTTCTGTTTTGTCCTTTTGTCTTATCAACTTGATAAAACAAATGAAAAAGTCTCTCAAGTCAATTCCATGCTAGTCCATCTACTTTTGAAGATAATTCCGGTTTCACTGGAGGTTATGCACATATGTTGTACAAATTTGAAAGCTTCAAAGTCGGAAGAAGTTGGATGCTTCATTAAGCAGCTCCTAGAAGCCGCTCCAGACTTTCTTATAGAATATCTGATTCATCTACAAGAGCACGTGATTAATGCTATTACTCCTAGAACCTCAGCTCGAAACATTCATGTCATGATAGAGTTCCTATTTATTGTTCTTTATGATGTGCCCAAACACATTATTCATCATGACAAATTATTTGTTCTCTTGGCACGTGTTGGGGCACTTATCAAGGAGGTATCCATTCTTGTTCGCAACTTAGAGGAGAAATCAACGAGTGAAGACAATATGAATCCAACAAGCTGTGCAGGTCTAAACTTGCTGGAAAATATTGAACTCCTGAAGGTGGATCTCAAGAATGATTTCTTAAAAGCCCGTGCAGACTCGTCTCTGCTCCATTTCCCCATGAGTGATGGACCGCTCTTCATGACTCTTCTACTCACAAACTTAAATGACTTGGTCAATTCCAATGCTTATTCAGTTTCTTTGATAAAAGAAGAAATCGGGGAGATGAAGGAAGACCTGGAAATCATAAGATCGTTCTTTGGGTATGTTGAGCAAGAGCTGCATAGAGATCTTTGGTACCCGTTTTCTAGATGTGGCATAAGGCACAACAATGGCATTAACTCAATTCTTGCCCGAGATCATGGTCTATTGCAGCTTATCTTCATACTTCCTGATACCGTAGAAAAGATCAAGCTTGTCAAAAAAGAGGTACACGAGAAGATCCCCAAGAGCAGCGGTATTATTGTTGCAAACGCTCCCAACAAGTCGGTTGAAAGAAACTCATCGAGTACAGTTGGTCAAATAATTGTAGGTTTTGAGGAGGAGACAGAGTGGATAATTAGGAAGCTCACCAGCGGACCAGCTGAGATAGATGTCATTTCCATAGTCGGCATGCCAGGGCTTGGAAAAACTACTTTGGCTTACAGAGTGTATAATGATAAGTCCATTGTTGATCATTTCGACGTTCGTGCTTGGTGCACAGTCGACCAGGAACGTAATGAGAAAAAGTTGTTGCAAAAAATCTTCAATCAAGTTATTGGTTTGAAAGAAAGAGTCGGTGAGAATGACATAGATGATGACGTTGCTGATAAGCTGCGGAAAAAACTATTAGCAAAACGGTACCTTATTGTCTTGGATGACATGTGGGATACTGCAACATTGGATGAGCTAATGAGACCTTTTCCTGAATTTCAGAAAGGAAGCAGAGTGATTTTAACAAGTCGGAAAGAGGAAGTTGCTTTGCACAGAAAATGCCACAGTGATCCTCTTTATCTTCAATTGCTAAGACCTAAACAAAGTTGGGAGTTATTAGAGAAAAGTGTATTTGGAGAAGAACGTTGCCCTGATGAACTAAAGGATGTTGGAGAAAAGATAGCTCGAAAGTGTAATGGGCTTCCTTTAGTACTTGATCTAATCGGTGGAGTCATTTCAAAGAAGGAAAAGAAAGAGGCTTTGTGGCTTGAAGTTCTCAATAATTTGAGTTCCTTTATTTTTAAGGATGAAGAGGAAGTGGTGAAGATTATACAATTAAGTTATGACCAGTTGTCAGATCATGTAAAGCCGTGTTTGGTTTACCTTGCAAGTTATCCAAAGGACGAAGATATAAGGATCTCTGAGTTGAAAGATTTTTGGATTGGTGAAGGACTTGTGGAGCAGATTGAGATGAAAAGTGCAGAAGAAGTAGTGAATGAGTTAATTTCCAGTAGCTTGGTAATACCTTTCGATAATTCTATTTGCAAAATTCATGATCTTGTGCATGACTTTTGTTCTATAAAATCTAGAAAGGAAAAGTTTTTTGACTTCATAGGAGGTCCAAACGCTTCGTCTTCTTCTGGTATGATGGCACGAGGAATTACCATTTGTTATGATCAACGTCTCTTTCATTTGGATGAAAATTTTGTGGTGTTTAATCCAGAAAAGAAGAATCCTTATGTTAAACACCTCCTCTCTCTGAAGGTCTATGATGGGTTTAAGGGTTGTCTTTCCTACAAGAGTCACCTAAAACACTTGAGGCTTCTCAAAAGTTTGGATTTGAAGGACATAACATTGACAGATTCTTTGCTGAATGAAATCGGTATGCTCGTTCATTTGAAGTACTTACTCATTCAGACGAAGGCTAACGCTCTCCCTCCGTCATTTTCAAACCTCTGCAATCTAGAAACTCTATCGGTGTATAACATAGAGGAATCATGCATGGTACTATCGCCTTGGTTTTGGAGTCTAGCAAAGCTACGAGATGTGCGGATGAAGTACGGTGCTCTTTTTGATCAAACTGTGTTAGACGAGGACTCAAGGTTAGAAAATTTGAGAACATTATCTCTGCTCTACCTTCCCGGTTTAGAAGACACAGAGGATATTATTTTCAAAAGGTTTCCTAAGCTTCAAAACCTTGAAGTCTGTATCGCACAACCAGAAGATTGTACAGCAGAGAAGATTTGTTTTCCAAGATTGGATGTCCTTAACGAACTTGAACAACTTCATGTATCTGCTTTTTGTGATTCATTCAGAGAAAATACATACTGCTTCCCTTTGAGCTTGAAGACACTGAAGTTGAAAGGGATCGCTCTGACATCCGATACACTATCAAGAATTGCTAGACTACCCAACCTTCAAGAACTAATTCTAAATTTCACAATCATCGAGGAAAGGAAAGAATGGAACATGGAAGATGTCACCTTCCAGAATCTCAAATCTCTAAAATTGGATTGTGTGTTATTTTCTGAATGGCAGGTTGATGCAGAGAATTCCTTTCCCGTGCTCGAGAACCTACATATAACTACATTGGATGAGCTTATGGAGATCCCAGACAGTTTTGGAGATATTGCGTCATTAGAACTTATCAAAGTATGGGACTGCCCTCAGCTTAAAGAGTCGATCTTCAATATTAAGGAATATGTTAAAGAAATGACCGGAGAAGACAAGCTTGAGGTATACTTCTATAATTGA

>PGSC0003DMG400013627

ATGGACAATTCTCACAGTATTCGTCACGAAATTACATCTCATGATCCACATTGGTCGTATGACGTTTTCTTGAGTTTTAGAGGTGAGGATACTCGAAAGAGCTTTGTTGATCACCTCTATACTAGTTTACGCGAAAAAGGAATACACACATTTCGTGATGATAAAGAGTTGAGTAGAGGAAAATCAATTTCCCCTGAACTCCTTAACGCTATAGAAAAATCGAGGTTTGCAGTTGTTATTTTTTCAAAAAACTATGCAGATTCATCGTGGTGTTTGGAGGAGCTGACGAAGATCGTTGAATGCAACCAACAGAGGGGACAAACGCTGATTCCAGTGTTTTATAGTGTGGATCCTTCTGTTGTGAGAAAACAGAAGGAGAGTTATGGAGACGCTTTCGCTAAACATGAAGAGAATTTGAAGGGATCAGATGAGAGGAATAAGATCCAAAGATGGAGGGATGCTTTAAAGGATGCTGCTAACATCTCTGGATTTGACGTCCAACACATGGAAGATGGGCATTTTGGATTTGCAAATCTGATAGTACTATTTCTTTATCTTTCTTTTAGGCATGAATCAAGGTGCATTAGACAGATTGCTTTGACAATTTTAAAAAGATTGGGGCGTGTACGACCTAAAGTTGCAGATCATTTAGTGGGCATAGATCCCCATGTACAAAATGTTATATCTATGATGAATTTGCATTCTGAAGCTGATGTTCGCATAATTGGGATATGGGGTATGGGAGGCATTGGCAAATCAACTATTGCGCGAGCTGTTTTTGATCAACTTCAGGAAGAGTTCGAAGGTAGTTGCTTTCTTGATAATGTTAGAGAAGTTTCAACAAAATCCGGACTTCAGCCTTTGTCAGAAAAAATGATTTCTGATACATTGAAAGAGAGTAAAGACAATCTTTACACCAGCACCACTTTGCTGATGAACAGGTTGAGCTATAAAAGAGTGATGGTCGTTCTTGACGATGTGGATAATGATGAGCAGATAGACTATTTGGCTGGAAAGCATGAATGGTTCGGAGCTGGGAGCAGAATCATCATCACCACTAGAAACAGGCAATTGTTATTGTCTCATGGGGTGGATCATGTGTATGAAGTTAGTCCTTTAGGGATTAATGAAGCTTTGATGCTCTTCAACAAGTTTGCATTCAAAGGACGTGAGCCGGAAGGTGATTTTTCTGAACTGGCGCTGCAAGTCGCGCAATGTGCTTGGGGCCTTCCACTGGCTCTCAAAGTATTGGGTTCATTTCTACATAAAAGAACAAAAGCAGAATGGAAGAGCGAGTTGAAGAGGCTGAAAGAAATTCCCCATGATGATGTGATAGGGAAACTTAAATTGAGCATTGATGCATTGAGTGATCTAGACAAGCAAATATTGCTTGACATTGCTTGTTTCTTCAAGGCGAAGCGAAGAGAACCTGTCACCAGAAAACTCCTTGCTTTCGGTTTCAAACCTGAAATTGGAGTACCAGTACTTATTCAAAGATCACTTTTATCCATATCTGATGATGACAGGTTCCAGATGCATGATTTAGTTCAAGAAACCGCTTGGTACATGGTTCGCCATGGACATCCTAGAGAGAAATTCAGCAGGTTGTGGGTTCCTGACGATATATGCGATGTTATGTCAAAGAAATCAGTAAGTTTACACTACATGGTTGATATATTGAACAACAACTTCATGGCCAAATTTCACAACTTTGGCCAGAAGATAAGGTACCATTGCCCTCATAAATATTGTTCGTACATATTTCTTCTTTGTCCGAGTGACCACTATTGTAAAATAATTTACAAACAGATATCTACCTTACATTGAGTGTTCATTTTTCTCTTGCAATTAGGCAAAATAAGTCACTTATTATATGTTTCATGATTTAAACAGTATCTTGACAAGCTGAAGTATTTAAACCTCAGCTACTCCAAAGGGTTAATTAGCACTCCCAACTTTAGCCAAATGCCATATCTTGAAAAGTTGAACCTGAGCAACTGTACGAACTTGGTAGGAGTTCACAGATCACTTGGAGATCTTACAAGGCTTCGGTACTTGAATTTGTCTCACTGCTCAAAGCTAAAGAGTATTTCGAATAACATCCATCTCGAGTCTTTGGAAAAGCTTCTTCTGTGGGACTGCACAAAACTCGAAAGTTTCCCTCAAATCATAGGATTAATGCCAAAGCTTTCAGAACTTCACTTGGAAGGGACTGCAATCAAAGAGCTACCCGAGTCCATCATAAATCTCGGTGGGATTGTGTCCATAAACCTCAGGAATTGCAAGGATTTGGAATGTATAACTTATAGCATTTGTGGTTTGAGATGTCTCAGGACTCTAAATCTATCTGGCTGTTCAAAACTTGAGACATTGCCAGAAACCCTTGGACAAGTGGAAACTTTAGAAGAACTACTTGTAGATGGAACTGCAATTAGCAAGCTACCATCAACCGTCTCGGAGATGGAAAACTTGAAAATCCTTTCTTTCAGTGGATGCAAGAAAAGGAAAAAAGATAAGGCGTTCTGGAAAAACAGTTTTAGCTTTCGCTTGAATCTAAAGTTAACTTCACTGCCAAATGTCAGGCGCATCACAAGACGATTAAATACTAGAAGAAATAAGAAGCCAGAAATATCAGGACCATCATTATCTGGTTTGTGTGCCTTAAAGAAACTAGATCTTAGTGACTCTGATTTGGTAGATGAAATCGCTGGTGATGTTTGGCAGCTGTCCTCATTGGAAGAGTTAAATTTGAGCCGAAATAATTTTGATGTATTTCCATCAAGAATATATGGGTTACAGCAATTCAAGGTCCTGAAAGTGGATGAATGCAAAAGCCTTGTAGCCTTGCCTGATCTTCCCTGGAGTATTGTAATGATAGAGGCAAATGAGTGCCCTTCCTTGCAGAGTCTTGGAAATCTTTCACCGCAACATGCATTCTTGAAAAAGGTCTCGTTTTTCAATTGTTTCAAATTGTACCAACAAAGCCAAAAAACCAGCATTGGCGCTGCGGATTTGTTGCTGCATTTGCTTCTCCAGGTAATTTCTCCATTTCATGAATTTATAAAATTATTATGTTTCCAAAATAAAGTATCAAGAGATCGAATTTGTTTAATTGTACAACAGGGTCACTCCACCTTTTATAGTCAATTTAGTATACTGATTGCTGGAGGAAAAATCCCCGAGTGGTTTGGTTATCAAAAAATGGGTCGATCAATCTCAGTCCAGCTACCTACAGATTGGCAAGACAACATTGCAGGGGTTGCATTCTCATTTGTTTTCGAGTGCCTTGTTCCAAAATCAAAACTAGGTGTTACTTTCAAGTTGATAAGTCCAAACCACCGAGAATACACATTTGAAAGTGCTCCAGCATCTGCTGCTTCAAAGATGGGGGAGGAGTACAAATATGATCACTTGTGGATAGCTTATATCTCTTTCCATCTTTTTCGTCTCCTCTTCCCCGAATTCACAACTGAAGATTGGAGTAAGGTTTGTTGTTGCCTTTCAATTAGCCTAAGACAAGAGCCATGGACCAAAGTGAGGAGGTGTGGGATTCATCTAGTGTACAAAAAAGACTTGACAACATCATTGGCAGCTGGGAGCAAAGAATTGACGGTGTATGACGAGGGAGGAGATTCAAAAAGGAAAGAAGAGGTGAAAGAGGATATCGTGGCACTAATGGCTGGGGTTAACGTTTTGAATTGGGACGTTGACTCCATTGAGCAAGATAACACTCAGCTTGCGAATTTGAGGAAATCACTTGCCTATAAGATTCAGAAGACTCTTTCCTTTGATTGCTAG

>PGSC0003DMG400003527

ATGGTGGATGCAGTGGTAACTGTATTCTTGGAGAAACTTCTAAATGTTCTCACTGAGGAAAGCAGATTTCTAAGTCAACACAGACAACAGTTTGAAAAACTGAAGAATGAACTGTTATTCATGCAAAGCTTTCTCAAGGATGCAGAGAGGCTCAAGAGGAAACACACCACTCTTAAAACGGTCATGGCCTGTTTGAGAGACTTAATCTTTGAAGCTGAAGAGATACTGGAGGACTGCCAGAATCAATCAGCAGATAGTGATGGGTCTAATAGATTCTCTACGCGCTTACATCCCAAAAGGCTATCTCATCGCCATCAAACTGGGAAGCGACTTGCTGAAATCAATGACAAGATCTCGGAAATAAAGCAAAACATTTCGACATACCTTAGAGTGCCACTTATGAATGAAGGAAGTATGGAGGCACACGATAATCTAATGACAAGATGGACTTCTTCCCTTTATGACCACACTCAGGTAGTTGGTTTGGAAGGTGACACAGAGAAGATAAAGGATTGGCTATTTGAAGCAAGTGATGGTTTACTTGCCATTGCATTTGTGGGTATGGGAGGGCTCGGGAAAACCACTCTTGCTCAGAAAGTCTTCAATGAAAGAAGCATGGAGAATCACTTTGAGAGGAGAATTTGGGTGTCTGTTTCTCAAACATTTACTGAGGAACAAGTCATGAGAAGCATATTAAAGAGTTTGGGAGATGCATGCATTGGTGACGACCAGAGTGAATTGTTAAGAAAAATAAATCAGTACCTTTTAGGAAAGAGGTTTTTGATTGTCATGGATGATGTTTGGAGCTTGGACAATGCTTGGTGGCAGAAAATCTATTCTGGACTACCCAAAGGTAATGGGAGCAGTGTTATTGTAACTACGAGAAATGAGTTAGTTGCTCGCAAGATGGGAGTCACAGAAGCAAGGACACACTGGCCAAAGTTCCTCAATGAGCACTACAGTTGGTTACTGTTCCGGAAGATTGCATTTGCAGCAACTGCAGGTGAATGCAATTTTCCTGATTTGGAGGATGTGGGAAAGGAGATTGTGGAAAAATGCAAGGGTCTTCCATTAGCAATCAAAGCAGTAGGAGGAGTGATGCTTTGTAAACCACCTTACTATCATGAATGGGTGCGTATTGCAAATCATTTCCGCGATGAATTGAAAGAAAATGATGACTCAGTGATGGCTTCATTACAGTTGAGCTATGATGAACTCCCTCCATACTTAAAATCCTGTTTCCTTTGTTTTTCACTCTTTCCTGAGGATTGTGTCATACTTAAAGACCAGCTGATCCGTTGGTGGATCGGAGAAAGTTTCATCCCTCTGAGAAGTGGTAGGTTATCAACTGAAGTTGGAGAAGATTGTTTCTCGCAACTATCCAATCGATGTTTGATAGAAGTTGTTGATAAGGCTTACAATGGTGTGATCCATACTTGCAAAATGCATGATATGGTTCGTGATTTGGTGATCAAAATTGCGGACGATGATTCATTTTCCACCCCATCTGATGCAAATTGTCGGCATTTGGGTATTAGTAGTGCGATGAATGGGAAGAAACTACTGAGCAATCGAAAATTACGAGCACTGCTGACAAACACCAAGAGTGGTGAAGTAAACAAAATCCCTTCTGATATTGCCAAGAAGTTCTGCAATAGTCGACATCTTCAGGTACTGGATCTGTCGAAATCAATTTTCAATGTGCCTCTTTCAAGTTTGCTGGAAGGCATTGGATCTGCCAAACAGCTTACTTATCTCAGTCTAAGCAATACACACCCGTTGATTGGTGTTCCAGATTCCATATCCAATCTTGAAAAATTACAGATTTTGGACTTCAGCTATTGCCAGAATATGAAAATGCTCCCCTCTTGCGTTTTAACATTTGTGGAACTAGCTGTTTTAGATTTGAACCACTGTGGGTCACTTGAGTACCTGCCAAAAGGATTGAGTAGGCTTTCCAATCTTCAAGTACTGCTTGGATTTAAGCCTGCAAAATTAAGCCAGCCTGGAGGTTGTCGTATTTCTGAACTCAGAAGCCTTACTCGACTGAGAAGGCTCAGTTTAAGACTAACTCAGGATGAGGAGATTGGCGATGATGAGGGGAACGCACTCATAGGTCTCCAAGAACTTCAATTTTTGACAATAAGTTGCTTTGACAGTCAAGATGATGGACTAGTCACAAAACTTGGTAAACTTTATCCCCCTCGACAACTCCACGAGCTGATTCTCAAATTCTATCCAGGTAAAGTAAGTCCTGAATGGCTTAACCCCAAATCTCTCCCCATGTTGCGATATATGTCAATTATTTCAGGTGATATGAAAGAAATGCACGAGAACTTCTGGGGTGATCGCAGTACTGTTTGGAAAATTGAGGGGTTAATGTTGGAAGCTTTAACTGATTTGAGATTGGAGTGGTCAGCAGTGAATCGAGTGATGCCTTCGTTAAGAATACTCAAGGCTAGCTGGTGCCCCGAGTTAGAGGCATTTCCAATTGAAGATGCAGGATTCAGAGGGGGGTTATGGAAGAAGGAAGAACATAGGTGCTGA

>PGSC0003DMG400005052

ATGGCTGAAGCTTTCGTTGAAGTTTTGTTACAAAACCTTAGCTCTTTTATTCAAAAGGAACTTGGATTGTTTTATGGCGTCGATGAAGAATTAAGAAAACTTTCAAGCTTGCTATCGACAATCAAGGCTGTCCTTCAGGATGCAGACCAGGAACAACTGAAAGAAAAGGCAATAAGAAATTGGTTGCAGAAGCTAAATTGTGCTACTTATGAAGTTGATGATGTCTTGGATGAATGTGCAGCGAAAGCGATCAGGTTGCAGGAAAAATCCCGAAGAATTGGATGCATTTCAATGCCATCTGGATTTACCTTGGAGAATCTTTTGTTTCGTCGACAGATAGGGAAAAAGGTTAAAGATGCCATCAGGAAACTAGATGGAATTGCCGAGGAACGTCTTAAATTTCATTTGTCTGAAGTGACTTCGAAGAAACGATTATCCACGACAGACGAAGTACGTGAAACTGGCTTTGTTTTAACTTCAGCAGAAGTCTACGGAAGGGACAATGACAAAAGGAAGATTGTCGAAATCTTAACGAAGCATGTGGATGATTTTCAAGAGCTTTTGGTTCTCCCTATAGTTGGAATGGGAGGTCTTGGAAAGACTACTCTTGCTCAATTAATTTACAATGATGTGTTGGTACATGAACATTTCGATTTGAAAATTTGGGTTTGCGTGTCACATAATTTTGACGAGAAGAGGTTGATAAGAGCAATCCTAGAAGCCATAGTTGGGAAAGATATAAATGCTTCCGAGTTAGCTTCGTTACAAAGTCAGCTCATAAACTTGTTGAGAGGGAAACGATATTTACTCATTTTGGATGATGTTTGGAATGAAGATCAGGAGAAATGGGATAAATTAAAAGCCTTACTAACAATTGGTTCGAGAGGTACTTCAGTTATTACCACTACTCGTCTAGAAAAGGTTGCTTCAATTATGGGAACGGTACAGCCACATCGTTTATCCTGCTTGTCGGAGTATGACTGTTGGTTGTTGTTCAAGCAACGTGCATTTGGTCTCGACAGAAAAGAAAGCTCCAAACTTGTGGATATTGGAAAAGAGATTGTAAGAAGATGTTGTGGGGTTCCTCTGGCTGCTAAAGCTCTAGGAAGTCTTTTGCGCTTTAAGAACGATGAAAAAGAATGGTTGTTTGTCAGAGATAGTGATTTTTGGAATTTGCCACAAGACGAAAGCTCAATCTTGCCTGCTCTAAGGTTGAGCTATTTTCACCTTCCTCAAGATTTGAGACACTGTTTTGCTTATTGCGCGATATTTGAGAAGGGCTCCAAAATAGACAAAGAAGAGCTAATTTATTTCTGGATGGCCAATGGATTTATCTCATCGGAGGGAAACTTGGAACCTGAGGATAAAGGTAATGAAGTATGGAATGAATTATACTGGAGATCTTTGTTCCAAGAAGTGCAACAAACTTCAGATGGGAAGATGTTGTTTAAGATACATGATCTTGTTCATGACCTGGCCCAATCTATAATGGATGATGGAATTCATGCAACAAAACTTGAAGGAGGGGAAAAGATATCGACAAGCAGAATTCGACATGCAACAATACATGCCGAAGATAAGTCATTTCTTGCTTTTCCTAAAAGTACTATGCCATACAACCCTTCGACAATCGCAATGTATGGTTCTTTAAGGGTGTTGATTTTTTGCTCAGTTCAGTTAAAGGAATTGCCATCTGCAATTGGAAATCTAATACATTTAAGGTATTTGGACCTTTTCAGTACATGTGTTGAGAGTCTTCCTCAGAGTATATGTTCTCTTCAGAATCTGCAGATGCTAAGTGTAGAAGATTGTTGCTTACTTCGTGTTTTACCGAAACATCTGAATTATCTGAGAAATCTTCGACATCTTCGGCTAAGAGGCTGTCCATTGAGTCACATGCCTCCTAATATAGCACAATTGACACACCTCAAGACGTTAAACAAGTTTGTCGTAGGGAAAAAAAGATGTTCCAAGCTTAGTGAATTGCGAGACTTGAATCTTCAAGGAGAACTAGTTATTGAGCACCTTGAGAGGGTTGAAAATCATATGGGGGCGAAAGAAGCTCTAATTTCAAAGCGAAACCTCCACAGTTTAGCTTTGTACTGGAACCATTCCGTTCGGTGTGAGTCATCAAAGGATGTTGATCTGCAGGTTCTTGAAGCACTAGAACCTCATTCCGACCTTAAGCATTTGAAGGTATCCGGTTTCAAAAGTACATGCTTGGCAAGTTGGATGAGGGCTTCAGTTTTGAGAACAATCATTACCCTTTATCTTCATGATTGTAAATACTGCTTGCATCTCTCTCAACTAGCTCAGCTTCCTTGTTTGAAATATCTTTCGCTGAGGGGAATTCATGTAGAGTACATTGACAATGACGTTGAAAGCGGAGTATCCCAATTAAGAAAGTTCCCGTCCTTGGAATCACTTGAGATGTGCAAGCTCCCAAATTTAAAAGGAGTTTCAATCGAAGAAGGAGAAGAACAATTTCCGAGTCTTCACGAAATGTGGATTGAAAACTGCCCTTTGCTGACATTTCCATGCCTTGTAACACTCAGAAATTTGAGAATTATGAAATGCTCAAACATGACTCTAGCCTCTATCTCTAATCTTTGCGGTCTAACCTGCTTAGAAATTGCTAACAACAAGGAACTAACTTCTTTCCCTGAAGAGGTGCTAACGAACCTCACCGATCTTGAAATATTGACGATTATGGATTTCTCAAAGCTGGAAGTACTACCAAACAACCTGGCAAGCCTCACTGCATTGAAGTCCCTGGATATTGGTTACTGCCACCAACTAGAGTCTCTACCCGAGCAGGGATTGCAAGGCCTCACCTCTGTCCGTAAATTGTCAGTCAGATGCTCTGATAGATTGAAATATCTGTCTGAAGGATTTCGGCACCTAGCTTCTCTGGAGGAACTTGAAATTTTTGGGTGTCCAAAGTTGGTTTCTTTTCCACAAGAGATTAAACACCTGAATTCCCTTCACCGCGTTCACCTTGATGGTCTGCCATTGTTCCACTCTAGAGAAGATACTGTTATTCACCCTGAGGAGTTGGGTTTCTGGCAACTGCCTGAGGCTTTACGGCATGTCCACAATCTGCAATCTCTTTCTGTATGTAGATTCTCAAGTTTGACTTTATTGCCTGAGTGGTTAGGCGAACTTACGTTTCTCAAGGAACTGAACATAGTACAGTGTGATAATCTTGCCTCACTGCCTGAGTGTATGGAGAGAATGAACCTCCAGTCCTTGAACATTTTGGGCTGTGCAATATTGGAGAAGCGTTGCAAACCGGGACAAGGAGAAGACTGGTACAAGATTGAGCACATTCCAAAAGTGAAAATCAGTCAAAATTGTGATTTTCTTAGTATCAACTTGTCATCAGAGAGGTTCAGTGGTCTAAGGAGATTTCACTTGTAG

>PGSC0003DMG400005046

ATGGTTTGGTGGGAGGCAGTGTTATCTCCAGCTCTGCAGGTTCTTTTTGACAAATTAGCTTCAGGGGATATATTAAACATACTTAAAGTATGGAATGTTAATGAATTGTTACTTGACAAACTTAAGATTTCTTACTTCATCAATACAGCTGTTCTTGATGATGCAGAAGAGAAACAGTACCTCAATCCAGCAGTCGAAACTTGGATCGATATGCTCAGAGATGCTGTCTTTGAAGCTGAGGATACTCTTGATGAACTAGCTACTGAAGCCTTGAGATGCAAACTAGAGACAGATTCTCAGAAATTTTCTCAGCAGGTGCGTAATAGTTGGAACTTTATCAGTATGAAATCAAGAATAGAAGAGCTTATAACTAGACTAGAATATATTGCAAAACAAAAAGATGTTCTTGGTTTAGAAAGTAACAAAAAGTGTTGTTATGGAAAGATGTACAGAGGTACTCCAAGTACTCCTTTGTTACTTGGATCACATGTTTATGGAAGGTATACTGAAAAAGAGGAACTAATCGAGTTATTGGTTTCAGATTGTGATGATACAAATAGAGTTGCTCCATTTTGTGTTATTCCTCTTATTGGTATGGGAGGGATTGGGAAGACTACTCTTGCTCAGATTGTCTATAATGACAAGAGAATTTGTGAGGAGTTTGACGTAAAAGCGTGGGCTTGGGTGTCTGATGATTTCAGTGTAACAAGTATAACCAAGTCACTGCTTGAATCTGCTACTGCAAAACCCTTCGACACGAATAGTTTGGAAATTATTCAGAATGGTCTGAAGAATATGTTCAGCAAAAAGAGAATTTTGATTGTTTTGGATGATGTATGGAGTGAGAGCTGTGATGACTGGAATGAGCTTTTAATCCCTTTCTTCGAGGGAGATAAAAGAAGTAAAATAATCGTGACAACACGAAATGAAGGAGTTGCATCAATCACTGGAATGCTTGCACCTTACCGTTTGCAGGAAATGTCACATGATGATTGTTGGTCTTTGTTCTTGCATCATGCTTTTGGTGTTCGAGGCATGGACATGAATCCGAGACTGAAAGAAATTGGAGAAGAGATCGTGAAGAGGTGTAAAGGCTTGCCTTTAGCTATCAAGACTCTAGGAGGTATGTTGTCTTTGAAATTGGACATTACTTATTGGACTGAAGTCTTGAATAGCAATTTATGGGATTTGCCTTCCAAGAAATATTCTGTTCTACCATCTTTAAGATTGAGCTACCATCATCTTCCGCCAAATTTAAGGAGATGCTTTGCATACTGTTCAATATTCCCGAAAGGGTATGAGTTCAACAAGAAAGATCTAGTCTTGCTGTGGATGGCAGAAGGGTTCGTGCAGCCAATGGCGCAGATAACTATGGAGGAAGTGGGAAATGGAAATTTTACTGAACTGCAATCTAGGTGTTTTTTCCAAGAATCCTCGCAAAATAGATCTCTCTTTGTGATGCATGATCTAGTACATGATTTGGCACTTAGTGTTTCCAGGAGAACATGTATTCAGCTGGAGGAAAATTGGAAATGCAGATTTTATGAGAATTGCGAGAAGGCTCGCTATTTCTCATGCATTCGTAGTAAATACGATGTCTTCAGAAAATTTGAAATGCTTAGTGAAATGAAGCGTTTACGCACATTCTTACCTTTAGCATCATCAGAAGGAGCTGAATTTTGCTATTTAACAAAGAAGGTACTGTCTGATATTTTGCCTAAGCTAAGCTGCCTTCGGGTGTTATCTCTGAGCTATTATTGTGTCACTGAGATTCCGGAATCCATTGGATTCTTGAAACATCTTCGCTTCCTCAATTTTTCCTACACAGAAATTAAATACCTTCCTCAGTCAATCAGTGATCTTTACAATCTACAAACGTTGCTACTATGCAACTGTTATTATCTCATTGAGTTGCCTGCTGACATGGGGAAACTGCTCAATCTTCGCTACCTTGATGTTAGCGGAAGTGGTTTACAAAAGATCTCGCTTGGTCTGGACAAATTGGTGTGCCTCAGGACATTACCAGAGTTTGTTGTGGGTAGTAATGTTTCTAGTAATAGGACATTACCAGAGTTCACAGTAGATACTAATACCGGTGGTACATTCGATCAAAAAAGTAAAGGTTCTGGTATAGGTGCATTGGGTAACTTGTTGCACCTTGAGGGTTCACTTTCAATACTGAATTTGGAAAATGTGGATAACATTTGGGATGCGCACGGAGCTAGTTTGATCACTAAGAAGCACCTTCGCGAGTTATTGCTTCAATGGAGTGATAGTTTTGAGGACCCAGAGAAGGCTAGAATGGAAACTGATGTTCTTGAGCTGTTAAGACCCCATCAAAATATAGAGAAGGTTACCATCAAGGGATATAGTGGTACAAAACTCCCTACTTGGACAGCGAATCCTTCATTCCACAAGCTGGTTTCTTTGAGTTTAATCAATTGCAAAGGCTGTCGATTCCTACCATCACTTGGTCAGCTTCCTTCACTCAAAAATCTCATGGTTAAAGGACTAAGTAAGATAAAAAGCATCGGTGATGAGTTTTTCGGCTATACTTCAACCATATTGACCCCTTTTGCATCTTTAGAGACTCTATCCTTTACGGATATGTTGGAATGGGAAGATTGGTTACTTGGTTATGATGGAGATAGAAAAGCATTCTGTAATCTCTTGGAGCTTCACTTGGAGGAGTGTCCTAAACTGCGGGGAGAATTACCTGACGTCCTTCCTTGCCTAGTAAAGCTTGTGATTTGTGAGTGTAAGCAGTTGGATTCTTCTCTTCCGCGACTTCCACAGCTAAATGAGTTGGAACTAAGGAGTTGTCATGTTAGGTTGATTAGCAGCATGCATGAGGTGACAAAACTCACTTCATTACAACTGTCTAATCTTTCAAATGAATATGTACCTGAATGTTTCCTAGCATCTCTTCGGCATCTGGTAATTAGGCATTGTGACTTACTGGTGTCCCTTTTCGAGGAAGGGCAGAATCTGCCTCGCAGATTGGAGTATGTAGAACTGGAGAACTGTCATAATCTGCAGAAGTTACCATCGTTACTTCATACTTTAACATCCCTTGAAGTAGTTATAATCACAAATTGTCCAAGGCTGGAATCTTTTGCAAGGAAGATATTTCCTTCTAACTTAAAAGCCCTTGCCATTCAAGGATGCAGTTTGGAGTCTTTACCTGAAGCTATGATGAACAGTATCTCATCTCTCAAGTATTTGTCCATCAACGGGTGTTTAATGCTAGCTTCTTTTCCAAGAGGTAGTGAGTTGCTTCCAACCACATGTCAGCAGCTGAAAATCGAGAAATGCCCGAATCTGGAGTTTCTGCCTGAAGGGATGATGCACATCAGCAATACCTCTCTCCAAGTATTGGAGATTTTCGATTGTTCTTCTATATCATCGTTCCCGGGAGGCCAACTGCCAGACACTTTGAAAACACTCACAGTTTGGAACTGCTTCAATCTCGAGGCATTACCTGATATCAGAACCTCAACAATGCTTCTTGAATCTTTACGAGTTGGGAATTGTACAAGTCTGAAGCACTTACCTCATGGCTTGAACAAGCTGTTGAATCTTAGTTATTTTGAAGTTGATGGTTGCCATGGTATCAAATGCTTTCCTCTAGAAGGCCTTCCACAAAACCTTACAAAAGTACTAATCATCGACTGTGAGAATCTGACATTCCTTCCAAAGTGGATGCAGAATCTTACATCTCTTCAAGAACTTCAGCTATCGAATTGTCCACTAATCACATCTTTCACGGAGGGTGGTTTTCCCACTAGCCTTGTGTCTTTGGATGTCAAAGATTGTAAGAATCTTATGCCAATGTCTGAATGGGGATTGCACAGGCTTGCCTCTCTTAGAAGGCTGACAATTCATGGGATCAGTTCAAATCTTTCTTATTTTCCTCAATGGTTGCTTCCATCAACTCTAGAAACACTCAATATAGTTCAGTTATCCAATCTTGAATCTCTTTCTCCCTGGCTGCAAAATCTCACTTCACTCGAAAATCTCAAGGTCAAAGATTGTCGAAAGCTTCTTTCGTTACCAAAGGAAGACATGCCACCAATGCTTTCCTATCTTGAGATAAGTGAATGTCCCTTGAGTGAACAAAAGTGTGACTTGTCCAAGATTGATCATATCCCTTGCACTGTGATGTAG

>PGSC0003DMG400043400

ATGACATTAGGTGCTTCATTGGTGGCAAAAGCTGTTGACTATACGGTGGCCCCTCTCGGCAATCAACTAAGATATCTTTATCAGTATAACAAAAATACCGACAATCTGAGAAAGCAAGCTGAAAGGTTGCAAGAGAAGACACAAGATATCCAAGCATTGGTGAACTATGCCAAAAGAAATGACAGAGAAATCAAAAGCACAGTTAAAAATTGGTTACAAGAGGTAGATGCAATCACTGCTGAGATCAAATCATGGGATGATAAAACTCAAAACTTGAGCAAAATGTGCCTCCCTCATCTGGTCTCACGGTATAAGCAGAGCAAGATGGCAACTAAAAAAATCATGACCATCCAAGAACTTCTTGGTAGAACTCTCACGGAAGATGTATCCATACCGCAGGCCCCTCCTTTGAATATACCATTCATTTCTGACCAAGAAAAACCCGAAAAGGACCAGGTTATTGAGATGGTAGCAAGTAGTTCATCTGATGTATCCATACCGCAGGCCCTTCCTTTGAATATACCATTCATTTCTGACCAAGAAAAACCCGAAAAGGACCAGGTTATTGAGATGGTAGCAAGTAGTTCATCTGATGTATCCATACCGCAGGCCCTTCCTTTGAATATACCATTCATTTCTGACCAAGAAAAACCCGAAGAGGACCAAGTTATTGAGATGGTGGCAAGTAGTTCATCCGTTGTTGGTCAAAACGTCAGTAGAGCAAATGGTGCTCTGCTAAGTGCGGATGATGTTTCTATCAAGCGAAGACTAACCATGGATCCAATTATTGCTTATCAGCTAAGAAAAAATGTGGCAAACATTATTCACCTGCAAAGAGAAGCAGTGAATCAGATCATTGAGGAACTAAAGGATGACCACTATAAGGGGATAGGGATTTATGGCATGGGGGGCATCGGTAAGACCACTCTTGCCGTAGAAGTAGGGAAATTGGCTAGAGACTGTGGAATTGTTAAGGAAGTTATAATGGTGGTTGTTTCCCAAACGCCAAACATAAGAAAGATCCAGGGTCAAATTGCGGATATGTTAAATCACAGACTTGAGGAGGAAAGCACATTAGGAAGAGCAGGTAGACTTTATACGCGACTGTCCAATGAGAGCGTACTTCTCATACTGGACGATGTTTGGTCATACATTGACTTAGCAGAGATAGGGATCCCTCATGGTGATGAGCACAAGGCCTGTAGGATTATGCTTACCACGCGACAAAAAGATCTATGTACAGCTATAGGGACCAAAGGAATACCATTGAAACTTTTATCAGAAGAAGAATCGTCGCATTTATTGAGGAAATATGCTTGCACGAGCACTTCTGACCTTTGTCCGGAGTTGGATAGCATGGTCATGAATTTTGTCAAAGAATGTCAAGGCCTGCCATTGGCACTTGTGACTGTTGGAAGTGCATTACGAGGAAAAGAACAAGTGGAGTGGGAAGCAGCACTACAGCTCCTTAAGAAGTCCCAACCCTTTAGCCCCACTTATGCAAGCAAGACCATTTTCTCCTGCCTGGATTTGAGCTACAATTTTTTGGAAAATGAGGAAATCAGGTTGTGCTTTCTGATGTGTTGTTTGTACCCAGAAGATCGTGAAATAAGTATTGAAGACTTGACTAGAGACTGGACAGGAAAAGGGCTGTTCTCAGATGTGGACACTATTGAAGAAGCTAGAGCCAGAGTGTGCTTAAGAGTTGGCCAACTTAAATCATCTTGCCTGTTGCTTGATGTTGGGAAGGAGGGTTTCGTTAAAATGCATGATGTTGTTCGTGACTTTGCTATATATATAGCATCTGAAGAGAAGCATGGATTCATGGTAAGAGCTGGTCACAATCTGAACAAGTGGCCACCAAGGGAGTCATTCAGCCAAAAAACAGCTATTTCCTTCATGAACAATAATATCCATGTGCTTCCAAGTGATGTACATTGCCCCAATCTTCAAATTTTACATCTTGGAGAAAATGAAGGTCTTGAACAGATACCAGTGGACTTTTTTATACAGATGAAAATGCTCCAGGTTTTGGATTTAAGTGAAAGGGTTGGTATCCATTCACTAAATCCTCTCTATCAGTTAGTCCCGAATGCCACAAGAAAAAATACATTTCCCCTCAGTTTTCCATCATCCGTTGAAGTTCTCACAAACCTGCGAACTTTACGTTTAGACCACTGCAAGTTAGCTGATGTATCTATTCTTGGAAAATTAAAGGGCCTGGAAATTTTAAGTTTATACGGGTCCTCTATCACACAACTTCCAAATGAATTTGGGGACTTGGTTAATTTGAGACTATTGGATTTGTCATTCTGTGTGTACCTTCAGAAAATCCCGGAGAACTTGATATCGCGCCTTGTTCAATTAGAAGAACTTTATATGGGTTGGAGTTTTCGATTGTGGCAGCTAGCTGATGGATCTGCTGAAGGGAGTGGCCAAGCAAGCTTGTCTGAGCTAATGTCGCTGCCCCAATTAAATATTTTGTGTGTGGAAGTCTCCACCCTTCTAGCTTTCCCTGAGAACTTTGATCTTCCTAGCATACACAAGTTTGAGATAACAGTAGGTTACCACTCAGCTATATGTTATCCAAACTCTAGGAGGTTCTATCTTAGAGAAATAAAGACTGGTATACCAAATGGGATGAAGCATATGCTTCAATTCTCAAAGGAATTGACAATGTTCTGTGCATCCAAGGTAATACTGAAAAGTATCTTTGATGTCGAAGGGGGCTTAAATCACTTGAAAACTCTTGAAATAACTGCCAATGATGAAATTACCTATTTCATTGATGAGGTTCTTCATAGCGATGCACCTTTAGTTTTAGGATCTTTGGAGAAATTACATCTTCGGACGTTCAAGAAGCTATTTTCACTTTCTGTCCGCCCAATCAAACCAGGTTCCTTCCAGAATTTGCGGATTCTAAGGGTTGAAAATTGTCATAATTTATTTTTTCTAATTCAAACTTCCTTGCTCCAGAGGCTATCAAGTATTGAAGAAGTTCATGTGTATTCATGTAACAAGTTACTCGATATATTTCAACTTAACGAGGCAGCTTTTGATGAAGAACAGAAGCTCCTCTCAACGCTCAAAAAGATCTGGTTAGCTAGATTGCCTACGCTGTTCGAAATCTGGAGAGTACCTAAGCAGTTACTCCTGAATGCAACCCTTCAAAATAAACAATGCTTCAGTAACATAACTGATGTGTTGATTAGGTATTGTGATAACTTGGAATATGTATTCCCATCTGTAGCTGCTCAAAATCTATGTCAACTGGATAATCTCCAAATTGTGGAGTGCCACAGATTGACAAGAATCATAGGAGAAGAACCAGAAGGTGTCTCAGCAAATGTTCAAAATGGACATGTTTTATTTCCCAACCTCAGAGCTGTTCATATAGGAAGCTGCAGAAACCTGAGAAATCTATTTTCGATTATGACAGCTCGAAGTCTTGGCAAACTAGAAGAGTTAAAGGTAAATGACATGCCAAATCTGGTAGAACTCATAAGCAATGAAGAAAGTGAAAGGGAAAGGGAAGAGGAAAACGATAAAATAATCGTGCTGCCAGAGTTGAAAGTCCTGAGAATTACAAAATCTGGAAACGTGGAACGGCTTTGTACAGAGGCTTTCTTTATGGATCTACCATCGTTAGAAGAATTTGTTCTTCTGGAGTGTCCTAAAATGGCTGACACTGTTAAACGTGGACTGGGTAGCGCATCAAATCTTCTTAAAGCACAGATTGAGGAACAATCATTCTTTGGCACAATGGCACGAGAGGTTTTCAGTAGAAAGGTGTGA

>PGSC0003DMG400024337

ATGGATCTTATTGATGATTTCTTACATGGCTTGAAGCAAATAAAAAATGAATTCATTGCTTCAAAATGGGATGACTTTGAAAAGCTAAGAATGGATCTGAGATTCCTAAGAATATTTGTCCTGTTTGGGAATTCAAATTTGGATGACTTTCATGGCAGGATGTCCCTAAATATAAACAAGTTCAGTAATAATGATAAAGTAAATCTAGCCATGACAACGGAGGAGAAAATGTTTGAATACCTCTTCAGACACCTCCATGATCTACCAAAGTATTGTTCTGATTTTCTTCTCCCCCAGATGACTGACTACAAAATTCTTCGCCAAGTATTCCATCATCTGAGAGATTTCTATCCTATCCTTGTGGCCAACAAAACAAGTACTGAATATCTCTATCCCCGCTTCCAATTGACTGCTGATAGAGTAACACAATTCTGTTTTGATCTTTGGACTGGACAGTATAGAGGAGATGATGGCGATTACAAGGAGTATGACGTCTCTCAATGCTCTTCCAAGATCACTTCTCTACTCATCTACTTAATCCCTCTTGAGCTGGATGTTCTATACATTTGTATTTCTAAGCTCATGAAAGAATCAAGGTCAACAGAACTAGAAGGGTTTGTTAAGCAAATCCTAAAAGCATCTCCAAGGATTCTTCAACTTCGTCTGATTCATCTCCAACGACGCATGGAAGGTGCAGTAGCTATCAATTACGCTCCAACTCAAAGCATTAATGTCATGATGGAATTCCTATTAATCTTTCTAACTGATATACCAAAGCGCTTTATCCATCCTGAAAAATTGAACGATATGTTGGCACATGTTGAATTACTTACAAGGAAGATATCCATTTTGGTGGGCAAGCTGTTGGAGGAGAGCTTTGAGAATAATATCAACGAAGCAGACTTTTCAGCTCCAGGCTTGTTGCAAGAAATTGAACAAATGAAGGGAGATATCAGATAGATTATTTTGAAAGCCCCAGAATCATCTCAACTTGGCTTTCCTATGGATGATGGTTTCCTCTTCATGAATCTTCTACTCGGACGTTTATACGATTTGTTCATTTCCAATGTTTATTTAGTTTCTCTGATAAAGAAAGAAATTGGGATGGTGAAAGAAAGCTTCGAATTCCTAAGATCATCTTTTGGGAAAGGCAGGCAAACATTGGATGACACTAGTGGAGTAGTTAAAGATTGTTGGGTGCGTACTTTAGATGTGGCATATGAGACAGAACATGTCATTAGTTCCATTCTTGTGAGAGATAAAGCTCTCTCGCATCTCATCTTCTCACTTCCAAGTGTCGCTGATAAGATCAAGCTTATCGTGGCAGAAGTCACCAGTTTACAGCTGGAGGATAAAAATGGGAATGACCCCCTTGATGCAAAGTCTTCCGACAAGCCAATTGAGTCAACCTCATCATCTTTTGTTGAGGTAACAATAGGTCATGAGGAAGAAGAATCCTAGATCACTGACCAGCTCCTAGATGAACATGAATTCGAGCTTGATGTCATTTCAATTGTCGGAATGCCAGGACTCGGTAAAACTACTCTGGCCAACAGAGTGTATAACAATACATTAGTTACTAATCATTCCAAAGTTCATGCTTGGTGCACTGTTTCCCAAAAGTATAACAAGTCATAGGTGTTGCGGGAGATTCTTCATCAAGTAACTGGCTCGGAAGGAAACAAAAGTGAGGATGACCTTGCTGAAAAGCTAAGAAGAGCACTACTTGATAAAAGATACCTCATCGTCTTGGATGATGTGTGGGATATTGCTACAGGGGAGATGTTAATAGCATGTTTTCCAAAGGATAAGAGAAGAAATAGAATTATTTTAACTAGCCGAAGTATGAAGGTAGGTTTGCAGGTTAAATGTCATAGTGATCCTCGCCGCCTTCAATTTTTAACACCTGAAAAAAGTTGGGATTTATCCGAAAAAAGGGTATTTGGAGAAGGACGCTGCCTTGCTGAACTGTCCTATGTTGGACACCAAATAGTTGTGAACTGTAAAGGTCTTTCTTTGGCTGTTGTTTTGATTGCTAGAGTAACTGTTAGAGTAAGGAAAGGAAAGAAAAAGGAAAATGAAAAGGATTTGTGGCTTAAGATTCAACATAATCTAGATTCCTTTATTTCTGCCAACAAGAATTTGCAGATGATGAAGGTTATGCAATTAAGTTATGACCATTTACCATACCACCTAAAGTCGTTGTTGCTTTACTTTGCAAGATCTCAAAAGAGCAAACGAACTCCAGTCTCTAAGTTGATGCAGTTGTGGATGGCCGAAGGGTTTGTGGATCATGATATCCCATCCAAGAGTAGTTTAGAGGAAGCAACTCAAAGTTACTTGGATGCTTTAATTTCCAGTAGCCTGATATTTGTGGATCATATCCCCTCCAAGAAGAGGACTTGGCCTTTTTCTGGTATGATCAAGGTTTGCTATGTGCATGATGTTGTGCACGATTTTTGTTCAGTAAAAGCCAAAAAGGAAAAGTTTTTCAAGTTAATCAATTCAGGTGCTCCATTTCATGCTTTGGATTTCCTACACCGTCGTCTAACCGTTCATACTAAACATAGCCAACTTCACAAAAAATGTGTTTTGTTCAATTCTACTAAGTCTTCAGCTGGTAGTAAGAATCTCATACCTTTGAAAGTGAGGGATTCTTTCTTTGAAGATTACGCCGACTATATCTGTCACACAAGACACTTGAAACTTCTTAGAGTGTTGCAACTGCGTAACATTCTTCTGAATGATTATTTAATAGAAGAAATAGGTTCTCTATTTTATTTGAGGTTCTTAAGGATTGAGACTCTTGATCTAAAAGTTATCCCACTGTTGTGGTTGAACCTCCAGAATCTGGAAACTCTGTTGATCAGAACACAATGTTCCACCATAGTATTACTGCCCAAAATTCTCAAACTGTCAAAGCTGAAACATATTAGCATTGACAACATGAGTTCTTTCTTTGAAAATGAAAAGGATGCAGACAATATTATGCATCAACGAAATAGAATATTGGAAGCTGAGAATTCAAAGTTGACAACTTTATCCCAAGTTCAAATTTCATATTCTGAAGGCACAAATGATGCTCTAGAGAAGTTCACAAATCTTCAGCACCTTGATTGCATCATCGAGGAGCCGATAGATCCTCCTTCACACAACGATTGGTTTCCTAAGTTTAATGTCCTTAATAAACTTGAATCACTCATTGCAAGATACAGAGATGACTCCGAGTTAATAGACCAAATCCCACCTCCCAATGAATATCACTTCCCTACAAGCTTGAAAGTGTTACGGTTGTATGGTTTTCTGCTGAGACCTACTTTGTTGTCAGTAATCGTGGCATTGCCTGAGCTTGAAATTCTTGTTTTAAACACATCTGATTTCAGCGAGGACAAGTGGGATGCAAGTAAGGACATCTATCAAAGTCTTAAGACTTTACATTTGGAAAATGTCAAATTATCAGAATGGCAAGTTGATAGGGAAACTTTTCCCAACCTTGAGGAATTAATACTAGAATATTGTTTCAAGCTTACGGAGATCCCTTCTGCATTTGGGGATATAGATACTTTAAAGTCCATTCGCGTGGTTCAAAGTAAGTGTGAGCTTGGAGATTCAGCCATGGAGATGAGGAAAGATGTGGAAGCTTACACAGGAGAGAACAAACTTCATGTCCATTTATCATATGAGTATAGTGCATTAGGTTACGAAGAAGAGAGCAAAGAAGAGGTAATTAATTTCTTAGTCATAAAATTGCTTTTGAGTTCAAGTTATATACTAACAACTTTTTTTTGTTGGATGGCATATTTGAACTTGGTTTAG

>PGSC0003DMG400002820

ATGGAATTCTTATCTATTTTTGTGGAAAAACTCACTGATTGCTTGATCCAGCCAGTTGCGCGGCAGATTGGTTATTTCTACTACTACAAGAGCAATATGAGATGTATGGATAAAGAATCTGAGAAGCTGAAGAATATCAGAATGACAGTGCAGCGAAAAGTGGAAGATGCCAGGAGAAACTTACAAGACATTTCACCTAATGGCAAGGCTTGGTTAACTAGTGTTGATGCAACTACTGCAGATGTGGAAGGCGTAATGCGAGGTGGAGCTGAGGTTGAAAGGGGTTGCTATTATGGTGTGTGTCCGAATTTGAAGTCACGTTACTCGATGAGCAGGAGAGCTAAGAAAATTACACTGGAGTTGATTGAACTTCAAAATGAAAGCAACAAGCCTGATGTTTTCTCATTTGATCATCCAGTCGAAAGTGAACCTGAGGCTATGTGTAGTAACAATGTTGAGGAGTTTGATTCCAGAAAATTGAAGGAGGATGAGGTCATGGCAGCTTTGAGAGATGATGGGGTTACTATTATTGGGATATGTGGTATGGGTGGAGTTGGTAAGACAACACTGACTGAGAAAATCAGACAAAAGGCGAAAAAAGAAAGGTTGTTTAAAGATGTTGTCATGGTAATTGTCAGTCAACAAATAGACTTTAAAAGAATTCAGAATGAGATTGCAGGAGGAGTCGGGCTGACATTAGAAGGGGATGATTTGTGGAGTCGTGGAGATCTGCTGCGTACAAGGTTAATGGATCAGAACAGTCGCATCCTTATAATCTTGGATGATGTTTGGAAGGCTCTTGAACTGGAGAAACTTGGAATTCCTAGTGGTAGCAACCACAAACATCGGTGCAAAGTGACATTCACAACGCGTTTCCAACATGTTTGTGAAGCAATGGGAGCTCAAAAGATCATGGAAGTTGGAACTTTATCTGAAGAGGAAGCATGGATCCTTTTCAGACAGAAAGTTGGTAATTCAGTCGATGATCCTTCTCTACTTGATATAGCAAAAGATGTTGCCACAGAATGCAAGGGGCTGCCACTTGCAATTATTACAGTTGCAGGAGCACTAAAAAAGTGTAAAACCAAGCGTTCGTGGGACTGTGCCCTTGAAGAATTACGCGGTGCAGAAACAATAGATATCCCTGAAGTGCACACAGAGGTGTATAAACCTCTGAGGCTAAGCTATGATTACTTGGGAAGTAATGAAGCCAAGTGCCTCTTTTTGCTTTGTTCCTTGTTTGAGGAAGATAGTAATATCTGTCCTGAAGAATTACTTAGATATGGAATGGGGCTTCGCATCTTTCCGAGAATCAAAAATTTGGAACATGCAAGAAATAAGGTGTGTTATCTGTTGGAAATATTGAAAGATTGTTTCTTGTTATACCAAGGTTCAGAAAAAAATTATGTCAAAATGCATGATGTGGTCCGTGATGTGGCTATATATATTGCGTCTGAGGGCAAGGATATATTTATGGTAAGCCATGATGTGAACTCAGAAGAGTTCCCAAGAAAAGACTCTTACGAGCAATACAGTCACATGTCAATTGTTGCAAATAATTTGTTTGATGAGCTTCCTAGCCCAATATTTTGCCCAAAACTGAAGCTTCTAATGTTGAAACTTTGTTTTGAAGAAGAGCCAATCAAATTAAAGGATGATTTTTTTGATGGAATGAGTAAACTCAATGTCTTAAGTTTTAGGACAGACGATTATCGTTACTCCATTCTACCCTTTCCAGCATCCATTCAGAGGTTGTCAAATTTGAGGACGTTGTGTCTGAGTAATTTAAAGTTGGATGACATATCCATTATTGGGAAACTTGTCACTTTAGAAATTCTCAGCATAAGAGATTCTAAGTTAGGGGAGCTGCCCGTGGAGATAGGAAAATTGACCAATCTAATTATGTTAGAGTTGCGGAATGAGGAAAAAGCACTGGAAATGATTTCACCAGGGGTCTTATCAAGATTAGTTCGATTGGAAGAACTTCATTTGGTGGGAGTAGAAAATTGTAGTTACTCCAACTTGAGGGAGCTGGAATCGTTATCGAGATTGACTTCACTGACATTAAGTGAATGTTCTGGAGATGTGATCTACAGTAACTTGGGCCTTTCCTCTAAGTTGACACGGTACGCTCTTACAGTGGGTAGAGCTTACAAAGCGACTTCAAGCATGGATGATTACGACAAGAATATTGCCTTAAAGGTCACTGAGACTGCCCCATTGGGTGATTGGATCCGCCACATGTTGAGGAAGAGCGAACATGTACATTCAACCGGAGAGGGCTCCAAGAACGTGTTGGCTGAGTTGCAGCTGGATGAATTTCAGAATGTGAAATATCTCTGCCTGAAACACTTTGATTCATTAGTACATATCCACTGTCAGAATAATATTTCATTCCCCAAACTTGAAAGACTGGAGGTAACAAAGTGTCGTTGTCTACAGTATGTTTTTTGTGTGTCATTGGCTGGAGGGAGTTTAACAGTAGCTTGCCGTGATGATGAGGAAGAGGAGATCTCTCGGAGGACACGTGAAGTAATAAAGTTCCCCAATTTATATGATTTGAACCTTCGATCTCTGAAATGCCTCACTCACTTTTGCAATGACACTGTTGAGGGCATTGAGTTCCCTCGGTTACGGAAAATGCACTTCAGCAGCTTACCTGAGTTCCAAAATTTCTGGCATACAACCAGCAACTCTATCACTGACTCAAATCCTCTTTTTGATGAAAAGGTTCCCTTCTACTGCTCTACGTGTGACCGATCAATTGTATATTTGCAGGTTTCTTGTCCCAACCTGGAAGAGCTATCTATCGATAGGGCTGACAGCATAACTGCTCTATGCTCTCACCAACTTCCAACTGCCTACTTCAGCAAACTTGTAAAATTCGAAGTAGATGGTTGCAGAAAATTGAGAAACTTGATGTCTCCATCAGTGGCCAGAGGTCTTATGAATCTCCGAATACTAGATATAAGAGACTGTCTGTCAATGGAAGAAGTGATCACAGAAGAGGAACAACAAGGAGAAGAAATCATGACTAATGAGCCCTTATTTCCCCTGTTGGAACATCTGAACCTTTACGGTCTGCCAAAGCTGGTGCATTTCTTTCTAGCCAAGTGTGCTCTTGAATTTCCATTTCTCAGGGATGTGTGGATTCATGACTGCCCTGAAATGAAGACGTTTGTCCAACAGGGAATATCTGTGAGTACACCAAGTGTTGAAAGTGTGAACAATGATGATGAGGTGAAAGTAGTTGATCTCAATAAAGCCTTGTTCAATTCCAAGGTTTGTCTTGTGCCGCTAGCTGTTATAGTAATAGTTATTTCTGTTTGTCACTTAAAGCAACTTTTTTTAGTTTTACTTACTGTTACTATGATGCAAGATGAGAATATGGTTCAATTTTGGATTTGTACTTTAAGCTATATTGAATTTTCATCCCTTATATAAAACACAAGGTGGCAACAATTGCAGGTTTCTTGTCCCAGACTGGAAAAGTTACAACTCAATGGAGCTCACAGTATAATTGCTCTATCCCCTCACCAACTTCCAACATGCTACTTCAGCAAACTTGAAACATTGCATGTATCGTTTTGTGGAAATTTGAGAAACTTGATGTCTCCATCAGTGGCCAGAGGTGTTTTGAATCTCCAAACACTAGATATAGAATGTTGTGAGTTAATGGAAGAAGTGATCACAAAAGAGGAACAAGGAGAAGAAATCATGTTTAATGATCCCTTATTTCCCCTGTTGGAAGAGTTGAGGCTTCAAAGTCTGCCAAAGCTGGGACATTTCTTTCCCACTAAGCATGCTCTTGAATTTCCATTTCTCAGAGAAGTGTGGATTAGTGACTGCCCCGATATAAAGACGTTTGTCCAACAGGGATCTGTGAGTACACCGAGTCTTGAAAGTGTGAGCATTGATGATGAGGTGAAAGTAGTTGATCTCAATAAAGTGATGTTCAATTATAAGGTTTGTCTTGTACCGCTAGCTGTACGGATTTGTAGGGGAATCTCTTTTATTTCTTTCCTTAGCAGCTCATGTACATGTATTTATATTGCTTACATTTTGGAATGAGATATCAAGTTATTCTCCATACCTTGTCTATTACATGTTATCGTAGTCAGGTCCATGCTACAATTGGGCCAGTGCGTGAAGGTAGGTGTTAGAGTGGGTTAGAATCACATATTGGTTGGTGATGTAATGACGGTCTGCTTATATGGATTTGGACGATCCTCATGTCATCAACGTTTGTAGGTTACTTGTCCCAGCCTGGAAGAGCTACGTATCTGGATGGCTCACAACATAACTGCTCTATGCTCTCACCAACTTCCAACTGCCTACTTTAGCAAACTTGAAAGATTGGACGTATCGACTTGTGGAAAATTGAGAAACTTGATGTCTCCATTACTTGCTAGAGGTGTTTTGAATCTCCGAATACTAAAGATAGCAGGCTGCCAATCAATGGAAGAAGTGATCACAGAAGAGGAACAACAAGGAGGAGAAATGACTAATGGGCCCTTATTTCCCAAGTTAGAACAGCTGGATCTTTACGATCTTCCAAAGCTGGGGCATTTCTTTCAGACGAAGCATTCTCTTGAATTTCCATTTCTCAGGAAAGTGTATATTTATAGCTGCCCTTCAATGAAGACATTTGGCCTCAGTTCTGTGAGTACACCGAGTCTCGAAAGTGTGAACTATGATGATGAGGTGAAAGTAGATGATCTGAATAAATGGATACAACAGATGTTCAATTCTAAGGTTTGTCTAGTGCCGCTAGCTGTA

>PGSC0003DMG400007469

ATGACAGATGCAGGGGTGAACTTTCTGGTGGGGAATTTGTTGGATCTGTTAAGTGGTAATATAGATCTGATTAGAGGGGTAGACGATGAGTTCAAAAATTTACTGGAAGAAGTGCAACGCCTGAAAGCATTCCTTGACGATGATGCTAAATTCCACAGCGAGAGCAGTTTATGGGATCAATTGGTGAAAGAAATTCAAAAAATGGTACATAAATCTGAAGATGTAATTGATAAATTTCTGATTCAATCTAAGCTGCATCGAGATAAGAATAAAGTTGAAAGATTCTTCGATGTCGGTCATATGGCAGTAGTTAGGGATCTTGCAGCTGAAATTAAAGACATCCATGAGAAGGTGAAGAAACTTCGGCAAGACCATACAGATTCTTTCAGTCCTAAACCACTTCTTGACATCCCTGAAAAAGCCCACCAAGTAACCCAGGTATGCAACTTTCCATATCTTATTTTATTTTTAAAAATATATCGAATTAATGTTAATAAGGGTCCTTTGGAGGATGATGAAGTGGTCGGGTTTGATGAGGAAGCTAACGAAGTGATGAAGCGACTTGTTGAAGGAGTGTCGGAAAGTGTGGATATTATCCCAGTAGTGGGTATGGCCGGACTTGGAAAAACTACACTGGCAAGAAAAATCTGCAATGATTCTAAGCTTTCATTTGAATTTTTCAGAATCATTTGGGTTAATGTTGGTCTGGAATACAAAATAAAGGATATTTATCTTACGATTCTCAACTTCTTCACAAAACACATAGAAGATCATTTCAATGAGGATGTGGACGCATTAGCTAAGACAATATGTGGTTTTATGAGTGAAGAAGGAGGTAGATGTCTCATTGTTTTAGATGATGTGTGGGTAGCAGAAGTAGTTGATGCTGTCAAGAAAGTTTTCCCCAGAAACAAAAAGGGGCATCGGATCATGCTGACCACGCGTGATGGATACCTTGCTACCTATACCAATCCAGATCCACATAATTTGAAGCGTTTGACACCATCAAATAGTTTTGAGTTGTTGGAAAAAAGGGCTTTTGGCAATGGAAATTTTCCTGTGGAGTTAGTTGAACTTGGAAAAAACATTGCACAAAGATGCTGGGGAGTACCACTTGCAACAGTGCTAATTGCAGGACTATTAAGAGGTCTTCGGAACAAAAGTGACTGGGTAAGAGTTGAAAGCAATGTGTCGCAACGTCTTCATAACAAGGATTATGATTTTGCTTTATTTATAGAGATGAGTTACGATCGTTTGCCTCAAGAAATGCAGAGGTGCTTTTTATACTGTGGCATCTTTCCTCGGGGCTTTGATATCCCTGCTTGGAAATTGATTCGCCTGTGGATTGCGGAAGGGTTAATAAAACCCCAGCAGTCATCCACTCTTGAGGAGATAGCAGAGCGTCATTTGAATGACCTTGTTCATAGGAATTTAGTGATACTGTTGCAAAAGAGGTCTGATGGTTCAAATAAAGACATGTCGTCTTCACGACATGTTACAGAGTTTTGCAGAACAAAGGCTGCTAACAAATGGCTTTTTCAAGAAATATGTACAACAGTTGATAACGCTATTCCTTCTATACAGGACTCAGACACTTGCCGTCGATTGTGCATTCAACCATCTACTCTGAATGACTTTCTCTCCACAAAACCTTCTGCAGAACATGTCAGATCTTTCTATTGTTTTTCCTCGAAACAAAAACAAACTGACTTGTCTCCTAATGACATCAAACTCATTCACAAGCATTTCCACTTACGTAAAGCAGACATATGGAACATGTTACAGTTGAGGCATCTGCACACCAGCATACCTGCAAAATTGCCGTCCCCTACTATCACCACAGGTAAACCTTCTTGCCTGCAAACTCTTTCTTTAGTTGCACCAGAAAGTTGCAAGAAAGATGTGTTCGCCAAGGCATGTTTTCTAAAAAAATTGAGCATTCGAGGGCAAATGGCGGTTTTTTTTGAGTCCAGGGGTGGAATCAGCAATCTTGAAGAGCTTAAGTGCCTGGAACATTTGAAACTGTTGAATGATGTTCTTTACATGAATAAAACAATTCATCTTCCAGCAACATTCTTCAGACTTGTACGTACAGTGAACAAGTTAACTTTGGCAAACACAAGGTTTTCATGGAATGAGGCGGACAAGTTAGGGCAATTGGAATCCCTTGAGGTTCTAAAGTTAAAACATAATGCATTTGTTGGTAATTCCTGGAAGCCAGAGATTGGAGGTTTTCGCCAGCTCCAGGTCTTATGGATTGAAAGTTCAGCAGAATTAGAATCTTGGGAGGCTTCGGATGTTAACTTCCCCATGCTTAGTCACCTTGTTCTTATCTCTTGTGATAAGCTTGACGCTCTACCAATTGAGCTAGCTGATTTACCTAACTTTCAAGAGATGAGGCTGGAGAAAATGAGCAAAGCCATCGAATCTGCAAAAGCAATAGAGGAAAGGAGGACATCTAAGAGCATCAAATTCAAGCTAACCATATCATGTTGA

>PGSC0003DMG401007461

ATGGCAGATGCAGTAATGACTTTGCTTGTGGAGAGCTTGTGTCAACTAATCACTGAAAAAGTGAAGCTGATTGGAGGTGCAAAGGATGAGTTGGATAATCTGCTGCAAGTAATTAACACACTGAAAGCATTCCTTCAGGATAATGATCAACAAGAAAGCAACAGCAATTCGTGGAAAACATTTGTGAAGAATATCCGAACACAAGCGCATAAAGCTGATGACGTTATTGACAAATTTGTACTTGAGGCGAAGCTGCATGAAGAAAAAAATATATGGAAAAAAACTTTATCTTTCGGCAGGCATATCAAAAGAGTTAATAATCTTGCAAAAGGTATCAAAGATATCTGTGAGAAGGTGAAGGTGATTCGCCAAGAAAATCCACAAGTTCTCCAGCCCAGACCAATGCTAAATCTACCCAGAAGACTTGCAAGGGAGCCTCAGGTAAATTATAAAGCTACATTCTTGGAGGTTAGAAATATCGTTGGGTTCACTACAGAATCAAATGAAGTGATGGAGCGGCTTGTTGGAGGAACAGAGGAGGTAGAATGCATACCTATTGTGGGTATGACTGGACTTGGCAAAACCACACTGGCAAGAAAAGTATGGAGTGATTCTCAGATTACAGCTATATTTTCCAAGATCATTTGGGTTTCTGTCGGACAGTCCAACGAACTAGAGGATATCTTTTTTGTGATTCTCAAATCATCTACGGGACCCAATCAAGAATTTCGCTTGACTGGTGAGAATGAACTAGCTAAAGTAATAGCTAGTTTTGGGACTGAACAACACAGGTGTCTCATTGTGTTGGATGATGTGTGGGCGAGTGAACTTGTAGATTTTTTCAAAAATGTTCTTCCAGAAAACAAGAAAAGACATAGGATCTTGATGACCACTCGCCGCAGAGATTTGGCTAGCTGTGCAAGTGAAAGACCTTATCATCTCAAGTTTCTCACTGGAGAAGACAGTTTTCGGTTGCTGGAAAAGAGAGTTTTCGACAATGGAAGGTGTCAAAATGATTTAGTAGAGCTTGGAAAGAGTATTGCAAATAAATGTGGTGGATTACCACTTGCATTAGTGGTAATTGCTAGAGCTTTAAAAGTTATTCAGAATGTATATGAATGGAAAAGGATTGAGAACAACTTTTGGCAGTATGTTGTGAGAGAGAATGACACAACAAGCTGCTGGAAAATTGTGGAAGCCAGTTATGAAAGTTTGTCCTCTGAAATGAAAACGTGCTTCTTGTATTGCCTAGCCTTTCCTAAAGGCTACGATATTCCTGTTCAGATGTTGATTCGCTTGTGGATGGCGGAGGGGCTAATAGAGTCGAGTCCAATAAATTTCTCTTCCTCTGAGGACAAAGCAGATAATTATTTGATGGAGTTTGAGAATTTGGACCTTGTGATAGTGTCGAGAGAGATTACTGGTTCGATAAAAAGGGTTAAATGTAATGAGATGTTCTATGAGTTCTACAAGATGGAGGTGACTAAAGAAAGTGTTTTTCAAGAACTACGTCTCACACCTGATTTTCCATCGATAAATGATCCAGATACTTCTCGTCGATTGCATATTGAATCATGTGATCTGCCAGATTTTATCTCCAGAATTTCCAGTGCAGAACATGTTAGATCTTTCTTATGTTTTTCCTCGGAAGATCTAGACCAACAAACCAACTTATCCTCTCATGTTGACATTCAACTCATCCGGAAAGCATTTCCACTTCTCAGGGTTTTGAAAATTGAATCAATCAACATTCCCTCCTTGAAGAATCTTAACCTACTATTTCATCTGAGGTACATTTCTATCTCAGGTAATTTCAAGGAACTTCCTGCATTCTTTAGTAAGTTTTGGAGTTTACAATCTATTATAGTTAATACATCCCAGCCCACTCTCAACATAAAAGCAAATATATGGAACATGCCAAAGTTGACGCATTTGAAGACCAACAAACCTTCTAAATTGCCTCTCCCTTCAACCCAAAAGGGAAAAGGTTCCTCCTCCCTGCTGCAAACTCTTTCTAAGATTTCACCAGAAACTTGCAACAAAGCTGTGCTCGGACAGGCTTCGAACAGCCTCAAAAAATTGAGTATTCAAGGGAAAATAGAAGATTCTCTTCAAACTAACAGTGACCGATTCAGCACTTTTGAAGGGTTTCAAACTTTAGAAAAGTTTGAGTGCCTAGAAAATTTGAAACTGGTAAGTGATGTTCCTTGCCATACTAATGATTTTCAGTTTCCGAAAGCATTATTCAGATTTCTACTCAAGCTTAAGAAGTTAACTTTGTCAAAGACAATGTTTGATTGGAAAGATGCAACTAAACTAGGGCAACTGGAATGCCTGGAGGTCCTCAAGTTGAAAACTGATGCATTCACAGGGAAGTCATGGAATCCGGAGAAAGGTGATTTTCGAAAACTGCAGGTCTTGTGGATTCATAGCAATGACCTCCATACTTGGGAGGCTTCAGGATCTCACTTCCCCAAACTTAGGGTACTTGTTCTTGAGTGTCCTAAGCTTGAGAGTGTGCCAGCTGAACTATCTCAGTTTCAGCAGCAGAGT

>PGSC0003DMG400007462

ATGGCGGATGCTGTAGTGAATTTTCTCGTTGAGAATTTATTGCAGCTGTTAAGCGAAAATATAGATCTGATAAAAGGCGTAGACGATGAATTCAAAAATTTACTGGAAGAAGTGCAACGCCTGAAAGCATTCCTTGAGGATGCTGCTAAATTCCACAGCGAGAGCAGTTTATGGGATCAATTGGTGAAAGATATTCAAAAAATGGTACATAAATCTGAAGATGTAATTGATAAATTTCTGGTTCAAGCAAAGCTTCATCGAGATAAGAATAAAGTTGGAAGATTCTTCGATGTCCCTCATATGGCTGTAGTTAGGGCTCTTGCAGCTGAAATTAAAGGGATTCATGAGAAGGTGAAGAAACTTCGAGAAGACAACAAAGACTCATTCAAGCCTAAACCAATTCTTGACGTCCCCAAAAAAGGGCACGAAGTAACCCAGGTATACCCTTCTTTGGATGATGATGAAGTGGTTGGGTTTGATGTGGAAGCTGAGAAAGTGATCAAGCGACTTGTTGAAGGACCAGCAGAAAGTCTGAATATTATTCCAGTGGTGGGTATGCCCGGACTTGGAAAAACTACACTGGCAAGAAAAATCTACAATGATTCTACACTTTCATTTGAATTTTTCAGCATCCTTTGGGTTTATGTCGGTCGGGAATACAAAATAAAGGATATTTATCTTAGGATTCTCAAACATTTCAAAAACAGCATAGACGAGTATCTCAATGAGGATGAGGACAGATTAGCTAAGGTAATAAGTGGTTTTATGAGTAAAGGTGGAAGATGTCTCATTGTTTTAGATGATGTGTGGGAAGCAAATGCCATTGATCACGTAAAGAAAGTTTTCCCAGAAAACAAAAAGGGGCATCGAATCATGATGACCACACGTGACGGATTACTAGCTTCTTATACCAATCCAGATCCTCATAATCTGAAATTCTTGACTACAGAAGAAAGTTTTGAGTTGTTGGTAAAGAGAGTTTTTGGCAAGGGTAGTTGTCCTGATGAATTAAAAGAAAATGGACAAAAAATTGCAGGAAGCTGTGGTGGAGTACCACTTGTTGTAGTGGTAACTGCAGGAGCGTTAGCAGGTCGTTCGAATACAACTGAATGGAAAGTAGTTGAGAGAAATGTGATACAATATGCTTTTAACGTGAATAAAGATTGTGGTGTATTTGTAGAGATGAGTTACGATCGTTTGTCTCAAGAATTGCAGACGTGCCTTTTATATTCTGGTATCTTTCCTCGGGGCTTTGATATCCCTGCTTGGAAATTGATTCGCCTGTGGATTGCAGAAGGGCTAATAAAGCCCCAGCAGTCATCCACTCTTGAGGAGATAGCAGAGCTTCATTTGAATGATCTTGTTCATAGGAATTTAGTGATACTGTTGCAAAAGAGGTCTGATGGTCAAATAAAGACATGTCGTCTTCATGACATGTTACATGAGTTTTGCAGAACAAAGGCTGCTGACAAATGGCTTTTTCAAGAAATATGTACAACAGTTGATAACGCTGTTCCTTCTATACAGGATCCAGATACTTGCCGTCGATTGTGTATTCAACCATCTACTCTGAATGACTTTCTCTCCACAAAACCTTCTGCAGAACATGTCAGATCTTTCTATTGTTTTTCCTCCAAACAAAAACAAACTGACTTGTCTCCTAATGACATCAAACTCATTCACAAAGCATTTCCACTTATGAGAGTCTTGGATGTTGAGTCTCTCAAATTTCTTTTCTCCAAAGACTTTAACAATTTATTTCATTTGAGGTATGTTGCTATCTCAGGTGACTTTAAGGCCCTCCCTCCTACCTTTGGTAAATTCTGGAATTTACAAACTCTTATACTTAATACAAGTACCTCAGAGCCCACTCTTGACGTAAAAGCAGACATATGGAACATGTTACAGTTGAGGCATCTGCACACCAGCATACCTGCAAAATTGCCGTCCCCTACTATCACCACAGGTAAACCTTCTTGCCTGCAAACTCTTTCTTTAGTTGCACCAGAAAGTTGCAAGAAAGATGTACTGGCAAAGGCGTGTCATCTAAAAAAATTGAGCATTCGAGGGCAAATGGCGGCCTTTCTTGAATCCAGGGGTGGAATCAGCAATCTTGAAGAGCTAAAGTGCCTGGAACATTTGAAACTGTTGAATGATGTTCTTTACATGAATAAAACAGTTCAACTTCCTGCAGCATTCTTCAGACTTGTACGTACAGTAAAGAAGTTAACTTTGGCAAACACAAGGTTTCAATGGAGTGAGGCGGAAAAATTAGCGCAACTGGAATCCCTTGAGGTTCTAAAGTTGAAAGAAAATGCATTCATGGGTGATACTTGGAAGCCGGAGGTAGGAGGTTTTAGTAAACTCCGAGTATTGTGGATCGAGAGAGCAGAATTAGAAACTTGGGAGGTGTCGAATCTTAACTACCCCATCCTTAGGAACCTTATCCTTGTCTCTTGTGATAAGCTCAACGCTGTACCAGTTGAGCTGGCTGGTATACCTAATTTTCGCGAGATGAAGTTGGAGAACACAATCAAAGCAGTCAAATCTGCAAAAGATATACTGGAACTTAAGAAATCTCAAGAAATAATATTCAAGCTCAGCATATTCCCTCCTGAATCTGAATCCAAGGCCACACAGTAA

>PGSC0003DMG400007471

ATGTTATATACAACTTGTGAAGATCAGCCATTTCTGTTTTCTGATGTGATTCATTTGATCAATATGTTCAAAAGGGGTCCTTCTTTGGAGAATGATGAAGTGGTCGGGTTTGATGACGAAGCTAGCAAAGTGATCAAGCGACTTGTTGAAGGACCGGTGGAGAGTCTGGATATTATCCCAGTGGTGGGAATGCCCGGACTTGGAAAAACTACACTGGCAAGAAAAATCTATAATGATCCTAAGCTTTCATATGAATTTTTCAGCATCCCTTGGGTTTACGTCGGTCAGGAATACAAAATAAAGGATATTTATCTTAGGATTCTCAAATGCTTCAAAAAAAGCATAGAAGATCATCTCAATGATGATGTGGATACATTAGCTAAGGTAATAAGTGATTTTATAAATAAAGGAGGAAGATGTCTCATTGTTTTAGATGATGTGTGGGTAGCAGAAGTCATTGATGATGTAAAGAAAGTTTTCGCAGAAAACAAAAAGGGTCATCGAATCATGATGACCACGCGTGATAGATATCTAGCTACTTATGCCAATACAGAACCTCATGATCTGAAATTCTTGAATGGAAAAGAAAGTTTTGAATTGTTGGAAAAGAGAGTTTTTGGCAAGGGAAGTTGTCCTGATGAATTAGTTGAACTTGGAAAAAAATTTGCAGGAAACTGTGGTGGAGTACCACTTGCTGTAGTGGTAATTGCAGGAGCGTTAAGAGGTCGTCCGAATACAAATGATTGGGTAAGAGTTCAGAAAAATGTGGCAGAGCATTTTTATAAAAATACTCAAGCGGGCTGCTTGAAATTTGTAGAGATGAGTTACAATCGTTTGCCTCAAGAAGTACAGACGTGCTTTTTATACTGCGGTGTCTTTCCTCGAGGCTTTGATATCCCTTCTTGGAAATTGATTCGTTTGTGGATAGCGGAAGGGGTAATAAAGCCTCAACAGACATACACTCCTGAGGAGATCGCTGAGTTTTATTTGAATGATCTTGTCAATAGAAATTTAGTGATATTGATGCGAAAGAAGTCTGATGGTCAAATAAAAATATGTCGTCTTCACGACATGCTGCATGAGTTCTGCAGAACTGAGGCTAGTAACAAATGGCTTTTTCAGCAAATGCATCTAACATCTGATCAAGCTATTCCTTCCATTCAAGACAAAGATACTTGTCGTCGATTGTGCATTCAGCCCTCTATTCTGAATCAGTTTCTCCTCGATAAGCCTATTGCAGAGCATGTCAGATCTTTCTATTGCTTTTCCTCGGAACAAAAACAAATTGACTTGCCTCCTAATGACATCAAACACATCCACAAAGCATTTACACTTATCAGAGTCTTGGACGTTGAACCCGTCAAATTTCTTTCTCCAAAGATTTTTAACCAGTTATATCATTTGAGGTATGTTGCCATCTCAGGTGAATTCAAGTCCCTTCCTCCGTCCTTCGGTAAATTCTGGAATTTACAAACTCTAGTAATCAATACAAGTACCTCAGAGTCCACCCTTGAAGTAAAAGCTGACATATGGAACTTGTTACAGTTGAGGCATTTCCACACCAACATACCTGCAAAATTACCAGCACCTGCTACCACGACTGGTAAAACTTCTTGTCTACAAACTCTTTCTATGGTCGCGCCAGAAAGTTGCAAGAAAGATGTACTAGCAAAGGCTTGTCTTCTGAAAAAATTGAGCATTCGAGGGCAAATGGCATCTTTTCTTGAACCCAAAGGTGGAATCAGCAATCTTGAAGAGCTAAAATGCCTGGAACATTTGAAGCTGTTAAATGATGTTCTTTACATAAATAAAACAATTCACCTTCCTCCAGCATTCTTCAGACTCGTTCGGACAGTGAAGAAGTTAACTTTGGTAAACACAAGGTTTTCTTGGAGCGATGCGAATAAATTGGCACAGTTGGAACACCTGGAAGTCCTAAAGTTGAAGGAAAATGCATTCATAGGTGACTCTTGGAAGCCGGAGATTGGAGGTTTTAGCGCGCTCCAGGTGCTGTGGATTGAAAGGTCAGACTTAGAATCTTGGATGGCATCAAGTCATAATTTCCCAATACTTCGGCACCTTGTTCTTATCTCATGTGATAAACTCAAGGCTGTGCCACTTGGATTGGTTGAGATACATAACTTTCAAGAGATGAGACTGGACAATAGCAAAGAAGCAGTCAAATCTGCAAAAGAGATAGAAAGCAAGAAACCGAAGTTCAAGCTCACCATATTCCCTCCTGAAACTGATTCCAAGGACACACAGTGA

>PGSC0003DMG402017089

ATGGAAGCTTATACTGCTGTAATTTCTCTTCTTAGAACTCTTGATCAACCATATATTTCAAAACTCTTTCATGGTCGCACTGCTAATGCGCTTGATTCTCTTCGTGCTACAGCTGAATATTTCCATGATGTTCTTGAAAAAGCTAGCAATAAGAGTGGTGTTGACATTGAAAAAATCAAATCTTTGGAGGAAGAAATTAGAATTGTTGTTAGTGAGGCAGAGGATGTTGTTGAAATGAAAATTTCAAAAATCATCAAAGGCGACAGATGGACATTTCGAATTTTACCACACCATGATATGCTACCAGTTGTTGAAAAAATGGATACCACAAAGAAACAAGTGATGGAGATTCTTTCTCATGATGCTGATCAAATTCTTGAATTAACTGGCGCTTCATCTATGAGTGATCCAATGTTGTCAGGTCACTTGAAAGATGATATCGTGCAGGGACTTGATGGTGACTTGGAGATAATAGATAAAAGATTGAGAGGACCAACGTCGTATCTAGACATTGTCACCATATCAGGTATGGGTGGCATTGGCAAAACAACACTCGCTAAAAAAACTTATGATCATCTTCAAATCAGGCATCACTTTGACATTTTTGTTTGGGTTACAATATCTCAAGAATTTCGATATAGAAATGTATTGTTAGAAGCTTTACATTGCATTTCAAAGCAAAGAGTTAATGTGAACACAAAAGATTATGATAAGATGAATGACAGTGAGTTAGCTGATCTGGTGCAAAAGAGTCTAAAGGGTCCTAGATACCTTGTTGTTGTTGATGATATTTGGAGTAGGGATGTTTGGGATAGCATATCACAAATATTTCCTAATCGCAACAATGGGAGTCGAGTCTTATTGACTACTAGGGAAACTGATGTAGCAATCTATGCAAATACTAGTATCCATCATAAGATGAACCTCTTGGATTTAGATAATAGTTGGAAGTTACTCCGTGATAAGGTGTTTGGACTAGAACACGATCATCCTCCCGAATTGGAAGAAATTGGAAAAAAATGCTTAGGAGTGCTCGGTTTGAGTTACCATCACTTGCCTAATCGACTCAAACCATGCTTTCTTTCTCTGGGTGATTTCCCAGAGGATTTTCAAGTTGATACTCGGAGATTGATCCAATTATGGATCGCAGAAGGTTTCATAAGGACGTCTTCCGGAAGTCGTGAAAGCTTGGAGGAAGTGGCTGAACATTATTTGGAGGATCTTATCAGCAGGAACTTGATAATGGCTAGAAAAAGAGATTCAATGGTGAGGGTAAAAGTATGCGGAATACATGATCTGCTGCGTGAGTTCTGTTTGATAGAAGCTGAAATGACAAAGTTTATGCATGTTGTGAGAACTCCATTGTCACCAGCACAAAAGCCTAATTTTCGTCGCTTCAGTATTCAAGAGTATATTGAGGATCCTTATAAGCTCTTACCCCGTGTAGCCAGATCTATCTACTGCTTTTCTTACTTGGGTCTACCTTATGATCCCCATATTAAGCTTCTCCCTAGATTGCCCATCTACCGTCATGATCCTAGATTACTTGGATTTTTCTCTTGTTTCAACCATCTCAGGGTATTGGCCATCGTCCTTAACTGCTTCCACTCATTTCCACTTGTGATTACAAAGTTATTTCATTTGAGATATCTCCAAGTTCGATGTTTTGGAAATATTCCTAAATCAATCTCAAAGCTTTTGAATTTGCAAACTCTAATTTGTGATGGTAGTTCTTTAGATATAACTTTACCTAGGAAGATATGGATGTTGAAGAACTTGAGGTATATACGTTTAGGGGCAGCCACTTATTTACCGAGTCCTAGAATAGGTAACAATCTTGTGACAGGGATGCCAAATCTAGAGGAACTTTACGGTATTTGTTACTCTAGTTGTACAAATGAAGTCATTTCTAGCACTCCCAATCTAAAGAGATTGATCATTCGTGCACATTTTATCGCACTAATGAATTTTCCGGGTCGTCCCTTCGATATGTCCAGCTTGACAAAACTTGAGACATTCAAGCTTTACTGGGAAGTTTATTTGGAAGACCCCATCAAGATATTTGTTTTTCCAGAATCACTTAGAAGATTGTCTTTAACTTGGTGTTATGAAGCTCTTACCCCCTGTAGCCAGATCTATCTACTGCTTTTCCTAACTTGGGTCTACCTTAATGATGATGTATGGAAATTGAGTGACAAAGACATATTCAGAAGCTTGAAGTTGTTGTTACTGAGCTACCCAAATCTTAAGGGTTGGGAAGCAAGCAGCGATAACTTCCCTAATCTAAAACGCCTTGTTCTGAAGAAATGCGAGGACCTGCAAGAAATTCCAACAGATTTCGTGGAAATTTGTACTTTGGAATCGATTGAGTTACATGATTGCACCACTACTGCTGAGGATTCTGTCAGAAATATCGAACAAGAACGAGAGGACATGGGATATAATATCCTTAAGGTCTACATCCATAACAGCCACAGTAAGTTATAA

>PGSC0003DMG400017085

ATGGCAAATGAAGCTCTGAACTTTTTCGTGGAGAACTTGTTGCAGCTGTTAAGTGAAAATGTGGTGTTAATAAAAGGCGTGGAGGACGAATTCAAAAATTTATTGGATCAAGTGCAAAGCCTGAAAGCATTCGTTGACGATGCTGCTAAATTCCACAGAGATAGCAGTGTATGGGATGAATTGGTGAAAGATATTCAAAAAATGATATACAAAGCTGAAGATGTAATTGATAAATTTATAGTTCAAGCAAAGCTGCACACAGAAGAGAAAAAAGTGAAATCCTTTAGATCTCTTGCAGATGAGATCAAAGGGATCAGTGAGAAAGTTAAGGAATTTCGAGAAGAAAGTAAACAGTCATTCCAGCCCAGACCAATTGTTGAGCACCCTAAAAGAGGGCGCGAAATAACCCAGATATGCCTTGTTAAAGGACGGGAAGAATCACTTGATATTATCCCAGTGGTGGGTATGCCCGGACTTGGAAAAACAACACTGGCAAGAAAAATCTACAATGATCCTAAGCTTTCATATGAATTTCATAGCATCGTTTGGGTTAATGTTGGTATGGAATACAAAATAAAGGATATTTATCTTAGGATTCTCAAATTCTTCACAAAACGCATAGAAGATCATCTCAATGATGATGAGGACACATTAGCTAATATGATAAGTGCTTTTATCAGTAATCGAGGTAGATGTCTCATTATTTTGGATGATGTGTGGGAAGAAAATGTCATTGATCACGTAAAGAAAGTTTTTGCAGAAAACAAAATGGGGCATCGAATCGTGATAACCACGCGTGACAGATATCTAGCTGCTTATGTCAATCCAGAAGCTCATCATCTGAAATTCTTGACTCAAGAAGAAAGTTTTGAATTGTTGGTAAGGAGAGTTTTTGGCAAGGAAAGTTGTCCCGTTGAATTAGTACACTTTGGAAAAAAATTGCAGTACACTGTGGTGGAGTACCACTTGTTGATGGTAATTGCAGGAGCATTAAGAGGTCGTTCGACTACAAATGATTGGCTAAGAGTTGAGAGAAATATGGCACAACATCTTTATAGCAATTATAATAGAGATAGTGGTGTATTTTTAGAGATGAGTTACGATTGTTTGCCTCAAGAAGTGCAAACGTGCTTTTTATATTGTGGTATCTTTCCTCGGGAGTTTGATATCCCTGCTTGGAAATTGATTCGCATGTGGATTGCGGAAGGGTTAATGAAGCCCGAGCAGTCATCCACTCTTGAGGAGATAGCAGAGCATCATTTGAACGATCTTGTTAATAGGAATTTAGTGATATTGTTGCAAAAGAGGTCTGATGGTCAAATAAAGACATGTCGTCTTCATGGCATGTTACACGAGTTTTGCAGAAGAAAGGCTGCTAACAAATGGCTTTTTCAAGAAATATGTACAGCAGCTGATAACTCTCTTCCTTCTATACAGGACTCAGATACTTGCCGTCGATTGTGTATTCAACCATCTACTCTGAATGACTTTCTCTCCACAAAACCTTCTGCAGAACATGTCAGATCTTTCTATTGTTTTCCTCAAAACAAAAACAAACTGAATTGTCTCGTAATGAAATCAAACTCCTTCACAAAGCATTTCCACTTAAGGGTCTTGGATGTTGAATCTCTCAAATTTCTTTTCTCCAAAGACTTTAACCAATTATTTCATTTGAGGTATATTGCTATCTCGGGATACATTAAAGCCCTTCCTCCGCCCTTTGGTAATTTCTGGAATTTACAAACTCTTATACTTAATACAAGTACCTCAGAGCCCACTCTTGACGTAAAAGCAGACATATGTAACTTGTTACAGTTGAGGCATCTGCACACCAGCATACCAGCAAAATTGCCATCCCCGACTGTCACAACAGGTAAACCTTCTTCTCTACAAACTCTTTCTATGGTTGCACCAGAAAGTTGCAAGAAAGATGTGCTGGCAAAGGCGTGTCATCTAAAAAAATTGAGCATTCGAGGGCAAATGGCGGCCTTTCTTGAATCCAGGGGTGGAATCAGCAATCTTGAAGAGCTAAAGTGCCTGGAACGTTTGAAACTGTTGAATGATGCTCTTTACATGAATAAAACAGTTCAACTTCCTGCAGCATTCTTCAGACTTGTACGTACAGTAAAGAAGTTAACTTTGGCAAACACAAGGTTTCAATGGAGTGAGGTGGAAAAATTAGCACAACTGGAAGAAAATGCATTCATGGGTGATACTTGGAAGCCGGAGGTAGGAGGTTTTAGCAATCTGCAGGTATTGTGGATCGAGAGGGCAGAATTAGAAACTTGGGAGGTGTCGAATCTTAACTACCCCATCCTTAGGAACCTTGTTCTTGTCTCTTGTGATAAGCTCAACGCTATACCATTTGAGCTGGCTGATATAACTAACCTTTGCGAGATGAAGCTGGATAACACAATCAAAGCGGTCAAATCTGCAAAAGATATACTGGAACGTAAGAAATGTCAAAAAATAATATTCAAGCTCAGCATATTCCCTCCTGAAGCTGAATCCAATGCCACACAGTAA

>PGSC0003DMG400017086

ATGGCAAATGAAGCTCTGAACTTTTTCGTGGAGAACTTGTTGCAGCTGTTAAGTGAAAATGTGGTGTTAATAAAAGGCGTGGAGGACGAATTCAAAAATTTATTGGATCAAGTGCAAAGCCTGAAAGCATTCGTTGACGATGCTGCTAAATTCCACAGAGATAGCAGTGTATGGGATGAATTGGTGAAAGATATTCAAAAAATGATATACAAAGCTGAAGATGTAATTGATAAATTTATAGTTCAAGCAAAGCTGCACACAGAAGAGAAAAAAGTGAAATCCTTTAGATCTCTTGCAGATGAGATCAAAGGGATCAGTGAGAAAGTTAAGGAATTTCGAGAAGAAAGTAAACAGTCATTCCAGCCCAGACCAATTGTTGAGCACCCTAAAAGAGGGCGCGAAATAACCCAGATATGCCTTGTTAAAGGACGGGAAGAATCACTTGATATTATCCCAGTGGTGGGTATGCCCGGACTTGGAAAAACAACACTGGCAAGAAAAATCTACAATGATCCTAAGCTTTCATATGAATTTCATAGCATCGTTTGGGTTAATGTTGGTATGGAATACAAAATAAAGGATATTTATCTTAGGATTCTCAAATTCTTCACAAAACGCATAGAAGATCATCTCAATGATGATGAGGACACATTAGCTAATATGATAAGTGCTTTTATCAGTAATCGAGGTAGATGTCTCATTATTTTGGATGATGTGTGGGAAGAAAATGTCATTGATCACGTAAAGAAAGTTTTTGCAGAAAACAAAATGGGGCATCGAATCGTGATAACCACGCGTGACAGATATCTAGCTGCTTATGTCAATCCAGAAGCTCATCATCTGAAATTCTTGACTCAAGAAGAAAGTTTTGAATTGTTGGTAAGGAGAGTTTTTGGCAAGGAAAGTTGTCCCGTTGAATTAGTACACTTTGGAAAAAAATTGCAGTACACTGTGGTGGAGTACCACTTGTTGGTAATGGTAATTGCAGGAGCATTAAGAGGTCGTTCGACTACAAATGATTGGCTAAGAGTTGAGAGAAATATGGCACAACATCTTTATAGCAATTATAATAGAGATAGTGGTGTATTTTTAGAGATGAGTTACGATTGTTTGCCTCAAGAAGTGCAAACGTGCTTTTTATATTGTGGTATCTTTCCTCGGGAGTTTGATATCCCTGCTTGGAAATTGATTCGCATGTGGATTGCGGAAGGGTTAATGAAGCCCGAGCAGTCATCCACTCTTGAGGAGATAGCAGAGCATCATTTGAACGATCTTGTTAATAGGAATTTAGTGATATTGTTGCAAAAGAGGTCTGATGGTCAAATAAAGACATGTCGTCTTCATGGCATGTTACACGAGTTTTGCAGAAGAAAGGCTGCTAACAAATGGCTTTTTCAAGAAATATGTACAGCAGCTGATAACTCTCTTCCTTCTATACAGGACTCAGATACTTGCCGTCGATTGTGTATTCAACCATCTACTCTGAATGACTTTCTCTCCACAAAACCTTCTGCAGAACATGTCAGATCTTTCTATTGTTTTCCTCAAAACAAAAACAAACTGAATTGTCTCGTAATGAAATCAAACTCCTTCACAAAGCATTTCCACTTAAGGGTCTTGGATGTTGAATCTCTCAAATTTCTTTTCTCCAAAGACTTTAACCAATTATTTCATTTGAGGTATATTGCTATCTCGGGATACATTAAAGCCCTTCCTCCGCCCTTTGGTAATTTCTGGAATTTACAAACTCTTATACTTAATACAAGTACCTCAGAGCCCACTCTTGACGTAAAAGCAGACATATGTAACTTGTTACAGTTGAGGCATCTGCACACCAGCATACCAGCAAAATTGCCATCCCCGACTGTCACAACAGGTAAACCTTCTTCTCTACAAACTCTTTCTATGGTTGCACCAGAAAGTTGCAAGAAAGATGTGCTGGCAAAGGCGTGTCATCTAAAAAAATTGAGCATTCGAGGGCAAATGGCGGCCTTTCTTGAATCCAGGGGTGGAATCAGCAATCTTGAAGAGCTAAAGTGCCTGGAACGTTTGAAACTGTTGAATGATGCTCTTTACATGAATAAAACAGTTCAACTTCCTGCAGCATTCTTCAGACTTGTACGTACAGTAAAGAAGTTAACTTTGGCAAACACAAGGTTTCAATGGAGTGAGGTGGAAAAATTAGCACAACTGGAAGAAAATGCATTCATGGGTGATACTTGGAAGCCGGAGGTAGGAGGTTTTAGCAATCTGCAGGTATTGTGGATCGAGAGGGCAGAATTAGAAACTTGGGAGGTGTCGAATCTTAACTACCCCATCCTTAGGAACCTTGTTCTTGTCTCTTGTGATAAGCTCAACGCTATACCATTTGAGCTGGCTGATATAACTAACCTTTGCGAGATGAAGCTGGATAACACAATCAAAGCGGTCAAATCTGCAAAAGATATACTGGAACGTAAGAAATGTCAAAAAATAATATTCAAGCTCAGCATATTCCCTCCTGAAGCTGAATCCAATGCCACACAGTAA

>PGSC0003DMG400005577

ATGGCAAATGAAGCTCTGAACTTTTTCGTGGAGAACTTGTTGCAGCTGTTGAGTGAAAATGTGGTGTTAATAAAAGGCGTAGAGGACGAATTCAAAAATTTATTGGAAGAAGTGCAACGCCTGAAAGCATTCGTTGACGATGCTGCTAAATTCCACAGCGATAGCAGTTTATAGGATGAATTGGTGAAAGATATTCAAAAAATGATACACAAAGCTGAAGATGTAATTGATAAATTTCTAGTTCAAGCAAAGCTGCACAGAGACGAGAAAAAAGTTGGAAGATTCTTCGATGTGAGTCATCTCGAAACAATTAGATCTCTTGCAGATGAGATCAAAGGGATCAGTGACAAAGTTAAGGAATTTCGAGAAGAAAGTAAACAGTCATTCCAGCCCAGACCAATTCTTGAGCACCCTAAAATAGGGCACGAAATAACCCAGGTATGCTAAAATCCCCATATATTTTACCAGAGTTTGTTTAAAATTCTTTTAAATATTTTCAGTTGTTACTGCTCTATAGTCATACTCTTTTCTTTTTTGCAAAAATTATCCGCACTGTTTTTTAATGCATTCTGTTTGATTATTATGTTAAAAAGGGTCTTTTCTTGGACAATGATGAAGTGGTCGGGTTTAATGAGGAAGCTAACAAAAGTGATCAAGTGACTTGTTAAAGGACGGGAAGAATCACTTGATATTATCCCAGTGGTGGGTATGCCTGGACTTGGAAAAACAACACTGGCAAGAAAAATCTACAATGATCCTAAGCTTTCATATGAATTTCATAGCATCGTTTGGGTTAATGTCGGTATGGAATACAAAATAAAGGATATTTATCTTAGGATTCTCAAATTCTTCACAAAACGCATAGAAGATCATCTCAATGATGATGAGGACACATTAGCTAATATGATAAGTGCTTTTATCAGTAATCGAGGTAGATGTCTCATTATTTTGGATGATGTGTGGGAAGAAAATGTCATTGATCACGTAAAGAAAGTTTTTGCAGAAAACAAAATGGGGCATCGAATCGTGATAACCACGCGTGACAGATATCTAGCTTCTTATGTCAATTCAGAAGCTCATCATCTGAAATTCTTGACTCAAGAAGAAAGTTTTGAATTGTTGGTAAGGAGAGCTTTTGGCAAGGAAAGTTGTCCCGTTGAATTAGTACACTTTGGAAAAAAAAATTGCAGCTCACTGAGTGGAGTACCACTTGTTCTAATGGTTATTGCAGGAGGATTAAGAGGTAGTTCGACTACAAATGATTGGCTAAGAGTTGAGAGAACTATGGCACATCTTTTTATCAATTATAATGTAGGTTGTGGTGTATTTGTAGACATGAGTTACGGTCGTTTGCCTCAAGAAGTGCAGATGTGCTTTTTATATTGTGGTATCTTTCCTCGGGGCTTTGATATCCCTGCTTGGAAATTGATTCGCATGTGGATTGCGGAAGGGTTAATAAAGCCCCAGCAGTCATACACTCTTGAGGAGATAGCAGAGCATCATTTGAACGTTCTTGTTGATAGGAATTTAGTGATATTGTTGCAAAAGAGGTCTGATGGTCAAATAAAGACATGTCGTCTTCATGGCATGTTACACGAGTTTTGCAGAAGAAAGGCTGCTAACAAATGGCTTTTTCAAGAAATATGTACATCAGCTGATAATTCTCTTCCTTCTATACAGGACTCAGATACTTGCCGTCGATTATGTATTCAACCATCTACTCTGTGTGACTCTCTCCACAAAACCTTCTGCAGAACATGTCAGGTCTTTCTACTGTTTTTCCTCAAAACAAAAACAAACAATCAAACTCCAACTATAAGGGTCTTGGATGTTGAATCTCTCAAATTTCTTTTCTCCAAAGACTTTAACAATTTATTTCATTTGAGGTATATTGCTATCTCGGGTGACATTAAAGCCCTTCCTCCGCCCTTTGGTAATTTCTGGAATTTACAAACTGTTATACTTAATACAAGTACCTCAGAGCCCACTCTCGACGTAAAAGCAGACATATGGAACTTGTTACAGTTGAGGCATTTCCACACCAACATACCTGCAAAATTGCCATCTCCGACTATCACGACAGGTAAACCTTCTTGTCTACAAACTCTATCAAACTACACAACAATGGTGAGCATCTAA

>PGSC0003DMG400015872

ATGGCAGATGCAGTTGTGGATTTTCTGGTAGAGAATTTGTTGCAGCTATTAACTGAAAATGTGAAGCTAATTGGAAGTGCAAAAGGAGAATTGGACAATCTACTTAAAGTTGCTCAACAACTCAAAGCATTTTTAGATGATGCTGCAAAATACGGATACACGAATAGTGAGCAGTGGAAAGTTTTGGTGATAGAAATACATAAAACTGTACACAGAGCTGAAGATGCCATTGATAAGTTTCTGGTTCAAGCAAAGCTGCATCAAGAAAGAAACACAATGGGAAGGATATTTGATTGGCCACGCAATCTGATTAAAGTTAGGAATCTTGCTGCTGAGATTAAAGGAATAAATGATCAAGTCAAAGAACTTCGTAGCAGCAATCAAGCACTTCAGGCAACCCATGTTATTGAGCTCCCTAAAAGAGGGGAAGTAACTCAGGTAATGGACAGTGTCGATATTATCCCAGTGGTGGGGATGCCCGGACTTGGAAAAACTACTCTGGCAAGAAAAATCTATAATGATTCAAAGCTTACATATGAATTTTACAGCATTGTTTGGGTTTACGTTGGTCAGGAATGCAAAGCAAAGGATATTTATCTTAGGATTCTTAAATTCTTCAAAAAAAACATAGAAGATCATCTCAATGATGATGTGGACACATTGGCTAAGGCAATAGGTGGTTATATCAAGAAAGGAGGTAGATGTCTCATTGTTTTAGATGATGTGTGGGAAGACGAAGTCATTGATCATGCAAGGAAAGTTTTCGCAGAAAACAAAAAGGGTCATCGGATTATGATGACCACACGTGACTTACGTGTTGCTAAATTTGCCAATCGAGAACCTCATGAGCTGAAATTCTTGGAGAAAGAAGAAAGTTTTGAATTGTTGGTAATGAGAGTTTTTGGCAAGGGAAGTTGTCCAAATGAATTAGTGGTAATTGGGAAAAAAATTGCAAAAAAATGTGGTGGAGTACCACTTGTAGTAGTGGTAATTGCAGGAGCATTAAGAGGTCGTTTGGACAAAAAAGATTGGGAAAGAGTTGACAGAAATGTAGTGCAACATTTAGGTGAGCATACCGAGGATAGCTGCCTGAAATTTGTGAAGATGAGTTATGATCATTTGCCCAGAGAAGTGCAGATGTGCTTCTTGTATTGTGGTGTCTTTCCTCGAGGCTTTGATATCCCTTGTTGGAAATTGATTCGTTTGTGGATAGCAGAGGGGTTGATAAAGCCGCAGCCTGAGTCCACTCTAGAGGAGATAGCAGAGTTTTATTTGACAGATCTTCTCAACAGAAATTTAGTAATAATAATGCAAAAGAGGTCTGATGGTCAAATAAAACATGTCGTCTTCACGACATGTTGTATCAGTTCTGCAAAAAGGAGGCTAGTAACAAATGGCTTTTTCAAGAACCTGATCAATCTAAACTGGACCCAGATACTTGCCGTCGATTGTGTATTCAACCATCTAATCTGTCTGACTTTCTCTCCACAGTACCTGTTGCAGAACATGTCAGATCTTTCTATTGTTTTTCCTCAAAACAAAAACCAATTGACTTGTCTGCTAATGAAATCAAACTCATTCACAAAGCCTTTCCACTTAGTCTTGGATGTTGAATCTCTCAAGTTTATTTTCTCCAAAGACTTTAACCAATTATATCATTTGAGGTATATTGCTATCTCAGGTGACTTTAAGGCCCTCCCTCCTACCTTTGGTAAATTCTGGAATTTACAAACTCTAATACTTAATACAAGTACCTTAGAGCCCACTCTTGAAGTAAAAGCAGAAATATGGAACTTGTTACAGTTGAGGCATCTCCACACCAACATACCTGCAAAATTGCCATCCCCGACTACCACCACAGGTAAACCTTCTTGTCTACAGACTCTTTCTATGGTTGCGCCAGAAAGTTGTGAGAAAGATGTGCTAGCAAAGGCGTGTAATGTTAGAAAATTGAGCATTCGAGGGCAAATGGCGGCTTTTCTTGGTGCTTACAAGGGTGGTATCAACAATCTTGAAGAGCTACAGTGTCTGGAACATTTGAAATTGTTGAATGATGTTCTTTTCATAAATAAAACACTTCACCTTCCACTAGCATTCTCCAAACTGGTACGTACAGTGAAGAAGTTAACTCTGACAAACACAAGGTTTTCTTGGAGTGAGGCGGATAAATTGGGGAAGTTGGAATCTCTTGAGGTCCTAAAGTTTAAAGAAAATGCATTCACGGGTGATTTCTGGAAGCCAAAGAGTGGATTTAGCGCACTCCAAGTCTTGTGGATTGAAAGGTCAGAATTAGAATCTTGGGAGGCTTCGGTTATTAACTTCCCCGTGCTTAGGCAACTTGTTCTTATCTCTTGTGATAAGCTCGATGATGTGCCACTTGAGCTGGCTGATATCCCTAGTCTTCAAGAGATGAGGCTGGATAACACAAGCAAAGCAGTAAACTCTGCAAAAATTGTTCGGGATAGCAAGACATCTAAAAGCATGAAACTCAAGCTAAGCATATTCCCTCCTGAAAATGAATCCAAGGTTGCACAGTGA

>PGSC0003DMG401015877

ATGGCAGCTTATACTGCTGTAATTTCTCTTCTTCAAACTGTTGATCAACCAAATATTGCAGAACTCTTTCATGATCACAGTGCTGAAATGCTCGATTCTCTTCGTGCTACAGCTGAATATTTTCAAGATGTTCTTGAAAACACTAGCGATGAGAAAATCAAATCTTTGGAGGAAGATATTAGAGTTGTTGTTAGTGAGGCAGAGGATGTTGTTGAAATGAAGATTTCTGAAATCATCAAAGAAGAAAGCTGGACATTTGGAATTTTACAACACCAGGATATGCTACCAGTTGTTGAAAAAATGGATACGACAAAGAAGCAAGTGATGGAGATTCTTTCTCATGATGCTGATCAAATTCTTGAATTAACCGCGGATTCCTTGATTGACGCTTCTTCTATGAGTGATCCAATGCTGTCAGATCACTTGAAAGATGATATTGTGCAGGGACTTGATGGTGACTTGGAGAAAATAGATAAGAGATTGAGAGGACCAACGTCGTATCTAGACATTGTCACCATATCAGGTATGGGTGGCATTGGCAAAACAACACTCGCTAAAAAAGCTTATGATCATCTTCCAATCAGGTGTCACTTTGACATTTTTGTTTGGGTTACAATATCTCAAGAATTTCGATATAGAAATGTATTGTTAGAAGCTTTACATTGCATTTCAAAGCAAAGAGTTAATGTGAACACAAAAGATTATGTTAAGATGAATGCTAGTGAGTTAGCTGATCTGGTGCAAAAGAGTCTAAAGGGTCCAAGATACCTTGTTGTCGTTGATGATATTTGGAGTAGGGATGTTTGGGATAGCATATCACAAATATTTCCTAATCGCAACAATGGGAGTCGAGTCTTATTGACTACTAGGGAAACTGATGTAGCAATATATGCAACTACTAGTAGCCCTCATAAGATGGACCTCTTGAATTTAGATAACAGTTGGAAGTTACTTCGTGTTAAGGTGTTTGGACTAGAACACGATCATCCTGCTGAGTTGGAAGAAATTGGAAAAACAATAGCAGGAAAATGCCAAGGACTTCCCTTAATAATTTCAGTGATTGCAGGGCCTCTCTCTAAAACGGTGCCCAAGACATTAGAAAGTTGGAAGTATGTTGCCAAAACATTAAGTGAAATCATTGCTAGTCATCCAAATAAATGCTTAGGAGTGCTCGGTTTGAGTTACCATCAGTTGCCTAATCGACTCAAACCGTGCTTTCTTTCTATGGGTGATTTCCCAGAGGATTTTCAAGTTGATACTCGGAGATTGTTCCAATTATGGATCGCAGAAGGCTTCATAAGGGCGTCTGCTGGAAGTCGTGAAAGTTTGGAGGAAGTGGCTGAACATTATTTGGAGGATCTTATCAACAGGAACTTGATAATGGCTAGAAAAAAGAGATTCAATGGTGAGGTAAAAGCATGCGGAATACATGATCTGCTGCGTGAGTTCTGTTTGATAGAAGCTGAAATGACAAAGTTTATGCATGTTGTGAGAACTCCATTTTCACCAGCACAAAAGCCTAATTTTCGTCGCTTCAGTATTCAAAAGCGTATTGAGGATGCTTATAAGCTCTTACCCCCTGTAGCCAGATCTATCTACTGCTTTACTTACTTGGATCTACCTTATGAACCCCGTATTAAGATTCTCCCTAGATTGCCCATCTACCGTCATGATCCTATAATACATGGATTTTTCTCTTGTTTCAACCATCTCAGGGTATTGGCCATCTTTCCTTCCTCGTTCAGCACATTTCCACTTGTGATTATGAAGTTATTTCATTTGAGATATCTCCAATTTCGATGTTTTGACAACAATATTCCTGAATCAATCTCAGAGCTTCTGAATTTGCAAACTCTAATTTGTGAGGGTTTTCCTTTAGAGATAACTTTACCTAGGAAGATATGGATGTTGAAGAACTTGAGGTATATACGTATAGGGGGAGCCACTTATTTACCAAGTCCTAGAATAGATAAGAATCTTGTGACAGGGATGCCAAGTCTTGAGGAACTTTACGGTATTTGTTACTCTAGTTGTACAAATGAAGTCATTTCTAGCACTCCCAATCTAAAGAGATTGATCATTCGTGTACATTGGAGCATACTAAAGAATTTTCCGGATCGTCCCTTGGATATGTCCAGCTTGACAAAAACTTGAGACATTCAAGCTTTACTGGGAAGGTTATTTGGAAGACCCCATCAAGATATTTGTTTTTCCAAAATCAATAGAAGAGTGTCTTTAACTAGGTGTGAAAAGTTTATTTGGGAAGAGATATCATCAACTTTTATCATGTTGCCACATCTTGAAGAGCTCAAACTTAAATGTTGTCGAGCCAAAGATGATGTATGGAGATTGAGTGACAAAGACATATTCAGAAGCTTGAAGTTGTTGTTACTGAGCGAGCTAAATCTTAAGGGTTGGGAAGCAAGCAGCGATAACTTCCCTAATCTTAAACGCCTTGTTCTGAAGAAATGCAAGGACCTGCAAAAAATTCCAACAGATTTCGTGGAAATTTGTACTTTGGAATCGATTGAGTTACATGATTGCACCACTACTGCTGAGGATTCTGTCAGAAATATCGAACAAGAACGAGAGGACATGGGATATAATATCGTTAAGGTCTACATCCATAACAGCCACAGTAAGTTATAA

>PGSC0003DMG403015877

ATGGCAGCTTATACTGCTGTAATTTCTCTTCTTCAAACTCTTGATCAACCAAATATTTCAGAACTCTTTCATGATCACAGTACTGAAATGCTCGATTCTCTTCGGGCTACAGCTGAATATTTTCAACATGTTCTTGAAAACACTAGCAATGAGAAAATTAAATCTTTTGAGGAAGATATTAGAGTTGTTGTTAGTGAGGCAGAGGATGTTGTTGAAATGAAGATTTCTGAAATCATCAAAGAAGAAAGCTGGACATTTGGAATTTTACAACACCAGGATATGCTACCAGTTGTTGAAAAAATGGATACGACAAAGAAACAAGTGATGGAGATTCTTTCTCATGATGCTGATCAAATTCTTGAATTAACCGCGGATTCCTTGATTGGCGCTTCTTCTATGAGTGATCCAATGCTGTCAGATCAGTTGAAAGATGATATCGTGAAGGGACTTGATGGTGACTTGGAGATAATAGATAAAAGATTGAGAGGACCAATGTCGTATCTAGACATTGTCACCATATCAGGTATGGGTGGCATTGGCAAAACAACACTCGCTAAAAAAGCTTATGATCATCTTCCAATCAGGTATCACTTTGACTATTTTTGTTTGGGTTACAATATCTCAAGAATTTCGATAAGAAATGTATTGTTAGAAGCTTTGCATTGCATTTCAAAGAAAAGTCCAAAAGATTATGATGAGATGAATGACAGTGAGTTAGCTCATCTGGTGCGAAAGAGTCTAAAGGGTCCTAGATACCTTGTTGTTGTTGATGATATTTGGAGTAGGGATGCTTGGGAGAGCATATCACAAATATTTCCGAATCGCAACAATGGGAGTCGAGTCTTATTGACTACTAGGGAAACTGATGTAGCAATACTTGCAAATATTAGTAGCCATCATAAGATGAACTTCTTGGATTTAGATAATAGTTGGAAGTTACTCCGTGATAAGGTGTTTGGACTAGAACACGATCATCCTCCTGAGTTGGAAAGAAATTAGGAAAAAAAATAGCAAGAAAAATGTCAAGGACTTCCCTTAATAATTTCAGTGATTGCAGGGCATCTCTCTAAAACGGTGCCCAAGACATTAGAAAGTTGGAAGTATGTTGCCAAAAACCTTAAGAGTGAAATCATTGCTAGTCATCCAAATAAATGCTTAGGAGTGCTTGGTTTGAGTTACCATCACTTGCCTAATCGACTCAAACCGTGCTTTCTTTCTATGGGTGATTTCCCAGAGGATTTTCAAGTTGATACTTGGAGATTGATCCAATTATGGATCGCAGAAGGTTTCATAAGGACGTCTTCTGGACGTCATGAAAGCTTGGAAGAAGTGGCAAAACATTATTTGGAGGATCTTATCAGCAGGAACTTGATAATGGCTAGAAAAAAAAGATTCAATGGTGAGGTAAAAGTATGCGGAATACATGATCTGCTGCGTGAGTTCTGTTTGATAGAAGCTGAAATGACAAAGTTTATGCATGTTGTGAGAACTCCATTTTCACTAGCACAAAAGCCTAATTTTCGTCGCTTCAGTATTCAAAAGTATATTAGTCATAAGCTCTTACCCCCTGTAGCCAGATCTATCCACTGCTTTTCTTCCTTGGATCTACCTTTTGAATCCCGTATTCATGGATTTTTCTCTAGTTTCAACCATCTCAGGGTATTGGCCATCTTCTCTGCCTTGTTCGTCTCATTTCCACTTGTGATTAGAAAGTTATTTCATTTGAGATATCTCCAAGTTCAATGTTCTGGCAATATTCCTGAATCAATCTCAGAGCTTCAAAATTTGCAAACTCTAATTTCTGATGCTTATTCATTGGATATGACTTTACCTCGGAATATATGGATGATGAAGAACTTGAGGTATATACGTTTAAGGGAAGCCGGTTATTTACCAAGTCCTAGAATAGATAACAATCTTGTGACAGGGATGCCAAATCTAGAGGAATTTTNNNNNNNNNNNNNNNNNNNNNNNNNNNNNNNNNNNNNNNNNNNNNNNNNNNNNNNNNNNNNNNNNNNNNNNNNNNNNNNNNNNNNNNNNNNNTTTGTTACTCTAGTTGTACAAATGAAGTCTTTTCTAGCATCCCCAATTAAGAGATTGACCATTCACGCACCTTTTAGAGTAGGATACATTATTCGATGTCTTCTCTTGGATATGTCGAGCTTGACAAAACTTGAGGCATTCAAGCTTTCCTGGAGATATAATTATGAAAATCCCATCAAAAGATTTGTTTTTCCGGAATCACTTAGAAGATTGTCTTTAACTAGGTGTTTTAAGTTTATTTGGGAAGAGATATCATCAACTTTTATCATGTTGCCACATCTTGAAGAGCTCAAACTTAAACATTGTCTAGCCGATGATGATGTATGGAAATTGAGTGACAAAGACATATTCAAAAGCTTGAAGTTGTTGTTACTGAGCGATGTAAATCTTAAGTGTTGGGAAGCTAGAAGTGATAACTTCCCTAATCTAAAACGCCTTGTTCTGAAGAAATGCAAGGACCTGAAAAAAATTCCAACAAATTTTGGGGAAATTTATTCTTTGGAATTGATTGAGTTACATGATTGCACCACTACTGCTAAGGCTTCTGTCAGAAAGATCAAACAAGAACAAGACGACTTGGATAATAATATCCTTAAGGTCTACATCCATAACAGCTACAGTAAGTTATAA

>PGSC0003DMG401026842

ATGGCAGTTGTGAATTTTCTGGTAGAGAATTTGTTGCAGCTATTAACTGAAAATGTGAAGCTAATTGGAAGTGCAAAAGGAGGATTGGACAATCTACTTAAAGTTGCTCAACAACTCAAAGCATTTTTAGATGATGCTGCAAAATACGGATACACGAATAGTGAGCAGTGGAAAGTTTTGGTGATAGAAATTCATAAAACTGTGCACAGAGCTGAAGATGCCATTGATAAATTTCTGGTTCAAGCAAAGCTGCATCAAGATAGAAATACGATGGAAAGGATATTCCATTGGCCAAGTCTTCTGATTAAAGTTAAGAATCTTGCTGCTGAGATTAAAGGAATAAATGATCAAGTCAAAGAACTTCGTAGCAGCAATCAAGCACTTCAGGCAACCCCTGTTCTTGAGCTCCCTAAAAAAGGGGAAGTAACTCAGGTAAAGGGTCCTTCTTTGGAGAATGATGCAGTGGTCGGGTTTGATAAAGAAGCTAACACAGTGATCCAGCGACTTGTTGAAGGACCGATGGATAGTGTCGATATTATCCCAGTGGTGGGGATGCTCGGACTTGGAAAAACTACACTGGCAAGAAAAATCTATAATGATTCTAAGCTTACATATGAATTTTACAGCATTGTTTGGGTTTACGTCAGTAAGGAATGCAAAGCAAAGGATATTTATCTTAGGACTCTTAAATTCTTCAAAAAAAGACATAGAAGATCATCTCAAAATGATGATGTGGACGCATTGGCTAAGGCAATAGGTGGTTATATCAAGAAAGGAGGTAGATGTCTCATTGTTTTAGAAGATGTGTGGGAAGACGAAGTCATTGATCACGTAATGAAAGTTTTCGCAGAAAACAAAAAGGGTCATCGGATTATGATGACCACACGTGACTCACGTGTTGCTAAATTTGCCAATCTAGAACCTCATGAGCTGAAATTCTTGAAGAAAGAAGAAACTTTTGAATTGTTGGTACTTGCTTTTAAAATATAA

>PGSC0003DMG403026842

ATGGCAGATGCAGTGGTGAATTTTCTGGTAGAGAATTTGTTGCAGCTAATAAAAGAAAATGCGAAGCTAATTGGAAGTGCAAAAGGAGAATTGGACAATCTACATAAAGTTGCTCAACAACTCAAAGCATTTTTAGATGATGCTGCAAAATTCGGATACACGAATAGTGAGCAGTGGAAAGTTATGGTGATCGAAATACATAAAACTATGCACAGAGCTGAAGATGCAATTGATAAATTTCTGGTTCAAGCAAAGATGCATCAAGATAAAAATATGATCGAAAAGATGCTCGATTGGCCAAGTCATCTGATTAAAGTTATGAATCTTGCTGCTGAGATTAAAGGAATACATGATCAAGTCAAAGAGCTTCGTAAAAACAATCAAGCACTTCAGGCCACCCCTGTTCTTGAGCTCCCTAAAAAAGGGGAAGTAACTCAGGTAAAGGGTCCTTCTTTGGAGAATGATGCAGTGGTCGGGTTTGATGAAGAAGCTAACACAGTGATCAAGCGACTTGTTGAAGGATCGATGGAAAGTGTCGATATTATCCCAGTGGTGGGGATGCCCGGACTTGGAAAAACTACACTGGCAAGAAAAATCTGTAATGATTCTAAGCTTACATATGAATTTTACAGCATTGTTTGGGTTTATGTCGGTCAGGAATGCAAAGCAAAGGATATTTATCTTAGGATTCTTAAATTCTTCAAAAAGAACATAGAAGATCATCTCAATGATGATGTGGACACATTGGCCAAGGCAATAGGTGGTTATATCAAGAAAGGAGGTAGATGTCTCATTGTTTTAGATGATGTGTGGGAAGACGAAGTCATTGATCACGTAATGAAAGTTTTCGCAGAAAACAAAAGGGTCATCGGATTAATGATGACCACACGTGACTCACGTGTTGCTAAATTTGCCAATCCAGAACCTCATGAGCTGAAATTCTTGAAGAAAGAAGAAACTTTTGAATTGTTGGTAATGAGAGTTTTTGGCAAGGGAAGTTGTCCAAATGATTTAGTGAGAACTGGGAAAAAAATTGCAGAAAAATGTGGTGGAGTACCACTTGTAGTAGTGGTAATTGCAGGAGCATTAAGAGGTCGTTCGGACAAAAAGGAGTGGGAAAGAGTTGAGAAAAATGTGGTGCAATTTATGGGTGAGCATACCGAAGATAGCTGCCTGAAATATGTGAAGACGAGTTATGATCATTTGCCCCAAGAAATGCAGATGTGCTTCTTGTATTGTGGTGTCTTTCCTCGAGGCTTTGATATCCCTTGTTGGAAATTGATTCGTTTGTGGATAGCAGAGGGGTTGATAAAGCCGCAGCCTAACTTCACTCTAGAGGAGATAGCAGAGTTTTGTTTGACAGATCTTCTCAACAGAAATTTAGTAATAATAATGCAAAAGAGGTCTGATGGTCAAATAAAAACATGTCGTCTTCACGACATGTTGTATCAGTTCTGCAAAAAGGAGGCTAGTAACAGATGGCTTTTTCAAGAACCTGATCAATCTACCCTGGTTGCAGAACATGTCAGATCTTTCTATTGTTTTTCCTCAAAACAAAAACCAATTGACTTGTCTGCTAATGAAATCAAACTCATTCACAAAGCCTTTCCACTTATGAGAGTCTTGGATGTTGAATCTCTCAAGTTTATTTTCTCCAAAGACTTCAACCAATTATTTCATTTGAGGTATATTGCTATCTCAGGTGACTTTAAGGCCCTCCCTCCTACCTTTGGTAAATTCTGGAATTTACAAACTCTAATACTTAATACAAGTACCTTAGAGCCCACTCTTGAAGTAAAAGCAGAAATATGGAACTTGTTACAGTTGAGGCATCTCCACACCAACATACCTGCAAAATTGCCATCCCCGACTACCACCACAGGTAAACCTTCTTGTCTACAGACTCTTTCTATGGTTGCGCCAGAAAGTTGTGAGAAAGATGTGCTAGCAAAGGCGTGTAATGTTAGAAAATTGAGCATTCGAGGGCAAATGGTGGCTTTTCTTGGTGCTTACAAGGGTGGTATCAACAATCTTGAAGAGCTACAGTGTCTGGAACATTTAAAATTGTTGAATGATGTTCTTTTCATAAATAAAACACTTCACCTTCCACGAGCATTCTCCAAACTGGTACGTACGGTGAAGAAGTTAACTCTGACAAACACAAGGTTTGCTTGGAATGAGGCGGATAAATTGGGGATGTTGGAATCTCTTGAGGTCCTAAAGTTTAAAGAAAATGCATTCACGGGTCATATCTGGAAGCCAAAGAGTGGATTTAGCGCACTCCAAGTCTTGTGGATTAAAAGGTCAGAATTAGAATCTTGGGAGGCTTCGGTTATTAACTTCCCCGTGCTTAGGCAACTTGTTCTTATCTCTTGTGATAAGCTTGATGATGTGCCACTTGAGCTGGCTGATATACCTAGTCTTCAAGAGATGAGGCTGGATAACACAAGCAAAGCAGTAAAATCCGCAAAAAATGTTCGGGATAGCAAGACATCTAAAAGCATGAAACTCAAGCTAAGCATATTCCCTCCTGAAAATGAATCCAAGGTTCCACAGTGATATAAAAGGTCAGTTGAATTCATTTATACTGAAATCATTAAAATCCACATTATAGTCTTCACTAATAGTCCTGCTGGTGAATATCATAAATAA

>PGSC0003DMG400006003

ATGAATCAACAAAAAAGTAGTAGTAGTACTTCTTTGCTTCCAATAACTCCCGAAATTATTCGTTGGAGTTACGATGTTTTTCTAAGTTTTAGAGGTGAAGATGTACGTAAAACTTTTGTTGATCATCTCTATGTTGCTCTTCAACAAAAAGGGATCAACACATTCAAAGATTCTGAGAAATTAGAGAAAGGTAATTCTATTTCACCAGGACTTATGAGAGCAATTGAAGAATCGCGCATATCTTTGATTATTTTCTCAAAGAACTATGCTAATTCGAGGTGGTGTTTGGATGAAGTAGCGAAAATCATGGAATGCAAGAACGTGAAAGGACAAATTGTTATTCCGGTGTTTTATGATGTAGATCCATCAACAGTAAGGAAACAAAAATCGAGCTTTGAAGAAGCATTTAACAATTATGAAGATTGTTTCAAGGTGCAAAAATGGAGAGGAGCACTAGAGGAAGCAGCTAATTTATCTGGTTGGGATTTGCCAAATACTTCCAATGCGCATGAAGCTATAGTGATAAAGCAAATTGTGGAAGATATAATGGCTAGATTGGGTAGTCAGAGACACACGAAAAACGGTGAAAATCTTGTTGGAATCGAGTCACGTATGCAAAAAGTGTATAAAATGCTTGGCATGGGGTCTGGTGGAGTTCGTTTCGTTGGAATATTGGGAATGAGTGGAGTTGGGAAGACAACTCTAGCGAGAGTCATTTATGATAATATTCGGAGTCACTTTGAAGGTTCGTGTTTTCTTCATGAAGTTAGAGACCGTTCTGCAAAACAAGGACTAGAGCATTTGCAAGCTATACTTCTTTCTGAGATCCTTGTCATGAAAGATGTAAACATCAACAATTTATACGAAGGAGTTAATATGCAGATACAAAGATTACAGTACAAAAAGGTTCTTCTTGTTCTTGATGATGTTGATCATGTAGATCAGTTAGATGTTTTAGCGAGGAAACGTGAATGGTTTGGTCATGGAAGTAGAGTTATCATAACAACTAAAGATAAGCACTTGCTTGTTGAACATGAGGTAGAAAAGATATACAGAATGACAACATTAAATAAAGACGAAAGTTTGCAGCTCTTTAAGCTATATGCCTTCAAGAAAAACCGTCTTATGGATGAATTTAGGGATGTTTCAGCTCAAATTATAAGGCATTGTGATGGACTCCCGTTGGCTTTGAAAGTCCTCGGAAGTTTCTTGTATGGAAGAGATTTGGATGAATGGACAAGTGAAGTAGAACGATTGGAACAAATCCCAGAAGACAGAATTGTGAAGAAACTCGAATTATGTTTCAATCGACTAAACAGAATTGAACAAAAAATATTACTAGATATTGTGTGTTTCTTTATAGGAAAGAAGAAACAATCAGTTACAAGAATATTAGAGAGTTTTAATTTTAGTCCTGTTATTGGCATAAAAGTTCTCATGGAGAAATCTCTGATTACGGTTTCACAAGGTCGGATTCAGGTGCATCAATTGATACAAGAAATGTGTTGGTATATTATCCGTCAAGAAGCTTCTGATGATCCAAGAAGATATAGTAGGTTGTGGCTACCTCATCATATTTCTCATGTACTTGCTGGAGATTTGGGCACTGAAAAGATTGAAGGCATGTCATTGAACTGGGCTTTTGCACAAGAAGTGAATGTCAGCAGCGCGGCATTCACACAAATGAGCAGACTAAGGTTTCTCAGTATCCAGAACAAAAATGTTCATCAGGGCCCTAATTTCCTACCTGGTGAGTTGAGGTGGTTCAATTGGCACGCGTATCCATCAAGAAGTCTGCCTGTTAGTTTTCAGGGCGAAAAGTTAGTTGGTTTGAAGTTGAAAGATAGTGGAATCATACAACTTTGGCAAGGCTCCAAGGTTCTAGGAAAATTGAAATACATCAACCTTAGTGAGTCACGGAAGCTAGTAAGGACCCCGGATTTTTCAGGCATCCCTAATCTTGAAAGGCTGGTACTTGAAGGATGTGTAAATTTGGTAGAAATCAATTTTTCTGTTAGAGATCTCAGAAGGCTAGTCTTGTTGAATCTGAAGAACTGTAGAAATTTAAAGACCTTACCGAAGATCATTCAATTGGAAAGTCTTGAGGTTTTGATTCTATCAGGCTGCTTAAAGCTCAAAAAGTTGTCGATAATAAAAGAAGAAATGAATCGTTTGTCACAAGTGTACTTGGAAGGAACTGGTTTGAGAGAACTACCCGAATCAATTGAGAACTTTTCAGGAGTAACTTTGATAAATCTAAGTAACTGCAAGGATCTTGAGAATCTTCCAAGCAGTATTTTTAGATTGAAGCGTCTAAGAACACTTGATCTATCCGGATGTTCAAGACTAGAAGAATTATCAGATGATTTGGGACTTCTAGTCGGTTTAAAGGAGCTCCATTGTGATGACACAGCCATCCGAACACTGCCTTCCTCTATCTCTCAGCTAAAGAACCTTAAGCACTTATCTCTCCGGGGATGTAAAAATGCTTTGGGTTTACAAGTATGGACCTCACTCATTTTGTCGCGGCTATTTGGGAAAGGCCACAACTCTATGGGTCTGGTTTTTCCTAATTTATCAGGTCTTTGCTCATTGACAAAGCTGGACATAAGTGATTGCAATATTTCAGACGGACGAATCCTATCTAATCTTGGGTTCTTGCCATCTTTGGAGGAAGTGAATCTCGGGAAGAATAACTTTGTTGATATTCCATCTGCAAGCATCAATGGTCTATCTCGACTTAAAGTTGTTGAATTGGTTGGTTGTAAGAGGCTCGAGATTTTTCCAGAGCTTCCTTCAAGTATAGAAGAGGTGTATGCCGATGAATGTACATCATTGAGGAGTACTGGCATTGATCAATTAAGCAAATATCCAATGTTGTATCGAGTTTCACTTACTCAATGTCACCAACTTGTCAAAAATGAACCTGATGTTGCTATTATTGATTCATTGTGGAATCACATGCTCAAGGTAAGTAAATATAGCTCTTTATATATGTACACGTTCGTTAACTCAAGAATCTATAACATATATTGCATGATATAGGGACTATCGATGGTGGATGACGAATTCAGTATATGTATCCCTGGATCTGAAGTTCCTGACTGGTTTATGTACAAGAACTTGGGTCCTTCATTATCAGTGAAGCTACCTAAAAATTGGTACACAAACAAATTCATGGGCTTTGCTCTTTGTGTTGTTTTTGACAGTTTTAAGGAACCTTCCTGTATGAATCATGGTTACCTTAAAAAAATAACCCAATTTCATCTCATGTTTAAGCTCGTAAGGCACGATGGAAAGGCTGGTCTGCTTTTTAAAAGTATTGGCTCAGTAGGAAGTGAGGAATGTCCTGATTCAGGGCACACTCTCCTGGCCTATACATCATTTGATAATTTTTGGTCAACGTACGAGAAATGTGTTTGCAATCCTAACGACTGGATTCAAATCAAGGTTTGTGGGACTGATGCAAATGTTGCAATCAAAGGTTGGGGAATGCATCTTCTGTACGAGAACGATATTATAAATGAGGAGTTGATGACACAAAATGCCACCTCTCAATCTGGAAAGATGGGCTTGTTTCATGCAATCTTTGATGGATCAAAGTATACGAAAAGAAAACGACACGGACATAAATCGTTTTCTAGGCTGCCACCATATGATGAACCAGGATATGTCAATTCCTTTGGAGAAACAAGTAATGAAGATTTTGATGAAACATTATTTGGGACAGGAATTACTGGGATTATAGAAGGTCTACAAACAGTTTCTGTAGCTATTCTTGAATCAATTTTTTAA

>PGSC0003DMG400005970

ATGGATTTTCTTCTGCACGTACACATGCATTTAGGAGCGGATTCATGGACTACAGATCAAAAGTATACTTTTCCTGAACAGATCAACCGTTTGAGAATTTTGTTCAAAGAGTTACCATTGATTAACGACATTCAGGATGAGATGAGGATCCATTTCTTCAAGCGACTAGTGACGCTGGTCATTCATGCTGGACTTACTGTCTACTCGCAGGGTAATTGGGACCAAAAATTGCTTCTTCTCGATGGCATTATTCAGTCAGTCAAAACAGAGATTTGCCACAAGATCCGAGAGTGGGTAACATCTCATTTGCCTAAGAATGATAAATTGGGTTTTTCCAATTGCCTTCTTATTAGCTTGAAAGAGTTCCTGTCTGGTCATTCTGCTTCACTTGCTTCTGTGAAGGATCAAATCGAGGTAGTCCATGAGGAGCTTAACTTTTTCGAGCCTTTCATCATGCGTGTAGCAGAACAAGGCAATAATAAGCATCACGAACTCCAAAATCTTGTTGGAAGAGTCATTGATAAGGCTTATGAGGTCGAGTACATACTTGATTCTTTTGCAATTAGTGATGTACCTATTACTTTTCTTAGGACGTGGCTCTTGGAAATCATAAGGGAGATTGAGCTCATTAAGACTGAGTTGACCAAACCCAAGGGAAAAACTATGACAAGTGCCTCCCAAGCTACCGACGGAGAACTGGTTGGTTTTACAGATGTCTGTAAAACTATAAGAGATCAACTAGTTGGAGGATCACGAGAACTGGATGTTGTTTCCATTGTGGGTATGGCTGGATCAGGCAAAACAACCCTTGCTCGAAGTTTCATCAATGATGGCATAATTGGTACTCATTTTGATATCTGTGCAGAGTGTCGTTTTTCACAAGAATACACGCGTGAAGACTTGCTGTTTTCCATTTTGAGTTCTGCTAATTCTGGTCTCACTGATATTAGTAAAAGAAGTGCTGATATATTAGCTGATAGACTAAGAAAAACTCTATTGCCAAAGAGGTACCTCTTAATCATTGACGATGTGTGGGCAGTACAAGCATGGGACGATTTGAGATTATGCTTTCCTGAGGCCAAAAAAGGTAGCAGAATAATTCTGACGACCAGACTTGAAGAAGTTGCTACGTATGCTAAATGTGTCACTGAACCCATACACTTGCGTAGTATGAAAGATTCAGAGAGTTGGTTGTTATTACAAAAAAAGGTATTTGGGGAAGAAATGTGCCCCGAAGATCTAAAAGAAGTTGGGCAAAACATTGCATTCAAGTGTAATGGGCTGCCACTTTCCATTGTTTTAGTGGCTGGTCTTCTCACAAAGATTGACAAGACAGAAAGATGCTGGACGCGGATGGAATTAAGTTTTGGGGAAAGAGTGCAGGATGGCGCAAAGGATTTAGTAAAATTAAGTTATGAAGATTTACCTAACAAACTGAAATCATGCTTTTTATATTTCAGTGCATTTCTAGAGGACAGGGAAATTTCAGTGTCAAAATTAACAAGCTTATGGATTGCTGAGGCATTCTTAAAAAACAATGAGGACAAGTGTTTGGAGGATACGGCAGAAGATTACTTGAAGGATCTTATCGGAAGAAACCTCATTATGATAACTAAGAGGAACTCAACCGGGAAGATCAAAGCATGTCGTGTGCATGATCTAATGCTCGATTTCTGCAAGGAGAAAGCTAAGGAAGATAATTTCCTTTTGTGGCTGAAAAGGTATTACCTCTTTATATGTTTTGATTTAATTAGGATATTTTCTCTTATTCCTTATCCTACTTATATTCTTGATTCCAGGGATCGTCATTCCAATCCTCCGCGTTTTTATTCTAAAAGGCCTATGCACCGCCGTTTATCCTTTTGTTCTAATCGGGATGATCTTTCTGAGTGGAAGCCATCAAGCTCACATGCTCGTTCTATCTTATTCAGGGAGCTTAGTGATAGTGCATGTTCATCAATGGGACATGCCTCTTTCATCTTTGGCAACTTCAAATATCTCAGGGTGTTAGACTTAGAGGTGGTTGTTGTAGATTCTTTTCCCACTGAACTTAATCAGCTAAGGTACCTTGCTGTTCAAACTACTAAGAATTCTATTCCATCATCTATAGAAAATCTTTGGAATCTTCAAACGTTCATAGTCAAAAGAAATGGAGGACAAGTATGGTTGCCAGATACTTTTTGGAAGCTGAGTAAGTTGAGATATGTTAGCATTTGTGATGGCGCTTTGTTTGCTTCGCGTGATGCACCAGAATCCTGTGATGGAAAGTCCTTAAAGCTGGACAATTTGAAAACGTTTTCTTCAATATATATCTCCAGGATGAACAATATGGAAAGGATGGTAAGAAGAACACCCAATCTTCGGAAGTTAAGATGCGTATTTGCTGATTTAGGGCGTTGGGGAAAGAATGAAAATCGGTTTCCTGTATTAGACTCATTATCTCAACTTGAAACGCTAAAAGTAGTCTTCGTTGGTATCTCGGAGGTAGGTCCTTCAAGATTAAACTTTCCAGAGAATCTCAAGAAACTGACATTATGCAAATTTCCTTTGCCACCTGAGGAAATTGCAACTATTGCCAAACTTGTCAACCTTGAGGTACTTAAACTGCGACAAGTTGCCTTTGAAATGGGTGAATGGGAAGTGAGAGATCAGGAGTTTTCTCAGCTCAAACTCTTGGAACTAGAAAATCTCCAACTCTCAAAATGGGAAGTGTCTGAGGAAGCCTTCGATCGCCTTGAGAAATTGGTTTTGCGTGGATGTTTACATCTCGAAGCCATCCCTGATAGTTTTCAGGAACTAAGTTGTTTGCGATACATTGAGGTAAAATCATGCAGTGAAGATGTTGCCGACTCAGCTAGGATTATCAAAGAAACAAGAGAGGGTTATGGCCACAAATGTGACGTTAAAATCTTTTCTTAG

>PGSC0003DMG400029462

CAGCTTCCATTTGAGGAAGAAAGAAGAAGAATCAGCAGTCATATTGATGATTTCTTAAATGGCTTGAAGAAAATAAAAAATGAAAAAATAATTGCTTCATCAAAATTGGATGACATTGAAAAGCTAAGAATGGAGTTGAGATTCCTAAGAACATTTGTTCTGTTTGGGAATTCCAGTTTGGATTACTTTTATTACAGAATGTCCGAGAAAATAGATGAGTTCGATGAACTTAGTTGCTCACTTTTCTATCAAGATGAAGATGATAAATTAATCCTAGCGAAATGCGACATGGAGTGTCTTGCCCCTCTGCTGCTGGAAGAGATGAAAAGTTATTTTTTGAGTTTGGAGAATGATTATTATGTAGCCGTGACGACTGAAGAGAAAATGTTTCAATATCTCTTCAGACACCTTCATGATCTACCAAACTATAGTGCTAATTTGCTTCTCCCCTTGATGAGTGACTTCAAGATTCTTCAGCAAGTATTCCGACATCTGAGAGATTTCTATCCTATCCTCAAGGCCAACAAAGCACACACTGAATATCTCTACCCTTGGCTCCAATTGACTGCTGATAGAGTAACCCAATTCTGTTTTGATCTTTGGACTGGAGAGTATAAACGATATTATGATGGTTACCAGGTCTCTCAATGCTCTTCCAAGATTGCTTCTCTACTCATCGACATAATCCCTCTTGAGCTGGAGGTTTTATACATTTCTACTTCTAAGCTCATGAAAGAATCAAGGTCAACTGAACTAGAACGGTTTGTTAAGCAAATCCGAAAAGCATCACCAAGGATTCTTCAAAATTATCTCATTCTTATCCAAGGACACATGGCAGGTGCAGTAGCTGTCAATTACGCTCCAACTCAAAGCATTAATGTCATGATAGAGTTCCTATTGATCTTTCTCACTGATATACCAAAGCGATATATCCATCTTGACAAATTGAATGATATGTTGGCACATATTGGACTACTTACAAGGAAGATATCTATTCTGTTGGAGGAGAGCTCTGAGTATAATATCAATGAAGCGGACTTTTCAGCTTCAGACTTGTTGCAAGAAATTGAACAAATGAAGCGAGATATTAGACAGATTTTTTTGAAAGCTCCAGAGTCATCTCAACTTCGCTTTCCTATGGATGATGGTTTCCTCTTCATGAATCTTCTACTCAGACATTTAAATGATTTGCTCATTTCCAATGCTTATTCAGTTTCTCTCATAAAGAAAGAAATTGGGATGGTGAAACAAAGCCTTGAATTCATAAGATCATCTTTCGAGAAAGTCAGGCAAACATTGAATGACACTAGCCAAGTAGTTAAAGATTGTTGGGTGCGTGCTTTAGATGTGGCATATGAGGCAGAACATGTCATTAATTCCATTCTTGTCAGAGATAATGCTCTCTCGCATCTCATCTTCTCACTTCCGAGTGTCACTGATAAGATCAAGCTTATCGTGGCAGAAGTCACCAGTTTACAGCTGGAGGATAACAATGGGGATCACCCCCTTGATGCAAAGTCTTTCGTCGAGCCAATTGAGTCAACCTCATCACCTTTTGTTGAGGTAACAGTAGGTCATGAGAAAGAAGAATCCCAGATCATTGGCCAGCTCCTTAATCAACATGAATCTGAGCTTGATGTCATTTCAATTGTCGGAATGCCAGGACTCGGTAAAACTACTCTGGCCAACAAAGTGTATAACAATACATTAGTTGCTAGTCATTTCAAGATTCGTGCTTGGTGCACTGTTTCCCAAAAGTATAACAAGTCAAAGGTGTTGCGGGAGATTCTTCAGCAAGTTGCTAGCTCTGAAGAAAAAGAAAGTGAGGATCACGACCTTGCTGAAAAGCTAAGAAGAGCATTGTACGATAAAAGGTACCTCATCGTCTTGGATGATGTGTGGGATATTGCAACAGGGGAGATGTTAATAGCATGTTTTCCAAAGCTTAAGAGAGGAGATAGAGTTATTTTAACTAGCCGAATTAGTAAGGTAGGTTCGCAAGTTAAATGTCGTACTGATCCTATTGACCTCCAAGTTTTAACACTTGAAAAAAGTTGGGAACTATTCGAAAAAAGGGTATTTGGAGAAGGAAGCTGCCCTGCTGAACTGTCAGATGTTGGACACCAAATAGTTAAGAAATGTAAAGGACTTCCCTTGGCTATTGTTTTGATTGCTGGAGTAATTGTTAGAGGAAAGAAAAAGGAAAAGAATTTGTGGCTTAAGATTCAACATAATCTGGATTCCTTTATTTCTGCCAACATCAATTTGCAGATGATGAAGGTTATGCAATTAAGTTATGACCATTTACCATGCCACCTGAAGCCGTTGTTGCTTTACTTCGCAAGATCTCAAAAGAACAAACGAACTCCGGTCTCTGAGTTAAAGCAGTTGTGGATGGCCGAAGGGTTTGTGGATTATGATATCCCATCCAAGGGTAGTTTAGAGGAAGCAACTCAAAGATACTTGGATGCTTTAGTTTCCAGTAGCCTGATAATGGTGGATCAAACCCACTCCAAGAAGAGTATGCCTTTTTCTGTTAGGATCAAGGTTTGCTATGTGCATGATGTTGTGCACGATTTTTGTTCAGAAAAAGCTAAAAAGGAAAAGTTTTTCAAGTTAATGAATCCAGGTGCACGATTTCATGCTTCGGATTTCCTACATCATCGTCTAACCATTCATATTGACGACAGTCAACTCCATAAAAAATGTGTTTTGTTCAATTTTAATAAGTGTTCAGCTGGTAGTAAGCATCTCATATCTTTGGAAGTTAGCGGTTCACTTCTTAACTCCAGCTATATCTGCCATACAAGATCCTTTGGACTTGCTAGAGTGTTGCAACTGGATAGCATTATTCTGGAAGATTCTTTAATGGAAGAAATAGGCTCACTATTTCATTTGAGGTTCTTAAGGATTCGGACTCGAGATGTAAAAGCTATCCCAGTGTCGTGGTTGAACCTCCAGAATCTGGAAACTTTGTTGATCAATACTAGATATTCCACCATGGTATTACTGCCCGGAATATTCAAACTGTCAAAGCTGAAACATGTGAAAATTGACATGAGTTCTTTCTTTGAAGAGGATGTAATGGATGTGGACAATATTATGCATCAACCAAGTAGAATATTGGAAGCTGAGAATTCAAAGTTAGAAGACTTGACAACTTTATCTAAAGTTGATATCTCATATACTGAAGCCACGAGTGATGTTCTGGAGAAGTTCCCAAATCTTCACCACCTTCATTGCCACATCAAGGAACCGATAGATCCTCCTACACATTGCGATTGGTTTCCCAAGTTTGATGTCCTTAATAAACTTAAATCACTCATTGCAATATATGAGAATTCCGGCTATCCTGACAAAATCCGACAACCCAATGAATATTACTTCCCTAACAGCTTGAAAGAGTTACGGTTGTCTGGTTTTTCCCTGAGACCTGATTTGTTGTCAGCAATCGCGGCATTGCCTCAGCTTGAAATTCTGGAGTTTGTCAGCTGTAATTTCGTGGAGGACAAGTGGGATGCAAGTGAGCACATCTATCAACGTCTTAAGACTTTGTCTATGCGAGTGGTCAACCATTCGGAATGGGAAGTTGATACGGAAACCTTTCCCAAGCTTGAGGAATTAATACTAGAAGATTGTTACAAGCTTAGGGAGATCCCTTGTGCATTTATGGATATACACACTTTAAAGTCCATTCATTTAAATAATAATAAGCGTGAGCTTGGAGATTCAGCCATTGAGATTAAGAAACAAGTAGAAGCTTTCACAGGAGAGGACAGACCTCAAGTCCATATAGATATGAAGAAGAAGCTGAAGAAGAGGCTGAAGAAGAAGCTGAAAGAAGAGGTAATTAATTCTTATATCGTTACCCCTACATACATATATACATTTAATTTAAATCATACTATATATGATTTGCAGAATGCCCAAAGGACTCAAGGATAG

>PGSC0003DMG400029460

ATGGATAGTCTTGTCCCTCTGCTGCTGGAGGAGATGGAAAGTTATTTAAGTTTGAAGAATGATAATTATGTAGCAATGACCACAGAGGAGAAAATGTTTGAATACCTCTTCACAAACCTCCATGATCTACCAAACTATTCTTCTGATTTGATTCTCTCGCAGATGACTGACTACAACACTCTTCGCCAAGTATTTCGTCGTCTGACAGATTTCTATCCTATCCTCCTGGCCAACAAAACAACTACCACTCAATATCTCTTTCCTCGCTTCCAATTCATCGCCCATACATTTCTACAATTCCATTTTGCTATTTGGACTGGAAACTATAATCCCTCATCCAAAGTCTCTGAATACTCTTCCAACGTCACTTCTCTACTGATCGACATAATCCCTCTTGAGCTGGAGGTTCTATACATTTCTACTTCTAAGCTCATCAAAGAATCAAAGTCAACTCATCAACTAAAACGGTTAGTGAAGCAAATCCTAAAAGCATCTCCAAAGATTCTTCAAAATTATCTCATTCATCTCCAAGGACGCATGGTAGCTGTCAATTACTCTCAAACTCAAAGTATTAATGCCATGATCGAGTTCCTATTGATCTTTCTCACCGATATACCAAAGCGCTTTATCCATCCTGACAAATTGAACGATATGTTGGCACATGTCGGACTACTTACAAGGAATCTATCTATTCTCATGGAGGAGAGCTCTAATATGAATGAAGCAGACTTTTCAGCTCCAGACTTGTTGCAAGAAATTGAACGAATGAAGGGAGATATCAAACAGAATTTTTTGAAAGCCCCGGATCAGTCATCTCAACTCTGCTTTCCTATGGATGATGGTTTCCTCTTCATGAATCTTCTACTCAGACATTTAAATGATTTGCTCATTTCCAATGCTTATTCAGTTTCTCTCATAAAGAAAGAAATTGGAATAGTGAAACAAAGCCTTGAATACCTAACATCATCTTTCAGGCAAACATTGGATGACACCAGTGGAGTAGTTAAAGATTGTTGGGTGTGTGCTTTAGATGTGGCATATGAGGCAGAACATGTCATTAATTCCATTCTTGTCAGAGATAATGCTCTCTCGCATCTCCTTTTCTCACTTCCGAGTGTCACTGATAAGATCAAGCTTATCGTGGCAGAAGTCACCAGTTTACAGCTGGAGGATAAGAATGGGGATGACCCTATTGATGCAAAGTCTTCCGTCGAGCCAATTGAGTCAACCTCATCATCTTTTGTTGAGGTAACAGTAGGTCATGAAGAAGATGAAGCCAAGATGATTGACCAGCTCCTTGATAAACATGAATCCGAGCTTGATGTCATTTCGATTGTCAGAATGCCAGGACTCGGTAAAACTATTCTGGCCAACAAAGTGTATAACAATACATTAGTTGCTACTCATTTCAATGTTCGTGCTTGGTGCACTGTTTCCCAAAAGTATAACAAGTCAAAGGTGTTGCGGGAGATTCTTCTACAAGTTACTGGCTCGGGAGGAAAAAAAAGGGTATTTGGAGAAGGAAGCTGCCCTGCTGAACTGTCAGAAGTTGGACACCAAATAGTTGACAAATGTCAGGGGCTTCCTTTGGCTGTTGTTTTGATTGCTGGAGTAATTGTTAGAGGAAAGAAAATGGAAAATGATTTGTGGCTTAATATTCAAGATAATTTGGATTCCTTTATTTCTGCCAACATCAATTTGCAGATGATGAAGGTTATGCAATTAAGTTATGACCACTTACCATACCACCTGAAGCCGTTGTTGCTTTACTTTGCAAGATCTCGAAAGAGCAAACGAACTCCAGTCTCTAAGTTGATGCAGTTGTGGATGGCTGAAGGGTTGGTGAACCATGATATCCCATCCAAGTGTAGTTTAGAGGAAGCAACTCAAAGATACTTGGATGCTTTAGTTTCCAGTAGCCTGATAATGGTGGATCATATGCTCTACAAGAAGAATATGCCTTTTTCTGTTAGGATCAAGGTTTGCTATGTGCATGATGTTGTGCACGATTTTTGTTCTGTAAAAGCGAAAAAGGAAAAGTTTCTCAAGTTAATCAATCCAGGTGCTCGATTTCACGCTTCTTATTTCCTACACCATCGTCTAACCATTCGTACTGACAACAACCAACTCCACAAAAAATGTGTTTTGTTCAAGTCTAAAAAGTGTTCAGTTGGGAGTAAGCATCTCGTATCTTTGAAAGTTAGCGGTTCACTTCTCCACTCCAGCTATATCTGTCACACAAGACCCTTTGGACTTGTTAGAGTATTGCAACTGGATAGCATTGAGTTGAATGATTCTTTAATGGAAGAAATTGGCTCCCTATTTCATTTGAGGTTCTTAAGGATTCGGACTCGAGGTGTAAAAGCTATCCCAGTGTCGTGGTTGAACCTCCAGAATCTGGAAACTCTGTTGATCAGTACTGGATATTCCACCATGGTATTACTGCCCAGAATATTAAAACTGTCAAGCAGAGGAAGAGGATGCGGACAATATTATGAATCAACCAAAGTTAGAAGACTTGACAACTTTATCCAAAGTTTTTAATCTTCATACACTGAAGCCACGAGTGATGCTCTGGAGAAGTTCCCGAATCTTCAGCACCTTCATCGCATCATTGTGGAACCGCGGTACCCTCTTACTCCTACACACGGCGATTGGTTTCCCAAGCTTGATGTTCTTAATAAACTTGAATCACTCTTTGCAAAATACAGGATGCCCAAAGAGTACTTATTTCACACCGAGTCAATCTACCAAATACCACATCCCAATGAATATCACTTCCCTAGTAGCTTGAAAGAGTTACGGTTGAATTTATTTCACGTGAGACCTGCTTTGTTGTCAGCAATCGCGGCATTGCCTCACCTTGAAATTCTGGTGATTATCGACTCTGATTTCGTGGAGGGTAAGTGGGATGCAAGTGAGGACATGTATCAAAGTCTTAAGACTTTGACATTGCGAATGGTCAACCTTTCAGAATGGCAAGTTGATAGGGAAACTTTTCCCAAGCTTGAGGAATTAATAGTAGAATACTGTGAGGAGTTTACGGAGATCCCTTGTGCATTCGCGGATATAGATACTTTAAAGTTCGTTCATTTAACAAATATTAACAGTCAGGTTGTAGATTCAGCCATGGAGATTAAGAAAGATGTAATTGATTTCGCAGGAGAGGACAGACTTCATGTCCATATATTCCATTGCTAA

>PGSC0003DMG400029457

GAGGTGCCACTTGAGGAAAAAAGAAGAAGAATCAGGAATCTTATTGATGATTTATTGAATGGGTTGAAGAAAATTCAGAATGAAGAAGAAGAAGAATTCCTTGATTCAAAATGGTATGACATTGAAAAGCTAAGAATGGAAGTGAGATTCTTAAGAACATTTGTCCTGTTTGGGAATTCAAGTTTGGGCAAGGACTTTTATGAGAGGATGTCCAACAAAATAAACAAGTTCAGTGTTATGACTCAGTCACTACTTAATGATGAAGTAATTCTAGAGAAATACAACATGGATGGTGTTGTCCCTCTGCTGCTGGAAGAGATGCAAAGTTATTTGAGTTTAAAGAACGATAATTATGTAGCAATGACCACAGAGGAGAAAATGTTTGAATACCTCTGGATAGACCTTCATGATCTACCAAACAGTACTTCTTATTCGGATGATGACTTTTATGAGAGGATGTCCAAGCAAATAAACATTCTTCGCCAAGTATTCCGTCGTCTGACAGATTTCTATCCTATCCTCCTGGCCAACAAAACAACTACTACTCAATATCTCTTTCCTCGCTTCCAATTCATCGCCCGTAAATTTCTACAATTCTATTTTGATATTTGGACTGGAAAGTATAAACCAAGTTATGAAGCTGATGAGTACTCTTCCAAAGTCTCTTCTCTACTCATCAAGATAATCCCTCTTGAGCTGGAGGTTCTATACATTTCTACTTCTAAGCTCATAAAAGAATCAACGTCAACTCAATTAAAACGGTTTGTGAAGCAAATCCTGAAAGCATCTCCAAAGATTCTTCAAAATTATCTCATTCATCTCCAAGGACGCATGGTAGCTGTCAATTACTCTCAAACTCAAAGTATTAATGTCATGATCGAGTTCCTATTGATCTTTCTCACCGATATACCAAAGCGCTTTATCCATCCTGAAAAATTGAACGATATGTTGGCACATGTCGCACTACTTACAAGGAAGATATCTACTCTCATGGAGGAGAGCTCTAATATGGATGAAGCAAATTTTTCAGCTCCAGACTTGTTGCAAGAAATTGAACGAATGAAGGGAGATATCAAAAAGATTTTTTTGAAAGCCCCGGATCAGTTTCATGTGGATGATGGTTTCCTCTTCATGAATCTTCTACTCAGACATTTAAATGATTTGCTCATTTCCAATGTCTATTCAGTTATGCTGATAAAGAAAGAAATTGGGATGGTGAAAGAAAGCCTCGAATTCCTAAGATCATCTTTCATGAAAGTCAGGCAAACATTGGATGACAGTAGTGGAGTAGTTAAAGATTGTTGGGTGCGTGCTTTAGATGTGGCATATGAGGCAGAACATGTCATTAATTCCATTCTTGTCAGAGATAAAGCTCTCTCGCATCTCATCTTCTCACTTCCGAGTGTCACTGATAAGATCAAGCTTATCGTGGCAGAAGTCACATGTTTACAGCTGGAAGATAAGAATGGGGATGGCCCCCTTGATGCAAAGTCTTCCAACGAGCCAATTGAGTCAACCTCGTCATCTTTTGTTGAGGTAACAGTTGGTCATGAAGAAGATGAAGCCAAGATGATTGACCAGCTCCTTGATAAACATGAATCCGAGCTTGATGTCATTTCCATTGTCGGAATGCCAGGACTCGGTAAAACTACTCTGGCCAACAAAGTGTATAACAATACATTAGTTGCTAGTCATTTCAAGATTCGTGCTTGGTGCACTGTTTCCCAAAAGTATAACAAGTCAAAGGTGTTGCGGGAGATTCTTCAGCAAGTTGATAACTCTGAAGAAAACGAAAGTGAGGATCACGACCTTGCTGAAAAGCTACGAAGAGCATTGTACGATAAAAGGTACCTCATCGTCTTGGATGATGTGTGGGATATTGCAACAGGGGAGATGTTAATAGCATGTTTTCCAAAGTTTAAGAGAGGAGATAGAGTTATCTTAACTAGCCGAAATAGTGAGGTAGGTTTGAAACTGAAATGCCATAGTGATCTTCTCTGCCTTCAACTTTTAACATTTGGAAAAAGTTGGGAGTTATTCGAAAAAATGGTATTTGGAAAAGAAAGCTGCCCTGCTGAACTGTTGGATGTTGGACACCAAATAGTTAAGAAATGTCAAGGGCTTCCTTTGGCTGTTGTTTTGATTGCTGGAGTAATTGTTAGAGGAAAGAAAGAGGAAAAGGATTTGTGGCTTAAGATTCAACATAATTTGGATATTTCTGCCAACAACAATTTAGAGATTATGAAGGTTATGCAATTAAGTTATGACCATTTACCATACCACCTGAAGCCGTTGTTGCTTTACTTTGCAAGATCTCGAAAGGGCAAACGAACTCCAGTCTCTAAGTTGATGCAGTTGTGGATGGCTGAAGGTTTTCTGGACCATGATATCCGATCCAAGAATAGTTTAGAGGAAGTAACTCAAAGTTACTTGGATGCTTTAATTTCCAGTAGCCTGATAATGGTGGATCATATCAAGGGGAGTCACCCATTTTTTGTAAAGATCAAGGTTTGCTATGTGCATGATGTTGTGCATGATTTTTGTTCAGTAAAAGCGAAAAAGGAAAAGTTTTTCACGATAATCAATCCGGGTAATCGATTTCATGTTTCTGATTTCCTACACCATCGTCTAACCATTCATATTGAGGAGGGCCAACTCCACAAAAAATGTGTTTTGTTCAAGTCTAAGAAGTGTTCATTTGGTAGTAAGCATCTCATATCTTTGAAAGTTAGCGGTTCGCTTCTCCACTTCAGGTATATCTGTCATACAAGACACTTTGGACTTGTTAGAGTGTTGCAACTGGGTAGCATCCTTTTGAAAGATTTTTTAATGGAAGAAATAGGGTCCCTATTTCATTTGAGGTTCTTAAGGATTCATACTCTTAATGGAAAAGCTATCCCAGAATCGTGGTTGAACCTCCAGAATCTGGAAACTCTGTTGATCAATACTGGAGATTCCACCATGGTATTACTGCCCAGAATGTTACAACTGTCAAAGCTGAAACATGTGAAAATTGACATGAGTTGTTTCTTTGAAGAGGAAGAGGACGCGAAAAAAATCCAAAGTAGAATATTGAAAGGTGAGAATTCAAAGCTGACAACTTTATCCCAAGTCAATATCTCATATTCTGTAGGCACGAATGATGCTCTGGAGAAGTTCCCAAATCTTCAGCACCTTGATTGCACCATAGTGGAAGCGCAGCACCCTCCTACCCAGGACGATTGGTTTCCCAAGCTTGATGTGCTTAATAAACTTGAATCATTCATAGCAATATACTGTTGCCCCGAGTTAATCCCTCAACCCAAAAAATATCACTTCCCTAGCAGCTTGAAAGAGTTACGGTTGTATCGGTTTACCATGACACCTGCTTTGTTGTCTGCAATCGTGGCATTACCTCAGCTTGAAATTCTGGCGATTATCGACTCTGATTTAGTGAAGGATAAGTGGTATGCAAGTGAGGGCATGTATCAAAGTCTTAAGACTTTGAGTTTGCGACTGGTCAACCTTTCAGAATGGGAAGTTAATAGGGGAGCTTTTCCCAAGCTTGAGGAATTAATACTAGATAATTGTTACAAGCTTACGGAGATCCCTTGTGCATTTGCGGATAGAGAAACTTTAAAGTCCATTCATTTAATGAATATGAACCGTGAGGTTGAAGATTCAGCCATTGAGATTAAGAAACAGATAATAGAATTCGCAGGAGAGGACAGACTTCAAGTCCATTTATCAGATGTGTTGTATGAATTATAG

>PGSC0003DMG400029506

ATGGATCTGAGATTCCTAAAAACATTTGTCCTGTTTGGGGATTCAAGTTTGGATGACTTTTATGAGAGGATGTCCAACAAAATAAACAAGTTCAGTGTTATGACTCGGGGACTACTTAATGATGAAGTAATTCTAGAGAAATACAACATGGATGGTGTTGTCCCTCTGCTGCTGGAAGAGATGCAAAGTTATTTGAGTTTGAAGAATGATAATTATGTAGCAATGATAACAGAGGAGAAAATGTTTGAATACCTCTTCACAAACCTTCATGATCTAACAAACATTACTTCTTACAGGCCGATGTCCACGAAAATAAACATTCTTCGCCAAGTATTCCGTCGTCTGACAGATTTCTATCCTATCCTCCTGGCCAACAAAACAACTACTACTCAATATCTCTTTCCTCGCTTCCAATTCATCGCCCATACATTTCTACAATTCTATTTTTATATTTGGACTGGAAAGTATAAATCAAGTTATGATGCTGATGAGTACTCTTCCAAGATTGCTTCTCTACTCATCGACATAATCCCTCTTGAGCTGGAGGTTCTATACATTTCTACTTTCAAGCTCATGAAAGAATCAAGGTCAACTCAATTAAAACGGTTTGTGAAGCAAATCCTAAAAGCATCTCCAAAGATTCTTCAAAATTATCTCATTCATCTCCAAGGACGCATGGTAGCTGTCAATTACTCTCAAACTCAAAGTATTAATGTCATGATCGAGTTCCTATTGATCTTTCTCACCGATATACCAAAGCGCTTTATCCATCCTGACAAATTGAACGATATGTTGGCACATGTCGCACTACTTACAAGGAATATATCTATTCTCATGGAGGAGAGCTCTAATATCAATGAAGCGGACTTTTCGGCTTCAGACTTGTTGCAAGAAATTGAACAAATGAAGCGAGATAGTAGACAGATTTTTTTGAAAGCTCCAGAGTCATCTCAACTTTGCTTTCCTATGGATGATGGTTTCCTCTTCATGAATCTTCTACTCAGACATTTAAATGATTTGCTCATTTCCAATGCTTCTTCAGTTTTTCTGATAAAAAAAGAAATTGGGATGGTGAAAGAAAGCCTTGAATTCCTAACAACATCTTTCGGGAAAGTCAGGCAAACATTGAATGACACTAGTGGAGTAGTTAAAGATTGTTGGGTGCGTGCTTTAGATGTGGCATACGAGGCAGAACATGTCATTAATTCCATTCTTGTCAGAGATAAAGCTCTCTCGCATCTCATCTTCTCACTTCCAGATGTCATTGATAAGATCAAGTTTATCGTGGCAGAAGTCACCAGTTTACAGCTGGAGGCTAAGAATGGGGATGACCCCCTTGATGCAAAGTCTTCTGACGACCCAATTGAGTTAACCTCATCATCTTTTGTTGAGGTAACAGTAGGTCATGAGGAAGTAGAAGCCCAGATCATTGACCAGCTCCTTGATGAACATGAATCCGAGCTTGACGTCATTTCGATTGTCGGAATGCCAGGACTCGGTAAAACTACTCTGGCCAACAAAGTGTATAACAATACATTAGTTGCTAGTCATTTCAAGATTCGTGCTTGGTGCACCGTTTCCCAAAAGTATAACAAGTCAAAGGTGTTGCAGGAGATTCTTCAGCAAGTTACTGGCTCGGAAGAAAAAGGAAGTGAAGATGACCTTGCTGAAGAGCTACGAAGAGCACTGTATGATAAAAGGTACCTCATCGTCTTGGATGATGTGTGGGATATTGCAACAAGGGAGATGTTAATAGCATGTTTTCCAAAGCTTAAGAGAGGAGATAGAGTTATTTTAACTAGCCGAATTAGTAAGGTAGGTTCGCAAGTTAAATGTCGTACTGATCCTATCGACCTCCAAGTTTTACTACCTGAAAAAAGTTGGGAACTATTCGAAAAAAGGGTATTTGGAGAAGGAAGCTGCCCTGCTGAACTGTCAGAAGTTGGATACCAAATAGTTGAGAAATGTAAAGGGCTTCCTTTGGCTGTTGTTTTGATTGCTGGAGTAATTGTTAGAGGAAAGAAAAAGGAAAAGAATTTGTGGCTTAAGATTCAACATAATTTGGATTCCTTTATTTCTGACAACATCAATTTGCAGATGATGAAGGTTATGCAATTAAGTTATGACCATTTACCATGCCACCTGAAGCCGTTGTTGCTTTACTTCGCAAGATCTCAAAAGACCAAAAGAACTCCGGTCTCTAAGTTAAAGAAGTTGTGGATGGCCGAAGGGTTTGTGGATTATGATATCCCATCCAAGGGTAGTTTAGAGGAAGCAACTCAAAGTTACTTGGATGCTTTAATTTCCAGTAGCCTGATAATGGTGGATCAAACCCACTCCAAGAAGAGTATGCCTTTTTCTGTTAGGATCAAGGTTTGCTATGTGCATGATGTTGTGCACGATTTTTGTTCAGAAAAAGCTAAAAAGGAAAAGTTTCTCAAGTTAATGAATCCAGGTGCTCGATTTCATGCTACGGATGTCCTACATCATCGTCTAACCATTCATACTGACGACAGTAAAAAATGTGTTTTGTTCAATTTTAATAAGTGTTTAGCTGATAGTAAGCATCTCATATCTTTGAAAGTTAGCGGTTCACTTCGTCACCCCAGCTATATCTGCGATACAAGATCCTTTGGACTTGCTAGAGTATTGCAACTGGATAACATTATTCTGGACGATTCTTTAGTGGAAGAAATAGGCTCCCTATTTCATTTGAGGTTCTTAAGGATTCGAACTCCAGTAAAAGTTATCCCAGTGTCGTGGTTGAACCTCCAGAATCTGGAAACTCTGTTCATCAATACTGGAATGTCCACCATGGTATTACTTCCCATAATATTCAAACTGTCAAAGCTGAAACATGTGAAAATTGCCAGGAGTTCTTTCTTTGAAGAGGAAGAGGAAATGGATGCGGACGATATTATGCATGAACCAAGTAGAATATTGGAAGCTGAGAATTCAAAGTTAGAAGACTTGACAACCTTATCTGAAGTTGATATCTCATATACTGAAGCCATGAGTGGTGTTCTGGAGAAGTTCCCGAATCTTCAGCACCTTCATTGCCACATCAAGGAACCGATAGATCCTCTTACACAGGGTGATTGGTTTCCCAAGTTTGATGTCCTTCATAAACTTGAATCACTCTTTGCAATGTACGAGAATGTTGGCTATCCCAAGTTAACAGAAAAAATCGGACAACCCAATGAATATTACTTCCCTAACAGCTTGAAAGAGTTACGGTTGAATGGTTTTCCCCTGAGACCTGATTTGTTGTCAGTAATCGCGGCATTGCCTGAGCTTGAAATTCTGGAGTTTATTTACTGTGAATTCGTAGAGGACAAGTGGGACGCAAGTGAGGACATCTATCAAAGTCTTAAGACTTTGTCTATGCGAATGGTCAATCATTCGGAATGGCAAGTTGATATGGAAACCTTTCCCAAGCTTGAGGAATTAAGACTAGCACATTGTTACAAGCTTACGGAGATCCCTTGTGCATTTATAGATATACACACTTTAAAGTCCATTCATTTAACTAATGTTAAGCGTGAGGTTGAAGATTCAGCCATTGAGATTAAGAAACAGATAGTAGATTTCGCAGGAGAGGACAGACTTCAACTCCATATATCAGATGTGTTGTATGAATTAGAAGCTGAGGAAGAGGATGAAACAGAG

>PGSC0003DMG400029505

ATGGATCCGTTTACGGCCGCTGCATTAACCACGGCGGTGACAGCCACCGTGAGCCTTCTGGTGGAGAACTTGTCGCATCTTATAAGTTATAATTGGAAGTTGTATACAGGATTGAAGAAATCGTGTGAAGATTTGTTTGATGAAGTGAAGCGATTAAATGCATTCTTAGTTGATAACGCGAATCAGAGAAGTAATAGTACGCAATGGGAGGTACTAGTCGATAAAATTCGACGTACAGTATATAAAGCAGAGGATGTTGTTGATAAATTATTGATTCAGGCCAAGATAGACCAAGAGAGTAGTATAGCTAAAAAATTGATTCACAAAACTTACAAAAACAGGAATTTTACAGAGGAAATCAATGAGATACTTGAAGAGGTGAGGAAAATTCTTGATGAAAATCAACATTTGTTTGAGGCCAACCCAATGATTGATCATCATGCTGAAAAAGTTGTCCAGGAGGAACAGGTTCGTTTATCAAGACAAGTACTGTTAATAACTTGTTTGTCCATCTTAAAGGGTTCGTCATTGGAAAATCACGAAGTGGTTGGATTTGATGAGGAAGCAGCAAAAGTGATCAATCGTCTGGTTGAAGGAGTAGAGTGTCTAGATGTTATCCCGGTTGTAGGAATGCCGGGACTTGGTAAAACCACACTGGCAAGAAAAATCTATAATGATCCTAAGATTTCGCGAGAATTTTTCAGTTACATTTGGGTTTTCATCGGACAATCAACGTGTGTAAAACGGGATATCCTTTTTAATATTCTGAAAAAGTTCACAAATTCCGTTGATGAATTCAAAAACAGAAATGAGGCAGACATAACTCAGGAAATACGTAAGCGCGTGGCTAATGGAGGTAAATGTCTCATTGTCTTGGATGATGTGTGGGATCCAAATGTTGTAGATTTTGTCAAGACAGTTTTCCCTGATAATAAAAAAGCCCACAGGATCATGATGACCACTCGACACGAAGACATTGCTAGATCTGTCAATAAATATCCCCACAATCTGAAATTTCTGGATGGAGATGAAAGTTTCCAGCTGCTAGAAAAGAGAGCTTTTGGCGTTAGCCGTTGTCCTGTTGAGTTAGTAGAACATGGAGAAGCCATTGTAGCAAAATGTAGTGGAGTACCACTTACAATTGTGGTAATTGCAGGAGCTTTAAGAGGTCGTACGAGCGAAATTGATTGGAAAGTAGTTAGAGAAAATGTGGGGAAGCACCTTTTAGAAGACAAACGTCAGAGATGTTTGAATGTTGTGAGACTGAGTTACAATCATTTGCCTCAAGAAAAAAAGGCTTGCTTCTTGTATTTTGGTGCCTTTCCTCAAGGATTCGATATCCCTGCTTGGAAATTGATTCGACTCTGGATTGCCGAGGGACTCATAATGTCCAAGTTATCAGGCATTGAAATTGAAGATATAGCAGAGTATTATCTGAATGACTTTGCCAACCGGAACTTAGTGATGGTAACAGGAAAGAGGTCTAATGGTCGAATAAAAACTTTTCGTGTTCACGACATGTTACATGAGTTCTGCGTTGAAGAGGGTACTAGATTGACTCTTTTCAAACAAGTATGTCTCACATCTGATCAAGACATACAAAACTCAATTACTTGTCGTCGAGTGTCTATTCAATCATCTGTTCCTCAAAATTTCATCTCAAAAAAGACAGTTGAAGAACATGTTAAGTCATTGTTATGTTTTTCCTCAAAACAAAAACAAGTTGACTTTTCTAATATCGATGTCAAACTCATCCCTAACGCATTTCCACTTATGAGAGTCTTAGACATTGAATCCCTCAAGTTTAGTATTCCCAGGGAATTTTACCAGCTATTGCACTTGAGGTATATTGCTATCTCAGGTGAGTTCAAGGAACTTCCTAAACTCTTCACTTCTTTCTGTAATGCACAAACTCTTATTCTGAATACTTCCAAGCCCACCCTTGATATAAAAGCTGACATATGGAACATGCCACGTTTACGCCATCTGCGCACCAACAAACCTGCAATCTTGCCACCTCCTACTGCCTCATCAACAAGTAGTAGTACAAATTCTTGTTTGTTGCAAACTCTATCTCTGGTTACACCAGAAAGCTGCAATGGAAATGTTCTTTCAAAGGCTGGTAATGTCAAAAAAATGAGTATTGAAGGTAATTTGACGCCTTTTCTTGAAACTAGCAAGGGTGAATTTTTCAGCAATTTTCAAGTGCTAAAGCTCCTGGAAAGTTTAACACTGCTAAATGATGATAAGAGTAATAAAGCTCTTCACCTTCCGTCAACATTCTCCGAATGTTTACCAAATTTGAAGAAGTTAACTCTGTCAAAAACAAGGTTTGACTGGAACCAGGCATATAGATTGGGGCAGGTGAAAAATCTCCAGGTCCTAAAATTGAAAGAAAATGCATTCACGGGGCCGTCCTGGAAGATGGAGCCAGGAGGTTTCAAGAAACTTCAGGTCTTGTGGATTGAAATGGCAGATTTCGTGTCGTGGGAGGCATCAAACTGTCCCTTCCCAAGACTTAGGAGCCTTGTCCTGATCTCCTGCCTTAATCTTGAGGCTGTGCCACTTGAGCTTGCCGATTTGGATAACCTTCAAGAGATGACACTGGACAACACAAGCAAAGCAAGTGAATCTGCAAGAGAAATAGAACGCGAGAAGAAGAAGAAGCAAGCTGATCTAGAAAGTGGCAAGTTCAAGCTCACTATTCCCTATTGA

>PGSC0003DMG400029453

GAGCTGCCATTTGAGGAAGAAAGAAGAAGAATCAGGGATCTTATTGATGATTTCTTGAACGGCTTGAAGAAAATAAAGAATGAATTCATTGCTTCAAAATGGGATGACATTGAATTGCTAAGAATGGATCTGAGATTCCTAAGAACATTTGTCCTGTTTGGGAATTCCAGTTTGGATGACTTTTATGAGAGGATGTCACTGAATATAAGCAAATTCAATGAACTTGCTTGGTTAGTTTTCTATCATGAAGAAGATAAATTAATCCTAGCGAAATACGACATGGAATGTCTCACCCCTCTGCTGCTGGAAGAGATGAAAAGTTATTTGAGTTTGGAGAATGATTATTATGTAGCCACGACGACAGAGGACAAAATGTTTGAATACAGGGACAGACTATTCAAACACCTTCATGATCTACCAAAGTATTGTGCTCATTTACTTCTCCCCCTGATGAATGAATACATGATTCTTCGACAAGTATTCAGACATCTGAGAGATTTCTATGAGTTGGAATGCATAGCGGCCAACAAAACAAGAACTGAATATCTCTATCCTCGGTACCAAATGATAGTTGATAGAGTAACACAATTCTGTTTTGATCTTTGGACTGGAAAGAGTAAAGAAGAAATTGGTGATGATTACTATTTCCACAGGTATGACGTATCTGAATGCTCTTCCAAGATCACTTCTCTACTCATCGACATAATCCCTCTTGAGCTGGATATTCTATACATTTCTACTACGAAAATCAAGGAAGAACCAAGGTCAACTGAACTAAAAGGGTGGGTTAAGCAAATCCTAAAAGCATTTCCAAGAATTCTTCAAAATTATCTGATTCTTATCCAAGGACGCATGGAAGGTGCAGTAGCTGTCAATTACTCTCCAACTCAAAGCATTAATGTCATGATGGAGTTCCTATTGATTTTTCTCACCGATATACCAAAGTGCTTTATCCATCGTGACAAATTGAACGACATGTTGGCACATGTTGGAGTACTTACAAGGAAAATATCTATTCTGGTGAGCAAGCTGTTGGAGGAGAGCTCTGAGAATAATATCAACGAAGTGGACTTTTCAGCTCCAGACTTTTTGCAAGAAATTGAACAAATGAAGGGAGATATCAGACAGGTTTTTCTGAAAGATCCAGAGTCATCTCAACTTCGCTTTCCTATGGATGATGGTTTCCTCTTCATGAATCTTTTACTCAGACATTTAAATGATTTGTTCATTTCCAATGTCTATTCAGTTACTCTGATAAAGAAAGAAATTGGGATGGTGAAACAAAGCCTTGCATTCCTTAGATCATCTTTCGGGAAAGTCGGGCAAACATTGGATGACACTAGTGGAGTAGTTAAAGATTGTTGGGTGCGTGCTTTAGATGTGGCATATGAGGCAGAACATGTCATTAATTGCATTCTTGTTAGAGATAAAGCTCTCTCCCATTTAATCTTCTCACTTCCGAGGGTCACTGATAAGATCAAGCTTATTGTGTCAGAAGTCACCAGTTTAGGGCTGGAGGATAAGAATGGGGATGACCCCCTTGATGCAAAGTCTTCCGACGAGTCAATTGAGTCAACCTTGTCATCTTTTGTTGAGGTAACAGTAGGTCATGAGGAAGACGAATCCTGGATCATTGATCAGCTCCTTGATGAACATGAATCCGAGCTTGATGTCATTTCGATTGTAGGAATGCCAGGACTCGGTAAAACTACCCTGGCCAACAAAGTGTATAAAAATACATTAGTTGCTAGTCATTTCAATGTTCGTGCTTGGTGCACTGTTTCTCAAAAGTATAACAAGTCAAAGGTGTTGCTGGAGATTCTTCAGCAAGTTACTGGCTCGGAAGGAAAAGAGAGTGAGGATGACCTTGCTGACAAGGTACGAAGAGCACTATTCGATAAAAGGTACCTCATTGTCTTGGATGATGTGTGGGATATTGCAACAGGGGAGATGCTAATAGCATGTTTTCCAAAGGTTAAGAGAGGAAATAGAATTATCTTAACTAGCCGAAATAGTGAGGTAGGTTTGAAAGTTAAATGCCATAGTGATCTTCTCCACCTTCAACTTTTAACACCTGAAAAAAGTTGGGAGTTATTCGAAAAAAGGGTATTTGGAGATGAAGGAAGCTGCCCTGCTGAGTTGTCAAAAGTTGGACACAAAATTGTTGAGAAATGTCAAGGGCTTCCTTTGGCTCTTGTTTTGATTTCTGGAGTAATTGTTAAAGGAAGGAAAGGAAAGCAAAAGGATTTGTGGCTTAAGATTCAACATAATCTGGATTCTTTTATTTCAGCAAACAACAATTTGCAGATGATGAAAGTGATGCAATTAAGTTATGACCATTTACCACACCACCTGAAGCCGTTGTTGCTTTACATTGGAAGATCTCAAAAGAGCAAACGAACTCCAGTCGTTGAGTTGATGCAGTTGTTGATAGCAGAAGGGTTGGTGGATAATGATATCTGTTCCAATAGTAGTTTAGAGGAAGTAAGTCAAAGTTACTTGGATGCTTTAATTTCTAGAAGCCTGCTAATGGTGGATCATATCAAGAAGAGTAAGTCTTTTTCTGTTAGGATCAAGGTTTGCTATGTGCATGATGTTGTGCATGATTTTTGTTCAGTAAAAGCGAAAAAGGAAAAGTTTTTCAAGTTAATCAATTCAGGTGCTCCATTCCATGCTTCTTATTTCCTATACCGTTGTCTAACCATTCATACTAACTCCAACAAACTCCACAAAAAATGTGTTTTGTTCAATTCTAATAAGTGCTCAGCTTGCAGTAAGCATCTCATATCTGTGAAAGTGAGTGGTTCTCTTAATAACTCCAGGTATATCTGTCACACAAGACACTTGAGACTTCTTAGAGTGTTGCAACTGGATAGCATTATTCTGGAATATTCTTTAGTTGAAGAAATAGGCTCCCTATTTCATTTGAGGTTATTAAGGATTTGGGCTAGTATAAAATCCATCCCACTGTCGTGGGTGAACCTCCAGAATCTGGAAACTCTGTTGATCGAAACGGAATATCCCATCATGGTACTACTGCCCAGAATACTCAAACTGTCGAAACTGAAACACATGAGTGTCGTCCACTTAAGTTCTTTCTTTGAATTGGACGACAAATGGAACATTATTATGGATCAATCAACTAGAATATTGGAAGGTGAGAATTCAAAGTTAGAAGACTTGACAACTTTGTCCCAAGTTCTTATCTCATATACTGAAGGCATGAGTGATGCTCTGGGGAAGTTCCCAAATCTTCAGCACCTCAATTGCATCATCAAGGAACCGATAGATCCTCCTACACAAGGCGATTGGTTTCCCAAGCTTGATGTCCTTAATAAACTTGAATCACTCATCGCAAGATACCAGAAGGCATGGGCAGATTATCAGTACGATAATGATCCCGAGTTAAAAGACAAAATCCCACCTCCCAATGAATATCACTTCCCTACAAGATTGAAAGAGTTACGGTTGTATGGTTTTCCCCTGAGACCTGCTTTGTTTTCAGCAATCGCGGCATTGCCTGAGCTTGAAATTCTGGAGTTAATCGACTCTGCTTCTGTTGAGGACATGAAGTGGGATGCAAGTGAGGACATCTATCAAGGTCTTAAGACTTTGCGGTTGGTATCTGCTAAATTTTCAGAATGGCAAGTTGATTCGGAAACTTTTCCCAAGCTTGAGGAATTAATACTAGAATACTGTGAGGAGCTTATGGAGATCCCTTCTGCATTTGCGGATATAGAGACTTTAAAGTTCATTCGCGTGGTTTATATTAAGCCTGACCTTGGAGATTCAGCCATGGAGATTAAGAAAGATGTAGCAGCTTACACAGGAGAGGACAAGCTTGATATCCATTTATCATATGCGTATGACGAAGAAGAGGTAATTAATTTATTCATTTAA

>PGSC0003DMG400029504

ATGGAAAAAGAAAAATGGGTTAGAAGAAGAAGAAAGGAATGCCTACCATTTGAGGAAGAAAGAAGAAGAATCAGGGATCTTATTGGTGATTTCTTGAATGGCTTGAAGAAAATAAAGAATGAATTCATTGCTTCAAAATGGGATGACATTGAAGTGCTAAGAATGGAATTGAGATTCTTAAAAACATTTGTCCTGTTTGGGAATTCGAATTTGGATGGCTTTGATTACAAGATAGACCATAATATAGAAAAGTTCCATTCTCTAACTGATTTTATTTTCAATGAAGATGAATTTATCCTAGCCAAATACAACATGGATAGTCTTACGCCTTACCTGCTGAAAGAAATCAAAAGTTATTTGAGATTGGAGAATGTAGCCATGATGACTGAGGAAAATATGTTTGAATATATGGACAGCCTCCTCAAGAACCTCCATGATCTACCAAAGTATTCTTCTGATTTGCTTCTACCCCTCATGAGTGAATACAAGATTCTTCGGCAAGTATGCAGACATCTCAGACATTTCTATGAGTTGGAATGCAACAAAATTACAACAACAAAATTTCTCTATGCTCGGTACCAAATGACAGTTGATAGAGTAACACAATTCTGTTTTGATCTTTGGACTGGAAAGTATAAAAACTATACTTTCTTTGATAACTATGCCATCTATCGATGCTCTTCCGAGATCACTTTTCTACTCATCGACATAATCCCTCTCGAGCTAGAGGTTCTATACATTTGTACTTCTAAGCTCATCAAAGAATCAAGGTCAAAAGAACTAGAAAGATGTGTTAAGCAAATCTTAAAAGCATCTCCAAGGATTCTTCAAAACTATCTGATTCTTCTCCAACAAGGCATGGCAGGTGCACTTGCTGTCAATTATGCACCAACTCAAACAATTAATGTCATGATCGAGTTCCTGTTGATCTTTCTCACTGATATACCAAAGCGATTTATCCATCATGAAAAATTGAACGATATGTTGGCACATGTTGGAGTACTTACAAGGAAAATAACTATTTTGGTGACCAAGATGTTGGAGGAGAGCTCTGAGAATAATATCAATGAAGCGGACTTTTCAGCTTCAGACTTTTTGCAAGAAATTGAACAAATGAAGGGAGATATCAGACAGATTTTTTTAAAATCCCCGGAGTCATCTCAACTTCGTTTTCCTATGAATGATGGTTTCCTCTTCATGAATCTTCTACTCAGACATTTAAATGATTTACTCATTTCCAATGCTTATTCAGTTTCTATCATAAAAAAAGAAATTGGGATGGTGAAACAAAGCCTTGAATTCCTAAGATCATCTTTTGGGAAAGTCAAAAAAACTAGTGGAGTAGTCAAAGATTGTTGGTTGCATGCTTTAGATGTGGCATATGAGGCAGAACATGTCATTAATTCCATTCTTGTCAGAGATAAAGCTCTCTCGCATCTCATCTTCTCACTTCCGAATGTCATTGATAAGATCAATCTTATCGTGGCACAAGTCACCACTTTACAGCTGGAGGATAACAATGGGGATCACCACCTCCTTGATGCAAAGTCTTCCGAAGAGACAATTGAGTCAACCTCATCATCTTTTGTTGAGGTAACAGTAGGTCATGAGGAAGAAGAAGCCCGGATCATCTGCCAGCTCCTTGATGAACACGAATCCGAGCTTGATGTCATTTCGATTGTCGGAATGCCAGGACTCGGTAAAACTACTCTGGCCAACAAAGTGTATAAAAATACATTAGTTGCTACTCATTTCAATGTCCGTGCTTGGTGCACTGTTTCACAAAAGTATAACAAGTCAAAGGTGCTGCGGGGGATTCTTCAGCAAGTAACTGTCTCGGAAAAAAAAGAAAGTGAGGATGACCTTGCTGAAAATCTACGAAGAGCACTACTCGATAAAAGGTACCTTATCGTCTTGGATGATGTGTGGGATATTGAAACAGGGGAGATGTTAATAGCATGTTTTCCAAAGGTTAAGAGAGGAAATAGAATTATCTTAACCAGCCGAAGTAGTAAGGTAGGTTTGCAAGTCAAATGCCATAGTGATCCTCTCCACCTTCAACTTTTAACACCTGAAAAAAGTTGGGATTTATTCGTAAAAAAGGTATTTGGAGATGAAGGAAGCTGCCCTGCTGAACTGTCAGAAGTTGGACACCAAATAGTTGAGAAATGTCAAGGACTTCCCTTGGCTATTGTTTTGATTGCTGGAGTAATTGTTAGAGGAAAGAAAAAGGAAAAAGATTTGTGGCTTCAGATTGAACATAATCTGGATTCCTTTATTTCTTCAAACAACAATTTGCAGATGATGAAGGTTATGCAATTAAGTTATGACCATTTACCATACCACCTGAAGCCGTTGTTGCTTTACTTCGCAAGATCTCAAAAGAACAAACGAACTCCAGTCTCTACATTGATGCAGTTGTGGATGGCCGAAGGGTTGGTGGATCATGATAGTTCAGAGGAAGTAACTCAAAGTTACTTGGATGCTTTAATCTCCAGTAGCCTGATAATGGTTGATCATATCCCCTCCGAGAGTGTTTTGATGATGTCTGCAATAATCAAGGTTTGCTATATGCATGATGTTGTGCACGATTTTTGTTCAGTAAAAGCGAAAAAGGAAAAGTTTTTCAAGTTAATCAATTCAGGTGATCCATTTCATGCTTCGGATTTCCTACACCGTCGTCTAACCATTCATACTGATGACAGCCCAATCCACAAAAGATGTGTTTTGTTCAATTCTAATAAATGTTCAGGTGGTAGTAAGCATCTCATATCTTTGACAGTGAGTGGCTGGCTTGATTACTCCAGTTACATCTGTCACACAAGACACATGAGACTTGTTAGAGTGTTGCAACTGGATGGCATTGTTCTGGAAGATTCTTTAATGGAAGAAATAGGCTCCCTATTTCATTTGAGGTTCTTAAGAATTCAGACTAGTATAAAAACTATCCCAGTGTCGTGGGTGAACCTCCAGAATCTGGAAACTCTGTTGATCAATACAGAAATAACCATGGTATTACTGCCCAGAATATTGGAACTGTCAAAGCTGAAATATGTGAGCATTGACCAGAGTTCTTTCTTTGACGAAGAGGATGCAGACGAAAGTAGAATATTGGAAGCTGAGAATTCAAAGCTGACAAATTTATCCCATGTTGATATCTCGTATTCTGAAGGCACGAATGATGCTCTGAAGAAGTTCCCAAATCTTCAACACCTTCAGTGCACCATCAGGATACCCAAGGATCATCCTACACATGGCGATTGGTTTCCCAAGTTTGATGTCCTTAACAAACTTGAATCACTCTTTGCAGAATACCACAACCCATGGAACGATTATGTTTATCCCAATGAATATCACTTCCCTAGCAGCTTGAAAGAGTTGCGGTTGTATAATTTTCTCGTGAGACCTGCTTTGTTGTCAGCAATCGCGGAATTGCCTCAGCTTGAAATTCTGGAGTTTGTAGAGTCTAATTTCGTGGAGGATAAGTGGGATGCAAGTGAGGACATCTATCGAAGTCTTAAGATTTTGAGTTTGGTATTAGCCAAATTATCAGAATGGGAAGTTGATAGGGAAACTTTTCCCAAGCTTGAGGAATTAATACTAGAAAATTGTTACGAGCTTACGGAGATCCCTTCTGTATTTGGGGATATAGACACTTTAAAGTCTATTCAAGTGGTTCAAAGTAAGCGTGAGGTTGGAGATTCAGCCATGGAAATTAAGAAAGATGTGGCAGCTTACACAGGAGAGGACAGACTTCATGTCCATATATCAGATGTGTATGAATAA

>PGSC0003DMG400006531

ATGGCTGAGGCCTTTGTATCATTTGCAGTTCAAAAATTGGGTGATCTACTCATACAACAAGTTTCCCTGCGTAAAAATCTAAGAGAAGAAGTTACATGGCTGCGAAACGAGCTTCTCTTAATGCAGTCTTTCCTCAAAGATGCAGAACAAAAGCGAAGTGGAGATGAAAGAGTTGAACAATGGGTGCTTGAGATCAACTCTATTGCTTATGACGTTGTTGCTATACTCAAGACTTATAGCATCGAGGATGGTAAATGTGCTAGTTGTCTCAAGGTTTGCGCTTGCATCTGTAGGAAGAAATCCTACAATGTCGCCAAAGAGATCCAATCACTCAAGCAACGAGTCATGGATATCTCTGTCAAGCGAGAGACTTATGATATTACAAATACTATCAATTATAATGCAGGAAAAGGGACAAGTAATAAGGTTAGAACATTGAGGAGAACTACCTCATATGTGGATGACCAGGATTACATTTTTGTTGGACTTCAAGATGTTGTACAAACATTGGTATATGAACTTCTCAAAGCAGAGCCTCGTCGAAGCGTCCTCTCCATTTATGGAATGGGCGGTTTAGGCAAGACCACTCTTGCCAGAAAACTTTACATCAATCCTAATATAGCCTCTAGCTTCCCAACATGCGCTTGGATATGTGTTTCTCAAGAGTACAACACAATGGATCTTCTTTCGACTATCATAAAATCCATCCAAGGTCGCACCAAGGTAACTCTAGATTTGTTGGAAAGCATGCCAGAAGGAGATCTAGAAATTTATCTTCGTGATCTATTAACAGAACGCAAATACCTTGTGGTGGTTGATGATGTACGGCAGAAAGAAGTATGGGAGAGATTGAAAAGAGCATTCCCGGATAGCAAGAATGGCAGCAGAGTCATTATTACCACGCCCAAAAAGGATGTCGCTGAAAGAGCGGATAACAGAGGTTTTGTCCATGAACTTCGTTTCCTAACCCAAGAAGAAAGTTGGGATCTCTTTCGTAGGAAACTACTTGATGTTCAAGCAATGACCTTCACTATGGAGAGGGTAGCTAAGAATATGGTGGGAAAGTGTAGAGGCTTACCTCTTGCAATTGTTGTATTGAGCGGACTACTTTCACATAAAAAGAGGCTAGATGAATGGCAAAAGGTGAAAGATCACCTTTGGAAGAACAATGTTGAAGATGAATATATTGAAATCTCCAACATACTATCATTAAGCTACAATGATTTGTCAACTGCGCTCAAGCAGTGTTTTCTCTACTTTGGTATTTTTCCAGAAGATCAAGTGGTCAAGGCTGAAAACATAATACGGTTGTGGATGGCGGAGGGTTTCATACCAAGAGGAGAAGAAAGAATGGAGGATGTCGCTGAAGGCTACTTGAATGAGCTGATAAGACGAAGCTTGATACTAGTTGTACATACATTTTGGGAAAGAGTTACTGCATGTAGGGTTCATGATTTACTCCATGATCTTGCGATACAAGAGGCATTGAAAGTAAACTTCTTTGGCATTTATGATCCAAAAAGACACTCCATATCCTCTTTATGTATCAGACATGTCATTCATAGTCAAGGAGAAAGGTACCCCTCACTTGATCTTTCTAACTTAAAGTTGAGGTCAATTATGTTCTTCGATCGTAAGATTAGTCTTATAAATTTCAGTAGTGTGTTCCAACATCTCTATGTGTTATACTTGGAGATGTGTGTTGACAAGAATCCACATCCACATTTAGTTCCTGATGCCATAGGAAGTTTGTACCACCTCAAGTTCTTAAGATTGAGAGGTCGTATCCATGATCTTCCCACTTCCATTGGCAACCTCAAGAATCTACAGACACTTGTTGTCAATGGATACTCTTGCAAACTACCCCAAGAGACAGCTGACCTAATAAATCTAAGACATTTAGATGCTCGGTATTCAGAATCTCTGAAACATCTAAGCAAACTCACTAGCCTTCAAGTTCTTAAAGGCGTTTATTGTGATCAGTGGAAAGATGTTGACCCTGTTGATTTAGTCAACCTTCGAGAATTAACCATGTGTAGTATTTGGAACTCTTACTCCCTAAAGAACATTAGCAGCTTGAAAAACCTTAGCACTCTCAGATTGTTTTGTGAACGTGATGAATTATACACATCTCCATTCCCATCCCTTGAATTTCTTTATTCTTGTGAAAAGCTGCAGAAATTGTGGTTAAAAGGGAGAGTAGAGAAACTGCCTCTGTTTCCAAATTCCATCACAATGATGGTTCTTTGGCAGTCAAGACTCATGGAGGATCCGATGCCTATTTTGGGAAAGTTGCCAAACCTAAAAGATCTCATATTACAAGAAGCTTATAAAGGAAAAGAAATAATGTGCAGCGATAACACCTTCAGTCAACTAGAGTCCCTTCGTCTTTATGATCTTTCAAACCTAGAACGATGGCATTTAGACACAAGTGCCATGTCTCTCATTAAAGGTCTTCATATCCATGCTTGTCCAGAGCTGAAGGAGATTCCAAAGAGAATGAAAGATGTTGAAGAGAAATTAAATATGGTGGAGCTTTACAAGCATATGTGA

>PGSC0003DMG400006533

ATGGCTGATGCCTTTGTATCATTTGCAGTTCAAAAATTGGGTGATTTCCTCATACACGAAGTTTCCCTGCGTACAAGTCTCAGAGATGAAATTAGATGGCTGAGAAATGAGCTACTCTTCATGCAGTCATTCCTAATAGATGCAGAACAAAAACAAAGTGGAGATCAAAGAATTCAACAATGGGTGTTTGAGATCAACTCGATTGCTAATGACTCTGTTGCTATACTCGAGACTTACAGCTTCGAGGCTGGTAAAGGTGATAGTCGTCTCAAGGTTTGTGCTTGCATCTGTATGAAGGGGAAGAAATTCTACAATGTCGCCAAGGAGATTCAATCACTCAAGCAACAAATCATTGATATCTCTCGCAAACGAGAGACATATGGTATTACAAATATCAATAGTAATGCTGGAGAAGGTACAAGTAATCAGGTTAGAACATTGAGGAGAACTACCTCATATGTGGATGACCAGGATTACATTTTTGTTGGCTTTCAGGATGTTGTACAAACATTGCTAGCTCAACTTCTCAAACCAGAGCCTCGTAGAAAGGTCGTCTCCATTTATGGAAAGGGTGGATTGGGCAAGACTAATCTTGCGAGAAAACTCTACACCAGTCCTATTATAGCCTCTAGCTTCCCTACACGCGCTTGGATATGTGTTTCTCAAGACTACAACACAATGGATCTTCTTAAGACTATCATTAAATCCATCCAAGGTTGCACCAACGAAACTCTAAATTTGTTGGAAAGGATGACAGAAGGAGATCTAGAAATTTATCTTCGTGATCTATTAAAAGAACGCAAATACCTTGTGGTGGTTGATGATGTATGGCAGAATGAAGCATGGGAGAGTTTGAAAAGAGCATTCCCGGATAGCAAGAATGGCAGCAGAGTCATTATTACCACGCGCAAAGAGGATGTCGCTGAAAGAGCAGACGACAGAGGTTTTGTTCATAAACTTCGTTTCCTAAGTCAAGAAGAAAGTTGGGATCTCTTTCGTAGGAAACTACTTGATGTTCGAGCAATGGTTCCAGAAATGGAAAATCTAGCTAAGGAAATGGTGGAAAACTGTAGGGGCTTACCTCTTGCAATTGTTGTATTGAGCGGACTACTTTCGCATAAAAAAGGGCTAAAAGAATGGCAAAAGGTGAAAGACTGCCTTTGGAAGGACATTGAAGAAGACTCTTTTCATGAAATCTCCTCCATACTATCATTAAGCTACAACGATTTGTCAACTGCGCTCAAGAAGTGTTTTTTGTACTTTGGTATTTTTCCAGAAGATCAAGTGGTCGAGGCTGATAACATAATACGGTTGTGGATGGCAGAGGGTTTCATTGTACCAAGAGGAGAAGAAAGAATGGAGGATGTCGCTGAAGGCTTCTTGAATGAACTGATAAGACGAAGCTTGGTTCAAGTGGCTCAAACATTTTGGGAAAGAGTTACTGAGTGTAGGGTTCATGATTTACTTCGTGATCTTGCGATACAAAACGCATTGGAGGTAAAGTTCTTTGACATTTATGATCCAAGAAAGCACTCCATATCTTCTTCATGTATCAGACATGCTATTCATAGTCAAGGAGAAAGGTACCTCTCATTTGATCTTTCTAACTTAAAGTTGAGGTCAATTATGTTCTTCAATCGGGATTTTTGTAATGTGTTCCAACATATAGATGTGTTTCGACATCTATATGTGTTGTACTTGGATATTAAAGAAGGTGGTGTTATACCTGATGCCATAGGGAGTTTGTACCACCTCAAGTTGTTAAGCTTGAGAGGTATTGATAATCTTCCCTCCTCCATTGGCAACCTCAAGAATTTACAGACATTTGTCGTTGTCAATGAGGACGGATCATTTTGCCAACTACCCCCCAATACAGCTAACCTAATAAATCTAAGACATTTAGTTGCTCCGTATTCAGAGCCTCTGGTACGTATAAACAAACTCACTAGTCTTCAAGTTGTTGATGGCATTGCTTGTGATCAGTGGAAAGATGTTGACCCTGTTGATTTAGTCAATCTTCGAGAATTAAGTATGCATTATATTAACAAATCTTACTCCCTAAAGAACATTAGCAGCTTGAAAAACCTTAGCACTCTCAGATTGTCGGGTGAGTATGGTAACTCATCTCCATTCCCATCCCTTGAATTTGTTAATTGTTGTGAAAAGCTCCAGAAATTGTGGTTAGATGGGGGAGTAGAGAAACTGCCTGTGTTTCCAAATTCCATCACAATGATGGTTCTTATAGACTCAAAACTCATGGAAGATCCAATGCCTATTTTGGGAATGTTGCCAAACTTAAGGAATCTCGAATTATTAGTAGGAGCTTATGAAGGAAAAGAAATAATGTGCAGTGATAACAGTTTCAGTCAACTAGAGTTCCTTCGTCTTTATGATCTTGAGAATCTAGAAACATGGCATTTAGCCACAAGTGCCATGCCTCTCATTAAAAGTCTTGCTATCAGTCGCTGTCCAAAGCTGAAGGAGATTCCAGAGAGAATGAAAGACGTGAAATGTATTTCATAA

>PGSC0003DMG400011529

ATGGCTGATGCCTTTGTGTCATTTGCAGTTCAAAAATTGGGTGATTTCCTTATACAACAAGTTTCCCTGAGATCAAATCTGAGAGATGAAGTTAGATGGCTGAGAAATGAGCTATTCTTCATACAGTCTTTCCTCAGAGATGCAGAACAAAAGCAATGCGTTGATCAAAGAGTTCAACAATGGGTGTTTGAAATCAACTCTATTGCTAATGACGCTGTTGCTATACTCGAGACTTATAGCTTCGAGGCTGGTAAAGGTGCTAGTCGTTTCAAGGCTTGCGCTTGCATCTGTAAGAAGGAGAAGAAATTCTACAATGTCGCCAAGGAGATTCAATCACTCAAGAAACGAATCATGGATATCATTCGCAAGAGAGAGACTTATGGTATTACAAATATCAATAGTAATGCTGGAGAAGGGACAAGTAATCAGGTTACAACATTGAGGAGAACTAACTCATCTGTGGATGACCAGGATTACATTTTTGTTGGCTTTCAGGATGTTGTACAAACATTGCTAGCTGAACTTCTCAAAGTAGAGCCTCGTCGAAGCGTCCTCTCCATTTATTGCATGGGCGGATTAGGCAAGACCACTCTTGCAAGAAACCTCTACAGAAGTCCTAATATAATCTCTAGCTTCCCTACACGCACTTGGATATGTGTTTCTCAAGAGTACAACAAAATGGATCTTCTTAAGACTCATAAAATCCATCCAAGGTCGCACCAAGGAAACTCTAGATTTGTTGGAAAGGATGACGGAAAGAGATCTAGAAATTTACCATCACTATTGAAAGAATGCAAATACCTTGTGGTGGTTGATGATGTATGGCAGAGAGAAGCATGGGAGAGTTTGAAAAGAGCATTCCCGGATAGCAAGAATGGCAGCAGAGTTATTATTACCACGTGGAAAGTGGATGTCGCTGAAAGAGCAGACGACAGAGGTTTTGTCCATAAACTTCGTTTCCTAAGCCCAGAAGAAAGTTGGGATCTCTTTTGTAGGAAACTGCTTGATGTTGGAGCAATGGTTCCAGAAATGGAAAGTCTAGCTAAGGATATGGTGGAGAAGTGTAGAGGCTTAGCTCTTGCAATTGTTGTATTGAGCGGACTACTTTCGCATAAAAAGGGGCTAGACGAATGGAAAAAGGTGAAAGATCACCTTTGGAAGAACATTAATATCTCCAACATACTATCATTAAGCTACAATGATTTGTCAACTGCGCTCAAGCAGTGTTTTTTGTACTTCAGTATTTTTCCAGAAGATAAAGTGCTCGAGGCAGAAAACATAATATGGTTGTGGATGGCTGAGGGTTTCATACCAAGGGGAGAAGAAAGAATGGAGGATGTCGCAGAAGGCTTCTTGAATGAGCTGATAAGACGAAGCTTGGTTCAAGTAGCTAAAACATTTTGGGAAAAAGTTACTGAATGTAGGGTTCATGATTTACTTCGTGATCTTGCGATACAAAAGGCATTGGAGGTAAACTTCTTTGACATTTATGATACAAGATACCACTCCATATCCTCCTCATGTATCAGACATGCCATTCATAGTCAAGGAGAAAGGTACCTCTCACTTGATCTTTCTAACTTGAAATTGAGGTCAATTATGGTCTTTGATCACAATTTTTCTAACACAAGTCTTATAAAGTTCAGTAGTGTGTTCCGACATTTATATGTGTTGTACTTGGATATTGAAGTTGGTTTTACACCTGGTGCCATAGGAAGTTTGTACCACCTTAAATTCTTACACTTGAGAGGTGTCTGTTATATTCCCTCTTCCATTGGCAACCTCAAGAATTTACAGACACTTCATGCCAATAGTAACTTTTTCTCATGCCACCTACCCCCCGAAACAGCTGACCTAATAAATCTAAGACATTTAGTTGCTCCGTATTCAAAACCTCTGAAACGTATAAGCAAACTCACAAGTCTTCAAGTTCTTAAAGACATTTATTGTGATCAGTGGAAAGATGTTGACCCTGTTGATTTAGTCAATCTTCGAGAATTAAGCATGACTAATATTACCAGATTTTACTCCTTAAACAACATTAGCAAATTGAAAAACCTTAGCACTCTCAGATTGTTGTGTCCTGGTGATGGACCATTCCCATCTCTTGAATTTGTTAATTGTTGTGTAAAGCTCCAGAAATTGTTCTTAAATGGGAGAATAGAGAAACTGCCTGATATGTTTTCAAATTCCATCACAATGATGATTCTTCAGTACTCACAACTGATAGAAGATCCGATGCCTAATTTGGGAATGTTGCCAAACCTAAGGGATCTCCAATTGAGAGGAGCTTATAAAGGAAAAGATATAACCTGCAATGATAACAGCTTCAGTCAACTAGAGTTCCTTCGTCTTGATAGTCTTGGGAGGCTAGAAAGATGGCATTTAGGCACAAGTGCGATGCCTCTGATTAAAGGTCTTTATATCTATGATTGTGAAAGGCTGAAGGAGATTCCAGAGAGAATGAAAGACGTGGTGAAGTTTTCCAAGCCTATATATGGGCGATAA

>PGSC0003DMG400011528

ATGGCTGATGCCTTTGTGTCATTTGCAGTTCAAAAATTGAGTGATTTCCTCAGTAGCCAAGTTTCCCTGAGAACAAATCTCAGAGAGGAAGTTGAGTGGCTGATAAAAGAGCTACACATCATAAAGTCATACCTCGGAGATGCAGAACAAAAGCAAACTGGAGATAATAGAGTTCAACAATGGCTGTTTGAAATCAACTCTATTGCTAATGACGCTGTTGCTATACTCGAGACTTACAGCTTCGAGGCTGGTAAACATGCTAGTTGTCTCAAGGCTTGTGCTTGCATATGTAGGAAGGAGAAGAAATTCTACAATGTGCTCGAGGAGATTCAATCACTCAAGCAGCGAATCATAGATGTCTCTGGCAAGCAAGAGAATTATGGTATTACAAATATCAATTATAATGCAGGAGAAGGGCCAAGTAATCAGGTTACAACATTGAGGAGAACTACCTCACATGTGGATGACCAGAATTACGTTTTTGTTGGACTTCAGGATGTTGTAGAAAAATTGCTAGATGAACTTCTCAGAGCAGAGGCTCGTCCAAGCGTCATCTCCATTTGCGGAATGGGTGGTTTAGGCAAGACCACACTTGCGAGAAACCTCTATATCAATCCTAATATAGTCAATAGCTTCCATATGCGTGCTTGGATATGTGCTCCTGAAGAGTACAACACCGTGGATGTCCTCAAGAATATCATAAAATCCATCCAATCTTGCAGCAAGGAAATTCTAGATTGGTTGGAAAGGATGACAGAAAGAGATTTAGCAATATACCTTCGTGCTCTTTTAAAAAAACGCAAATACCTTGTCGTGGTTGATGATCTATGGCATAGAGAAGCGTGGGAGAGTTTGAAAAAAGCATTCCGAGATGGCAAGAAAGGCAGCAGAGTCATTATTACCACGCGCAGAGTGGATATCGCTAAAACAGCAGACGAAGGTTTTGTCCATAATCTCCGTTTCCTAAGCCAAGACGAAAGTTGGGATCTCTTTTGTAGGAAACAACTCCATGTTCAAGCAATGGTTCCAAAAATGGAAAGGTTAGCTAGGGATATGGTGGAAAAGTGTGGAGGCTTACCTCTTTCAATTGTTGTATTGAGCGGACTACTTTCGCATAAAAGGGGGCTAGACGAATGGCAAAAGGTGAAAGATCACCTTTGGGAGAACATTATTGAAGATGAATGTATTGAAATCCCCAACATATTATCATTAAGCTACAACGATTTGTCAATTGCGCTCAAGCAATGTTTTTTGTACTTTGGTATTTTTCCAAAAGATCAAGTGGTCGAGGCTGAAAACATAATACGCTTGTGGATGGCGGAAGGTTTCATACCAAGAGGAGAAGAAAGAATGGAGGATGTCGCTGAAGGCTTCTTGAATGAGCTGAAAAGACGAAGCTTAGTTCAAGTGGTAGATACATTCTGGGAAAAAGTTGAAGTATGTAGTGTTCATGATTTACTTCATGATCTTGCGATCCATAAGGCATTGAAGGTAAACTTCTTTGACATTTATGATCCAATAAACCATTCCATATCCTCTTCATGTATCAGACATGGCATTCATAGTCAAGAGAAAAGGTACCTCTCACTTGATCTTTCTAACTTGAAGTTGAGGTCAATTATGTTCTTCAATCTAGGATTTCGTAACATGAGTCATATAAACTTCAGTAGTGTGTTCCAACATGTATATGTGTTGTACATGGATATTATTGATGGCACTTTACCTGATGCCGTAGGAAGTTTGTACCACCTCAAGTTCTTAAGATTGACAGGTGTTATTCATGATTTACCCTCCTCCATTGGCAGCCTTAAGAATTTACAAACACTTTGTGTCAAATGTAATTTCAAACAGTTCTGCCAACTACCCCCCGAAACAGCTGACCTAATAAATTTAAGATATTTAGGTGTTCCCTATTCAGAACCTCTGAATCGTATAAGAAAACTCACAAGTCTTCAAGCTCTCGAAGGCGTCTCTTGTGATCAGTGGAAAGATGTTGATCCTGTTGATTTAGTCAATCTTCAAGAATTAAGCATGATTAGTATTACCAAATCTTACTCCCTCAACAAGATTAGCAACTTGAAAAACCTTAGCACTCTCAAGTTGTTGTGTTATGCTGATGAATCATTCCCATCTCTTGAATTTCTTATTTCTTGTCATAACCTCAAGAAATTGTTGTTAGAAGGTAGAATAGAGAAACTGCCTTTGTTTCCAAATTCCATCACAATGCTGACTCTTGTCGACTCAAAACTCATGGAAGATCCGATGTTTATTTTGGGAATGTTGCCAAACCTAAGGAATCTCTGTTTGTTTAGAGCTTATCAAGGAAAAGAAATAATCTGCAGTGATAACAGTTTTAGTCAACTGAAGTTCCTTAGTCTTGATTGTCTTTGGAACCTAGAAAGATGGAATTTAGCAACAAGTGCCATGCCTCTGATCAAAGCTCTTCGTATCGATCGCTGTCTAAAGCTGAACCAAATTCCTGAGAGAATGAAGGATGTGGAGCCATTGCCTATTGAAGAGAAATTAAATGCGCTGAAGCTTTTCAACCATATGTGA

>PGSC0003DMG400011527

ATGGCTGATGCCTTTGTGTCATTTGCAGTTCAAAAATTGGGTGATTTCCTCATTCAGGAAATTAACCTACGTTTAAGCCTAAGAGAGGATATGCAGTGGCTCCGAAACGAGCTTCTCTTCATGCAGTCTTTCCTCAAAGATGCAGAACTAAAACAATGTGGAGATCATAGAGTTCAACAATGGGTGTTTGAAATCAATTCTGTTGCGAATGATGCTGTCGCTATACTCGAGACTTACAGCTTTGAGACTAGTAAACGTGCTAGTCGTCTCAAGGCTTGCGCTTGCATATGTAGGAAACAGAAGAAATTCCACAATGTCGCTGAGGAGATTCAATCACTCAAGCAACGAGTCATGGATATCTCTCGTAAACGAGATACTTATGGTATTACAAATATCAATAGTAATGCTGGAGAAGGGCCAAGTAATCAGGTTAGAACATTGAGGAGAACTACCTCATATATAGATGATGATCACATTTTTGTTGGCTTTCAGGATGTTGTACACACATTGCTATCTGAACTTCTCAAAGCAGAGCCTCGTCGAAGAGTCCTCTCCGTTTATGGAATGGGCGGTTTAGGCAAGACCACTCTTGCGAGGAAACTTTACACCAGTCCTAATATAGCCTCTAGCTTCCTTACACGAGCTTGGATATGTGTTTCTCAAGAGTACAACACAATGGATCTTCTTAAAACTATCATAAAATCCATCCAAGGTTGCGCCAAGGAAACTCTAGATTTGTTGGAAAAGATGGCAGAAATAGATCTAGAAAATCACCTTCGTAAACTATTAACAGAACGCAAATACCTTGTGGTGGTTGATGATGTATGGCAGAGAGAAGCATGGGAGAGTTTGAAAAGAGCATTCCCGGATAGCAAGAATGGCAGCAGAGTCATTATTACCACACGCAAAGAGGATGTTGCTGAAAGAGCAGACGACAGAGGATTTGTCCATAAACTTCGTTTCCTAACCCAAGGAGAAAGTTGGGATCTCTTTTGTAGGAAACTGCTTGATGTTCGAGCAATGGTTCCAGAAATGGAAAGTCTAGCTAAGGATATGGTGGAGAAGTGTAGAGGCTTACCTCTTGCAATTGTTGTATTGAGCGGACTACTTTCGCATAAAAAGGGGCTAGACGAATGGCAAAAGGTGAAAGATCACCTTTGGAAGAACATCATTGAAGATAAATCTATTGAAATCTCCAACATACTATCATTAAGCTACAATGATTTGTCAACTCCGCTCAAGCAGTGTTTTCTCTACTTTGGTATTTTTCCAGAAGATCAAGTGCTCGAGGCTGATAACATAATACGGTTGTGGATGGCGGAGGGTTTCATACCCAGAGGAGAAGAAAGAATGGAGGATGTTGCTGAAGGCTTCTTGAATCAGCTGATAAGACGAAGCTTGGTTCAAGTGACTAAAACATTTTGGGAAAAAGTTACTGAATGTAGGGTTCATGATTTACTCCATGATCTTGCGATACAAAAGGCATTGGAGGTAAACTTCTTTGACGTTTATGATCCAAGAAGCCACTCCATATCCTCTTTATGTATCAGACATACCATTCATAGTCAAAGAGAAAGGTACCTCTCACTTGATCTTTCTAACTTAAAGTTGAGATCAATTATGTTCTTCGATCCAGATTTTTGTAAGATGTGTCTTATAAACTCCAGTAGTGTGTTCCAACATCTATATGTGTTGTACTTGGAGATGCGTGTTGACGACAGGTATTTTGTATTTGTACCTGATGCCATAGGAAGTTTGTACCACCTCAAGTTCTTAAGATTAGGAGGTATCCATGATCTTCCGTCTTCCATTGGCAACCTCAAGAATCTACAGACACTTGTTGTCAATGAGGGCAGACACAATTGTGTACTACCCCCCGAGACAGCTGACCTAATAAATCTAAGATATTTAGATGCTCTGTATTCACCACCTCTGAAACGTCTAAGCAAACTCACTAGTCTTCATGTTTTTAAAGGCATTGGTTGTAATCAGTGGGAAGATATTGACCCTGTTGATTTGGTCAATCTTCGAGAATTAAGTATGCGTTATATTAACATATATTACTCCCTAAGCAAGATTAGAAGATTGAAAAACCTTAGTACTCTCAGATTGTTTTGTGATGAACGTATTGTAACAGACTTATATCGATTCCCATCCCTTAATTTTCTTAATTGTTGTGAAAAGCTCCAGAAATTGTGGTTAAAAGGGAAAATAGAGATACTGCCAGATCTGTTTCCAAATTCCATCACCATGATGGTTCTTTGGAGGTCACAACTCACAAAAGATCCGATGCCTATTTTGGGAATGTTGCCAAACCTAAGAGATCTCATATTGCAAGAAGCTTATAATGGAAAAGAAATAATGTGCAGTGATAACAGCTTCCGTCAACTAGAGTTCCTTCATCTCTATCATCTTTGGAATCTAGAAAGATGGCATTTAGCCACAAGTGCCATGCCTCTCATTAAAGGTCTTGCTATCGATCGCTGTCCAAAGCTGAAGGAGATTCCAGAGAGAATGAAAGATGTTGAAGAGAAGTTAAATGTGGTGGATCTTCACAAGCATATGGGAGGTTTTATTTTTTAA

>PGSC0003DMG401011526

ATGGCTGATGCCTTTGTGTCATTTGCAGTTCAAAAATTGGATGATTTCCTCATTCAGGAAATTAACTTACGTTTAAGTCTAAGAGAGGATATACAGTGGCTGCGAAACGAGATTCTCTTCATGCAGTCTTTCCTCAAAGATGCAGAACTAAAACAATGTGGAGATCATAGAATTCAACAATGGGTAATTGAGATCAACTCTATTGCTAATGACGCTGTTGCTATACTCGAGACTTACACCTTCGAGGCTGGTAAACGTGCTACTCGTCTCAAGGCTTGCCCTTGCATATGTAGGAAGGAGAAGAAATTCTACAATGTCACCAAGGAGATCCAATCACTCAAGCAACAAATCATGGATATCTCTCGTAAGCGAGAGACTTATGGTATTACAAATATCAATAATAATGCAGGAGAAGGACCAAGTAGTCAGGTTAGAACATTGAGGAGAACTACCTCATATGTAGATGAGGATCACATTTTTGTTGGCTTTCAGGATGTTGTACAAACATTGCTAGCTGAACTTCTCAAAGCAGAGCCTCGTCGAAGCATCCTCTCCATTTATGGAATGGGCGGATTAGGCAAGACCGTACTCTTGCCAGAAACCTCTACACAAGTCCTAATAGTCTCAAGCTTCCCTACACGCGCTTGGATATGTGTTTCTCAAGAGTACAACACAATGGATCTTCTTAAAACTATCATAAAATCCATCCAAGGTTGCGCCAAGGAAACTCTAGATCTGTTGGAAAAGATGGCAGAAAGAGATCTAGAAAATCACCTTCGTAAGCTATTAACAGAACACAAATACCTTGTGGTGATTGATGATGTGTGGCAGAGAGAAGCATGGAAGAGTTTGAAAAGAGCATTCCCGGATAGCAATAATGGCAGCAGAGTTATTACCACGCGCAAAGTGGATGTCGCTGAAAGAGCAGACAACAGAGGTTTTGTCCATGAACTTCGTTTCCTAAGCCAAGAAGAAAGTTGGGACCTCTTTTGTAGGGAACTACTTGATGTTCGAGCAATGATTCCAGCGATGGAAAGTCTAGCTAAGGATATGGTGGGAAAATGTAGAGGCTTACCTCTTGCAATTGTTGTATTGAGTGGACTACTTTCGCATAAAAGGGGGCTAGACAAATGGCAAAAGGTTAAAGACTGCCTTTGGAAGGACATTGAAGAAGACTCTATTGAAATCTCCTGCATACTATCATTAAGCTTCAACGATTTGTCAGCTGCACTCAAGCAGTGTTTTCTGTACTTTGGTATTTTTCCAGAAGATCAAGTGGTCGAGGCTAATATCATAATACGGTTGTGGATGGCAGAGGGTTTCATCATACCCAGAGGAGAAGAAAGAATGGAGGATGTCGCTGAAGGTTTCTTGAATGAGCTGATACGACGAAGCTTGGTTCAAGTGGCTAAAACATTTTGGGAAAAAGTTACTAAATGTAGTGTTCATGATTTACTCCATGATCTTGCGATACAAAAGGCATCCGACACAAACCTCTTTGACATTTATCATCCAAGAAAGCACTCCATATCCTCTTCATGTACCAGACTTGCCATTCTTAGTCAAGGAGAAAGGTACCGCTCACTTGATCTTTCTAATTTGAAGTTGAGGTCAATTATGTTCTTCGATCCATATATTTATAATGTGTTCCAACATATAGATGTGTTTCGACATCTATATGTGTTGTACTTGGATATTAAAGGTGGTGTTATACCTGATGCCATAGGGAGTTTGTACCACCTCAAGTTGTTAAAATTGAGAGGCATCCATAAGCTCCCCTCCTCAATTGGCAACCTCAAGGATTTACAGACACTTGTTGTTGTCAATGAGGATGGATCATTTTGCCAACTACCCCGTGAAACAGCTGACCTAATAAATTTAAGACATTTAGTTGTTCCATATTCAAAACCTCTGAAACGTATAAGTCAAATCACTAGCCTTCAAGTTCTTGATGGCATTGGTTGTGATCAGTGGAAAGATGTTGACCCTGTTGATTTAGTCAATCTTCGAGAATTAGGCATGGATAATATTAGCAAATCTTACTCCCTAAACAACATTAGCAGCTTGAAAAACCTTAGCACTCTCAGATTGTTTTGTAAGTATGACGGATCATTCCCATCCCTTGAATTTGTTAATTGTTGTGAAAAGCTCCAGAAATTGTTCTTATATGGGAGAATAGAGAAACTGCCTCATCTGTTTCCAAATTCCATCACAATGATGATTCTTTTGAAGTCAAAACTCATGGAAGATCCGATGCCTATTTTGGGAATGTTGCCTAACCTAAGAAATCTCGGATTACTTTATGCTTATGAAGGAAAAGAAATAATGTGCAGTGATAACAGTTTCAGTCAACTACAACTTCTTACTCTTAATGATCTTTACAACCTAGAAAGATGGCATTTAGGCACAAGTGCAATGCCTCTGATTAAACGTCTTCATATCGATAGTTGTGGAAAGCTGAAGGAGATTCCAGAGAGAATGAAAGATGTGAAGCGTATTTGA

>PGSC0003DMG400011525

ATGACTGATGCCTTTGTGTCATTTGCAGTTCAAAAATTGGGTGATTTCCTAATACAGAAAGTTTCCCTGCGTAAAAATCTGAGAAAGGAAGTTGAGTCGCTGAGAAATGAGTTACTCTTCATGCAGTCTTTCCTCAGAGAGGCAGAACAAAAGCAAAGTGATGATCAAAGAGTTCAACAATGGGTGTTTGAGATCAACTCTATTGCTAATGACGCTGTTGCTATACTCGAGACTTACAGCTTCGAGGCTGGTAAAGGTGCTAGTCGTCTCAAGGCTTGTGCTTGCATATGTAGGAAGGAGAAGAAATTCTACAATGTCGTTGAGGAGATCCAATCACTCAAGCAACGAATCATGGATATCTCTCGCAAACGAGACACTTATGGTATTACAAATATCAATAATGCTGGAGAAGGGTCAAGTAATCAGGTTAGAAAATTGAGGAGAACTACCTCATATGTGGATGACCAGGATAACATTTTTGTTGGACTTCAGGATGTTGTACAAAAATTGCTAGCTGAACTTCTCAAAGCAGAGCCACGTAGAAGCGTCATCTCCATTCATGGCATGGGAGGATTAGGCAAGACCACTCTTGCAAGAAACCTCTACAACAGTTCTAATATAGTCTCAAGCTTCCCTACACGCGCTTGGATATGTGTTTCTCAAGAGTACAGCACAATGGATCTTCTTAAGACTATCATAAAATCCATCCAAGGTCGCACAAAGGGAACTCTAGATTTCTTGGAAAGGATGACAGAAAGCGATCTAGAAATTTACCTTCGTGATCTATTGAAAGAAGTCAAATACCTTGTAGTGGTTGATGATGTATGGCAGAGAGAAGCATGGGAGAGTTTGAAAAGAGCATTCCCAGAAAGCAAGAATGGCAGCAGAGTTATTATTACCACGCGGAAACATGATGTTGCGGAAAGAGCTGACAACAGAGGTTTTGTCCATGAACTTCGTTTCCTAAGCCGAGAAGAAAGTTGGGACCTCTTTTGTAGGAAACAACTTGATGTTCGGGCAATGGTTCCAGAAATGGTAAGGATAGCTAGAGATATGGTGGAAAAGTGTAGAGGCTTACCTCTTGCAATTGTTGTATTGAGCGGACTACTTTCACATAAAAGGGGGCTAGACGAATGGCAAAAGGTGAAAGACCACTTTTGGCAGAACATTCAAGATGACTCCATTGAAATCTCCTACATACTATCATTGAGCTACAACGATTTGTCAGCTACGCTCAAGCAGTGTTTTCTGTACTTTGGTATTTTTCCAGAAGATCAAGAGGTCGATGCTGAAAAGATAATACTGTTGTGGATGGCCGAGGGTTTCATACCAAATGGAGAAGAAAGAATGGAGGATGTCGCTGAAGGCTTCTTGAGTGAGCTGATAAGACGAAGCTTGATACAAGAGGTACGTTCATTTTGGGAAAAAGTTACTGTATGTAAGGTTCATGATTTACTTCGTGATCTTGCCGTACAAAAGGCATTTGATATAAAATTCTTTGACATTTATGATCCAAAAAAGCACTCCATATCCTCCTTATGTATCAGACATGTGATTCATGGTCAAGGAGAAAGGTACCTCTCACTTGATCTTTCTTACTTGAAGTTGAGGTCAATTATGTTCTTCGATCCAGATTTTCGTAACATGCATCTTACAAACTTCAGTAGTGTGTTCCGACATATATATGTGTTGTACTTGGATATTGGAGGTTATGTTATGACTGATGTCATAGGAAGTTTGTACCACCTCAAGTTCTTAAGCTTGAGAGGTGTCTGTAATATTCCCTCCTCCATTAGCAACCTCAAGAATTTACGGACACTTCTTGTCGATGACCATGGAGGGTTTAGCCGTCTACCCCAAAAGACAGCTGACCTAATAAATCTAAGACATTTAGTTGCTCCATATTCAGAGCCTCTGAAATGTATAAACAAACTCACGAGTCTTCAAGTTCTTAAAGGCTTTCGTTGTGATCAGTGGAAAGATGTTGACCCTGTTGATTTAGTCAATCTTCGAGAATTAAGCATGGATTATATTAACAGATCTTACTCCCTAAACAACATTAGCAGCTTGAAAAACCTTAGCACTCTCAGATTATTGTGTTATGCTGATGAATCATTCCCATCTCTTAAATTTGTTAATTCTTGTCAAAAGCTCCAGAAATTATGGTTAAGAGGGAAAATAGAGAAACTTCCTCTGTTTCCAGATTCCATCACAATGATGGTTCTTTGGAAGTCAAAACTCATGGAAGATCCGATGCCTATTTTGGGAATGTTACCAAACCTAAGGAATCTCGAATTAGAAGAAGCTTATGAAGGAAAAGAAATAACGTGCAGTGATAACAACTTCAGTCGACTCGAGTTCCTTCGTCTTCATCGTTTTGACAAGCTAGAAACATGGCATTTATCTACAAGTGCCATGCCTTCAATTAAAGGTCTTGACATCAAATACTGTCCACATCTATCTCATATTCCCAAGAGAATGCAAGACGTGGACAGAACTCCTTTTTGGCTTGTTTCATAA

>PGSC0003DMG400011524

ATGGATCTTCTTAAAACTATCATAAAATCCATCCAAGGTTGCGCCAAGGAAACTCTAGATCTGTTGGAAAAGATGGCAGAAAGAGATCTAGAAAATCACCTTCGTAAGCTATTAACAGAACACAAATACCTTGTGGTGATTGATGATGTGTGGCAGAGAGAAGCATGGGAAAGTTTGAAACGAGCATTTCCAGATAGAAAGAATGGCAGCAGAGTTATTATTACCACGCGCAAAGAGGATGTAGCTGAAAGAGCAGATAACAAAGGTTTTGTCTATAGACTTCGTTTCCTGAGCCAAGAAGAAAGTTGGGATCTCTTTTGTAGGAAACTACTCGATGTTCGGGCAATGGTCTCAGCGATAGAAAGGCTAGCTAAGGATATGGTGGACAAGTGTGGAGGTTTACCTCTTGCAATTGTTGTTTTAAGCGGACTACCTTCACATAAAAGAGGGCTAGAAGAATGGCAAAAAGTGAAGGACAACCTTTGGCAGAACATTAAAGACGACTCTATTGAAGTCTCCTACATACTATCATTGGGCTACAACGATTTGTCAACTGCACTCAAGCAGTTTTTCCTGTACTTTGGTATATTTCCAGAAGATCGTGTGGTCCATGTTGATCACATATTATGGTTGTGGATGGCTGAAGGATTCGTACCAACAGGTAAAGAAATAATGGAAGATGTTGCTGAAGGCTTCTTGAATGAGCTGATAAGACGAAGCTTGATACAAGTGGTAAGAACATTTTGGGAAAAAGTCAGTAAATGTAGGATTCATGATCTACTTCGCGATCTTGCTGTACAAAAAGCATTGGAGGTAAACTTTTTGGACATTTATGATCCAAGAAAGCACTCCATATCCTCCTTTTGTCTGAGACATTCCATTCATAGTCAAGGAAAACGGTATCTCTCACTTGATCTTTCTAACTTTAAATTGAGGTCACTTATGTTCCTTGATCCAGACTTTTAA

>PGSC0003DMG400011523

ATGGCTGATGCCTTTGTGACATTTGCAGTTAAAAAATTGGGTGATTTCCTCATTCAGGAAGTTTCCCTGCTTACAAATCTGAGAGATGAAGTTAGATGGCTGAGAAATGAGCTACTCTTCATGCAGTCTTTCCTCAGAGATGCAGAACTAAAGCAAAGTAGAGATCAAAGAGTTCAGCAATGGGTGTTTGAGATCAACTCTATTGCAAATGACGCTGTCGCTATCCTAGAGACTTACAGCTTCAAGGCTGGTAAAGGTGGCGATGGATTTGCTAGTTGTCTCAAGGCTTGCGCTTGCATCTGTAAGGAGGAGACCAAATTCTACAAAGTCTCCAAGGAGATCCAATCACTCAAGCAACGAATTATGGATATCTCTCGCAAGCGAGAGACTTATGGTATTACAAATATCAATAATAATGCAGGAGATGGACCAAGTAATCGGCCAAACAATCAGTCTGCCATTGTTACAACATTGAGGAGAACTACCTCCTATGTGGATGGTCAGGATCACATTTTTGTTGGATATCAGGATGTTGTAGAAAGATTGCTATCTGAACTTCTCAAAGCAGAGCCTTGTCGAAGCGTCATCTCCATTTATGGCATGGGTGGTTTAGGCAAGACCACTCTTGCAAGAAATCTGTACATCAGTCCTGATATAGTCAATAGCTTCCATACACGAGCTTGGATATGTGTTTCTCAAGAGTACAACACCGTGGATCTCCTCAGGTATATCATAAAATCCATCCAAGGTTGCACCAAGGAAACTCTAGATTTGTTGGAAAGGATGACAGAAAGAGATCTAGAAATTTACCTTCGTGATCTTTTAAAAAAACGCAAATACCTTGTGGTGGTTGATGATCTATGGCAGAGAGAAGCATGGGAGAGTTTGAAAAGAGCATTCCCGGATAGCAAGAATGGCAGCAGAGTTATTATCACCACGCGCAAAGAGGATGTTGCTGAAAGAGCAGACAGCAGAGGTTTTGTCCATAAACTTCGTTTCCTAAGCCAAGAAGAAAGTTGGGATCTCTTTTGTAGGAAACTACTTGATGTTCAAGCAATGACCTCCACAATGGAGAGGCTAGCTAAGGATATGGTGGACAAGTGTGGAGGCTTACCTCTTGCAATTGTTGTATTGAGTGGACTACTTTCGCATAAAAGGGGGCTAGGCGAATGGCAAAAGGTGAAAGACCGCCTTTGGAAGAACATTGAAGATGACTCTATTGAAATCTTCTACGTACTATCATTAAGCTACAATGATTTGTCAACTGCGCTCAAGCAGTGTTTTCTGTACTTTGGTATTTTTCCAGAAGATCAAGTGCTCGAGGCTGAAACCATAATACGATTGTGGATGGCCGAGGGTTTCATACCAAGAGGAGAAGAAAGAATGGAGGATGTCGCTGAAGGCTTCTTGAATGAGCTAATAAGACGAAGCTTGGTTCAAGTGGCTCGAACATTTTGGGAAAAAGTTACTGAATGTAAGGTTCATGATTTACTTCGTGATCTTGCCATACAAAAGGCATTGGAGGTAAACTTCTTTGACATTTATGATCCAAGAAACCACTCCATATCCTCCTTATGTATCAGACATGCCATTCATGATAAAGGAGAAAAGTACCTCTCACTTGATCTTTCTAACTTAAAGTTGAGGTCAATTATGTTCTTCGATCCAGATTTTCGGAACATGAATCTTATAAACTTCAGTAGTGTGTTCCAACATATATATGTGTTGTACTTGGATACTCTTGGTGGCACTGTACCTTATGCCATAGGAAGTTTGTACCGCCTCAAGTTCTTAAGCTTGAGAGGTATTGGTAATCTTCCCTCCTCCATTGGCAACCTCAAGAATTTACAGACACTTTGTGTCAAAAGTGAATTTAGACATTTGTGCCAACTACCCCCTGAGACAGCTGACCTAATAAATCTAAGGCATTTAGTTGCTCCGTATTCTGAACCTCTGGTACGTATAAGCAAACTCACTAGTCTTCAAATTCTTAAAGACATCTCTTGTGATCAGTGGAAAGATGTTGACCCTGTTGATTTAGTCAATCTCCTAGAATTAAGCATGATTGATATTACCAAATCTTACTCCCTAAACAACATTAGCAGATTGAAAAACCTTAGCACTCTCAAATTGTTTTGTAGAGGTGGTCAATCATTCCCAGACCTTGAATTTCTTATTATTGTGAAAAGCTCCAGAAATTGTGGTAGAATAGAGAAACTGCCTCTGTTACCAAATTCCATCACAATGATGGTTCTTTTGGACTCAAAACTCATGGAAGATCCGATGCCTATTTTGGGAATGTTGCCAAACCTAAGAAACTCGAATTTCTTAGAGCTTATCAAGGAAAGAGATATTCTGCAGAGCTTATCAAGGAAAGAGATATTCTGCAGTGATAATAGCTTCCGTCAACTCGAGTTCCTTAGTCTTGCTTGTCTTGAGAACCTAGACACATGGCATTTAGCCACAAGTGCCATGCTTCTGATTAAAGGTCTTCGTATTGATCACTGTAAAAAGCTGAACAAGATTCCCCAGAGAATGAAGGACGTGAAGCTTTTCAAGCATACATGA

>PGSC0003DMG401011522

ATGGCCGATGCCTTTCTGTCATATGCAGTTCAAAAATTGGGTGATTTCCTAATACAGAAAGTTTCCCTGCTTACAACTCTCATAGATGAAGTTAGATTGCTGAGAAATGAGCTACTCTTCATACAGTCTTTCCTCAAAGATGCAGAACTAAAGCAATGTGGAGATCAAAGAATTCAACAATGGGTGTTTGAGATCAACTCTATTGCTAATGACGCTGTTGCTATACTCGAGACTTATAGCTTCGAGGTTGCTAACGGTAATGATGATGGATTTTCTAGTCGTCTCAAGGCTTGCGCTTGCATCTGTAGGAAGGAGACCAAACTCTACAAAGTTGCCAAGGAGATTGAATCACTCAAGCAACGAATCATGGATATCTCTCGCAAACGAGATACTTATGGTATTAGAGATATCAATAATGCAGGAGAAGGTTCAAGTAATCAGTCTGCCATGGTTAGATCATTGAGGAGAACTACCTCCTATGTGGATGATCAGGATCACATTTTTGTTGGATTTCAGGATGTTGTAGAATCATTGTTAGCTGAACTTCTCAAAGCAGAGCCTCGTCGAAATGTCATCTCCATTTACGGTATGGGCGGTTTAGGCAAGACCATACTTGCGAGAAACCTCTACACAAATCCTATCATAGTCTCTAGCTTCCCAACATGCGCTTGGATATGTGTTTCTCAAGAGTATAACACCATGGATCTCCTTAGGAATATCATAAAATCTATCAAAGGTTGCACCAAGGAAACTCTAGATTTGTTGGAGAGGATGACAGAAAGAGATCTAGAAATTTACCTTCGTGATTTATGAAAAGAACGCAAATACCTTGTTGTCGTTGATGATGTATGGCAGAGAGAAGCATGGGAAAGTTTGAAAAGAGCATTCCTGGATAGCAAGAATGGAAGCAGAGTGATTATTACCACTCGCAAAGAGGATATTGCTGAAAGAGCAGACAACAGAGGTTTTGTCCATAGACTTCGTTTCCTGAGCCAAGAAGAAAGTTGGGACCTCTTTTGTAGGAAACTAGTTGATGTTCGGGCAATGGTTCCAGTGATGGAAAGGTTAGCTAGAGATATGGTGGACAAGTGTGGAGGCTTACCTCTTGCAATTGTTGTATTGAGTGGACTACTTTTGCACAAAAGGGGTCTAGATGAATGGCAAAAGGTGAAAGACCACCTTTGGAAGAACATTAAAGATGACTCTATTGAAATCTCCTACATACTATCATTAAGCTACAACGATTTGTCAACTGCACTAAAGCAGTGTTTTCTGTACTTTGGTATTTTTCCAGAAGATCAAGAGCTCGAGGCTGAAAACATAATACGGTTGTGGATGGCCGAGGGTTTCATACCAAGAGGAGAAGAAAGAATGGAGGATGTCGCTGAAGACTTCTTAAATGAGCTGATAAGACGAAGCTTGGTTCAAGTGGCTGGAACATTTTGGGAAAAAGTTATTCTATGTAGGGTTCACGATGTAGTTCGTGATCTTTCCATACAAAAGGCATCGGAGGTAAACTTCTTTGATATTTATGATCCAAGAAACCACTCCATATCCTTCTTATGTATCAGACATGCCATTCATGATCAGGGAGAAAAGTACCTCTCACTTGATCTTTCTAACTTAAAGTTGAGGTCAGTTATGTTCTTCGATCCAGATTTTCTTAACATGAGTCTTATAAACTTCAGTAGTGTGTTCCAACATATGTATGTGTTATACTTGGATACTTTTGGTGGAACTATACCTGATGCCATAGGAAGTTTATACCACCTCAAGTACTTAAGATTGACAGGTATCCATTGTCTTCCCTCCTCCATTTGCAACCTCAAGAATTTACAGACACTTCGTGTCGTAAACGAAAACGGGCGCTTATGCCAACTACCCAGAGAGACAGCTGACCTGATAGATCTAAGATATTTAGATGCTCCTTATTCAAAACCTCTGAAATGTATAAACAAACTCAGTAGTCTTCAAGTTCTTAAAGGCCTTGCTTGTGATCAGTGGAAAAATGTTGACCCTGTTGATTTAGTTAATCTTCGAGAATTAAGCATGCATGATATTACCAAATCCTACTCCCTAAACAACATTAGCAGCTTGAATAACCTTAGCACTGTCACATTGTGTTGTGAAGATGATGAATCATTCCCAGCCCTTGAATTTCTTACTTCTTGTCAAAAGCTCCAGAAATTGTGGTTAGAAGGGGAAATAGAGAAACTGCCTCTATCAGACGCATTTCCAAATTCCATCACAATGATGGTTCTTGTGAAATCAAAACTCATGAAAGATCCGATGCCTATTTTGGGGATGTTGCCAAACCTCAGGAATCTCGATTTATTAAAAGCTTATGAAGGAAATGAATTAACTTGCAGTGATAACAGTTTCAGTCAACTAGAGTTCCTTCGTCTTGATGATCTTGAGAAGCTAGAAAGATGGCATTTAGGCACAAGTGCCATGCCTCTCATTAAAGGTCTTGCTATCTATCATTGTCCAAAGCTAAAGGAGATTCCCGAGAGAATGAAAGACGTGGAGAGAACTCCTTATCGATGA

>PGSC0003DMG400011521

ATGGCTGATGCCTTTGTGTCATTTGCACTTCAAAAATTAGGTGATTTCCTCATACACGAAGTTTCCCTGCTTACAAGTCTCAGAGATGAAATTACATGGCTGAGAAATGAGCTACTCTTCATGCAGTCATTCCTCAAAGATGCAGAACAAAAGCAAAGTAGAGATCACAGAGTTCAACAATGGGTGTTTGAGATCAACTCTATTGCTAATGACGCTGTCTCTATACTCGAGACTTACAGCTTCGAGACTGGTAAAGGTGATGATGGATTTGCTAGTTGTCTCAAGGCTTGCGCTTGCATCTGTAGGAAGGAGAAGAAATTCAATCACTCAAGCAACGAATCATGGATATCTCTCGCAAACGAGAGACTTATGGTATTACAAATATCAATAGTAATGCAGGAGAAGGGCCAAGTAATCAGATTAGAACATTGAGGAGAACTACCTCATATGTGGATGACCAGGATTACATTTTTGTTGGCTTTCAGGATGTTGTACAAACATTGCTAGCTGAACTTCTCAAAGCAGAGCCTCGTCGAAGCGTCCTCTCCATTTTATGGAATGGGGGGTTTAGGCAAGACCACTCTTGCGAGAAAACTTTACATCAGTCCTAATATAGCCTTTAGTTTCCCTACACGCGCTTGGATATGTGTTTCTCAAGAGTACAACACAATGGATCTTCTTAGGAATATCATAAAATCCATCCAAGGTCGCACCAAGGAAACTCTAGATTTGTTGGAAAGGATGACAGAAGGAGATCTAGAAATTTATCTTTGTGATCTATTAAAAGAACGCAAATACCTTGTGGTGGTTGATGATGTATGGCAGAAAGAAGCATGGGAGAGTTTGAAAAGAGCATTCCCAGATAGCAAGAATGGCAGCAGAGTCATTAATACCACGCGCAAAGAGGATGTCGCTGAAAGAGCAGACGACAGAGGTTTTGTTCATAAACTTTGTTTCCTATGCCAAGAAGAAAGTTGGGACCTCTTTTGTAGGAAACTAGTTGATGTTCGAGCAATGATTCCAGCAATGGAAAGTCTAGCTAAGGATATGGTGGAAAAGTGTAGAGGCTTACCTCTTGCAATTGTTGTATTGAGCGGACTACTTTCGCATAAAAAAGGGGCTAAACGAATGGCAAAAGGTGAAAGACTGCCTTTGGAAGAACATTATGAAGATAAATCTATTGAAATCTCCAGCATACTATCATTAAGCTTCAACGATTTGTCAGCTGCGCTCAAGCAGTGTTTTCTGTACATTGGTATTTTTCCAGAAGATCAAGTGATCGATGCTGAAGACATCATACCCAGAGGAGAAGAAAGAATGGAGGATGTCGCTGAAGACTTCTTAAATGTGCTGATAAGACGAAGCTTGGTTCAAGTGGAAAAAATATTTTTGGAAAAAGTTACTAAGTGTAGGGTTCATGATTTACTCCGTGATCTTGCCATACAAAAGGCATCCGACACAAACCTCTTTGACATTTATCATCCAAGAAAGCACTCCATATCCTCTTCATGTATCAGACTTGCCATTCATAGTCAAGGAGAAAAGTATCACTCACTTGATCTTTCTAATTTGAAGTTGAGGTCAATTATGTTCTTCGATCCAGTTTTTCCTAATGTGTTCCAACATATATATGTGTTGTACTTGCATATGGATTATGGTTGTGTTATACCTGAATCCATAGGGAGTTTGTACCACCTCAAGTTGTTAAGCATTAGATCAGGTGTCTGTAATATTCCCTCCTCCATTGGCAACCTCAAGAATTTACAGACACTTGTCGTTGTAAATGGGTACGCATCATTTTGCCAACTACCCCGCAAGATAGCTGACCTAATAAATCTAAGACATTTTGTTATTCCATATTCAGAGCCTCTGAAATGTATAAACAAACTCAGGAGTCTTCAAGTTCTTAAAGGCATTCGTTGTGATCAGTGGAAAGATGTTGACCCTGTTGATTTAGTCAATCTTCGAGAATTAAGCATGGATCGTATCAGGAGCTCTTACTCCCTAAACAACATTAGCAGCTTGAAAAACCTTAGCACTCTCAGATTGTCTTGTCAAGATTATGATGAATCATTCCCATCCCTTGAATTTGTTAATCGTTGTGAAAAGCTCCAGAAATTGTGGTTAAAAGGGGAAATAGAGAAACTGCCTAATCTGTTTCCAAAGTCCATCACAATGATGGTTTTGAGGAACTCGGGACTGACAAAAGATCCGATGCCTATTTTGGGAATGTTGCCAAACCTAAGGAATCTCAAACTAGAAGATGCTTATGAAGGAAAAGAAATAATGTACAGCGATAACAGTTTTAGTCAACTCAAGTTCCTTAATCTTGATCATCTTCAAAACCTAGAAAGATGGGATTTAGGCACAAGTGCAATGCCACTCATTAAAGCTCTTCATATCTATGAATGTCCAAACCTGAAGGAGATACCAGAGAGAATGAAAGACTTGAAGAAGCTTGAAGAGACCAAGTTTTTTTGTACAAATAGTGTGTTAATATTTGGGTTAAACAGGAGAAGAAGTTCGCATGAATCAAGTTGGATTATTTCAACATTCATTTTTTTTTGTTTGAATAAAATCAATATTATTTTA

>PGSC0003DMG400011520

ATGGCTGATGCCTTTGTGTCATTTGCAGTTCAAAAATTGAGTGATTTCCTAATACAACAAGTTTCTCTGAGAACAAATCAGAGAGATGAAATTAGATGGCTGAGAACTGAGCTACTTATCATACAGGCATTCCTCAAAGATGCAGAACAAAAGAAAAGTGGAGATCATAGAATTCAACAATGGGTGTTTGAGATCAACTCTATTGCTAATGACGCTGTTGCTATACTCGACACTTACAGTTTCGAGGCTGGTAAACGTGCTAGTCGTCTCAAGGCTTGCGCTTGCATATGTAGGAAAGAGAAGAAATTCTACAATGTCGCCAAGGAGATTCAATCACTCAAGCAACGAATCATGGATATCTCTCGCAAACGAGATACTTATGGTATTACAAATATCAATAGTAATGCTGGAGAAGGGTCAAGTAATCAGGTTACAACATTGAGGAGAACTACCTCATATATAGATGATGATGACATTTTTGTTGGCTTTCAGGATGTTGTACAAACATTGCTAGCTGAACTTCTCAAAGTAGAGCCTCGTAGAAGCATCGTCTCCATTCATGGAATGGGCGGATTAGGCAAGACCACTCTTGCAAGAATCCTCTACAACAGTCCTAATATAGTCTCAAGCTTCCCTACACGTGCTTGGATATGTGTTTCTCAAGAGTACAACACAATGGATCTTCTTAAGACTATCATAAAATCCATCCAAGGTTGCACCAAGGAAACTCTAGATTTGTTGGAAAAGATGGCAGAAACAGATCTAGAAAATCACCTTCGTGATCTATTGAAAGGACGCAAATACCTTGTGGTGGTTGATGATGTGTGGAAGAGAGAAGCATGGAAGAGTTTGAAAAGAGTATTCCCGGACAACAAGAATGGCAGCAGAGTTATTATCACCACACGCCAAGGGGATGTCGCTGAAAGAGCAGACGATAAAGGTTTTGTCCATAAACTTCGTTTCCTAAGCCAAGAAGAAAGTTGGGACCTCTTTTGTAGGAAACTACTTGATGTTCGAGCTATGGTTCCAGCAATGGAAAGTCTAGCTAAGGATATGGTGGGAAAATGTAGAGGCTTACCTCTTGCAATTGTTGTATTGAGTGGACTACTTTCGCATAGATGGGGGCTAGACAAATGGCAAAACGTGAAAGACTGCCTTTGGAAGGACATTGAAGAAGACTCTATTGAAATCTCCTACATACTATCATTAAGCTTCAGCGATTTGTCAGCTGCGCTCAAGCTGTGTTTTCTCTACTTTGGTATTTTTCCAGAAGATCAAGTGATCAAAACTGACAACATAATGCGGTTGTGGATGGCAGAGGGTTTCATCATACCAAGAGGAGAAGAAAGAATAGAGGATGTCGCTGAAGGTTTCTTGAATGAGCTGATAAGACGAAGCTTGGTTCAAGTAGTGGATACATTTTGGGAAAAAGTTACTGAATGTAGGGTTCATGATTTACTTCGTGATCTTGCCATACAAAAGGCATTGGAGGTAAACTTCTTTGACATTTATGATCCAAGAAAGAACTTGAAATCCACTTCATGTATAAGACATGCCATTCATAGTGAAGGAGAAAGGTACCTCTCATCACTTGATCTTTCTAACTCGAAGTTGAGGTCAATTATGTTCTTTGATCCACATTTTCGTAATCTGTTCCAACATATACATGTGTTCCAACATATATATGTGTTGTACTTGGATATTAATTATGGTAATGTTATACCTGATGCCATAGGGAGTTTGTACCACCTCAAGTTGTTAAGCTTGAGAGGTGTCCGTAATATTCCCTCCTCCATTGGCAACCTCAAGAATTTACAGACACTTGTTGTTGTCAATATTGACTTACTTTGCCAACTACCCCCCGAAACAGCTGACCTAATAAATTTAAGACATTTAGTTGTTCCATATTCTAAACCTCTGGCAGGTATAAGCAAACTCACTAGTCTTCAAGTTCTTCACCTTGATTGTGATCAGTGGAAAGATGTTAACCCAGTTGATTTTGTCAATCTTCGAGAATTAGAGATGGGTAATATTAGGAAATCTTACTCCCTAAACAACATTAGCAGCTTGAAAAACCTCAACACTCTCAGATTGTGTATTTTGTTTGGTCAACAATATACATATCCATTCCCATCCCTTGAATTTGTTTATAGTTGTGAAAAGCTCCAGAAATTGTCGTTAGGTGGGAGATTAGAGAAACTGCCTGTGTTTCCAGATTCCATCACAATGATAGTTATTTGGCAGTCAAGACTGACAAAAGATCCGATGCCTATTTTGGGAATGTTGCCAAACCTAAGGAATCTCCACTTGTCCAGAGCTTATGAAGGAGAAGAAATAATGTGCAGTGATTACAGCTTCAGTCAACTAGAGCTCCTTCATCTTTATGGTCTTGACAACTTAGAAAGATGGCATTTAGGCACAAATGCCATGCCTCTCATTAAAGATCTTTCTATCTATAATTGTCAAAAGCTGAAGGAGATTCCAGAGAGAATGAAACACGTGAAGCATTTCAAACATATACGAAGTCTTATATGA

>PGSC0003DMG400011517

ATGGCTGATGCCGTTGTGTCATTTGCAGTTCAAAAATTGGGTGATTTCCTCATTCAGGAAGTTAACCTACGTTTAAGTCTAAGAGAGGATATGAAGTGGATGAGAACTGAGCTTCTCTTCATGCAGTCTTTCCTCAAAGATACAGAACTAAAACAATATGGAGATCATACAATTCAACAATGGGTAACTGAGATGAACTCTATTGCTAATGACGCTGTTGCTATACTCGAGACTTACAGTTTAGAGGCTGGTAAAGGTGCTAGTCGTCTCAAGGCGTGCGCTTGCATATGTAGGAAGGAGAAGAAATTCTACAATGTCGCCAAGGAGATTCAATCACTCAAGCAACGAATCATGGATATCTCTCGTAAAAGAGATACTTATAGTATTACAAATATCAATAGTAATGCTGGAGAAGGAACAAGTAATCAGGTTACAACACTGAGGAGAACTACCTCATATGTGGATGACCAGGATTACATTTTTGTTGGACTTCAGGATGTTGTAGAATCATTGCTAGCTGAACTTCTCAAAGCAGAGCCTCGTCGAAACGTCCTCTCCATTCATGGGATGGGTGGATTGGGCAAGACCACTCTTGCCAGAAACCTCTACAACAGTCCTAATATAGTCTCAAGCTTCCCTACTCGCACTTGGATATGTGTTTCTCAAGAGTACAACACAATGGATCTTTTTAAGACTATCATAAAATCCATCCAAGGTTGCGCCAAGGAAACTCTAGATTTGTTGGAAAAGATGGCAGAAACAGATCTAGAAAATCACCTTCGTGATCTATTAGAAGGATGCAAATACCTTGTGGTGGTTGATGATGTGTGGCAGAAAGAAGCATGGGAGAGTTTGAAAAGAGCATTTCCGGATAGAAATAATGGCAGCAGAGTTATTATCACCACGCGCAACCAGGATGTCGCTGAAAGAGCAAACAACAGAGGTTTTGTCCATAAACTTCGTTTCCTAAAACAAGAAGAAAGTTGGGATCTCTTTTGTAGGAAACTACTTGATGTTCGAGCAATGGTTCCAGCAATGGAAAGTCTAGCTAAGGATATGGTGGAAAAATGTAGAGGCTTACCTCTTGCAATTGTTGTATTGAGTGGACTACTTTCGCATAGATGGGGGCTAGACAAATGGCAAAACGTGAAAGACTGCCTTTGGAAGGACATTGAAGAAGACTCTATTGAAATCTCCTACATACTATCATTAAGCTTCAACGATTTGTCAGCTGCGCTCAAGAAGTGTTTTCTCTACTTTGGTGTTTTTCCAGAAGATCAAGTGATCGATGCTGACGACATAATGCGGTTGTGGATGACAGAGGGTTTCATCATACCAAGAGGAGAAGAAAGAATGGAGGATGTCGCTGAAGGCTTCTTGAATGAGCTGATAAGACGAAGCTTGGTTCAAGTAGCTCAAACATTTTGGGAAAAAGTTACTGTATGTAGGGTTCATGATTTACTTCGTGATCTTGCCATACAAAAGGCATTGGAGGTAAACTTCTTTGACATTTATGATCCAAGAAAGAACTTGAAATCCACTTCATGTATAAGACATGCCATTCATAGTGAAGGAGAAAGGTACCTCTCATCACTTGATCTTTCTAACTCGAAGTTGAGGTCAATTATGTTCTTTGATCCACATATTTGTAATGTGTTCCAACATATAGATGTGTTCCGACATATATATGTGTTGTACTTGGATATTGATTATGGTAAAGTTATACCTGATGCCATAGGGAGTTTGTACAACCTCAAGTTGTTACGCTTGAGTGTTGTCAGTAAAATTCCCTCCTCCATCGGCAACCTCAAGAATCTACAGACACTTGTTGTTGTCAATATGGACAGACGCTTTTGCCAACTACCCCCCGAAACAGCTGACCTAATAAATTTAAGACATTTAGTTGTTCCATATTCTATACCTCTGGCAGGTATAAGCAAACTCACTAGTCTTCAAGTTCTTCGCCTTGATTGTGATCAGTGGAAAGAAGTTGACCCAGTTGATTTAGTCAATCTTCGAGAATTAGAGATGCGTAATATATACAAATCTTACTCCCTAAACAACATTAGCAGCTTGAAAAACCTTAACACTCTCAAATTGTGTATTTTGCTTGGTCCACAATCTACATATCCATTCCCATCCCTTGAATTTGTTAATAGTTGTGAAAAGCTCCAGAAATTGTTGTTAGGTGGGAGATTAGAGAAACTGCCTGTGTTTCCAGATTCCATCACAATGATATTTCTTTGGGAGTCAAGACTGACAAAAGATCCGATGCCTATTTTGGGAATGTTACCAAACCTAAGGAATCTCGAATTAGAAGCAGCTTATGAAGGAGAAGAAATAATGTGCAGTGATTACAGCTTCAGTCAACTAGAGCTCCTTCATCTTTATGGTCTTGACAACTTAGAAAGATGGCATTTAGGCACAAATGCCATGCCTCTCATTAAAGATCTTGTTATCCATCATTGTCCAAAGCTGAGGGAGATTCCAGAGAGAATGAAAGACGTGAAGCATTTCAAACGTATATGA

>PGSC0003DMG400032584

ATGGCTGATGCCTTTCTGTCATTTGCAGTTCAGAAATTGGGTGATTTCCTCATTCAGGAAGTTAACCTACGTTTAAGTCTAAGAGAGGATATACAGTGGCTGCTCTTCATACAGTCTTTCCTCAGAGATGCAGAACTAAAGCAAAGTAGAGATCAAAGAGTTCAACAATGGGTGTTTGAGATCAACTCTATTGCTAATGATGCTGTTGCTATGCTCGAGACTTATAGATTCGAGGCTGATAAAGGTGCTAGTCGTCTCAAGGCTTGCGCTTGCATTTGTAGGAAGGAGAAGAAACTCTACAATGTCGCCAAGGAGATTCAATCACTCAAGCAATGAATCATGGATATCTCTCGCAAACGAGAGACTTATGGTGTTACAAATATCAATAGTAATGCAGGAGAAGGGCCAAGTAATCAGATTATAACATTGAGGAGAACTACCTCATATGTGGATGACCAGGATTACATTTTTGTTGGCTTTCAGGATGTTGTACAAACATTGCTAGCTCAACTTATCAAAGCAGAGCCTCGTCGAACCGTCCTCTCCATTTATGGCATGGGCGGATTAGGCAAGACCACTCTTGCAAGAAACCTCTACACCAGTCCTAATATAATCTCAAGCTTCCCTACACGCGCTTGGATATGTGTTTCTCAAGAGTACAACACAATGGATCTTCTTAGGAATATCATAAAATCCATCCAAGGTCGCACCAAGGAAACTCTAGATTTGTTGGAAAGGATGACAGAAGGAGATCTAGAAATTTATCTTCGTGATCTACTAAAAGAACGCAAATACCTTGTGGTGGTTGATGATGTATGGCAGAAAGAAGCATGGGAGAGTTTGAAAAGAGCATTCCCGGATAGCAAGAATGGCAGCAGAGTCATTATTACCACGCGCAAAGAGGAAGTCGCTGAAAGAGCAGACAACATAAGTTTTGTTCATAAACTTCGTTTCCTAAGTCAAGAAGAAAGTTGGGATCTCTTTTGTAGGAAACTACTTGATGTTCGAGCAATGATTCCAGCAATGGAAAGTCTAGCTAAGGATATGGTGGAAAAGTGTAGAGGCTTACCTCTTGCAATTGTTGTATTGAGCGGACTACTTTCGCATAAAAAGGGGCTAAACGAATGGCAAAAGGTGAAAGACTGCCTTTGGAAGAACATTATTGAAGATAAATCTATTGAAATCTTCAGCATACTATCATTAAGCTTCAATGATTTGTCAGTTGCGCTCAAGCAGTGTTTTCTGTACATTGGTATTTTTCCAGAAGATCAAGTGATCGATGCTGAAGACATAATACGTTTGTGGATGGCAGAGGGTTTCATCATACCCAGAGGAGAAGAAAGAATGGAGGATGTCGCTGAAGACTTCTTGAATGAGCTGATAAGACGAAGCTTGGTTCAAGTGGCAAAAATATTTTTGGAAAAAGTGAGGGTTCATGATTTACTCCGTGATCTTGCCATACAAAAGGCATCCGACACAAACCTCTTTGACATTTATCATCCAAGAAGGCACTCCAAATCCTCTTCATGTATCAGACTTTCCATTCATAGTCAAGGAGAAAGGTATCACTCACTTGATCTTTCTACTTTGAAGTTGAGGTCAATTATGTTCTTCGATCCAGTTTTTCCTAATGTGTCCCAACATATATATTTGTTTCGACATATATATGTGTTGTACTTGCATATGGATTATGGTGGTGTTCTACCTGATGCCATAGGGAGTTTGTACCACCTCAAGTTGTTAAGCATTAGATCAGGTGTCTGTAATATTCCCTCCTCCATTGGCAACCTCAAGAATTTACAGACACTTGTCATTGTAAATGGGTACGCATCATTTTGCCGACTATCCCGCAAGATAGCTGACCTAATAAATCTAAGACATTTAGTTGTTCAATATTCAGAGCCTCTGAAATGTATAAACAAACTCACGAGTCTTCAAGTTCTTAAAGGCATTCGTTGTGATCAGTGGAAAGATATTGACCATGTTGATTTAGTCAATCTTCGAGAATTAAGCATGGATCGTATCAGGAGCTCTTACTCCCTAAACAACATTAGCAGCTTGAAAAACCTTAGCACTCTCAGATTGTCTTGTCAAGATTATGATGAATCATTCCCATCCCTTGAATTTGTTAATTGTTGTGAAAAGCTCCAGAAATTGTGGTTAAAAAGGGAGAATAGAGAAACTGCCCTGTTTCCAAAGTCCATCACAATGATGGTTTTGAGGAACTCGGGACTGACAGAAGATTCGATGCCTATTTTGGGAATGTTGCCAAACCTAAGGAATCTCAAACTAGAAGATGTTTATGAAGGAAAAGAAATAATGTGCAGCGATAACAGTTTTAGTCAACTCAAGTTCCTTAATCTTGATCATCTTCAAAACCTAGAAAGATGGGATTCAGGCACAAGTGCAATGCCTCTCATTAAAGCTCTTCATATCTATGAATGTCCAAACCTGAAGGAGATACCAGAGAGGATGATTGACTTGAAGAAGCTTGAAGAGACCAAGTTTTTTTTGTACAAATAG

>PGSC0003DMG400032582

ATGGCTGATGCCTTTGTGTCATTTGCAGTTCAAAAATTGGGTGATTTCCTCATACAGCAAGTTTCCCTGCGTAAAAATCTGAGAGATGAAGTTAGATGGCTGAGAAATGAGCTACTCTTCATGCAGTCTTTCCTCAGAGATGCAGAACTAAAGCAATATGGAGATCAAAGAGTTCAACAATGGGTGTTTGAGATCAACTCTATTGCTAATGATGCTGTTGCTATACTCGAGACTTACAGCTTCGAGGCTGGTAAAGGTGATGACGATGGATTTGCTAGTCGTCTCAAGGCTTGCGCTTACATCTGTAGGAAGGAGAAGAAATTCTACAATGTCGCCAAGGAGATTCAATCACTCAGGCAACGAATCATGGATTATAACAGTCCTAATATAGTCAATAGCTTCCCTACACGCGCTTGGATATGTGTTTCTCAAGAGTACAACACAATGGATCTCCTTAGGAATATCATAAAATCCATCCAAGGTTGCACTAAGGAAACTCTAGATTCGTTGGAAAGGATGACAGAAAGAGATCTAGAAATTTACCTTTGTGATCTATTAAAAGAGCACAAATACCTTGTGGTGGTTGATGATGTATGGCAGAGAGAAGCATGGGAGAGTTTGAAAAGAGCATTCCCGGATGGCAAGAATGGCAGCAGAGTCATTATTACCACGCGCAAAGAGGATGTCGCTGAAAGAGCAGACAACAGAGGTTTTGTCCATAAACTTCGTTTCCTAAGCCAAGAAGAAAATTGGGATCTCTTTAGTAGGAAACTACTTGATGTTCGAACAATGGCTCCAGAAATGGAAAGTCTAGCTAAGGATATGGTGGAAAAGTGTAGAGGCTTACCTCTTGCAATTGTTGTATTGAGCGGACTACTTTCGCATAAAAAGGGGCTAAACGAATGGCAAAAGGTGAAAGATCACCTTTGGAAGAACATTAAAGAAGATAAATCTATTGAAATCTCCAAGATACTATCCTTAAGCTACAATGATTTGTCAACTGCACTCAAGCAGTGTTTTCTCTACTTTGGTATTTTTCCACAAGATCAAGTGCTCGGGGCTGATAGCATAATACGGTTGTGGATGGTCGAGGGTTTCACACCCATAGGAGAAGAAAGAATGGACGATGTCGCTGAAGGCTTCTTGAATGAACTTATAAGACGAAGCTTGGTTCAAGTGGCAAAAACATATTGGGAAAGATTTACTGAATGTAGGGTTCATGATTTACTCCATGATCTTGCGATACAAAAGGCATTGGAAGTAACTTCTTTGACATTTATGATCCAAGAAGCCACTCCATATCCTCTTTATGTATCAGACATGATTCATAGTCAAGGAGAAAGGTACCTCTCACTTGATCTTTCTAACTTAAAGTTGAGGTCAATTATGTTCTTCAATCCAAATTTTCGTAAGATGAGTCTTATAAACTTCAGGAGTGTGTTCCAGCATCTATATGTGTTGTACTTGCATATGCATGGTGGGAATATGTCTATAGTACCTGATGCCATAGGAAGTTTGTACCACCTCAAGTTCTTAAGATTGAGAGGCATCCAAGGTCTTCCCTCTTCCATTGGCAACCTCAAGAATTTACAGACACTCTATGTCAATGACGGCATACAGTATTGTGAACTACCCCATGAGACAGCTGAGCTAATAATTCTAAGACATTTTGTTGCCCTGTATTCAAAACCTCTGGAACGTATAAGCAAACTCACTAGTCTTCAAGTTCTTCAAAACATCTGTTGTGATCAATGGAAAGATGTTGACCCTGTTGATTTAGTCAATCTTCGAGAATTAAGCATGCATGATATTACCAAACCTTACTCCCTAAACAACATTCGCAGCTTGAAAAACCTTAGCACTCTTAGATTGTTGTGTTGTGCTGATGAATCATTCCCATCTCTTGAATTTGTTAATTGTTGTGAAAAGCTCAAGAAATTGAGGTTAGAAGGGAGAATAGAGAAACTGCCTCATCTGTTTCCAAATTCCATCACAACGATGGTTCTGAGGTTCTCAGTACTGACAGAAGATCCGATGCCTATTTTGGGAATATTGCCAAACCTGAGGAATCTCGATTTGCTTAGAGCTTATGAAGGAAAAGAAATAATGTGCAGTAATAACAGCTTCAGTCAACTAGAGTTCCTTCATCTTTATGATCTTGAGAAGCTAGAAAGATGGGATTTAGGCACAAGTGCCATGCCTCTGATTAAAGGTCTTGGTAACGATGACTGTCCAAATTTAAAGGAGATTCCCGAGAGAATGAAAGACGTGGAGCTGTTGAAGAGGAATTATACGTGGTGA

>PGSC0003DMG400032581

CAAGAATGGAAGCAGAGTTATTATACCACGCACAAAGAGGATGTCGCTGAAAGAGCAGACGACAGAGGTTTTGTCCATAAACTTCGTTTTCTAAGCCATGAAGAAAGTTGGGATCCCTTTTGTAGGAAACTACTTGATGTTCGAGCAATGACCTCAGCAATGGAGAGGCTAGCTATGGATATGGTGGACAAGTGTAGAGGCTTACCTCTTGCAATTGTTGTACTGAGCGGACTACTTTCGCATAAAAGGGGGCTAGATGAATGGCAAAAGGTGAAAGATCACCTTTGGCAGAACATTGAAAACAACTCGATTGAAATCTCCTACATACTATCATTGAGCTCTAACGATTTGTCAACTGCACTCAAGCAGTGTTTTCTGTACTTTGGCCTTTTTCCAGAAGATCAAGAGGTCGATGCTGAATACATAATATGGTTGTGGATGGCCGAGGGTTTCATACCTAATGGAGAAGAAAGAATGGAAGACGAAGCTTCCAAGAAACCATTCCATATCACAACGCGCCAAGGGGATGTCGCTGAAAGAGCAGACGATAGAGGTTTTGTCCATAAACTTCGTTTCCTAAGCCAAGAAGAAAGTTGGGACCTCTTTTGTAGGAAACTACTTGATGTTCGAGCAATGGTTCCAGCAATGGAAAGTCTAGCTAAGGATATGGTGGGAAAATGTAGAGGCTTACCTCTTGCAATTGTTGTATTGAGTGGACTACTTTCGCATAGATGGGGGCTAGACAAATGGCAAAACGTGAAAGACTGCCTTTGGAAGGACATTGAAGAAGACTCTATTGAAATCTCCTACATACTATCATTAAGCTTCAACGATTTGTCATCTGCGCTCAAGAAGTGTTTTCTCTACTTTGGTATTTTTCCAGAAGATCGAGTGATCTATGCTGAAGACATAATGCGGTTGTGGATGGCAGAGGGTTTCATCATACCAAGAGGAGAAGAAAGAATAGAGGATGTCGCTGAAGGCTTCTTGAATGAGCTGATAAGACGAAGCTTGGTTCAAGTGGTGGATACATTTTGGGAAAAAGTTACTAATATTACAATCGAACTACACGAAACAATAGTTATCAACAAAAAGATTTTCTTCATGCCAAAAACACACTGA

>PGSC0003DMG400032578

ATGGTTGATGCCTCTGTGTCATTCGCAGTAAAAAAATTGGGGGATTTTCTCATACAGGAAGCTTCCCTGCGTACAAGTCTCAGACAGGATGTGCGGTGGATGCGAAATGAGCTGTTCTTCATGCAGTCTTTCCTCAAAGATGCAGAACAAAAACAAGTTGTAGATCAAAGAGTTCAACAATGGGTGTTTGAAATCAACTCGATTGCTAATGACGCTGTTGCTATACTCAAGGCTTATAGCTTCGAGGATAGTAAAGGTGACGACGCTAGATTTGCTAGTCGTCTGAAGGCTTAAACTTGCATCTGCAGGAAGGAGACAAAGTTCTATAACGTCACCAAGGAGATCCAATCACTAAAGCATCGAATCATGGATATCTCTCACAAACGAGAGACTTATGGTATTAGAGATATGAATAATGCAGGAGAAAGGCCAAGTAATCGCCCAAACAATCAGTATGACATGGTTAGAACATTGAGGAGAACTACCTCATATGTAGATGAGGATCACATTTTTGTTGGCTTTCAGGATGTTGTACAAACATTGCTAACTGAACTTCTCAAACCAGAGCCTCACAGAAGAGTCATCTCCATTTATGGTATGGGAGGATTAGGCAAGACAACTCTTGCGAGAAACCTATACATCAGTCCTAATATTGCCTCTAGCTTCCCTACACGTGCTTGGATATGTGTTTCTCAAGAGTACAACACCATGGATCTCCTTTGGAATATCATAAAATCCATCCAAGGTTGCACCAAGGAAACTCTAGATTTGTTGGAAAAGATGACAGAGAGAGATCTAGAAATTTACCTTCGTGATCTTTTAAAAGAACCCAAATACCATGTGGTGGTCGATGATTTATGGCATAGAGAAGCATGGGAAAGTTTTGAAATGAGCATTTCCAGAAACGGCAGCAGAGTTATTATTACCACACGCAAAGAGGATGTCGCTGAAAGAGCAGATAACAAAGGTTTTGTCTATAGACTTCGTTTCCTGAGCCAAGAAGAAAGTTGGGATCTCTTTTGTAGGAAACTACTAGATGTTCAGGCAAAGGTCTCAGCAATGGAAAGGCTAGCAAAGGAAATGGTGGACAAGTGTGGAGGTTTACCTCTTGCAGTTGTTGTATTAAGCGGACTACTTTCACATAAAAGGGGNNNNNNNNNNNNNNNNNNNNNNNNNNNNNNNNNNNNNNNNNNNNNNNNNNNNNNNNNNNNNNNNNNNNNNNNNNNNNNNNNNNNNNNNNNNNNNNNNNNNNNNNNNNNNNNNNNACTATCATTAAGCTACAATGATTTGTCAACTGCGCTCAAGCAGTGTTTCCTGTACTTTGGTATTATTCCAGAAGATCATGAGGTCCATGTTGATCACATATTATGGTTGTGGATGGCTGAAGGATATGTACCAACAGGAAAAGAAATAATGGAGGATGTTGCTGAAGGCTTCTTGAATGAGTTGATAAGAAGAAGCTTGATACAAGTGGTACGCACATTTTGGGATAAAGTTGGTAAATGTAGGATTCATGATCTGCTTCGCGATCTTGCCGTACAAAAAGCATTGGAGGTAAACTTTTTTGACATTTATGATCCAAGAAAGCATTCCATATCCTCCTTGTGTCTTAGACATGCCATTCGTAGTCAAGGAAAAAGATATTTATCACTCGATCTTTCTAACTTGAATGTTAGGTCACTTATGTTCCTTGATACGGATTTTTTAAAGATGGGTCTTATAAAGTTCCATAATGTGTTCCAACATCTATATGTGTTGTACTTGGAGATGCGTGTTGACAATATGTCTATAGTACCTGATGCCATAGGAAGTTTGTACCACCTCAAGTTCTTAAGATTTAGAGGTATCCATGATCTTCCCTCTTCCATTGGCAACCTCAAGAATTTACAGACACTTCTTGTCAATGACTATGGATACTTTTGCCAACTACCCCGCGAGACAGCTGACCTGATAAATCTAAGGCATTTAGTTGCTTCGTATTCAAAACCTCTGAAACGTATAAGCAAACTCACTAGTCTTCAAGTTCTTAAAGGCATCCATTGTGATCAGTGGAAAGATGTTGACCCTGTTGATTTAGTCAATCTTCGAGAATTAAGCATGCATGAGATTACCAAAACTTACTCACTAAACAACATTAGCAGCTTGAAAAACCTTAGCACTCTAAAATTGTGTTGTGTAGCTTATGAATCATTCCCAAACCTTGAATATCTTAGTTCTTGTCAGAACCTGGGTTTAGAGGGGCAAATAGAAAAACTGCCTCTCTCAGAGCAGTTTCCGAATTCCATTGCCATGATGGTCCTTCGGTACTCAGAACTCATGGAAGATCCCATGTCTACTCTAGGGATGCTGCCAAATCTAAGGAATCTCGATTTGTTTAGAGCTTACGGAGGAAAAGAAATTACCTGCAGTGATAACAGCTTCAGCCAACTGGAGATCCTTCGTCTTGATTGTCTTGAGAACTTAGAAAGATGGCATTTAGCCACAAGTGCCATGCCTCTGATTAAAGGTCTTGGTATCCAGCGTTGTCAAAAGCTACATGAGATTCCCGATAGAATGAAAAACGTGGAGAGAACTCCTTTTCAATGA

>PGSC0003DMG402032547

ATGGTTGATGCCTTTGTGTCATTTGCAGTTCAAAAACTGGGGGATTTTCTCATACAGGAAGCTTCCCTGCATTCAAGTCTCAGACAGGATGTACGGTGGCTGCGAAATGAGCTGTTCTTCGTGCAGTCTTTCCTCAAAGATGCCGAACAAAAGCAAGTTGTAGATCAAAGAGTTCAACAATGGGTGTTTGAGATCAGCTCTGTTGCTAATGACGCCGTTGCTATATTGGAGACTTATAGCTTCGAGGCTAGTAAAGGTAGTCGTCTGAAAGCTTGCACTTGCATCTGCAGGAAGCAGACAAAATTCTACACCGTCAGAAAGGAGATCCAATCACTCAAGCATCGAATCATGGATATCTCTCGCAAACGAGAGACATATGATATTAGAGATATCAATAATGCAGGAGAAGGGCCAAGTAATCGCCCAAACAATCAGTCTGACATGGTTAGAACATTGAGGAGAACTACCTCATATGTGGATGATGACCAGGATTACATTTTTGTTGGCTATCAGGATATTGTAGAAACATTGCTAGCTGAACTTCTCAAACCAGAGCCTCGCCGAAGCGTCATCTCCATTTATGGTATGGGCGGATTAGGCAAGACAACTCTTGCGAGAAACCTATACATCAGTCCTAATATTGCCTCTAGCTTCCCTACACGTGCTTGGATATGTGTTTCTCAAGAGTACAACACCATGGATCTCCTTTGGAATATCATAAAATCCATCCAAGGTTGCACCAAGGAAACTCTAGATTTGTTGGAAAAGATGACAGAGAGAGATCTAGAAATTTACCTTCGTGATCTTTTAAAAGAACCCAAATACCTTGTGGTGGTCGATGATTTATGGCATAGAGAAGCATGGGAAAGTCTAAAACGAGCATTTCCAGACAGCAAGAATGGCAGCAGAGTTGTTATTACCACGCGCAAAGAAGATGTCGCTGAAAGAGCAGATAACAAAGGTTTTGTCTATAAACTTCGTTTTCTGAGCCAAGAAGAAAGTTGGGATCTCTTTTGTAGGAAACTACTAGATGTTCAGGCAATGGTCTCAGCAATGGAAAGGTTAGCTAAGGATATGGTGGACAGGTGTGGAGGTTTACCTCTTGCAATTGTTGTATTAAGCGGACTACTTTCACATAAAAGGGGTCTAGAAGAATGGCAAAGGGTGAAGGACCACCTTTGGCAGCACATTAAAGATGACTCTATTGAAATTTCCTACATACTATCATTGAGCTACAACGATTTGCCAACTGAGCTCAAGCAGTGTTTCCTGTACTTTGGTATTATTCCAGAAGATCATGAGGTCCATGTTGATCACATATTATGGTTGTGGATGGCTGAAGGATTCATACCAACAGGAGAAGAAATCATGGAGGATGTTGCTGAAGGCTTCTTGAATGAACTGATAAGACGAAGCTTGATACAAGTGGTACGAACATTTTGGGAAAAAGTTAGTAAATGTAGGATTCATGATCTACTTCGCGATCTTGCTGTACAAAAGCATTGGAGGGTAAACTCTTTTGACATTTATGATCCAAGAAAGCACTCCATATCCTCCTTGTGTCTCAGACATGCCATTCATAGTCAAGGAAAAAGGTATCTCACACTTGATCTTTCCAACTTGAAGTTGAGGTCACTTATGTTCCTTGATCCGGATTGTTTAAATATGGGTCCTATAAAGTTCCGTAATGTGTTCCAACATCTATATGTGTTGTACTTGGAGATGCGTGTTGACAATATGTCTATAGTACCTGATGCCATAGGAAGCTTGTACCACCTCAAATTCTTAAGATTGAGAGGTATCCGTGATCTTCCCTCTTCTATTGGCAACCTCAAGAATTTACAGACACTCTTGTCAATGACTATGGATACTTTTGCCAACTACCCCGTGAAACAGCTGACCCTAATAAATCTAAGGCATTTAGTTGTTTCGTATTCAAAACCTCTGAAACTTATTAGCAAACTCACTAGTCTTCAAGTTCTTAAAGCCATCCATTGTGATCAGTGGAAAGATGTTGACCCTGTTGATTTAGTCAATCTTCGAGAATTAACCATGCATGATATTACCAAAACTTACTCCCTAAACAACATTAGTAGCTTGAAAAACCTTAGCTCTCTCAAATTGTTGTGTAAAGATTGTGAATCATTCCCAGCCCTTGAATTTCTTTATTCTTGTCAAAAGCTCCACAAATTGTGGTTACAAGGGAGAATAGAGAAGCTGCCTCTGTCGGACCAATTTCCAAATTCCATCATAATGATGATCCTTTCTAGCTCAGAACTCACAGAAGATCCCATGCCTACTCTAGGGATGCTGCCAAGTCTAAGGAATCTCGATTTGTTTAGAGCTTACGGAGGAAAAGAAATCACTTGCAGTGACAACAGCTTCAGTCAACTGGAGATCCTTCGTCTTGATTGTCTTGAGAACTTAGAAAGATGGCATTTAGCCACAAGTGCCATGCCTCATATTAAAGGTTTTGCTATCCGTCGTTGTCCAAAGCTACATGAGATTCCAAAGAGAATGAAAGACGTGGAGACATTGAAGAGAGACTGTCTTAGATAA

>PGSC0003DMG400032576

ATGGCTGATGCCTTTGTGTCATTTGCAGTTCAAAAATTGGGTGATTTCCTAATACAGAAAGTTTCCCTGCGTAGAAGTCTCAGAGATGAAATTAGATGGATGAAAAATGAGCTACTCTTCATGCAGTCATTCCTAATAGATGCAGAACAAAAACAAAGTGGAGATCAAAGAATTCAACAATGGGTGTTTGAGATCAACTCTATTGCTAATGACGCTGTTGCTATACTCGAGACTTACAGCTTCGAGGCTGGTAAAGGTGCTAGTCGTCTTAAGGTTTGTGCTTGCATCTGTTGGAAGGAGAAGAAATTCTACAATGTCGCAGAGGAGATTCAATCACTCAAGCAACAAATCATGGATATCTCTCGCAAACGAGAGACTTATGGTATTACAAATATCATTAATGCCGGAGAAGGACCAAGTAATCAATCTGCCATGGTAAGAACATTGAGAAGAACTACTTCATATGTGGATGACCAGGATTACATTTTTGTTGGACTTCAGGATGTTGTACAAACATTGCTAGCTCAACTTCTCAAAGAAGAGCCTCGCCGAAGTGTCCTCTCCATTCATGGCATGGGCGGATTAGGCAAGACCACTCTTGCTAGAAACCTCTACAAAAGTCCTAATATAGTCTCAAGCTTCCCTACACGTGCTTGGATATGTGTTTCTCAAGAGTACAACACAATGGATCTTCTTAAGACTATCATAAACTCCATCCAAGGTTGCACCAAGGAAACTCTTGATTTGGTGGAAAAGATGGCAGAAACAAATCTAGAAAATCACCTTCGTAAGCTATTAACAGAACGCAAATACCTTGTGGTGGTTGATGATGTATGGCAGAGAGAAGCATGGGAGAGTTTGAAAAGAGCATTCCCGGATAGCAAGAATGGCAGCAGAGTTATTATTACCACGCGCAAAGAGGATGTCGGCTCAAAGAGCAGACGACAGAGGTTTTGTCCATAAACTTCGTTCCTAAGCCAAGAAGAAAGTTGGGATCTCTTTTGTAGGAAACTACTTGATGTTCGAGCAATGGTTCCAGAAATGGAAAGTCTAGCTAAGGATATGGTGGAAAAGTGTAGAGGCTTACCTCTTGCGATTGTTGTATTGAGCGGACTACTTTCGCATAAAAAGGGGCTAAGCGAATGGCACAAGGTGAAAGATCACCTTTGGAAGAACATTAAAGAAGATAAATCTATTGAAATCTCCTGCATTCTTTCATTAAGCTACAACGATTTGTCAACTGCGCTCAAGAAGTGTTTTCTCTACTTTGGTATTTTTCCAGAAGATCAAGAGGTCGAGGCTGAAAACATAATATGGTTGTGGATGGCCGAGGGTTTCGTACCAAGAGGAGAAGAAAGAATGGAGGATGTCGCTGAAGGCTTCTTGAATGAGCTGATAAGAAGAAGTTTGGTTCAAGTGGCTAAAACATTTTGGGAAAAAGTTACTGAATGTAGGGTTCATGATTTACTTCGTGATCTTGCGATACAAAAGGCATTGGAGGTGAACTTCTTTGACATTTATGATCCAAGAAGCCACTCCATATCTTCTTTATGTATCAGACATGGCATTCACAGTGAAGGAGAAAGGTACCTCTCATCACTAGATCTTTCTAACTTGAAGTTGAGGTCAATTATGTTCTTCGATCCAGATATTTGTAATGTGTTCCAACATATAGATGTGTTTCGACATCTATATGTGTTGTACTTGGATATTAAAGGTAGTGTTATACCTGAAGCCATAGGGAGTTTGTACCACCTCAAGTTCTTAAGATTGAGAGGTATCCGTGATCTTCCCTCTTCCATTGGCAACCTCAAGAATTTACAGACACTTGCTGTTGTCAATGAGGGCAGATCATTTTGCCAGCTACCCCCAAAAACAGTTGACCTAATAAATTTAAGACATTTAGTTGCTCGGTATTCAGAACCTCTAGAACGTATAAGCAAACTCACTAGCCTTCAAGTTCTTAAAGGTGTTTGTTGTGATCAGTGGGAAGATGTTGACCCTGTTGATTTAGTCAATCTTCGAGAATTAAGCATGCATTATATTAGCGATTCTTACTCCCTAAACAACATTAGCAGCTTGAAAAACCTTAGCACTCTCACATTGTCGAGTGAGCTTAATCACTCATCTCCATTCCCATCCCTTGAATTTCTTAATTCTTGTCAAAAGCTCCAGAAATTGTGGTTAAAAGGGAGAATAAACAAACTTCCTCTGTTTCCAAATTCCATCACAATGATGCTTCTTTGGAAGTCAAGCCTGACAGAAGATCCGATGCCCATTTTGGGAATGTTGCCAAACCTAAGGAATCTCGAATTAGAAGAAGCTTATGAAGGAAAAGAAATAGCATGCAGCGATAACAGTTTCAGTCAACTAGAGCTCCTTACTCTTAATGATCTTTACAACCTAGAAAGATGGCATTTAGGCACAAGTGCAATGCCTCTCATTAAAGGTCTTCATATCTATGATTGTCCAAAACTGAAGGAGATTCCGAAGAGAATGAAAGACGTGAAACGTATTTTATAA

>PGSC0003DMG400032572

ATGGCTGATGCCTTTCTGTCATTTGCAGTTCAAAAATTGGGTGATTTCCTCATACAACAAGTTTCCCTGCGTAAAAATCTGAGAAAGGAAATTGAGTGGCTGAGAAATGAGCTACTCTTCATACAGTCTTTCCTCAAAGATGCAGAACTAAAGCAATGTGTAGATCATAGAGTTCAACAATGGGTGTTTGAAATCAATTCTGTTGCGAATGATGCTGTCGCAATACTCGAGACTTACAGCTTTGAGGTTGATAAAGGTGATGACCATGAATTTGCTAGTCGTCTCAAGGCTTGCGCTTGCATATGTAGGAAAGAGAAGAAATTCTACAATGTCGCCGAGGAGATTCAATCACTCAAGCAGCGAATCATAGATATCTCTCGCAAGCGAGAGACTTATGGGATTACAAATATCAATAGTAATGCTGGAGAAGGGCCAAGTAATCAGGTTAGAACATTGAGGAGAACTACCTCATATGTGGATGACCAGGATTACATTTTTGTTGGACTTCAGGATGTTGTACAAACATTGCTAGCTCAACTTCTCAAAGCAGAGCCTCGTCGAAGCGTCCTCTCCATTTATGGCATGGGCGGATTAGGCAAGACCACTCTTGCAAGAAACCTCTACACCAGTCCTAATATAATCTCAAGCTTCCCTACACGCGCTTGGATATGTGTTTCTCAAGAGTACAACATAATGGATCTTCTTAAGACTATCATAAAATCCATCCAAGGTCGCACCAAGGGAACTCTAGGATTGTTAGAAACTATGACAGAAGGAGATCTAGAAGTTCACCTTCGTGATCTATTGAAAGAACACAAATACCTTGTGGTGGTTGATGATGTATGGCAGAGAGAAGCATGGGAAAGTTTGAAAAGAGCATTCCCGGATAGCAAGAATGGCAGCAGAGTTATTATTACCACGCGCAAAGTGGATGTCGCTGAAAGAGCAGACGACAGAGGTTTTGGCCATGAACTTCGTTTCCTAAGCCAAGAAGAAAGTTGGGATCTCTTTTGTAGGAAACTACTTGATGTTCGAGCAATGGTTCCAGCAATGGAAAGTCTAGCTAAGGATATGGTGGAAAAGTGTAGAGGCTTACCTCTTGCAATTGTTGTATTGAGTGGACTACTTTCGCATAGAGGGGGGGTGGACAAATGGCAAGAAGTGAAAGATCAACTTTGGAAGAACATTATTGAAGATAAATTTATTGAAATCTCCTGCATTCTATCCTTAAGCTACAATGATATTTTGTCAACTGTGCTCAAGCAGTGTTTTCTGTACTTTGGTATTTTTCCAGAAGATCAAGAGGTCGATGCTGAAAAGATAATACTGTTGTGGATGGCCGAGGGTTTCATACCAAATGGAGAAGAAAGAATGGAGGATGTGGCTGAAGGCTTCTTGAATGAGCTGATAAGACGAAGCTTGGTTCAAGTGGCTAATACGTTTTGGGAAAAAGTTACTGAATGTAGGGTTCATGATTTACTCCGTGATCTTGCCATACAAAAGGCATCCGACACAAACCTCTTTGACATTTATCATCCAAGAAAGCACTCCAAATCCTCTTCATGTATCAGACTTGCCTTTCATGGTCAAGGAGAAATGTATCACTTACTTGATCTTTCTAATTTGAAGTTGAGGTCAATTATGTTCTTCAATCAGGATTTTCGTAATGTGTTCCAACATATAGATGTGTTTCGACATATATATGTGTTGTACTTGCATATTAAAGGCTGTGGTGCTATACCTGATGCCATAGGAAGTTTGTACCACCTTAAGTTCTTAAGCTTGAGAGCTAACCGTGATCTTCCATCTTCCATTGGCAACCTCAAGAATCTACAGACACTTGTTGTCAATGAGGGGGGATACTACTCCTGCAAACTACCCCGCGAGACAGCTAACCTGATAAATCTAAGACATTTAGTTGTTTGGTATTCAAAACCTCTGGCACATATAAGCAAACTCACTAGTCTTCAAGTTCTTGAAAGAGTTTGTTGTGATCAATGGAAAGATGTTGATCCTGTTGATTTAGTCAATCTTCGAGAATTAAGCATGTTTCATACTAGCAAAACTTACTCCCTAAACAACATTAGCAGCTTGAAAAACCTTAGCACTCTCAAATTGATTTGTGGAGAAACTGAATCATTCCCATCTCTTGAATTTGTTAATTGTTGTGAAAAGCTCCAGAAATTGAGGTTAGAAGGGAGAATAGGGAAACTGCCTCTGTTTCCAAATTCCATCACAATGATGCTTCTTGAGAACTCAAAACTGACGGAAGATCCGATGCCTATTTTGGGAATGTTGCCAAACCTAAGGAATCTCCATTTGGTTGGAGCTTATGAAGGAAAAGAAATAATGTGCAGTGATAACAGCTTCAGTCAACTAGAGTTCCTTCATCTTTATGATCTTTCAAAGCTAGAAAGATGGGATTTAGGCACAAGTGCAATGCCTCTGATTAAAGGTCTTGGTATCCATGACTGTCCAAATTTAAAGGAGATTCCAGAGAGAATGAAAGACGTGGAGCTGTTGAAGAGGAATTATATGTGGTGA

>PGSC0003DMG400016029

ATGATAAGAGTTGCTGCTAGTTATGCAGAAGATGTTGTTGAGATGAAAATTTCTCAAATTATCACAAGCTTAAGCTGGACATTTGGAATTTTACAACACCAGGATTTGCTACCATTTGTTGAAAAAATGGATACAACAAAGAAACAAGTGGCTCGGGGCGTGACAATTGTTTCTCATGATGCTGATCAACTTCTTGAATTATCCTGGAATTCCTTGATTGACACTTCTTCTACAAGTTATCCAATGTTGGAAGATGATATTGTGCACGGACTTGATGATGACTTGGAGATCATAATTAATAGGTTGAAAGGACGATCACGGGATCTAGAGATTGTCACAATATCAGGCATGGGTGGCATTGGCAAAACAACACTCGCTAGGAAAACTTATGATCATCTCGCAATCAGGTATCATCACTTTGACATTCTTGCTTGGGTTACAATATCTCAAGAATTTAGAGTTAGAAATGTATTGTTAGAAGCTTTACGTTGCATTTCAAAGCAAGCAGTTCGTGTCAACGCAAAAGATTATGATAAGATGGATGACTCTGAGTTAGCAGACCTAGTGCAGAAGAACCTAAATCGTCGAAGATACCTTGTTGTTGTTGATGATATTTGGAGTACAGATGTTTGGGATAGTATAAGAGGAATATTTCCTGATTGCAACAATAAAAGTTGAATTTTATTGACTACTAGGGAAACTGTGGTAGCGATATATGCAAATACTAGTATCCCTCATAAGATGAACCTCTTGAGTTTAGCTAACGGTTGGAAGTTACTTTGTGATAAGGTTTTTGGACCAAAACATGATTATCCTCCTGAATTGGAAGAAATTGGAAAGGAAATAGTAGAAAAATGCCAAGGACTACCCTTAACAATTTCAGTGATTGCGGGACATCTTTCTAAAATGACTAGGACATTAGAAGGTTGGAAGGATGTTGTCCAAAACTTAAGTGAAATCATTGCTAGTCATCCAAATCAATGCTTAGGAGTGCTTGGTTTGAGTTACCACCACTTGCCTAATCGCCTCAAACCTTGCTTTCTATCTATGATTAGTTTCCCAAAGGATTTTCTGGTTGAATCTTGTATGTTGATCCAATTATGGATTGCAGAAGGTTTCATAAGGATGTCTGCTGGAAATTGTAAAAGTTTGGAGGAAGTGGCGATAGATTATTTGGAGGACCTTATTAGCAGGAACTTGTTACAGATTAGAAAAAGGAGATTCAATGGTGAGATAAAAGCATGTGGAATACATGATCTACTGCGTGTGTTATGTTTGATAGAAGCTGAAATGACAAAGCATATGCATGTTCAGAGAACCCACCCTACTCTACCAACACAAAAGCATAATACTCGTCGTTTCAGTTTTCAAACTGAGTCTAATTCAGATGATGATTGTTGTAAGCTATTACCCCCTTTTGCCAGATCTATATACTTATTTTCTCAATTGAATCTACCTTTTCCAAATCATATTAAGCTTCTCAGAATCTTGCCCATCCCCCGTTTTTCTCTCGTTTCAACACAATATGGTGAATTTCTCTCCCGTTTCAACCTACTCAGGGTATTGGTCATCTTCAATATAGATGTAGATGTAGATGCAGAGTTCCCACCATTTCCACTAGTGATTACAAAATTATTTCATTTGAGATTTCTCCATTTTCCATTTAACAGCAATATTCCTGACTCAATCTCAGAGCTTCAGAACTTGCAAACTCTAATTTGTAATGGTTATACTTTTTTACCTGGGAAGATATGGATGATGAAGAACTTGAGATATATACGTCTGGAGGGACCCTCTTATTTACCTAGCCCTAGAACATAAAGTCTTGTGACAGGGATGCAAAATCTAGAGGAACTTTCTAATGTCTGCTACGACAGTTGTACAAACGAAGTCTTTTCTGGCATTCCCAATATAAAGAGATTGATCATTCCTGTACCTTTTCTCTGTAAAAACTATTCACCCAATCGGCTCATTGATATGTCCATCTTGAGAAAACTCGAAGCACTCAAGTGTTATTACTGGAGACATAGTTGGATCGTGGGTATCACCATCAGGAGTTTTGTTTTTCCAACATCACTTAATAGGTTGAGTTTAAACAATTGTAGTAATTTTATTTGGGAAGACATATCATCAACTGTTATGATATTGCCAAATCTTGAAGAGCTCAAACTTAAATATTGTCGAACCTTGAATGATGAATGGAGATTGAGTGATGAAGACAAGTTCAAAAGCTTGAAGTTGTTGTTACTGAGCAGCCTAAATCTTGAGCGGTTGGAAGCGAGGAGTGATAACTTCCCAAATCTAAAACGCCTTGTTCTGAAGATGTGCTACAGGTTCCAAGAGACTATTTTCAAATCTATGAGATGA

>PGSC0003DMG400023656

ATGGCAGCCTATACTGCTGTAATTTCTCTTCTTCAAACTCTTGACCAACGAAATCCACAACTCTTTCATGGTCACACTGCTGAAGCGCTCGATTCTCTTCATGCTACTGCCGAATATTTCCAAAACGTTCTTGAAAATGCTAGCAAGAGTAGATTTGACACTGAAAAGATCAAATCTTTGGAGGAAAAAATTAGAGTTGCTGCTAGTTATGCAGAAGATGTTCTTGAGCTGAAAAGTTCTCGAATTGTCAAAGTCTCGAGGTGGAAATTTGGAATTTCACAACACCTGGATTTGCTAAAAGCTGTTAAAAAAATGGATACAACAAAGGAACAAGTGATAAAGATTGTTTCTCATGATACTGATCAAGTTCTCGAATTATCCGGGGATTCCTTGATTGGCACTTCTTCTACAAGTTACACAATGCTGGAAAATGATGTCGTGAACGGACTCGATGGTAATTTGGAGATCATAGTTAATAGGTTGAAAGGACGACCACGGGATCTAGACGTTGTCACAATAACAGGCATGGGAGGCATTGGCAAAACAACACTTGCTAAAAAAGCTTATGATCATCTCACAATCAGGCATCACTTTGACATTCTTGCTTGGGTTACAATATCTCAAGAATTTCGACCTAGAAATGTATTGTTAGAAGCTTTAAACAGCATTTCAAAGCAAACAGATATTGTGAACGCAAAAAATTATGATAAGATGGATGACAATGAGTTAGCTGACCTGGTGCAAAAGAACCTAAAAGGTCGAAGATACCTTGTTGTTGTTGATGATTTTTGGAGTAGGGATGTTTGGGATAGTATACGTAGAATATTTCCTAATTACAATAATGGAAGTCGAATCTTATTGACTACTAGGGAAAACGAGGTAGCAATGTATGCAAATACTTGTAGCCCTCATGAGATGAAACTTTTGAGTTTAGAAAACGGTTGGAAGTTACTTTGTGATAAGGTGTTTGGACCAAAACATGATCATCCTCCTGCATTGGAAGAAATTGGAAAGGAAATAGTAGAAAAATGCCAAGGACTACCCTTAACGATTTCAGTGATTGCGGGACATCTCTCTAAAGTGGCCAGGACATTAGAGGGTTGGAAGGATGTTGCCCGAACCTTAAGTGAAAACATTGCTAGTAATCCAAATAAATGCTTAGGAGTGCTCGGTTTGAGTTACTATCACTTGCCTAATCGCCTCAAGCCTTGTTTTCTTTCTATGAGTAGTTTCCCAGAGGACTTTCAGGTTGATACTCGGAGATTGATCCAATTATGGATTGCAGAAGGTTTCATAAGGACATCTGTTGGAAGTCGTAAAAGCTTGGAGTAAGTGGCATAACATTATTTGGAAGACCTTATTAGCAGGAACTTGATACAAGCTAGAAAAAGGAGATTCAATGGTGAGATAAAAGCATGTGGAGTACATGATCTACTGCGTGAGTTCTGTTTGATAGAAGCTGAAATGACAAAGCATATGCATGTTCAGAGAACTCACCCTATTCTTCCAACACAAAAGCATAATGTTCGTCGCTTCAGTTGTCAAACCTTATCTTATTCAGTTGAAAATTGTTTTAAGGTATTACCCCCTGTGGCCAGATCTATCCACTTATTTTCTCGATTACACCTACTTCATAAACCCCGTCATAAGCTTCTCGGCACATGTCCCATCTACCATCGTAATCCTATAATACATGAATTTTTCTCTCGTTTCAACCTTCTAAGGGTATTGGCCATCTTCAATACAATTGGATACTTCGAATCATTTCCACTTGTGATTACAGAGTTGTTTCATTTGAGATATCTCCAAGTTCCATTTTACGCAGATATTCCTGAATCAATCTCAGAGCTTCAGAATTTGCAAACTCTAATTTGTAGTGGTAATTATTTTGATATAACTTTACCTGAGAACATATGGATGATGAAGAACTTGCGGTATATACGTCTGAAGGGACCCTCTTATTTACCCAGTCCTAGAACAGAAAGTCTTGTGACAGGGATGCCAAATCTAGAGGAACTTTCTATTCTTTGTTACACGAGTTGTACAAATGAAGTCTTTTCTGGCATTTCCAATCTAAAGAGATTGATCATTTCTCTACCTTTTAACAATACAAAGTTTCCCTATCGGCTATTGGATATGTCCAGTTTGACAAAACTCGAAGCATTCAAGTTTTACGGGTCACCTTATTTGGGAAACCCCATCAAGAAATTTGGTTTTCCAACATCACTTAAGAGGTTGAGTTTAAGCAAGTGTAATCATTTTGTTTGGGCAGACATATCATCAACTGTTATGATGTTGCCAAATCTTGAAGAGCTCAAACTTAAACATTGTCGAACCTTGAATGATGAATGGAGATTGAGTGATGAAGACAAGTTCAAAAGCATGAAGTTGTTGTTACTGAGCAAACTAAATCTTGAGCATTGGGAAGCTAGCAGTGATAACTTCCCAAATCTATAACGCCTTGTTCTGAAGAATTGCAACAAGATGCAAGAAATTCCAACAGATTTAGGGGAAATTTGTAGTTTGGAATCAATTGAGTTACATGATTGCAGCACTACTGCTGAGGATTCTGCAAGAAAGATTGTACAAGAACAAGAGGACATGGGAAATAATATCCTTAATATCTATATCCATAACAGTCGCAGTAAGTTGTAA

>PGSC0003DMG400023645

ATGGCAGCTTATAGTGCTGTAATTTCTCTTATTCAATCTCTTGACGAACGAAATTTTCATGAACTCTTTCATGGTCATACTGCTGAAACGCTCGATTCTCTTCGTGCTATTGCTGAATATTTCCAAAAAGTTCTTGATGAATTTGAACCTGGAAAAATCAAATCTTTGGAGGAAAAAATTAGAGATGCTGCTAGTGAGGCCGAAGATATTGTTGAGCTGAAAACGCGTCAAATCATCAAAGGGAGAAGCTGGATGTTTGGAATTTTACAACACCAGGATTTGCTACCACTTGTTGAAAAAATGGATACAATAAAGAAACAGGTGATGGAGATTGTTTCTCATGATGCTGATCAAATTCTTGAATTATCCGGGGATTCCATGATTGGTACTTCTTCTACAAGTTATGCAATGCTGTCAGATAAGTTGGAAGATGGTATCGTTCAGGGAATTGATGATGACTTGGAGATCATAATTAAAAGATTGACAGGACCACCATCGGATCTAGACATTGTCACAATATCAGGCATGGGTGGCATTGGCAAAACAACACTCGCTAGAAAAGCTTATGATCATCTAACAATCAGGTATCACTTTGACATTCTTGCTTGGGTTACAATATCTCAAGAATTTCGATGTAGAAATGTTTTGTTAGAAGCTTTACATTGCATTTTAAAGAAAATAGATATTGTGAATGCAAGAGATTATGATAAGATGGATGACAATGAGTTAGCTGACCTGGTGCAAAAGAACCTTAAGGGTCGAAGATACCTTGTTGTTGTTGATGATATTTGGAGTAGGGATGTTTGGGATAGTATAAGAGGAATATTTCCTAATTACAACAATGGGAGTAGAATCTTATTGACTACTAGAGAAACTGAGGTAGCAATGTATGCAAATGCTTGTAGACCTCATGAGATGAACCTTTTGGATTTAGAAAACGGTTGGAAGTTACTTTGTGATAAGGTGTTTGGACCAAAACATGATCATCCTCCCGAATTGGAAGAAATTGGAAAGGAAATAGTAGAAAAATGCAGAGGACTACCCTTGACAATTTCAGTGATTGCTAGACATCTCTCTAAAGTGGCCAGGACATTCGAAGGTTGGAAGGATGTTGCCCAAACCTTGAGTGAAATTATTGCTAGTCATCCAGATAAATGCTTAGGAGTGCTCGGTTTGAGTTACCACCACTTGCCTAATCACCTCAAACCTTGCTTTCTATCTATGAGTGGTTTCCCAGAGGATTTTCAGTTTGAGACTCTGATATTGATCCAATTATGGATTGCAGAAGGTTTCATTATAAGGACTTCCGAAAATGGTAAAAGTTTGGAGGAAGTGGCAATAGATTATTTGGAGGACCCTATTAGCAGGAACTTGATACAGGCTAGAGAAAGGAGATTCAATGGTGAGATAAAAACATGTGGAATACATGATCTACTGCGTGAGTTCTGTTTGACAGAAGCTGAAATGACAAAGCATATGTATGTTAACAGAACTTACCATACTTTTCCAACACAAATGCATAATGTTCGTCGCTTCAGTTTTCAAGCTTTATCTTATTCAGCTGATGATTGTAGTAAGGTATTACCCTCTGTGGCCAGATCTATTTACTTTTTTTCTTACTTGGATCCACAACTTAAACCCCAATTACATCAATTTTTCTCCCGTTTCAACCTTCTCAAGGTATTGGCCATATTCAATAAACGTGTATGGTTCGAGTAGTTTCCACTTGTGATTACAAAATTGTTTCATTTGAGATATCTCCGAGTTCACTTTAGCGGAGATATTCCTGAATCAATCTCAAAGCTTCAGAATTTGCAAACTCTAATTTGTAGAGGTTTTTCTATTCATGTAACTTTACCTGGGAAGATATGGATGATGAGGAACTTGAGGTATATGCATTTGAGAGAAGCCACTTATTTAGCCAGTCCTAAAACAGAAAGTATTCTAAATAAGCATCTTGGGATGCCAAATCTAGAGGAACTTTCTGGTGTTTGCTTCAACAGTTGTACAAATGAAGTCTTTTCTGGCATTCCCAATATAAAGAGGTTGATCATTCATGTACCTCATTTCAGGGAAATTATTCCCCATCGGCTATTGGATATGTCTAGATTGACGAAACTCGAAGCATTCAAGTTTTACGCAACTTTTTTTCAACACCCCATCAAGAGATTTGGTTTTCCAACATCACTTAGGCGGTTGTCTTTAAATTTTTATCATGATTTTGATTGGGCAGACATATCATCAACTGTTATGATGTTGCCAAATCTTGAAGAGCTCAAACTTAAACATTGTCCAACCATGAGTGATGAATGGATATTGTGTGATGAAGGTAAGTTCAAAAGCTTGAAGTTGTTGTTACTGAGTCTCACAAATCTTGCGCGTTGGGAAGCTAGCAGTAATAACATCCCAAATCTAAAACGCCTAGTTCTGAAGAAGTGTCGCGGCCTGCAAGAAATTCCAGCAGACTTTGGGGAAATTTGTACTTTGGAGTCAATTGAGTTACATGATTGCAGCACTACTGCTGAGGATTCTGCAAGAAATATTGTACAAGAACAAGACGACATGGGAAATAATTTCCTTTAA

>PGSC0003DMG400019732

ATGGCCGCTACTTACGCTGCACTAACTTCTGTGTTGGGAACCATAGACAAGCTTTTACGGTCCAACTTATTAGTAGGCCTAGAAGAGGTTCATAAGCAACAATTGGAATCACTCGACAAGATGTTTGGCACTCTGCAAGTGTCTCTAATTGGCAAATGTGATGGCGGAGATCCAATTATTACCAAGGATTTGCAAAGAAGAATCAAAGATGTTGCACTTGATGCAGAGGATGAAGTTGAATCACTAATGAAACAACTTATTATTGAGCTGGATGATGATGAACAAGTCCTTGAATGTTGTCGTACGAAGCTTGATAAGGTCTCTCAACAGGCTATACAAGTAACTGATTCTGTGGAAGAGCTGATCATCAAGCAGAAGATAAACAATTGCCCAGAAGCTGGAAGTAGCGCTTCTCCACGATTAGATGCTTCAATCCGTGAGAACGTTATGGAAGGGTACAATGAAGAAAGAGAAAGGATGGTTCAAAGACTTACCAGAGGCTCAGGATCAAATAGACAGGAAGTGGTCTCTGTTGTGGGGATGCCGGGCATAGGTAAGACAACTTTTGCCAAAACTATATTATTCGAAAACTCTATTAAGAGGGTCTTTCGTATTCGTGGTTGGATTACTGTGTCTAACAACTATGATTTAAGAAAGTTGCTCCTAGTCCTCCTTCGTGATGTTATTAGAATGGGAGACGGGAATGATAATACAATGGATATTGGAAAACTAGCTGAGCGCGTACAACAAGGTTTGAAGGGAGAAAAGTATTTGATTGTTGTGGATGACATATGGAGTAAAGATGCTTGGGATAGAATTTCACATTGGTTTCCAGATTGTGGTAACAAAAGTCGAATTTTGTTGACTTCTCGAGACAGGGAGGTTGGTGAGTATGCTGCTACTAATCCTAAAGATGGTTTGGTACTGATGCGTCCTCTGACGCAAGATGAAAGTCGGTGTCTGTTTTACCACAAGGCATTTGGGGAAAATTACAGTATTAGAGGGTCAGATATTGATGAATTTGAGAAGGTTGGAGAAAAAGTTATAACAAATTGCAAAGGATTACCTCTAATGATTACTGCAATTGCTGGTATACTCTGTAGCAAGAGTAAATTGGATGAGTGGATGGAAGTAGCTCAAAGTGTAAGCTCATTAGTAAATGATGATGATTACAAACAATGCTTAAAAGTTGTTGCTTTGAGCTACAATCATCTTCCTTCTCTTATGAAAGCTTGCTTTTTGCATTTCGGAGTTTTCCCAAAAGCCCATGTCATTTCTGTGAAGAAGTTAATTAGATTATGGATTGCAGAAGGACTCGTAAATCTAAAAGGAGTTGAGGAATTCGAACAAGTAGCTGCTCGTGTTTTACATGATTTTATTGGGAAAAGTCTTGTTATTGTTGACAAGAGTAGTTTGAATGGGCAAATTAAGACATGTAGGATTCATGATCTTTTTCATGATTTTTGCTCGAAGGAAGCTGAAAGCGAGAATCTTCTGTATGTTGTTCGTTCAGATTCCACCACTATTTCTCAAGTTCACACAAATTTCCGTCAAGGTTGTAGGTGGATGTCAGTTCCATCAATATATTACTCCAGTCAGTATACTCCTATTAAAATACGCTCTCTTTACGGATATTATAAATATTTTAGCGAAGGACTTTTTCATTTCAAACTACTAAGAGTATTGGACTTGGAGGGAGAGACTGCCTCAACACTCGGTAATATTACTGGAGACCTTGTTTGTTTAAGGTATTTGTCTGTCATGACTCATGACACTTTTGCGATTCTCGCAATTACCAATCTTTGGAATCTACAGACTCTCATTTTAGATAAGAAATCACTTAGCCGTATTTTTCATAAAATTGTAACTTTTCCAAAAGAGATTTGGCAAATGTCACAGTTAAGGCATCTTTATTCAATAGGCATTTCTCTATCTTCTCCTGGAGATAAGGTTCTCGGAAACTTACAGAGTGTTTCTGGTTTGAGTCCTCGTTGTTGTACAAAGGAAATATTTGAAGGGATTAAGAAAGTGAAAAAATTGGCCATTCGCGGATGGAATGGGGAATATCCTACCGATCTTAAATGGATAGATAATCTTAAACATTTGCAAGATCTAGAGTCACTAAGTATTGAAATACAATATTGGAGTACAATAAATGGTACCAGGTTTTTTAGTCTTACAAGTTCAGATTCTTTTCCACAAAAACTCAAGAAGTTGAAACTTAGCCGCACATGTCTACCGTGGGAATACATGTCCATTATCAGCAAGTTGCCCGAACTTGAGGTACTCAAACTGAAGTATGATGCCTTAGTTGGCGACGAGTGGAAAGCAACAGACCAGATTGGGTTGCCGAAGTTGAGGTTCTTGCTCCTTGCTAATCTCAACCTTGAAAAATGGAGAACCACCGCCGGCTCTCATGATCATTTCCCCGGCCTTGAGTGCGTAATTATCACAAATTGCAAATTCTTAAAAGAGATTCCTCAAGGATTTGCTGATAGCAAGAAACTGGAGCTGATTGAGTTACACAAATGTGATCCTTCCTTGGTGGCTTTTGCTGAGAAGATCCAGGAAAAACACGAGGATTTGGGGAGGAACAAACTTAAAGTTACTGCCTTCAATTCAGGTAAATATATTTATCTGTCAACATTCAGTAGTTAG

>PGSC0003DMG400019741

ATGGAATCTATAACGGTTGCAGCGGTGAGTCCGGCGGCGACAAAAGCGGTAAGCTTTCTGGTGGATAGTCTATCGCAGCTACTATCGGAAAATGTCGAACTGATAAGAGGTGCGAAGGGAGATTTCCAGCGATTATTGGATGAAATTGAACCCTTAAATGAGTTACTAGCTGGAGATTCTGCACAATTGAAAACCAAAAACAACATCGATTTGGATAAATTGTTCCTGAAAATTCAACGAATAGTATATAAAGCTGAAGATGCGATCGATAAATTCCTAATTCAGGCGAAGATTGACCAAGATAACGTGTTCAGTAAAATCCTTCCAATTCACAAATGGATCCACAATTGGAAAATCGCACCGGAATTCAAGGATATTCTCGAACAAGTGACCGGAATTCGCGAAGAGAGTCAACAGGTTTTCCAGAAAAGCCATATACAGAGTACTGCTTTACAGCCTGGAAAAGGTACGGGGACACAGGTATCTTCTTCCTCGTCTCAATTTATGTTTCAAAAGGGTCCTGCTGAGGAGGATATCGAAGTGGTTGGTTTTGATAAGCCTGCAGAAGATGTTATAAAGCGACTCGGTGAAGGATCCAAGGATCTTGATGTTATACCTATTGTGGGAATGCCAGGCCTTGGAAAGACCACACTGTCAAGAAAAGTTTACAATGATTCTTCCCTTGATTTTCATTTTTACAAGAGAATGTGGATTTATGTTGGGACATCAAAGAAACCAAAGGATATTCTTGTTGAGATTGTGAATGAAGTCGCGCCAAGCAATAGTAAAGAACTAATTAAAGACAAGGATGAGGACCAATTAGCTCATATCATACGTGATTTTCTTGTTGAAAGAGGTAAATATCTCATTGTCTTGGATGATGTGTGGGACACACAAGTTGTAGATTTTGTCAAGAAAGCTTTCCCAAACAACAAATCCCAGCCCCGAGGGGACAGAATCATGTTGACAACTCGCCAACAACGTGTGGCTGAAGCTGTCAGCGCTCGTCCTCACAATCTGGAAAAATTGTCAAAAGGGGATAGTTTCAAGTTGTTGGAACAGAGAGTTTTTGGCAAAACAAGGGAGTGTCCTATTAATTTAAGAGGATATGGAGAAGAGATTGTAGATAAGTGTTGTGGCGTACCACTAGCCATAGTGGTGATTTCAGGAGCTTTGAAAGGTTGTATGGATGAAAGTGAATGGATAGTAGTCAAGGAAAATGTGGGGAAGATCTTTATAAACAAGGACGACGATAAAAGCTGCTTGAAATATGTTGAAACGAGTTACAATCATTTGCCACAACAGAAAAAGGCAGCCTTCTTGTATTTTGGAGTATTTCCTCAAGGCTTTGATATTCCTGCTTGGAAACTTATTCGCTTATGGGTTGCTGAGGGGCTAATAAAGTCCGATCTTCAAGGCAGTGAAATCGAGAAGGTTGCAGAGACTTACTTGAGCGACTTTGCCGGTAGGAATTTAGTGATGGTGATGCAAAAGAGATCTAACGGTCAAATCAAAACATGTCGTCTCCATGACATGTTGCATGAGTTCTGCATTATTGAGGCTAAAAGGATAAGTCTCTTTCAACAAGTATATCTCCAACCTGGTGTTCAAGTTTTTCCTTCTATAGAAGATCCAAATACTTCTCGTCGACTATGTATTCAATCCTCTATTCCGTATAATTTTATCCCTAAAGATAGAATTGTACAGCATGTTAGGTCTCTCTTATGTTTTTCCTCAAACCAAAAGCAAATTGACTTGTCTAATCTAGATATCCAACTCATCCCCTACGCCTTTCCACTCATCAGGGTGTTGGACATTCAATCCCTCATATTTGAATTCTCTGAGATGTTTTATCGGCTATTTCACTTGAGGTATATTGCCATCAAAGGCAACTTCACGGTCCTTCCTTCACTCTTTGGTAATTTTTGGTATTTACAAACCCTTATACTTCATACAGATACTTCAAGCTCCACCCTTGAGATAAAAGAGGACATATGGAAATTGTTACAATTGAGACATCTGCACTCCAACCTTCCTGTGAAATTGCCTCCCCCTCCTACTCCAACAAGCAAGATTCGTACTTCTTGTCTACAAACTCTTTCTAAGGTTACACCAGATAGTTGCAAAAAAACTGTGCTTGCAAAGGTTATTCATCTCAGAAAATTGGGTATTGAAGGGCAATTGGCAGTTCTTCTTGGAAAGTCTACCAAGGAACGTGGATTCGACAGCTTCCAAGAGCTAACACGCATCGAAAATTTGAAATTGTTGAACAATGGTTGGAGTGAAGAGCTTCACCTTCCTCCACACTTTTTCAGCATACTAGAAACACTGAACAAGTTAACTTTGTCAAATACAGGTTTTGAGTGGAGTGAGGCAGATATATTGGGGCAGTTGAAATGCCTTAAGGTACTAAAATTGAAAGAGAATGCATTCATAGGGAATAAATGGGAACCCAAGGAAGGAAGTTTTAGCAAGCTCCAAGTCCTGTGGATTGACTGGGCAGAAAACTGGGAAACTTGGGATGCATCAAATTGTCGGTTCCAAAGTCTTACACACCTTGTTCTTATTTCCTGTTATGATCTCAAGGCTGTGCCACACGAGCTGGCTGATTTACCTTATCTTCAAGAGATGAAACTGATGCGCACATTCAAGGCAGTCAGTTCTGCCATAGAAATCAAAAGCAAGAAACTAGGGAAGCAAAATCCGGAAAGCAGCATCAAATTCAATCTCATTATATTGCCCCCTGCCTCGTCCACAAATTAA

>PGSC0003DMG400019740

ATGGCCGCTACTTACGCTGCACTAACTTCTGTGTTGGGAACCATAGACAAGCTTTTACGGTCCAACTTATTAGTAGGCGTAGAAGAGGTTCATAAGCTACAACTGAAATCACTCGACGAGATGTTTGGCACTCTGCAAGTGTCTCTAACTGGCAAATGTGATGGCGGAGAACCAATTATTACCAAGGATTTGCAAAGAAGAATCAAAGATGTTGCACTTGATGCAGAAGATGAAGTTGATTCACTAATGAAACAACTTATTATTGATCTGGATGATGATGAATGTTGTCGTGCGAATCTTGATAAAGTCTCTCAACAGGTTATACAAGTAACTGATTCTGTCAATGAAGAGCTGATCATCAAGCAGAAGATCAACAATTGTCCAGAAGCTGAAAGTAGCGCTTCTCCACGATTAGATGCTTCAATCTGTGAGAACGTTATGGAAGGGTACAATGAAGAAAGAGAAAGGATGGTTCAAAGACTTACCAGAGGCTCCGGATCAAATAAACTGGAAGTGGTCTCTGTTGTGGGGATGCCGGGCATAGGTAAGACAACTTTCGCCAAAACTATATTATTCGATAACTCTATCAAGAGGGTCTTTCAAATTCGCGGTTGGATTACTGTGTCTAACAACTATGATTTAAGAAAGTTGCTCCTAGTCCTCCTTCGTGATGTTATTAGAATGGGAGACGGGAATGATAATACAATGGATATTGGAAAACTAGCTGAGCACGTACAACAAGGTTTGAAGGGACAAAAGTATTTGATTGTTGTGGATGACATATGGAGCAATCAAGATTGGGATAGAATTTCACATTGGTTTCCAGATTGTGATAACAAGAGTCGAATTTTGTTGACTTCTCGAGACAGGGAGGTTGCTGATTATGCTGCTCGCAATACTAAAGATGGTTTGGTACCGATGCGTCTTCTGACACAGGAGGAAAGTCGGTATCTGTTTTACCACAAGGCATTTGGGAAAAATTACAGTATTCGAGGGTCAGAGGTTGATGAATTCGAGAAGGTTGGAGAGGAAGTTGTAACAAATTGCAAAGGATTACCGCTAATGATTACTGCAGTTGCTGGTATACTCTCTAGCAAGAGTAAACTGGATGAGTGGATGGAAGTAGCTCAAAGTGTAAGCTCATTAGTAAATGATGATGATTACCAACAATGCTTAAAAGTTGTTGCTTTGAGCTACAATCATCTTCCTTCTCTTATGAAAGCTTACTTTTTACATTTCGGACTTTTCCCAAAAGCCCATGTGATTTCTGTGAAGAAGTTGATTAGATTATGGATTGCAGAAGGACTCGTAAATCTAAAGGGAGTTGAGGAATTTGAACAAGTAGCTGCTCGTGTTTTACATGATCTTATCGGGAAAAGTCTTGTTATTGTTGTTGACAAGCGAAGTTTGGATGGGCAAATTAAGACATGTAGGATTCATGATCTTTTTCATGATTTGTGCTTGAAGGAAGCTGAAAATGAGAATCTTTTGTATGTTCTTGGTCCAGACCTTACCACTCATTTAGACAGAAATTTCCATAAAGGTCCCAGGTGGATGTCAATTCAATCAGACTTTAATATTAGTTACTCCAGTCGGTATACTTCTATTAAAATACGCTCTCTTTACATATCTTATATATATTTTAGCAAAGATCTTTTTCATTTCAAACTACTAAGAGTATTGGACATGGAGGACAGTATCATCACAAGTAAAATTACTGGAGAGCTTGTTTGTTTAAAGTATTTGGCTATGTGGCCTAGTGCGACATGTGTGGAACTACCAATTTCCAATCTTTGGAATCTACAGTCTCTCATTTTTAATAAAGGTTCATCTGGATATTTTTTACATAGTGTTGTAGCTTTGCCAAAAGATATTTGGCAAATGTCACAATTAAGGCATCTTTCTGCAAGAGGCATTTATCTATCTTCTCCTGGAGATAAGGTTCTCGGAAACTTACAGTGTGTTTCTGGTTTGAGCCCTTGTTGTTGTACAAAGGAAATATTTGAAGGGATTAAGAAAGTGAAAAAATTGGCCATTTACGGAAGGAAGGAGGAATATCCTACCGATCTTAAATGGACAGATAATCTTAAATATTTACAAGATCTCGAGTCACTAAGTATTACAGCTAAAATGTTTTATGATACAGATAAAACCAGGTTGTTTAGTCTTACAAGTCCAGATTCTTTTCCACAAAAACTCAAGAAGTTGAAACTTAGCTACATAAGTCTACAGTGGGAATACATATCCATTATCAGCAAGTTGCCCGAACTTGAGGTGCTCCAACTGAAGGGTGGTACGTTATTTGGCGACGAGTGGAAAGCAACAGACCAGATTGGGTTCCCGAAGTTGAGGTTCTTGCTCCTTGATAATCTCCTCCTTGAAAAATGGGAAACCACCACAGGCTCTCATGATCATTTCCCCAGCCTTGAGCGCATAATTATCATAGATTGCCACTTCTTAGAAGAGATTCCTCAAGGATTTGCTGATAGCAAGACACTGGAGCTGATTGAGTTACACAAATGTGAACCTTCCTTGGTGGCTTTTGCTGAGAAGATCCAGGAAAAGCACGAGGATTTGGGGAGGAACAAACTTAAAGTTACTGCCTTCGATTCAGGTAAATATATCTATCTGTCAACATACAATATATATAGTTAG

>PGSC0003DMG400041458

ATGGATCTTGTTCAAGAATCTTCAACTTTCCTTTCCATCACCAAGGATGCAAAGGTTCTACTAGATATGCTTCTAGTGAAAGTGAACTCTGTTGCTGTCATAGCATGGGCTATGAATATGCATTCTGAACTCATAAAACTAAATCGTTTTGTTTTAAGAAGAAGAGATGCTACATTGAGTCATGAGGTAGCTGAGATGCTTAAGCTTGCTTGTTATGATGCTGAACGAATCATTGACAAGTATGAAATTTGGTCTCTGCAAAACCAGTTGCTTCCTAGTTCGACATCATCAAAGGTGCGTGGTTTAATTTTCTCCTGTAGATTAAACCCTTTTGCTTATAGAAATCGAGTGTGTCTTGAAATTCGAAAGCTCGTAAAGATATTGGATAAGATAGGAGAAAAGATTGAACTTGTTGAGTGGGACCACACTAACTGCTACATTTTTCTACAATCAGGTTTTGACAGTACACGTACTCGTGTATCTGTACATTACTCTGGTGTTGTGGGTAGGGACATTGATAGAGATACCGTTATCGAAGCACTTTTAAAATCAGCAACCCTTTTTGTATATCCCATTGTTGGAGTTGAAGGTATTGGGAAAACTACTCTTGCCAAGTTGGTGTATAATGATCCAAGGATAGTTAGTCGGTTTCAGCTGCGTTTATGGGTTCGTGTGTCTCGTGTCTTTAAAGTAGAGGAAGTATTAGAACAAATTGTGAATTCAGTTAGAGAAGATTTATGTGAGAAACTTGATATGAATGAACTGAAGAATCTAGTTCATCAGACTTTGTATGGAAAGAATTATTTGATTGTGTTAGATGATGTGTGGAATGAGGACCCACTGAAGTGGGATGAACTGAAGAAGTTGTTGATGGTGGGTGCTTGTGGAAGTAAGATTCTTCTAACTACTCGGAAAAAGGAAGTAGCTTCGATAATGGGGACGGTTCCTGCATACTGTTTGAAGGGTTTGTTACGTGAAGATAGTCTGACTTTGTTCTTGAATAAGGCATTTGAACATGATCAAGAAGTACTATATCCAGAACTTGTTGACATTGCGCAAATGATAGTGCGTAAATGTAAGGGAAATCCCTTATTCTTAATGATTGTAGGATCTTCATTGCGCAAGAAAACTCAGAGATGGGAGTGGGAGATAGTTAACAATCACAGCGGGTGGAACTCAAATCAGAATGGCGAAATTCCATCTGCCCTAAGAGTGAGCTATGAGCAATTGACATCTAATCTCAAAGTTTGTCTTGCTTACTGCTCAATATTTCCCAAGGGCTGTGTGATTGAAATAGATAAATTGATACAGCTGTGGGTAGCACAAGGTCTCATTAGTAATTCTAATGAATCAGAAGATCTTGAGCACGTTGCAATTCAACATTTCCAAGAGTTATTGTCGAGATCCTTTTTTCAAGACGTTGAAGAATATAGTTCCGTTTATACTTCAATCTGTACAATGCATGACCTTGTACATGATCTTGCACTGTCAGCAGCAGGGGTTGAATTCTGTACAGTAAATTCTCACATACAAAACATTTCTGATGAGGTCAGACATGTGGCGTTTTCTGACTATGATTTGTCAGGCAAGGAACTGCCAACATCCCTTGTCAGTAACCAGGCATTAAGGACTATATCCTTCTCCATTGATGGAGTAGGGCCGATGAGTACAATGTTCGTTGAGAATTGCATAGCAAGATTCATGCAACTTAGGGTGCTAGATATCAGTGATTCATGTTTCGATGAGCTGCCTAGCTCTGTTGGCGAATTGAAGCATTTAAGATATCTTGATGTAAGTTCCAATGGAAGCATTAAAGAATTACCTGATTCGATTAACAAGTTGCTGAGCTTACAGACACTTCGAGTTTCTCATTGTCCACAACTTGAAGGGCTGCCTAAAGATATTGGAAATTTGATCAGCCTAAGACACTTATATATAACCACCAAGCAAGCATATTTTCCTGATAAAGCAATTGGCTGCTTATCATCTCTTCGTTCTTTGTGCATTCACAGCTGCAACAATCTCGTATCTTTGTCTGAAGGTCTGCAACATCTGACTAACCTTCGCACTTTGGCAATCATCGGTTGCCCAAGACTGACCTTTTTCCCAAGTGCTATGAAGCACCTTACTGCTTTAGAGAATCTGTTGATTGTTGACTGTAAAGATCTTACATTGTTGGAGTGGCAAGATATTGAAGGACTTAGGATGCTTCGGTCATTGGTTATCGGAGGCTTACCTGAATTGGAGTCAAAAGATGTTCACTGCCTTAGGAGCCTTCAGATGTTGGTACTTGCTGGTTTACCAGAGTTAGTTACTTTGCCGCGATGGCTTGAAGGTGCTAGTGCTACTCTACAATACCTGAGGGTGGAAAGGTGCCTGAATTTTGCAGCACTGCCAAAGTGGCTGGAAAATCTTACTGCACTTGAAAAACTTGAAATTTCCAAGTGCCGTAAATCATTTTCATTGCCCAAGGGGATGAGTCGCCTCACAAACCTGAAGGTACTTAAGATCGACAACTGA

>PGSC0003DMG400037159

ATGGATCTTGTTCAAGAATCTTCAACTTTCCTTTCCATCGCCAAGGATGCAAAGGTTCTACTAGATATGCTTCTAGTGAAAGTGAACTCTGTTGTTAACATTGCAAGGGCTATGAATATGCATTCTGAACTCATAAAACTAGAGCTTTTTGTTGTAACAGAAAGAGTTGCTACATTGAGTCATGAGGTAGCTGAGATGCTTAAGCTTGCTTGTTATGATGCTGAACGAGTCATTGACAAGTATGAAATTTGGTCTCTCAAAAACCAATTGCTTCCTAGTTCGACATCATCAAAGGTGCGTGGTTTAATTTTCTCCGGTAGATTAAACCATTTTGCTTATAGAAATCGAATGTGTCTTGAAATTCGAAAGCTCATAAAGAAATTGGATAAGATAGGAGATTTTGAAGAAAAGATTGAACTTGTTGAGTTGGACTCCTTTCCCATCTGGGTTACTGCACAAGGAAGGTGTGCCAGTTCACGTACTCGTGTATCTGTACATTACTCTGGTGTTGTGGGGAGGGTCATTGATAGAGATACCGTTATCGAAGCACTTTTAAAATCAGGAAATGAAGCAACCCTTTTTGTATATCCCATTGTTGGAGTTGGAGGCATTGGAAAAACTACTCTTGCAAAATTTGTGTACAATGATCCAAAGATAGTTAGTCATTTTCAACTGCTTTTGTGGATTCACGTGTCTCGCGTGTTTAAAGTAGAGGAAGTATTAGAACAAATTGTGAATTCAGTTAGAGAAGATTTATTATGTGAGAAACTTGATATGAATGAACTGAAGAATCTAGTTCATCAGACTTTGTATGGAAAGAATTATTTGATTGTGTTAGACGATGTGTGGAATGAGGACCCAGTGAAGTGGGATGAACTGAAGAAGTTGTTGATGGTGGGTGCTTGTGGAAGTAAGATACTTGTAACTACTCGGAAAAAGGAAGTAGCTTTGATAATGGGGACGGTTCCTGCTTACTGTTTGAAGGGTTTGTTAAATGAAGATAGTATTACTTTGTTCTTGAGTAAGGCATTTGAACAAGGTCAAGAAGTGGACCATCCAAATCTCGTTTGGATTGCGCGAATGATAGTGCGTAAATGTCAGGGAAATCCCTTATTCTTAATGATTGTAGCATCTTCATTGCGCAAGAAAACTGAGAGACGGGAGTGGAGTATAGTTAACAATCACAGTGGGTGGAACTCAAATCAGAATGGCGAAATTTCATCTGCCCTAAGAGTGAGCTATGAGCAATTGACATCTAATCTCAAAGTTTGTCTTGCTTACTGCTCAATATTTCCCAAGGGCTGTGTGATTGAAATAGATAAACTTATACAGCTGTGGGTAGCAGAAGGTCTCATTAGTAAGTCTAATGAATCAGAAGATCTTGAGCACGTTGCGATTCAACATTTCCGAGAGTTATTGTCGAGATCTTTTTTTCAAGACGTTGAAGAATATCGTTCCGTTTATACTTCAATCTGTACAATGCATGACCTTGTACATGATCTTGCACTGTCAGCAGCAGGGGTTGAATTCTGTACAGTAAATTCTCACATACAAAACATTTCTGATGAGGTCAGACATGTGGCGTTTTCTGACTATGATTTGTCAGGCAAGGAACTGCCAACATCCCTTGTCAGTAACCAGGCATTAAGGACCATATCCTTCTCCATTGATGGATTAGGGCCGACGAGTACAATGTTTGTTGAGAATTGCATAGCAAGATTCATGCAACTTAGGGTGCTAGATATCAGTGATTCATGTTTCGATGAGCTGCCTAGCTCTGTTGGCGAATTGAAGTATTTAAGATATCTTGATGTAAGTTCCAATGGAAGCATTAAAGAATTACCTGATTCGATTAACAAGCTGCTGAGCTTACAGACACTTCGAGTTTCTCATTGTCCACAACTTGAAGGGCTGCCTAAAGATATTGGAAATTTGATCAGCCTACGACACTTATATATAACCACCAAGCAAGCATGTTTTCCTGATAAAGCAATTGGCTGCTTATCATCTCTTTGTTCTTTGTACATTCACAGCTGCAACAATCTCGTATCTTTGTCTGAAGGTCTGCAACATCTGACTAACCTTCGCACTTTGGCAATCATCGGTTGCCCAAGACTGACCTTTTTTCCAAGTGCTATGAAACACCTTACTACTTTAGAGAATCTGTTGATTGTTGACTGTAAAGAGCTTACATTGTTGGAGTGGCAAGATATTGAAGGACTTAGGATGCTTCGGTCATTGGTTATCGGAGGCTTACCTGAATTGGAGTCAAAAGATGTTCACTGCCTTAGGAGCCTTCAGATGTTGGTACTTGCTGGTTTACCAGAGTTAGTTACTTTGCCGCGATGGCTTGAAGGTGCTAGTGCTACTCTACAATACCTGAGGGTGGAAAGGTGCCTGAATTTTGCAGCACTGCCAAAGTGGCTGGAAAATCTTACTGCACTTGAAAAACTTGAAATTTCCAAGTGCCGTAAATCATTTTCATTGCCCGAGGGGATGAGTTGCCTCACAAACCTGAAGGTACTTAGGATCGACAACTGA

>PGSC0003DMG400030044

ATGGCTGATGCTTTTGTGTCGTTTGCAGTTCAAAAATTGGGTGATTTCCTCATACAACAAGTTTCTCTGCGTACATCTCTAAGAGAGAAAGTTGAGTGGCTGAGAAATGAGCTACTCTTCATACAGTCTTTCCTCAAAGATGCAGAACTAAAGCAATGTGGAGATCAAAGAGTTCAACAATGGGTGTTTGAGATCAACTCTATTACCAATGATGTTGTTGCTATACTCGAGACTTATAACTTCGAGGCTGGTAAAGGTGATGATGATGGATTTGTTAGTCGTCTCAAGGCTTGCGCTTGCATCTGTAGGAAGGAGACTAAATTCTACAACGTCAGTAAGGAAATTCAATCACTCAAGCAGTGAATCATAGATATCTCTCGTAAGCGAGAGACTTATGGTATTACAAATATCAATAATAATGTAGGAGAAGGGCCAAGTAATAAGGTTACAACGTTGAGGAGAACTACCTCCTATGTGGATGACCAGGATTACATTTTTGTTGGATTTCAGGATGTTGTACAAACATTGCTAGCTGACTTCTCAAAGAAGAGCCTCGTCGAAGCGTCCATTTATGGTATGGGCGGATTAGGCAAGACTACTCTTGCGAGAAACCTCTATAGAAGTTCTAGCATAGTCTCTAGCTTCCCTACACGCGCTTGGATATGTGTTTCTCAAGAGTACAACACAATGGATTTTCTTAAGACTATCATAAAATCCATCCAAGATTGCACCAAAGAAACTCTAGATTTGTTGGAAAGGATGACAGAAAGAGATCTAGAAATTTACCTTCGTGATCTTTTAAAAGAACGCAAATACCTTGTGGTAGGGTTGATGATGTATGGCAGAGAGAAGCATGGGAGAGTTAAAAGAGCATTCCCGGATAGCAAGAATGAAAGCAGAGTTATTATTACCACGCGCAAAGAGGATGTCGCTGAAAGAGTAGACGACAGAGGTTTTGTCCATAAACTTCATTTCCTAAGCAAAGAAGAAAGTTGGGATCTCTTTTGTAGGAAGCTACTTGATGTTCGAGCAATGATTCCAAAAATGGAAAGTCTAGCTAAGGATATGGTGGAAAAGTGTAGAGGCTTACCTCTTGCGATTGTTGTACTGAGCGGACTACTTTCGCATAAAAAGGGGCTAGACGAATGGCAAAAGGTGAAAGATCACCTTTGGAAGAACATTATTGAAGATAAATATATTGAAGTCTCCAACATACTATCATTAAGCTACAGTGATTTGTCAACTGCGCTCAAGCAGTGTTTTCAGTACTTTGGTATTTTTCCAGAAGATAAAGTGGTCGATGCTGAAAACATAATATGGTTGTCGATGGACGAGGGTTTCATACCAAGAGGAGAAGAAAGAATGGAGGATGTTGCTGAAGGCTTCTTGAATGAGCTGATAAGACGAAGCTTGATACAAGTGGTAGATACAGTTTGGGAAAAATTTACTGAATGTAGGGTTCATGATTTACTCCGTGATCTTGCCATACAAAAGGCATTGGAGGTAAACTTCTTTGACATTTATGATCCAAGAAGCCACTTCGTATCCTCTTTATGTATCAGACATGCCATTCATAGTCAAGGAGAAAGGTACCTCTCATTTGATCTTTCTAACTTGAAATTGAGGTCAATTATGTTCTTCGATCCAGATTTTCATAAGATGAGTCTTATAAACTTCAGTAGTGTGTTCCAACATCTATATGTGTTGTACTTGGAGATGTGTTTTAATAGTTTGTCTATAGTACATGATGCCATAGGAAGTTGTACCACCTCAAGTTCTGGGCAGATTCTCTTGCGAACTACCCCTGAGACAGCTGACCTAATAAATCTAAGACATTTAGATTCTCTGTATTCAAAACCTTTGAAACGTCTAAGCAAACTCACTAGTCTTCAAGTTCTTAAAGGTGTTCTTTGTGATCAGTGGAAAGATGTTGAACCTGTTGATTTAGTCAATCTTCGAGAATTAACCATGCATGATATTACCAAAACTTGCTCCTTAAACAACATTAGCAGCTTGAAAAACCTTAGCACTACCAGATTGTTTTGTGAAGGTTATGAATCATTCCCATCTCTTGAATTTCTTAATTGTTGTGAAAAGCTCCAGAAATTGTGGTTAAAAGGGAGAATAGAGAAACTGCCTTTGTTTCCAAATTCCATCACCATGATGTTTCTGAGGAACTCAAAACTCAGAGAAGATCTGATGCCTATTTTGGGAATGTTGCCAAACCTAAGGAATCTCATATTGTATGGAGCTTACGAAGGAAAAGAAATAATGTGCAATGATAACAGCTTCAGTCAACTAAAGTTCCTTTGTCTTGATCAACTTTCAAACCTAAAAATATGGCATTTAGGCACAAATGTCATGCCTCTCATTAATGATCTTCATATCAAAAACTGTTGAAACCTAAAGGAGATTCCTCAGAGAATGAAAGACGTGGCAAGCATATATGAAGTCTTAATTGATAATAGGAAAGGTAAACATACAAACAAATCTCAATCCTACATAACTTTCTTCATTTTATAG

>PGSC0003DMG400030045

ATGGCTGATGCTTTTGTGTCGTTTGCAGTTCAAAAATTGGGTGATTTCCTCATACAACAAGTTTCTCTGCGTACATCTCTAAGAGAGAAAGTTGAGTGGCTGAGAAATGAGCTACTCTTCATACAGTCTTTCCTCAAAGATGCAGAACTAAAGCAATGTGGAGATCAAAGAGTTCAACAATGGGTGTTTGAGATCAACTCTATTACCAATGATGTTGTTGCTATACTCGAGACTTATAACTTCGAGGCTGGTAAAGGTGATGATGATGGATTTGTTAGTCGTCTCAAGGCTTGCGCTTGCATCTGTAGGAAGGAGACTAAATTCTACAACGTCAGTAAGGAAATTCAATCACTCAAGCAGTGAATCATAGATATCTCTCGTAAGCGAGAGACTTATGGTATTACAAATATCAATAATAATGTAGGAGAAGGGCCAAGTAATAAGGTTACAACGTTGAGGAGAACTACCTCCTATGTGGATGACCAGGATTACATTTTTGTTGGATTTCAGGATGTTGTACAAACATTGCTAGCTGACTTCTCAAAGAAGAGCCTCGTCGAAGCGTCCATTTATGGTATGGGCGGATTAGGCAAGACTACTCTTGCGAGAAACCTCTATAGAAGTTCTAGCATAGTCTCTAGCTTCCCTACACGCGCTTGGATATGTGTTTCTCAAGAGTACAACACAATGGATTTTCTTAAGACTATCATAAAATCCATCCAAGATTGCACCAAAGAAACTCTAGATTTGTTGGAAAGGATGACAGAAAGAGATCTAGAAATTTACCTTCGTGATCTTTTAAAAGAACGCAAATACCTTGTGGTAGGGTTGATGATGTATGGCAGAGAGAAGCATGGGAGAGTTAGAGCATTCCCGGATAGCAAGAATGAAAGCAGAGTTATTATTACCACGCGCAAAGAGGATGTCGCTGAAAGAGTAGACGACAGAGGTTTTGTCCATAAACTTCATTTCCTAAGCAAAGAAGAAAGTTGGGATCTCTTTTGTAGGAAGCTACTTGATGTTCGAGCAATGATTCCAAAAATGGAAAGTCTAGCTAAGGATATGGTGGAAAAGTGTAGAGGCTTACCTCTTGCGATTGTTGTACTGAGCGGACTACTTTCGCATAAAAAGGGGCTAGACGAATGGCAAAAGGTGAAAGATCACCTTTGGAAGAACATTATTGAAGATAAATATATTGAAGTCTCCAACATACTATCATTAAGCTACAGTGATTTGTCAACTGCGCTCAAGCAGTGTTTTCAGTACTTTGGTATTTTTCCAGAAGATAAAGTGGTCGATGCTGAAAACATAATATGGTTGTCGATGGACGAGGGTTTCATACCAAGAGGAGAAGAAAGAATGGAGGATGTTGCTGAAGGCTTCTTGAATGAGCTGATAAGACGAAGCTTGATACAAGTGGTAGATACAGTTTGGGAAAAATTTACTGAATGTAGGGTTCATGATTTACTCCGTGATCTTGCCATACAAAAGGCATTGGAGGTAAACTTCTTTGACATTTATGATCCAAGAAGCCACTTCGTATCCTCTTTATGTATCAGACATGCCATTCATAGTCAAGGAGAAAGGTACCTCTCATTTGATCTTTCTAACTTGAAATTGAGGTCAATTATGTTCTTCGATCCAGATTTTCATAAGATGAGTCTTATAAACTTCAGTAGTGTGTTCCAACATCTATATGTGTTGTACTTGGAGATGTGTTTTAATAGTTTGTCTATAGTACATGATGCCATAGGAAGTTGTACCACCTCAAGTTCTAGATTGAGAGGTATCTGTAATCTTCACTCTTCCATTGGCAACCTCAAGAATCTACAGACACTTTTTGTTGTCAATGAGGGCAGATTCTCTTGCGAACTACCCCTGAGACAGCTGACCATAAATCTAAGACATTTAGATTCTCTGTATTCAAAACCTTTGAAACGTCTAAGCAAACTCACTAGTCTTCAAGTTCTTAAAGGTGTTCTTTGTGATCAGTGGAAAGATGTTGAACCTGTTGATTTAGTCAATCTTCGAGAATTAACCATGCATGATATTACCAAAACTTGCTCCTTAAACAACATTAGCAGCTTGAAAAACCTTAGCACTACCAGATTGTTTTGTGAAGGTTATGAATCATTCCCATCTCTTGAATTTCTTAATTGTTGTGAAAAGCTCCAGAAATTGTGGTTAAAAGGGAGAATAGAGAAACTGCCTTTGTTTCCAAATTCCATCACCATGATGTTTCTGAGGAACTCAAAACTCAGAGAAGATCTGATGCCTATTTTGGGAATGTTGCCAAACCTAAGGAATCTCATATTGTATGGAGCTTACGAAGGAAAAGAAATAATGTGCAATGATAACAGCTTCAGTCAACTAAAGTTCCTTTGTCTTGATCAACTTTCAAACCTAAAAATATGGCATTTAGGCACAAATGTCATGCCTCTCATTAATGATCTTCATATCAAAAACTGTTGAAACCTAAAGGAGATTCCTCAGAGAATGAAAGACGTGGCAAGCATATATGAAGTCTTAATTGATAATAGGAAAGGTAAACATACAAACAAATCTCAATCCTACATAACTTTCTTCATTTTATAG

>PGSC0003DMG400020935

ATGCATACTCTATTTGTTGAAGGAGAATCATCTAATTCTTTCCAATGGAGGTACGATGTATTTCTAAGTTTCAGTGGTGAAGATACTCGAAAAAACTTCATCAGTCATCTTAAATTTCGATTGTGTCAAGTTGGAATTTGTACCTTTATAGATGATGAGGAAGTGAGGAAGGGCGAGGTCATTTCAACGGAACTTGAAAAAGCAATTGAACAATCTAGAGTTTCCATTGTTGTTTTCTCGAAAAAATATGCTTCATCTAGTTGGTGTCTTGAAGAACTTGTTAAAATTCTCGAATGCAGAGAGACGTTGAAGAAGGTTGTTTTGCCTATATTTTATGATGTTGATCCTTCTCAAGTACGCAACCCAATTGGATATTTTGATGAATCTTTGACTAGACGATTTGGGGCACAAAGAACGGAGAAGTGGAAAACTGCACTTACTAAAGTTGCAAATTTATCTGGATGGGATTCGCGAAATGTTGTTTATGGGTACTATTTCTTGATTCAACTATTTAATTTTATCTTTAATTTGACCTTTGTTTATACGATGATACAAATTCATTTATTTTTATTTTTGCTAAAAATTTGTAGGCATGAATCAGAATTAATTGAAAGTATTATAAAGCGAGTCTTACAAGAGGTAAGCCAGACATCTCTAGATGTTGCTTGTTACCCTGTTGGAATTGATTCTAGTATCAAAGATCTAATAGAGTTGTTATTCAAAAGTGGATGTCAAGAAGACGTTCGGATGATTGGTATATATGGCATTGGTGGAATTGGAAAAACTACTCTTGCTAAAGCTTTCTACAACCAAATATGTCGACACTTCGGTAGTAGTTGTTTCCTTTCAAATGTTAGATCAGAAGCTGGCACATTTAATGGTCTAGTCAAGTTACAAGAAAAACTTCTTCATCAAATCCTAAAAACTAAGGATTTCGAAGTTAATGATGTTGCTGAAGGCGTTAGTTTAATCAAAGCAAGACTAGGGTCAATGAAAGTTCTGATTGTTCTTGATGATGTGGACCATATAAGCCAATTAGAATCCTTAATAAGAGAAAGAAATTGGTTTGGTTCGGGTAGTTTAATAATTGTTACAACCCGAGACAAGCATTTGCTATGTGGGCTTACAACAAAAGAAAAATTCAAGGCCAAACTTTTATATGACAATGAAGCTATGCAACTCTTTAGTTGTCGTGCCTTTAATAGTTTTTTTCCACCACATGAGTATGTTGAGTTGTCACAAGAAATAATCAAATATTCAGGTGGTCTGCCATTAGCTCTTGTGACATTGGGGTCACATTTGCGAGGAAGATCGGTAGAAGAATGGAGACATGAATTCGTTAAACTAAGAGCAATTCCTCATAGTGATATTCAAAAGATTCTCAAGATAAGCTTTGACGGACTTGATTATGATACTCAAAGTGTTTTCCTTGATATTGCATGCGCCTTCCATGGATTCTTTGAGGATGAAGTTACCAAAATACTAAATGCGTGTGGTTTTTATTCTGAAAGTGCAATTGCAACTTTAGTCCAAAAACACTTGCTCCATAGGGCTTGGCATCGCTTAGTGATGCATGATCTAGTGCGAGCCATGGGAAGAGAAATCGTTCGCATGGAATCACCCCGAGACCCGGGAAAACGGAGTAGATTGGTCATCCCTCAAGAAGTCTGCTATGTTTTACAAGGAAATAAAGTTAGTAATTCTCCACCAATTTATATAAGACATTTTTTTCCTCTTAATTTCTACGACTTAACATGGTCTTACGATTTCTTTTATTGTTGTAAAAGTTTGAAGAGGTTGGATCTTTCTGATTGCAAGAGTCTCAAAAGAACTCCGAACTTCAACGGTTTACAAAGTCTTGAGTTTTTGTTGCTAAATGGTTGCTCAAGTCTGAGAAAGATCCATCCATCAATAGGAAATTTGTGCAGACTAAGACTTCTAAATTTGCGTGGTTGCAAAAAGCTTATGGATCCTCCGAGTAGTATATGTCAACTAAAATCCCTTGGATGGTTGGACATTAGTGGCTGCTCATATATAAAAACACTGCCGGTTGACTTTGGAGTTATGCCAGGTCTAAGAACTCTTTCTGCACTTGAAACGGATATAAAACAATGGCACGGGTTTGTTGAAATGCCAAGACATCTTGAATCATTGAAAGTGGGAGGTGAAAATTTGCAAGGCAAAAGGAGATCTCTTGGAAGACGAGTCCATTGGATACAATCCTTGTCGACTTCTCTTTCGTGTTTAAGTCTTATATATTGTGGCTTCTCCGAGACTGATATACCTAGGGATATAGGGAAATTATACAACTTAACTTATCTAAATTTGAGTGGCAACAGCTTTCGCTGTCTACCTTTTGACTTTTCTGAGTTACAATTGTTGTGTTCATTGAATTTGAATGACTGTGAGGATCTTGAAACACTCCCATCAGTATCTAATTTAAAATACCTTCGGACTTTTGAAGTTGCAAATTGCAGGAAGTTGGTCAACATTACAGGGCTAGAAAATCTCCCTTCTATAGAGCGGATCAACATGCTTAATTGTACTTCACTGCAGAATCCATTCAAAGAAGGCTTCTTTAGTGCACCTGCTCTAGCATTTCCATCTAGAGAATATCCACATCTGGTTAGGCTCTCTCTCTTTCTCTCTCTCTCTCTCTCTTTCCTGTCATAG

>PGSC0003DMG400002947

AAGGGTCCTTCTTTGGAGAATGATGCAGTGGTCGGGTTTGATGACGAAGCTAATAAAGTGATCAAGCGACTTGTTGAAGGACCGATGGACAGTGTGGATATTATCCCAGTTGTGGGAATGCCCGGACTTGGAAAAACTACACTGGCAAGAAAAATCTATAATGATTCTAAGCTTACAAATAAATTTTATAGCATTGTTTGGGTTTACGTCGGTCAGGAATGCAAAGCAAAGGATATTTATCTTAGGATTCTTAAATTCTTCAAAAAAACCATAGAAGATCATCTCAATGACGATGTGGACACATTTGGCATGATGTGTGGGAAGTTGAGTTATGATCATTTGCCCCAAGAAGTGCAGATGTGCTTCTTGTATTGTGGTGTCTTTCCTCGAGGCTTTGATATCCCTTGTTGGAAATTGATTCGTTTGTGGATAGCAGAGGGGTTGATAAAGCCGCAGCCTAACTTCACTCTAGAGGAGATAGCAGAGTTTTGTTTGACAGATCTTCTCAACAGAAATTTAGTAATAATAATGCAAAAGAGGTCTGATGGTCAAATAAAAACATGTCGTCTTCACGATATGTTGTATCAGTTCTGCAAAAAGGAGGCTAGTAACAGATGGCTTTTTCAAGAACCTGATCAATCTACCCTGGACCCAGATACTTGCCGTCAATTGTGTATTCAACCATCTAATCTGTCTGACTTTCTCTCCAAACACCTTTTGCAGAACATGTCAGATCTTTCTATTGTTTTTCCTCAAAACAAAAACCAATTGACTTGTCTCCTAATGAAATCAAACTCATTCACAAAGCCTTTCCACTTAAGAGTCTTGGATGTTGAATCTCTCAAGTTTATTTTCTCCAAAGACTTCAACCAATTATTTCATTTGAGGTATATTGCTATCTCAGGTGACTTTAAGGCCCTCCCTCCTACCTTTGGTAAATTCTGGAATTTACAAACTCTAATACTTAATACAAGTACCTTAGAGCCCACTCTTGAAGTAAAAGCAGAAATATGGAACTTGTTACAGTTGAGGCATCTCCACACCAACATACCTGCAAAATTGCCATCCCCGACTACCACCACAGGTAAACCTTCTTGTCTACAGACTCTTTCTATGGTTGCGCCAGAAAGTTGTGAGAAAGATGTGCTAGCAAAGGCCTGTAATGTAAGAAAATTGAGCATTCAAGGGCAAATGGCGGCTTTTCTTGGTGCTTACAAGGGTGGTATCAACAATCTTGAAGAGCTACAGTGTCTGGAACATTTGAAATTGTTGAATGATGTTCTTTTCATAAATAAAACACTTCACCTTCCACTAGCATTCTCCAAACTGGTACGTACGGTGAAGAAGTTGACTCTGACAAACACAAGGTTTGCTTGGAGTGAGGCGGATAAATTGGGGGACGTTTGGAATCTCTTTGAGGTCCTAAAGTTTAAGAAAATGCATTCACGGGTGATCTCTGGAAGCCAAAGAGTGGATTTAGCGCACTCCAAGTCTTGTGGATTGAAAGGTCAGAATTAG

>PGSC0003DMG400002960

ATGGCAGCTTATACTGCTGTAATTTCTCTTCTTCAAACTCTTGATCAACCAAATATTTCAGAACTCTTTCATGATCACAGTACTGAAATGTTCGATTCTCTTCGTGCTACAGCTGAATATTTTCAAGATGTTCTTGAAAACACTAGCAATGAGAAAATCAAATCTTTGGAGGAAGATATTAGAGTTGTTGTTAGTGAGGCAGAGGATGTTGTTGAAATGAAGATTTCTGAAATCATCAAAGAAGAAAGCTGGACATTTGGAATTTTACAACACCATGATATGCTACCAGTTGTTGAAAAAATGGATACCACAAAGAAACAAGTGATGGAGATTCTTTGTCATGATGCTGATCAAATTCTTGAATTAACCGGGGATTCCTTGATTGGCGCTTCTTCTATGAGTGATCCAATGCTGTCAGATCAGTTGGAAGATGATATTGTGCAGGGACTTGATGGTGACTTGGAGATAATAGATAAGAGATTGAGAGGACCAACGTCGGATCTAGACATTGTCACCATATCAGGTATGGGTGGCATTGGCAAAACAACACTCGCTAAAAAAACTTATGATCATCTTCATTTTTGTTGGGTTACAATATCTCAAGAATTTCAATATAGAAATGTATTGTTAGAAGCTTTACATTGCATTTCAAAGCAAAGAGTTATTGAGAACACAAAAGATTATGATAAGATGAATGACAGTGAGTTAGCTGATCTGGTGCAAAAGAGTCTAAAGGGTCCTAGATACCTTGTTGTTGTTGATGATATTTGGAGTAGGGATGTTTGGGATAGCATATCACAAATATTTCCTAATCGCAACAATGGGAGTCGAGTCTTATTGACTACTAGGGAAACTGATGTAGCAATATATGCAAATACTAGTAGCCCTCATAAGATGAACCTCTTGGATTTAGATAATAGTTGGAAGTTACTCCGTGATAAGGTGTTTGGACTAGAATACGATCATCCTCCTGAGTTGGAAGAAATTGGAAAAAAAATAGCAGAAAAATGTCAAGGACTTCCCTTAATAATTTCAGTGATTGCAGGGCATCTCTCTAAAACGGCCAAGACATTAAAAAGTTGGGAGTATGTTGCCAAAACCTTAAATGAAATCATTGCTAGCCATCCAAATAAATGCTTAGGAGTGCTCGGTTTGAGTTACCATCACTTGCCTAATCGACTCAAACCATGCTTTCTTTCTATGGGTAATTTCCCAGAGGATTTTCAAGTTGATACTTGGAGATTGATCCAATTATGGATCGCAGAAGGTTTCATAAGGGCACCTGCTGGAAGTCGTAAAAGCTTGGAGGAAGTGGCAAAATGTTATTTGGAGGATCTTATCAGCAGGAACTTGATAATGGCTTGAAAAAGGAGATTTAATGGTGAGGTTAAAGTATGCGGAATACATGATCTGCTGCGTGAGTTCTGTTTCATAGAAGCTGAAATGACTAAGTTTATGCATGTTGTGAGAACTCCATTTTCACCAACACAAAAGCCTAATGTTCGTCGTTTCAGTATTCAAAAGGCATTTTTAGATGAGTATGCTTATAAGCTATTACCTCCTGTTGCCAGATCTATGTACTACTTTTCTCTATTGAATCTACCTTTTGAACCCCGTATTAAGCTTCTCGGGATATTGCCCATCTACCGTCATGATCCTATAATACATGGATTTTTCTCTCGTTTCAACCTTCTCAGGGTATTGGCCATCTTCAATACAAATGTCAGGTTCGAGTCATTTCCAGTTGTGATTACAAAGTTATTTCATTTGAGATATCTCCAAATTCGATGTTTTGGCAATATTCCTGAATCAATCTCAGAGCTTCAGAATTTGCAAACTCTAATTTGTGATGGTCATTCTTTAGATATAACTTTACCTGAGAAGATATGGATGATGAAGAATTTGAGGTATATACGTTTAGGGGGAGCCAGTTATTTACCAAGTCCTAGAATAGATAACAAGATGCCAAATCTAGAGGAATTATTCAGTATTTGTTACTCTAGTTGTACAAATGAAGTCTTTTCTAGCATTCCCAATCTAAAGAGATTGACCATTCGTCTACCTTTGAGTGTCGGAGACAATATTCCCTCTCAGCTCATCGATATGTCCAGCTTGAGAAAACTCGAGTCAATCAAGTTTAACTGGTTACTTCCTTTCAAAAACCCCATCAACATATTTGCTTTTACAACATCACTTAGGACGTTGTCTTTAACTGATTGTGCTAATTTTATTTGGAAAGACATATCATCAACTTTTATCATGTTACCAAATCTTGAAGAGCTCAAACTTAAAAGTTGTCGAGCCGAAGATGATGTATGGAGATTGAGTGATAAAGACATTTTCAAAAGCTTGAAGTTGTTGTTACTGAAATACCCAAATCTTAAGTGTTGGGAAGCTAGCAGTGATAACTTCCCAAATCTAAAACGTCTCGTTCTCATGAATTGCAACAACCTGCAAGAAATTCCAACAGATTTTGGGGAAATTTGTACTTTACAATCGATTGAGTTACATAATTGCAGCACTTCTGCTGATGAGTCTGCCATAAATATTGTACAAGAACAAGAGGAAATGGGAAATAATATCCTTAAGGTCTACATCAGCCACGGTAAGTTTTAA

>PGSC0003DMG400024231

ATGGCTGATGCCTTTCTATCATTTGCAGTTCAAAAATTGGGTGATTTCCTAATACAACAAATTTCCCTGCGTAAAAATCTGAGAAAGGAAATTGAGTGGCTGAGAAATGAGCTACTCTTCATACAATCATTCCTCAAAGATGCAGAACAAAAACAATATGGAGATAATAGAGTTCAACAATGGGTGTTTGAGATCAACTCTATTGCTAATGATGCTGTTGCTATACTCGAGACTTATAGCTTCGAGGCTGGTAAAGGTGCTAGTCGTGTCAAGGCTTGTGCTTGCATCTGTAGGAAGGAGAAGAAATTCTATAATGTTTCCAAGGAAATTCAATCACTCAAGCAACAAATCATGGATATCTCTCGCAAACGAGAGACATATGGTATTACAAATATCAATAGTAATGCAGGAGAAGGACCAAGTAATCAGTCTGCCATGGTTAGAACATTGAGGAGAACTACCTCATATGTAGATGACCAGGATTACATTTTTGTTGGACTTCAGGATGTCGTACAAGCATTGCTAGCTCAACTTCTCAAAGCAGAGCCTCGTCGAAGTGTTGTCTCCATTTATGGCATGGGCGGATTAGGCAAGACCACTCTTGCAAGAAACCTCTACAGAAGTCCTAATATGGTCTCTAGCTTCCCTACACGCGCTTGGATATGTGTTTCACAAGAGTACAACACAATGGATCTTCTTAAAACTACCATAAAATCCATCCAAGGCCGCACCAAGGAAACTCTACAATTGTTAGAAACTATGACAGAAGGAGATCTAGAATTTCACCTTCATGATCTATTGAAAGAACGCAAATACCTTGTGGTGGTTGATGATGTATGGCAGAGAGAAGCATGGGAAAGTTTGAAAAGAGCATTCCCGGATGGCAAGAATGGCAGCAGAGTTATTATTACCACGCGCAAAGAGGATGTCGCTGAAAGAGCAGACGACAGAGGTTTTGGCCATGAACTTCGTTTCCTAAGCCAAGAAGAAAGTTGGGATCTCTTTTGTAGGAAACTGCTTGATGTTGGAGCAATGGTTCCAGAAATGGAAAGTCTAGCTAAGGATATGGTGGAAAAGTGTAGAGGCTTACCTCTTGCAATTGTTGTATTGAGTGGACTACTTTCGCATAGAGGGGGGGTGGACAAATGGCAAGAGGTGAAAGATCACCTTTGGAAGAACATTATGAAAGATAAATCTATTGAAATCTCCTGCATTCTATCATTAAGCTACAACGATTTGTCAACTGTGCTCAAGCAGTGTTTTCTGTACTTTGGTATTTTTCCAGAAGATCAAGTGCTCAAGGCTGAAAACATAATAAGGTTGTGGATGGCCGAGGGTTTCGTACCAAATGGAGATGAAAGAATGGAGGATGTCGCTGAAGGCTTCTTGAATGAGCTGATAAGACGAAGCTTGGTTCAAGTGGTGGATACATTTTGGGAAAAAGTTACTGACTGTAGGGTTCATGATTTACTCCGTGATCTTGCCATAAAAAAAGGCATTGGAGGTAAACTTATTTATCATCCAAGAAAGCACTCCAAATCCGCTTCATGTATCAGACTTGCCATTCATAGTCAAGGAGAAAGATATCACTCACTTGATCTTTCTAATTTGAAGTTGAGGTCAATTATGTTCTTTGATCCACATTTTCGTAATGTGTTCCAACATATAGATGTGTTTCGACATATATATGTGTTGTACTTGCATATTAAATATGGTGGTATTATACCTGATGCCATTGGGAGTTTGTACCACCTCAAGTTCTTAAGCTTGAGAGGTATCGATGACCTTCCGTCTTCCATTGGCAACCTCAAGAATCTACAGACACTTGTTGTCAATGAGGCGGATTACACTTTCCAACTACCCCGCAAGACAGCTGACCTAATAAATTTAAGACATTTAGTTGCTCAGTATTCAGAACCTCTGGTACATATAAGCAAACTCACTAGTCTTCAAGTTCTTAAAGGTGTTGGCTGTGATCAATGGAAAGACGTTGACCCTGTTGATTTAGTCAATCTTAGAGAATTAGGAATGGATTATATCAAGAAATCTTACTCCTTAAACAAAATTAGCAGCTTGAAAAACCTTAACACTCTCACATTGGTGTGTATAAATTATGATGAATCATTTCCATCCCTTGAATTTGTTAATTGTTGTGAAAAGCTCCAGAAATTGTGGTTAGATGGGAGAATAGAGAAACTGCCTCTGTTTCCAAATTCCATCACAATGATGGTTCTTGAGAACTCAGAACTGACAGAAGATCCGATGCCTATTTTGGGAATGTTACCTAACCTAAGGAATCTTGAATTAGATGAAGCTTATAAAGGAAAAGAAATAATGTGCAGTGATAACAGCTTCAGTCAACTAGAGTTTTTTAGTGTTTATGATCTTGAGAACCTAGAAACATGGCATTTAGGCACAAGTGCCATGCCTCTCATTAAGGGTCTTCGTATCTGTCGTTGTCAAAAGCTGAAGGAGATTCCAGTGAGAATGAAAGACGTGAAGTGTATTTGA

>PGSC0003DMG400024234

ATGGCTGATGCCTTTCTGTCATTTGCACTTCGAAAATTGGGTGATTTCCTCAGTCAACAAGTTTCCCTGCTTACAAGTCTCAGAGATGAAATCAAATGGCTGAGAAATGAGCTACTCTTCATACAACCTTTCCTCAAAGATGCAGAACTAAAGCAATGTGGAGATCAAAGAGTTGTACAATGGGTGTTTGAGATCAACTCTATTGCTAATGATGTTGTTGCTATACTCGAGACTTACACCTTTGAGGCTGATAAACGTGCTAGTTGTCTCAAGGCTTGTGCTTGCATATGTAGGAAGGAGAAGAAATTATACAATGTCGCCAAGGATATCAAATCACTCAAGAAACGAATCATGGATATCTCTCGCAAACGAGAGACTTATGGTATTACAAATATCAATAGTAATGGCTGGAGAAGGGCCAAGGGAGAACTACCTCATATATGGATGACCAGGATTACATTTTTTGTTGGCTTTCAGGATGCTGTACAAACATTGCTAGCTGAACTTCTCAAAGCAGAGCCTCGTCGAAGCGTCCTCTCCATTTATGGAATGGGGGGTTTAGGCAAGACCACTCTTGCGAGAAAACTTTACATTAGTCCTAATATAGCCTCTAGTTTCCTTACACGCGCTTGGATATGTGTTTCGCAAGAGTACAACACCATGGATCTTCTTAGGAATATCATAAAATCCATCCACGATCGCACCAAGGAATCTCTAGATTTGTTGGAAAGGATGACAGAAGGAGATCTAGAAATTTATGTTCGTGATCTATTAAAAGAACGCAAATACCTTGTGGTGGTTGATGATGTATGGCAGAAAGAAGCATGGGAGAGTTTGAAAAGAGCATTCCCGGATAGCAAGAATGGTAGCAGAGTCATTATTACCACGCGCAAAGAGGATGTCGCTGAAAGAGCAGACGACAGAGGTTTTGCTCATAAACTTCGTTTCCTAAGCCAAGAAGAAAGTTGGGATCTCTTTCGTAGGAAACTACTTGATGTTCGAGCAATGGTTCCAAAATGGAAAGTCAGGCTTACCTCTTGCAATTGTTGTATTGAGCAGACTACTTCGCATAAAAAGGGGCTAAACGAATGGCAAAAGGTGAAAGATCACCTTTGGAAGAACATTAAAGAAGATAAATCTATTGAAATCTCTAACATACTATCATTAAGCTACAATGATTTGCCAGCTGCGCTCAAGCAGTGTTTTCTTTACTTTGGTATTTTTCCAGAAGATCAAGTGTTCGAGGCTGATAACATAATACGATTGTGGATGGCCGAGGGTTTCATACCCAGAGGAGAAGAAAGAATGGAGGATACCGCTGAAGGCTTCTTGAATGAGCTGATAAGACGAAGCTTGGTTCAAGTGGCTAAAACATTTTGGGAAAGAGTTACTGAATGTAAGGTTCATGATTTACTCCATGATCTTGTGATACAAAAGGCATTGGAGGTGAACTTCTTTGACATTTATGATCCAAAAAGCCACTCTATATCCTCTTTATGTATCAAACATGTCATTCATAGTCAAGGAGAAAGGTACCCCTCACTTGATCTTTCTAACTTAAAGTTGAGGTCACTTATGTCCTTCGATCCAGATTTTCGTAAGATGAGTCTTATAAACTTCAGGAGTGTGTTCCAACATCTATATGTGTTGTACTTGGAGATGCGAGTTGACAATATGTCTATTGTATTAGTACCTGATGCCATAGGAAGTTTGTACCACCTCAAGTTCTTAAGCTTGAGAGGTATCGATGACCTTCCGTCTTCCATTGGCAACCTCAAGAATCTACAGACACTTGTTGTCAATGAGGGCAAATACACTTGCCAACTACCCCGCGAGACAGCTGACCTAATAAATTTAAGACATTTAGTTGCTCGGTATTCAAAACCTCTGGTACATATAAGCAAACTCACTAGTCTTCAAGTTGTTGATGGCATCCATTGTGATCAATGGAAAGATGTTGACCCTGTTGATTTAGTCAATCTTGGAGAATTAAGCATGCATCATATTAAGAAATCTTACTCCCTAAACAACATTAGCAGCTTGAAAAACCTTAGCATTCTCACATTGTTTTGTGAGTGTTATAAAACATTCCCATCCCTTGAATTTGTTAATTGTTGTGAAAAGCTCCAGAAATTGAGGTTAAAAGGGGTGATAGAGAAACTGCCTGATCTGTTTCCAAATTCCATCACAATGATGGTTTTGAGGGACTCAAGATTGACAGAAGATCCGATGCCTATTTTGGGAACGTTGCCAAACCTAAGAAATCTCGAATTGATTATAGCTTATGAAGGAAAAGAAATGATGTGCAGAGATAACAGCTTCAGTCAACTAGACTTCCTTATTCTTAATGATCTTTACTACCTAGAAAGATGGCATTTAGCCACAAATTCCATGCCTCTGATTAAAGGTTTTGCTATTTATGATTGTCCAAACTTGAAGAAGATTCCAGAGAGATTGAAAGACGTGAAGCAAAGATAA

>PGSC0003DMG400002217

ATGAAGGAGGAAGAGAGAAAGGGGAAGCAAACAACTCAGTGGGAGTCATTTTCTGCTCTTTACAAGGATGTTGTCAACGTTCTGGATTTCATTGAGAGGTTAAAGAATGAAGAAGATAAAAAGACTGTTGAAATGGTTGATCAAATTGAAAATCTGAAATTTGGGATGGCATTTATTTGTACGTACATCCAGCTTTCTTATTCCGATTTGGAGCAATTTGATGAATTAATGTCTATCCAAAGACAAGAGGTTGAAAATATTATTCGATCAATTCTCTGTGATGTTGAGTTTAACGTGGCAATTAAATACAACATACATCATGTCCTTACTCGCCTCAGGGATAATATTGATTATTTTATCAGCTCTCAGAATCGTTCTACATCAAGTGCCACCATGACTGAGGAGCAGATGGACTTCCTCCTCCTGAATCTCCATCACCTATCCAAGTATCTTACTGAACAGACTCAGTATGAGATTCTTCACAATGTGTGTGGCAACATGAAAGATTTCCATGGTTTGATAGTGAATGGGTGCATTGAGCACGAGATTGTTGAATATGTCTTACCTCAGTTTCAACTCATGGCTGAGAGAGTAGGACGCTTCCTTTGGGATGATCAAATTGATGGAGAGTCTCGACACTTCAAGCTAGCACATCTACTCATGAAGATTATTCCAATTGAGCTGGAGGTTGTGAACATATGTTTTACAAATTTGAAAGCTTCAACTTCAGCAAAAGTTGGACACTTCATTAAGAAACTCCTGGAAATCTTTCCTGACATTCTTAGAGAATATCTGATTCATCTACAGGAGCACATGATAAATGTTACTACCACCACTACTTCAGGGGCTCAAAACATTCATGTCATGATAGAGTTCCTATTAATTATTCTTTCTGATATGCACAGGGACTTTATTCATCATGACAAATTATTTGATCTTTTGGCTCGTGTTGGAGAACTTACCAGGGAGGTATCAACTCTTGTTCGCGACTTAGAAGAAAAATTAAGGAATAAAGAGAGTACTGACAAAACAAATTGTGCAACCCTAGACTTGCTGGAAAATATTGAACTCCTCAAGGAAGATCTCAAACATGTTTATCTAACAGTCCCGGACTCATCTCAATGTTGCTTCCCCATGAGTGATGGACCTCTCTTCATGCATCTGCTACACATGCACTTAGATGATTTGCTGAATTCCAATGCTTATTCAATTGCTTTAATAAAGGAAGAAATCAGGTTGGTGAAAGAAGACCTAGAATCCATAAGATCTTTTTTTGTGAATATTGAGCAGGAATGGTACAAAGATCTCTGGGCACGTGTTTTAGATGTGGCATATGAGGCAAAAGATGTCATTGATTCAATAATTGTTCGAGATAATGGTCTCTTACATCTTATTTTCTCACTTCCCATTACCATAATGAAGATCAAGCTTACCAAAGAACAGGTCTCCAATTTACATGAGAAGATTCCCAAGAACAGAGGTCTCCTTGTTGTAAACTCTCCCAAGAAGCTAGTTGAAAGCAAGTCATTACCGGCTGGTAAAATTATTGTAGGTTTTGAGGAGGAGACAATTTGGATAATTGAGAAGCTCACCCGTGGATCGGCAAATCTAGATGTCATTTCGATCACTGGCATGCCGGGTTCAGGTAAAACTACTTTGGCGTACAAAGTATACAATGATAAATCAATTTCTAGTCATTTTGACCTTCGCGCATGGTGCACAGTCAACCAAGAATATGATGAGAAAAATTTGTTGAATAAAATTTTTAATCAAGTTAATGGCTCGGATTCAAAATTGAGTGAGAATATTGATGTTGCTGATAAGCTACGGAAACAACTCTATGGAAAGAGGTATCTTATTGTTTTAGATGACGTGTGGGATACCTCAAAATGGGATGAGTTAACAAGACCTTTTCCAGAAGTTGAGAAAGGAAGTAGAATTATATTGACGACTCGAGAAAAGAAAGTGGCTTTGCATGGAAAGCGCAACACTGATCTTCTTGACCTTCGATTGCTAAAACCAGAAGAAAGTTGGGAGTTATTAGAGAAAAGGGCATTTGGAAACAAGAGCTGCCCTGATGAACTATTGGATGTTGGAATAGAAATAGCCCAAAATTGTAAAGGGCTTCCTTTGGTGGCTGATCTGATTGCTGGAGTCATTGCAGGGAGGGAAAAGAAAAAGGCAGTGTGGCTTGAAGTTCGAAATAATTTGAATTCCTTTATTTTGAACAGTGAAGTGGAAGTGATGAAGGTTATAGAACTAAGTTATGACCATTTATCAGATCAAGTAAAATTGTGCTTGCTTTACATTGCAAGTTATTCGAAGGACACTGGAATGATAATTTCTGGGTTGATAGATTTATGGCATGCTGAAGGACTTGTGGAGCAGACAGAGATGAAGAGTGAGGAAGAAGTGATGAAGGTTTATTTGGATAACTTAATTTCCAGTAGCTTGGTAATTATTTTCAATGAGATTGGTGAGTACCCGACTTGCCAACTTCATGACCTTGTGCATGACTTTTGTTTGATAAAAGCAAGAAAGGAAATGTTGTTTGACCGGATAAGTTCAAGTGCCCCATCTTCTTCTTCAGATTTGATGCCACGTATAGTGACCATTGATTGTAAAAGCAAGTTCTTTGGCCTTGAAAATTTTGTCCTGTTCGGTTCAAATAAGAAAAGGCATTCTGGTAAACACCTGTATTCTTTGAGGATAAATGCAGATAATCTTGACGGCGGTCTTTCTGATACATGTCACCTAAGACACTTGAGGCTTCTTAGAGTGTTGAAACTGGATCACACTTTTAAGGTGAAAGATTCTTTGCTGAATGAATTATGCATGTTGGTTCATTTGAGGTTCTTACGAATTGGGACAGAAGTTAAATCTCTGCCTTCATCTTTCTCAAACCTCCGGAATCTAGAAATCCTCTCGGTGTATAACAACGGATCAACCTTGGTACTATTACCGAGAATTTGGGATCTAGTAAAGTTGCGAATACTGCACATGACTTCTTGTTCTTTCCTTGATATGGATACAGATGAACCAATTCTGATAGCAGAGGACACAAAGTTAGAGAACTTGAGATATTTAGAGAACCTGGTGCTTTCCTATTCGAAAGATGCAGAGGATATTTTCAAAAGGTTTCCCAATCTTCGAATTCTTGTATTCGTTCTCAAGGAATCACGGGATTGTTCAACAAAACGACAGTGGTTCCCAAAATTGGATTTCCTAACTGATCTAGAACGCCTCAGTGTAGAATTTGAAAGTTCAAATGACAGTGAGCCCTCTGTAGGGACAAATTGGCTGTGTAATTTTCACTTCCCTTCAAGTTTGAAAATATTGTCATTAGGTAAATTTCCTCTCTCACCCGATTCATTATCAACATTAGCTAGACTGTCCAACCTTGAAGGGTTGTCCCTTGAGAACACAATCATTCACGGGGGAGAATGGAACATGACGGAGGAAGACACCTTTGAGAATCTCAAATTTTTGGAGTTGGATGAAGTGACACTTTTTAAGTGGGAGATTGGAGAGGAATCATTTCCCGTGCTTGAGAAATTAGAACTGTGGAGATGTCGTATGCTTGAGGAGATTCCGCCAAGTTTTGGGGATATTTGTTCATTGAAAATTATCAAACTTGTAGAGAGCCCTCAACTTATAGATTCTGCTATGAAGATTAAGGAATATGTTGAAGATATCATGGGAGGGGACGAGCTTCAGGTTGTTGGCCGGAACAATGTCCCATTATTTTAA

>PGSC0003DMG401016933

ATGGAATTCTTATCTATTTTTGTGAAAATGATCACAGATTGCTTGATCCAGCCAGTTGCGCGAGGGATTGGGTATTTCTATTACTACAAGAGCAACATCACATCTCTGGATGAAGAATCTCAGAAGCTGGAGAATATCAGACACGGCGTGGAGGAAAGAGCAGAGGCTGCCCGGAGAAACTTACAAGTCATTTCACCCAATGTTGAGGCTTGGTTAACTAGTGTTGATATCACTACTGCAGATGTGGCAGCTGTGATGCAACGAGGTAGAATTGAGGTTGAAAGATATGGTTGGTGTCCAAACTTGAAATCACGTTACTCGCTGAGCAAGAGAGCCAAGAGAATTACCCTGGAGATGATTGAACTTCGAAATGAAGGTAACAAGCATGATGTTTTCTGCTATCCTGTCGTAGAAATTGAAGCTATTTCTAGTAACAGTACTGAAGAATTTGACTCCAGAAAATTGCAAGAGGAAGAGGTCATGGCAGCTTTGAGAGATGATGGGGTCAATATGATTGGGATATGTGGTATGGGTGGTGTTGGTAAGACAACACTGGCTGAGAAAATAAGAGCAAGGGCGAAACAAGAAAGGTTGTTTGATGATGTTGTCATGGTAACTGTCAGTCAACAACCAGACTTCAAAAGAATTCAGGGTGAGATCGCAAGAGAAGTCGGGCTGACATTAGAAGGGGATAATTTGTGGAGTCGTGGAGATCGGCTGCGTTCAAGGTTAAAGGATCAGAACAGCCGCGTTCTTATAATACTGGATGATGTTTGGGAGGCTCTTCATGATCTAGAGAAACTTGGAATTCCAAGGGGTAGCAACCACAACCATCGGTGCAAAGTGACATTGACTACGCGTCTGAGAGATGTTTGTGAAGCTATGGAAGCTCAGAAGATCATGGAAGTTGGAACCTTACCTGAAAATGAAGCATGGATTCTTTTTAGGCAGAAAGCCAGTAATTTAGTTGACAATCCTTCTCTCCTTGACATAGCAAAAGATGTTGCCAAAGAATGCAAGGGGTTGCCACTTGCAATTATCACAGTTGCAGGAGCACTAAAGCATAAAACCAAGCCTTCATGGGAGGATGCCCTTAAACAATTACGTGATGCAAAAACAAGAAATATCCCTGGAGTGCACACAAAGGTGTATAAAATTCTGAGGCTAAGCTATGATCACTTGGAAAGTGATGAAGCCAGGTACCTCTTTTTGCTTTGTTCTTTGTTCGAGGAAGATAGTGATATTTGGACTGAAGAATTACTTAGGTATGTAATGAGGCTTGACATCTTTTCGGAAATCGAAAATTTAGAACATGCAAGAAATAGAGTGTGTCTTCTGTTAGAAACACTGAAAGGTTGCTTCTTGTTATCCCAAGGTTCGGACAAAAATTATGTCAAAATGCATGATGTGGTCCGTGATGTGGCTATATATATTGCGTCTGAAGGAGAGCATATCTTTATGGTAAGTCATAATGTGAACTCAAAAGAGTTCCCAAGAAGAATTTCTTATGAGCATTTCAGTCACATGTCAATTGTTGCAAATAAATTTGATGAGCTTCCTAGACCAATCGTTTGCCCAAAACTGAAGCTTCTAATGTTAAAACTCTGTTTTGAAAAGCCATTCAAATTACAGGACGATTTTTTTGATGGCATGAGTAAACTCAATGTTTTAAGCATGAGGGGAGACAGATATAAGGAGTCCATTTGGCCTCTTCCAGGATCCATTCAGAGGTTGTCAAGTCTGAGGACGCTGTGTCTGAGTAAATTAAGGTTGGATGACATATCCGTTATTGGGGAACTTGTCACTTTAGAAATTCTCAGCATCAAAGATTCTCAGTTAGAGGAGTTGCCAGTGGAGATAGGAAAATTAACCAATCTAATTATCTTAGAGCTGCAGAATTATAAGCAAGTAGAACTTGAAAGGATTTCACCAGGTGTCTTATCAAGACTAGTACGATTGGAGGAATTACATATGGTGGGAGTAGAACATTTTAGTTACTCCACCTTAAGAGAGCTGGAATCCTTATCGAGATTGACTGCACTGACATTAAGTAAATGTTCCGGAGATGTGATCTACAATAACTTGGGCCTTTCCTCCGAGTTGACACGGTACGCTCTTACATTGGGTAGAGCTTACAGAACGACTTCAACCATAGATGATTATGACAAGAATATTTCTTTAGAGGTCACTGAGACCACCCCATTGGGTGATTGGATCTGCCACAAGCTTAGGAAGAGTGAACTTGTACATTCAACTGGAGAGGGCTCCAAGAATGTGCTGACAGAGTTGCAGCTGGATGAATTTCAGAATGTGAAATATCTTCTCCTGGATGATTGTGATTCATTGACACATTTATTAAAGATCCACTGTCAGAATAATATTCCATTCCCTGAACTCGAAAGACTTGAGGTAAGCCGTTGTCGTGGTCTACAGTATGTTTTTTGTGTGCCCTTGGCTGGAGGGAGTTGGACAGTAGTTTGCCCTAATGATGAGGAAGAGGAGATCTCTCGGAGGACTCGTGAAGTAATCAAGTTCCCCAATTTATATGAATTGGACCTTCATTCTCTTGAATGCCTCACTCACTTTTGCAGTGACAGTGTTGAGGGCATTGAGTTCCCTCGGTTACGGGAAATGAGCTTCTTTGAGTTACCAGAGTTTCAAAATTTCTTGCCTACAACCAACAACTCAATCACTCACTCAAATCCTCTTTTTGATGAAAAGGTCTTCTTCTACTGCTCTATGTTTATAAATTTACAGGTTTCTTGTCTCAGTCTGGAAGAGCTATCTATCGATGGAGCTAATAGCATAAGTGCTCTATGCTCTCACCAACTTCCAACAACCTACTTCAGCAAACTTGAATCATTGTATGTATCAAATTGTGGAAAATTGAGAAACATGATGTCTCCATCAGTGGCTAGAGGTGTTTTCAATCTCCGAATACTAAAGATAGATGGTTGTCAATCGATGGAAGAAGTGATCACAGAAGAGGAACAACAAGGAGAAGAAATCATGACTAATGAGCCCTTATTTCCCCTGTTGCAAGAATTGAGGCTTCAAAGTTTGCCATGGCTTGGGCATTTCTTTGTGACGAAGCATGCTCTTGAATTTCCATTTCTCAGAGAAGTGACTATTCATGACTGCCGTGAAATGGAGACGTTTGTCCAACAAGGATTTGTGAGTCTCGAAAGGGTGAACAATGACGATGAGGTGAACAATAAAGTGATGTTCAATTCTAAGGTTTGTCTTGTGCCTCTAGTTGTACTCTCGCTCCACGTTCCAGTTGGGCCTGGATGTGAAGGGGATGTTAGAGTCCCACGTTGGTTGGAAAATGGAATGGTGGTCTGCATATGTGTACTTGGTCAATCCTCCCCTCTTGAGCTAGCTTTTGGGGTTGATTTAGGTCATATCTTTACAATTTTCATATCAAACTCTCCAATACTGTCTTTACTGCCTTTCTTCATCCCTTCAATCGTACGTTTGCAGGTTTCTTTTCCCAGCCTGGAAGAGCTATATATCAATGGCGCTAACAGCATAAGTGATCTATGCTCTTACCAACTTCCAACTGCCCACTTCAGCAAACTTGAAATATTGAATGTAAAGGAGTGTGCTAAATTGAGAAACTTGATGTCTCCATCAGTGGCCAGAGGTGTTTTGAATCTCCGAATACTAGAGATAAATGACTGCCAATCAATGGAAGAAGCGATCACAGAAGAGGAACAAGAAGAAGAAGAAATCATTACTAATGAGCCCTTATTTCCCCTGTTGGAAGAGCTGAAGCTTCAAAGGCTACCAAAGCTGAGGCATTTCTTTCTCGCCAAGCGTGCTCTTGAATTTCCATTTCTCAGAGTAGTGTGTATTCATGACTGCCCTGAAATGAAGACTTTTGTCCAACAGGGATCAGTGAGTACCCCAAGTCTCGAAAGTGTAAACAATGATGATGAGGTGAAAGTAGTTGATCTCAATAAAGTGATGTTCAATTCTAAGGTTTGTCTCGTGCCGCTACTATATAGCGCTATTTTGAATTTTCATCCCTTACATAAAATACAAGTTGTCCTTCATCATTTGCAGGTTTCTTGTCCCAGCCTGGAAGAGCTAGAACTCGACAGGGCTGAAAGCATAAGTGCTCTATGCTCTCACAAACTTCCAACTGCCTACCTTAGCAAACTTGCAAAATTGTATGTATCGAATTGTGCTAAATTGAGAAATTTGATGTCTCCGTCAGTGGCCAGAGGTGCTTTGAATCTCCGAATACTAGAGATAAAAGACTGCCAATCAATGGAAGAAGTGATCACAGAAGAGGAACAACAAGGAGAAGAAATGACTAACGAGTTTTTATTTCCACTGTTGGAAGATCTGGAGCTTAAAGGGCTGCCTAAGCTGGGGAATTTCTTTCTGACGAAACATGCTCTTGAATTTCCATTTCTAAGAGTAGTGAGGATTCATGACTGCCCTGAAATGATGACGTTTGTCCAACAGGGATCTTTGAGTACACCGTGTCTCAAAAGGGTGAACAATGACAATGAGGTGAAAGTAGATGATCTCAATAGAGCGATGTTCAATTCTAAGGTTTGTCTTGTGCCGCTAGCAGTATTATTGATTACTATATATATTTGTGTCATGCATTAA

>PGSC0003DMG400010527

ATGGATGGATTGGCTGAAACAGGATCATCTTCTTCTTCTTCTTCTTCTTTGTGGCCATGCACTTATGATGTTTTCTTAAGTTTTAGAGGAGAGGATGTACGGAAGAATTTCGTCGATCATCTATATACAGCTTTGCAGCAAAGAGGAATTCACACTTTTAAAGATGATGAAAAACTTGAAAGAGGGAAATCTATTTCACCTTCACTTTTCAAAGCCATCGAAGAGTCGATGATTTCCATCATCATATTCTCTCAAAACTATGCTTCTTCTTCGTGGTGTCTAGATGAGCTAGTTAAGATCACTCAATGCATGAAACTCAGGGGACAGATTGTTCTTCCTGTCTTCTATGACGTGGATCCATCTGTCGTAAGAAAACAAAAGGCAAACGTTGGTGAATTCTTCGCTAAACATGAGTTAGATTTCAAAGATGATGAAGAAAGGGTGAAGAGATGGCGTACTGCTATGACAGAAGCAGCAAATGTATCTGGTTGGGATTTGCCAAATATTGCTAACGGGTAAATTTATGTGTACTTTTCACTCTTGGTTTTCCGTCTTCCATCTGTGTGTTGTTTTCCATAAGAACATCGAGTTTGTAACTCAATTCTTTCTGATATCAGGCACGAATCAAAATGTATCGAGCAAATTGTAGAATGTGTCATGGAGATATTAGATCATTCTGCTTCTGATGCTACTGAAAATCTTGTTGGAATACGCTCAAGAATGGGGACGGTGTATTCCTTGTTGAATCTGGAGTCTGATAAAGTTCAATTCGTTGGAATATGGGGAATGAGCGGAATTGGGAAAACAACTATAGCAAGAGCCATCTACGACAAGATTTTCCGTTACTTTCAAGGTACTACTTTCCTTCATGAAGTTGGAGAAAATTCAGCCAAACATGGTATCCAACATTTGCAGCAGATACTTCTTTCTGAACTACTTCTGTTAAAAGATCTAAGAATAAACAACGTATTTGAAGGAACCAGCTTGGTAAGAAGACGACTAAATGGGAAACGAGTCCTAATTGTTCTTGATGATGTCAATCATGGAAACCAGTTAGATGCCCTAGCTAAAAGCCATGACTGGTTTGGTGCAGGCAGTATAATCATCATAACAACAAAGGATAAGCAGTTGCTTCGTCAATATAACGTGGACAAAATGTATAAAGTGAGTCTGTTAAACACTGATGAAAGTATTGAACTCCTTAGTTCATATGCATTCCAGAAACACCATCCCAAAAGTGGATATGAAGAGATCATAGCTGAAGTTGTTCGGTATGCTGGTGGTCTTCCATTAGCTCTTAAAGTTTTGGGTTCCTCTCTGTATGGCAGAGGCATGATTGAATGGAGAGAAACAGTGGAGAGACTAAAACAAATTCCAGAAGGCGAAATTGTAGAAAAACTCAAAGTAAGTTTCAATGGACTAAGTGAGATTGACCAAAAGATCTTCTTAGATATTGCATGTTTCTTTAAAGGGAAGAAGAAAGGTTCTGTAATTAGAATTCTTCGTAGTTTCAGTTTTACTCCTGTCATTGGCATAAGAAATCTCATCGAAAAATCTCTTGTAACTGTTTCAAAAGGTCGGATTGTGATGCATCAGTTGATCCAAGAGATGGGTTGGTATATTGTTCGCAAAGAAGCTTCAAACAATCTTGGCAAGTATACTAGGCTCTGGTCTCCCGATGATATTCTTCATGTACTATCTGAAAATGGCACAGAAGCTGTGGAAGGCATATGGTTGCACTTGCCTATTCCGAAAGACATAAATGTTGGTGCAGAAGCCTTCAAATATACGGACAACCTGAGGCTGCTCAAGATGCACAATGCAAGTGTCTCCGTAGCTCCAGATTGTCTTCCTAATAAATTGATATGGCTTCATTGGCATGGCTACCCAATGAAGTCCCTTCCAGCAGGTTTTCGAGCAGAAAGGCTTGTTTGTCTGAAAATGCAGTATAGCCGCGTTGTACACTTGTGGAAGGGAATAAAGGTCCTACACAAACTGAAGTTTCTCAACCTTAGTCACTCCCAAAAGCTAGTCAGCTGTCCAGATTTCACGGGGGTGCCCAATCTCGAGAAGTTGGTTCTTGAAGATTGTTCGAGTATAATTGAGATCCACCCTTCTGTCGGATATCTCAAAAATCTTGTTCTACTAAACCTGAAGAACTGCAGGAATCTTAAGAGCCTTCCAAACAATATTCGATTGGATAATCTTGAGACTTTAATTCTTTCTGGCTGCTTGAAACTCGCGAATTTCCCAGAAATCACGAGTGACATGAATTGCTTATCTGAGGTCTACTTGGAAGCTACAGATGTAAAAGAGTTGCCTTCATCAATTGAACGTCTCACTGGCCTTCAATTGATGAATCTAGGCTACTGCAGGAATCTTACAAATTTACCAAAAACCATAGGCAGATTAAAATCTCTTAGGATTCTTATTCTCTCTGGATGTTCAAAACTAGAAAAGTTGCCAGAGGAACTAGGACATATAGCAATCTTGGAGGAACTCTATTGTGACGAAACTGCCATTCGAAGCCCACCATCCTCAATTACACTATTGAAGAACCTTAAGATCTTATCTTTTCATGGATGTAAAGGCATGGTATCTCAGACATGGAATTCACTTTTCTTGGCATGGCTTCGGCCAAGAAAACATAATCAAAAGCCAACTCCTTTTCCAGTTTTATTTTCTTTGAGGAAATTGGATCTTAGTGATTGTTGTATGTTGGATGAAGGAATTCCTAGTGATGCTAATCCTAGTGGGAATAATTTTGTGGATATCTCTCAAGCAAGTCTCAACATGCTTCCGCGGCTCAGAATCCTTGAGCTAGTGAGTTGTGAGAGGCTTGAAAGGTTGCCAGAACTTCCAACAACAATAGAGGAAGTTTTTGCAGATAATTGTACATCCCTGATGACTGATAATGTAGGAATATTGACCAACTACAAGATGTTGCAGCGAATATCGTTCACTAATTGTGTTGGACTACTTGAGAATCAGCAGATGCATGACATGGCTACCTCATTGTGGCTTCACCTATTCAAGGTCTCCTTACGTCTTCATCCTTATGTATTGTTATCTTCGTACCCTTTACGTTCATATTCGAGATCAGACAAT

>PGSC0003DMG400043376

ATGGCGATTCCATTTCTGAAGAACTCGGCTATAGAAGAGTCTCAAGTTGTCATAGTCGTTTTCTCAAAGAATTATGCTACATCAAGGTGGTGCTTAAATGAATTAGTGAATATCATGAAATGCAAGGAGGAGGAAAATGGACAAATAGTCATACCGGTCTTCTATGATGTGGATCCATCACATGTACGATACCAAAGAGAGAGCTTTGCAGAAGCATTTGCCAAACACGAATCGACGTATAAGGATGAGGGTGATGAGGGGATGCAAAAGGTGCAAAGATGGAGGAATGCCTTAACTGCTGCCGCAGATCTAAAAGGATATGATATCCGTGACGGGAAACTAAAATCCTTACTTCATATAGAAATCAATGATGTTCGGATTGTAGGGATCTGGGGAGTAAGTGGAGTTGGTAAAAAGACAACAGCAAAAGCCATCTTTGATACGGGAGGACATAGCCGACTTTGGGACGTTGAAGATTTTGAAGAAGTGATGGTTAACAATACAGTAAGTAGGCTAGATAATGCAATAATATTCAATTTTTAA

>PGSC0003DMG401010614

ATGGTCGATGCAGCTGTGTCCTATGCAGATGAAAAACTGGGGGATTTCCTCATAGAGGAAGTTTCCCTGCGCCAAAGTCTGAGAGAGAACGTTCTTTGGCTGAGAAATGAACTGTCTTTCATGCAAGCTTTCCTCAAAGATGCCCAAAAAAAGCAAGAACAAGATAACCTCGTGCAGCAATGGATATTTGAAATCACGTCCGTTGCTAATGAAGCTGTGTACATCTTAGAAGCATACAGTTTGGATGCGGCAAAAGATGGTGATTATGCTGTTGGATTTGTTGATCGTCTCAAGGCTTGTTCTTGCATCCATCAGAAAGAGGCCAAATTCCACAACATTGGGAAGGAAATCCAGTCCCTCAAGGAAAGAGTCATGGATATCTCTCGAAAACGAGATACCTATGGTATTACTTATATGAATATTAATGCTAGAGAAGGGCCAAGTAATAGGCCAAATGATCCGTCTTCCTCATTAATAAGAACATTGAGGAGAGCAGTGTCCTATGCAGAGAAAGATCAACTCTTTGTTGGCTTTCAAGAGGTCTTTCAAAGACTACTTGATGAAATTCTCAAAAAGTAGTCTCGTCGAAATGTCCTTTCAATTTATGGTATGGGTGGGTTGGGAAAGACCACTCTCGCAAGAAACCTATATAACTCCCCAGGTCTACTAGCTATCTTTCAAACTCGTGCTTGGATATGCGTCTCTCAACAGTATAGTATCCTTCGGAGTATCATAAAATCTATCCAAGGTTACCATGAAAAAATGCTAAAATTGCTGAAGGAGATGACGGAGACAGATCTAGAAACTCACCTCCGTAATCTACTGAAAGAGCGAAAATACCTTGTGGTGGTTGATGATGTATGGCATCGAGAAGCTTGGGAGAGCCTAAAAAGAGCCTTACCGGATAACAACAATGGCAGTAGAGTTATCCTTACAACACGAAAGGAGGACGTGGCTGAAAGAGTTGATGACAAAGGTTTCTCGCATAAACTTCGTTTTCTAAATAAAGAAGAAAGTTGGGACCTTTTATGCAAGAAACTACATCCAGAGAATAAGATGTCTAGTGCTGACTTGTTTTCCCCATCAATGAAAAGGCTAGCTACGGAAATGGTGGAGAAGTGCAGAGGCCTACCACTTGCCATTGTGGTACTAGGTGGGCTACTTTCCTACAGAAAGGGGGTGGATGAATGGCAAAAGGTGAAGACGCATCTTTGGCAGCATATGAAGAACGACTCTGTTGAGATCTCTCACATCCTATCATTGAGCTACAATGATTTGTCATTTGAGCTCAAGCAATGCTTTCTATACATTGGCAGTTTCCAAGAAGATCATGTGATTGACGCTGAAAAGTTGATGCGCCTGTGGCTGGCAGAGGGATTCATACCAAGAATTTGAGAACATATGGAGGTTATTGCTGAAAACTTCCTACATGAGCTAATAAGTCGAAGCTTGATTCAAGTTGCGGAGACATTCTTTGATAAAATTCTTACATGTAGGATACACGACCTACTACGGGATCTTGCTGTACAAAAAGCCATGGAGGTTAACTTGTTTGACATTTATGATCCAAGAGTAAACTTGGTCACCCCATTTCGTCACCGACATGCTATTCACACACAAACTCAAAGGTATCTTTCACTTGATCTTTCTAAATTGAAGGTAAGGTCAATATTGCTCCTTGACAAAAAATTTAAGCACTTGGATGATAAAAAGTTTATAAAGTTATGTACAACGTTCCTGCATTTATATGTGCTGGACTTGCAGAATATCTACCTGATGCTAATAGGCAATTTGGTACACCTCAAGTTCTTAGGCTTGTCTAACACCAATCTTTTCAAACTCCCATCTTCCATTGGCAAACAATAAAACCTACAAACGCTGGAAGCACTGATTGATGACCATTGCTCATGCCAATTACCTCCTCAGATAGCCAAGCTCACAAATTTGAGACATTTAATTACTCGATATGAGGTACCGTTGCAAGTTGACAGGCTACGAACTCTTAAATATATTCGTTGTGATCAGTGGAAACATACTGATGCTTCTCGTTTAGTCAACCTTCAAGAATTGGGCATGGAACATATTATGAAATCTTACTCCCTAAAATCCATTGGGAGCCTAAAAACCCTCATCACCTTGTTGCTAGTTTGCAGTTATGGTGAAACTTTTCCTCCCCTTGAACCTCTTTCTTCTTGTGAAAACCTTCAGAGATTGTGGTTATCAGGGGGAATAGAAAAATTAGCAAATTTAAACAATCTGCCAAAATCCATCACTGTGTTAGTCCTACAGTCTACACGCTCTACAGGACTCGAGGAAGATCCAATGCCAATTCTGGGGAAGTTTCCAAACTTAAGGCATCTCGAGTTATCACGGGCCTACAAGGGAAAGAAAATTACTTGTAACAGCAACAGTTTTAGTCAGCTGGAGACCCTTAGACTTGTTAATCTTGGAAATCTTGAAAGCTGGCATCTTCACACAACTGCTATGAGTGTGCTAAAAAGTTTGAGTATATTTCGGTGTCCGAAGCTGCAGAAAATTCCAGAAAGAATGGAACATATTGCGGTGCTTGATGGTAATCAAAGCAAACTGGTGTACTATTATCTGTTCCCACATTAA

>PGSC0003DMG400010613

ATGGTTGATGCAGTTGTGTCCTATGCAGTTCAAAAACTTGGGGATTTCCTCATAGAGGAAGTTTCCCTGCGCCAAAGTCTGAGAGAGAACGTTCTTTGGCTGAGAAATGAACTGTCTTTCATGCAAGCTTTCCTCAAAGATGCCCAAAAAAAGCAAGAACAAGATAACCTCGTGCAGCAATGGATATTTGAAATCACGTCCGTTGCTAATGAAGCTGTGTACATCTTAGAAGCATACAGTTTGGATGCGGCAAAAGATGGTGATTATGCTGTTGGATTTGTTGATCGTCTCAAGGCTTGTTCTTGCATCCATCAGAAAGAGGCCAAATTCCACAACATTGGGAAGGAAATCCAGTCCCTCAAGGAAAGAGTCATGGATATCTCTCGAAAACGAGATACCTATGGTATTACTTATATGAATATTAATGCTAGAGAAGGGCCAAGTAATAGGCCAAATGATCCGTCTTCCTCATTAATAAGAACATTGAGGAGAGCAGTGTCCTATGCAGAGAAAGATCAACTCTTTGTTGGCTTTCAAGAGGTCTTTCAAAGACTACTTGATGAAATTCTCAAAAAGTCTCGTCGAAATGTCCTTTCAATTTATGGTATGGGTGGGTTGGGAAAGACCACTCTCGCAAGAAACCTATATAACTCCCCAGGTCTACTAGCTATCTTTCAAACTCGTGCTTGGATATGCGTCTCTCAACAGTATAGTACCCCAGATCTCCTTCGGAGTATCATAAAATCTATCCATGGTTGCAGTGAAGAACTGCTCAAATTGCTGAAGGAGATGTCAGAGAGAGATCTAGAAACTCACCTCCGTGATCTATTGAAAGAGCGCAAATACCTTGTCGTGGTTGATGATCTATGGCATCGAGAAGCTTGGGAGAGCCTGAAAAGAGCCTTACCGGATAACAACAATGGAAGTAGAGTTATCCTTACAACACGAAAAGAGGATGTGGCTGAAAGAGTCGATGACAAAGGTTTCTCCCATAAACTTCGTTTTCTAAATAAAGAAGAAAGTTGGGACCTTTTATGCAAGAAACTATGTCCAGAGAATAAGATGGGTGGTATTGACTTGTTCTCTCCTTTAATGAAAAAGCTAGCTGAGGAAATGGTGGACAAGTGCAAAGGCTTACCACTTGCAATTGTGGTACTAGGTGGGCTACTTTCCCACAAAAGAGGGGTGGACCAATGGCAAAAGGTGAAGACACACCTTTGGCAGCATATGAAGAATGACTCTGTTGAGATCACTCACATCCTATCACTGAGCTACAATGATTTGTCATTTGAGCTCAAGCAATGTTTTCTGTACTTTGGCATTTTCCGAGAAGATGAAATGATTGATACAGAAAAGTTGATGCATTTATGGCTGGCAGAGGGATTCATGCCAAGAATGAGAGAAGTACACATGGAGGATATTGCTGAAAACTTCCTTCATGAGCTGATAAGTCGAAGCTTGATTCAAGTGGCCGACACATTCTTCGATAAAATTCTTACATGTAGGATGCATGACCTACTTCGGGATCTTGCTGTACAAAAGGCCATGGAGGTTCACTTGTATGACATTTATGATCCAAGAGTAAATTCGGTCACTCCATTTCGTCACCGACATGCTATTCACGCGCAAACTCAAAGGTATCTGTCACTTGATGTTTCTAAATTGAAAGTGAGGTCAATATTGTTCTTTGATAACGAGTTTAAGAACTTTGACTATAGTAAGAAGTTCATGACGTTCTGTATGTCATTCCCCCATCTATATGTGCTAGACTTGGAGAATATCCTTTTTGATGGGAATGAACTACCTGATGCTATAGGCAATTTGGTACACCTCAAGTTCTTAGGCTTGTCTAATACCAATCTTTTCGAACTCCCACCTTCTATTGGCAAACTAAAAAGCTTACAAACGCTGGAAGCATTGATATTTGACAATTGTTCTTGCAAACTACCTCCTCAGGTAGCCCAGCTCACAAATTTGAGACATTTAGTTTCTCGATATGAGGTTCCTTTGCAAGTTGACAGGCTGACAAATTTACGAACTCTTAAGTACATTCGTTGCGATCAATGGAAAGATACTGATGCTTCTGGTTTAGTCAACCTTCAAGAATTGGGCATGGAAAAGATTAGGAAATCTTACTCCCTAAAATCCATTGGCAGTCTGAAAAGCCTCACCACCTTGTTTCTAGTTTGCCGATATGGTGAAACTTTTCCTCCCCTTGAACCTCTTTCTTCTTGTGAAAACCTTCACAGATTGTGGTTATCAGGGGGAATAGAAGAACTAGCAGATTTAAACAAGCTTCCAAAATCCGTCACTGTGTTAGTCCTAGAGTGTGCACGACTTAAGGAAGATCCAATGCCAATTCTGGGGAAACTTCCAAACTTAAAGCATCTTGAGTTATCATGGGCGTACAAGGGAAAGCAAATTACTTGCAAGGGCAACAGTTTTGGTCAACTGGAGACCCTTACACTTGGTAATCTTGAAAAACTAGAAAGCTGGCATCTTGACACAACTGCTATGAGTGTGATTAAGAGTTTGAGTATATTTGGGTGTCTGAAGCTGAAAAAAATTCCAGAAAGAATGGAACATATTGCGGTGATTGATAGTAATCAAGCCAAAGGGAATACTATGATCGGTTCCCAAATTAACTAG

>PGSC0003DMG400010612

ATGGTTGATGCAGTTGTGTCCTATGCAGTTGAAAAACTGGGAACATTCCTCATAGAGGAAGTTTCCCTGCGCCAAAGTCTGAGAGAGAATGTTCTTTGGCTGAGAAATGAACTGTCTTTCATGAAAGCTTTCCTCAAAGATGCTGAAAAAAATCAAGAACAAGATAACCTGGTGCAGCAATGGGTATTTGAAATCACATCCGTTGCTAATGATGCTGTGTATATCTTAGAAGCATACAGTTTGGATGCGGCAAAAGATGGTGATCATGCTGCTGGATTCGTTGATCGTCTTAAGGCTTATGCTTGCATCTGTCAGAAGGAGGCCAAATTACACGACATTGGGAAGGACATCCAGTCCCTCAAGGAAAGAGTCATGGACATCTCTCGAAAACGAGATACCTATGGTATTGCTCACAACAATAGTAATGCTGGAGAAGGGCCCAGTAATAGGCCAAACTATACGTCTTCCATGTTAACATTGAGGAGAGCAGTGTCCTATGCAGATGAAGATCAACTCTTTGTTGGATTTCAAGAGGTCTTTCAAAGACTGCTTGATGAACTTCTAAAAGAGGAGTCTCACAGAAAAGTCCTTTCAATTTATGGTATGGGTGGGTTGGGCAAGACCACTCTTGCAAGAAACCTATATAACTCCCCAAGTTTAATCACTACCTTTCACACTCGTGCTTGGATATCTGTCTCTCAACAGTATAGTATTCCAGATCTCCTTCGGAGTATCATAAAATCTATTGAAGGCTGCAGTGAAGAATTGCTCAAATTGCTGAAGGAGATGTCAGAGAGAGACCTAGAAACTTACCTCCGTAATCTATTGAAAAAGCACAAATACCTTGTGGTGGTTGATGATGTATGGCATCGAGAAGCTTGGGAGAGCCTGCAAAGAGCCTTACCAGATGACAACAATGGCAGTAGAGTTATCCTTACAACACGAAAGAAGGAAGTGGCTGAAAGAGTCGATGACAAAGGTTTCCCCCATGAACTTCGTTTTCTAAATAAAAAAGAAAGTTGGGACCTTTTATGCAAGAAACTACATCCAGAGAATAAGATGGTTGGTGCTGACTTGTACTCCCCATCAATGGAAAGGCTAGCTAATGAAATGGTGGAGAAGTGCGGAGGATTACCACTTGCTATAGTAGTACTAGGTGGGATACTTTCCTACAGAAAAGGGGTGGACGAATGGCAAAAGGTGAAGACACACCTTTGGCAACATATGAAAAATGACTCTGTTGAGATAACTCACATCCTATCATTGAGCTACAATGATTTGTCATTTGAGCTCAAGCAATGCTTTTTATACATTGGCATTTTCCAAGAAGATCATGTGATCGACACTGAAAAGTTGATGCATCTGTGGCTGGCAGAGGGATTCATACCAAGAATTAGAGAAGAACATATGGAGGATATTGCTGAAAACTTCCTACATGAGCTAATAAGTCGAAGCTTGATTCAAGTGGCCGAGACATTCTTCGATAAATTTTTTGCATGTAAAATACACGACCTACTTCGGGATCTTGCTGTCCAAAAGTCCATGGAGGTTAACTTGTTTGACATTTATGATCCAAGTGTAAGCTCGGTTACTCCATTTCGTCACCGACATGCTATTCATGGACAAACTCAAAAGTATCTTTCACTTGATCTTTCGAAATTGAAGGTAAGGTCAATATTGTTTTTTGATAAAGAATTTGAGAAATTGGATAATAGTGAAAAGTTCATGACATTCTGTACGACGTTCCCACATCTATATGTGCTGGACTTGGAGAACATCTATTTTAGTGAGGGTAAACTACCTGATGCTATAGGCAATTTGGTACACCTGAAGTTCTTAGGCTTGTCTAACACTAATCTTTTCAAACTCCCATCTTCCATTGCCAAACTAGAAAACCTACAAACGCTGGAAGCACTGATTGATGACTATTGCTCATGCCAATTACCTCCTCAGATAGCCAAGCTCACAAATTTGAGACATTTAATTACTCGATATGAGGTTCCCCTGCAAGTTAACAGGCTGACAAATCTACGAACTCTGAAATATATTCGTTGTGATCAGTGGAAAGATACTGATGCTTCTGGTTTAGTCAACCTTCAAGAATTGGGCATGGAACAAATTGCGAAATCTTACTCCCTAATATCCATTGGCAACCTGAAAAGCCTCACTACCTTGTTTCTAATTTGCAATCGTGGTGGAACTTTTCCTCCCCTTGAACCTCTTTCTTCTTGTGAAAACCTTCACAGATTGTGGTTATCAGGGGGAATAGAAGAACTAGCAAATTTAAACAATCTGCCAAAATCCATCACTGTGTTAGTCCTACAGTCTCCATTCTATACAGGACTCGAGGAAGATCCAATGCCAATTCTGGGGAAGTTTCCAAACTTAAAGCATCTCGAGTTATCATGTGCTTACATGGGAAAGAAAATTACTTGCAACGGCAACAGTTTTGGTCAGCTGGAGACCCTTAGACTTGAATATCTTGACAATCTTGAAAGCTGGCATCTTCACACAACTGCTATGAGTGTGCTTAAAAGTTTGAGTATACGTCAGTGTCCGAAGCTGATGAAAATTCCAAAAAGAATGGAACATATTAAAATGCTTGATGGTAATCAAAGCCAACGGGAGTACTATTATCGGTTCCCACATTAA

>PGSC0003DMG400007999

ATGGCGGTGACGGACTTTTTCGTCGGCGAAATCACCACCGAACTCTTAAAAAACCTTCTGCTAATAGTTAAAAAATCCACTTTATGCCGTTCAAGTGCCGAGAATCTCATCGACAGCATCAATGGTCTCCTTCCAATCATCCAAGAAATCAAACAAACCGGTGTTGAACTTCCACAGATACGTCAAACTCAGCTCGACGATTTCTCCAAACTTCTCCGAGATGGTTACGAACTCGCCGGAAAAGTTGTCCACTCCGGCCGTTGGAACATGTACAGGAACCTACAGTTGGCTAGGAAAATGGAGAGGCTGGAGAAAAGAGTAGCGAGGTTCATGCAAGTTACAATGCAAGCTCATGTACTTGCGGATGTTCATCATGTTAGGTTTAATATGGAGCAGAGATTTGACGTGCTTGAGCATAGGCTTAAAGCTATAAAAATCGGAGTTGACGATAAAAGTGGTGGTGGTGGTGGAGGAGGAGGGTGTTTAGGGGAAGCTGTGAAAAGAATGGAAGAAGATGAGAAATGGTTTGAGAATAGTTTTGTAAATTTAGGTGCTGGGATTGAATTGGGGAAGAGGAAAGTGAAGGAGATGCTGATGGGTGAACAGGATAGAGGTGTGTTTGAGATTTGTGGAATTGGGGGTAGTGGCAAAACTACCTTGGCTAAAGAGATTTGTAAAGATGATCAAGTTAAAAGTAAGTTCAAGGACAAGATTTTCTTTTTCACTGTTTCTCAATCTCCAAACGTGGAGCAATTAAGGAAAATGATTTGGGAAAAGATATCAGGGTGCAATCTCCATGGTTATGGACACGGGGAGATGTTACCCCAGTGGAACCTACAGTACCAATGGAATACGAAATCTGCATCCCCAGTACTCTTGATTCTTGATGATGTGTGGTCTGCATCTGTGCTAGAGCCACTAATTTTCAAGATCCCCGGCTGCAAGATCCTAGTTGTATCGCGTATCAAGTTTCCTCCATCAATCATTGACTGTATTTATGATTTAGAGTTGTTAAAGGAAGATGAAGCTATGCCCTTATTTTGCCATTTTGCATTTGGACACAATTCCTTTCCGCGTGGTTTCAGCCAAAAGCTTGTCAAAGAGATTGTGGATGAATGTGAAGGGCTTCCTTTGGCTCTTAAGGTCATTGGATCTTCATTAAAGGGAAAACCTGAGATGTTCTGGATAAGTGCAAAAAACAGATTATCACGATGCCAACCTGTCTGCGAGTCTCATGAACTGCAGTTGCTTGAGCGAATGAAATTGAGTATTGACTGTTTGCCTGAGAAGGTGAGAGAGTGTTTCCTGGACTTGGGTGCTTTCCCAGAAGACAAAAGGATTCCTCTTGATGTTCTAATTAACATGTGGGTGGAGCTACATGATATTGATGAGGAGGAGGCTTTTCACATTCTTGTTGAACTTTCAGACAAAAATCTCCTAAATCTAGTCAAAGATGCACGGGCTGGAGACATGTATACAAGTTACTATGAGATATCGGTGTTTCAGCATGATGTATTACGAGACCTAGCAATTCATATGAGCAACCGTGATGATATAAATCAGAGAAAGCGATTGGTTATGCCACGAAGAGACGTAAACTTTCCAAGAGAATGGGAAAGAAATGTGGACAAACCTTTCCATGCACGAGTTATCTCTGTGCATACAAATGCAGATGAAATGAGAGAAATGGACTGGTTCAGAATGGATTGCCCGAAAGCTGAAGTACTGATTCTCAATTTTGCCTCATCTGAGTACTTCTTGCCTCCTTTCCTGGAGAATATGCCAAAGCTAAGGGCATTGATAATCATAAACTATAGTGCTGGCAATGCAGTTCTTCATAACATGTCTGTATTCAGTCATTTAACCAACTTGAGAAGCCTTTGGTTTGAGAAGATATCTGTCACTCACTTATCTGACTCCACAAATCCTCTCAATAACTTGCGGAAGATATCTCTAGTGCTTTGTGACATGAAAAACAGCTTAGATGAGTCAGATGTGGACCTCCCTGGTTTGTTCCCACAGCTTTCTGAGTTCACAATGGATCATTGCATCAACTTCAATAAGCTGCCATCAAGTATTTGCCGGTTGCATAAGCTCAACAGCCTTAGTATCACTAATTGTGATAGTCTTTATGAACTTCCATCTGATTTAGGTGAATTACAAACTCTACAAGTTATAAGGATATATGCCTGCCCACATCTGAAAAGGCTTCCCCCGGGAATTGGTCATCTGGTAAAGCTGAAGTACCTTGACATTTCACAATGTGTTGGTTTGAGATGTCTCCCTGAAGCAATTGGTTGCTGTAGAAACTTGGAGAAGATTGATATGAGGGAGTGCCCTCAAATTGACAGTTTGCCAAGCGCTCTATCCTTTCTTGAATCATTACGTTGTGTTATTTGTGACGAAGAAGTTTTTTGTCAATGGAAAGATGTTGAGAAGGCTGTACCAGGTCTCTGTGTACAGGTTGCCGAGGAGTGCTATACTCTTGATTGGCTATCTCAGTAATCAAGTTTTCAATTCCCTTATCTTGGGTTTCTTTCGTGTAAATGTAATAAGGCTCAGTCAATTTTCCAGTTCTGTATTTTCCCCTTTGCATGGGGTTCATTGTATCTCTTGTAA

>PGSC0003DMG400028339

ATGGAAGATGAATGCAATTCATTCTATACATTCTTAGTTGAATATCTGTTTGCACAGGAACTTGATACCCTTAGCGTTCTGCAACGTATTGAAGAGTATTGGGGACCATTCCGTATGTATTTGGAGGAATTCAGATCAAAAGTGTCTGATTTCCTTGAAACTTCAGAAGAACATATGTCTTTGTTAGATAGACTGAGGTTCCTTAAAAGGGATTTCAAGTTCCTGAGTATCATTATCGAATTGCATATCTTCGAAGATGAACCACATGTCAGTTGGGGAAGAGTTCGAGCATTGTTCCACGGTGCTGGAGATGAACTCATTCAGATGTACAGTACGCAAGTAAGTCCGTGGAGTCAACAATTTTATGACTATTTTTGCCACTTGCAATATGAGTTTCAGCAGACTAAGTTGGAAATCATAAAGGCTAATTTCCCCGTTCCCAAAATATCATGTCAAGTTTTAGCCAATGAGGATGGTATTGTGATTCCCGGTTTTGTTATGAAATTCATTGATATTGTCGCTGATAATATCAGTAATTTACTGAAATTTGATGATCCAAGTTCACCACTATGTATTGGGGGACGAAGCATGGTTCAAAAAGAAATGGTTTTGAAGGGGTTGAATTTCCTCTCTAGTTTTGTCTGCTTTGTTTCAGATAGATGCATAGAGACTCGGGTCCAACATGCTTTGTTCACTCATGCTGTACAAGTGGCCTGGCAAACAACTATGACTACGTGGTTGTATCTTCCGAGCAATGAATACATGTATCAAGACACAGCTCCGAACGAAGAGAATCCTTTGCTTTCTGATCTTCTGCAGAGCAAAATCCAGCCTATTCAGCCAAGCATATGCAGGTTCTATATTCATATCCTGCAAGCTCTAAATTTGGTTCAGTCACAATGGTATCCCGTTATCAATGTTAAGTATGTATTCGATTGTGAAGTTGGGTTTCTGGAGTCTCTTCGACACACTTTAAAAGAACTACCAGTCTCTAGTAATTGCATTGCAATAAAGGCAGAACTTCAGGAGACGCTCAACTTCTTCGGAGCCATTCTAGTCAATCTACCAACACAGGTCCTTGAATATCATCTTCAAGAAATAGACTCTGCGATTGTTGATGCAGGACTTCTGGTTTTCTCATTAAATGATGATAATGAGAATCTTGATTTCAGGGGAAAAATCCAAAGTATGCAAGGTGTGATCTACCTCGTCGCTAGAAAGAAATTCCTCCTCCAGTTCAACTTACCTGGAATTGACAGAGTGGGCTCTGCTGATTTCATTTTAGACAACAAGGAGAAGTTTCTGAGCATGTATTCAAATTCAGTTGATTCAGTTAAGAACCAACTTCCGATAATTCAGAAAGAACTCAAGTTCTTTCAAGCTGTCGTAGAACAGCAAGACGGACTTCAACATTTTGCAACAAAGACTACTCGTTTGGTGTATGAGGTAGAACATATGGTTGATGCTTGTAAGAAAAAAGATGTTCCTGACTGGTGTATTTTTATCTGGATCTTGAACATTGGAGAGGATATTAGAATGCTCATGGCAGAGGTAGCAGAGATTCATAATGAGATACTGTCATCTCCAAACAAGCTAACTTCATTTGTGCAGTTGGTTCTCAAGGGATTCGTTCGGATCTTTGGTGTTGCTTCATTACAATTCGCTAGTACACGAAGGATCAATGAAGAAATCATAGGCTTTGAGGATGTGAAGGATGAACTGATAGGCAAACTAAAAGGAGGATCATCACGCCTTGATGTAATCGCAATAGTTGGAATGGCTGGATTAGGCAAGACAACTCTGGCTAACAAGCTATATTCTGATAAGTCAGTTGTCTCTTATTTTGACATCCATGCCCATTGCTGTGTCTCTCAAGAATATACGCGGAAGGACTTATTACTAGCCATTCTACATGATATTACTGATGAAAGAGCTAAACTTAGAAGAGAGACTGAAAATGAATTGGCAGATAAGCTTCGCAAACTTTTAATGCGCAAGAGATACCTTCTCCTCATTGATGATGTCTGGGAAACTAGGGCATGGGATGATCTAAAGTTGTGCTTCCCTGAAGATAACAACAGAAGCAGAATTATTCTGACAACTCGGCATTATGAGGTCGCCTCTCATGCTAAACATGATAGTGATCCCCATAAGCTTCGATTTCTCAACAGTGATGAAAGTTGGATGTTGTTAAACAAAAAGGTGTTCAACAATGAAAGTGGTCCTCTTATCCTAAGAGATGTCAGCCAAGAAATTGTAAGAAAGTGCGGCGGACTTCCTATTTCAATTATTCTGGTAGCTGGTATCCTCACAAGAATGAAGAAGGAAAAACATTGTTGGGAACAAATGGCAACAAATTTAGGTACAAACATTCAGGATCAGATGGAGGGCACTCTTGATCTGAGTTATCAGAATTTACCACCCTATTTGAAACCATGCTTTCTGTATTTGGGAGTATTCCCAGAGGATGGAGAGATTCAAGTCTCAAAGTTAACATGGTTGTGGATAGCGGAAGGCTTCATAAAACCTCATACAGGGAAGACTTTGGAGGAAATCGCAGAAAATTACTTGGAGAATCTTGTTGGGAGAAACCTTGTGATGATTGATAAAAGGAGTTCTGATGGAAGGATCAAGACATGTCACATTCATGACCTGGTGCATGAAGTTTGCAGGAAGAAAGCCAAGTTGGAGAACATTTTACAAAGGATAAATGGGAATCAAAACCTTCAATATTTTTCTTTCAATCTATTCATCTTTAAGGCTTCATCTGATCATACATATCTCTCTGTTAACAGGGATGCAGGTTCGGATCCTACACAATTCTTCCCTCCAAAATGCAATACTTCACGTCGCTTATCTCTTCATTCTCAGTGTGATGATCTTGCAAAATGGTGTTTGTGTTTCTCCAATTTGAAATCTTTCCAGTTCAGGGAGTCCAGAAGAACTACATTCTCTTCAATACACCGCACATCAAACATTCTTAAAAGGTTCAAGTTTCTGAGGGTGTTAGACTTTGAATTCACTGTCATTGATTCTTTCCCACAAGAATTGATCCTTTTGAGGTATGTTACTTTTCGGACCGACAATGATACATTATCCCTCCCAGCCAATCTTTGGAACCTTGAAACTTTGATAGTTCAAGGAACTAGAGGACGAATATCACTACCGGAAACCATTTGGAAGATGGTTAAGTTGCGGCATCTGCAAATAAACGACCAAGCATTTTTCACTATGCAGAATGAACAGGAATTCTTAGTAATCCCTTCGAAAATGGATGATTTGCAAACTCTTTCCTCAGTATATTTTTCTTGTGCGGAAAGTGCTGATAAGATATTGGCAAAGACACCAAATCTTCGGAGACTGACATGTGAAGTTTCTGCATTTGATGGCTCATTTACTGCATTTAACAATCTTACAATGCTCGGAATTCTCAAGATGTCTTCTGGTGCTGCATTGACATCAGTCGATCAGCTGAAGCTCCCGTCACACCTCAAGAAATTGACACTGTCCAATTTTAGCATTCACCTCAATGAAGTCACAACTCTTTCAAATCTCGAGGTACTCAAACTGCTAGGAGTTACCATTAGTTCCAATACGTGGAAAGTGAAGGATGAGCAGTTCAGCAAACTCAAATTCTTGAAACTAGAAAATCTATCTTTTTCAGAATGGGATGTCTCAGATGATGCATTTCCATGCCTTGAACACTTGGTATTGAAAAGATGTCGATATCTTGAGGAGATCCCTTCTTGCTTCGGATACATGCCTTCCCTGAAATCCATTGAGGTAGAGTCATGCAAAGAATCACTCGCCGACTCTGCTATGGTCATCAAGGAAATGCAAGTTGAAGACATGGGATTTTCTGATTTTGAGGTCATAATCCACAGAATAGATCAGCAATGCTCCAACACTTGGTCTCGAATTACTTATCAGGTATGTTGTTGA

>PGSC0003DMG400025099

ATGGCTGAGGAGTGTTGCTGTGTGATGGCTTCCTTAAAACTTGCCATTGGTGAGCAGTTTGATAGTTTGACGAATAATCTATTGGACAATGCTGTGGGGAAACTATCATATGTAGCTTTCACTCTCACCTGCCTCAAGAATGCTCACCCTGAGAACGGGATATCTACACAACTTAAGGCCCTATTTGTGGAAGCTCTTGGTGTCTTTTCTGAGAATGGTTCTCAGGTGAATGAACAAGGCGAGACAAGTGATGATGCCATCAGAATGATATCAAAGGTGCTGAAGATAATTCGACTGGAGAATATCGCTGAGCGAATCAAAACTTCAAAGCCATCAAGGTCATCTAGCCTGCTAATTACTTTGGAGATGGTGGAGTCTGTTGTAGCTTTGCTTGATTGTTTTGCTTTTATACTGATTGCATTTGATCCGAAGCGTCATAATGCCTCTCCCGCGTCTGTGCTTAAAAAGAAGCTAAGATACCTACGTGTCTTCTTGAGATTTACTGCAAAGTGGTGCATTGAGCATGAGAGTATAAAGGATCTCTTCACCTGTGCTGAAGATGTAGCTTATGCTGCACTTCACCTATGTTTCTTAGAGTTGGCATACAATATGGAGAACGATGAAGACCAGTCGCATGTGCTGGACCATGAGTTCTCTAAACTGCTGGAAAGGATAGGTCCAATGAACATGCCTGAACTGAGACAGATTTATCTGAACCTATTGATAGAGTCAAAGTCATCACGATCAGAGACTACAATGGATGCAAAATCTATGTATTATTTAGTTGATGCTCTCAAACAGGATCTGGAAGAGCTGCTAAGTCATGATGCCAGCTTGAAAAATACTTTCGATGATCAAATTCCTTGGCTCCAAAAAGGACTTACTCGCCTTTCTAGATTCCTCTGTGACATAGCATTTAAAGGCACTTCACTTGAAGAATTTAGTTCTATTCAGTCACATATCGAAGCTCTGATCATTGAGGCAGCAATTGTGATCTACTCATCCTGCTATGATGGCATGAAGAATACTGAAATAGACCATGAGCTCTTTTTGTTGCAACTGAAGTTTAATCATGTCAAGGTAGAAATCGATCTGATTCAGCTACTAAACGGTGAAGCCACCATAATGGCTCCCTTGAAATATCTGATTGACTGTGTTCGAGAAGAGCTGATACTCTTGGGAACTTTTCTCATGGATTCATCGGAGCAGTGCAAAGAGCAAACTAAGATAACTGATTTTTTGACCCTTAATCAGACTGTGACTAACCAAGCATGGTCAGTCATTAAATCTCTTTCTCGTGACTCGAAGCAAGAAGACATGGCCAGGGAAATTAATCACTTGCACTTTAAATTGCTTCTTAAATTCAAGTTTATTAAGGCAGTGATTAGACAGATGTGTCCCAACATTTCATCATCATCAACACTGGATCATCCTACGATAGATCTTCTGAATTTTCTTCCAATTGACTTTGGTGTCATTGATTCTTATTTCAGCATGCTAAAATCCTCAAAGACACAATCTTCACATATCCCCAAGGTGGATGAGGTTTTGATGGGGTTTCATGAATATATTCTTGGCAATGTGCTACTGAAAGATGAAAGTTATTCGATGTTTACTGTTGCAAATGAGGTTAAAAAGTATTACTATGGGTTGTTGCTCCTTGTAACGTATCTTGTTGAACCTCCAGTTCAGATCAATGAATGCGTGAAGCAGAATGATTTCTTGACTAGATTTGGAACTCTTTCAATTGAAGCTGAATCTTCTATCTGTTCAATTTATAAGGAGGCTGTGGATAGCAACAAAAGTAGGAAGGGCAATCTTATTATTCAGTTTTTGACGATTGCTTTCGATCTTATCAAGTCTGAAGGAAGCTTGATGATTCTACAACAGCAGAAAGCCACTTTGGAAGCTGAAATTCTGGATCAGATTGAAAGTGTTCATGAAGAGCTTATTTTCCTTAGAGCTTTTCTCATGGATGTTCTCACACAACACACACAGCTTAACGAATTGCATGATCTCTTAATGCATGCTGAAGTGACTTCCCACAAGTTAGGACAGATCAGTTGTTCTTGTTATGGGAGTTCCGTGGACGGGTCCAGCACTCAGCAAATGAGGCTTCCATTATCTGATCTTCTACAAGAGATTGAGACTGTCAAGGTAGAGTTCAGAAAAGTATTCTTTCAACTTCTGGATGCATCACCTTGCAACATGACAGGTGGTGAAGGCCTTATCAATTTTTTATCGAACCGCCAAGACAGGCTGTTCAACTATGATGATTGTTCAATCTCTTTTCTGAAGAATCAGATCCTAGTAGTCAAAGACAAATCAGAGTACTTGGGATCTTTTGTTGCAGATATCGTACAGTATCGTGATATGCATCAAGAACTCAAAGACCTCGTGAGACGTGTTCAAGATATAAACTATGTATGTCTCTTCCATGTCAAGGGTTATAAACCCACCTGGTATTACATGTTATATCTCTCTGATGTCAAGCAACTGCTCAAGCATATTGAGGCAGAGGTCAAAATGATCTGTCTTAAAGTTCCACATTCATTAGGTTATAGTTTCCCCAAGACAGATGGATTAGGATTTTTCAGTTGTTTCTTGGGAAAATTGGAGGAGCTGTTGCGTTCTAAGATTGATTCGGTTATCAATTTAAAGCATCAGATTGAATCAGTCAAGGAGAGCTTACTGTGTCTCAGATCATTGATGAATCATTTTGCGGAAAACTTAGATGAGCATGATGAAGTTTATGGTATTATAATAACAAGTGCTACTGAAATGGCATACAAGGCAGAGTATGTCATCGACTCGTGCTTGTCCAGTTCTCATCCACTTTGGTACAAAGTTCTTTGGATTTCTGAAGTTGTTGATAACATTAAGCTTGAAAATCATGTTGTTAGTGAGACTTGTGGAAGAAAGAAGATAGACGTGAAGGTGCATAAATTTGTAAATACCTCTGTGAGTCTTGGACCATCTTTATCAGGTAATACTCCAAGAACAAATGAAGAAATGGAGGGTTTTCAGGAGGCAATGGACAAAATAAAGAAGCAGATACTTAGAAGACCTCCTCATCTAGATGTTATCTCGATAGTTGGTATGGCTGGAATCGGGAAGACCACTCTTGCAGAGAAGATTTACAATGATCTCATAGCTACCCCTCACTTTGATGTTCACGCTAAGTGTCGTGTGACTCAAGTATATTCATGGAAAGAATTGCTGCTTACCATCTTGAATTGTGTTCTTCAGCCTGCTGATCGCACCGAAAAAGAAGATGGTGAATTAGCTAATGAGCTGCGTCAAGTTTTGTTAACCAAGAGATTCCTAATCCTCATTGATGATCTGTGGGATACAACAGCATGGGACTACTTATCTATGTGCTTTAAAGATGCTCATAGTGGGAGTAGAATTATCCTAACAACTCGTCTTACTGACATTGCCAGTTATGCTAAATGTGAAAGCAATCCCCATCATCTTCGTTTATTCAGAGATGATGAGAGTTGGACATTATTACAGGAAGAGGTGTTTCAAGGGGATAGCTGTCCACCTGAACTTGTAGATGTGGGATTTCGAATAGCAAAAAGTTGTGGAGGGTTGCCTCTCTTCATTGTGTTAGTTGCTGGTGTTCTGAAAGAGGAAAAGAAGAACGAAGATTCGTGGAAAAAAGTAGAGGAAAGTCTAGGTTCACGTAACGGTGGTAGCTTGGAAGAGAGCATGTCTTTGATTGAATTCAGTTATAAGAACTTACCACACCATCTGAAGCCTTGTTTTCTCTACTTTGGAGGATTTTTAAAGGGCAAGGATATTCATGTCTCCAAATTGTTTCGGTTGTGGCAAGCTGAAGGATTTGTACAAGAGAACAAGGAAAAAACCACAGAAGATGTCACACAATACTTTTTTGAAGATCTTATTAGTAGAAATATAGTAATGGCCATGGAGAGGAGACCGAATAGCAAGGTGAAAAGGTGTCGTATTCATGATCTCTTGCACAATTTCTGCTTGGAAAAGTCCAAGCAAGAAAATTTCCTTAACCAAATCAATAGGTACAAAAAATTATATTGATGTCATTTTATTATGCTTATACCAACTGCCAAGGGTCCTTCGGAAACAGCCTCTCTACCTCCACGAGGTAGTAGTAAGGTCTGCGTACATTCTACCCTCCCCCAGACCGCATTTGTTGTTTTGTTATGCTTATACCGACTCTCATTGTCATGTATTTATTTATTCAGGGGAGTGGATATGCTTCCTGAAAAGCCTGAGGACTACCGGTTGTTCATGCATTCTTACCAGGATGAGATTGATTTGTGGCGTCCATGTCACTCAAATGTCCGGTCCTTACAATTCAAAGTTGTAGATCCGGACAACCTGTTATGGCCACGTGATATCTCGTTCCTATTTGAAAGCTTCAAACTTGTTAAAGTGTTGGATTTGGAATCGTTCAACGTTGGTGGTACTTTTCCCAGTGAAATACAGAGTCTTATTCATTTGAGGTACTTAGCTGTTCAAACTGATGCAAATTCAATTCCTTCTTTTATAGCTAAACTTCAGAATCTAGAAACTTTTGTGGTAAGAGGATTGGGAGGAGAGGTGATATTACCTCGTTCTCTTCTGAGGATGGTCAAATTGAGGCATATACTTGTAAAACGTCGTGCTTCATTTACTTTGCATGAGAACATGGATGAATCACTTGCTAACTCTCAGTTAAATGATTTGGAAACATTTTCGACTCCACGTCTCTCTTATGGTAAAGATGCTGAGACAATTTTGGCAAAGATGCCAAATTTGAGAAAGTTGAGTTGTATATTTTTGGAAACTTTTAGTTATTCGGAGAAATTGAAGGGAAGGTGTGTTCTTTTTCCGCGATTAGAGTTTCTAAGTCATCTTGAATCAGTCAAGCTGGTTTCCAACAGTTATCCATCTAAACTTCCACACGAGTTCAATTTCCCCTCAAAACTAAAGGAATTGACTCTGTCCAAGTTTCGTCTTCCCTGGTCTGAAATTTCTATAATCGGAGAATTGCCTAACTTGGAGATTCTGAAGTTACTTTTCCGAGCCTTTGAAGGGGATCGATGGGAAGTGAAAGATGCTGAGTTCCCTAAACTCAAGTACTTGATATTGGACAATATCAACTTTTCACAATGGTCCATCTCAGACGATGCTTTTCCTGAGCTTGAAAATTTGAGTTTAACCAAATGTGAGCGGCTTGAGGAAATCCCTTCTCATTTTGGTGAAGCTGTGTCTATAAAAAGCATTGAAGTAAATAGATGTGGATCGTCCGTTGCTAATTCAGCCCTGGAAATTCAAACAACGCAACATGAAGAAATGGCAAATGATGCGTTCACAGTTACCATACAACCTCCAGATTGGGCTACAAGATCATCTCTTTGA

>PGSC0003DMG400000813

ATGGAGAATTCTGAAGAGTCTTGCATCGAACCTTATTATCCATCAATAGATGAAGAACTAGTGGGGTTTGATGATTATGCACAAAATATATTTAAGTATTTGATTAGTGGAGGAAAGGACCTGGACGTTGTCTCGATTGTTGGAATGGCTGGTTTAGGGAAAACAGCTCTGGCTAGAAAGATATACGGTAGTCTTTCTATTCGTTGTCATTTTGATGTTGTATTTTGGTGTTCTGTTTCACAAACATCTAATAGGAGAGAGTTGGTGCTTCGGATTCTAAAACAAATTACACGTGATCATCATTCTAACTATGCTGACTTGGATTATCCTGAAGACATATTGCGAAAGTGTCTATACGGAAAAAGATATCTCATTGTATTAGATAACATATGGGAAGTCCAGGCATGGGATTACATTCGACAATGTTTCCCTGATGGTGATAATGGAAGTAGAATAATGGTAACAACTAGAAATGAAGAGGTGGCTAATTATATGACGAGGTATAGTGATCCTTATTCACTTCCATTTCTAAAAGATGAAAAGAGTTGGGAACTATTGCAGAAGAAAGTATTTAAACGAGGGAACTCGTGTCCTTTGGAGCTAGTCAATGTAGGACAACTAGTTGCCAAAAAATGTAAAGGATTACCTTCTTTGATTATCATGATTGCTATAACTCTTTCAGAAGAAAGAGAAGCGTCTTCGTGGCTTAAGGTTGCATATGATATAAGTTCTCATGTTTTCGACAAGGAGAGAAGCATGATGACGATACGATCAAGTTATGGCCATTTACCGGATCATTTAAAGCCTTGCCTTCTCTATATGGGATTGTTTCCTAAAGATTACGAAATTCCAGTGTCTGATCTACTCAAGTGGTGGATAGCTGAGGAGTTTGTGCAGAACATTGACACGTTGAATCCAGAAGAATTATCAGAGATTTGCTTGCATGATCTTGTTGGCAGAAACCTCGTAGTGGTTTCCAAAACAAGCTTGAATGGTAAAATGAAATGCTGCATAGTTCTTGATCAAGTGCGTGAGTTTTGCTTGAGAAAAATTACAGAAGAAAAGTTCATGCACCTCATAGTGCCGTATAGTTATCCAGATCAACCCGAAGAACAAAGGTTATGCATGTACATACATGACCGTACTATGACAAGTGATTTCAAGGAAAGTGATCAGGAGTTCATTGTTCATCCGAAATTCAGTATATTGGACCGTAAGAATCCTTTCCGTTTGCTTAATAACTTAAAACTTGTTCGGGTTTTACATTTATTGGATATCTACTTGGACAATTCTTTGGCTGCTACATTTCAGTCATTCCCTCACTTGAGGTACCTTGCAATTTTTGTTAAAGCATTTGATTTCAAATGGGTGTCACACCTACTTCATCTACAAACTTTGCGGGTTCGTTCATCTTATATAATGATATCCCCTGCTATATGGAAAATGTCAAAGTTGAGGCATGTGGACATAAACGAATTTCCCGTTACAGTATGGGATGAAGATGATAAAGATGATATTGTGTTAGATAACTTGAAGACTCTTGGAATGTGTTCTATGTCCGCGGCTGACATGACTTGGGAGTTTTGGAAAAAGTTTGCAAATCTTGAAGAACTCAGGCTCCACATAAATGAATTTGAAAATCATGTTCCTTATCATTTTTGCTTTTTCCCCTTACGTCTCAGGTGTTTGTCTCTCAGTGAAATATTCCTAACTGATGGATTAGTTTCAAGTATTACAAATTTGCGATGCCTTGAGACTCTTAAATTATCCGAGATATACTTTGCAGGGGAAAAGTATTGGGATCTCGGTGATAGCACGTTTGTACAACTCAAGTTTTTGAAACTACATCGTGTTTTTATGACCAAATGGAGTTGCTCAGAGGAATCATTTCCTTGCCTTGAATATCTTGTTATAAAAAATTGTCCCAAGCTTGAAGAGATCCCGGATAGCTTTGCTGATATTCCAACACTGCAATTGATTAAAGTGATCAATTGTAGTGAGTCAGTTCGCAATTCAGCTCAAAACATTAAAGAAGATGTAGAAAACACTGAAGGAAATGAGAGACTCCAACTTCATATCCCCAAGAACTACTAA

>PGSC0003DMG400000815

TTTTACATGGAGAACATCGAGTTAACTACTTCTCGATGCTATTTTTCATCGAATGATGAGGAATTTGTGGGGTTTAAGGAGGATGTAAAACAGATCATTAAGAAATTGACTAGAGGAACAAAAGAACGCGAAGTTATATCGATTGTTGGAATGGCAGGACTAGGCAAAACAACTTTGGCTAGAAAGGTGTACAACAATCATTCTGTTGCTGATCACTTTGATGTTCGCGCATTTTGTATTGTTTCACAAACATATAGCATCAGAAAGCTATTGTTTGAGATTCTTAAACAAGCCACGGGTGAAAAACGTGACATGATCAAAGAGGATGAGGACGTTGCAGATACGTTGAGGAAGGCACTTTATTGTAAGAGATACCTCATTGTTTTAGATGATATGTGGAACTATGAGGCATGGGAAGATTTGCAATCATGGTTCCCTTGTGTGGAAACAGGAAGTAGAATTATGGTAACAACTTGCGTCCAAGAGGTTGCTATTAAGATGAGTGATCCTTATTTACTTCGATTCCTAACAGATGAAGAGAGTTGGGAATTATTGCAAAAGAAGGTATTTAAATGCGAGGGGGTTCCCTTAGAGCTAGAAAAAGCAGGATTTGAAGTTGCTCGAAACTGTAAGGGATTGCCTCTTGTGATTGTGTTGAATGCTGGTATTATCGCACAAAAGGTATTATCAAAGTGGATTTGGCCCAAAGCTAGACAAATGCAAATCTAA

>PGSC0003DMG400000816

ATGGAGAATACTGAAGAGTCTTGCATCGAACCTTATTATCCACCAATTGACGAAGAACTAGTGGGGTTTGATGATGATGCAGAAAATATAACCAAGTATTTGATAAGAGGAAGAATGGACCTGGACATTGTCTCGATTGTTGGAATGGCTGGTTTAGGGAAAACAGCTCTGGCTAGAAAGATATACAATAGTCGTTCTATTATTGATCATTTTGATGTTCGAGCTTGGTGTTCTGTTTCACAAACATATAATATGAGAGAGTTGTTGCTTCAGATTCTAAAACAAATTACAGGTGATCATCATTCTAACTATGTTGACTTGGATTATCCTGAATTCATATTGCGGAGGATTCTAAAGGCAACAAAATATCTCATTGTATTAGATAACATATGGGAAGTCCATGGATTGGCTTATTTTCGAGCATGTTTCCCTGATCGTCATACTGGAAGTAGAATAATGGTAACAACTAGAAATGAAGAGGTGGCTAGCTATGTTAAGGGGTATAGTGATCCTTATTCACTTCCATTTCTAAAAGATGAAAAGAGTTGGGAACTATTGCAGAAGAAAGTATTTCAACGAGGGAACTCGTGTCCTTTGGAGCTAGTTAATGTAGGACAACTAGTTGCCAAAAAATGTAAAGGATTACCTTCTTTGATTATCATGATTGCTGAAATTCTTTCAGAAGAAAGAGAAGCGTCTTCGTGGCTTAAGGTTGCATATGATATAAGTTCTCATGTTTCCGACAAGGAGATGATCATGAAGACGATACAATCAAGTTATGATTATTTACCAGACCATTTAAAGCCTTGCCTTCTCTACATGGGATTGTTTCCTAAAGATTACGAAATTCCAGTATCTAATCTACTCAAGTGGTGGATAGCTGAGGAGTTTGTGCAGAGCATTGACACGTTGAAGCTAGAAGAATTATCAGAGATTTGCTTGTGTAATCTTGTTGGAAGTAACCTCGTAGTGGTTTCTAAAACAAGGTCGAATGGTAAAATGAAATGCTGCATAGTTCTTGATCAAGTCCGTGACTTTTGCTTGAGAAAAATTACAGAAGAAAAGTTCATGCAGCTCATAGGTTATCCAGATCAACCCGAAGAACAAAGGTTATGTATGTACATAGAAGACCGTACTATGACAAGTGATTTCAAGGAAAGTGATCAGGAGTTCATTGTTCATCCGAAATTCAGTATATTGGACCGTAAGAATCCTTTCCGTTTGCTTAATAACTTAAAACTTGTTCGGGTTCTACATTTATTGGATATCTACTTGGACAATTCTTTTCCTACTGCATTTCAGTCATTGCCTCACTTGAGGTACCTTGCAATTTTTGTTAAAGCATTTGATTGCAAATGGGTGTCACACCTACTTCATCTACAAACTTTGCGGGTTCGTTCATCTTATATAATGATATCCCCTGCTATATGGAAAATTTCAAAGTTGAGGCATGTGGACATAAACGAATTTCCTGTTACAATATGGGAAGAAGATGATATTGTGTTAGATAACTTGAAGACTCTTGGAATGTGTTCTATGTCCGTGGCTGACATGACTCGAAAGTTTTGGGACAAGTTTGCAAATCTTGAAGAACTCAGGCTCCACATTAATGAATTTGCAGATCATATTTCTGATTATTCAAGCTCTGCATTGATGAATTTGGATATTATTTTCCCCTCGCGTCTCAAGTGTTTGTCCCTCAGTGAAATTTTCCTAACGGATGAATTAGTTTCAAGTATTGCAGAATTGCGATACCTTGAGACTCTTAAATTGTCCGAGATATACTTTGCAGGGGAAAAGCATTGGGATCTCAGTTACACGTTTGGTCAACTCGAGTTTTTGAAATTACATCGTGTTTTTATGACCAAGTGGAGTTGCTCAGAGGATTCATTTCCTCGCCTTGAATGTCTAGTTGTAAAAAATTGTTCCAAGCTTGAAGAGATCCCGGATACCTTTATGGACAGTGGATTTTTGCGATTGATAAAAGTTATTGATTGTAGTGATTCTGTTTGCAATTCAGCTGTGAAGATTAAAGAAGAATTAGGAGAATGTTATGGGATCTCGATCCAAGTTGATATCTCGAAAAAAGACAAATGA

>PGSC0003DMG400030498

ATGGCTTATGCTGCTCTTTCTTCACTTATGCAGACACTGCAGCATCTCTTGCAGGCTAATTCACCTTTGAATAGTTGTGCAAGTTGTATACAACAACAACATGTTGAATCTTCCTATCAAAGTCTATGTGATCTTCAAGTTTTTCTTGAGGATACCACAAATGAAGCCAATGATATTGAAAATCTCAACGTTTTAGAAAAAAAGATCAGAGATGTGGTCTACAAAGCAGAAGATAGAGTTGATTCAAGCTTAAGAAGCATCATTCTAGCAGATATTGAAGACGACCGAGAAAAGGCTTGTAAATTCTTCAATGAAGAATTGCAACAAGTTGAAATAGAATTTGCTTCTCTCAGGAAAGAGGTGATGGTGATCGAGCTTAACAAGCGTGGAAGCAAATCAACAGAATTAGCAAAAACTTCCTCCTCAATATCAACCGAGGAAACGACTTTTGTTGGGATGAAGGATGACTACGAAGCCATACTAAACTGCCTCAATGCCCAAACAAAAGAGCTAATAGTCATATCACTTGTTGGTATGGGCGGTATAGGTAAGACAACTCTTGCTAGAAAAGTTTTTGATAACTCAATTATTCGTGATCGATATGATAAACATGCATGGGTCACCATATCTGAACAATATAATAAGAGACAAATGCTTCTTGAAGTTGCCTCTTCAATTACTGGAATCAACAATCAAGAAATGAGCAATGATGAATTAATGGTGATTGTGTATAGAGGTCTCAAGGGTAGGAGATTTCTAATTGTCATAGATGATCTTTGGAACACTGAGGCTTGGGACCAAATGCGAAGAATATTTCAAAATGATAACAACAAAAGCCGAATTATACTAACCACTCGTCTCAAAGATGTTGCTGATTATGCTAGTTGTCGTGATTTTCCTCCTCGTGATGTGTCTTTCCTAAGTTTAGATGATAGCTGGAAACTATTTACCGAAAGACTATTGATAAAAGATCCTTGTCCTCCTCAACTAGAAGAAATAGGGAAGCATATTGTACGGAAATGTCAAGGATTACCTCTCTCAATCGTTGTCGTTGCTGGAATTCTTGGAAAAATGGATCCAACGCATGACAACTGGAAGAAAGTAGAGGGAAATTTGAACTCATTCTTTGGTACAGTGTCTGAACAATGTCAAGTAATACTTTCTTTGAGCTACAGTTATTTGCCCCATTATTTGAAGAGCTGTTTTCTCTATGTTGGAGGTTTTCCTGAAGATATGGAAATTGTTATTTCAAAGTTGATTAGGCTATGGATAGCTGAGCAATTTGTAAACTCAAGAAGCGATAAAAGTTTAGAAGTGGTGGCAAAGGAGTATCTACAGGACTTAATTGATAGAAGTCTAATTTTGGTTGGTACACGAAAGGCTAATGGAAGCATGAAAACTTGCAAGATTCATGATCTTCTTCGACACCTATGCATAAGAGAAGCTCAAATTGAGAATTGTTTGCAGGTCATAAATTATAATGTCCACAACTCCAAAGAAGACATAAATTACCAACGACGAGTGATGCTTCTTCTTAATATTGACATGAGGCATGTTTATCTTCCGGAACACGGGAGTGGTATTACAAATACAACTCGCAGCTTTGTTGTTATGAGAAATCCTAATGAAGTTCGTGTAATGTGTCCTATTACTTCATGTTTCAAGCTGCTTAAGGTTTTAGATGTTTATTCTATTGATTACGATTTCTCTCGTGTTATACCTCAGCTTGTACATTTGATATATGTTGCTGCAAACATTAAGGAAGCTCCTTCAGTAGCCAGATTGTGGAATCTACAGACCATAATTCTCGATACGTTAGCAGGCTGGATCTACCTCCCTCTAGAGATCTGGACAATGTCAAATGTAAGACATCTTGAAATTGGACGGGCTATAGTTATGGCTAATCCTGTTGAGGCAGAAAGTCATAATAGTATTGGAGAACAACCTGAATCTTTGTACCTGAATAACTTGCAAACACTCTCTCTCGATTCCTCGTCTTTCTTGGCAGAAATCCTAAAGAGAACACCCAATGTAGATAAGCTAAAGATTAGAGCTGTTAATCTACATAACGAATGGTCATCTCTTCTTGATTGTCTCATTCTTCTACAGAGGCTTGAAAAGCTAAGCATAAGAGCAATAGCAGAAAATGGTCCTCTCATTCTCCCAAGTGCTTTTTGCGCGCCTAATCTAAAAAAATTAAGGTTAGATTGGACTAATTTGGCATGGGAAGATATGGTTGTGCTGGCTAATTTACCAAATCTTGAGGTGCTCAAAACAGAAGGTGCATTCAATGGAACAAAATGGATACTAAATGAACATGTTGTGTTTCAAAAACTAAAGTATCTACGATTTGAGCTCGAAGATCTTGAAAGTTGGGAAGCATCTAGTGATAATTTTCTCGTGCTTGAGCAACTAATCCTGATTGATATCAATGAGCTGGATGAGATTCCTGAGAGTATTGGAGAAATAGCCACTCTAAAATTAATTCAAATAGAAAACTGTAGCTCTACAACAATCACTAGTGCAACGAAGATTCAAGAAGAGCAAGAAAGCTTGGGAAATTATGAGCTTCAAATTCGAATTATAGGTATGTTAAAATTCCATCTTTGA

>PGSC0003DMG400030497

ATGGCCTATGCTTGTATTTCCTCACTTATGCAAACTCTGCAGCAACTCCTGCAAGCTAAATCACCTTTGATTTGTGAAAGTTCTATACAACAACATGTTGAATCTTCTTATCAAAGTCTATGTTCTCTTCAGGTTTTTCTAGAGGATACCACAAATGAAGCCAATGATATTGAAAATCTCAAGATTTTAGAGAAAAAGATCAAAGATGTAGTCTACAAAGCAGAAGATAGAGTTGATTCATGCTTAACAAGCATACTTCTAGCAGATAATGAAGACGACCGAGAAAAAGCTTGTAAATTCTTCAATGAAGAATTACAACAAGTTGAAACAGAATTTGATTCTCTCAGGAAAGAGGTGATGGTGATCGAGTGCAACAAGCGTGGAAGCAAATCAACGGAGTTAGCAGCAACAACTTCTTTCTCATCAATCGAGGAAACGACTTATGTTGGTATGAAAAAAGACTACAAAGCCATACTGAATTGCCTCAATGCCCAAGCAAAAGAGCTAATAGTCATATCACTTGTTGGTATGGGCGGTATAGGTAAGACAACTCTTGCTAGAAAAGTTTTTGGTAACTCAACTATTCGTGATCGATTTGAGAAACATGCATGGGTCACCATATCTGAACAATATAATAAGAGACAAATGCTTCTTGAAGTTGCTTCTTCAATTAGTGGAGTCAACAATCAAGAAATGAGCAATGATGAATTGATGGTTATTGTGTATAGGAGTCTCAAGGGTAGGAGATTTCTAATTGTCATAGATGATCTTTGGAGTACTGATGCTTGGGACCAAATGCGAAGAATATTTCAAAATAATAACAATAAAAGCCGAATTATACTAACCACTCGTCTCAAACATGTTGCTGATTATGCAAGCTCCCCTGATTTTCCTCCTCATGATGTGTCTTTTCTAAGTTTTAAAGATAGTTGGAAACTATTTACCAAAAGACTATTCAAAAAAGATCGTTGTCCTCCACAACTACGAAAAATAGGTAAGCATATCATACAACAATGTCAAGGATTACCTCTCTCGATTATTGTCATTGCTGGACTTCTTGGCAAGATAGGTGTGACATATGATAATTGGAAGAAAATTGAGGAAAATTTGAACTCATTTCATGGTTCGGTTTTAGAACAGTGTCAAGCAATTCTTTCTTTGAGCTACGATTACTTGCCCCCATACTTGAAGGCGTGCTTTCTCTATATTGGAGGTTTTCCTGAAGATATGGAGATTCGTGTATCGAAGATGATGAGCTCATGGATAGCTGAGCAATTCATAAAGGCAACAAGCGATAAAAGGTTAGAAGTTGTGGCAGAGGAGTATGTGCAAGAGTTGATGGATAGGAGTCTAATTTTGGGTAAAACACGAAAGCCTAATGGAAGATTCAAAACTTGCAAAATCCATGATCTTCTTAGGCAGTTGTGCATAAGAGAAGCTCAAAGTGAAAATGTTGTGCATTTTCCTTACAGTGATCTTGTTCCCGCTTACTCAGATGACATAAATGATCGTAGGCGTGTGATGATTCCTTTTCTCATTGAGGATTATTTTTGCAATCATCCAAGTCAAATGAGTGGTAAGATTACAACCCGCAGCTTGATCTTTATGGGAGCTGGTAAATCTTATCTAGTACCTGAAATAAATCACTGGCCTGATAGTATTTCAGATTTCAAGCTACTTAAGGTGTTGGATGCACGTGAAATTGGCTACGATTTCTCACATATAATACCTCAACTTGTACATTTGAGGTATATTGATGCAAGAATTGAGAATCCTTCTTCACTAGCCAAATTGTTCAATCTACAAACCATAATTGTCTATTCAAGGAGAAATGTGCAGCTCCCCGCGGAGATTTGGACAATGTCACAGATAAAACACCTCGATATTGAACAAATGGATATGCCTAATCCCCTTAGTATTGGAGATCAACAGCCTGAATTTTTGTACCTGAATAACTTGCAAACACTTGCTCTTGATTCCTCGCCTTTTTTGGCAGAAATCCTAGAGAGAACACCCAATGTAGAAAAGCTAAAGATTAGAGCTGCTAATACACATAATGAGTGGTCTGATTTTGTTGATTGTCTCATTAATCTACAGAGGCTGGAAAAGCTAAGTATAACAGCAACAGAAGACAACAGTCCGCGCATTCTGTCTAGTGCTTTTTGTGCGCCTAATCTAAAACATTTGAGATTAGCTTATACTTCATTGCCTTGGGAAGAAATGGATGTGCTTGCTAATTTACCCAATCTTGAGGTGCTCAAAGCAGATAGTGCATTCAATGGAACAGATTGGACGCTAAATGAAGATGTTGTGTTTCAAAAACTAAAATATCTACGAATTTGGAGCGCAGATGATCTGGAAAGATGGGAAGCAACTAGTGATAATTTTCCAATGCTTGAGAAACTAGTCCTTCAGCATCTCGAAGAACTAGAGGAGATTCCACAAAGTGTTGGAGAAATAATGACATTAAAATTAGTCCAAGTGGAAAATTGCAATCCTGCAGTCGACACAAGTGCAAGGAATATTCTACAAGAGCAGCAAGGCTGGGGAAATTGTGAGCTTCAAATTGAAATTGTACCTTCATTACAATACGATCTTTGA

>PGSC0003DMG400018428

ATGGCATCTTCTTCTTCTTCTGCGAGTAATTCAAAGTATTGTCCTCGATGGAAGTACGTTGTGTTTCTAAGCTTCAGAGGTGAAGACACTCGAAAAACATTTACGGGTCATTTGTATGAAGGTTTGAAAAATAGGGGAATAAGCACCTTTCAAGATGATAAAAGGCTAGAGCATGGAGATTCAATTCCGAAAGAACTCTTGAGAGCTATCGAAGAGTCTCAAGTTGCACTTATTGTTTTCTCAAAGAATTATGCTACATCTAAGTGGTGCTTGAATGAACTAGTGAAGATCATGGAATGCAAAGATGAAGAAAATGGACAAACAGTCATACCAATCTTCTATGATGTGGATCCATCACATATTCGAAACCAAAGTGAAAGCTTTGGAGCAGCATTTGCCGAACATGAATTAAAGTATAAGGATGATGTTGAGGGGATGCAGAAGGTGCAAAGATGGAGAAATGCCCTAACTGTTGCCGCAAATCTAAAAGGATATGATATCCGTGACGGGTTAGTTGAATACACAAATCCAAACTTCAAATGGAAACAACGATGTTCGGATTTTAGGGATCTGGGAATAGGCGGAGTCGGTAAAACAACAATAGCAAAAGCTATTTTTGATGCTATATCTTATCAATTTAAAGCTTCCTGTTTTCTTGCAGATGTTAAAGAAAATGCAAAAAAGAATGAATTGCATTCTTTACAAAACACCCTTCTCTCTAAACTGTTAAGAAAAAAAGATGATTATGTCAATGATAAGTATGATGGGAAGTGCATGATTCCGAGCATACTTTGTTCTATGAAGGTGCTAATTGTGCTTGATGATATAGATCACATTGAGCATTTGGAGTATTTAGCAGGTGATGTTGATTGGTTTGGTAATGGCAGTAGAGTCATTGTGACAACTAGAAACAAACATTTGATAGAGAAGGATGATGCGATATACGAAGTGTCTACACTACCTGATCATGAAGCTATGCAATTATTCAATAAGCATGCTTTTAAAAAAGAAGATCCAGATGAGAGTTTTAAGAAGTTCTCATTGGAGGTAGTAAATCACGCTAAAGGCCTTCCTTTAGCCCTCAAGGTGTGGGGTTCTTTATTGCATAAAAAGTGTCTAACTTTGTGGAGAAAAACTGTAGAGCAAATAAAGAAAAACTCTAATTCAGAAATTGTTGAAAAACTCAAAATAAGTTATGATGGGTTGGAGCTTGAAGAGCAAGAGATATTTCTAGATATTGCATGTTTCTTACGTGGAATTGAAAGAAAAGAAGTCATGCAAATTCTTGAGAGTTGTGACTTTGGAGCTGAATACGGATTGAATGTTCTGATTAATAAATCTCTTGTGTTCATCTCTGAAGATGATACGATTGAAATGCATGATTTGATTGAAGATATGGGTAGATATGTGGTGAAAATGCAAAAGCTTCCGAAAAACCGTAGCAGAATATGGGATGTTGAAGATTTCAAAAAAGTGATGATAGACTATACAGTAAGTAAGCTAAACAATGCAATAATATTTAATTTCTCTTTTTATCAGGGGACCATGACAGTGGAAGCAATCTGGTTTAGTTGCAATGAAGAAGAACTATGCTTTAATAATGAGGCAATGAAAAAAATGAAAAGTCTTAGGATATTACACATATTTGATATTTTCGAATTCTCTGCTCCATTTCGCTCTTCGAATGACCATGATGACTCTATTGAGTACCTGTCCAATAACTTGCGTTGGTTAGTCTGGAATCACTATCCTTGGAAGTCATTGCCAGAAAATTTTAAACCAGAAAAGCTTGTTCATCTTGAACTTCATGGGAGTTCGCTGCATTATTTATGGAAGGAAACAGAGGTACCATTTTATTTAAGCTACTTTCTAAGGAAAAGGGTAAATACCCTCAACTTTGCGATTCAGCATTTGCCGTCTCTACGAACGCTAGATCTCAGCTTTTCTAGAAGCCTGGTGCAAACACCAGATTTCACGGGGATGCCAAATTTGGAGTATTTGAATCTGGAGGGCTGTAGTAAGCTTGAAGAGGTTCACTATTCCCTAGCATATTGCGAAAAACTCATTGTGTTAAATTTGAATGATTGTTACAAGCTTTGGAGTTTTCCACGTGTTAACATGAAATCTCTTGAATCTATGAATCTACGAGCTTGCTATAGTTTAATGGAATTTCCAGAATTCCTCCGTACAATGAAGCCGGAGTTGGTGAGTCTTTCAGTAAACAGTGGGATAAGGGAACTACCATTATCTATTCAGTACCCAATTCATCTAACAGAGCTTTATTTGAATGACATGAATCTTGAAGCTCTTCCAAGCAGCATTGTTACGTGGAAAGGTTTGGTGAAGCTAAATGTGTCGTACTGCTTAACAATTAAAAGCTTGCCCAAAGAGATAGGTGATTTAGAAAACTTGGAGGAACTTGATGCTACACGTACTCTAATTTCACGACCTCCTTCTTCCATCGTCCGCTTGAACAAGCTTAAATCCTTGAAGTTTGTGAAAATTAAAACAGAAGATGAAGTGTACTTTGTGTTTCCTCCAATTAATGGCGGGTTACTCTCATTGGAAATCCTGGATCTCAGTTCCTCCAAATTCATAGATGGAAGAATTCCGGAAGATATTGGATGCTTATCCTCTTTGAAAGAGTTGCATCTCAAGGGAGATAATTTTGAGCATTTGCCTCAAAGCATTGCCCAACTTGGTGCTCTTCGACTCTTGTACTTAAACGATTGCAAGAGGCTTACACAGCTGCCTGAATTTCCACCTCAATTAGATACAATATGTGCAGATTGGCGCAATGATTTGATCTGTAATTCACTGTTTCAAAATATCTCATCATTCCACCATGACATCTCTGCTTCAGATTCCTTGTCGTTAAGAGTGTTTACGAGTCAGGGGAGTAATATCCCTAGTTGGTTCCACTATCAGGGAATGGATAAAAGTGTTTCAGTCAATTTGCCTGAAAATTGGTATGTATCAGATAACTTCTTGGGATTTGCTGTATGTTACTTTGGAAGTTTAATTGAAAACACGGCTCAGTTGATTATTAGTTCTGAAGGGATGCCGTGTATCACCCAGAAACTTGTCTTATCCAATCATTCAGAATGTTATGCATATTTTAGGATTCAGTTTTTCTTGGTACCTTTTGCTGGCATATGGGATACATCTAACGCAAATGGTAAAACACCAAATGACTATGGGCACATTATGTTATCTTTTCCTGAAGAATTGGAGGAGTTTGGACTTCGTTTGTTCTATAAAGATGAATCTGAGCTTGTTGAGACCAATGATGAACCACCAACAGAACTTTCCATTGAGACCAGTGATGAACCATCAACAGAACTTTCCATTGGGATAAGGAGGATCAGATATGACGATAGTGAACATCATGAAGAAGCCAGTTGTAACTCTTCTAAGAAACAAAGGTCATAA

>PGSC0003DMG400018429

ATGGCATCTTCTTCTTCTTCTGCGAGTAATTCAAAGTATTGTCCTCGATGGAAGTACGTTGTGTTTCTAAGCTTCAGAGGTGAAGACACTCGAAAAACATTTACGGGTCATTTGTATGAAGGTTTGAAAAATAGGGGAATAAGCACCTTTCAAGATGATAAAAGGCTAGAGCATGGAGATTCAATTCCGAAAGAACTCTTGAGAGCTATCGAAGAGTCTCAAGTTGCACTTATTGTCTTCTCAAAGAATTATGCTACATCTAGGTGGTGCTTGAATGAACTAGTGAAGATCATGGAATGCAAAGATGAAGAAAATGGACAAACAGTCATACCAATCTTCTATGATGTGGATCCATCACATGTTCGAAACCAAAGTGAAAGCTTTGGAGCAGCATTTGCCGAACATGAATTAAAGTATAAGGATGATGTTGAGGGGATGCAAAAGGTGCAAAGATGGAGAAATGCCCTAACTGTTGCCGCAAATCTAAAAGGATATGATATCCGTGACGGGTTAGTTGAATACACAAGAGGATACATAAAAGTCCTCCTAAACTATCAATCTTCTTCTTTATGTAGGATTGAATCAGAGAATATTCAACAGATCGTAGACTGCATCTCTTCCAAGTTTCGCACAAATGCTTATTCTTTATCTTTTTTGCAAGATGTTGTGGGAATAAACGATCACTTAGAGAAACTAAAATCCAAACTTCAAATGGAAATCAACGATGTTCGGATTTTAGGGATCTGGGGAATAGGCGGAGTCGGTAAAACAACAATAGCAAAAGCTATTTTTGATGCTATATCTTATCAATTTAAAGCTTCCTGTTTTCTTGCAGATGTTAAAGAAAATGCAAAAAAGAATAAACTGCATTCTTTACAAAATACCCTTCTCTCTGAACTTTTAAGAGAAAAAAAAGGTTACGTCAATAATAAGTATGATGGGAAGCGCATGATTCCGAACATACTTTGTTCTATGAAGGTGCTAATTGTGCTTGATGATATAGATCACAGTGAGCATTTGGAGTATTTAGCAGGTGATGTTGGTTGGTTTGGTAATGGCAGCAGAGTCATTATAACAACTAGAAACAAACATTTGATAGAGAAGGATGATGCGATATACGAAGTGTCTACACTACCTGATCATGAAGCTATGCAATTATTCAATAAGCATGCTTTTAAAAAAGAAGATCCAGATGAGAGTTTTAAGAAGTTCTCATTGGAGGTAGTAAATCACGCTAAAGGCCTTCCTTTAGCCCTCAAGGTGTGGGGTTCTTTATTGCATAAAAAGTGTCTAACTTTGTGGAGAAAAACTGTAGAGCAGATAAAGAAAAACTCTAATTCAGAAATTGTTAAAAAACTCAAAATAAGTTATGATGGGTTGGAGCTCGAAGAGCAAGAGATATTTCTAGATATTGCATGTTTATTCCGTGGAAAAAAAAGAAAAGAAGTCATGCAAATTCTTGAGAGTTGTGACTTTGGAGCTGAATACGGATTGAATGTTCTGATTAATAAATCTCTTGTGTTCATCTCTGAAAATAATATGATTGAAATGCATGATTTGATTAGAGATATGGGTAGATATGTGGTGAAAATGCAAAAGCTTCCGAAAAAACGTAGCAGAATATGGGATGTTGAAGATTTCAAAAAAGTGATGATAGACTATACAGTAAGTAAGCTAAACAATGCAATAATATTTAATTTCCAGCATTTGCCGTCTCTACGAAAGCTAGATCTCTGCTTTTCTAAAAGCCTAGTTCAAACACCAGATTTCACGGGGATGCCAAATTTGGAGTATTTGAATCTGGAGTACTGTAGTGAACTTGAAGAGGTTCACAATTCCCTAGCATATTGCGAAAACCTCATTGAGTTAAATTTGAATTGGTGTAACAAGCTTAGGAGATTTCCATGTGTTAACATGAAATCTCTTGAATCTATGGATCTACAATCGTGCTATAATTTAATGAAGACGGAGTTAGTGATTCTCTCAGCAAAGGGTTGGATAAGGAAATTTTCATCATCTATTCAGTACCTAACTGATCTCACAAACCTAGATTTGAGTGGCATGGAAAACCTTGAAGCACTTCCAAGCAGCATTGTTAAGTTGAAAGGTTTGGTGAAGCTAAATGTGTCGTACTGCTTTACAATTAAAAGCTTGCCCGAAGAGATAGGTGATTTAGAAAATTTGGAGGAACTTGATGCTACATGTACTCTAATTTCACGACCTCCTTCTTCCATTGTCCGCTTGAACAAGCTTAAATCCTTGAAGTTTGTAAAAATTGAAACAGAAGATGAAGTGTACTTTGTGTTTCCTCCAATTAATGGCGGGTTACTCTCATTGGAAATCCTGAAGCTCAGTTCCTCCAATTTCATAGATGGAAGAATTCCGGAAGATATTGGATACTTATCCTCTTTGATAAAGTTGCATCTCAAGGGAGATAATTTTGAGCATTTGCCTCAAAGCATAGCCCAACTTGGTGCTCTTCGAGTCTTATCCTTAGAAGGTTGCAAGAGGCTTACACAGTTGCCAGAATTTCCACCGCAATTAGATACAATATGTGCAGATTGGCACAATGATTTGATCTGTAATTCACTATTTCAAAATATCTCATCATTCCAGCATGACATCTCTGCTTCAGATTCCTTGTCGTTAAGAGTGTTTACGAGTTGGGGGAGTAATATCCCTAGTTGGTTCCACTATCAGGGAATGGATAAAAGTGTTTTAGTCAATTTGCCTGAAAACTGGTGTGTATCAGATAACTTCTTGGGATTTGCTGTATGTTACTCTGGAAGTTTAATTCAAAACATGGCTCAATTGATTATTAGTTCTGAAGGGATGCCGTGTATCACCCAGAAACTTGGCTTATACAATTATTCAGAATGTATTCCAGATTGTGTGATTCAGTTTTTCTTGGTACCTTTTGCTGGCATATGGGATACATCTAACGCAAATGGTAAAACACCAAATGACTATGGGCACATTATGTTATCTTTTCCTGAAGAATTGAAGAAGTGTGGACTTCGTTTGTTCTATAAATATGAATCTGAGCTTGTTGAGACCAATGATGAACCACCAACAGAACTTTCCATGGGGATAAGGAGGATCAGATACGACGATAGTGAACATCATGAAGAAGCCAGTTGTTCCTCTTCTAAGAAACAAAGGTCATAA

>PGSC0003DMG400018441

ATGGCAGATTTCACCTACAAAGTACAAGCTCTGTTTCAAGGTACTGCCGTTGACCTCAAAAAAGTCAACAAGATCCATCACATTGATCGTGTTACCTCTCGGATACAAGAAAAGATACGGATCACTAAGTTGGAAATCAGAGATATCCATTTAGTATTATCCAATAAAGACGGCATCGATACTCCTGAATTTGTAATTGAATTCATTGATACTGTCATTCAGAATCTGTCTGATTTTGTTGAGCTGGGAGATTCTGTCTTTAAGGAGTTGAAGTTACTGAGGAACTTTGTTTGCTTTGTTTCGGGCAGATCCATGGAGCCGAAAAGCCTACATGCTTTCTTGACTCATGTTTTACTTGTGGTTGGTCGCGCAGCAATGATTGTCTGGTTGTATTTTCCAAGCAATGACAGCGAAAATCAAGAAATGAATGATCTGTTTTCTGATCTTTTGAAGATGAAGATTAAGCCCATTCAGTCAGGCGTTCGTAAGATCTATATTGATGTCCTGCAAGCTGTAACACATCCGATTATCCAAATTGAGAATGCAGCTGATTGTATAGTTGGCTTTATGGAGACTCTCCAACACAACTTGAAAGAGCTACAAACTATTAGTAATCTCAGTCAGATTGTTGGTTTGACAGATCAAATAGTAATCCTTCAGGAGATGCTTGACCTCTTATTTGACAATATCAGATGTCTGTCTGTACAAGTTTTCGAATTTCACCTCCAAGACATTGACACTGTGGTGGTTGATTCTGGACTTCGTGTTTATTCGTTACATGAAGATGTGGCATCGGAGGAAGTGACTCCTGATTTGCAAGGTACTATCGAGCGCGTCAAGACATTGATCTACCATATTATCCGAAAGGAGTTCCAATCTAGTTTGCCAAGGATTCATGGAATAGGCTATGTTGATTTCCTCTTAAGCAACCTGAAGAAGTTCCATGACCATTATTCAAATTCGCTTGCTCATTTCACGAATGAACTTCAAATGATTCAAACAGAACTGGAGAGCTTACAACCTTTTCTCAGATCTGTTGCATTAGAGAGACACAACAAATACGACAAGCTTCAGCATTCTGTGGCACTAGTGATTGGCAAAGCATATGAGGTTGAATATGTCGTTGATGGTTGTGTAAGCAATAAAGTTCCTGAGTGGTGTATCATGCTTTGGCTCTTGGAACTCATAGAGGAGATTAGAGCAGGGGTAGCAAAGATCCAAAGTCTTGAGGTTGACTCAGCGTCACATGGTACCGAAGATACTTACACTTCTCATACTCAAAGGATTGATGATGATATTGTAGGCTTTGAGGATGAAATTGGAACACTAAGACGCCAACTAACAGGAGGATCCAAAAATCTAGATATTATCTCAATTGTTGGCATGCCTGGAGCAGGAAAGACGGCCTTGGCCAACAAACTCCTTTTGGATAAATCAGTTGCCGATCGCTTTGATATCTTTGCACAATGTTACATGTCTTCAGTACATTCACGGAGGGAACAGTTACTGTCTATTCTGGAGACACTACGTGTTTCTATTGATGAGAGCTCTCCTTTTGGTAAAGAGACTAGTGATTTAGCAGCTATGCTTCGCCAAATTTTACAAACCGGAAGATATCTCATTCTCCTTGATGACGTACCAAATGCTAGGGCATGGAATGATTTAGAACATTGCTTCTGTGATACCAATAATGGAAGTAGAATTCTTCTAACAACGCGACACTCTAATGTTGCCTACTATGCTAGATCAATTAGTGAACCCCTACATCTTCGCATGCTTAATGATGGTGAAAGTTGGACATTACTGAAAAAGAAAGTATTTGGTGAAGGATTCTGCTCCCCTCTCTTTGAAAAAGTTGGGCCAAAGATAGTACGAAAGTGTGGAGGGCTGCCTCTTTCAATTGTTTTTGTGGCCTGTATACTCGCTGGGATGGAGAGGACGGAACAATGTTGGAAGCAAGTGGCCAGGAGTTTAGGAACTGAAATTCGCTGTTACCCGGAGAACATTATAGAACAAAGTTATCAGAACTTGCCGTATCATTTAAAGTCATGCTTTCTTTACTTTGGAATGTTTTCAGATGAGGAAGAAATTAACATTTCAAAGTTAACATTGTTGTGGATTGGAGAAGGATTTGTAAAAAATGATGAACACAGGACTTTGGAAGATATAGCAGAAGGTTACTTGAAGAATCTTGTTGAAAGTAATCTAGTGATGCTTGCTAAGAGAAATTGTGGTGGTAAGGTCAAAGTATGTCGCATTCATGATATCTTGTTTCAGTTCTGCAAGGAAAGAGCGCATACGGAGAATCTTATACAAAGGATACAGAGGTCTAGGTCCGAAGGAGACGTTTACTTTCCCAATCAACTTGGTCAACGCCGATTGGCCTTCTATGCTGAAGTTGATGATCTAGTAGAATGGAGATCGTCTTGCTCACTTGTTAGCTCTGTGCTGGTCGGGAAAGATAACATATATGCATTCTCTTCGATTGCTAATGCCTCAGACATGTTTCATGATTTTCGGTTTCTAAAAGTATTGGATTTGGAGTTCACTGTGATTGATTCTATCCCAACTAACATGGTCTACTTGAGATATTTTGCTGCACAAACTTCTCACGAGTCAATTACATCATCCATACACATGTTGTGGAACCTTGAAACTTTGATTGTCAATGGAATGGGAGAATATATGTCAGTACCCTCAACAATTTGGAACATGGTTAAATTGAGACATCTGCACATTTCCCCCTCCTTTACTGCAGAGGAATTGCTTGAGGACTCATCAAAACTTAATGATTTGGTAACTTTTTCTACTCCATATTTTTCTTGTGTAGAGGATGCGGAATTGATGCTGGGGAAAACGCCTAATATTCGGGAACTGAAATGCAAATTTAAGGGTTTGAGCAGTGATCAATTCAGTGTGTTGGATTTTCCAACACAGCTTGAGGTGCTACACATTTTTGGTGATGAACATGTAGAATCCTTACCATATCTTGTATGCATCTCTGCGTCGAATCTCAAAAAATTGAAAGTATCCTATTATATACTGGGCTCTCAACATTTATCAAGCTTTTCTCAGCTTCAGAACCTTCAAGAACTTGAACTGAATTTTGTTGAATTTGCGGATGAAGAATGGGAAGTGAGTGACGAGTTTGCTGAACTCAAAGTTTTGAAAATAGTGAACTGCTCCAGTTTTAAAGAATGGACTGTTTACGATGATGCATTTCCTAAGCTTGAACACATGGTTCTGCATTGTTGCCAATTACTTGTGGAAATTCCTTCTTGGTTCGCAGAAGTTTCTTCTCTAAAGTACATTGAGGTAGATAATTGCAATGGATCTGTTGTCGAGTCAGCCAGGATTATCCAGAGAACAAAAGTTGAAGAGTATGCCAATGATGATTTAGAGCTCGTCATCAAGGAATCATAA

>PGSC0003DMG400018442

ATGAACCATAATATTCTTCAATCTCTGCAACGTGTTGAAGATCAATGGGGTTTAGATATGCCGGAGAGTTTAGAGGATGTAATCAAGTTCCTTAAAAGAGAGGTTACATTCCTTGATTTTTTTCTCAGCTTACAGAGCTTCACAGATTTATCATACATGCTTGAAATCGTACACAACGTTCCAGCTCTGTTTCATGATGCTGCCGTTAACCTTAAAGAAATCAGCGAGATCGATCATGTTGATGGCGTTACCAATCAATTGCAAGTCAACATTCAGAAGACTAAGTTGGAAATCAGATATGAATACTTATTTCCAAAAGTCAACAAGGATGGTATTATTGTGACCCCCAAATTTGTAATCGATTTCATTGATACTGCTGTAGTGAATCTGGGTAATTTATTGAAGATTTATTGTTCTAGTTCACTACTTTTTGTTCGTGGACCTAACAAAGAGATCGGAGATGTTTTTAAGGAGTTGAAGTTATTGAAAAATTTTGTATGCTATGTTTCGGACAGATTCATAGAGGTGAAAAGTCAATATATTGATTTCTTCATTCATGTTTTAGAGGTGACCAGCCACGCTGCAATGGTTGCCTGGTTGTATCTCCCAAGCAATGACAACGAAAATCAGGAAACGAATGGTTTGCTTTCTGATCATCTGAACATGAAGATTAAGCCCATTGACCCAAGCATCCGCAAGATCTATATTGATGTGCTGCAAGCTCTACGATCAGAATGGCGTCCAATTATTCCAATTGATCATGTAGCTGACTGTGTAGCTGGCTTTGTGGAGACTCTCCAACACAACTTGAAGGCACTATCGGTTAGTAATCTCAATACTCATCAAATATCAGCTCTTCAGGAGATGCTCAACCTCTTAATTGCCAATCTGTCCATACAAGATCTTGAATTTCACCTTCAAGATATCGATACTGTGATGATTGATTCTGGAATACTTGTTTACTCGTTGTGTGAAAATGTGGTGTTAGGGGAAGTGACTATTGATTTACCAGTTATGCTTGAGCACATCAAGATATTGATCTACCACATCATCCGGAAGGAGTTCCAATCTAATTTGCCAAGGATTCATGGACTAGGCTATGTTGATTTCGTCTTAAGCAATTTGAAGGAGTTCCAAGACCGTTATCCAGTTTCATTTGCTTTTATGAAGATTCAACTTCAAAAAATTCAAAAAGAACTGGAGAGCGTGCAACCTTTTTTGAGGTCGGTTACAGAAAAACAATACAATATTCACGACAAACTTCAGAAAAGTGTGGCTCTACTGATTGGCAAAGCATATGAGGTAGAATATATCGTTGACTCTTGTGTGAGCAAAAGAGTTCCTGACTGGTGTCTCATGCGTTGGCTCGTGGACATTAGCGCAGAGGTTGCAGAGATGCAACAAAAGAAAATCTTTGAGGTTGACTTAGTGTCACCTTATACCATTTCTATTGATACTTCCAGTAAGTCGTCAGAACTGAAAAAAATGTCAGCGATCAAAGAAAAAATTATAGGTTTTAAAGATGAGATCGAGACATTAGTACACAAACTAATACGAGGATCCAGAGAGCTGGACATTATCTCCATTGTTGGTATGCCTGGAGCAGGAAAGACAACTTTGGCCAACAGACTCTATTCTTATGATTCAGTTGTCTCTCACTTTGATATCCGTGCACAATGTCATGTGTCTCCAGAATATTCGCAGAGGGGCCTATTACTGTCTCTTTTGGCGATGCTACAGGTTTCTATTGATGAAACATCTCTTCTTAGTAAAGAGACTGATGAATTAAAAGATATTCTTTCCAGAATTTTACGTTCCAAGAGATATCTTATCCTCCTTGATGACGTATGGGATCACAAGGCGTGGGATGATTTAAAATGTTGCTTTCCTGATGACAATACTGGAAGTAGAATTCTTCTTACAACGAGAAATCACGATGTTGCCGACTATGTTAAATCAGTGAATAAACCCCATCATCTTAGCTTGTTGACCTACGAAGAAAGTTGGGAGCTACTAAAAATGAAAGTGTTTGGCAATGGAAGTTGCTCTCCTCTCCTTGAAAAAGTAGGGCAAGAAATCGTAAGAAAGTGTGGAGGTCTGCGGTTGTCAATTGTTTTGGTGGCTGGCATTCTATCAAAAATTGAGCAGACAGAAGAATGTTGGAGTGAAGTGGCTAAGCATTTAGGTATCAATATGTTAAGTGCCTTAAATGACATTATAGAGCAAAGTTATCAACATTTGCCCTATCATTTAAAGTCCTGCTTTCTTTATTTTGGAACTTTTTTAGAGTTCAAAGAAATCAATGTTTCAAAGTTAACATGGATGTGGATTGGAGAAGGCTTTGTAAACGATCTTGAAGGTAAGAGTTTGCAGGACATAGCGAGAGGTTACTTGGAGAATCTGATTAGAAGAAACCTAGTGATGAATGCTAAGAGGAGTTCTGATGGTAAGGTCAAAGCATGCCGCGTTCATGATCTATTGCTTGATTTCTGCAAGAAAAGAGCAGCGGAAGAGCACTTTCTATCATGGATAAAATGGTACATACACAGGGACCAAAACGACAAATCTTTGTTAGGTATATCCTCTCGCAAGCAGTTCGCTCAACGTCGTCTGGCCTTCTACTGTGAAGAGGAGAATCTTGTAAAATGGAGTTCGTCTTGCTGTCCTGTTGACTCTGTACATTTCGGGGAAGACAAAAGAACAAATGTTTCTAGTCGCCAAGTCCCACAAATTTTTTACAACTTCAAGTTTCTTAAAGTGTTGAATTTGGAGTCCACTGTTATCAATTCTTTCCCAACGGTGCTAGTTTACTTGAGGTATTTTGCTGCACAAACTGATCAGGATTCAATTACATCGCTCATTGCCAATCTTTGGAACCTTGAAACGCTGATACTCAAGCCAACAAAAGGAAAGCTGAAACTACCCGTTACAATTATGAAGATGGTTAGATTGAGACATTTGTGCATCGATAACACTTATTTCACTTTAAATGGTGAGGAAGGATTACTTGAGAAGTTGGAAGTTCTTTCCACTCCATGCTTTTCATGTGCAAAGGATGTGGAATTGCTAGTGCAAAAAACGCCGAATCTTCGGGAGCTGAGATGCTCATTTGTTGGTTTCAGACAAGAATGTTTACCTTGCTTGGACTTCCTTGAGAAACTTGAGATTCATTTAGCAGCAGATTCAACTGTAAGTGGTCCATACATCTTCCCAGCAACGGTCAGAAATTTGACACTATCCAACTTTTTCCTGGGCAGTTGTCACCGATCCAACATTTCAATGCTTCCCAACCTTCGCGTGCTGAAACTGGAATCAATTTTCTTTGACAATGATGAGTGGGAAGTGAGAGATCACGAATTCTTTCAACTCGAGGTCTTGAAGCTTGTAAAATGTGAATTTCTTGAAGAATGGAATGTCTCAAATTCTGCCTTTTGTATGCTTGAACATTTGGTTTTGCGTGAATGCCCATATCTTAAGGAGATCCCTTTTTGTTTTCGAGACAAGTCTCTATCCATCAAGGTGAAGTCATGCAGCGAATCTGACGAGCGGTCAGCCACAGATATCAAACTATATCAGGAAGATTATTATCGGGATTTCAGCCAGGAGGAGTGGTGTAGTGAATCTGTTGAGCAGTCATCACCCTCAGATATCAAACAATACCAGGAATATAGTTCTTGGGATTCCAAACTCGATATCTTCATCCCGAAGAAGAGCATGAGTAGCTTATCAGGTACGCTACTGAGAAAATGCAATTACACGTCACTCTCATTTCTATTTTAA

>PGSC0003DMG400025545

ATGTATTTCAACAATGAATTGTCTGATCTGAAAAATAGCTTCCGTAGAAGGCTGGAACTTTTCACTGACTCGGATGTTGGAAGGGATCGAATAAATTTCTTTCTATGGGAGTTAAAATTTCTTGATTGTTTTCTCTATTTGAAAAGGTTACCTTTTGCAAGTGAATGCGGTATTCTAAATGTCTCACAGAAAATGATAGAATTTTGGAATATTCCGTGGGATAGGGTAATTACTATGTATATGTATGCTAAAAGAACTAGTCCTTTCGATGCATTTGTCTACTGGAAGGAGGCAATTTGGAAGACTAAGCAAGAATTCAGAGCCCAATACTCCTTTCCGAAATCACCACTTGCAGCCAACAAGGTGAGCCCCGAATTTGTGATGGAAGTCATCGATGATTTTGTGGGGAACATCAATGTTCTAGTGAAGATCAATGATCCATGTTCGTGGTTTTTTGTTCCAGGACTCAAGGAACAAATAGAACAAGTGTTAAAGGAGTTGAAGTTATTGAGATTTTTTGTCTGCTTTGTTTCAAACAAATGTATAGAGCCTCAATACGGATGTACTACTTTTTATACTCATGCTTTAATTGAGGCCAGTCACATCGCAATGGTTGTGTGGATGCATTTGCCAGTCTATGGCAACGGAAATCAAGACTTGGCTCCAACTGAAGTCAGTCGTTTGCTTTTCTCTGATTTCATGGAAATGAAGATTAAGCCCATTCAGCCAGGCAACAGTATTTATATTGATGTCCTGCAAGCTTTGAAGTCAACCATACCACAAGCTCAAAAGAAGCATGCAGCTGAGAGTGGCATTGTGGAGATTCCAACAAACGGTTTGACGGTTGGTTTCAGTGATCAAATGGCTAACCTTCAGGACATGCTCTGCCTTCTAAGAGACAATCTCATTCATCTGCCAATACTAGATCTGGAATTTCATCTTCAAGATATGGATTCTGTTATTGTTGATGCAGGACTTCTTATTTACTCATTATATGATATCAAGGGGGAGAAGGAAGACACAATATTGGATGATATGAACCGAGCACTTGGTTTTGATCTTCCAAGAAACATTGAGCCGATCAAGGCGATGGTCTACCTTGTCATGCAAAAGGCATTTCAATCTAACTTGCCAAGGATTCATGGACTAGGCTATGCCGATTTTCTATTGAAACACCTGAAGGATTTCCAAGGCCGTTATTCAGATTCACTTGCTTTCCTCAAAAATCAACTTCAAGTAATTCAAACGGAATTTGAGAGCATGCAACCTTTCCTGAAGGCTGTTGTAGAAGGGCCACACAACAAGCTCAAGACACTGAATGAAGATTGTGCTATACAGATAATTAGGAAAGCACATGAGGTTGAATATGTAGTTGATGCTTGTATTAACAAAGACGTTCCTCAGTGGTGCATCGAGTGTTGGCTCCAGGATATCATAGAGAAGATTACTTGTATCAAAGCAAAGATTCAGGAAAAGAACATGGTTGAGGATACAATGAAGACTGTCATTGCTAGTACATCATCACAATTGGTAAGGACTCCAAGGATGAATGAAGAGATTGTTGGGTTTGAGGATGTCATAGAAAATTTAAGAAAAAAACTACTGAATGGAACCAAAGGGCAAGATGTCATTTCAATTCACGGCATGCCAGGTTTAGGTAAGACGACTTTAGCCAACAGACTCTATTCTGACAGGTCAGTTGTTTCTCAATTTGATATTTGTGCACAATGTTGTGTGTCTCAAGTATATTCGTATAAGGACTTGTTATTGTGCTTGATACATGATGCTATTGGTGAGGATTCTGATCAGCATAGAGAACTTCATGACAATGAATTAGCTGATAAGCTTCGCAAAACCTTATTGCGCAGAAGGTACCTTATCCTTGTTGATGATGTGTGGGAAGATAGTGTTTGGGATGATTTAAGAGGTTGTTTTCCAGATGCCAATAACAGAAGCAGAATCATTCTAACGACAAGACATCATGAAGTTGCCAAATATGCTCGTGTTCATAGTGATCCCCTTCATCTTCGTATGTTTGACGAAGATGAAAGTTGGAAGTTGCTTGAAAAGAAAGTGTTTGGTGAACAAAGCTGCTCCCCTCTCCTAAGAGATGTTGGGCTAAGAATAGCAAAAATGTGTGGACAACTCCCTCTTTCAATTGTTTTGGTGGCTGGTATTCTGTCGGAGATGGAAAAGGAAGTAGAATGTTGGGAACAAGTGGCCAACAATTTGGGTACCCACATTCACAATGACTCAAGAGCCATTGTAGACCAAAGTTATCATGTTTTACCCTGTCATCTTAAGTCTTGCTTCCTTTATTTTGGAGCATTTTTAGAGGATAGAGTGATTGATATTTCAAGGTTAATAAGGTTATGGATATCAGAATCACTTATAAAAAGTTGTGAAGACAGGAGCTTGGAGGATATAGCAGAAGGTTACTTGGAGAATCTTATTGGAAGAAATCTAGTAATGGTTACTCAGAGGGCCATTTCAGATGGTAAGGTCAAAGCATGTCGCCTTCATGATGTATTACTCGACTTCTGCAAGGAAAGAGCAGCTGAGGAGAATTTTCTACTATGGATAAAACGGTATATTCACAGGGATCAGAGTACCCAAACTGTTTACTCTCACAAGCAGCATGCTCACTTAGCCTTCACTGAAATGGATACTCTTGTAGAATGGAGTGCATCTTGCTCATTTGTTGGCTCTGTACTTCTTAAGAATTATGACCCACACTTTGACGCATTTCACTTAGCCTTCGCAATGAGTACATCCTCTCATGCTTTTGCAATTTCACGCATTTTATTAAATTTCAAGTTTCTAAAAGTATTGGATTTGGAGCACGACGTTGTTATTGATTTTATTCCAATTGAGCTCTTTTACTTGAGGTATTTATCTGCACACATTGATCAGGATTCAATTCCTTCAAGCATATCCAATCTTTGGAACCTTGAAACTCTTATATTAAATCGTAGACCAGTTGTTAATCAGGAAACGCTATTACTACCTAGTACAATTTGGGATATGGTTAAACTGAGACATCTGCATATTCGTAACTTTAGCGCAGAAAGTGAAGATGCATTACTCGAGAACTCCCCAGAACTTTCTGATTTGGAAAGCCTTTCCAGTCCATTTTTCGCTCATGTTGAGGATGCAGAATTGATGCTGAGAAAAACACCTAATCTTCGAAAACTGATATGTAAAGTCCAGTCCTTAGAATACCCCCATCAGTACCATGTGTTGAATTTTCCAATACGGCTTGAAATACTAAAGCTTTATCGATTAAAAGCCTTTAAAACCATCCCCTTTTACATCTCTGCACCAAATCTCAAATACCTAGAACTCTCTGGCTTTTACCTAAATTCTCAGTATTTATCAGAAACTGCTGATCATTTGAAGAACCTTGAGGTACTCAAACTGTACTACGTTGAATTTGGTGATCATAGGAAATGGAAAGTGAGCAATGGCAAGTTCCCTCAACTCAAAATCTTGAAACTAGAAGATTTGTCCTTGATGAAATGGATTGTAGCTGATGATGCCTTTCCTAACCTTGAACAATTGGTTTTGCGTGGATGGCGACATCTTGAGGAGATCCCTTCTTGTTTCATGGACATCCTTTCTCTCAAGTACATCGAGTTAGAAAACTGCAATGAGTCGGTTGTCAAGTCAGCCATGAATATACAAGAAACACAAGTCGAAGATTATCAAAATACTAATTTCAAGCTCCTCATCAAGGTACACTACTGA

>PGSC0003DMG400003380

ATGTATTTCAACAAAGAATTATCTAATCTGAAAGATCGACTCCTTAGCACGGTGCAAGATCAAAATTACTCGGATGTTACAAGGGATCGAATAAATTTCTTTCTATGGGACTTAAAATTCCTCGATTGTTTTCTCCATTTGAAAAGGTTGCCTTTTGCAAGTGAATGCGGTATGCTAAAAGTCTCACAGAAAATCATAGAAATTTGGAAGTTTCAGTGTAAACCCTATGATTGTCCAGATCTATTTCCGTACAAGACTCTGTATGTTTACGATTCATTTGGCTACTGGAAGGATGTAATTTGGAAGACTAAGCAAGAATTCAGAGCTGAATACTCTTTTCCAAATACATCACTTGCAGCCAACAAGGTGGATGATGATGTAGCCCCAAATATGGAAGTCATCGATGTTTTTGTGGAGAACCTCAATGTTCTAGTGAAGATCAATGATCCAATTTCGTGGCTTTTTGTTCCAGAACACAAGGAACAAATAGAACAAGTGTTAAAGGAGTTGAAGTTATTGAGATTTTTTGTCTGCTTTGTTTCAAACAAATGTATAGAGCCTCAATACCGACATACTACTTTTTATATTCATGCTTTAATTGAGGCTAGCCACATTGCAATGGTTGTCTGGTTGCATTTGCCAGTCGTCTATGGAAATCAAGACGTGGCTCCAAGTGAAGTCAGTCGTTTGCTCTCTGATTTCATGGAATTGAAGATTAAGTCCATTCAGCCAGTCATCAGCCGCAACAATATTTATATTGATGTCCTGCAAGCTTTGAAGTCAACCATACCACAAGCACAAAAGAAGCATGCAGCTGAGAGTAGCACTGTGGAGATTCCAACACACAGTCTGACGGTTGGTTTGAGTGATCAAATGGCTAACCTTCAGGAGATGCTCTGCCTTCTAAGAGATAATCTCATTCATCTGCCAATACTAGATCTGGAATTTCATCTTCAAGATATGGATTCTGTTATTATTGATGCTGGACTTCTTATTTACTCATTATATGATATCAAGAGGGAGAAGGAAGACACAGTATTGGATGATATGAACCGAGCACTTGGTTTTGATCTTCCAAGAAACATTGAGCCTATCAAGGCGATGGTCTACCTCATCATGCAAAAGGCATTTCAATGTAACTTGCCAAGGATTCATGGACTAGGCTATGTCGATTTTCTATTGAAAAACCTGAAGGATTTCCAAGGCCGTTATTCAGATTCACTCGCTTTCCTCAAGAATCAAATTCAAGTAATTCAAACGGAATTTGAGAGCTTGCAACCTTTCCTGAAGGTTGTCGTAGAAGAGCCACACAATAAGTTCAAGAGACTGAATGAAGATTGTGCTATACAGATAATTAGGAAAGCACATGAGGTGGAATATGTAGTTGATGCTTGTATAAACAAAGGCATTCCTCATTGGTGCCTCGAGCGTTGGCTCCAGGATATCATAGAGGAGATTACTTGTATCAAAGCAAAGATTCAGGAAAATAACACTGTTGACGATACAATGAAGACTGTCATTGCTCGTACATCATCACAACTGGCAAGGACTCCGAGGATGAATGAAGAAATTGTTGGGTTTGAGGATGTCATAGAAAATTTAAGAAAAAAACTACTGAATGGAACCAAAGGGCAAGATGTCATTTCAATTCATGGCATGCCAGGTTTAGGTAAGACGACTTTAGCCAACAGACTCTATTCTGACAGGTCAGTTGTTTCTCAATTTGATATTTGTGCACAATGTTGTGTGTCTCAAGTATATTCGTATAAGGACTTGTTATTGTCCTTGATACGTGATGCTATTGGTGAGGATTCTGATCAGCATAGAGAACTTCATGACAATGAATTAGCTGATAAGCTTCGCAAAACCTTATTGCGCAGAAGGTACCTTATCCTTGTTGATGATGTGTGGGAAAATAGTGTTTGGGATGATTTAAGAGGTTGTTTTCCAGATGCCAATAACAGAAGCAGAATCATTCTAACGACAAGACATCATGAAGTTGCCAAATTTGCTAGTGTTCATAGTGATCCCCTTCATCTTCGTATGTTTGACGAAGATGAAAGTTGGAAGTTGCTTGAAAAGAAAGTGTTTGGTGAACAAAGCTGCTCCCCTCTCCTAAGAGATGTTGGGCTAAGAATAGCAAAAATGTGTGGACAACTCCCTCTTTCAATTGTTTTGGTGGCTGGTATTCTGTCAGAGATGAAAAAGGAAGTAGAATGTTGGGAGCAAGTGGCCAACAATTTGGGTACCCACATTCACAATGACTCAAGAGCCATTGTAGACCAAAGTTATCATGTTTTACCCTGTCATCTTAAGTCTTGCTTCCTTTATTTTGGAGCATTTTTAGAGGATAGAGTGATTGATATTTCAAGGTTAATAAGGTTATGGATATCAGAATCATTTATAAAAAGTTGTGAAGGCAGGAGGTTGGAGGATATAGCAGAAGGTTACTTGGAGAATCTTATTGAAAGAAATCTAGTAATGGTTACTCAAAGGGCCGATTCAGATGGTAAGGTCACAGCATGTCGCCTTCATGATGTACTACTCGACTTCTGCAAGGAAAGAGCAGCTGAGGAGAATTTTCTACTATGGATAAAACGGTATATTCACAGAGATCAGATTACCAAAGCTGTTTACTCTCACAAGCAGCATGCTCACTTGGCGTTCACTGAAATGGATAATCTTTTAGAATGGAGTACGTCTGGCTCACTTGTTGGCTCTGTACTTTTTAAGAATTATGACCAATACTTTGCCGATCGTCCCTTTTTCTCTCATGCTTTTGAAATTTCACACATTTTACCACATTTCAAGTTTCTAAAAGTACTGGATTTGGAACACCAGGTTGTTATTGATTTTATTCCGACTGAGCTCCCTTACTTGAGGTATTTCTCTGCGCTCATTGATCAGAATTCAATTCCTTCAAGCATATCCAATCTTTGGAACCTTGAAACTCTTATATTAAAGCGTAGATCAGCTGCCAGATATAACAGGGTATTACTACCTAGTACAGTTTGGGATATGGTTAAATTGAGATATCTGTATATTCCTTACTTCAGCCCTGAAATAAAAAGCAAAGCATTACTCGAGAACTCTCCAAAACTTGATGATTTGGAAACCCTTTCCAATTCATATTTCACTCGTGTTGAGGATGCAGAATTGATGCTGAGAAAAACACCTAATCTTCGAAACCTGACATGTAAAATCGAGTGCTTGAAATACCCCCATCAGTACCATGCATTGAATTTTCCAATACGGCTTGAAATACTAAAGCTTTATGGATCAAAAATCGTCAAAACCATCCCCTTTTACATCTCTGCACCAAATCTCAAATACCTGATACTCTGTGGCTTTTACCTGGATTCTCAGTACTTATCAGAAACTGCTGATCATCTGAAGAACCTTGAGGTACTCAGACTGTCCTTCGTTATATTTGGTGGTCATATGAAATGGAAAGTGAGCAATGGCAAGTTCCCTCAACTCAAAATCTTGAAACTAGAATATTTGCCCATGATGAAATGGATTGTAGCTGATGATGCCTTTCCTAACCTTGAACAATTGGTTTTGCGTCGATGTCATGATCTTATGGAGATCCCTTCTTGTTTCATGAACATCCTTTCTCTGCAGTACATCGAGGTAGAAAACTGCAATGAGTCGGTTGTCAAGTCAGCCATGAATATACAAGAAACACAAGTCGAAGATAATCAAAATACTAATTTCAAGCTCGTTCTCATCGAGGTACACTACTGA

>PGSC0003DMG400023062

ATGTTTTCCGGCGAATATTTGTCTTCAAAAAAACATCACCCGCTCGATTTTTTGCAGCGAATTGAAGACGAATGGGGTAGTTACATGCCGGAGACTATAAAGGAACGAATTTATTTACTCAAAACAGAGTTCAAATTCATGGATATTTTTCTCAGTATTCAGAGCTTCACTGATGAACCAAACATGCTAAAAAATGTCACTCAGAAAGTTCATGCTCTGTTTCAAGATTCCGCATTTGATGTATCAAAAATATATCAAAATCTCAATCGCCTTACTTCCCTGCTGCAGAACAAAATTCGGGTGACTAAGATGGAAATCAGAGCTAATTACTCCTTCTTCTCCGGAATTTCATTGAAATTGCCTTCGCTCGAAAAGAGTGGGGTTGATAATTCCAAGTTTGTAATGAAATTCATCAATGGTGTTGTACAGAATCTTCACGAGTTAGCAGAGATTGATGATCTCTGTTCTCGCGAAATACAGGAGGTTTTAAAAGAGCTGAAGTTGTTGAAGAGTCTTGTAGGCTTTTTATCAAACAGGTGGTGTGCTGAGCCTCAGAGCGTACGTACTTTCTTCGGTCATGTTCTAGTTGTGGCTGGGTTTGCAGCAATGGTTGTCTGGTTGTGCATGCCCAGCTATGACAATAACAGAGATCAGGATTTGGTTCCTGGTGAGATGAATTTCTTGCTTTCTTATCTCGTGCGGATGAGGATTAAGCCTGTTAATCCGTGCATCCACAAAATCTATGTTGATGTACTACAAGCTTTGAAATGGAAAATGCAATCAAATTTGTCTATGAATATCCAAAATGTGTATGTAGCTGAGATTGAAGCTGGCTTTGTGGAGACTCTCATGCACAACTTGGAGGAGATACGGAGTATTAGTACTCTTAGTCGGATAGAGTTTTTGAATCATCAAATGGCGACCCTTGTGGAGATGCTCAAATTCTTGAGAGCCAGTCTGATCCATTTGCCAACACTGGGTCTTGAATTTCATCTTAAAGATATTGATACTGTCATCATTGATGTGGGACTTCTCGTTTACTCGTTATACGATAGCAAGGAGCAGGAGGAAGTGAACCAAAGACTATTTATTGATTTGCCCAAAAGCATTCAGCATATCAAGGAAGTGATCTTCCTCGTGAGTCGAAAGGCATTTCAATCTAATTTGCCTAGGGTTCATGGACTAGGCTGTGTTGATTTCCTTCTGAACAACTTGAAGGAGTTCCAAGACCGTTATAAAGATTCACATTATTCTTTTGTTAAGAGCCAACTTCAAGTTATTCAGAAGGAACTCGAGGGCTTACAACCTTTTCTCAAGGATGTGGCGGAAGAGTGCTACAATAAGCATGAAAGGCTCCAACATTGTGCTGCACTATTAAATGGCAAAGCTTATGAGGTGGAATATATAGTTGATGCTTTTATTAGAAAAGGAGTTCCTGAATGGTGTCTTGTTCGTTGGCTCTTCGACATCATAAAGGAAGTTATACTTATCAAGGAAGAGGTCACAAAGATTCAGGAAAAGGAACTTTTTAAGTTTGCCTTTGTATTACATGATACCTTGGATACCACCCCTGCTCATATATCATCAGAATCGACTAACACTCCAAGGATGACCGGTGAAGAAATTGTCGGTTTTGAAGATGTTATGGAAAAACTAAGAGAACAACTAATAAGAGGAACCAAACAGCTAGATGTTATCTCAGTTGTTGGAATGCCTGGATTAGGCAAGACAACTGTGGCCAACAAACTGTATTCTGACGAGTTAGTTGTCTCTCGATTTGATATCCGTGCAAAATGTTGTGTATCCCAAGCATATTCACGTAGGAGCGTGTTACTTTCCATTCTACGTGATGCCATTGGTGAGTCTCCTACTCTTACTAAATTGTCTACGGATGTATTAGCAGATCAGCTTCGCAAAACTTTATTGTGGAAAAGATATCTTATCCTTGTTGATGACATATGGGAAGCTAGTGTTTGGGATGATTTAAGATGTTGTTTCCATGATTCCAATAATGCAAGTAGAATTATCCTAACAACACAACATGCTGATGTTGCTGAAAATGCTAAATCTGTTAGTGATCCACTTCATCTTCGTATTCTTAATGATGATGAAAGTTGGAAGTTACTAAAACAGAAAGTGTTTGGTGAAGAAAGCTGCTCTGTGCTCCTTTCAAATGTTGGGCAAGAAATAGCAAACAAGTGTAGGGGGCTGCCTCTTTCAATTGTTTTGGTGGCTGGTATGCTAACAAAGATGGAGAAGAGTGAACAATGCTGGAAACAAGTGGCTATGAATTTATGTACTAACGTTCTCAGCAACTCGAAGGCCATCATAGAGCAAAGTTATCAGAACTTACCTTATCATCTAAAGCCTTGCTTTCTTTATTTTGGAGTATTTTTGGAGGACAAAGAAATCAACATTTCAATCTTGACATGGTTATGGATTTCAGAAGGATTTATAAAAAGTCGTGATGACAAGAGTCTGGAGGATATAGCAGAAGGCTACTTGGAGAATCTTATTGGAAGAAATCTAGTTATGGTTGCTAAATGGGGTTCCGGCGGTAAGATCAAAACATGCCGTATTCATGACCTGTTGCTTTATTTCTGTAAGGAAAGAGCCAAGGAGAAGAATCTTCTACTGTGGATGAAACGGAGGGACCAAAATGTCAATACGTCTTCCTCTATTTACTCTCACAAGCAGCTTGTTCAACGTCGCATGTCCATCAATTCTCAAGTGGTTGATCTTGTGAAATGGAGCTCACTTGTTGGCACTGTACGTTGCAGGGAAGATAGAAACAAAGGTTCCTTTTCAATTGTCCAATTCTCTCACATTTACTTCAGATTTCTAAAAGTGTTGAATTTGGAGTTCATTGTAATTGATTCTTTCCCAACTGAGCTAGTTTACTTGAGGTATTTTGCTGCACGAACTTCTCAGAAGTCAATCACATCGTCCATAGTCAATCTTCGAAACCTTGAAACTTTGATAGTCAAACCGATGGGAGGAAAATTGATATTGCCACTTACACTTTTGAAGATGGTTAAATTGAGACATCTGCAGATATATAGCAAAGCTCATTTTTCCACTTTAAATGCTGCAGAAGAATTGCTTGAGAACTCAAAATTCGACAATTTGATAACTCTTTCCTCTCCAACATTTTGTTGTGTGAGGGATGCAGAATTGATGTTGAGAACACCTAACCTTCGAAAACTGAGATGCTCATTTGTTGGTTGGGGTTATCCTTCTCATGTAATGAGCTCCCTAACACGGCTTGAGACACTCAGTATTAAAATGGATTCTTGCGGAAGTTCTCCATCCAACTTTCCACCAAATCTCAAAAAATTGACTTTGTCGAACTTTACCATGTATTGGCTGCAATCAAGCATTGCAATGCTTCCAAACCTTCAGGTACTCAAGCTGGTAGCAGTATTTTTCTCAAAGGCCGAATGGGAAGTGACGAGTGACAAGTTCCATCAACTCAAAGTTTTGAAAGTAGTTGATTGTCCTTGTTTTAAAAAATGGAATGTCTCTGACGATGCTTTTCCTCGCCTTGAACACTTGGTACTGAGAAGATGTCGATATCTCGAGGCAATCCCTTCTCGCTTTGGAGACATCACATCTCTAATATCCATTGAGGTGAAGTCATGCAAAGAATCGCTTGTCAAGTCAGCCATGGTCATCAGGGAATCACAAGTTGAAGAGATGCAGAATTATGATTTCAAGGTCTTCATCCACAAGTAG

>PGSC0003DMG400025615

ATGGAACTGTATGAAGGAATAAAAGTTTTGTTGATGAAGAGATTCTTAATCCTCATTGATGATGTATGGGACACAAAAGCATGGGACTATTTACATATGTGCTTTCAGGGTATTAAAAATGGTAGTAGAATAATCCTAACTACCCGGCTCTCTGAGGTGGCGCAATATGCTAAATGTGAAAGTAATCCCCATGATCTTCCTTTACTGAGAGATGATGAGAGTTGGAAGTTATTACAGAAAAAGGTTTTCCATGGAGACAACTGCCCATCTGAACTTGGAGATGTGGGCTTTCGGATAGCAAAAAGTTGTGGCGGATTGCCTCTCTTCATTGTGCTAGTAGCCGGTGTTCTGAAAGAGAAAAATAACAAAGCAGATTTGTGGAAAGAAGTAGAAGAAAGTCTAGATGCACTGAACATCGATAGCTTGGAAGAGAGCATGTCGATAATTGGATTCAGTTACATGAATTTACCACATCATCTGAAGCCTTGCTTTTTGTATTTTGGAGGATTTTTGAGGGGCAAAAGCATTCACGTCTCGAAATTGACAAGGTTATGGTTGGCAGAAGGTTTCGTACTTGAAAATAAGGAAAAAGGACTAGAAGATGTTGCACAAGATTTCTTGAAAAACCTTATTAGTAGAAATCTAGTCATGGACATGGAGAAGAGGTTCAATGGCAAGTTGAAAATGTGTCGTGTTCATGATCTATTGCACAAATTCTGCTTGGAGAAGGCCAAACAGGAGAATTTTCTGCTCTGGATCTATAGGTACAAAAGAACTATATATACTACTCACTCTGTTTCAATTTGTTTGTTTGGTTTTGACTTGATAAAAGTTGCCAATGCCACCAGTGATGATCAGTATACTACAATGGCGTGTGATATCTCCTTCATCCTTGACAGCTTCAAACTTGTTAAGGTATTGGATTTGGAATCAATCAACATTGGTTATACTTGGAGGTTCCTAAAGTTACTAAACCAAGCATTTGAAGGAGTTCAGTGGAATGTGAATGATACAGAGTTCCCTGAACTCAAATACTTAAAATTGGACAGCCTCAACTTTGCTCAATGGTCGATTTCTGAGGATTCGTTCCCTAGTCTTGAACGATTGGTTTTAACAAACTGTAAGAGGCTTGAGAAAATCCCTTCTCATTTTGAGGACGTTGTTTCTCTAAAAAGCATTGAAGTAAATTGGTGCAGCTGGTCTGTTGCTAATTCAGCAGAGGAAATTCAAACAACGCAACGTGAAGATATGGCAAATGATGAGTTCACAGTTACTATACAACCTCCTGATTGGGATAGAATATCATCTCCTTGA

>PGSC0003DMG400025611

ATGGCTGCAGATTCTTGCAATGCAGTTATAAAAGGTATAGACTCTGTGAAGAGTAGACATTTGGGAAATTCGGTGAATAATGAATTGGAGGGTGCTAAGAAACAGCTAGAGTTTGTAGCTGAATTTCTCAAGCAATTGGAGAAACGTACCCCCGAGAATAGGCTTTCTGCACAAATTGAGTCCTTATTTGAAGAAGCTCACAATGACTTTTATGAGATATGGTGCCATATGAATAATGAAGGCCGGACAAAGGTCACAATCAGAATGATTTCAAAGGTGCTGAAAAAACTAAAACCGGCGTTTATTGCTAGGCGAATCAAAGATTCAAAGCCATTGAGATCAACTATTGGAATTACTGTCGAGATGATGACATTTGTAGATTCTTTGCTTGAGAGTGTTCTGGTTCTATGGAACTGTATGAAGGATTTTATCACCCCTCGCATCACTAAAGTCGAAGTGCTTGAAAAGAAGCTGATATCTCTGAGATTCCTCATTTTTACTGCATATTCTTGCGTTTATGAGGACGAGACAACGATGAGTGATCTCTTCACCCATGCTGAGGATGTAGCTTACACTGCTGCACACTTATCTTTCTTATATTTGGACCCGGAAAGTGTGGTGCATTCTGAGTTCTCTAAGCTGTTGGAAACAGTAAGTCCTTTTGGACCAGAATTGAGACAGATTTATACGTGTGTCCTAAAATGCCGGTCAAATTTATCAGGATCAAAAACTCCAATGATAGCTCATGAAGTTGTGGAAGGTTTTCTTTGTTCTCTCCGAGAGGACCTAGAAGAACTGCTAAGTCGTGACGACAGCTTGAAAGTGGCCTTTGATGATCATATGCAATGGCTCCAACAAGGATTGGTTTACCTTGGTACTTTTCTTCTGAACTTGCCAACACCATGCACTGAGGACCAAAAGCGATTTTCTCTTCTATCACATATTGAAGATGTGGCTAGTGAGGCGGCAATTCTGATCTACACCTTGTATGATGAGGATGTGGACAAGACTACTCATTTTCCTTTGCAAGTGAAGTTTAATCATGTCAAGATAGAGGGTGAGATGATTAAGCTGCATGAAACAACCGCGGTGGATTCTTTGAAGGATCTGATTGATGAAGTTCAACAAGAGCTGATGTTCTTGAGAACTTTTCTCATGGATTCTTTGCAGCAGTGCATAGAGCAAGCTAAGATAACTGATGTCTTGTCACTGGTTCTATATGTAACCACCGAAGCAGGATCTGTAGTTAATTCTCTTTCAAATGATTTGGAGCAAGAAGATTTGGTCAGGGAAATGGATATTGCATATTGTCAATTGATTCTTAAGTTTAAATTTGTCAATGCAGTAATCAGACAAACGTGTCCCGTCATTTTTGATTCATCGGAATCAAACCATCCTATGAGAAAGCTTCTGGACTTTCTTCCTATTAACTTTGATGTCATTGATTCATATTTCAGCATGCTAAAATCCTCAAAGACATCATCCTTTGGTAGCCCGAAGATTGGTGAGGTTTTGATGGGGTTTCTTGAATATATTCTCGATCATCTCCAAGAGCTGCTGAATGATGAAGGCAGTTTGATTGTTGCTGCTACAAATGAGGTGAAAAAGTTCTACCAGGGATTGTTGCTTCTAGTAACGTTTTTCATTGACACCTCGATCCAGTACACTGAATGTGAGAGACAAAATTATCTCTTGACTGAAATTGAAACAATAGTGATTGAGGCTGAATCTGTTGTCAATTCATTGTTTAAGACTACTGAAGTAGAGCATGTGCTTTTCCGTTTGCAAGTTAAGCTTAATCTTATCAAGGTAGAGAGCGGTTTGATTGAGCTACGGAAACATGAAGCCACTGTGATTTCTCCTTTGAAGGATCTAATTGTCAATGTCAAGGATGAGCTGATATTCTGGAGAAGTTTTCTCATGGATTCATTGGAGCAGACCAAAGGGAAAACTAAAATTACCATGCCTTTTTCCGTGCAAATAAAGCTTAACCATATAAAGGTAGAGAGCAGTTTGATTGAGCTACTAAAACATGGCGCCAACATGATAGCTCCTTTGAAATGTCTGATTAAAGATGTGCAGGAAGAGCTGATATTCTTGAGAACTTTTCTCATGGATTCATTGGAGCAGTGCGTAGAGCAAACTCAAATAAGTGATGTTTTGACCATGGTTCAATCTGTGACCACTGGAGCAGGATTGCTTATTAGCTCTCGTTACTTCAATTCAAATCAAGGAGACTTGGACGGAGAAATCAATCTCTTGCATTTCGCATTGCTTCTTATGTTCAAGTTCATTAAGGCAGTGATTAGACAGATGTGTCCCGTTATTTATGCATCCACGACAGTGATTGATCATCCTTTAATAAATCTGCTGAACTTTGTTCCTATCAACTTTGTGATTATTTATTCTTACTTTAGCATCCTCAAATCCACAAAGACAATATACTTGAGAAGCCCCAAGATGGATGACGTATTGATGGTGTTTCTTGACTATATTCTCAACAATCTCAGTGTACTACTGAAGGATGAAACCAATTTGTTTGTTACTGCTGCAAATGAGGTGAAAAGGTTTTACCAAGGGTTGTTGCTTATAGTAACATTTATTGCTGATCCCCCAAGTCAGTACATTGAATGTAAGAACAAAAATGACCTCTTGATGGAAATTGAAACTATCGCAATTGAGGCTGAATTTGCTGTCCGTTCATCTTATGAGGATCATTCAATGCTTTTACCTTTGCAACTGAAGCTCAATTGTGTTATGGCAGAGAGCAGTCTGACTAAGCTACTTAAACATACCGATATGGATCCTTTGAAGAATCTGATTGTCAATGTCAAGGAAGAGTTGATATTCTTGCGGACATTTTTCATGGATTCATTGGAGCAGTGCCAAGGGGAAACCAAGATAACTCTGCAATTTCCTTTGCAGGTGAAGCTTAATCATGTCAAGGTAGAGAGCAGCTTGATCGAGCTACAGATACATGACGCCACCTTGATGGCTCCTTTGAAGGATCTGCTTGACAATGTTAAGCTAGAGCTGATATTCTTGAGAGCTTTTCTCATGGATTGGTTTAACAAGTGCAAAGAGCAAACTAAGATAACTGATGTTCTGACCCTGGTACAATCTGTCACTACCGATACAGGATCACTCATTAATTTTCTTTCTCATAATTCAAAGCAAGGAAACTTGTTGTGGGAAATTAGTCTCCTGCATTTCGGATTGTTTCTTAAGTTTAAGTTCATCAAAAGAGTAATTGGACAGATGTGTCCCATTATTTCTGCATCATCAACTCCAAATGATGCTGAGATAAATCTGCTGGAGTTTATTCCGATTAACTTTGAGGTCATTCGTTCTTATTTCAGCAAGCTGAAATTCTCTAAGACATCATTCATAGGAATCCCAAGGATGGATGAGTTTTTGATGGAGTTCCTTGAATATATTCTAGACAATCTCCGCGAGTTACAAAAGGATGAAGCTGATTTGAGATTTCATGAGGTGAAAAAGTTTTACCAAGGGTTACTGCTCATAGAAACATTTCTTGCTGATCTCCCTGTTGAATGCAAGAAACAACAGGAGCTCCTGACAGAAATTGAAAATATCGCAATTGAGGCCGAAACTGCTGTCAGTTCCTGTTGTGAGAAGACTACTGAAGCAGACCATGTGCTATTTCCTTTGCAAGTGAAGCTTAATCATATCAAGGTAGAGAGAAGTTTGATTGAGCTAACAAAACATGAACACTTGGTAGCTCCTTTGAACGATATGATCGAGAATGTTAAGCAGGAGTTGATATTCTTGAGAAATTTTCTCATGGATTCATTGAATCAGTGCAACGAGCAAACAAAGACAACTGATGTTTTGTCCCTGATACAATCTGTTACCACCGAAGCAGGATCTGTCATCAATTCTCTCTCTCGTAATTCAAAGCAAGGAGGCTCAGCTAAGGAAATAAATCTCTCACATTTCCAATTGCTTCTTAAGTTTAACTTTATCAAGGCAGCAATTAGACAGATGTGTCCTATCGCTTCTGCATCGTCATCATCGAACCATCTCATTATGATAAATCTGCTGAACTTTTTTCCTTTTGACTTTGAGGTCATTGATTCATATTTCAGCATGGTTAAATCCTCAAAGACATCGTCCTTAGGTAGCTCGAAGATGGATGAGATTTTCATAGGGCTTCATGAGTACATCCTTGACAGCCTCAAAGTTTTACCAAATAATGAAACCAATGTTGTATTTACTGATAAAAGGAAAATGGTTTACCAAGGGTTGTTGCTCCTGGTAACATTTCTTGTTGATCCCCCAAATCAGTACATTGAATGCATACAGCAAAATGATCTCTTGACAGAAATTGAAACTATTGCACTCGAGGCTACAGATGTTATCCGTTCATCTTACAAGGATGTTGTAAATAGAAATGAAAGTAAGAAAGTCAATCTAGAGATCAAGCTTTTGACAGTGGCTTTCAAGCTTATCAAGTCTGAGGGAAACTTGACACATCTGCTAAAGCACAAAGCCACGTTGGAAGCTCAAATTATAGCTCTGATTGAAGATACTCATGAAGAGCTTGTTTTCCTCAGAGCTTTTTTTATAGATCTTCTCAGGCAACACAAAGAGCTTTACAAATTGCATGATCTTCTAGTGCATGCTGAAGTGACTACCCACAATGCAGTACTAATCAGTGGTTCTTGTTGTGAGGAAATGAGTCTTTCATTAGTTATGTTGCTACGTGAGATCAAGTCCGTCAAGGCAGAGGTAAGAAGCGTATGCTTTGAAGATTTGGATGCATCACCTTGCAACATGACTAAGACAAATGTAGAAGGCCTTGTCAAGTTTTTACTAAACAATCTGGACAGGGTGTTCACCTGTGATGCTGGTTCGATCCCTTTTATGAAGAATCAAATACCTGTCGTCCAAGAAAATCTGGTGTGTTTGGGCTCTTTCCTTGAACATATTGTACAGCACCGCGACATGCATAGGGAACTCAAGGACCTCATAGAACGTGTTCAAGAGGTCGTTAATAGTTCAAAGTATGTCATTTTCTTCTCTGTCAGTTGTGATAATCCTGTTTGGTATCACTTGTTATATCTTTATGATGTCAAACAAGTGCTTAAGTTTGTTGAAGAAGAGGTGAAAATGATTTGTTTTAAAGTCCCAGACTCTTCACTCTTTGGCTTCTCCAAGACCAGTGGATTAGGATTCCTCAATTGTTTTTTGGGCAAGTTGGATGAGCTGTTACATTCTAAGCTTGAGTTAATTACCGAATTGAAGCATCAGATCGGATCAGTCAAGGAAGAGTTGATACATCTAAGATCTTTTCTCAGTCATTTTTCAGAGAACAATGGTGAGCATGATGATGTTTATGGTCTTGTAACAAGTGTTACTGAAATGGCATACAAAAGTGAGTATGTTATCGACTCTTGTTTGTCGATTTCCTACCCACTCTGGTACAAAGTTCATTGGATTTCTGAAGTTGTCGAGAATATAAAGCTTCTTAATAAAGATGTTAGTGAGATATTTGGAAGAAAGCATATTGAAGTGACATTGCATAAAGTTGCAAAGACTTCCACTTATCTTATTGAGCCATCTTTATTAGCTAATACTCTGACAGAAAATAAAGAAATGGTGCTTTTCCAGGATGTGATGGAGAAAATAAAAAAGCAGCTACTTGGTGGATCGTCGCAGCTAGACGTTATCTCAATAGTAGGCATGCCTGGTTTGGGCAAGACTACTCTTGCCGAGCAGATTTACAATGATCCGATAGTTGCTGGTTACTTTGATGTTCATGGTAAGTGTCATGTGACTCAATCATATTCATGGAGAGAATTGTTGCTTACTCTTTTGAATGATGTTGAGCCTTCTGATCACATGAAGAAAGCAGATGATCAATTAGCTAAAGAGCTGCGTCAAGTTTTGTTGACGAAGAGATTCTTAATCCTCATTGATGATGTATGGGACACAAAAGCATGGGACTATTTACATATGTGCTTTCAGGGTATTAAAAATGGTAGTAGAATAATCCTAACTACCCGGCTCTCTGAGGTGGCGCAATATGCTAAATGTGAAAGTAATCCCCATGATCTTCCTTTACTGAGAGATGATGAGAGTTGGAAGTTATTACAGAAAAAGGTTTTCCATGGAGACAACTGCCCATCTGAACTTGGAGATGTGGGCTTTCGGATAGCAAAAAGTTGTGGCGGATTGCCTCTCTTCATTGTGCTAGTAGCCGGTGTTCTGAAAGAGAAAAATAACAAAGCAAATTTATGGAAAGAAGTAGAACAAAGTCTAGATGCACTGAACATCGGTAGCTTGGAAGAGAGCATGTCGATAATTGGATTCAGTTACATGAATTTACCACATCATCTGAAGCCTTGCTTTTTGTATTTTGGAGGATTTTTGAGGGGCAAAAGCATTCACGTCTCGAAATTGACAAGGTTATGGTTGGCAGAAGGTTTCGTACTTGAAAATAAGGAAAAAGGACTAGAAGATGTTGCACAAGATTTCTTGAAAAACCTTATTAGTAGAAATCTAGTCATGGACATGGAGAAGAGGTTCAATGGCAAGTTGAAAATGTGTCGTGTTCATGATCTATTGCACAAATTCTGCTTGGAGAAGGCCAAACAGGAGAATTTTCTGCTCTGGATCTATAGGTACAAAAGAACTATATATACTACTCACTCCGTTCAATTTGTTTGTCTGGTTTTGACTTCTTTAATTCACATGAAGTACTTTGCTGCTCGGACTGGAGCAGATTCAATTCCTTCATCTATAGCTAAGCTTTGGAATCTTGAAACGCTCATTTTAAAAGGAATGCGAGGACAGGTAACATTACCATGCTCACTTTTGAATATGACTAAGTTGAGGCATATACATGTAAATGACCGTGCTTCATTTAATCTCGACAATATGAGTGAATCCCTTGCTAATTCACAGTTAGCTAACTTGCAAACCTTTTCCACTCCATACGTCTCTTATGGTGAAGACGCGGAGATTATATTGATAAAGATGCCAAATTTGACAAAGCTAAAATGTATAGTTGGTTGCTCAGGGAAATGGAGGGGAGAATGTGTTCTGATTCCTAGATTAGACTTTCTAAGTCGTCTCGAATCCCTTAATTTGTTTTCCAACAACTGTCCAGTTGAATGTCTACGAGGATTCAATTTCCCTTCGGAGCTAAGGGAGTTAACTTTGTCAAATTTTTGTCTACCTTGGAGCGAAATCTCAATCGTTGGAACATTGTGCAACTTGGAGGTTCTAAAGTTACTAAACAAAGCCTTTGAAGGAATTCAGTGGAATGTGAATGATACAGAGTTCCCTGAACTCAGATACTTAAAATTGGACAGCCTCAACTTTGCTCAATGGTCGATTTCTGAGGATTCGTTCCCTAGTCTTGAACGTTTGGTTTTAACAAACTGTAAGAGGCTTGAGAAAATCCCTTCTCATTTCGAGGACGTTGTTTCTCTAAAAAGCATTGAAGTAAATTGGTGCAGTTGGTCTGTTGCTAATTCAGCAGAGGAAATTCAAACAACGCAACGTGAAGATATGGCAAATGATGCGTTCACAGTTACTATACAACCTCCTGATTGGGATAGAATATCATCTCCTTGA

>PGSC0003DMG401022603

ATGGAGGAATTCGAGCAGCAGGCAATTACTGCTTCAATCCCATCACTCCGGCTGTATTATGACATTTTTTTAAGTTTTAGAGGCGAAGATACTCGAGACAACATCACAAACAACTTATACAACGCCTTATATTCAAAAGGCATTCGAGTTTTTCGAGATTCCGAGGGGTTAACTCAGGGCGATGAGATCTCAACAGGTTTAATAGAAGCAATTAATGATTCTGCTGCTGTAATTGCTATAATTTCCCCAAATTATGCTTCCTCGAGATGGTGCCTCGAGGAATTAGCAACGATCTATGAGCTGGGTAAACTCGTTCTGCCTCTGTTCTATGGAGTTGACCCGTCGGATGTACGGAGGCAGCTAGGGCCATTTCTCGATGGATTTAGGGATTTGGAAAGGAAATTTTCACCGGAAAAGATGGTGAGATGGAGAAACGCCATGGAAAGAGTTGGAGGGGTCTCTGGTTGGGTTTACGATAATGGGGTTAACTTGATCTTAATTAATTTGCTTCCATTCTACAGTGACGAGTCACAGTTGATTCAGTTGGTGGTGCAAAGAGTTTTGAATGAATTAAGCAATTCCCCAATGGTTGTAGCTCCGTTTGTTGTTGGAATTGATTACAGTCTGAAAGAACTCATAACACAGTTGGATGTCAAGGGCAACGGTGTCAAAATCTTGGGGTTGCATGGAATAGGAGGAGTTGGTAAAACAACTCTTTCTAAGGCTCTTTACAATAAACTTGCTTCTGATTTTACACACAGGACTTTTATCTTGAATGTTAAGGAAATAGCTACTCAACAAGGCATTATGTCCCTTCAGAAGAAAATCATACAGGGTCTTTTCCCTAGCAACGCGTTCTCCTTCTCCCCTGCTAATGCCATTGAGGGAAGAGAAAAGTTCAGGCGAATGCTTCGAGAAAAGCGCATCCTGCTGGTCTTAGATGATGTTGATTATGTAAATGATGATGTAAACATACTAAAGGCTCTAATTGGGGGTAAAAATTGGTTTTTTGAAGGAAGCAGGGTTGTCATTAGTACTAGAAACAAAGGAGTTCTGATAGAAGATATCGTTGACGAGACATTTGAGGTGAGAGAATTGGGTGATACTGACTCACTAAAACTATTCAGTTACTATGCATTTAGAAGACCAGACCCATCCCCAACTTTTCTGAATTTATCCAAGCAAATTGTCTCAATTACTGGAAAGCTACCATTGGCTCTTGAAGTTTTTGGTTCTTTCTTGTTTGATAAAAGAAGCGAGGAGGAATGGGTAGATGCTCTAGGAAAGCTAAAACAAATTCGCTCACCCCGTCTTCAAGACATCTTGAAAATAAGTTACGATGGTCTTGATGATGAAGAGAAGTGTATATTCCTGGATGTTGCATGTTTATTTCTTGATCAACTAGATAAGAAAGTCGAAAATGTAATTGATGTGATGGAAGGATGTGGTTTTAGAGCCAGGATTGCATTTGACACATTGACCACTAGATCATTGGTTAAGGTGATTGATGGTGGCGACTTGTGGATGCATGACCAGATAAGAGATATGGGAAGACAGATTGTTAGGGAAGAAGGCTTTTCCGAACCTGGAAAACGCAGCAGGCTGTGGGATGTTGCTGATGTTTTAAGTGTGCTACAAGGAAGGAAGGTAACTGTGCAGGCGGCCAACAAGTTGAAAGTTATGAATATCTCTGATTGTCATAAAATATCAGCTATTCCTGATTTATCAAAGCATAAAATGTTGGAAAAGTTGATAGCTGAGCGTTGCAGCAACTTACAAAGGATTCACAAAACAGTTGGGAATCTGAAAACTTTACGTCATCTAAATCTAATAGATTGCCGCAACCTTGTTGAATTTCCAAGTGAGGTCTCTGGGCTGAAAAATCTTGAAAAGCTGATACTCTCAGGTTGCGAAAAATTGAAACAGCTACCTGAAGATATTGGCAAGATGAAGTCTTTACAAGAACTTCTATTAGATGAGACTGCTATAGAGAAGTTGCCTTCAAGTATATTTCGCTTAACAAAACTTGAAAGGTTAAGCTTAAACCACTGCTACTCATTGAAACAACTTCCCGGGTTAGTAGGAAATTTAAGTGCTTTGAAGGAACTCTCTCTTAATGGTTCTGCTGTGGAAGAAATACCTGATTCTATTAAAAATTTGGAGAACCTTCATACACTAAGCTTAATCAGGTGTAAGTCACTTGCTGCTCTTCCCCATTCTGTTGGCAACCTCAAATCTTTAGCAAATCTCTGGCTTTATGGCAGTGCAATAGAAATTGTACCAGAATCTATCGGTTGTCTATATTATCTTAGGTCCTTATCGCTTGGAAATTGTCAGCAGTTGACTGCATTGCCTGTTTCAATTAAAGGATTGGCTTCTTTGGTTGAGCTTCAAATAGAGAAGGTTCCGATCCGTAGTCTTCCACATGTTGGAGCCCTTAAATCACTGAAGACTCTTGAGATAAGGAACTGTGAGCGCCTTGGCTCGCTACCCGATTCCATTGGAGAATTATTAGCTCTTAAAACAATGACTATTACTCGAAATGATGCTATCACGGAGCTGCCAGAATCAGTTGGAGAGTTGCAGAATCTTGTCATATTGAGATTGACTAACTGTAAGCGACTGCACAAACTGCCAGATTCTATTGGGAAACTGAAGAACTTAGTACACTTGCTAATGGAGGAGACTGCAGTAACAGTATTACCTAAAACTTTTGGGATGCTATCGAGCTTAATGATTCTGAGGATGGGAAAGAAGCCTTTCTGTCAGGTATACAGTGGTGAAAATTAGTATAAATATTATATTTTGAATCCACATTTTCAAAAAATTACACTTATGCAGGTACCACAAAGTACTGAAATCACAGAAACAGCTACCTATGCAGAAAGGGAAACCGTACCTATTGTGCTTCCTTCATCTTTCTCAAAGTTATCCTGGTTAGAAGAACTTAATGCACGTGCATGGCGAATAGTTGGGAAAATACCTGATGATTTTGAGAAACTATCATCTTTGGAGTTCATCAATCTCGGTCACAATGATTTTTCCCATTTGCCGTCTAGTCTTAAAGGACTACATTTCTTGAAAAAGCTCTTAATTCCCCACTGCAAACAACTGAAAGCTCTTCCTCCTCTTCCTTCAAGTTTGCTCGAGATAAATGCTGCAAATTGTGGAGCACTAGAGAGTATGCACGATATCTCAGAATTAGTGTTCTTGCGCGAACTAAACCTCGCGAATTGCATGAGTTTGGTCGACGTCCAAGGTGTCGAATGCTTGAGATCCTTAAAAATGCTACATATGGTTGGATGCAACGTCTCTTGTGCCTCTATTGTTAGAAGGAAACTTGATAAGGTACTTTTTTCTCCTATATGCTTTTAG

>PGSC0003DMG400013506

ATGTCTTCTTCTTCATATGAATATTTGTCTATAGGGAAAGATTATGACCTTTCTCATTTAGAAAAGCTTGCCAATGAATGGGATTCCAGCCTCCCAAGAAATAAAGATCCAAAGAATCTAATTGAGATCATTTATATGTATCTTAAATTTTTAGATATGTTTCTCAGCTTGCAGAGCTTCACAACTGTATGTTGGTATGCTACACAGAAAGTTCAAGAAGTTTTTTCTTATGCTGAAACTGAAGTCAAAAGAATCAACCATCCCGGCGATGTTGCATCCGTGTTCTTTAAGTTGGAAGAACACATTTGTGAGACTAAGTTGGAAATCAGAGATAAATACTCATTTCCCGAAGCATCACTACCACTTTCCTCCATCGTTGCTACTCCCGAATTTGCAAAGAAATTCATTGATACTGTTGTAAGGAATCTCACTGATTTATCAGAGATTTGTCATCCTTATATTGACATAGTTCCTCACTCAAGCAAACAGCCAATGGAAGAGGTTGTAAAGGAGTTAAAGTTATTGAGAAATTTTGTCTACTTTATTTCAGATAGAATATGCTCGGATGAGTCGGATCCTCAAAGCCAACATACTTTCTTGACTCATGTTTTAGTTGTGGCTGGTCATGCAGCAATGATTTCTTGGTTGAATTTGCCAAACCATTGGAAAGAAAAAAACTTGGCTTCAGCTAAAATGAATGCTTCATTTTCCGACTTTCAGAAGAGGATTCAGCCTATTCAACCATGTATCCGCAAGATCTATGTTGATGTCCTGCAAGCTCTAAAATCAGGATGCCATCCAAATATTGAAGCTGAGTATGTTTCTGATTATAAATATGGCTTTGTGGAGACTCTCGTACACCGTTTGAAAGTGCTACCCGTTACTTTCAAGGATCAAATGCCAATGCTCAACTTCTTGAGAGACAATCTCATCAATCTACCAAGAGAGGCCTTTAAAGATCTTGATACCGCAATTATTGATGCAGGGCTTCTAGTTTACTCGTTGTATGACCGCGTGGAGGAGAAGGAAGACGTGGCTGTTGGGGAGTCAAATCAAGTGCCAGTTCTTGATTTCTCAGGAGATATTCCGAGTATACAAGCACTGATCTACCTCATCAGTCGGAAGTCATTTCACTCCAAATTACCTAAGATTGATGGACTGGGATCTATTGATATCATTTTAGACCACCTGAAGGAGTATCTAAGCCTCTATTCAGACTCACTTTCTTCTATAAAGAGCCAACTTTGGACAATTCAGCAGCAATTGGATCACTTTCAGAAGCATCACGATGGATCGTTCCAGTATTTTTCAATGCAGGTGATTTCTATAGCATATGAGGTGTATCATTTGGTTGTCGGTTGCATAAATAATGATATCCCTGAGTGGTGCCTTGTTCTCTGGATTGGAGACATCATAGAGGAGATTACACTACTCATGAAGAATCATGATGAAAAGAAAGTTGCTGACTTGGTATTGCATACTACCACTGATGTTGTCAGTGCTCATACGTCACAACTTACTCGGATTACAAGTATCAGAGAAGAAATGGTGGGTTTTGATGAGGTGGTGCAAACACTCAAGGGGAAGCTAATCAAAGGATCATCAAAATTGGATCTTATCTCAATTGTTGGAATGGCCGGATTGGGTAAGACAACTCTAGCTAACAAACTATTTCTTGATCAGTTAGTTGTTTCTCATTTTGATGTCCGTGCACAATGTTGTGTATCTCAAGTATATACACGTAAAGACTTGTTACTAACCATTCTTCGGGGTGTGAAGAAGGATACAGTTATCAGTGATAAACTACCAGAGAATGAATTGGCAGATAAGTTGCGTAAACTTCTATTTGGTCAGAGGTATCTTATCCTCATTGATGATGTCTGGGAAACTACTGCATGTGATGATCTAATGCCTTGCTTCTATGAAGCCAATAATGGAAGTAGACTTATCCTGACAACTCGCCATGATCATGTTGCCTACCATGCTAAACTCGTTAGTGATCCTCATTTTCTTCGAAAGTTTACTCTTGAAGAAAGTTGGATACTATTGAAGAATAAGGTGTTCAACAAAAAAAGTTGCCCCGCTGTCTTAGAAGATGTTGGCCAAAAGATAGCACAAAAGTGTGGAGGGCTACCTCTTTCAGTTGTCCTGGTAGCAGGTATTCTCGAAACGATGGAGAAGGAAAAACGTTGTTGGGAACAAGTTGCAATAAATTTAGGTCCACACATCCAGGCTAAGTCAGAGGATATAATAAATCTTAGTTACCAAGATTTACCATTTCATTTGAAACCTTGCTTTTTGTATTTTGGAGTATTTTCGGAAGACGAAGAGATAAAGGTATCAAAGATAACATGGTTGTGGACAGCAGAAGGTTTGGTAAAAACTCATAAAGAGAAGCTTTCAGAAGATATAGCAGAAAATTACTTAAAGAACCTTATTGGAAGAAACCTTGTGATGGTTTCCAAGAAAAGTTCCAATGGTAAGACCAAGACATGTCGCATTCATGACTTGTTGCTTGAGTTTTGTAAAAAGAAAGCCAAGGTGGAGAACTTTCTATTCTCTATTAACAGGGACAATGATAATATGAATCTTTCTTCTACTTCCTATCAGAAGCATAGCATTCAACGACGCTTATGCCTTCAATTTCAAGTTGATAATCTTGCAGAATGGAGTTCGATTTGTTCAGATGTACAATCTTTCCACTTGATGAAGGGAAGGAAAATTGGATTATCTTCAATTGGTTATGCATCACACACTTTTAACAGTTTCAAGTTCCTGTGGGTGCTAGATTTAGAATTCACGGTAATTGATTCTTTCCCAGAAGAGTTAACCTACTTGAGATATGTTGCTGTTAAAGTTGCTGAAGATTCTTCATTGACATTCTCGGACAATCTTTGGAACCTTGAAACTTTAATAGTTAAAGGACTTGGAGGACGAGTAACTTTACCAGATGCCATCTGGAAGATGGTCAAGTTGCGCCATTTGCACGTATACAAACACGCTATTTTCAATATAAACAATGCTCTCCAAGAGATGGATGGATTGAGAACTCTTTCCTCTACATGGTTTCCTTGTGTGGAGGATGCAAACAGGATCTTTGCAAAGACACCAAATCTTCAAAAACTGAGATGTGAAGTTTTGTCATGTGATGGCTTTTTCCCTACATTTAACAATCTTGAAATGCTCAAGTTTTCTTGGGGTCGTTTGGGGATATGGGCCACTGAGCTGAATTTGCCACCAAGTCTCAAGAAATTAACACTATCCAATGGTCGCGTATCTAGACTTGATCAGGTCGCAATCCTCCCCAGACTTGAGGTACTCAAACTGCTAAACGTTTCCATTAAATACGAAGAATGGAAAGTGACCGATGAGGATTTCCCTCACCTGAAATTCTTGAAACTGCAAGATCTTTCTTTTTCTGAATGGGATGTTTCGGATGATGCTTTTCCATACCTTGAGCACTTGGTGTTAAGAAGATGTCAATATCTTAAGATGATCCCTTCTTGCTTCGGATACATGATGTCTCTAAAATCCATTGAGGTAAAGTCATGCAAGGAGGAATCACTCGCCGAGTCAGCCTTGGAGATCAAGAATATGCAAGTTGATGAGATAGGAAATTCTGATTTCCAGGTCTTCATCCACAAG

>PGSC0003DMG400013486

ATGGCCGAAGATTCTTGCTTTATATTAAAAAAAAGCATCAGCGATTGTGTAAAGACGGGGCAGTTAGATAGTTCAGCCAAGGATCAATTGGACTATGCTAGGGCGATTGTAGATAATTTATGGTATTATTTCGGTTGGCTGAAGAAGGAAAACTGTAAGAATATGGTATCTGCGCAACTTAAGCCCTTAATCGATGAAGCCAATGATGGCTTTTGTGAGATACATTCTCTTTTGAAGAATCAAGGCCTGACAAGTGAGACAGTCAAAATGATTTCAGAGGTGATGAAAATGATCAAACCTGAGATTATTGCTGAGCGATTTATCAGTCCTTCCAAGCAATCAACATCATCATCCACCCGTATGATCACTATGGATATGGTGAACGTCGTCACAAGTATTTGCCATACTGTTGAATATCTGACAAAGATTACATTTCGGGAGTTTAAACATCTGGGAGTTCTCCTCAAGTTTACCGCAAAGCGGTGCAATATTGAACATGAGAAAATAAAGGATCTCTTCACCCTTGCTGAGGATATAGCTAAAACTGCAACTTACCTATCTATCTTGTGTTGGGAGACGTATGACCAGTACTCGGCACGTATATCAGCGCCCAAGTCTTCTATTTGGGTAACATATAATGTGTTCGCTGGTCCTGACTTCGAGTCCAAGATTTCAAAATTGCTAGAAAGGATAAATCCTATTAGGCCTGAATGGAGGAAGCTTTATATCAGTTTCCTGAAAGCATCACATTCGTCAGCTCCACTGATGCATGGTGGTCTTAAGAATCTATCACATGATCTTGATCTTGATCTTGCTCAGAAGTTTACTGAGTCTATCAGATACGATCTGGAAAAGCTGAAAAGTCATGATGCCAGTTTGAATGTTGCTTTTTCCAATAGATTTGAATGGCTCCAAGATGGACTTCTTCATCTCTCTGAATTTCACAAGATATTAGATCGCCAGCGACAAGACTTGAGTTCATTGCTCTCATTTCTGGAAGTTCTGGCCATAGAGGCAGCAATTGCAATCTACTGCTTGTGTGACATAGACTTAGAGAACAATATTGCTGAAGTAGACCTTATATTTCTTCCTTTGCAAATGAAGTTTAAGTATCTCAAGGTAGAGATCAGTCTGTTTCAGAAACTAAGGTTACCAGAGTCCCAGGTGGACTGCTTTCAGGAAGAATTGACTTCCATGAAAACGTTCCTCATGGATTCATTGGACAAGTGCAAAGTGCAGACTCAGCTAACTGATGTCTTGACCTTAGTTCTATCTGTCACCACCGAAGCAGAGTCATTCACTAATTCTCTTTCTCGTGATTCAGAGGATGGAGCATTAGCCAGGAAAATTAATCTCTTGCATTTTCAATTACATCTTAAATTTAAGTTTATTAGGGAAGCGATTTGTCCCTTCATTTCTGCATCATCAACACCAGTTCTGCCTGTGATATATCCGTTGAACTTTCTTCCTACTTATGTTGAGGCCATCAGTTCTTATTTCACCATGCTGAAATCCTCAAAGACATCGCCCTCCGCTGGTAGCCCGACGATGGATGAGGTTTTCATGGAGTTTCATGAATATATTTTTGAAAATCTGCTACTAAAGGTTGAAGCAGATCTGGAACTTACTGATACAGATAAAGTTAAAAGGTTTTACCACAGGTTGATGCCCTTGGTAACACTTGTTGTTGATCCCCCAATGCAGTACATTGAATGCAAGAGGCAAAATGATCTCTTGAGAAAAATTGGAACTTTAGCGATTGAGGCCGAAGCTGCTATCCGCTTATCGTATGAGGATGCTTTGGACAGAAGCAAAAGTAGGGAAGTCGGTCTTTTGCTTCAACTTTTGGCTGCGTCTTTCATGCTAATCAAGTGTGAGGGGAAGTTGATGGATCAACTAAAGCACAAAGCTATTCTTGAAACTGAATTTTTGGATCTTGTGGCAAATGCTCATGAAGAGATTATTTTCCTTAGAGGCTTTCTCATGGATCTTCTCAGGCAACACACGACAAAGCTTGACAAATCAGATGATCTCTTAATGCATGCTGAAGTGGCTGCCCACAAGTTTAGCACTTGTAGTTATGAAAGTTTTGTGGATGGAAGCAACACAGGGAAAATATCTGATTTCCTACAGGAGACCGAGTTTGTCGAGGTAGATGCATCACATGTATTGCCCTTAGTTCTATCTGTGGCCACTGAAGCACACTCATGCATTACTTCTCTTTCTCGTGATTCAGAGGAAAGAGAATTGATGAGCAAAATTAATCTCTTGCACTTTCAATTGCTTCTTAAGTTTAAGCTTATTAAGGCAGCGATTCGGCAGATTTGTCCATCCTTTTCTGCATCAACACCAGATCATCATGTGATATATCCGCTGAACTTTCTTCCTACTTACTTCGAGGTCATTGATTCTTATTTCACAAAGTTGAAATCCTCCTCTGATAGCCCGAAGATGGATGAGGTTTTGCTAAGGTTCCATGAATATATTTTCGAAAATCTGCTACTAAAGGATGAAGCTGATCTGAAACTTACTGATTCAGATAAACTCAAAAGGTTTTACCACAGTTTGCTGCTCTTTGTATCAATCCTTGTTGATCCCCGAATTCAGTACACTGAATGCAAGAAGCAAAATAATCTCTTGTCGGAAATTGGAGCTTTAGCGATTGATGCCAAAGCTGCTATCCGTTTATCATATGAAGATTCTTCGCAAAGCAACAGGAGTAAGAAGGTCAATCTTTTGCTTCAACTTTTAACTGTGGCTTTCATGCTAATCAAGTGCAAGGGGAAGTTGACGGATCAACTAAAGCACAAAGCCATTCTTGAAACTGAATTACTGAATCTGGTGGAAAATGCTAATGAAGAGCTAATTTTCCTTATAGTCTTTCTCATTGATCTTCTTGGGCAACAGACGATAGAGCTTAACAAATCAGATGGTCTCTTAATGCATGCTGAAGTGGCTGCCCACAAGTCAACATTAATTAGCACTTGTAGTTATGAAAATTTCGTGGATGGAAGTAGCTCTGCGGATATGAGTCTTTCATTATCCGATTTCCTAAAAGAGACCAAGTCTGTCAATGCAGAGATTAGAGAAGTATGCTTTCAGTTGCTGGATGAATCAGCCTCCTATATCACTGTGACAGATCTGAAATGCCTTATCAACATGTTATTGGACATGCTGAACCATCTGCATTCTAGGGGTGACGTGATCCCGGTCGTTAGGAATCAAATCCCAGTAGTTCAAGAGAAACTGGAGTTTCTTGCTGATATTTTAAAGCCGTGCAATATGCATACAGAACTCAAAGACCTTATGGAACGTGTTCAAAATGTTGCCTATGGGGAAAAGTATGTCATTTTCTTCTCTGTCAGTGGTGATAGTCGTGCTTGGTTCCACCAGTTATATCTCTATGATGTCAAACAAGTGTTTAATTTTGTTGAGGCAGAGGTCAAAACTATTACTTCTGAATTTCATGAAGTGACAGGGCTCAACTTCCCCAAGACCGATGGATTAGGATTTCTCAATTGTTTCTTGGGAAAGTTGGAGGAGCTGTTACACTCTAAGCTTGACCTAATTACCAAACTGAAGCCTCAGATTGTATTAGTCAAGGAGGAATTATTGATTCTAAGATCATTTTTCGATCATCCGGAAGAAACCTATGATGAGCATGATGAAATTTGTGGTCTTATAATCAGTGCTACTGAAATGGCATACAAAGCCGAGTATGTCATTGACACTTGCTTGGCCTGTTCTTACTCACAGATGTACAAAGCTTATTGGATATCCGAAGTTGTTGAGAATATTAAGCTTGTCAATAAAGATGTTGGTGAGAACCTCAAAAGAGAGGAGATTGATGTCAACCGAGTCGCAAAAGGTTCCACTAATATTGTACCATCATTATCAGCTAATACTTCAGGAGCAAATGAAGAAATGGTGGGGTTTCAAGATGTGATGGACAAATTAAAGAAGCAGCTTCTTGGAGGATCGCATCAGCTAGATGTTATCTCGATATTTGGTATGCCTGGAAATGGTAAAACCACTCTTGCAAAGAAGATTTACAATGATCCCACAGTTGTCTCTCACTTTGATGTTCGTGCTATGTGTCATGTGACTCAAGTATATTCATGGAGAGATTTGTTGCTTACTATTTTGAATGATGTTCTTGAGCCTGCTGATCGCACCAAAAAAGGAGATGACGAGTTAGCTACTGAGCTGCGTCGTGTTTTATTGACCAAGAGATTCTTAATTCTCATTGATGATGTGTGGGATAAAACAGCATGGGACGATTTGAAAATGTGTTTTCAAGGTTCTCAGAATAGGAGTAGAATTATTCTGACAACGAGGCTGTATGAGGTTGCCGATTATGCTAAATGTAACAGCGATCCCCATCCTCTGCGTTTACTCACAGATGATGAGAGCTGGAAGTTACTACAGGAAGAGTTGTTTCATGGTCAAAGCTTCCCATGTGAACTCGGAGATGTGGGATTACGAATAGCGAAAAGGTGTGGAGGGCTGCCTCTCTCAATTGTCTTAGTAGCTGGTGTTCTCAAAGAGAAAAAGAAGAAAGCAGATTGCTGGAAAGAAGTAGAAGAAAGTCTTAGTTCACACAACATTGGGAGCTCAGAAGAGAGCATGTCTATAATTGGATTCAGTTACAAGAATTTACCAAACCATCTAAAACCGTGTTTTCTCTATTTTGGAGGATTTTTGAGGGGCAAGGATATTCCAGTCTCGAAATTGTCACGAGTGTGGTTAGCAGAAGGAATTGTTGAAGACAGTAAGGAAAAAGGATCAGAAGATGCTGCCCAAGATTACCTGAAAGATCTTATTAGAAAAAATTTGGTAACGGATATGGAGAAGAGATCCAATGGAAAATTGAAAACCTGCCGTGTTCATGATTTGTTGCATCAATTCTGTGTGGAAAAGGCCAAGCAAGATAATTTCCTGTTCTGGATACATAGGTACAAAAAATTATATCGATTTTACTCTGATAACCTTCATCACAATTCTCATTTTCATGTTTTTTCTTTGAACAGTGGCCATGGTGTGGATTCCATTTCTTATCCTGAAAAGCCCGAGATATACCGCTTGTCTATATATTCTAAATGGGATGACTTTGCTCAGTGGCAGCAGGCTGGATCAAGTGTTCGTTCTTTGCTATTCAATGCCAGCAGTGATGATTATTACCCTGCAATGGCACATAATATCTCCTTCATCATTAACAGATTCAAACTTGTTAAGGTGTTGAATTTGGAATCCATCAACATAGGTGATACCTTTCCTAATGAATTAAAATCTCTAATTCATATGAGGTACTTCGCTGTTCGAACAACTGCTGATTCTATTCCTTCATCCGTAGCTGATCTTTGGAATCTTGAAACTTTCGTGGTTAACGGATTGCATAGAGTGTTGAAGTTACCTTGTTCACTTCTCAAGATGTTTAAATTGAGGCATGTACATGTAAATAGTCGTGCTTCATTCAGTCTGCACGATAACATGTGTGAGTCACAATTAGTTAATTTGGAGACCTTTTCCACTCCATGTCTCTCCTCTGGTGAAGATGCAGAGAAGATATTGAGAAGCATGCCAAATTTGAGAAAGCTGAGATGCATAGTTGAGGGATTATTGGGTTATTCAACTAAAGGAAGCATTGTTCGTTTTCCTAGATTAGACTTTCTACATCAGCTTGAATCTCTGAAGCTGCTTTCGTACAGTTATCCAACTAAACATCCTCACGAATTCAATTTCCCCTTAAATCTGAGGGAATTGACTTTGTCGAATTTTCGTCTACCTTGGACCCAAATTTGGACAGTTGGCAAACTTCCGAACTTGGAGATTCTAAAGTTACTTTTCAGAGCCTTTGAAGGGAATGAATGGGAAGTGAAAGATTCGGATTTCCCTGAACTCAAATACTTAAAATTGGACAACCTCAACATTGCAGAGTGGTCTGTCATGGATGATGCTTTTCCTAAGCTTGAACATTTAGTGTTAACGAAATGCAAGAAGCTTGAGAAAATCCCTTGTCATTTTGGGGATGTTGCATCTCTAAACATTGAAGTAAACTGGTGCAGCTGGTCTGTTGCCAATTCAGCTCAGGAATTTCAAACAACACAACATGAAGATATGGCAAATTATGCGTTCAGAGTTACAATACAACCTCCAGATTGGGACACAAGATCATCTCATTGA

>PGSC0003DMG400013490

ATGGCTCAAGAGTGTCGCGCAGTGATAGGTGCCATAGACCTTCTGCTGGGAAGGCATTTAGATAGTTCCATAATTAATCAATTGGAGAAGACTAGAACTCAACTAAAATGTGTATTAGGATTTCTGGTAAAACTGGAGAAGGTTTTCCCTGAGAACACGATATCCACACAACTAGGGGCCTTATTTCAACAAGCTCATGATGGCTTTTCTGAGATATGTACTCACATGGATCAACTGAAAATAATTAAACTGAACAACATTGCTGAGGTGCTGAAAATACTTAAACCCAAGAATATTGATGAAAGAATCAAAGCTTCAAAGCCATCAACGTCATCTAGCCAAATCACTACTATAGAGATGGTGAGGTTTGTCGATATTTTGCTTGATCATGTAAAGAATTATGTCTTTGTGAGGCTGCATTATCTACACGCTTTCTTCATATTAACTGCAAGTCGGTGCATTAAGCATGAGAGTATGCCGTATTTCTTCATCTATATTGAGAATGTAGCTTATACTGCACTACGACTATATTTCCAATGGATGGAAGAATATACAGATTTGCAACATGAGTTGCGCAGTTTGATAAGTTCTTCAAGTCCTGATTTGAGGCAGATACAAAAGAACCTCTTGACAGCGTTAAAATCATCAAGATCAGAGACTACATTGAAGTCCGGATATATGCTTGATTTTGTTAATGCTGTACGAGATGATCTGAAGGTGCGTCGTGCACACGGTGATCAAATTGAGGGGCTCAAAGAAGGACTTCTTAACAGTTTTCAATTATTACAGTACAATGAATATGGAGGCACTCAAATCATTGAAGCTCTGGCCTTTGAGGCAGCATTAATGATCAACTCCTATGATGAGGATCTTCTTTTTGTTTTGCAGCCGAAGCTTAATCATGTCAATGTAGTAATCGAACTGATTCAACTACGTAAAAGTCAAGTAACCTCTCTCCTGAGAGCTGATAGTCTTCTACTGATTGACTATGCTTTGGACGAGCTGGTGGTCGTGACAACTTTTCTCACGGATTCATTGGAGCAGTGCAAACAGCAACCTAAGATAACTGATCTTTTAACTCTTATCCAATCTGTGACTAACGAAGCATGTTCAGCTGTTAAAAATCTTTTATGTTATGCAAGACGAGAAGACTTGGTCTGGGAAATGAATCGGTCACATTTTCAATTGCTTCTTAAGTTCAACTTTATTAAGGCAGCAATTAGACAGATGTGTTCCACCATTTGTGCTTCATCAACTGAGATACATCTGCTGAACTTTCTTCCTATTAATTTTGAGGTCATTGGTTCTTACTTAAATTCCTCAAAGAAATCATCCTCTGGTCACCACAACATGGATTTACTTATGATGGGGTTTCATGAATATATTATTGACAATCTGCTACTGAAGCATGAAACTGATTTGTCATTTACAGTTGCAGATGATGTTAAAAAGTTCTACGATGGATTGTTGCTCCTGGTAACATCTCGTGTTGATCTTCTTGATCCTCTATCTCAGTACAGTGGATTTGGAACTATTGCAATTGAGGCCGAATCTGTGAATAGCAACAAAAGTAGGAAGAGCAATCTAGTTCTTCAGTTTTTGACTGTGGCTTTCAAGCTTATCGAATGTGAGAGAAGCTTGATGAATCTACAGAAGCACAAAGCCACTTTGGAAGCTCACATTCTGGATATGATTGAAAGTTCTCATGAAAAGCTTATTTATCTAAGAGTTCTTCTCATTGGTGTTGTCAGGCAACACACAGTCCTTAATGAATTGCATAATGTCTTAATGCATGCTGAAGTGACTGCTAACAAGATAGCACAAATCATCAAGGGAAGAAGCATTGAGGAAATTGGGCATTTATTATCTGAGATTGAGTCTGTCCATGTAGAGGTCAGAAAAGTATGTTTTCAATTTCTGGATGCATCACCTTACAACATGACAGATGGGGAAGGCCTTATTAGATTCTTATCGAAATACCAGGACTGGCTGCTCAACTTTGATGCTTGTTCAATTCCTTTTCTGAAGAACCAGATCCCAGTAATCAAAGACAAACTATTTTATTTGGGTTCTTTTATTGCAGATATTGTACAGCATCGCAAGATGCATCAAGAGCTCAAAGATCTTGTGAAACATGTTCAAGATATAAAGTTTGTCTGTCTCTTTCCCATCAGGGATAATGCACCTTCTTGGTGTTACGGGCAATATCTCTCTGATGTCAAGCAATTGCTTAAGTTTGTCGAGACAAAGGTCGAAGCGATTTGTCTCAAAGTTCCAGATTCTTCAAGTCATAGCTTCCCCAAGATAAATGGACTAGGATCACTCTATTGTTTCTTGGGAAAATTGGATGAGATGCTAAGTTCTAAGATCGATTCAGTTATCGACTTAAAACTTCAAATTGGGTCAGTGAAGGAGGGTTTATTGTGTCTAAGAACATTGACTGATCATTTCCCAGAAATCAACGATGAGCATGATGAAGTTTATAGTCTTATAACAAGAGTTACTGCAATGGCATACGAGGCAGAGTATGTCATAGACTCGTGCTTGACCTATTCTTATCCACTCTGGTACAAAGTTCTTTGGATTTCTGAATCTGTTGAGAATATTAAGCTTGTAAATGAAGTTGTTAGAGAAACATGTGAAAGAAAGAAGATAGACGTGATGGTGCACAAAGTTAAAAAGACCTCAACGAATCTTGTGCCGTCTTTATCAGCTAATAGTGAAGGATCAAATGAAGAAATGGAGAGTTTCCAGGAGGCGATGGACCAAATGAAGAAGCAGCTACTCCAAGGTTCACGTCAGCTAGATGTAATCTCATTGGTCGGCATGCCGGGAATCGGTAAGACTACTCTTGCCGAGAAGATTTACAATGATCCAGTAATCACCTCTTGGTTTGATGTCCGTGCTCAGTGTCGCGTGACTCAAGTATATTCATGGAGAGGTTTGTTGCTTGCCATTTTGAGTGGTGTGCTTGAGCCTATTGATCGCAATGAAAAAGAAGATGGCGAATTAGCTGATGAGCTGCGGCGATTTTTGTTGACCAAGAGATTCTTAATTCTCATTGATGATGTATGGGATGATAAAGTGTGGGACAATATACATATGTGCTTCAAAGATGCTCGGAATGGGAGTAGAATTATTCTAACAACACGTCTGAGTAACGTTGCCAATTATGCTAAATGTGAAAGTGAACCTCATCATCTTCGCTTGTTCAGAGATGATGAGAGTTGGACATTATTACAGCAAGAGCTGTTTCAAGGGAAGAGTTGTCCGCCTGAAATTGTTGATGTGGGGTTTCGAATAGCAAAAATATGTGGAGGGTTGCCTCTCTTCATTGTATTAGTTGCTGGTGTCTTCAAAGAGAAAAAGCTAATAAAAGCAGAATTGTGGAAGGAAATAGAGGAAAGTCTATGTTTGCTGAATATTGATAGCTTGGAAGAGAGCATGTCTATAATTGGATTTAGTTACAGGAATTTACCACAACAGCTGAAGCCTTGTTTTCTCTATTTTGGAGGACTTTTAAAGGGGAAGGATATTCATGTCTCAAAATTGACTCGGTTGTGGGTAGCTGAGGGTTTTGTACAAGCAAATGAAGAAAACGGACTAGAAGATGCTGCAGAATGTCTCTTGGAAGATCTTATTAGTAGAAATTTAGTCATGGGCGTGGAGAAGAGACCCAATGGAAAGCTCAAAACATGTCGCATTCATGATCTGTTGCATAAGTTCTGCTTGGAGAAGTCCAAACAGGAGAATTTCCTTCTTCATATCAATGGGTATAAAGAACTATACCTGATTTTATCATGTTATTGTTTATATCAACTCTCATTTTCATTAATTTTTTCTTTGAACAGATTCACTGGAGAGGATTCATTTCCTGAAATGTCTATGGATTACCGGTTGTTTGTTCATTCTTCTGAGGATCAGATTGATCAGTGGCAGCCATCTCGCTCAAATGTTCGCTCTTTGTTATTCAATGTGATTGATTCAGATAACTCAATCTTTCCGCGTAATATCTCTTTCATCTTTGACAGCTTCAAACTTGTTAAGGTGTTGGATTTGGAATCCGTCAACATTGGTGGTACTTTTCCTAGTGAAATACAATTTCTAATTCATTTGAAGTATTTTGCTGCTAAAACTGGTGGAAATTCAATTCCTTCATGTATAGCTAATCTTTGGAATCTCGAAACTTTTGTGATAAGAGGATTGGGAGGAGAGGTGATACTACCTAGTTCACTTCTAAAGATGGTTAAAATAAGGAATATACATGTAACTCATCGTGCTTCATTTAGTTTGCATGAGAATATGGGTGAATCACTTGCGGACTCTCAATTAGATAATTTGGAAACCTTTTCCACTCCACATTTCTCTTATGGTGAAGATACAGAGATGATATTGAGAAAGATGCCAAAATTGAGAAAGCTGAGTTGCATATTTTCGGGGACATTTGGCTATTCAGAGAAAGTGAAGGGAAGGTGTGTTCTCTATCCCAGATTAGAGTTTCTATGTCAGCTTGAATCCCTTAAGGTTGTCTCCAACAGCTATCCAGAAAAACTTCCACATGTCTTCAGTTTTCCCTCAAGACTTAGGGAATTGACTCTGTCAAAGTTTCGTCTACCATGGAGCCAAATCTTGAGTATTGGAGAACTTCCTAACTTGAAGATTCTAAAGTTACTTCTCAGAACCTTTGAAGGGGATGAATGGGAAGTGAAAGATTCAGAGTTCCGTGAACTCAAATACTTAGAATTGGAGAACCTCAACATTGCACAGTGGTCTGTCTCCGAGGATGCTTTCCCTATTCTTGAACGTTTGGTTTTAACCAAATGTAAGCGGCTTAAGAAAATCCCTTCTCATTTTGACGACGCTGTATCATTAAAAAGCATTGAAGTAAACTGGTGCAGCTTGGGTGTTGCTAATTCAGCCAAGGAAATTCAAGCGTTTCAGCATGATGAAATAGCAAATGATGCATTCAAAGTTACTATACAGCCTCCAGATTGGGATAGAAATTCATCTCCTTGA

>PGSC0003DMG401013522

ATGGCTCAGGAGTGTCTCTCAGTGATAGGTGCTATACACCTTCTGAAGGGAAGGCATTTAGATAGTTCCATAATTGACGAACTGGAAAATGCTAGATATCGACTAAAATGTGTGTCTCGATTTCTCATGAAACTGGAGGAGGTTCTCCCTGAGAACACGATATCTACACAACTAGGGGCCTTATTTCAACAAGCTCATGATGGATTTTCTGAGATATGTACTCACATGGATCAACATTACACCATCAAAATGACAAAGGCGCTGAAAAAAATTAAGCTGGAGAACATTGCTGAGCGACTCAAAGCAGCTTCAAAGCCATCAACATCAACTAGAGGAATGATATCAGAGGTGCTGAAAATACTTAAACCCGAGAATATTGCTGAAAGAATCAAAGCTTCAAAGCCATCAACATCATCTAGCCAAATCACTACTATGGAGATGGTGAGGTTTGTGGATGTTTTGCTTGATTATGTAAAGGATTGTGCCCCTGTGAAGCTGCGATATCTACAAGCTTTCTTCATATTAACTTCAAGTCGGTGCATAGAGCATGAGAGTATGCAGTATCTCTTCACCCATGTTGAGGATGTAGCTTATACTGCACTACAACAATATTTCCTACAGGTGGCCTACAAAATCAATTCAAAAATTGAGTTGCATAAATTAGTTAGTCCTTTTAAGCCTGATTTGAGGCAGATATATATAAGTCTCTTGACAGCGTCAAAGTCATCAAGATCAGAGACTACAATGAAGTCGGGATGTATGCTTGATTTTGTTAATGGTCTACAAGAGGATCTGGAGGAGACGCCTCTAGACCGTGATGAAATTCGGTGTGTCGGAAGAGGACTTTATTGCCTTTTCATATTTTTAAAGGACTTAAAATATACAGGCACTCCACTCAAAGAATTCAGTTCTCTTCAATCACTTATTGAAGCTCTGGCCTTTGAGGCAGCATTTGTGATCTACTCCTGTGATAATAAGGATCTTCTTTTTGTTTTGCAGCCAAAGTTTAATCATGTCAGTGTAGTAATCGACATGATACAGATACTAAACAGTCGAGCAACCATGACAGCTCCACTGAAAGATCTGATTGACTATGCTTGGGAAGAGCTGATAGTCGTGAGAACTTTTCTCATTGATTCATTGGAGCAGTGCAAACAGCAACCTAAGATAACTGATTTTTTAACTCTTATCCAATCTGTGACTGACGAAGCATTTAAAGTTTTGTATAAACTTTTAGTTTATACAAAACAAGAAGACTTGGCCTGGGAAATGAATCGCTCACATTTTAAATTGCTTCTTATGTTCAACTTTATTAAGGCAGTAATTAGACAAATGTGTCCTACCATTTCTGCTTCATCATCAACTGAGATATATCTGCTGAACTTTCTTCCTATTAGATTTGAGGTCATTGATTCTTATTTCTTGTCATCCTCTGGTCACGACAACATGGATTTAGTTATGATGGGGTTTCATGAATATATTATTGACAATCTGCTACTGAAGGATGAAACTGATTTGTCATTTACAGTTGGAGATGGCGTTAAAAATTTCTACTATGGATTATTGCTCCTGGTAACATCTCTTGTTGATCCTCTAGCTCAGTGCAGTGGATTTGCAATTGAGGACGAATCTGTGAATAGCAACAAAAGCAATCTTGTTCTTCAGTTTTTGACTGTGGCTTTCAAGCTTATCGAATCTGAGACAAGGTTGGGATATGTACTGGAGGGCAGTCTGCACAAAGCCAGTTTGGAAGCTCATGAAGAGCTTATTTATCTTAGAGTTCTTCTCATATATGTTCTCAAGCAACACACACAGCTTAATGAATTGCATCATATCTTAATGCATGCAGAAGTGACTGCCAAAAAGATAGCAAAAATCATCAAGAGAATAAGCATTAAGGAAATTGGGCTTTTATTATCTGAGATTAAATCTGTCAAGGTAGAGGTCAGAAAAGTATGCTTCCAGTTTCTGGATGCATCACCGTACATCATGACAGATGGAGAAGGCCTTATTGATTTCTTATTGAAATGCCAGGACGGGCTGCTCAATTATGATGCTTGTTCAATTCCTTTTCAGAAGCACCAAATCCCAGTAATCAAAGACAGACTAGTTTATTTGGGTTCTTTTATTGCAGATATTGTACAGCATCGTGATATGCATCAAGAGCTAAAAGACCTTGTGAAACATGTTCAAGATATAATGTTTGTCTGTCTCTTCCCTATCAGAGATTCTGCACCCTCTTGGTATTACAGGCTACATCTCTATGATATCAGGCAATTGCTTAAGTTTGTTGAGAAAGAGGTCAAAATGATTTGTCTCAAAGTTCCAGATTCTTCAAGTTATAGCTTCCCCAAGATAAATGAACTAGGATTTCTCGATTGCTTCCTGGGAAAATTGGATGAGATGTTAAGTTCTAAGCTCAAGTCAGTCATCGACTTAAAACATCAAATTGAGCCATTGAAGGAGGGCTTATTGTGTCTAAGAACGCTGACTGATCATCTCCCAGAAATCTACGATGAGCATGATGAAGTTTATAGTCTTATAACAAGAGTTACTGCCATGGCATACAAGGCCGAGTATGTCATGGACTCGTGCTTGACCTATTCTTATCCACTCTGGTACAAAGTTCTTTGGATTTCTGAAGCTGCTGAGAATATTAAACTTCTAAATAAAGTTGTTAGGGAAATATGTGAAAGAAAAAAGATAGATGTGGCAGTGCTCAAAGTTAGAAAGACCTCAACTCATCTTGTGCCGTCTTTATCAGCTAATACTCCAGGATCAAATGAAGAAATGGAGAGTTTCCAGGAGGCGATGGACCAAATGAAGAAGCAGCTACTTGGAGGTTCGCGTCAGCTAGATGTCATCTCATTGGTTGGCATGCCGGGAATTGGTAAGACTACTCTTGCCGAGAAGATTTACAATGATCCAGTAATCACCTCTCACTTTGATGTCCGTGCTCAGTGTCGCGTTACTCAAGTATATTCATGGAGAGATTTGTTGCTTGCCATTTTGAATGGTGTGCTTGAGCCTATTGATCGCAATGAAAAAGAAGATGGTGAATTAGCTGATGAGCTGCACCGATTTTTGTTGACCAAGAGATTCTTAATTCTCATTGATGATGTTTGGGATGATAAAGTGTGGGATAATATACATATGTGCTTCAAAGATGCTCGGAATAGGAGTAGAATTATTCTAACAACACGTTTGAGTAACGTTGCCAATTATGCTAAATGTGAAAGTGAACCTCATCATCTTCGCTTGTTCAGAGATGATGAGAGTTGGACATTATTACAGCAAGAGTTGTTTCAAGGGAAGAGCTGTCCTCCTGAAATTGTTGATGTGGGGTTCCGAATAGCTAAAAAATGTGGAGGGTTGCCTCTCTTCATTGTTTTAGTTGCTGGTGTTATCAAAGAGAAAAAGATAAAAGCAGAATTGTGGAAGGAAATAGAAGAAAGTCTAGGTTTGCTGAATATTGATAGCTTGGAAGAGAGCATGTCTATAATTGGATTCAGTTATAGGAATTTACCACACAAGCTGAAGCCTTGTTTTCTCTATTTTGGAGGACTTTTAAAGGGAAAGGATATTCAGGTCTCAAAATTGACTCGGTTGTGGGTAGCTGAGGGTTTTGTACAAGCAAATGAGGAAAACGTACTAGAAGATGCTGCAGAAGGTCTCTTGGAAGATCTTATTAGTAGAAATTTAGTGATGGATGTGGAGAAGTGA

>PGSC0003DMG400018619
[truncated: 1,232,751 more chars]
